# The gut microbiota in pediatric multiple sclerosis and demyelinating syndromes

**DOI:** 10.1002/acn3.51476

**Published:** 2021-12-09

**Authors:** Helen Tremlett, Feng Zhu, Douglas Arnold, Amit Bar‐Or, Charles N. Bernstein, Christine Bonner, Jessica D. Forbes, Morag Graham, Janace Hart, Natalie C. Knox, Ruth Ann Marrie, Ali I. Mirza, Julia O’Mahony, Gary Van Domselaar, E. Ann Yeh, Yinshan Zhao, Brenda Banwell, Emmanuelle Waubant, the US Network of Pediatric MS Centers, the Canadian Pediatric Demyelinating Disease Network

**Affiliations:** ^1^ Medicine (Neurology) University of British Columbia and The Djavad Mowafaghian Centre for Brain Health Vancouver BC V6T 1Z3 Canada; ^2^ The Montreal Neurological Institute McGill University Montreal Quebec H3A 2B4 Canada; ^3^ Center for Neuroinflammation and Experimental Therapeutics and Department of Neurology Perleman School of Medicine University of Pennsylvania Philadelphia Pennsylvania 19104 USA; ^4^ Department of Internal Medicine Max Rady College of Medicine Rady Faculty of Health Sciences University of Manitoba Inflammatory Bowel Disease Clinical and Research Centre University of Manitoba Winnipeg Manitoba R3E 3P4 Canada; ^5^ National Microbiology Laboratory National Microbiology Laboratory Public Health Agency of Canada Winnipeg Manitoba R3E 3R2 Canada; ^6^ Department of Laboratory Medicine and Pathobiology University of Toronto Toronto Ontario M5S 1A8 Canada; ^7^ Department of Medical Microbiology and Infectious Diseases Max Rady College of Medicine Rady Faculty of Health Sciences Winnipeg Manitoba R3E 0J9 Canada; ^8^ Department of Neurology University of California San Francisco San Francisco California 94158 USA; ^9^ Department of Internal Medicine Max Rady College of Medicine Rady Faculty of Health Sciences Winnipeg Manitoba R3A 1R9 Canada; ^10^ The Hospital for Sick Children University of Toronto Toronto Ontario M5G 1X8 Canada; ^11^ Division of Neurology Children's Hospital of Philadelphia Perelman School of Medicine University of Pennsylvania Philadelphia Pennsylvania 19104 USA

## Abstract

**Objective:**

To examine the gut microbiota in individuals with and without pediatric‐onset multiple sclerosis (MS).

**Methods:**

We compared stool‐derived microbiota of Canadian Pediatric Demyelinating Disease Network study participants ≤21 years old, with MS (disease‐modifying drug [DMD] exposed and naïve) or monophasic acquired demyelinating syndrome [monoADS] (symptom onset <18 years), and unaffected controls. All were ≥30 days without antibiotics or corticosteroids. V4 region 16S RNA gene‐derived amplicon sequence variants (Illumina MiSeq) were assessed using negative binomial regression and network analyses; rate ratios were age‐ and sex‐adjusted (aRR).

**Results:**

Thirty‐two MS, 41 monoADS (symptom onset [mean] = 14.0 and 6.9 years) and 36 control participants were included; 75%/56%/58% were female, with mean ages at stool sample = 16.5/13.8/15.1 years, respectively. Nine MS cases (28%) were DMD‐naïve. Although microbiota diversity (alpha, beta) did not differ between participants (*p* > 0.1), taxa‐level and gut community networks did. MS (vs. monoADS) exhibited > fourfold higher relative abundance of the superphylum *Patescibacteria* (aRR = 4.2;95%CI:1.6–11.2, *p* = 0.004, *Q* = 0.01), and lower abundances of short‐chain fatty acid (SCFA)‐producing *Lachnospiraceae* (*Anaerosporobacter*) and *Ruminococcaceae* (*p*, *Q* < 0.05). DMD‐naïve MS cases were depleted for *Clostridiales vadin‐BB60* (unnamed species) versus either DMD‐exposed, controls (*p*, *Q* < 0.01), or monoADS (*p* = 0.001, *Q* = 0.06) and exhibited altered community connectedness (*p* < 10^−9^ Kruskal–Wallis), with SCFA‐producing taxa underrepresented. Consistent taxa‐level findings from an independent US Network of Pediatric MS Centers case/control (*n* = 51/42) cohort included >eightfold higher abundance for *Candidatus Stoquefichus* and *Tyzzerella* (aRR = 8.8–12.8, *p* < 0.05) in MS cases and 72%–80% lower abundance of SCFA‐producing *Ruminococcaceae‐NK4A214* (aRR = 0.38–0.2, *p* ≤ 0.01).

**Interpretation:**

Gut microbiota community structure, function and connectivity, and not just individual taxa, are of likely importance in MS.

## Introduction

The human microbiome’s combined genetic load surpasses that of human genes with bacterial protein‐coding genes estimated as being over 300 times more abundant. Most (>90%) of the human microbiota reside in the gastro‐intestinal tract.^1^ Alterations in the gut microbiota may be influential in neurological diseases, including multiple sclerosis (MS).^2^ The gut microbiota regulates the immune system and contributes to the maturation and modulation of the CNS, including myelination, via multiple complex mechanisms.^3^ MS is considered an immune‐mediated and neurodegenerative disease, with the CNS being the primary target. While both genetic and early‐life environmental exposures are implicated in triggering MS, current knowledge surrounding these exposures remains incomplete. Animal models of CNS demyelination provide a proof‐of‐principle that the gut microbiota influence CNS‐directed immune responses.^4^, ^5^ Studies involving persons with MS, while still limited in size, suggest that compared with controls, subtle differences in key gut microbial taxa exist.^6^

The concept of a “period of risk” during which the inciting biology is triggered is important when considering risk factors for chronic disease. A symptomatic prodromal period, possibly extending for years before clinical MS onset in adults, has recently been recognized.^7^ Further, childhood and adolescence are key periods of risk exposure for MS. As such, analysis of the gut microbiota in pediatric‐onset MS patients represents a unique opportunity to examine pathological processes closer to actual risk acquisition. Children and youth have accrued fewer confounding exposures, such as medications and medical comorbidities compared to adults, permitting a unique window into the native gut microbiota.^8^

We compared the gut microbiota from stool samples of well‐characterized persons with pediatric‐onset MS and unaffected controls in a case‐control study, taking into consideration any prior disease‐modifying drug (DMD) exposure, and capturing key features seldom considered in MS studies, including other medications, dietary supplements, and stool consistency (the Bristol Stool Scale).^8^, ^9^ In addition, we included another disease group––participants with monophasic acquired demyelinating syndromes (monoADS)––to serve as an additional comparator to the chronic disease, MS. Generalizability of main findings was sought in an independent case‐control cohort of pediatric‐onset MS and unaffected controls.

## Methods

### Study design and participants

This case‐control study was embedded within two larger prospective North American studies of pediatric‐onset MS and related demyelinating diseases. Participants ≤21 years old who provided a stool sample and had monoADS or MS (McDonald criteria, 2017) and symptom onset (first clinical attack) <18 years or were an unaffected control were eligible. MonoADS was defined as an initial acute clinical episode of symptoms involving the CNS, with evidence of inflammatory demyelination and no new clinical or MRI findings of recurrent demyelination (median observation from first symptom onset = 9.1 years, range = 3.1–12.9 years).^10^ Unaffected controls had no known neurological or (auto) immune‐related condition (headache/migraine, asthma, and allergies were permissible) and were recruited using a mixed‐methods approach (e.g., via general pediatric clinic posters, and web‐based advertising), with the aim of enrolling age, sex, race, and geographical location representative individuals.

Informed assent/consent were obtained from participants/guardians. Ethical approval was obtained from each institution’s research ethics board.

The main and complementary analyses were conducted for the ‘Canada‐USA cohort’ which comprised MS cases, monoADS, and unaffected controls enrolled from four Canadian and one USA site (Children's Hospital of Philadelphia), between 11/2015 and 03/2018 through the Canadian Pediatric Demyelinating Disease Network. A second, independent “USA‐only cohort” comprised MS cases and unaffected controls enrolled from eight USA sites, between 06/2012 and 03/2018 through the US Network of Pediatric MS Centers was used to test generalizability of findings.

*Cohort characteristics* were captured for participants primarily through standardized forms and questionnaires administered to the participant/caregiver by trained coordinators at stool sample collection (details of data sources and categorization of variables are in Supplementary Methods). Briefly, these included demographics: age, sex, country of birth/residence, and race (white, non‐white); clinical: comorbidities, body mass index (BMI = height(kg)/weight(m)^2^), cigarette smoking (active or passive), medication use (*any* prior DMD use for MS, and, in the 30 days pre‐stool sample, any other medication/dietary supplement, defined using the World Health Organization’s Anatomical Therapeutic Chemical classification system, level 4 (Supplementary Methods)). Participants/caregivers completed the Block Kids Food Screener (NutritionQuest^©^)^11^ and, for the Canada‐USA cohort, the Bristol Stool Scale,^9^ adapted for children. The validated food screener captured the prior week’s diet,^11^ reported as the percentage caloric intake of protein, fat, and carbohydrate and total grams of fiber. The seven‐point ordinal Bristol Stool Scale captures stool consistency, considered a useful reflection of the gut ecosystem, and is associated with gut microbiota composition^9^ and was categorized into: hard (types 1–2); medium (3–5); or loose (6–7).

### Stool sample collection, sequencing, and bioinformatics

A common protocol was used for stool sample collection. The following were not permitted: antibiotics or corticosteroids within 30 days pre‐stool sample; any history of cytotoxic immunosuppressant use or major bowel‐related comorbidity (e.g., inflammatory bowel disease, IBD). The same collection kits were used for all participants, with stool shipped on ice before −80°C storage in the central laboratories (University of Manitoba IBD Clinical/Research Centre, Winnipeg, Canada or UCSF, USA), with all sequencing performed together (batched) at the National Microbiology Laboratory, Winnipeg. Dry ice was used for cross‐border shipping (USA to Canada) to prevent thawing.

DNA was extracted from stool fecal punches using the Zymo Quick‐DNA™ Fecal/Soil Microbe Miniprep Kit (D6010). The 16S rRNA gene (V4 region) was amplified in triplicate, combined, purified, and pooled in equimolar concentrations. Sequencing was performed via the Illumina MiSeq platform (reagent kitv.3, 2 × 300 bp base‐pair run),^12^ with paired‐end reads trimmed to 252 bp and clustered into amplicon sequence variants (ASVs) using Deblur (v.1.1.0) and QIIME2 (Quantitative Insights Into Microbial Ecology;v.2019.4).^13^, ^14^ Data were normalized using the median of ratios method (R‐package DESeq2; Differential Expression of Sequencing data) or rarefied to 16,181 sequences for alpha‐ and beta‐diversity analyses.

*Alpha and beta‐diversity* were examined as evenness, richness (Shannon, Margalef's index, Chao1), and weighted UniFrac.^15^ *Gut microbiota network analyses* (genus‐level) used the R‐package SPIEC‐EASI (SParse InversE Covariance Estimation for Ecological Association Inference, neighbourhood mode), when present in ≥80% of samples.^16^ Network connectivity were quantified as *degrees* and *betweenness*.^17^ The five most connected taxa were annotated and described. *Predicted metagenome functions* were generated using the validated Phylogenetic Investigation of Communities by Reconstruction of Unobserved States (PICRUSt2) algorithm, summarized as metabolic pathways (MetaCyc database).^18^, ^19^

### Statistical analyses

Cohort characteristics were described. The gut microbiota metrics were compared by disease, and then DMD status (grouped as three categories: MS, controls, monoADS; then four: MS [DMD‐naïve, exposed], controls, monoADS). Alpha‐diversity, network metrics (connectivity and betweenness), and the metabolic pathway relative abundances were compared between groups using nonparametric tests (Kruskal−Wallis [KW] rank sum test, Holm‐adjusted [adj .] *p*‐values). Beta‐diversity was similarly explored using permutational multivariate analysis of variance (PERMANOVA). The relative abundance of individual ASVs was compared between groups at the phylum, genus, and species‐level, using sex and age at stool sample (continuous) adjusted negative binomial models. Findings were expressed as crude and adjusted‐rate ratios (aRR) and 95% confidence intervals (95%CI), along with *p* and *Q*‐values (false discovery rate adjusted *p*‐values).

To guide future studies, *complementary analyses* were performed for the Canada‐USA cohort, with alpha and beta‐diversity compared by: sex, age at stool sample, race, country of residence, Bristol Stool Scale, BMI, dietary intake (protein, carbohydrate, fiber, and fat), and other medications/dietary supplements), categorized as shown in the Supplementary Methods. Finally, key main analyses (alpha‐, beta‐diversity and genus, and species‐level comparisons) were performed using a similar approach for the pediatric‐onset MS cases (DMD‐naïve and exposed) and unaffected controls within the independently acquired USA‐only cohort. Statistical analyses were performed using R (V.4.0.2).

## Results

In total, 109 participants in the Canada‐USA cohort and 93 in the USA‐only cohort fulfilled inclusion criteria. Characteristics are shown in Tables 1 and 2. In both cohorts, the MS cases/controls were similar in age at stool sample procurement (averaging 16.5/15.1 years for Canada‐USA and 15.9/15.6 years for USA‐only). Females represented 73%–75% of MS cases and 58%–69% of controls across both cohorts. As expected, monoADS participants were younger at symptom onset and at stool sample procurement versus the MS cases and/or controls; 56% were female (Table 1, Canada‐USA cohort). The average dietary metrics were rather similar across groups in both cohorts, as were the Bristol Stool Scale scores in the Canada‐USA cohort.

**Table 1:** Characteristics of the pediatric‐onset multiple sclerosis cases, unaffected controls, and monophasic acquired demyelinating syndrome participants, the Canada‐USA cohort.

Characteristic, *n* (%) unless stated otherwise	Multiple sclerosis cases, *n* = 32	Unaffected controls, *n* = 36	Monophasic demyelinating syndrome participants, *n* = 41
Female	24 (75%)	21 (58%)	23 (56%)
Age at symptom onset, years: mean (SD; range)	14.0 (3.9; 4–17)	–	6.9 (3.9; <1–14.6)
Age at stool sample collection, years: mean (SD; range)	16.5 (3.7; 5–21)	15.1 (3.44; 7–21)	13.8 (4.2; 5–21)
Self‐identified race:^1^ White	17 (61%)	13 (41%)	31 (78%)
Birth country:^2^ North America (Canada or USA)	22 (79%)	31 (91%)	35 (90%)
Country of residence (at stool collection): Canada	21 (66%)	27 (75%)	38 (93%)
USA	11 (34%)	9 (25%)	3 (7%)
Atopy‐related condition (dermatitis, psoriasis, asthma, or allergies): present	8 (25%)	7 (19%)	17 (41%)
Other comorbidity:^3^ present	2 (6%)	1 (3%)	1 (2%)
Disease‐modifying drug (DMD) exposure status:^4^ ever/never	23 (72%)/9 (28%)	–	–
Ever beta‐interferon	11 (34%)	–	–
Ever glatiramer acetate	7 (22%)	–	–
Ever dimethyl fumarate	4 (13%)	–	–
Other^4^	4		
Any other medication (excl. DMDs, incl. vitamins, supplements) 30 days pre‐stool sample:^5^ yes	27 (84%)	16 (44%)	28 (68%)
Mean and total number of different drug classes per child	2.0; total 21	0.8; total 17	1.1; total 13
Any vitamin or dietary supplement:^6^ yes	26 (81%)	10 (28%)	27 (66%)
Mean and total number of different vitamin or dietary supplements^6^	1.2; total 7	0.4; total 5	1.0; total 7
Bristol Stool Scale:^7^ median (IQR)	3 (2.5–4)	4 (3–4)	3 (3–4)
Hard (types 1–2)	8 (26%)	7 (20%)	9 (23%)
Medium (types 3–5)	21 (68%)	27 (77%)	28 (72%)
Loose (types 6–7)	2 (6%)	1 (3%)	2 (5%)
BMI:^8^ crude median (range)	22.8 (13.8–36.3)	19.9 (13.2–29.9)	19.7 (14.0–30.0)
Overweight/obese (≥85th percentile)	5 (16%)	6 (18%)	5 (12%)
Cigarette smoking (passive or active) ever pre‐stool sample	2	1	1
Block Kids Screener:^9^ dietary intake per day, median			
% protein caloric intake (range)	16% (8–23)	16% (10–24)	18% (13–26)
% fat caloric intake (range)	34% (28–51)	34% (23–45)	35% (26–43)
% carbohydrate caloric intake (range)	50% (28–63)	50% (35–68)	50% (33–64)
Grams of fiber (range)	9 (3–20)	11 (4–29)	10 (4–25)
Total with available/valid diet data *n* = 90	26	34	30

Percentage calculated with the denominator reflecting individuals with non‐missing data for that variable; ADS = acquired demyelinating syndrome; BMI = body mass index; DMD = disease modifying drugs; excl. = excluding; MS = multiple sclerosis; SD = standard deviation.

Antibiotic use: by design, no participant had used a systemic antibiotic within 30 days pre‐stool sample. Only one participant (with monophasic acquired demyelinating syndrome) had a record of antibiotic use within 3 months pre‐stool sample (i.e., >30 to 90 days pre‐stool sample). Additional numbers shown below ordered as “MS/controls/ADS”.

Totals exceed 23 (72%) of those ever DMD‐exposed pre‐stool sample as three MS cases were exposed to >1 DMD (the most recent pre‐stool sample is shown first: natalizumab, beta‐interferon [IFNB]; IFNB, dimethyl fumarate [DMF]; teriflunomide, DMF).

^1^ Race: For non‐whites, which included those self‐identifying as a mixed group, the most common were: Black (MS/controls/ADS; 6/3/3); Oriental (0/3/3); Black–Hispanic (0/5/0); Indian or Pakistani (1/3/1); *Unavailable* (4/4/1). European ancestry predominated (15/14/23), for non‐Europeans (including mixed heritage), more commonly identified regions were: Africa (4/5/1) and Asia (1/6/6); *Unavailable:* (6/2/3).

^2^ Birth country: Canada (13/21/29); USA (9/10/6); Other (6/3/4); *Unavailable* (4/2/2).

^3^ Other comorbid conditions (present pre‐stool sample): depression/anxiety (1/0/1); attention deficit hyperactivity disorder (1/0/0); hypothyroidism (0/1/0).

^4^ ‘All “ever DMD” participants had also been exposed within 3 months of stool sample procurement. “Other” DMDs were: rituximab (*n* = 2); natalizumab (*n* = 1); teriflunomide (*n* = 1).

^5^ “Other medications” exclude the MS DMDs and were grouped into drug classes according to the WHO’s Anatomical Therapeutic Chemical (ATC) classification system (level 4, details in Supplement 1).

^6^ The vitamins or dietary supplements were the most commonly used and the total number of classes are shown which included, by ATC group: A11A multivitamins, combinations; A11CC Vitamin D and analogues; A11E Vitamin B‐complex, incl. combinations (B6‐B12); A11GA Ascorbic acid (vitamin C); A11HA Vitamin E; A12AA Calcium; B03A Iron preparations; B03BA Vitamin B12 (cyanocobalamin and derivatives); B03BB Folic acid and derivatives; C10AX Other lipid modifying agents (Omega 3); uncategorized Fish Oil.

^7^ Bristol Stool Scale: 4 participants selected 2 responses (instead of one) and their scores were averaged. Unavailable: (1/1/2).

^8^ BMI unavailable for 2 controls.

^9^ Recent diet: excluded due to implausible daily caloric intake^20^, ^21^[<500 kcal/day (2/1/1); >5000 kcal/day (none)]; missing (4/1/10); total missing or excluded (6/2/11).

**Table 2:** Characteristics of the pediatric multiple sclerosis (MS) cases and unaffected controls from the USA Network of Pediatric MS Center’s microbiome study.

Characteristic, *n* (%) unless stated otherwise	MS cases = 51	Unaffected controls, *n* = 42
Female	37 (73%)	29 (69%)
Age at symptom onset, years: mean (SD; range)	14.5 (2.2; 8.6–17.9)	–
Age at stool sample collection, years: mean (SD; range)	15.9 (2.1; 9.6–19.7)	15.6 (2.8; 8.1–20.7)
Disease duration at stool sample collection, mean (SD; range)	1.3 years (1.1; 0.1–5.4) 16.2 months (13.3; 1.1–*65.3*)	–
Self‐identified race: White	35	34 1 missing
Disease‐modifying drug (DMD) exposure status: ever/never	33 (65%)/18 (35%)	–
Ever beta‐interferon	10 (30%)	–
Ever glatiramer acetate	20 (61%)	–
Ever dimethyl fumarate	3 (9%)	–
Ever natalizumab	6 (18%)	
BMI: crude median (range)	25.0 (17.4–47.0)	22.0 (9.0–43.9) 1 missing
Overweight/obese (≥85th percentile)	9	5
Block Kids Screener: dietary intake per day, median		
% protein caloric intake (range)	16.9 (10.2–25.7)	17.3 (12.0–25.7)
% fat caloric intake (range)	35.5 (21.2–44.9)	36.2 (25.0–47.0)
% carbohydrate caloric intake (range)	47.1 (32.5–67.3)	47.7 (29.8–65.6)
Grams of fiber (range)	10.1 (1.8–25.1)	12.1 (2.9–23.5)
Total with available/valid diet data	*n* = 46	*n* = 40

Percentage calculated with the denominator reflecting individuals with non‐missing data for that variable; BMI = body mass index; DMD = disease modifying drugs; MS = multiple sclerosis; SD = standard deviation. Beta‐interferon products used included: −1a (IM or SC), −1b (SC), and peginterferon beta−1a; 6 MS cases had been exposed to 2 different DMDs (the most common sequential combination was for a beta‐interferon or glatiramer acetate followed by natalizumab; *n* = 3 participants).

All cases had relapsing‐remitting MS, and the mean disease duration (from symptom onset) at stool sample = 30.1 months (SD:35.1) in the Canada‐USA cohort and 16.2 months (SD:13.3) in the USA‐only. Approximately one‐third of MS cases had never used a DMD prior to stool sample procurement [nine (28%) Canada‐USA and 18 (35%) USA‐only]. Beta‐interferon or glatiramer acetate were most commonly used (Tables 1 and 2).

For the Canada‐USA cohort, the mean number of non‐DMD medication/supplement classes used in the previous 30 days = 2.0 for MS, 1.1 for monoADS and 0.8 for control. The most common were vitamins/dietary supplements, with >80% (*n* = 26) of MS cases, 66% (*n* = 27) of monoADS and 28% (*n* = 10) of controls taking ≥one. Atopy was common, affecting 32 (29%) of the participants, but only 4 (4%) had any other comorbidity (Table 1).

### Canada‐USA cohort

#### Gut diversity and taxa‐level findings by disease and DMD status

**Figure 1:**
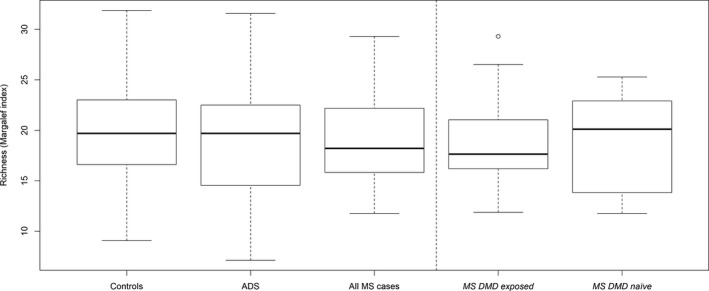
Gut microbiota alpha diversity (richness) for the pediatric‐onset multiple sclerosis cases (DMD‐exposed or naïve), monophasic acquired demyelinating syndromes (monoADS), and unaffected control participants for the Canada‐USA cohort. Margalef's richness index: (*S* − 1)/ln*(n)*, where *S* is the number of taxa, and *n* is the number of individuals. ASV data were rarefied. Box‐and‐whisker plots: thick black horizontal line = median; horizontal edges of box depict Q1 and Q3 (interquartile range); the ends of the whiskers represent one and a half times the interquartile range (1.5*IQR); circles = individual outliers. For example, as depicted, the median richness for each of the participant groups were 18.0 for the multiple sclerosis, 19.2 for the monophasic acquired demyelinating syndrome (ADS) and 18.8 for the unaffected controls. The clinical relevance of these slight differences is unknown. None of the comparisons were statistically significant (all *p* > 0.5). The overall group *p*‐values shown here are based on the Kruskal–Wallis test and the pairwise *p*‐values are based on the Dunn’s Kruskal–Wallis with a Holm adjustment for multiple comparisons: (1) Overall group *p* = 0.507; MS cases versus controls (*p = *0.737), ADS versus controls (*p = *1.00), MS versus ADS (*p* = 0.583). (2) Overall group *p* = 0.521; MS cases DMD‐exposed versus controls (*p = *0.761), MS cases DMD‐naïve versus controls (*p = *0.783), MS DMD‐exposed versus naïve (*p* = 1.00).

Alpha and beta‐diversity did not differ by disease status (across the three groups compared: MS monoADS, controls) or by DMD status (four groups: MS DMD‐exposed/naïve, monoADS, controls), all *p* > 0.1. Figure 1 depicts richness, all other diversity metrics are shown in Table 3.

**Table 3:** Alpha and beta diversity metrics for the gut microbiota of pediatric‐onset multiple sclerosis (MS) cases, unaffected controls and monophasic acquired demyelinating syndromes (ADS) participants in the Canada‐USA cohort.

Alpha diversity, median (quartiles)	MS cases, *n* = 32	Unaffected controls, *n* = 36	ADS, *n* = 41	MS versus controls versus ADS	MS cases only		Comparisons by DMD and disease status	
					DMD exposed, *n* = 23	DMD naïve, *n* = 9	DMD exposed versus naïve MS cases	DMD naïve MS cases versus DMD exposed MS cases versus controls
Richness (number of observed ASVs; Margalef index^1^)	18.2 (15.9, 22.1)	19.7 (16.7, 22.9)	19.7 (14.5, 22.5)	*p* = 0.507^KW^	17.6 (16.2, 21.0)	20.1 (13.8, 22.9)	*p* = 0.675^MW^	*p* = 0.455^KW^
Evenness (Shannon)	0.682 (0.634, 0.706)	0.680 (0.643, 0.693)	0.690 (0.669, 0.715)	*p* = 0.268 ^KW^	0.680 (0.634, 0.719)	0.684 (0.642, 0.689)	*p* = 0.438^MW^	*p* = 0.766 ^KW^
Chao1 (a richness estimate/assesses importance of rare ASVs)	216 (174, 257)	236 (201, 260)	214 (167, 260)	*p* = 0.4942 ^KW^	208 (180, 245)	232 (151, 280)	*p* = 0.722^MW^	*p* = 0.497^KW^
Beta diversity derived from Rsquared,weighted UniFrac; PERMANOVA				1.70%; *p* = 0.498^2^			2.10%; *p* = 0.686^2^	2.00%; *p* = 0.790^2^

ADS = acquired demyelinating syndromes; DMD = disease modifying drugs; MS = multiple sclerosis. Gray shading = not applicable. ^KW^ Kruskal–Wallis; ^MW^ Mann–Whitney test.

Findings not shown for the following comparisons (all *p*‐values were derived from two tests): (1) MS cases versus controls, ADS versus controls, MS versus ADS; (2) MS cases DMD exposed versus controls, MS cases DMD naive versus controls, MS DMD exposed versus naïve (all *p* > 0.05 based on Dunn’s Kruskal–Wallis with a Holm adjustment for multiple comparisons). Diversity metrics shown to 3 significant figures. ASV data were rarefied.

^1^ Margalef's richness index: (*S*−1)/ln(*n*), where *S* is the number of taxa, and *n* is the number of individuals.

^2^ Derived from PERMANOVA (Permutational Multivariate Analysis of Variance, R vegan package; adonis function). Percentage explained, derived from Rsquared.

At the taxon‐level, 8 phyla, 144 genera, and 228 species had sufficient coverage, based on their presence in 80% of samples, for modelling by disease status, with nominal significance (*p* < 0.05) reached for 5 (63%), 44 (31%), and 60 (26%), respectively (Tables S1–S3).

Overall, five phyla, 11 genera and four species reached significance (*p*,*Q* < 0.05) in either the disease or DMD status comparisons. Of the five phyla (*p*,*Q* < 0.05) – *Actinobacteria, Firmicutes, Fusobacteria, Patescibacteria, Verrucomicrobia,* three differed between the MS cases and controls (Table S1). Compared to controls, cases were depleted for *Actinobacteria* (aRR = 0.57;95%CI:0.36–0.91, *p*,*Q* < 0.035) and *Firmicutes* (aRR = 0.66;95%CI:0.46–0.95, *p*,*Q* < 0.038), while enriched for *Verrucomicrobia* (aRR = 13.9;95%CI:2.6–73.8); *p*,*Q* < 0.05), with the latter differing by DMD status, being higher in the DMD‐exposed, but not naïve, MS cases (*p*,*Q* < 0.05). Other group differences also emerged, for example, compared to monoADS, MS cases had a fourfold higher relative abundance of *Patescibacteria* (a recently identified superphylum; aRR = 4.2;95%CI:1.6–11.2, *p* = 0.004,*Q* = 0.01). This higher abundance remained consistent for both the DMD‐exposed and naïve MS cases, although only the former reached significance (*p*,*Q* < 0.035 vs. monoADS).

**Figure 2:**
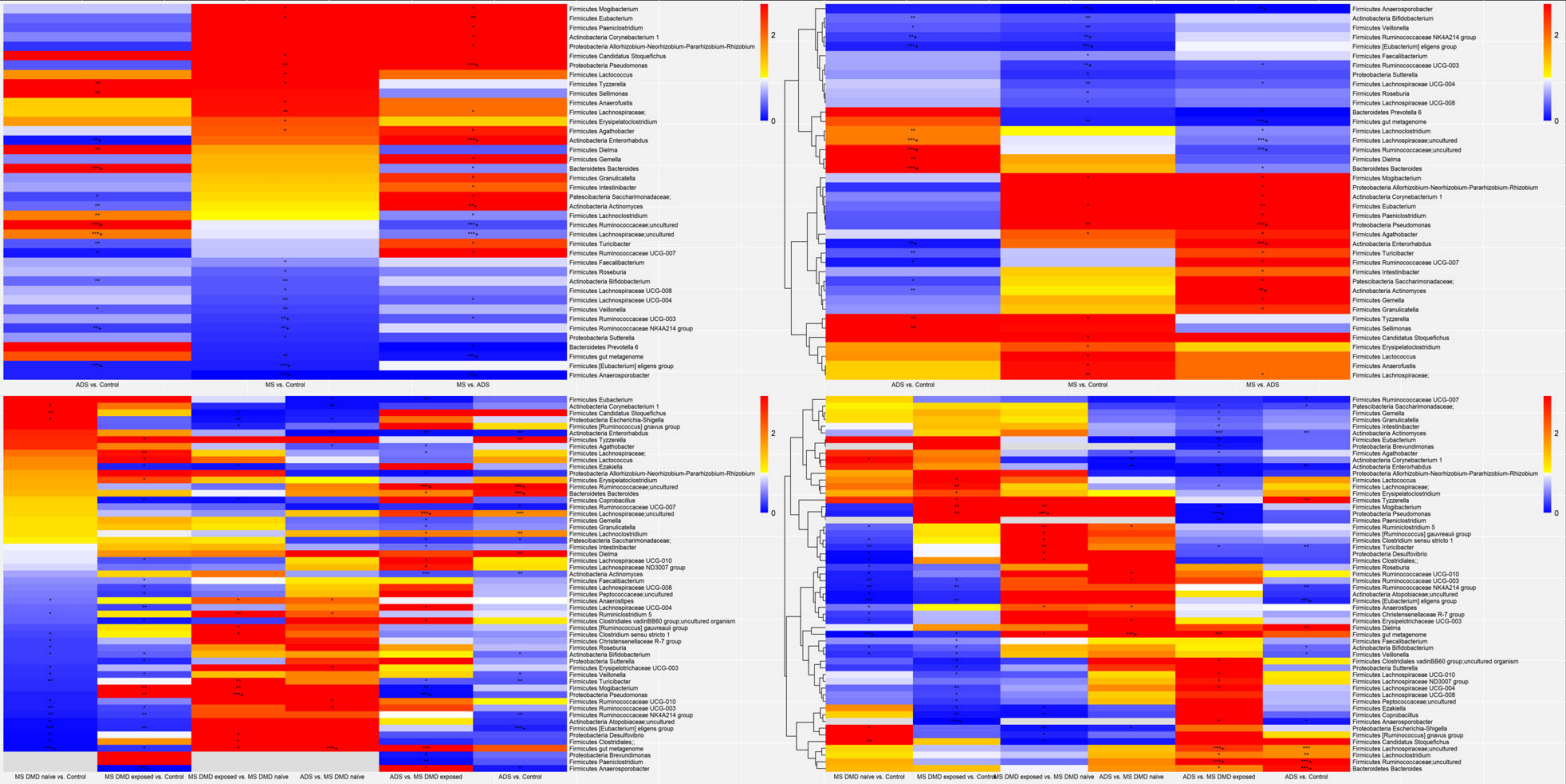
Heatmaps summarizing gut microbiota genus‐level findings (ASV counts) expressed as sex and age‐adjusted rate ratios for the pediatric‐onset multiple sclerosis (MS), monophasic acquired demyelinating syndrome (ADS) and unaffected control participants for the Canada‐USA cohort. (A) Three‐groups compared: multiple sclerosis, ADS, and controls. (B) Three‐groups compared: multiple sclerosis, ADS, and controls, overlaid with a hierarchical cluster analysis. (C) Four‐groups compared: multiple sclerosis (DMD‐naïve and exposed), ADS, and controls. (D) Four‐groups compared: multiple sclerosis (DMD‐naïve and exposed), ADS, and controls, overlaid with a hierarchical cluster analysis. ADS = monophasic acquired demyelinating syndrome, DMD = disease‐modifying drug, MS = pediatric‐onset multiple sclerosis; MS DMD‐naïve = MS case has never been exposed to a DMD at the time of the stool sample. Each Panel summarizes age and sex‐adjusted RRs derived from a single negative binomial regression model for each genus (two models in total, one for three group comparison, and another for four group comparisons), with only the RRs reaching nominal significance (*p* < 0.05) for at least one group comparison within a genus shown (see Tables S1–S3) for unadjusted and adjusted models). For each comparison, the second group forms the reference. *p < 0.05, **p < 0.01, ***p < 0.0001, ***+*p* < 0.0001 and *Q* < 0.05. (A) adjusted RRs were ordered from highest to lowest for each column as follows: (1) MS versus controls (middle column); (2) MS versus ADS (left); (3) ADS versus controls (right). (C) adjusted RRs were ordered from highest to lowest for each column as follows: (1) MS DMD‐naïve versus control; (2) MS DMD‐exposed versus control; (3) MS DMD‐exposed versus naïve; (4) ADS versus MS DMD‐exposed; (5) ADS versus MS DMD‐exposed; (6) ADS versus control. (B and D) Findings are ordered according to the hierarchical cluster analysis (R package pheatmap). Briefly, each taxon is assigned to its own cluster, then the algorithm proceeds iteratively, at each stage joining the two most similar clusters, continuing until there is a single cluster. At each stage distances between clusters are recomputed using the Lance–Williams dissimilarity update formula.^22^ Biological relevance is inferred from the clusters (rather than being directly assessed).

Eleven genera were identified (*p*,*Q* < 0.05): *Actinomyces, Anaerosporobacter, Bacteroides, Enterorhabdus, (Eubacterium) eligens, Pseudomonas*, *Ruminococcaceae NK4A214‐group, Ruminococcaceae UCG−003* and three uncultured/unnamed taxa within Lachnospiraceae*, Ruminococcaceae* and *Clostridiales vadin BB60* (Fig. 2A,C, and Table S2). Four genera *Anaerosporobacter, Ruminococcaceae UCG−003, Clostridiales vadin BB60, Pseudomonas* differentiated MS cases from the others, with the latter two influenced by the cases’ DMD exposure status. Both *Anaerosporobacter* and *Ruminococcaceae UCG−003* were lower in MS versus either controls or monoADS (for *Anaerosporobacter* both aRR<0.01, *p*,*Q* < 0.05; for *Ruminococcaceae UCG−003*, the respective aRR = 0.23;95%CI:0.09–0.59, *p*,*Q* < 0.05 and 0.35;95%CI:0.14–0.90, *p* < 0.05, but *Q* > 0.05), with the direction of findings for both genera consistent regardless of the MS cases’ DMD exposure. For *Clostridiales vadin BB60,* both DMD‐naïve and exposed MS cases were lower in relative abundance versus monoADS (*p*,*Q* < 0.01 and *p* = 0.001, but *Q* = 0.06, respectively), while the DMD‐naïve MS cases were lower versus both the controls (aRR = 0.02;95%CIs:0.00–0.17, *p*,*Q* < 0.04) and the DMD‐exposed cases (*p* < 0.05, although *Q* > 0.05). *Pseudomonas* was also lower for the DMD‐naïve versus exposed MS cases (*p*,*Q* < 0.03), while MS cases were enriched versus monoADS (aRR = 16.5;95%CI:4.3–62.5, *p*,*Q* < 0.007) which remained significant for the DMD‐exposed MS cases only (vs. monoADS, *p*,*Q* < 0.004).

Other genera appeared particularly relevant in differentiating monoADS participants from the other groups––both *Actinomyces* and *Bacteroides* differed versus the MS cases or controls (*p* < 0.05, although not all *Q* < 0.05), while no differences emerged when the MS cases and controls were directly compared (*p* > 0.05). Shared features for both disease groups were observed: versus controls, both *Ruminococcaceae‐NK4A214 group* and *(Eubacterium) eligens* were lower in MS and monoADS (*p*,*Q* < 0.05), while the two disease groups did not differ (*p* > 0.05). Finally, for the remaining three genera, findings were largely driven by differences in relative abundance between MS and monoADS participants. For example, both *Lachnospiraceae* and *Ruminococcaceae* were lower in MS (and the DMD‐exposed subgroup) versus monoADS, while the latter were enriched versus controls (all *p*,*Q* < 0.05). Conversely, *Enterorhabdus* was higher in MS versus monoADS (aRR = 26.2;95%CI:4.6–149.3, *p*,*Q* < 0.02), with the direction of findings consistent irrespective of the MS cases’ DMD‐exposure (*p* < 0.01). Further, the monoADS participants were depleted versus controls (*p*,*Q* < 0.05).

The four identified species (*p*,*Q* < 0.05) were housed within the genera: *Anaerosporobacter, Enterorhabdus, Pseudomonas*, or family *Ruminococcaceae* (Table S3). Findings largely mirrored the genus‐level observations. Briefly, *Anaerosporobacter* sp. (family *Lachnospiraceae*) was lower for MS cases versus either controls (*p* < 0.0001, *Q* = 0.003) or monoADS (*p* = 0.00153, *p*,*Q* > 0.05), with both aRR<0.02. Compared to monoADS, *Enterorhabdus* sp. was higher for controls (*p*,*Q* < 0.05) and MS cases (*p* < 0.05, *Q* > 0.05). *Pseudomonas* sp., and *Ruminococcaceae* sp. were both lower in monoADS, particularly versus the DMD‐exposed MS cases (*p*,*Q* < 0.05).

#### Gut microbiota network analysis by disease and DMD status

**Figure 3:**
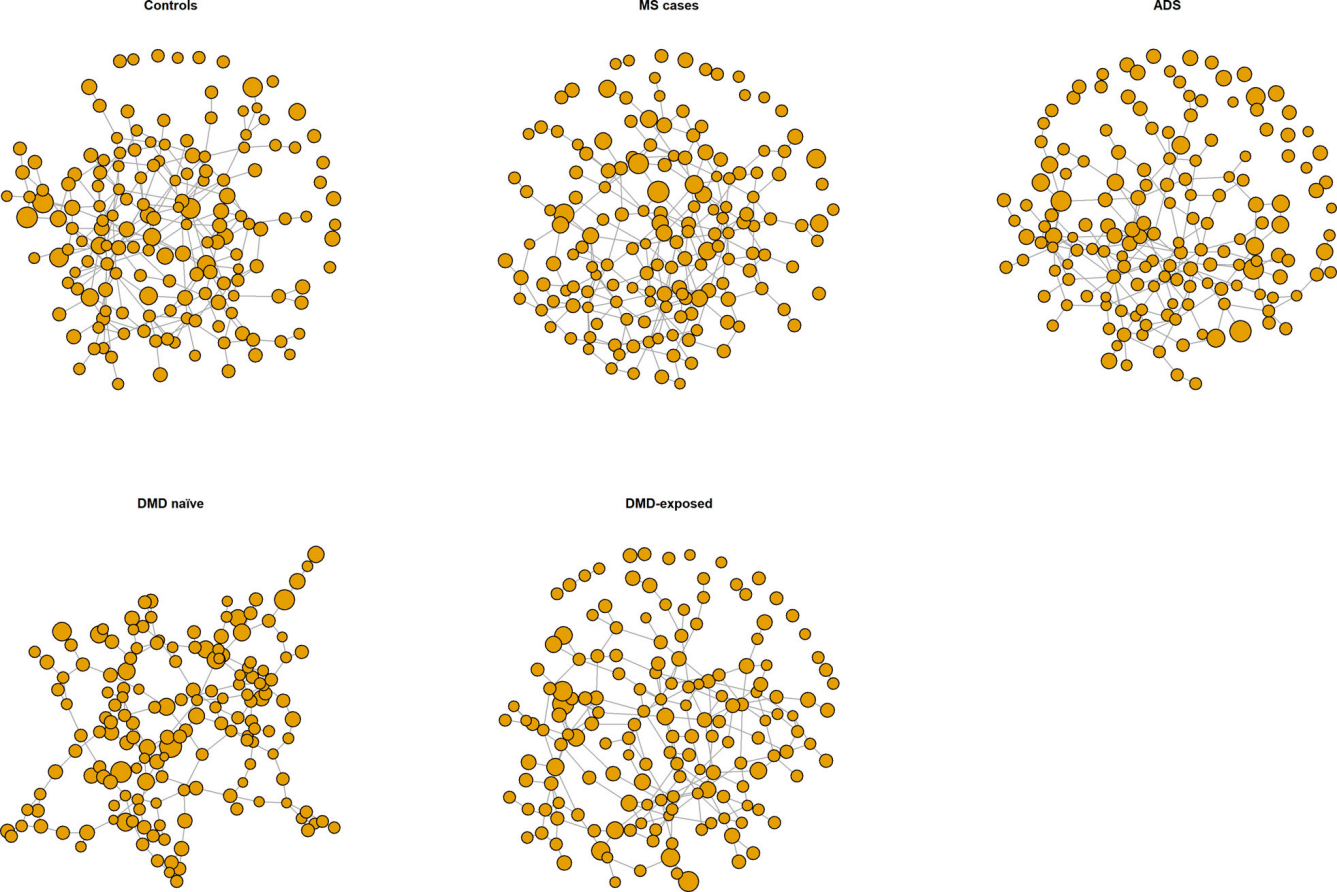
Gut microbiota network analysis (genus‐level): depiction of the gut microbiota networks. ADS = monophasic acquired demyelinating syndrome; MS = multiple sclerosis (pediatric‐onset). Each sphere represents a node (taxa); sphere size represents the normalized counts (number of taxa). Lines show connectivity; longer lines indicate less connectivity. Connectivity can be positive or negative (genera may promote or inhibit each other). Annotated network analyses plots, with the relevant genera labelled are shown in Figure S1. Figure 4 quantifies and compares connectivity (betweenness) between the control, monoADS and MS participants.

**Figure 4:**
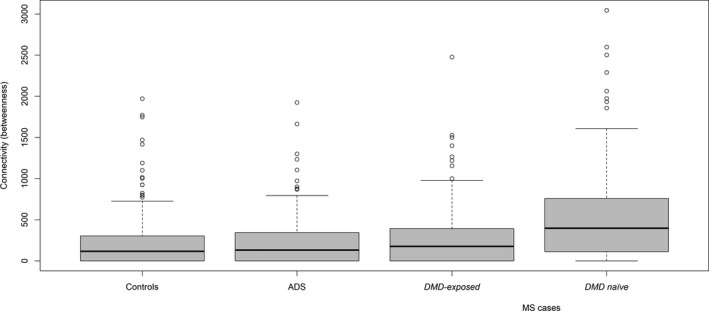
Gut microbiota network analysis (genus‐level): box plots show node (taxa) connectivity (betweenness) for the unaffected controls, monoADS, and pediatric‐onset MS cases (DMD‐exposed and naïve). ADS = monophasic acquired demyelinating syndrome; MS = multiple sclerosis (pediatric‐onset). Summary: node (taxa) connectivity (betweenness) differed between the 4 groups: controls, monoADS, DMD‐exposed and naïve MS cases (*p* < 10‐9 Kruskal‐Wallis) The DMD naïve MS cases also differed from each of the groups ‐ controls, monoADS, & DMD‐exposed MS cases (all *p* < 0.00007, Holm adjusted for multiple pairwise comparisons). Additional information and related analyses: The degree of connectivity did not differ between groups (all *p* > 0.05, data not shown).

From network analyses, the MS cases, monoADS, and controls’ genus‐level gut microbiota did not differ by degree of connectivity or betweenness (*p* > 0.1, Figs. 3 and 4). However, findings differed by DMD status; the naïve MS cases exhibited a visually distinct gut microbial network (Fig. 3) and had a higher connectivity (betweenness) versus the other three groups (DMD‐exposed cases, monoADS, and controls, all *p* < 0.00007, Fig. 4). Further, annotation of the five most connected taxa suggested distinct patterns across groups (Fig. S1A–E). For example, four of the five most connected taxa (by degrees or betweenness) for controls were short chain fatty‐acid (SCFA) producers (all were in the *Firmicutes* phylum, e.g., *Ruminococcaceae* family members [*UCG‐003, UCG‐005*], *Anaerostipes,* and *Veillonella*). Conversely, for the MS cases, several housed microbes commonly cited as opportunistic pathogens (e.g., *Actinobacteria* [phylum *Actinomyces*], *Gemella,* and *Leuconostoc* [phylum *Firmicutes*]). Among the most connected taxa (by degrees or betweenness) for the DMD‐naïve MS cases, several had been identified relatively recently; all were contained in the phylum *Firmicutes: Candidatus Stoquefichus, Mogibacterium, Phocea,* and *Subdoligranulum*.

#### Metagenomic predictions by disease and DMD status

**Figure 5:**
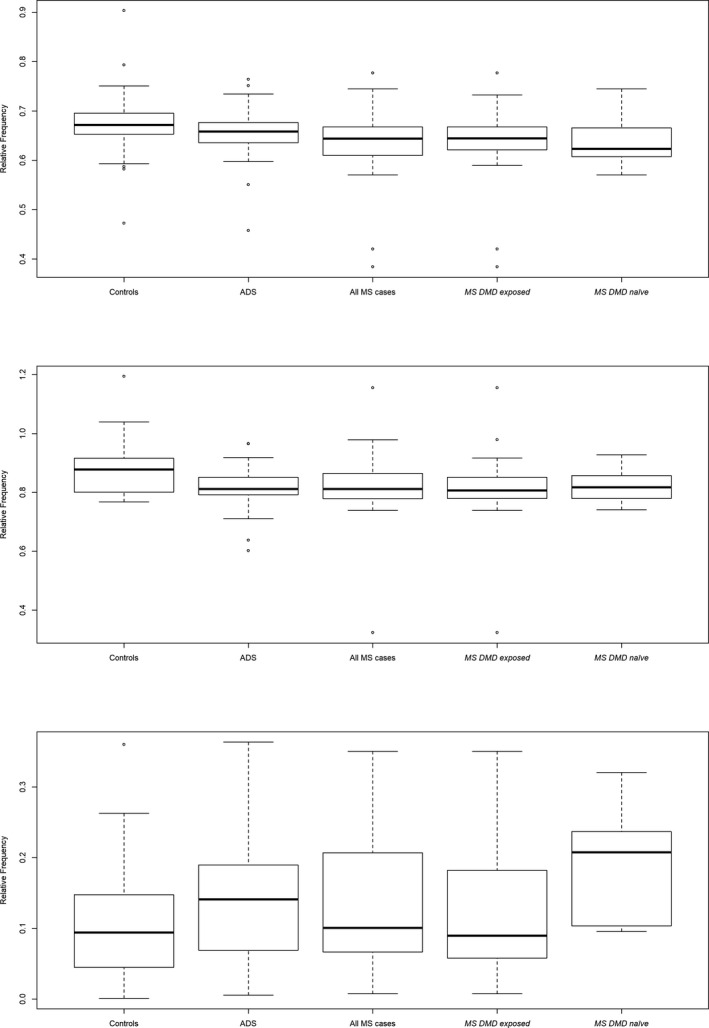
Metagenomic predictions: relative abundance of three predicted metagenomic pathways for unaffected controls (*n* = 36), monoADS participants (*n* = 41), and MS cases (all MS cases, *n* = 32, then by DMD exposure status [exposed, *n* = 23 or naïve, *n* = 9]). (A) Nicotinamide adenine dinucleotide (NAD) salvage pathway I. Summary of group comparisons: 3 groups: controls, ADS, MS cases, P=0.0311KW Pairwise comparisons where P<0.05. Controls vs: MS cases; **P=0.00939; adj.P=0.0282** 4 groups: controls, ADS, MS DMD exposed, MS DMD naïve cases P=0.0675^KW^ Pairwise comparisons where P<0.05.Controls vs: DMD+ MS cases; **P=0.0294**; adj.P=0.177; DMD‐ MS cases; **P=0.0419**; adj.P=0.209; (B) Pyruvate fermentation to acetate and lactate II pathway. Summary of group comparisons: 3 groups: controls, ADS, MS cases, **P=0.0182** **^KW^** Pairwise comparisons where p<0.05. Controls vs: MS cases; **P=0.0148; adj.P=0.0295**; monoADS; **P=0.0137; adj.P=0.0410** 4 groups: controls, ADS, MS DMD exposed, MS DMD naïve cases, **P=0.0456** **^KW^** Pairwise comparisons where p<0.05. Controls vs: DMD+ MS cases; **P=0.0245**; adj.P=0.123; monoADS; **P=0.0137**; adj.P=0.0820; (C) The reductive tricarboxylic acid (TCA) cycle I pathway. Summary of group comparisons: 3 groups: controls, ADS, MS cases, P=0.168^KW^ Pairwise comparisons; all P>0.05. 4 groups: controls, ADS, MS DMD exposed, MS DMD naïve cases, **P=0.0250** ^**KW**^ Pairwise comparisons where P<0.05. Controls vs: DMD‐ MS cases; **P=0.00559; adj.P=0.0336** DMD‐ vs DMD+ MS cases; **P=0.0161**; adj.P=0.0806. Plots provide key examples of where the relative abundance(s) of pathways were lower for the MS cases relative to unaffected controls, with the direction of findings remaining consistent regardless of DMD exposure (A and B) or differed by DMD exposure status, being lower for the DMD‐exposed relative to naïve MS cases (C). All predicted metagenomic findings are shown in Table S4. Box plots: Thick black horizontal line = median; horizontal edges of box depict Q1 and Q3 (interquartile range); the ends of the whiskers represent one and a half times the interquartile range (1.5*IQR); circles = individual outliers. Y axis represents the mean relative pathway abundance from metagenomic predictions, derived from a validated algorithm via PICRUSt2 (Phylogenetic Investigation of Communities by Reconstruction of Unobserved States) and summarized as metabolic pathways using MetaCyc (database of metabolic pathways and enzymes from all domains of life). ADS = monophasic acquired demyelinating syndromes; MS = multiple sclerosis (pediatric onset); DMD = disease modifying drug; DMD+/DMD− = DMD disease modifying drug exposed/naïve (never exposed); KW = Kruskal–Wallis rank sum test; adj.*p* = multiple comparisons adjusted *p*‐values (derived from the Dunn Kruskal–Wallis test with Holm adjustment for multiple comparisons). Bolded *p*‐values indicate <0.05 (reached nominal significance).

After filtering for sparsity, 193 of 399 identified pathways were assessed, and 50 differed between the MS, monoADS, and controls (*p* < 0.05, KW, Fig. 5, Table S4). From the pairwise comparisons, while the MS and monoADS participants did not differ from each other (all *p* > 0.05), both differed versus controls: five pathways were enriched and eight depleted in MS, and 17 were enriched and 31 depleted in monoADS (all Holm‐adjusted KW for multiple comparisons *p* < 0.05). For example, the *nicotinamide adenine dinucleotide salvage* and *pyruvate fermentation* pathways differed across the three main groups (KW, *p* = 0.0311 and *p* = 0.0182, respectively), being depleted in MS versus controls (both adj.*p* < 0.03; Fig. 5A and B). The latter pathway was also lower in monoADS versus controls (adj.*p* = 0.0410). While the direction of these findings was consistent regardless of whether MS cases were DMD‐naïve or exposed (unadj.*p* < 0.05), others differed, although none remained significant after multiple comparison adjustments. For example, both the *reductive tricarboxylic acid cycle I* and *glycolysis* pathways were enriched for the DMD‐naïve MS cases (unadj.*p* < 0.05, Fig. 5C).

#### Complementary analyses

For all Canada‐USA participants combined, those exposed (versus unexposed) to other medications/supplements exhibited lower alpha‐diversity (richness, Chao1 *p* < 0.02). Beta‐diversity also differed by other medication use, race, country of residence, and dietary intake (fiber), but all associations were small, explaining 3–8% of the variability in the gut microbiota (all *p* < 0.02). No other findings reached significance (all *p* > 0.05; Table S5).

#### Comparisons with the independent USA‐only cohort

##### Diversity

Consistent with the Canada‐USA cohort, alpha and beta gut microbiota diversity did not differ for the MS cases (DMD‐naïve or exposed) versus the unaffected controls, all *p* > 0.1 (data not shown).

##### Taxa‐level findings

**Figure 6:**
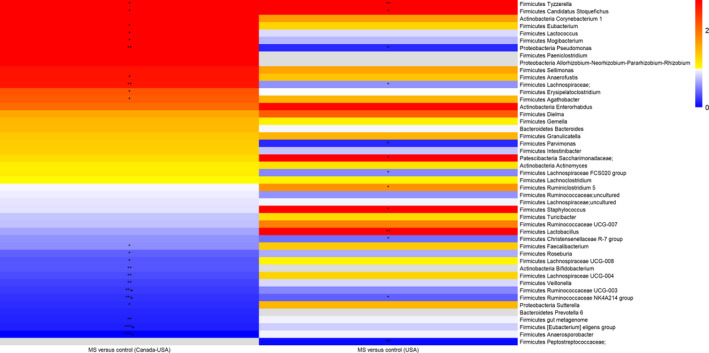
Heatmap summarizing gut microbiota genus‐level findings (ASV counts): Comparisons between the Canada‐USA and USA‐only cohorts for the multiple sclerosis cases and controls. Adjusted RRs were ordered from highest to lowest for the multiple sclerosis cases versus controls, first, for the Canada‐USA cohort, then for the USA‐only. Each Panel summarizes age and sex‐adjusted RRs derived from negative binomial regression models for each genus, with only the RRs reaching nominal significance (*p* < 0.05) in at least one of the cohorts within a genus shown (see Tables S2 and S6) for unadjusted and adjusted models). For each comparison, the control group forms the reference. RRs are colored from high (red) to low (blue); gray shading–not available. *p < 0.05, **p < 0.01, ***p < 0.0001, ***+*p* < 0.0001 and *Q* < 0.05.

While consistency in the direction of effect was observed across several genera and species for the MS cases and controls within each cohort, none reached both *p* and *Q* < 0.05 in both cohorts (Fig. 6, Tables S6 and S7). Briefly, at the genus‐level, when compared to controls, the relative abundance of *Ruminococcaceae‐NK4A214 group* was lower for the MS cases, reaching significance in both cohorts (aRR_Canada‐USA_0.20;95%CI:0.08–0.55, *p* = 0.002; aRR_USA_0.38;95%CI:0.18–0.82, *p* = 0.01), including for the DMD‐naïve cases (aRR_Canada‐USA_0.10;95%CI:0.02–0.47, *p* = 0.003; aRR_USA_0.27;95%CI:0.10–0.76, *p* = 0.01). *Christensenellaceae R − 7 group* was lower for the MS cases, reaching significance at either the genus‐ or species‐level in each cohort (genus‐level: aRR_Canada‐USA_0.58;95%CI:0.28–1.2, *p* = 0.1; aRR_USA_0.44;95%CI:0.23–0.83, *p* = 0.01; species‐level: (aRR_Canada‐USA_0.13;95%CI:0.02–0.81, *p* = 0.03; aRR_USA_0.25;95%CI:0.05–1.29, *p* = 0.096). Other genera were also lower in MS in both cohorts (aRR < 1, but *p* > 0.05), and all were housed in the phylum *Firmicutes*, including *Veillonella* and three within the order *Clostridiales* (*Roseburia, Ruminococcaceae UCG−003*). In addition, some genera were higher in MS versus controls in both cohorts, such as: *Candidatus Stoquefichus* (also identified as highly connected in the Canada‐USA network analyses) and *Tyzzerella* aRR_Canada‐USA_10.1–12.8; aRR_USA_8.8–9.1, all *p* < 0.05). The genera *Clostridium sensu stricto 1* and *Turicibacter* specifically differed by the MS cases’ DMD status in both cohorts, being higher for the DMD‐exposed versus naïve (aRR_Canada‐USA_4.1–5.5 and aRR_USA_2.6–2.9, all *p* < 0.05). Further, compared to controls, the DMD‐naïve cases exhibited a significantly higher relative abundance of the species *Clostridium innocuum group* (genus *Erysipelotrichaceae;* aRR exceeded 10, with *p* < 0.03 in both cohorts) and lower abundance of an unnamed species within the genus *Ruminococcaceae UCG−003* (aRR<0.4, *p* < 0.047 in both cohorts).

Conversely, two of the genus‐level findings differed across cohorts; both were higher for the MS cases (vs. controls) in the Canada‐USA but lower in the USA‐only cohort (all *p* < 0.05). These comprised *Pseudomonas* (*Proteobacteria* phylum) (aRR_Canada‐USA_5.5;95%CI:1.5–20.2, *p* = 0.01; aRR_USA_0.16;95%CI:0.03–0.85, *p* = 0.03) and an unnamed taxon within the functionally diverse *Lachnospiraceae* family (aRR_Canada‐USA_2.6;95%CI:1.4–5.1, *p* = 0.004; aRR_USA_0.57;95%CI:0.37–0.88, *p* = 0.001). The latter similarly differed for the DMD‐exposed cases, versus controls (aRR_Canada‐USA_2.8; 95%CI;1.4–5.8, *p* = 0.005; aRR_USA_0.48; 95%CI:0.30–0.78, *p* = 0.003).

On completion of the pre‐planned analyses, post‐hoc principal component analysis (PCA) plots (genus‐level) were generated to explore the potential for bias for the following elements: for the Canada‐USA cohort, presence of atopy, and use of supplements (all participants combined, then separated by disease status), for both cohorts combined, shipping‐related (use of additional dry ice shipment [yes, no]) and cohort (Canada‐USA, USA‐only). No remarkable patterns or clustering of individuals based on these elements were observed (all PCA plots available upon request from the authors).

## Discussion

While overall gut microbiota diversity did not differ between individuals with pediatric‐onset MS (DMD‐naïve or exposed), monoADS, and unaffected controls, important taxa‐level differences emerged. Compared to monoADS, MS cases exhibited a fourfold higher relative abundance of the recently identified superphylum *Patescibacteria* (aRR = 4.2; *p*,*Q* < 0.05), and a lower abundance of several butyrate‐producing genera and species within *Lachnospiraceae* (e.g., *Anaerosporobacter*) and *Ruminococcaceae* families (*p*,Q < 0.05). An unnamed species within the genus *Clostridiales vadin BB60* differentiated the DMD‐naïve MS cases from all others, being depleted versus either the DMD‐exposed cases, controls (*p*,Q < 0.01) or monoADS participants (*p* = 0.001, *Q* > 0.05). In addition, for all MS cases (either DMD‐exposed or naïve), gut metagenomic predictions suggested depletion in the tryptophan‐related *nicotinamide adenine dinucleotide salvage* and SCFA‐producing *pyruvate fermentation* pathways versus controls (Holm‐adjusted *p* < 0.03). These pathways were recently found of importance in persons with MS and MS animal models.^23^ Finally, network analyses revealed a distinct gut microbiota community structure for the MS cases versus monoADS and controls, with an overrepresentation of highly connected opportunistic pathogens, and an under representation of SCFA‐producing taxa. Together, findings suggest that the gut microbiota community structure, function and connectivity, and not just individual taxa, are of likely importance in MS.

We were able to compare our main findings with an independent cohort of pediatric‐onset MS cases and unaffected controls. Consistent across both cohorts, and with most other studies,^4^, ^6^, ^24^, ^25^, ^26^, ^27^ neither alpha nor beta gut microbiota diversity differed significantly for the MS cases (DMD‐naïve or exposed) versus controls. At the individual ASV‐level, these included a lower relative abundance of several SCFA‐producing taxa for the MS cases versus controls, such as *Ruminococcaceae‐NK4A214 group* which was 61%–79% lower for the cases (aRR:0.39–0.21) and 72%–90% lower for the DMD‐naïve cases (aRR:0.28–0.10) across both cohorts, all *p* < 0.02. Depletion of this genus has been reported in MS and other chronic diseases,^24^, ^28^, ^29^ as has depletion of other SCFA‐producing taxa in MS (versus controls), including for example, *Butyricimonas* ^27^ and *Clostridia* clusters XIVa and IV.^25^ *Christensenellaceae R‐7 group*, a member of the highly inheritable *Christensenellaceae* family,^30^ was lower for the MS cases versus controls across both cohorts, by 42%–52% at the genus‐level (aRRs:0.58–0.44) and 75–87% at the species‐level (aRRs:0.25–0.13), reaching nominal significance (*p* < 0.05) in the USA‐only cohort at the genus‐level, and in the Canada‐USA cohort at the species‐level. *Christensenellaceae* appears depleted in other immune‐mediated conditions, such as IBD, as well as MS, suggesting that diminution of these genera may be important for systemic T‐cell dysregulation disorders.^6^, ^30^ A large meta‐analysis, comprised 3,048 individuals, identified *Christensenellaceae R‐7 group*, along with *Ruminococcaceae UCG‐005*, as among the top five genera enriched in controls versus IBD‐participants.^31^ Considered as potential biomarkers of a healthy gut,^31^ these observations broadly concurred with ours, including, for example, our Canada‐USA cohort’s gut community network analyses; *Ruminococcaceae UCG‐005* genus was among the top five most connected taxa for the unaffected controls. Authors of the same meta‐analyses identified members of the family *Erysipelotrichaceae* as potential markers of gut inflammation.^31^ Relatedly, we observed >10‐fold higher relative abundance of *Clostridium innocuum group* sp., housed within this family for our MS cases in both cohorts (*p* < 0.03). Interestingly, both of the genus‐level taxa that differed between our cohorts (families *Pseudomonas* and *Lachnospiraceae*) house functionally diverse species, such that inconsistencies in findings across studies and diseases are not unexpected.^32^

Intriguingly, within our main Canada‐USA cohort, the DMD‐naïve MS cases’ gut communities were more connected (betweenness) versus all others (DMD‐exposed cases, monoADS, or controls), perhaps indicative of more resilient pathogenic or pro‐inflammatory microbial communities in the native MS gut. Findings warrant further investigation in larger populations, although accessing sizable groups of individuals with MS who are entirely DMD‐naïve is challenging in today’s therapeutic era. We found just one other study assessing gut community structures in adults with MS (aged 20–63 years).^33^ They also observed differences in network connectivity between DMD‐naïve MS cases (*n* = 45) and controls (*n* = 44).^33^ While the degree of connectivity was lower for the MS cases in this older population, inferences are similar to ours; gut microbiota communities and network structure are of likely importance, not just individual taxa. We annotated our gut microbiota community networks, observing overrepresentation in MS cases of opportunistic pathogens, for example, *Actinomyces* (*Actinobacteria* phylum), *Gemella*, and *Leuconostoc (Firmicutes* phylum). In contrast, for controls, SCFA‐producing taxa dominated, including *Ruminococcaceae* family members, *Anaerostipes* and *Veillonella* (*Firmicutes* phylum). Other members of the *Veillonellaceae* family are also reported as higher in controls (versus DMD‐naïve MS case),^5^ and higher in DMD‐naïve versus exposed MS cases.^34^ SCFAs represent key microbial metabolites, can exert anti‐inflammatory effects, and may facilitate beneficial microbiota‐gut‐brain interactions.^35^

Gut metagenomic predictions suggested depletion of the *nicotinamide adenine dinucleotide salvage* and *pyruvate fermentation* pathways in MS (DMD‐exposed or naïve) versus controls. Both pathways are implicated in MS pathogenesis, possibly mediated via SCFA‐production (acetate and lactate), neuronal mitochondrial damage and energy depletion.^36^, ^37^, ^38^, ^39^ Modification of these pathways may has potential neuroprotective and immunomodulatory effects in MS.^36^, ^37^, ^38^, ^39^ Our findings concur with the broader MS literature; for example, dietary precursors of the former *nicotinamide salvage* pathway include tryptophan and niacin (vitamin B_3_), and a higher abundance of gut‐derived tryptophan metabolites were associated with a lower risk of pediatric‐onset MS and subsequent disease activity.^23^ Two other pathways—*reductive tricarboxylic acid cycle I* and *glycolysis*—differed by DMD status, being lower for our DMD‐exposed versus naïve MS cases. Both are considered central hubs for energy metabolism,^40^, ^41^ and others report upregulation within active MS lesions, and a relationship of these pathways with disease severity.^42^, ^43^ Further, pathways related to energy metabolism (e.g., methane) and metabolism of other vitamins (e.g., retinol, vitamin A) have been shown to differ by DMD status.^34^ These predicted pathways provide mechanistic insights warranting future direct functional characterization of the microbiota.

### Strengths and limitations

Pediatric‐onset MS remains relatively rare, such that our study size was modest. Nonetheless, our participants were well phenotyped; our MS cases, controls, and monoADS participants were similar for important metrics rarely captured, such as stool consistency (via the Bristol Stool Scale). This is considered an important confounder in gut microbiota studies.^9^ Household controls (typically spouses, sometimes siblings) can be another approach, but are not well suited to a rare pediatric disease for several reasons.^2^, ^44^, ^45^ Adults are not suitable controls for children, and declining fertility rates renders it impractical to enroll a household sibling of similar age and sex (North American women average <2 children).^46^ Overmatching and misclassification also pose a threat^44^, ^47^; an unaffected sibling is genetically predisposed to be at a higher risk of MS, but may not develop MS until some (unknown) time in the future.

Our MS cases had a relatively short disease duration, averaging 2.5 years from symptom onset in our primary Canada‐USA cohort, and had thus accrued few comorbidities (aside from atopy), and had a low medication burden, aside from dietary supplement use. Nonetheless, our complementary analyses suggested some modest differences in gut diversity based on broad cohort characteristics, including race, country of residence, fiber intake and other medication, and supplement use. These modest differences, and others, may become relevant in larger cohorts and warrant further consideration, especially as comorbidity and chronic medication/supplement use are common, increase with age and disease duration,^48^ and most MS microbiota studies have included older adults with very long disease durations.^6^ This raises the possibility that these seldom reported exposures may have a profound impact on the adult MS gut microbiota, including, for example, the commonly used antipsychotics.^16^, ^17^ Further, supplement use (e.g., vitamin D) is common in persons with MS of all ages; our findings suggest that future work is needed to establish its full potential impact on the MS gut microbiota. Our inclusion of pediatric‐onset MS cases who were *never* exposed to a DMD and use of an additional comparator group (monoADS) may be of value to advance understanding of which differences in the gut microbiota are specific to MS, or are common to other neurological or demyelinating diseases. We also compared our main findings with an independent cohort of pediatric‐onset MS cases and unaffected controls, with all samples collected in a similar manner and sequenced in the same central facility.^49^ Finally, we were able to assign taxonomy using the newer ASVs^50^ rather than the operational taxonomic unit system employed previously in MS.^6^ ASVs are considered advantageous in achieving greater resolution and may have enhanced our ability to detect previously unrecognized taxa of importance in MS.

## Conclusions

Gut microbiota diversity was similar for pediatric‐onset MS cases versus either monoADS or unaffected controls. However, at the taxa‐ and gut‐community‐network‐level, differences were observed. MS cases, irrespective of prior DMD exposure, exhibited an overrepresentation of highly connected opportunistic pathogens, and an under representation of SCFA‐producing taxa. Further, several SCFA‐producing taxa, such as *Ruminococcaceae NK4A214*, and *Christensenellaceae R‐7 group*, identified as possible universal makers of gut health,^31^ were consistently lower for the pediatric‐onset MS cases versus unaffected controls across two independent North American cohorts. Together, findings suggest that commonality in the gut microbiota composition can be found across different MS cohorts, and that disruptions in key taxa may contribute to MS pathogenesis. Further, findings suggest that the gut microbiota community structure, function, and connectivity, and not just individual taxa, are of likely importance in MS. Further work is warranted to delineate the likely bi‐directional relationship between the gut microbiota and MS.

## Author Contributions

HT, YZ, CBe, GVD, MG, JH, and EW contributed to the original funded gut microbiota grant proposal. DA, AB‐O, RAM, JOM, EAY, and BB were part of the original Canadian Pediatric Demyelinating Disease Network study and facilitated collection of the Canada‐USA cohort characteristics.

JH facilitated training of study coordinators the collection of stool samples. CBe oversaw the biobanking. MG, NK, and GVD oversaw the 16S rRNA sequencing and bioinformatics; CBo, performed the stool extractions and 16S rRNA sequencing; JF, AM, NK, and FZ performed the bioinformatics, and FZ the statistical analyses and creation of Figures. JH and EW facilitated data access to the US Network of Pediatric MS Centers study cohort. All authors contributed to the interpretation of the data. HT drafted the manuscript. All authors revised the manuscript and approved the final version to be published.

## Conflict of Interest

JF, NK, MG, CBo, JH, and JO have no conflict of interest to report.

DA–is funded by the Canadian MS Society, the International Progressive MS Alliance, the Canadian Institutes of Health Research and the US Department of Defense. He has received personal compensation for serving as a Consultant for Alexion, Biogen, Celgene, Frequency Therapeutics, GENeuro, Genentech, Merck/EMD Serono, Novartis, Roche, and Sanofi. Dr. Arnold has an ownership interest in NeuroRx.

GVD is the Chief Bioinformatics Scientist with the National Microbiology Laboratory––Public Health Agency of Canada and has received research support in the last 3 years from the National MS Society, the Canadian Institute of Health Research, and Genome Canada.

CBe has served on advisory boards for Abbvie Canada, Amgen Canada, Bristol Myers Squibb Canada, Roche Canada, Janssen Canada, Takeda Canada, Pfizer Canada Sandoz Canada, consulted to Mylan Pharmaceuticals and Takeda, has received educational grants from Abbvie Canada, Pfizer Canada, Takeda Canada, Janssen Canada and has been on the speaker’s panel for Medtronic Canada, Janssen Canada, Takeda Canada, Pfizer Canada, and Abbvie Canada.

EAY has received research support in the last 3 years from the National MS Society, Canadian Institutes of Health Research, National Institutes of Health, Ontario Institute of Regenerative Medicine, Stem Cell Network, SickKids Foundation, Peterson Foundation, MS Society of Canada, and the MS Scientific Research Foundation. She has received funding for investigator‐initiated research from Biogen and has served on scientific advisory boards for Biogen, Alexion, and Hoffman‐LaRoche.

AB‐O is funded by the NIH, ITN, NMSS, and MSSOC. AB‐O has participated as a speaker in meetings sponsored by and received consulting fees and/or grant support from: Janssen/Actelion; Atara Biotherapeutics, Biogen Idec, Celgene/Receptos, Roche/Genentech, Medimmune, Merck/EMD Serono, Novartis, Sanofi‐Genzyme.

BB serves as a consultant to Novartis, UCB, and Roche. BB provides non‐remunerated advice on clinical trial design to Novartis, Biogen, Teva Neuroscience. BB is funded by the NMSS, NIH, and Canadian MS Society.

EW is funded by the NMSS, the NIH, PCORI, and the Race to Erase MS. EW has received consulting honoraria from Jazz Pharma, Emerald, and DBV. She volunteers on a clinical trial committee for Novartis.

HT is the Canada Research Chair for Neuroepidemiology and Multiple Sclerosis. Current research support received from the National Multiple Sclerosis Society, the Canadian Institutes of Health Research, the Multiple Sclerosis Society of Canada and the Multiple Sclerosis Scientific Research Foundation. In addition, in the last 5 years, has received research support from the UK MS Trust; travel expenses to present at CME conferences from the Consortium of MS Centres (2018), the National MS Society (2016, 2018), ECTRIMS/ACTRIMS (2015, 2016, 2017, 2018, 2019, 2020), American Academy of Neurology (2015, 2016, 2019). Speaker honoraria are either declined or donated to an MS charity or to an unrestricted grant for use by HT’s research group.

YZ and FZ were funded through research grants held by HT, including The Multiple Sclerosis Scientific and Research Foundation (PI: Tremlett, EGID: 2636).

AM is funded through the MS Society of Canada endMS Doctoral Studentship (EGID: 3246) and was funded through a The Multiple Sclerosis Scientific and Research Foundation (PI: Tremlett, EGID: 2636).

RAM receives research funding from: CIHR, Research Manitoba, Multiple Sclerosis Society of Canada, Multiple Sclerosis Scientific Foundation, Crohn’s and Colitis Canada, National Multiple Sclerosis Society, CMSC, and US Department of Defense. She is supported by the Waugh Family Chair in Multiple Sclerosis.

JF was part‐funded through research grants held by HT, including The Multiple Sclerosis Scientific and Research Foundation (PI: Tremlett, EGID: 2636).

## Supporting information


**Data S1**. Phenotyping participants and relevant data sources.
**Tables S1–S3**. Phylum (1.1, 1.2), Genus (2.1, 2.2) and species‐level (3.1, 3.2) differences in the gut microbial communities by individual amplicon sequence variants (ASVs) for participants with: pediatric‐onset multiple sclerosis (MS) [disease‐modifying drug (DMD) exposed and naïve], acquired demyelinating syndromes, and unaffected controls, expressed as rate ratios.
**File S1**. Metagenomic predictions (PICRUSt2).
**Table S4**. The relative abundance of 193 predicted metagenomic pathways and related comparisons between the unaffected controls (*n* = 36), ADS participants (*n* = 41), and MS cases (all MS cases, *n* = 32, then by DMD exposure status [exposed, *n* = 23 or naïve, *n* = 9]).
**Table S5**. Cohort characteristics and associations with the gut microbiota alpha and beta diversity metrics in the Canada‐USA cohort.
**Tables S6 and S7**. Genus (6.1, 6.2) and species‐level (7.1, 7.2) differences in the gut microbial communities by individual amplicon sequence variants (ASVs) for USA‐only cohort participants with: pediatric‐onset multiple sclerosis (MS) [disease‐modifying drug (DMD)‐exposed and naïve], and unaffected controls, expressed as rate ratios.
**File S2**. Gut microbiota network analysis.
**Figure S1**. Annotated gut microbiota network analysis plots (genus‐level) based on stool samples from pediatric‐onset MS cases (DMD naïve and exposed), ADS and unaffected controls.
