# Supplementary material for: The gut microbiota in pediatric multiple sclerosis and demyelinating syndromes
Source: Ann Clin Transl Neurol. 2021 Dec 9;8(12):2252–69. doi: 10.1002/acn3.51476 (PMC8670321; doi:10.1002/acn3.51476)
Supplement: Supplementary file 1 — Data S1. Phenotyping participants and relevant data sources. Tables S1–S3. Phylum (1.1, 1.2), Genus (2.1, 2.2) and species‐level (3.1, 3.2) differences in the gut microbial communities by individual amplicon sequence variants (ASVs) for participants with: pediatric‐onset multiple sclerosis (MS) [disease‐modifying drug (DMD) exposed and naïve], acquired demyelinating syndromes, and unaffected controls, expressed as rate ratios. File S1. Metagenomic predictions (PICRUSt2). Table S4. The relative abundance of 193 predicted metagenomic pathways and related comparisons between the unaffected controls (n = 36), ADS participants (n = 41), and MS cases (all MS cases, n = 32, then by DMD exposure status [exposed, n = 23 or naïve, n = 9]). Table S5. Cohort characteristics and associations with the gut microbiota alpha and beta diversity metrics in the Canada‐USA cohort. Tables S6 and S7. Genus (6.1, 6.2) and species‐level (7.1, 7.2) differences in the gut microbial communities by individual amplicon sequence variants (ASVs) for USA‐only cohort participants with: pediatric‐onset multiple sclerosis (MS) [disease‐modifying drug (DMD)‐exposed and naïve], and unaffected controls, expressed as rate ratios. File S2. Gut microbiota network analysis. Figure S1. Annotated gut microbiota network analysis plots (genus‐level) based on stool samples from pediatric‐onset MS cases (DMD naïve and exposed), ADS and unaffected controls. [file ACN3-8-2252-s001.pdf]

Supplementary Tables 1 to 3. Phylum (1.1, 1.2), Genus (2.1, 2.2) and species-level (3.1, 3.2) differences in the gut microbial communities by individual amplicon sequence variants (ASVs) for participants with: paediatric-onset multiple sclerosis (MS) [disease-modifying drug (DMD) exposed and naïve], acquired demyelinating syndromes, and unaffected controls, expressed as rate ratios

Table Key: ADS=acquired demyelinating syndromes, ASV=amplicon sequence variant, CI=confidence intervals; DMD=disease-modifying drug, MS=paediatric-onset multiple sclerosis; MS DMD- = DMD naïve, i.e., at the time of the stool sample the MS case has never been exposed to a DMD, DMD+ = DMD exposed, i.e., the MS case had ever been exposed to a DMD at the time of the stool sample, Ref = reference group

Each Table shows rate ratios (RRs) and corresponding 95%CIs. The unadjusted and age and sex adjusted RRs are derived from a single negative binomial regression model each (two models in total per table), with only the estimates reaching nominal significance (p<0.05) for at least one group comparison shown. Unless otherwise specified, the second group forms the reference value.

Bold: p<0.05, ***Bold and italics: p & q<0.05,*** q-values represent false discovery rate adjusted p-values, and were based on the adjusted model, using a conservative approach. For example, the model which included the 4 participant groups (MS DMD naïve, MS DMD exposed, ADS, controls) results in six comparisons; these six comparisons were used to derive the q-values.
↓ = Depleted taxa (p<0.05), ↑ enriched taxa (p<0.05), * both p & q <0.05

Table 1.1: Phylum-level differences in the gut microbial communities: participants with MS, and ADS compared to unaffected controls

		Rate ratios (95%CIs) unadjusted			Rate ratios (95%CIs) age and sex adjusted					
Taxa Identifier (for internal use)	**Phylum**	**MS cases (vs ADS)**	**MS cases (vs controls)**	**ADS (vs controls)**	**MS cases (vs ADS)**	**MS cases (vs controls)**	**ADS (vs controls)**	*MS cases (vs ADS)*	*MS cases (vs controls)*	*ADS (vs controls)*
PHYLUM _1	Firmicutes (Combined: 3419)	1.12(0.78,1.61)	0.54(0.37,0.79);p=0.00 142	0.49(0.34,0.69);p=0. 00006	1.29(0.89,1.87)	0.66(0.46,0.95);p=0.02646;q=0. 03673	0.51(0.36,0.72);p=0.00013;q=0.0 0117		↓*	↓*
PHYLUM _3	Verrucomicrobi a (Combined: 64)	7.52(1.53,36.93);p=0. 01291	15.79(3.07,81.27);p=0. 00097	2.10(0.45,9.79)	5.91(1.11,31.38);p=0.03709;q =0.03778	13.92(2.63,73.76);p=0.00197;q =0.00901	2.36(0.50,11.17)	↑*	↑*	
PHYLUM _4	Bacteroidetes (Combined: 731)	0.78(0.49,1.25)	1.16(0.71,1.87)	1.48(0.94,2.33)	0.81(0.50,1.32)	1.14(0.70,1.85)	1.40(0.89,2.21)			
PHYLUM _5	Actinobacteria (Combined: 228)	0.95(0.61,1.49)	0.59(0.37,0.93);p=0.02 221	0.62(0.40,0.95);p=0. 02672	0.93(0.58,1.47)	0.57(0.36,0.91);p=0.01748;q=0. 03205	0.62(0.40,0.95);p=0.02805;q=0.0 3673		↓*	↓*
PHYLUM _6	Proteobacteria (Combined: 226)	1.25(0.76,2.05)	1.30(0.77,2.17)	1.04(0.64,1.69)	1.08(0.64,1.81)	1.21(0.72,2.02)	1.12(0.69,1.81)			
PHYLUM _7	Patescibacteria (Combined: 13)	4.43(1.70,11.56);p=0. 00234	2.03(0.77,5.33)	0.46(0.18,1.17)	4.18(1.56,11.17);p=0.00434;q =0.01326	2.01(0.76,5.26)	0.48(0.19,1.22)	↑*		
PHYLUM _9	Tenericutes (Combined: 62)	3.21(0.22,46.22)	2.38(0.15,37.09)	0.74(0.06,9.83)						
PHYLUM _10	Cyanobacteria (Combined: 26)	0.05(0.01,0.27);p=0.0 0058	0.15(0.02,0.90);p=0.03 84	3.22(0.60,17.34)						
PHYLUM _14	Fusobacteria (Combined: 18)	0.03(0.00,0.57);p=0.0 192	0.31(0.02,5.84)	9.19(0.61,138.23)	0.02(0.00,0.31);p=0.00643;q= 0.01474	0.28(0.01,5.83)	18.68(1.21,287.53);p=0.03581;q =0.03778	↓*		↑*
PHYLUM _15	Epsilonbacterae ota (Combined: 5)	0.50(0.06,4.44)	0.42(0.05,3.80)	0.83(0.13,5.52)	0.41(0.04,4.29)	0.31(0.03,3.07)	0.75(0.11,5.07)			

Table 1.2: Phylum-level differences in the gut microbial communities: participants with MS, and ADS compared to unaffected controls for individual ASVs by DMD exposure

		Rate ratios (95%CIs) unadjusted					Rate ratios (95%CIs) age and sex adjusted									
		Ref.: MS cases DMD-		Ref.: MS cases DMD+	Ref.: Controls		Ref.: MS cases DMD-		Ref.: MS cases DMD+	Ref.: Controls						
Taxa Identifier (for internal use)	Phylum	MS cases DMD+	ADS	ADS	MS cases DMD-	MS cases DMD+	MS cases DMD+	ADS	ADS	MS cases DMD-	MS cases DMD+	MS DMD+ (vs MS DMD-)	ADS (vs MS DMD-)	ADS (vs MS DMD+)	MS DMD- (vs control)	MS DMD+ (vs control)
PHYLUM_1	Firmicutes (Combined: 3419)	1.24(0.68,2.27)	1.05(0.60,1.85)	0.85(0.57,1.26)	0.46(0.26,0.82);p=0.00848	0.58(0.38,0.87);p=0.00829	1.23(0.68,2.20)	0.90(0.51,1.59)	0.74(0.49,1.10)	0.57(0.32,1.00);p=0.04834	0.69(0.47,1.04)				↓	
PHYLUM_3	Verrucomicrobia (Combined: 64)	183.10(14.01,2393.74);p=0.00007	17.53(1.58,194.54);p=0.0197	0.10(0.02,0.53);p=0.00691	0.12(0.01,1.37)	21.92(3.83,125.42);p=0.00052	191.97(14.60,2523.91);p=0.00006;q=0.00212	21.48(1.79,258.50);p=0.01566	0.11(0.02,0.65);p=0.0146	0.11(0.01,1.34)	21.64(3.72,126.05);p=0.00063;q=0.00751	↑*	↑	↓		↑*
PHYLUM_4	Bacteroidetes (Combined: 731)	1.08(0.49,2.36)	1.35(0.65,2.81)	1.25(0.75,2.10)	1.09(0.52,2.30)	1.18(0.69,2.01)	1.02(0.47,2.23)	1.25(0.59,2.66)	1.23(0.72,2.09)	1.12(0.53,2.36)	1.14(0.67,1.95)					
PHYLUM_5	Actinobacteria (Combined: 228)	1.28(0.61,2.68)	1.26(0.63,2.52)	0.98(0.60,1.60)	0.49(0.24,0.98);p=0.04422	0.63(0.38,1.03)	1.35(0.65,2.83)	1.36(0.67,2.77)	1.01(0.61,1.66)	0.45(0.22,0.92);p=0.02827	0.61(0.37,1.01)				↓	
PHYLUM_6	Proteobacteria (Combined: 226)	0.47(0.21,1.06)	0.50(0.23,1.07)	1.06(0.61,1.83)	2.10(0.96,4.58)	0.98(0.56,1.72)	0.46(0.20,1.03)	0.57(0.26,1.25)	1.24(0.71,2.17)	2.01(0.92,4.39)	0.92(0.52,1.60)					
PHYLUM_7	Patescibacteria (Combined: 13)	2.28(0.49,10.71)	0.43(0.10,1.88)	0.19(0.07,0.54);p=0.00176	1.06(0.24,4.64)	2.42(0.85,6.90)	1.90(0.42,8.70)	0.39(0.09,1.75)	0.21(0.07,0.60);p=0.00348;q=0.03337	1.22(0.28,5.30)	2.31(0.81,6.55)			↓*		
PHYLUM_9	Tenericutes (Combined: 62)	118362120699.79(0.00,Inf)	26535289132.77(0.00,Inf)	0.22(0.01,3.61)	0.00(0.00,Inf)	3.31(0.19,56.91)										
PHYLUM_10	Cyanobacteria (Combined: 26)	0.10(0.01,1.85)	7.74(0.54,110.54)	74.34(10.77,513.29);p=0.00001	0.42(0.03,6.14)	0.04(0.01,0.31);p=0.00186	0.15(0.01,2.40)	6.41(0.44,92.61)	43.99(6.32,306.31);p=0.00013;q=0.00212	0.48(0.03,6.76)	0.07(0.01,0.49);p=0.0072			↑*		↓
PHYLUM_13	Deinococcus-Thermus (Combined: 5)	1.47(0.00,534.37)	0.22(0.00,65.66)	0.15(0.00,9.29)	1.25(0.00,344.59)	1.84(0.03,103.30)										
PHYLUM_14	Fusobacteria (Combined: 18)	1.03(0.01,126.19)	30.51(0.35,2652.65)	29.69(1.26,697.71);p=0.03527	0.30(0.00,27.94)	0.31(0.01,7.97)										
PHYLUM_15	Epsilonbacteraeota (Combined: 5)	0.00(0.00,Inf)	0.67(0.04,10.24)	15795974353928.10(0.00,Inf)	1.27(0.08,19.86)	0.00(0.00,Inf)	0.00(0.00,Inf)	0.63(0.04,10.90)	1665168266598124.00(0.00,Inf)	1.29(0.08,21.23)	0.00(0.00,Inf)					

Table 2.1: Genus-level differences in the gut microbial communities: participants with MS, and monophasic demyelinating disease compared to controls for individual ASVs

		Rate ratios (95%CIs) unadjusted			Rate ratios (95%CIs) age and sex adjusted					
Taxa Identifier (for internal use)	Genus-level findings<br>Shown as: Phylum;Class;Order;Family;Genus (combined # of ASVs)	MS cases (vs ADS)	MS cases (vs controls)	ADS (vs controls)	MS cases (vs ADS)	MS cases (vs controls)	ADS (vs controls)	MS cases (vs ADS)	MS cases (vs controls)	ADS (vs controls)
GENUS_1	Firmicutes;D_2__Bacilli;D_3__Lactobacillales;D_4__Lactobacillaceae;D_5__Lactobacillus (Combined: 49)	3.35(0.98,11.42)	0.93(0.26,3.27)	0.28(0.08,0.90);p=0.03354	1.91(0.53,6.85)	0.65(0.18,2.32)	0.34(0.10,1.12)			
GENUS_3	Firmicutes;D_2__Bacilli;D_3__Lactobacillales;D_4__Streptococcaceae;D_5__Streptococcus (Combined: 34)	1.57(0.91,2.72)	1.19(0.67,2.09)	0.76(0.44,1.29)	1.67(0.94,2.98)	1.24(0.70,2.21)	0.74(0.43,1.27)			
GENUS_4	Firmicutes;D_2__Clostridia;D_3__Clostridiales;D_4__Lachnospiraceae;D_5__Blautia (Combined: 99)	0.90(0.63,1.30)	1.04(0.72,1.51)	1.15(0.81,1.64)	0.91(0.62,1.34)	1.05(0.72,1.54)	1.15(0.81,1.64)			
GENUS_6	Firmicutes;D_2__Clostridia;D_3__Clostridiales;D_4__Lachnospiraceae;D_5__Coprococcus 3 (Combined: 25)	1.07(0.48,2.39)	0.98(0.43,2.24)	0.91(0.42,1.99)	1.00(0.44,2.31)	1.02(0.44,2.34)	1.01(0.46,2.20)			
GENUS_7	Firmicutes;D_2__Clostridia;D_3__Clostridiales;D_4__Lachnospiraceae;D_5__Anaerostipes (Combined: 33)	0.80(0.53,1.23)	0.84(0.54,1.30)	1.05(0.69,1.58)	0.90(0.58,1.40)	0.89(0.58,1.39)	1.00(0.66,1.50)			
GENUS_8	Bacteroidetes;D_2__Bacteroidia;D_3__Bacteroidales;D_4__Bacteroidaceae;D_5__Bacteroides (Combined: 282)	0.51(0.29,0.87);p=0.01453	1.55(0.89,2.72)	3.06(1.81,5.19);p=0.00003	0.53(0.30,0.93);p=0.02598	1.48(0.85,2.59)	2.80(1.66,4.72);p=0.00011;q=0.00889	↓		↑*
GENUS_9	Firmicutes;D_2__Clostridia;D_3__Clostridiales;D_4__Lachnospiraceae;D_5__Dorea (Combined: 34)	1.19(0.73,1.96)	1.19(0.72,1.99)	1.00(0.62,1.62)	1.14(0.68,1.92)	1.20(0.71,2.01)	1.05(0.64,1.70)			
GENUS_10	Firmicutes;D_2__Clostridia;D_3__Clostridiales;D_4__Lachnospiraceae;D_5__[Eubacterium] hallii group (Combined: 67)	0.92(0.62,1.36)	0.85(0.57,1.28)	0.93(0.63,1.36)	0.81(0.54,1.22)	0.78(0.52,1.17)	0.96(0.66,1.40)			
GENUS_13	Firmicutes;D_2__Clostridia;D_3__Clostridiales;D_4__Christensenellaceae;D_5__Christensenellaceae R-7 group (Combined: 135)	1.01(0.50,2.02)	0.67(0.32,1.36)	0.66(0.34,1.30)	0.90(0.43,1.87)	0.58(0.28,1.20)	0.64(0.33,1.27)			
GENUS_14	Bacteroidetes;D_2__Bacteroidia;D_3__Bacteroidales;D_4__Rikenellaceae;D_5__Alistipes (Combined: 48)	0.78(0.39,1.55)	0.84(0.42,1.70)	1.08(0.56,2.09)	0.83(0.41,1.71)	0.88(0.43,1.81)	1.06(0.54,2.07)			
GENUS_15	Actinobacteria;D_2__Actinobacteria;D_3__Bifidobacteriales;D_4__Bifidobacteriaceae;D_5__Bifidobacterium (Combined: 39)	0.97(0.48,1.92)	0.36(0.18,0.73);p=0.00456	0.37(0.19,0.72);p=0.00357	0.82(0.40,1.68)	0.34(0.17,0.69);p=0.00309	0.41(0.21,0.81);p=0.00948		↓	↓
GENUS_17	Firmicutes;D_2__Clostridia;D_3__Clostridiales;D_4__Ruminococcaceae;D_5__Ruminococcaceae UCG-014 (Combined: 60)	1.38(0.22,8.62)	1.47(0.22,9.65)	1.06(0.18,6.23)	1.07(0.16,7.24)	1.21(0.18,8.20)	1.14(0.19,6.77)			
GENUS_18	Firmicutes;D_2__Clostridia;D_3__Clostridiales;D_4__Ruminococcaceae;D_5__Ruminiclostridium 5 (Combined: 79)	0.94(0.57,1.57)	0.83(0.49,1.39)	0.87(0.54,1.43)	1.08(0.64,1.83)	0.97(0.58,1.64)	0.90(0.55,1.46)			
GENUS_19	Actinobacteria;D_2__Coriobacteriia;D_3__Coriobacteriales;D_4__Coriobacteriaceae;D_5__Collinsella (Combined: 22)	0.71(0.30,1.68)	0.59(0.24,1.41)	0.82(0.36,1.87)	0.68(0.28,1.66)	0.59(0.24,1.43)	0.86(0.37,1.97)			
GENUS_20	Firmicutes;D_2__Clostridia;D_3__Clostridiales;D_4__Lachnospiraceae;D_5__uncultured (Combined: 56)	0.58(0.39,0.86);p=0.00671	0.99(0.67,1.49)	1.71(1.17,2.49);p=0.00558	0.48(0.32,0.71);p=0.00024;q=0.01289	0.90(0.61,1.34)	1.89(1.31,2.73);p=0.00072;q=0.02138	↓*		↑*

GENUS_21	Firmicutes;D_2__Clostridia;D_3__Clostridiales;D_4__Ruminococcaceae;D_5__Ruminococcaceae NK4A214 group (Combined: 28)	0.89(0.34,2.32)	0.20(0.07,0.52);p=0.00116	0.22(0.09,0.55);p=0.0013	0.85(0.32,2.29)	0.20(0.08,0.55);p=0.00162;q=0.0443	0.24(0.10,0.60);p=0.00241;q=0.04643		↓*	↓*
GENUS_23	Firmicutes;D_2__Clostridia;D_3__Clostridiales;D_4__Ruminococcaceae;D_5__[Eubacterium] coprostanoligenes group (Combined: 79)	1.36(0.65,2.85)	0.99(0.46,2.11)	0.73(0.35,1.49)	1.42(0.65,3.08)	1.00(0.46,2.18)	0.71(0.34,1.46)			
GENUS_24	Firmicutes;D_2__Negativicutes;D_3__Selenomonadales;D_4__Veillonellaceae;D_5__Dialister (Combined: 22)	1.00(0.36,2.73)	1.02(0.36,2.88)	1.02(0.39,2.72)	0.91(0.32,2.60)	1.03(0.36,2.95)	1.14(0.43,3.03)			
GENUS_25	Firmicutes;D_2__Clostridia;D_3__Clostridiales;D_4__Lachnospiraceae;D_5__[Ruminococcus] torques group (Combined: 64)	1.09(0.71,1.67)	1.11(0.71,1.72)	1.02(0.67,1.54)	1.10(0.70,1.71)	1.09(0.70,1.71)	1.00(0.66,1.51)			
GENUS_26	Firmicutes;D_2__Clostridia;D_3__Clostridiales;D_4__Ruminococcaceae;D_5__Ruminococcus 1 (Combined: 51)	0.62(0.27,1.45)	0.65(0.27,1.56)	1.05(0.46,2.37)	0.75(0.31,1.79)	0.66(0.28,1.56)	0.87(0.39,1.97)			
GENUS_27	Firmicutes;D_2__Clostridia;D_3__Clostridiales;D_4__Lachnospiraceae;D_5__Marvinbryantia (Combined: 10)	0.64(0.21,1.97)	0.68(0.21,2.19)	1.07(0.36,3.20)	0.69(0.22,2.24)	0.64(0.20,2.06)	0.92(0.31,2.75)			
GENUS_28	Firmicutes;D_2__Clostridia;D_3__Clostridiales;D_4__Ruminococcaceae;D_5__Ruminococcaceae UCG-005 (Combined: 62)	0.88(0.40,1.93)	0.66(0.29,1.47)	0.75(0.35,1.59)	0.84(0.37,1.92)	0.66(0.29,1.50)	0.78(0.37,1.68)			
GENUS_30	Firmicutes;D_2__Negativicutes;D_3__Selenomonadales;D_4__Acidaminococcaceae;D_5__Phascolarctobacterium (Combined: 18)	0.20(0.04,1.06)	0.63(0.11,3.57)	3.19(0.62,16.31)	0.23(0.04,1.34)	0.64(0.11,3.74)	2.80(0.54,14.53)			
GENUS_31	Firmicutes;D_2__Clostridia;D_3__Clostridiales;D_4__Lachnospiraceae;D_5__Agathobacter (Combined: 51)	2.96(1.46,5.99);p=0.00251	2.19(1.06,4.52);p=0.03416	0.74(0.37,1.46)	2.51(1.23,5.13);p=0.01175	2.13(1.04,4.36);p=0.03782	0.85(0.44,1.66)	↑	↑	
GENUS_32	Firmicutes;D_2__Clostridia;D_3__Clostridiales;D_4__Ruminococcaceae;D_5__Faecalibacterium (Combined: 131)	0.75(0.45,1.25)	0.52(0.31,0.88);p=0.0156	0.70(0.43,1.14)	0.85(0.50,1.45)	0.56(0.33,0.95);p=0.03176	0.66(0.40,1.08)		↓	
GENUS_33	Firmicutes;D_2__Clostridia;D_3__Clostridiales;D_4__Ruminococcaceae;D_5__Intestinimonas (Combined: 17)	1.31(0.50,3.45)	1.46(0.54,3.95)	1.11(0.43,2.85)	1.48(0.54,4.04)	1.53(0.56,4.18)	1.03(0.40,2.65)			
GENUS_34	Firmicutes;D_2__Clostridia;D_3__Clostridiales;D_4__Lachnospiraceae;D_5__[Ruminococcus] gauvreauii group (Combined: 24)	1.15(0.53,2.47)	0.93(0.42,2.05)	0.81(0.39,1.71)	1.26(0.57,2.81)	0.98(0.44,2.17)	0.77(0.37,1.63)			
GENUS_35	Firmicutes;D_2__Clostridia;D_3__Clostridiales;D_4__Ruminococcaceae;D_5__Subdoligranulum (Combined: 61)	1.58(0.91,2.74)	1.04(0.59,1.84)	0.66(0.39,1.12)	1.65(0.93,2.94)	1.07(0.60,1.90)	0.65(0.38,1.10)			
GENUS_36	Bacteroidetes;D_2__Bacteroidia;D_3__Bacteroidales;D_4__Tannerellaceae;D_5__Parabacteroides (Combined: 75)	0.76(0.39,1.48)	0.68(0.34,1.35)	0.90(0.47,1.71)	0.66(0.33,1.33)	0.63(0.32,1.27)	0.96(0.50,1.83)			
GENUS_38	Firmicutes;D_2__Clostridia;D_3__Clostridiales;D_4__Family XIII;D_5__[Eubacterium] nodatum group (Combined: 12)	1.47(0.43,4.96)	2.56(0.73,9.05)	1.75(0.53,5.76)	1.96(0.57,6.74)	3.19(0.92,11.11)	1.63(0.50,5.29)			
GENUS_41	Firmicutes;D_2__Clostridia;D_3__Clostridiales;D_4__Ruminococcaceae;D_5__uncultured (Combined: 88)	0.28(0.15,0.54);p=0.00013	0.90(0.46,1.75)	3.19(1.70,5.99);p=0.00031	0.30(0.15,0.60);p=0.00056;q=0.01846	0.93(0.47,1.84)	3.08(1.64,5.80);p=0.0005;q=0.01846	↓*		↑*
GENUS_42	Firmicutes;D_2__Clostridia;D_3__Clostridiales;D_4__Family XIII;D_5__Family XIII AD3011 group (Combined: 40)	0.55(0.31,0.98);p=0.04183	0.60(0.33,1.10)	1.10(0.63,1.94)	0.59(0.32,1.08)	0.64(0.35,1.17)	1.09(0.62,1.91)			
GENUS_43	Firmicutes;D_2__Clostridia;D_3__Clostridiales;D_4__Lachnospiraceae;D_5__Eisenbergiella (Combined: 14)	2.46(0.91,6.63)	2.56(0.92,7.11)	1.04(0.40,2.73)	2.13(0.76,5.96)	2.36(0.84,6.59)	1.11(0.42,2.89)			
GENUS_44	Firmicutes;D_2__Bacilli;D_3__Bacillales;D_4__Family XI;D_5__Gemella (Combined: 3)	2.68(1.02,7.06);p=0.04583	1.31(0.49,3.50)	0.49(0.19,1.25)	2.83(1.05,7.59);p=0.03952	1.49(0.56,3.97)	0.53(0.21,1.33)	↑		

GENUS_45	Firmicutes;D_2__Clostridia;D_3__Clostridiales;D_4__Lachnospiraceae;D_5__Fusicatenibacter (Combined: 27)	0.91(0.54,1.52)	0.73(0.43,1.24)	0.80(0.49,1.32)	0.92(0.54,1.58)	0.74(0.43,1.26)	0.80(0.48,1.32)			
GENUS_47	Firmicutes;D_2__Clostridia;D_3__Clostridiales;D_4__Ruminococcaceae;D_5__Ruminiclostridium 9 (Combined: 29)	0.78(0.39,1.58)	0.64(0.31,1.33)	0.82(0.42,1.63)	0.91(0.44,1.89)	0.70(0.34,1.46)	0.77(0.39,1.52)			
GENUS_48	Firmicutes;D_2__Clostridia;D_3__Clostridiales;D_4__Peptococcaceae;D_5__Peptococcus (Combined: 13)	1.59(0.28,9.17)	0.20(0.03,1.20)	0.13(0.02,0.69);p=0.01644	1.26(0.21,7.75)	0.25(0.04,1.48)	0.20(0.04,1.05)			
GENUS_49	Firmicutes;D_2__Clostridia;D_3__Clostridiales;D_4__Peptostreptococcaceae;D_5__Romboutsia (Combined: 4)	1.76(0.89,3.45)	1.32(0.66,2.64)	0.75(0.39,1.44)	1.59(0.78,3.22)	1.25(0.62,2.53)	0.79(0.41,1.52)			
GENUS_50	Bacteroidetes;D_2__Bacteroidia;D_3__Bacteroidales;D_4__Marinifilaceae;D_5__Odoribacter (Combined: 16)	0.88(0.37,2.12)	0.71(0.29,1.75)	0.80(0.34,1.87)	0.96(0.38,2.39)	0.81(0.33,2.03)	0.85(0.36,2.00)			
GENUS_51	Firmicutes;D_2__Clostridia;D_3__Clostridiales;D_4__Lachnospiraceae;D_5__Roseburia (Combined: 32)	0.52(0.24,1.10)	0.37(0.17,0.82);p=0.01346	0.73(0.35,1.51)	0.52(0.24,1.15)	0.38(0.17,0.84);p=0.01676	0.73(0.35,1.53)		↓	
GENUS_52	Firmicutes;D_2__Clostridia;D_3__Clostridiales;D_4__Ruminococcaceae;D_5__Ruminococcaceae UCG-002 (Combined: 24)	0.89(0.41,1.91)	0.81(0.37,1.79)	0.92(0.44,1.93)	0.73(0.33,1.62)	0.67(0.30,1.48)	0.91(0.44,1.92)			
GENUS_53	Firmicutes;D_2__Clostridia;D_3__Clostridiales;D_4__Ruminococcaceae;D_5__UBA1819 (Combined: 7)	1.22(0.65,2.32)	1.10(0.57,2.13)	0.90(0.48,1.67)	1.17(0.60,2.26)	1.12(0.58,2.16)	0.96(0.52,1.77)			
GENUS_54	Firmicutes;D_2__Erysipelotrichia;D_3__Erysipelotrichales;D_4__Erysipelotrichaceae;D_5__[Clostridium] innocuum group (Combined: 5)	0.70(0.35,1.42)	1.26(0.61,2.61)	1.79(0.90,3.55)	0.70(0.33,1.47)	1.18(0.56,2.48)	1.69(0.85,3.37)			
GENUS_55	Actinobacteria;D_2__Coriobacteriia;D_3__Coriobacteriales;D_4__Eggerthellaceae;D_5__Slackia (Combined: 7)	1.34(0.18,10.09)	0.42(0.05,3.32)	0.31(0.04,2.19)	1.92(0.23,15.87)	0.40(0.05,3.29)	0.21(0.03,1.49)			
GENUS_56	Firmicutes;D_2__Clostridia;D_3__Clostridiales;D_4__Ruminococcaceae;D_5__Ruminococcaceae UCG-013 (Combined: 53)	0.72(0.42,1.23)	0.88(0.51,1.53)	1.22(0.73,2.05)	0.63(0.36,1.10)	0.82(0.47,1.43)	1.30(0.78,2.18)			
GENUS_57	Firmicutes;D_2__Clostridia;D_3__Clostridiales;D_4__Lachnospiraceae;D_5__Hungatella (Combined: 6)	0.90(0.31,2.62)	2.00(0.67,6.01)	2.22(0.79,6.23)	1.27(0.42,3.84)	2.25(0.75,6.79)	1.77(0.63,4.96)			
GENUS_58	Firmicutes;D_2__Clostridia;D_3__Clostridiales;D_4__Lachnospiraceae;D_5__Lachnospiraceae FCS020 group (Combined: 16)	1.45(0.79,2.65)	1.14(0.61,2.13)	0.79(0.44,1.42)	1.38(0.73,2.59)	1.12(0.59,2.10)	0.81(0.45,1.46)			
GENUS_59	Firmicutes;D_2__Negativicutes;D_3__Selenomonadales;D_4__Veillonellaceae;D_5__Veillonella (Combined: 18)	0.49(0.20,1.21)	0.29(0.12,0.73);p=0.00863	0.59(0.25,1.40)	0.71(0.29,1.74)	0.26(0.10,0.63);p=0.00306	0.36(0.16,0.84);p=0.01803		↓	↓
GENUS_60	Firmicutes;D_2__Clostridia;D_3__Clostridiales;D_4__Lachnospiraceae;D_5__GCA-900066575 (Combined: 21)	0.84(0.41,1.70)	0.81(0.39,1.68)	0.97(0.49,1.92)	0.95(0.46,1.99)	0.85(0.41,1.77)	0.89(0.45,1.76)			
GENUS_61	Firmicutes;D_2__Clostridia;D_3__Clostridiales;D_4__Ruminococcaceae;D_5__Ruminiclostridium 6 (Combined: 10)	0.43(0.14,1.36)	0.73(0.22,2.41)	1.71(0.56,5.21)	0.68(0.23,2.03)	1.09(0.36,3.27)	1.61(0.58,4.47)			
GENUS_62	Firmicutes;D_2__Clostridia;D_3__Clostridiales;D_4__Lachnospiraceae;D_5__CAG-56 (Combined: 16)	2.11(0.60,7.40)	1.62(0.44,5.92)	0.77(0.23,2.60)	1.47(0.40,5.39)	1.33(0.36,4.87)	0.91(0.27,3.05)			
GENUS_63	Firmicutes;D_2__Clostridia;D_3__Clostridiales;D_4__Ruminococcaceae;D_5__Negativibacillus (Combined: 8)	1.88(0.49,7.16)	1.10(0.28,4.36)	0.59(0.16,2.14)						
GENUS_65	Firmicutes;D_2__Clostridia;D_3__Clostridiales;D_4__Ruminococcaceae (Combined: 126)	1.71(0.85,3.44)	1.10(0.54,2.27)	0.65(0.33,1.27)	1.49(0.72,3.08)	1.07(0.52,2.21)	0.72(0.37,1.42)			

GENUS_66	Firmicutes;D_2__Clostridia;D_3__Clostridiales;D_4__Family XIII;D_5__Family XIII UCG-001 (Combined: 9)	0.72(0.26,1.98)	1.13(0.40,3.20)	1.56(0.59,4.15)	0.73(0.26,2.12)	1.08(0.37,3.10)	1.47(0.55,3.93)			
GENUS_67	Firmicutes;D_2__Clostridia;D_3__Clostridiales;D_4__Lachnospiraceae (Combined: 215)	2.05(1.09,3.85);p=0.026	2.68(1.40,5.13);p=0.00303	1.31(0.71,2.41)	1.98(1.02,3.85);p=0.04337	2.62(1.35,5.08);p=0.00437	1.32(0.71,2.45)	↑	↑	
GENUS_68	Proteobacteria;D_2__Deltaproteobacteria;D_3__Desulfovibrionales;D_4__Desulfovibrionaceae;D_5__Bilophila (Combined: 20)	0.86(0.38,1.98)	1.33(0.56,3.14)	1.54(0.69,3.45)	0.70(0.29,1.66)	1.31(0.55,3.11)	1.87(0.83,4.21)			
GENUS_69	Firmicutes;D_2__Clostridia;D_3__Clostridiales;D_4__Peptococcaceae;D_5__uncultured (Combined: 26)	0.45(0.12,1.65)	0.33(0.09,1.25)	0.73(0.21,2.55)	0.42(0.11,1.60)	0.30(0.08,1.16)	0.73(0.21,2.53)			
GENUS_70	Actinobacteria;D_2__Actinobacteria;D_3__Actinomycetales;D_4__Actinomycetaceae;D_5__Actinomyces (Combined: 17)	2.11(1.14,3.93);p=0.01826	0.81(0.43,1.52)	0.38(0.21,0.70);p=0.00168	2.71(1.43,5.14);p=0.00222;q=0.04554	1.12(0.59,2.11)	0.41(0.23,0.75);p=0.00355	↑*		↓
GENUS_71	Firmicutes;D_2__Clostridia;D_3__Clostridiales;D_4__Family XIII;D_5__[Eubacterium] brachy group (Combined: 10)	0.96(0.39,2.36)	0.75(0.30,1.90)	0.79(0.33,1.88)	0.94(0.36,2.41)	0.77(0.30,1.97)	0.82(0.34,1.98)			
GENUS_72	Firmicutes;D_2__Clostridia;D_3__Clostridiales;D_4__Lachnospiraceae;D_5__Lachnoclostridium (Combined: 69)	0.67(0.40,1.11)	1.12(0.66,1.87)	1.67(1.02,2.72);p=0.03951	0.54(0.33,0.90);p=0.01717	1.02(0.62,1.68)	1.87(1.17,2.98);p=0.00865	↓		↑
GENUS_73	Firmicutes;D_2__Clostridia;D_3__Clostridiales;D_4__Lachnospiraceae;D_5__[Eubacterium] eligens group (Combined: 28)	1.24(0.40,3.83)	0.15(0.05,0.47);p=0.00117	0.12(0.04,0.35);p=0.00013	0.96(0.29,3.13)	0.12(0.04,0.40);p=0.00053;q=0.01846	0.13(0.04,0.39);p=0.00028;q=0.01289		↓*	↓*
GENUS_74	Firmicutes;D_2__Bacilli;D_3__Lactobacillales;D_4__Carnobacteriaceae;D_5__Granulicatella (Combined: 3)	2.29(1.03,5.09);p=0.04262	1.33(0.59,3.02)	0.58(0.27,1.27)	2.41(1.04,5.56);p=0.03974	1.39(0.60,3.18)	0.58(0.26,1.26)	↑		
GENUS_75	Firmicutes;D_2__Clostridia;D_3__Clostridiales;D_4__Ruminococcaceae;D_5__Ruminococcaceae UCG-010 (Combined: 65)	0.39(0.12,1.31)	0.40(0.12,1.41)	1.04(0.32,3.35)	0.38(0.11,1.38)	0.40(0.11,1.43)	1.04(0.32,3.41)			
GENUS_76	Firmicutes;D_2__Bacilli;D_3__Lactobacillales;D_4__Streptococcaceae;D_5__Lactococcus (Combined: 9)	3.20(1.14,8.96);p=0.02667	4.34(1.50,12.54);p=0.00666	1.36(0.50,3.68)	1.99(0.69,5.75)	3.44(1.19,9.93);p=0.02265	1.73(0.64,4.67)		↑	
GENUS_77	Firmicutes;D_2__Clostridia;D_3__Clostridiales;D_4__Clostridiales vadinBB60 group;D_5__uncultured organism (Combined: 4)	0.10(0.01,0.73);p=0.02335	0.26(0.03,1.96)	2.52(0.38,16.89)	0.14(0.02,1.09)	0.20(0.03,1.60)	1.47(0.22,10.01)			
GENUS_78	Firmicutes;D_2__Clostridia;D_3__Clostridiales;D_4__Lachnospiraceae;D_5__Lachnospiraceae NK4A136 group (Combined: 44)	0.60(0.32,1.13)	0.65(0.34,1.24)	1.07(0.58,1.97)	0.63(0.33,1.22)	0.70(0.36,1.34)	1.10(0.60,2.03)			
GENUS_79	Firmicutes;D_2__Erysipelotrichia;D_3__Erysipelotrichales;D_4__Erysipelotrichaceae;D_5__Erysipelotrichaceae UCG-003 (Combined: 11)	0.83(0.36,1.90)	0.62(0.27,1.45)	0.75(0.34,1.66)	0.69(0.29,1.63)	0.56(0.24,1.32)	0.81(0.36,1.80)			
GENUS_81	Firmicutes;D_2__Clostridia;D_3__Clostridiales;D_4__Peptostreptococcaceae;D_5__Peptostreptococcus (Combined: 2)	2.82(0.44,17.89)	2.75(0.41,18.45)	0.98(0.16,6.06)						
GENUS_82	Firmicutes;D_2__Clostridia;D_3__Clostridiales;D_4__Defluviitaleaceae;D_5__Defluviitaleaceae UCG-011 (Combined: 12)	0.95(0.40,2.27)	1.03(0.42,2.54)	1.09(0.47,2.54)	0.86(0.35,2.12)	0.90(0.36,2.20)	1.04(0.45,2.40)			
GENUS_83	Firmicutes;D_2__Clostridia;D_3__Clostridiales;D_4__Clostridiales vadinBB60 group;D_5__uncultured Thermoanaerobacterales bacterium (Combined: 2)	1.39(0.08,22.93)	0.35(0.02,6.20)	0.25(0.02,3.77)						
GENUS_84	Firmicutes;D_2__Erysipelotrichia;D_3__Erysipelotrichales;D_4__Erysipelotrichaceae;D_5__Holdemania (Combined: 12)	1.17(0.64,2.14)	1.52(0.82,2.83)	1.30(0.72,2.33)	1.29(0.69,2.41)	1.59(0.85,2.98)	1.24(0.68,2.23)			
GENUS_85	Firmicutes;D_2__Clostridia;D_3__Clostridiales;D_4__Lachnospiraceae;D_5__Sellimonas (Combined: 13)	0.81(0.26,2.51)	2.95(0.92,9.54)	3.66(1.22,11.03);p=0.02098	0.54(0.17,1.74)	2.65(0.81,8.61)	4.93(1.64,14.83);p=0.00448			↑

GENUS_86	Firmicutes;D_2__Clostridia;D_3__Clostridiales;D_4__Christensenellaceae;D_5__uncultured (Combined: 32)	0.87(0.38,2.02)	0.74(0.31,1.75)	0.85(0.38,1.91)	1.11(0.47,2.63)	0.83(0.35,1.97)	0.75(0.33,1.68)			
GENUS_88	Firmicutes;D_2__Clostridia;D_3__Clostridiales;D_4__Ruminococcaceae;D_5__DTU089 (Combined: 4)	0.72(0.35,1.48)	1.44(0.68,3.03)	2.00(0.99,4.03)						
GENUS_89	Actinobacteria;D_2__Coriobacteriia;D_3__Coriobacteriales;D_4__Coriobacteriales Incertae Sedis;D_5__uncultured (Combined: 11)	1.32(0.31,5.53)	1.77(0.40,7.77)	1.35(0.34,5.40)						
GENUS_90	Firmicutes;D_2__Clostridia;D_3__Clostridiales;D_4__Ruminococcaceae;D_5__Anaerotruncus (Combined: 8)	0.68(0.33,1.41)	0.94(0.44,2.00)	1.38(0.68,2.81)	0.71(0.33,1.52)	0.91(0.42,1.97)	1.29(0.63,2.63)			
GENUS_91	Actinobacteria;D_2__Coriobacteriia;D_3__Coriobacteriales;D_4__Eggerthellaceae (Combined: 8)	0.47(0.18,1.27)	0.85(0.31,2.34)	1.78(0.69,4.62)	0.48(0.17,1.34)	0.69(0.25,1.92)	1.43(0.55,3.70)			
GENUS_92	Firmicutes;D_2__Clostridia;D_3__Clostridiales;D_4__Lachnospiraceae;D_5__[Eubacterium] ventriosum group (Combined: 30)	1.20(0.59,2.44)	0.81(0.39,1.68)	0.67(0.34,1.34)	1.21(0.57,2.53)	0.75(0.36,1.57)	0.62(0.31,1.24)			
GENUS_93	Proteobacteria;D_2__Gammaproteobacteria;D_3__Pseudomonadales;D_4__Pseudomonadaceae;D_5__Pseudomonas (Combined: 9)	11.13(3.06,40.47);p=0.00025	3.24(0.88,11.85)	0.29(0.08,1.03)	16.46(4.33,62.52);p=0.00004;q=0.00641	5.52(1.51,20.21);p=0.00993	0.34(0.09,1.19)	↑*	↑	
GENUS_94	Firmicutes;D_2__Clostridia;D_3__Clostridiales;D_4__Ruminococcaceae;D_5__Fournierella (Combined: 13)	0.40(0.11,1.41)	0.56(0.15,2.04)	1.40(0.41,4.73)						
GENUS_95	Firmicutes;D_2__Clostridia;D_3__Clostridiales;D_4__Ruminococcaceae;D_5__Angelakisella (Combined: 6)	1.13(0.45,2.81)	0.89(0.35,2.28)	0.79(0.33,1.92)						
GENUS_96	Firmicutes;D_2__Erysipelotrichia;D_3__Erysipelotrichales;D_4__Erysipelotrichaceae;D_5__Erysipelatoclostridium (Combined: 9)	1.35(0.70,2.58)	2.14(1.10,4.18);p=0.02604	1.59(0.84,2.98)	1.31(0.66,2.58)	2.18(1.10,4.30);p=0.02505	1.67(0.88,3.15)		↑	
GENUS_97	Actinobacteria;D_2__Coriobacteriia;D_3__Coriobacteriales;D_4__Eggerthellaceae;D_5__Gordonibacter (Combined: 10)	1.30(0.71,2.38)	1.06(0.57,1.97)	0.81(0.45,1.46)	1.43(0.77,2.68)	1.11(0.60,2.08)	0.78(0.43,1.40)			
GENUS_98	Firmicutes;D_2__Clostridia;D_3__Clostridiales;D_4__Lachnospiraceae;D_5__Lachnospiraceae UCG-010 (Combined: 18)	0.60(0.21,1.68)	0.48(0.16,1.38)	0.80(0.29,2.17)	0.41(0.14,1.20)	0.50(0.17,1.45)	1.21(0.45,3.30)			
GENUS_99	Firmicutes;D_2__Clostridia;D_3__Clostridiales;D_4__Lachnospiraceae;D_5__[Eubacterium] xylanophilum group (Combined: 20)	1.00(0.28,3.52)	0.59(0.16,2.15)	0.59(0.17,1.99)	1.25(0.33,4.66)	0.64(0.17,2.39)	0.51(0.15,1.76)			
GENUS_100	Firmicutes;D_2__Clostridia;D_3__Clostridiales;D_4__Lachnospiraceae;D_5__GCA-900066755 (Combined: 2)	0.56(0.18,1.74)	1.21(0.37,3.95)	2.16(0.72,6.49)	0.70(0.22,2.26)	1.31(0.40,4.32)	1.87(0.62,5.63)			
GENUS_102	Firmicutes;D_2__Clostridia;D_3__DTU014;D_4__uncultured bacterium;D_5__ (Combined: 2)	0.51(0.11,2.40)	0.20(0.04,0.95);p=0.04326	0.38(0.09,1.66)						
GENUS_103	Firmicutes;D_2__Clostridia;D_3__Clostridiales;D_4__Ruminococcaceae;D_5__Ruminococcaceae UCG-003 (Combined: 12)	0.43(0.17,1.08)	0.26(0.10,0.66);p=0.00483	0.60(0.25,1.45)	0.35(0.14,0.90);p=0.02967	0.23(0.09,0.59);p=0.00217;q=0.04554	0.65(0.27,1.57)	↓	↓*	
GENUS_104	Firmicutes;D_2__Clostridia;D_3__Clostridiales;D_4__Ruminococcaceae;D_5__Hydrogenoanaerobacterium (Combined: 5)	0.55(0.11,2.73)	0.30(0.06,1.51)	0.54(0.12,2.36)	0.54(0.10,2.89)	0.30(0.06,1.54)	0.55(0.12,2.42)			
GENUS_105	Firmicutes;D_2__Clostridia;D_3__Clostridiales;D_4__Christensenellaceae;D_5__Catabacter (Combined: 6)	0.98(0.30,3.25)	0.56(0.16,1.91)	0.57(0.18,1.81)	0.98(0.30,3.21)	0.82(0.25,2.68)	0.84(0.28,2.53)			
GENUS_106	Firmicutes;D_2__Clostridia;D_3__Clostridiales;D_4__Ruminococcaceae;D_5__Ruminococcaceae UCG-007 (Combined: 2)	4.06(0.63,26.23)	0.73(0.12,4.47)	0.18(0.03,1.09)	8.50(1.15,63.06);p=0.03626	0.76(0.13,4.67)	0.09(0.01,0.58);p=0.011	↑		↓

GENUS_108	Proteobacteria;D_2__Deltaproteobacteria;D_3__Desulfovibrionales;D_4__Desulfovibrionaceae;D_5__Desulfovibrio (Combined: 11)	2.50(0.35,17.83)	1.52(0.20,11.49)	0.61(0.09,4.08)	2.20(0.29,16.43)	0.74(0.10,5.53)	0.34(0.05,2.20)			
GENUS_109	Firmicutes;D_2__Erysipelotrichia;D_3__Erysipelotrichales;D_4__Erysipelotrichaceae;D_5__Dielma (Combined: 5)	0.49(0.16,1.47)	3.27(1.02,10.47);p=0.04569	6.74(2.26,20.08);p=0.00062	0.36(0.12,1.14)	1.60(0.50,5.08)	4.41(1.50,12.95);p=0.00698			↑
GENUS_110	Firmicutes;D_2__Clostridia;D_3__Clostridiales;D_4__Lachnospiraceae;D_5__Lachnospira (Combined: 35)	0.86(0.40,1.88)	0.57(0.26,1.27)	0.66(0.31,1.40)	0.75(0.33,1.70)	0.53(0.23,1.19)	0.70(0.33,1.49)			
GENUS_111	Firmicutes;D_2__Bacilli;D_3__Lactobacillales;D_4__Leuconostocaceae;D_5__Leuconostoc (Combined: 7)	0.87(0.13,5.82)	0.83(0.12,5.86)	0.95(0.15,6.01)	0.41(0.06,2.80)	0.39(0.06,2.63)	0.95(0.16,5.59)			
GENUS_112	Firmicutes;D_2__Bacilli;D_3__Bacillales;D_4__Staphylococcaceae;D_5__Staphylococcus (Combined: 4)	1.13(0.26,4.89)	0.75(0.17,3.38)	0.67(0.16,2.75)	1.31(0.29,6.04)	0.89(0.20,4.05)	0.68(0.16,2.80)			
GENUS_113	Firmicutes;D_2__Clostridia;D_3__Clostridiales;D_4__Ruminococcaceae;D_5__Candidatus Soleaferrea (Combined: 20)	0.72(0.41,1.27)	1.00(0.56,1.79)	1.38(0.80,2.40)	0.82(0.46,1.49)	1.06(0.59,1.92)	1.29(0.74,2.24)			
GENUS_114	Firmicutes;D_2__Clostridia;D_3__Clostridiales;D_4__Family XIII;D_5__Mogibacterium (Combined: 5)	20.68(3.09,138.39);p=0.00179	12.87(1.84,90.26);p=0.01013	0.62(0.10,3.99)	11.43(1.59,82.42);p=0.01562	8.89(1.24,63.67);p=0.02956	0.78(0.12,5.01)	↑	↑	
GENUS_115	Firmicutes;D_2__Clostridia;D_3__Clostridiales;D_4__Clostridiales vadinBB60 group;D_5__uncultured bacterium (Combined: 38)	1.33(0.25,7.07)	0.15(0.03,0.86);p=0.03329	0.12(0.02,0.59);p=0.00915						
GENUS_116	Patescibacteria;D_2__Saccharimonadia;D_3__Saccharimonadales;D_4__Saccharimonadaceae (Combined: 1)	3.92(1.11,13.80);p=0.03338	1.10(0.31,3.88)	0.28(0.08,0.95);p=0.04183	4.49(1.23,16.35);p=0.02284	1.24(0.35,4.33)	0.28(0.08,0.92);p=0.03681	↑		↓
GENUS_117	Actinobacteria;D_2__Coriobacteriia;D_3__Coriobacteriales;D_4__Eggerthellaceae;D_5__Enterorhabdus (Combined: 9)	22.84(4.27,122.28);p=0.00026	1.41(0.27,7.35)	0.06(0.01,0.32);p=0.00082	26.16(4.59,149.13);p=0.00024;q=0.01289	2.02(0.38,10.67)	0.08(0.02,0.40);p=0.00212;q=0.04554	↑*		↓*
GENUS_119	Proteobacteria;D_2__Gammaproteobacteria;D_3__Betaproteobacteriales;D_4__Burkholderiaceae;D_5__Parasutterella (Combined: 15)	0.84(0.24,2.91)	1.52(0.42,5.46)	1.81(0.54,6.01)	0.61(0.17,2.23)	1.15(0.31,4.18)	1.88(0.56,6.28)			
GENUS_120	Firmicutes;D_2__Clostridia;D_3__Clostridiales;D_4__Lachnospiraceae;D_5__Lachnospiraceae ND3007 group (Combined: 16)	0.50(0.21,1.19)	0.59(0.24,1.45)	1.18(0.51,2.74)	0.44(0.18,1.09)	0.55(0.22,1.36)	1.25(0.53,2.91)			
GENUS_121	Bacteroidetes;D_2__Bacteroidia;D_3__Bacteroidales;D_4__Barnesiellaceae;D_5__Coprobacter (Combined: 7)				1.26(0.09,18.07)	0.30(0.02,4.29)	0.24(0.02,2.86)			
GENUS_122	Firmicutes;D_2__Clostridia;D_3__Clostridiales;D_4__Ruminococcaceae;D_5__Butyricicoccus (Combined: 41)	1.28(0.70,2.34)	0.79(0.42,1.48)	0.62(0.34,1.11)	1.34(0.71,2.53)	0.80(0.42,1.50)	0.59(0.33,1.07)			
GENUS_124	Firmicutes;D_2__Clostridia;D_3__Clostridiales;D_4__Ruminococcaceae;D_5__Oscillibacter (Combined: 28)	1.01(0.52,1.96)	1.29(0.65,2.56)	1.28(0.67,2.44)	0.81(0.41,1.63)	1.12(0.56,2.25)	1.38(0.72,2.64)			
GENUS_126	Firmicutes;D_2__Clostridia;D_3__Clostridiales;D_4__Lachnospiraceae;D_5__Lachnospiraceae UCG-008 (Combined: 6)	0.67(0.27,1.66)	0.43(0.17,1.08)	0.63(0.27,1.51)	0.49(0.19,1.24)	0.34(0.14,0.87);p=0.02364	0.70(0.30,1.66)		↓	
GENUS_127	Firmicutes;D_2__Clostridia;D_3__Clostridiales;D_4__Lachnospiraceae;D_5__Lachnospiraceae UCG-001 (Combined: 23)	0.79(0.20,3.13)	1.98(0.48,8.21)	2.52(0.66,9.57)	0.76(0.18,3.23)	2.04(0.48,8.65)	2.68(0.70,10.34)			
GENUS_128	Firmicutes;D_2__Clostridia;D_3__Clostridiales;D_4__Lachnospiraceae;D_5__Lachnospiraceae UCG-004 (Combined: 29)	0.53(0.23,1.26)	0.38(0.16,0.92);p=0.03295	0.71(0.31,1.64)	0.38(0.15,0.92);p=0.03172	0.30(0.12,0.73);p=0.00767	0.79(0.34,1.82)	↓	↓	
GENUS_129	Firmicutes;D_2__Clostridia;D_3__Clostridiales;D_4__Clostridiales vadinBB60 group;D_5__gut metagenome (Combined: 14)	0.13(0.04,0.51);p=0.00298	0.21(0.05,0.82);p=0.02519	1.57(0.44,5.59)	0.07(0.02,0.26);p=0.00011;q=0.00889	0.14(0.04,0.57);p=0.00547	2.17(0.62,7.62)	↓*	↓	

GENUS_131	Firmicutes;D_2__Clostridia;D_3__Clostridiales;D_4__Ruminococcaceae;D_5__Flavonifractor (Combined: 17)	1.11(0.63,1.93)	2.02(1.14,3.59);p=0.01602	1.83(1.07,3.14);p=0.02832	1.02(0.58,1.81)	1.73(0.98,3.05)	1.69(0.99,2.87)			
GENUS_132	Tenericutes;D_2__Mollicutes;D_3__Izimaplasmatales;D_4__uncultured organism;D_5__ (Combined: 2)	0.24(0.01,6.36)	0.07(0.00,2.04)	0.31(0.02,5.85)						
GENUS_134	Firmicutes;D_2__Clostridia;D_3__Clostridiales;D_4__Lachnospiraceae;D_5__Moryella (Combined: 3)	0.93(0.33,2.61)	0.68(0.24,1.97)	0.73(0.27,1.98)	0.74(0.26,2.13)	0.71(0.25,2.02)	0.95(0.36,2.53)			
GENUS_135	Firmicutes;D_2__Clostridia;D_3__Clostridiales;D_4__Ruminococcaceae;D_5__Ruminococcaceae UCG-009 (Combined: 13)	0.28(0.07,1.16)	0.40(0.09,1.69)	1.40(0.37,5.37)	0.31(0.07,1.35)	0.42(0.10,1.82)	1.35(0.35,5.25)			
GENUS_136	Actinobacteria;D_2__Coriobacteriia;D_3__Coriobacteriales;D_4__Atopobiaceae;D_5__uncultured (Combined: 5)	0.71(0.09,5.35)	0.15(0.02,1.17)	0.21(0.03,1.45)	0.66(0.08,5.42)	0.13(0.02,1.09)	0.20(0.03,1.43)			
GENUS_137	Proteobacteria;D_2__Gammaproteobacteria;D_3__Pasteurellales;D_4__Pasteurellaceae;D_5__Haemophilus (Combined: 3)	1.08(0.40,2.91)	1.23(0.44,3.42)	1.14(0.44,2.98)	0.81(0.29,2.27)	0.93(0.33,2.62)	1.15(0.44,3.01)			
GENUS_139	Firmicutes;D_2__Clostridia;D_3__Clostridiales;D_4__Ruminococcaceae;D_5__Oscillospira (Combined: 12)	0.70(0.26,1.85)	0.84(0.31,2.29)	1.20(0.47,3.08)	0.59(0.21,1.62)	0.75(0.27,2.05)	1.27(0.50,3.24)			
GENUS_140	Firmicutes;D_2__Clostridia;D_3__Clostridiales;D_4__Clostridiaceae 1;D_5__Clostridium sensu stricto 1 (Combined: 22)	1.69(0.76,3.75)	0.75(0.33,1.71)	0.45(0.21,0.97);p=0.04079	1.52(0.67,3.48)	0.85(0.37,1.94)	0.56(0.26,1.20)			
GENUS_141	Firmicutes;D_2__Clostridia;D_3__Clostridiales;D_4__Ruminococcaceae;D_5__Phocea (Combined: 3)	0.93(0.38,2.27)	0.97(0.39,2.42)	1.04(0.44,2.45)	0.83(0.33,2.10)	0.98(0.39,2.49)	1.18(0.50,2.81)			
GENUS_142	Proteobacteria;D_2__Gammaproteobacteria;D_3__Enterobacteriales;D_4__Enterobacteriaceae;D_5__Escherichia-Shigella (Combined: 10)	1.84(0.61,5.59)	1.01(0.32,3.19)	0.55(0.19,1.62)	1.26(0.40,3.95)	0.93(0.30,2.91)	0.74(0.25,2.14)			
GENUS_143	Firmicutes;D_2__Clostridia;D_3__Clostridiales (Combined: 29)	1.61(0.18,14.28)	0.50(0.05,4.68)	0.31(0.04,2.55)	3.34(0.34,32.56)	1.12(0.12,10.79)	0.33(0.04,2.79)			
GENUS_144	Actinobacteria;D_2__Coriobacteriia;D_3__Coriobacteriales;D_4__Eggerthellaceae;D_5__Eggerthella (Combined: 3)	0.94(0.49,1.81)	1.42(0.72,2.79)	1.51(0.80,2.85)	0.89(0.45,1.77)	1.23(0.62,2.43)	1.37(0.72,2.61)			
GENUS_145	Firmicutes;D_2__Clostridia;D_3__Clostridiales;D_4__Ruminococcaceae;D_5__Ruminiclostridium (Combined: 7)				1.02(0.31,3.33)	0.68(0.21,2.20)	0.67(0.22,2.00)			
GENUS_146	Firmicutes;D_2__Clostridia;D_3__Clostridiales;D_4__Lachnospiraceae;D_5__Anaerosporobacter (Combined: 3)	0.07(0.01,0.95);p=0.04588	0.02(0.00,0.24);p=0.00272	0.24(0.02,2.87)	0.01(0.00,0.19);p=0.00191;q=0.04554	0.00(0.00,0.02);p=0;q=0.00115	0.12(0.01,1.38)	↓*	↓*	
GENUS_147	Firmicutes;D_2__Clostridia;D_3__Clostridiales;D_4__Ruminococcaceae;D_5__GCA-900066225 (Combined: 20)	1.37(0.54,3.50)	0.65(0.25,1.68)	0.47(0.19,1.16)	1.41(0.53,3.75)	0.67(0.25,1.77)	0.48(0.19,1.18)			
GENUS_148	Firmicutes;D_2__Clostridia;D_3__Clostridiales;D_4__Ruminococcaceae;D_5__Pseudoflavonifractor (Combined: 3)	0.72(0.12,4.10)	2.10(0.33,13.40)	2.93(0.51,16.69)	0.64(0.10,3.96)	2.09(0.32,13.76)	3.27(0.57,18.85)			
GENUS_149	Firmicutes;D_2__Clostridia;D_3__Clostridiales;D_4__Lachnospiraceae;D_5__Tyzzerella (Combined: 7)	0.71(0.12,4.01)	9.80(1.61,59.76);p=0.0134	13.84(2.52,75.97);p=0.00249	0.91(0.15,5.54)	10.12(1.63,62.66);p=0.01288	11.10(2.02,60.96);p=0.00561		↑	↑
GENUS_152	Proteobacteria;D_2__Gammaproteobacteria;D_3__Betaproteobacteriales;D_4__Burkholderiaceae;D_5__Noviherbaspirillum (Combined: 4)	1.68(0.16,17.53)	0.37(0.04,3.79)	0.22(0.02,2.02)						
GENUS_153	Proteobacteria;D_2__Gammaproteobacteria;D_3__Betaproteobacteriales;D_4__Burkholderiaceae;D_5__Sutterella (Combined: 37)	0.31(0.07,1.27)	0.18(0.04,0.76);p=0.01975	0.57(0.14,2.25)	0.30(0.07,1.32)	0.18(0.04,0.78);p=0.02183	0.59(0.15,2.36)		↓	

GENUS_154	Firmicutes;D_2__Clostridia;D_3__Clostridiales;D_4__Peptostreptococcaceae;D_5__Intestinibacter (Combined: 5)	1.85(0.95,3.59)	1.26(0.63,2.49)	0.68(0.36,1.30)	2.07(1.04,4.12);p=0.03802	1.33(0.67,2.65)	0.64(0.34,1.22)	↑		
GENUS_155	Cyanobacteria;D_2__Oxyphotobacteria;D_3__Chloroplast (Combined: 12)	0.15(0.02,0.96);p=0.04543	0.98(0.14,6.72)	6.56(1.08,39.86); p=0.04101						
GENUS_156	Firmicutes;D_2__Clostridia;D_3__Clostridiales;D_4__Peptostreptococcaceae;D_5__Terrisporobacter (Combined: 2)	1.91(0.63,5.78)	0.47(0.15,1.47)	0.25(0.08,0.72);p=0.01068						
GENUS_157	Proteobacteria;D_2__Alphaproteobacteria;D_3__Caulobacterales;D_4__Caulobacteraceae;D_5__Brevundimonas (Combined: 1)	10.19(0.87,119.57)	2.15(0.24,19.31)	0.21(0.02,2.48)	8.19(0.75,89.73)	3.65(0.40,33.32)	0.45(0.04,4.65)			
GENUS_159	Firmicutes;D_2__Bacilli;D_3__Lactobacillales;D_4__Lactobacillaceae;D_5__Pediococcus (Combined: 3)	6.42(0.39,104.92)	0.46(0.03,7.88)	0.07(0.00,1.07)	4.57(0.25,82.63)	0.88(0.05,15.54)	0.19(0.01,2.88)			
GENUS_160	Actinobacteria;D_2__Actinobacteria;D_3__Micrococcales;D_4__Micrococcaceae;D_5__Rothia (Combined: 9)	1.36(0.48,3.88)	1.07(0.36,3.14)	0.79(0.28,2.17)	1.51(0.50,4.54)	1.12(0.38,3.36)	0.74(0.27,2.07)			
GENUS_162	Proteobacteria;D_2__Gammaproteobacteria;D_3__Pasteurellales;D_4__Pasteurellaceae (Combined: 1)	3.12(0.29,33.57)	1.38(0.12,15.88)	0.44(0.04,4.40)	2.82(0.24,32.63)	0.77(0.07,8.81)	0.27(0.03,2.67)			
GENUS_163	Firmicutes;D_2__Clostridia;D_3__Clostridiales;D_4__Lachnospiraceae;D_5__[Ruminococcus] gnavus group (Combined: 9)	0.95(0.30,2.99)	1.08(0.33,3.51)	1.14(0.38,3.44)	0.85(0.26,2.82)	1.05(0.32,3.47)	1.23(0.40,3.76)			
GENUS_168	Firmicutes;D_2__Erysipelotrichia;D_3__Erysipelotrichales;D_4__Erysipelotrichaceae;D_5__Turicibacter (Combined: 20)	2.35(1.11,5.01);p=0.02603	0.85(0.39,1.85)	0.36(0.17,0.75);p=0.0063	2.27(1.03,4.99);p=0.0418	0.77(0.35,1.70)	0.34(0.16,0.71);p=0.00416	↑		↓
GENUS_169	Firmicutes;D_2__Clostridia;D_3__Clostridiales;D_4__Lachnospiraceae;D_5__Tyzzerella 3 (Combined: 5)	2.23(0.21,23.50)	2.94(0.26,33.23)	1.32(0.13,12.90)						
GENUS_170	Firmicutes;D_2__Clostridia;D_3__Clostridiales;D_4__Lachnospiraceae;D_5__[Eubacterium] ruminantium group (Combined: 7)	0.63(0.06,6.36)	0.68(0.06,7.38)	1.08(0.12,10.11)						
GENUS_174	Firmicutes;D_2__Erysipelotrichia;D_3__Erysipelotrichales;D_4__Erysipelotrichaceae;D_5__Merdibacter (Combined: 3)	0.35(0.07,1.65)	0.81(0.16,4.05)	2.32(0.52,10.45)						
GENUS_175	Firmicutes;D_2__Clostridia;D_3__Clostridiales;D_4__Ruminococcaceae;D_5__Anaerofilum (Combined: 8)	1.37(0.33,5.74)	0.43(0.10,1.86)	0.32(0.08,1.25)	1.79(0.41,7.91)	0.58(0.13,2.50)	0.32(0.08,1.28)			
GENUS_176	Actinobacteria;D_2__Coriobacteriia;D_3__Coriobacteriales;D_4__Coriobacteriales Incertae Sedis;D_5__Raoultibacter (Combined: 2)	1.83(0.24,14.09)	0.80(0.10,6.30)	0.43(0.06,3.12)	1.99(0.24,16.67)	0.38(0.05,2.93)	0.19(0.03,1.35)			
GENUS_177	Firmicutes;D_2__Clostridia;D_3__Clostridiales;D_4__Eubacteriaceae;D_5__Eubacterium (Combined: 2)	8.21(0.98,68.72)	4.26(0.50,36.34)	0.52(0.06,4.27)	24.40(2.66,223.77);p=0.00472	10.13(1.22,84.43);p=0.03231	0.42(0.05,3.53)	↑	↑	
GENUS_182	Firmicutes;D_2__Clostridia;D_3__Clostridiales;D_4__Lachnospiraceae;D_5__[Eubacterium] fissicatena group (Combined: 8)	1.08(0.48,2.44)	1.21(0.52,2.80)	1.12(0.51,2.47)	1.23(0.52,2.88)	1.35(0.58,3.16)	1.10(0.49,2.44)			
GENUS_183	Firmicutes;D_2__Clostridia;D_3__Clostridiales;D_4__Lachnospiraceae;D_5__UC5-1-2E3 (Combined: 1)	0.56(0.10,3.25)	1.44(0.23,8.89)	2.57(0.46,14.21)						
GENUS_189	Firmicutes;D_2__Clostridia;D_3__Clostridiales;D_4__Peptostreptococcaceae;D_5__Paeniclostridium (Combined: 2)	9.05(1.07,76.64); p=0.04333	4.12(0.47,35.89)	0.46(0.06,3.71)	15.02(1.66,135.56);p=0.01578	5.24(0.62,44.42)	0.35(0.04,2.90)	↑		
GENUS_190	Firmicutes;D_2__Bacilli;D_3__Lactobacillales;D_4__Enterococcaceae;D_5__Enterococcus (Combined: 4)	8.81(0.70,110.71)	1.50(0.11,20.36)	0.17(0.01,1.98)						

GENUS_192	Actinobacteria;D_2__Actinobacteria;D_3__Bifidobacteriales;D_4__Bifidobacteriaceae;D_5__Alloscardovia (Combined: 1)	1.27(0.07,23.70)	0.64(0.03,12.33)	0.50(0.03,8.26)						
GENUS_193	Firmicutes;D_2__Erysipelotrichia;D_3__Erysipelotrichales;D_4__Erysipelotrichaceae;D_5__Faecalitalea (Combined: 5)	0.75(0.21,2.67)	1.90(0.51,7.01)	2.52(0.74,8.62)	0.80(0.21,3.01)	1.79(0.48,6.73)	2.23(0.65,7.69)			
GENUS_194	Bacteroidetes;D_2__Bacteroidia;D_3__Bacteroidales;D_4__Prevotellaceae;D_5__Prevotella 6 (Combined: 9)				0.01(0.00,0.35);p=0.01129	0.15(0.00,5.64)	15.55(0.79,307.51)	↓		
GENUS_195	Bacteroidetes;D_2__Bacteroidia;D_3__Bacteroidales;D_4__Porphyromonadaceae;D_5__Porphyromonas (Combined: 17)	1.04(0.19,5.77)	2.59(0.44,15.40)	2.50(0.47,13.41)	0.97(0.19,5.05)	2.64(0.49,14.32)	2.72(0.56,13.15)			
GENUS_196	Actinobacteria;D_2__Actinobacteria;D_3__Corynebacteriales;D_4__Corynebacteriaceae;D_5__Corynebacterium 1 (Combined: 8)	9.62(1.84,50.38); p=0.00739	6.77(1.25,36.50);p=0.02621	0.70(0.13,3.72)	8.04(1.49,43.40);p=0.01546	4.21(0.85,20.91)	0.52(0.10,2.83)	↑		
GENUS_197	Firmicutes;D_2__Clostridia;D_3__Clostridiales;D_4__Family XI;D_5__Ezakiella (Combined: 4)	0.31(0.06,1.61)	0.19(0.04,1.01)	0.61(0.13,2.85)	0.66(0.12,3.52)	0.40(0.08,2.13)	0.61(0.13,2.84)			
GENUS_200	Bacteroidetes;D_2__Bacteroidia;D_3__Bacteroidales;D_4__Prevotellaceae;D_5__Prevotella (Combined: 11)	0.20(0.03,1.60)	1.39(0.16,11.92)	6.89(0.93,51.22)						
GENUS_201	Proteobacteria;D_2__Alphaproteobacteria;D_3__Rhizobiales;D_4__Rhizobiaceae;D_5__Allorhizobium-Neorhizobium-Pararhizobium-Rhizobium (Combined: 2)	13.37(1.49,120.12);p=0.02063	4.05(0.60,27.51)	0.30(0.03,2.97)	19.04(1.69,214.34);p=0.01708	3.75(0.54,26.12)	0.20(0.02,2.12)	↑		
GENUS_202	Proteobacteria;D_2__Gammaproteobacteria;D_3__Betaproteobacteriales;D_4__Burkholderiaceae (Combined: 7)	0.73(0.04,12.82)	0.22(0.01,3.86)	0.30(0.02,4.17)						
GENUS_203	Firmicutes;D_2__Clostridia;D_3__Clostridiales;D_4__Family XI;D_5__Peptoniphilus (Combined: 8)	2.07(0.30,14.17)	3.38(0.46,24.66)	1.64(0.25,10.68)	2.52(0.34,18.67)	4.89(0.65,36.58)	1.94(0.30,12.76)			
GENUS_204	Firmicutes;D_2__Erysipelotrichia;D_3__Erysipelotrichales;D_4__Erysipelotrichaceae;D_5__Candidatus Stoquefichus (Combined: 3)	0.98(0.13,7.64)	8.47(1.00,71.31);p=0.04948	8.62(1.16,64.12); p=0.03534	2.74(0.34,21.69)	12.79(1.57,104.03);p=0.01717	4.68(0.66,33.05)		↑	
GENUS_205	Firmicutes;D_2__Clostridia;D_3__Clostridiales;D_4__Ruminococcaceae;D_5__Ruminococcaceae UCG-008 (Combined: 7)	0.38(0.04,3.87)	1.38(0.12,15.43)	3.65(0.38,34.97)	1.56(0.16,14.89)	3.64(0.37,36.19)	2.34(0.26,20.77)			
GENUS_206	Actinobacteria;D_2__Coriobacteriia;D_3__Coriobacteriales;D_4__Atopobiaceae;D_5__Atopobium (Combined: 8)	3.42(0.49,23.61)	0.84(0.12,5.71)	0.25(0.04,1.60)	3.52(0.47,26.28)	1.03(0.15,7.16)	0.29(0.04,1.90)			
GENUS_212	Firmicutes;D_2__Clostridia;D_3__Clostridiales;D_4__Lachnospiraceae;D_5__Shuttleworthia (Combined: 6)				0.45(0.07,2.98)	1.70(0.25,11.50)	3.80(0.64,22.51)			
GENUS_214	Firmicutes;D_2__Clostridia;D_3__Clostridiales;D_4__Eubacteriaceae;D_5__Anaerofustis (Combined: 6)	1.65(0.71,3.88)	2.25(0.93,5.46)	1.36(0.59,3.17)	1.98(0.82,4.75)	2.64(1.09,6.42);p=0.03182	1.34(0.58,3.09)		↑	
GENUS_217	Firmicutes;D_2__Clostridia;D_3__Clostridiales;D_4__Christensenellaceae;D_5__Christensenella (Combined: 1)	2.59(0.46,14.67)	1.95(0.33,11.53)	0.75(0.14,4.10)	1.96(0.32,11.99)	1.02(0.17,6.13)	0.52(0.10,2.85)			
GENUS_218	Firmicutes;D_2__Clostridia;D_3__Clostridiales;D_4__Peptostreptococcaceae (Combined: 6)	1.92(0.09,43.26)	1.14(0.05,27.65)	0.59(0.03,12.07)						
GENUS_231	Firmicutes;D_2__Clostridia;D_3__Clostridiales;D_4__Family XI;D_5__Parvimonas (Combined: 6)	2.89(0.73,11.52)	1.25(0.31,5.09)	0.43(0.11,1.66)	2.69(0.64,11.41)	1.35(0.32,5.63)	0.50(0.13,1.94)			
GENUS_234	Firmicutes;D_2__Clostridia;D_3__Clostridiales;D_4__Family XI;D_5__Finegoldia (Combined: 1)	9.78(1.07,89.54); p=0.04362	3.22(0.34,30.71)	0.33(0.04,2.84)	6.08(0.70,52.56)	4.77(0.56,40.97)	0.78(0.10,6.01)			

GENUS_236	Firmicutes;D_2__Clostridia;D_3__Clostridiales;D_4__Family XI;D_5__Anaerococcus (Combined: 6)	1.18(0.16,8.60)	1.15(0.15,8.88)	0.98(0.14,6.68)	0.91(0.12,6.84)	1.79(0.23,13.79)	1.98(0.30,13.16)			
GENUS_237	Actinobacteria;D_2__Actinobacteria;D_3__Corynebacteriales;D_4__Corynebacteriaceae;D_5__Corynebacterium (Combined: 1)	0.88(0.38,2.06)	0.74(0.31,1.78)	0.84(0.37,1.91)	0.94(0.39,2.22)	0.81(0.34,1.91)	0.86(0.39,1.92)			
GENUS_238	Firmicutes;D_2__Bacilli;D_3__Lactobacillales;D_4__Aerococcaceae;D_5__Abiotrophia (Combined: 1)				2.83(0.38,21.24)	3.65(0.47,28.52)	1.29(0.18,9.11)			
GENUS_239	Actinobacteria;D_2__Actinobacteria;D_3__Bifidobacteriales;D_4__Bifidobacteriaceae;D_5__Scardovia (Combined: 1)				3.76(0.32,44.48)	0.86(0.08,9.05)	0.23(0.02,2.32)			
GENUS_244	Firmicutes;D_2__Clostridia;D_3__Clostridiales;D_4__Lachnospiraceae;D_5__Lactonifactor (Combined: 2)	6.97(0.81,59.67)	8.86(0.95,82.23)	1.27(0.15,10.92)						
GENUS_245	Firmicutes;D_2__Clostridia;D_3__Clostridiales;D_4__Lachnospiraceae;D_5__Lachnospiraceae NC2004 group (Combined: 7)	3.08(0.38,24.88)	1.18(0.14,9.80)	0.38(0.05,2.91)	2.97(0.33,26.57)	1.10(0.13,9.59)	0.37(0.05,2.89)			
GENUS_247	Firmicutes;D_2__Clostridia;D_3__Clostridiales;D_4__Family XI;D_5__Murdochiella (Combined: 3)	0.08(0.00,2.63)	0.16(0.00,6.16)	2.10(0.09,49.49)						
GENUS_248	Firmicutes;D_2__Erysipelotrichia;D_3__Erysipelotrichales;D_4__Erysipelotrichaceae;D_5__Coprobacillus (Combined: 1)	1.24(0.23,6.57)	0.38(0.07,2.07)	0.30(0.06,1.51)	1.12(0.20,6.30)	0.47(0.08,2.60)	0.42(0.08,2.07)			
GENUS_252	Actinobacteria;D_2__Actinobacteria;D_3__Corynebacteriales;D_4__Corynebacteriaceae;D_5__Lawsonella (Combined: 1)	1.01(0.07,15.21)	0.19(0.01,2.59)	0.19(0.02,2.11)	0.97(0.05,17.62)	0.13(0.01,2.02)	0.14(0.01,1.64)			
GENUS_256	Firmicutes;D_2__Clostridia;D_3__Clostridiales;D_4__Ruminococcaceae;D_5__Ruminiclostridium 1 (Combined: 9)				3.15(0.31,32.01)	1.42(0.15,13.94)	0.45(0.05,3.95)			
GENUS_258	Firmicutes;D_2__Clostridia;D_3__Clostridiales;D_4__Ruminococcaceae;D_5__Papillibacter (Combined: 2)	1.39(0.37,5.22)	0.60(0.16,2.26)	0.43(0.12,1.53)	1.32(0.33,5.24)	0.65(0.17,2.50)	0.49(0.14,1.74)			
GENUS_259	Actinobacteria;D_2__Actinobacteria;D_3__Actinomycetales;D_4__Actinomycetaceae;D_5__Varibaculum (Combined: 2)	0.27(0.05,1.45)	0.18(0.03,0.98);p=0.04673	0.65(0.14,3.03)	0.26(0.04,1.48)	0.19(0.03,1.07)	0.73(0.16,3.45)			
GENUS_261	Proteobacteria;D_2__Gammaproteobacteria;D_3__Xanthomonadales;D_4__Xanthomonadaceae;D_5__Stenotrophomonas (Combined: 5)	8.00(0.73,87.65)	2.46(0.24,24.76)	0.31(0.03,3.32)						
GENUS_262	Firmicutes;D_2__Clostridia;D_3__Clostridiales;D_4__Ruminococcaceae;D_5__uncultured bacterium (Combined: 1)	1.08(0.17,6.99)	0.71(0.11,4.65)	0.66(0.11,3.83)	1.61(0.23,11.08)	0.92(0.14,6.00)	0.57(0.10,3.39)			
GENUS_263	Firmicutes;D_2__Clostridia;D_3__Clostridiales;D_4__Lachnospiraceae;D_5__Oribacterium (Combined: 8)	0.78(0.14,4.43)	0.54(0.09,3.12)	0.70(0.14,3.41)	0.46(0.07,3.16)	0.16(0.03,1.00)	0.35(0.07,1.71)			
GENUS_267	Actinobacteria;D_2__Actinobacteria;D_3__Actinomycetales;D_4__Actinomycetaceae;D_5__F0332 (Combined: 2)	0.42(0.08,2.25)	0.39(0.07,2.15)	0.92(0.20,4.31)	0.65(0.12,3.38)	0.53(0.10,2.74)	0.82(0.18,3.63)			
GENUS_271	Patescibacteria;D_2__Saccharimonadia;D_3__Saccharimonadales;D_4__Saccharimonadaceae;D_5__uncultured Candidatus Saccharibacteria bacterium (Combined: 1)	4.33(0.78,23.86)	3.41(0.60,19.27)	0.79(0.13,4.64)	4.05(0.68,24.28)	3.13(0.54,18.21)	0.77(0.13,4.62)			
GENUS_288	Firmicutes;D_2__Clostridia;D_3__Clostridiales;D_4__Lachnospiraceae;D_5__Tyzzerella 4 (Combined: 3)	1.81(0.25,13.08)	2.88(0.38,22.10)	1.59(0.23,10.80)	1.55(0.19,12.32)	2.29(0.29,18.15)	1.48(0.21,10.22)			
GENUS_302	Proteobacteria;D_2__Gammaproteobacteria;D_3__Pseudomonadales;D_4__Moraxellaceae;D_5__Acinetobacter (Combined: 8)				2.61(0.18,37.27)	0.36(0.03,4.03)	0.14(0.01,1.66)			

GENUS_309	Proteobacteria;D_2__Alphaproteobacteria;D_3__Azospirillales;D_4__Azospirillaceae;D_5__Azospirillum (Combined: 2)	0.00(0.00,Inf)	0.00(0.00,Inf)	0.69(0.05,9.29)							
GENUS_351	Firmicutes;D_2__Clostridia;D_3__Clostridiales;D_4__Peptostreptococcaceae;D_5__uncultured (Combined: 4)	6.99(0.12,391.41)	104.61(1.60,6856.69);p=0.02933	14.98(0.29,769.86)							
GENUS_365	Firmicutes;D_2__Clostridia;D_3__Clostridiales;D_4__Christensenellaceae (Combined: 4)	1.50(0.17,13.01)	0.33(0.04,2.60)	0.22(0.03,1.61)	1.89(0.18,19.45)	0.30(0.04,2.55)	0.16(0.02,1.21)				

Table 2.2: Genus-level differences in the gut microbial communities: participants with MS (DMD naïve and exposed), and monophasic demyelinating disease compared to controls for individual ASVs

		Rate ratios (95%CIs)<br><br>unadjusted					Rate ratios (95%CIs)<br><br>age and sex adjusted									
		Ref.: MS cases DMD-		Ref.: MS cases DMD+	Ref.: Controls		Ref.: MS cases DMD-		Ref.: MS cases DMD+	Ref.: Controls						
Taxa Identifier (for internal use)	Genus-level findings<br><br>Shown as: Phylum;Class;Order;Family;Genus (combined # of ASVs)	MS cases DMD+	ADS	ADS	MS cases DMD-	MS cases DMD+	MS cases DMD+	ADS	ADS	MS cases DMD-	MS cases DMD+	*MS DMD+ (vs MS DMD-)*	*ADS (vs MS DMD-)*	*ADS (vs MS DMD+)*	*MS DMD- (vs control)*	*MS DMD+ (vs control)*
GENUS_1	Firmicutes;D_2__Bacilli;D_3__Lactobacillales;D_4__Lactobacillaceae;D_5__Lactobacillus (Combined: 49)	0.69(0.09, 5.30)	0.23(0.03, 1.56)	0.34(0.09, 1.30)	1.19(0.17, 8.26)	0.82(0.21,3.28)	0.53(0.07,4.06)	0.37(0.05,2.62)	0.69(0.17,2.77)	0.98(0.14,6.92)	0.52(0.13,2.10)					
GENUS_3	Firmicutes;D_2__Bacilli;D_3__Lactobacillales;D_4__Streptococcaceae;D_5__Streptococcus (Combined: 34)	1.13(0.45, 2.81)	0.69(0.29, 1.63)	0.62(0.34, 1.13)	1.09(0.46, 2.59)	1.22(0.66,2.28)	1.17(0.47,2.92)	0.67(0.28,1.62)	0.57(0.31,1.07)	1.11(0.46,2.67)	1.30(0.69,2.42)					
GENUS_4	Firmicutes;D_2__Clostridia;D_3__Clostridiales;D_4__Lachnospiraceae;D_5__Blautia (Combined: 99)	1.41(0.77, 2.58)	1.43(0.81, 2.52)	1.01(0.68, 1.51)	0.80(0.45, 1.42)	1.13(0.75,1.71)	1.42(0.78,2.60)	1.43(0.80,2.56)	1.01(0.67,1.52)	0.80(0.45,1.44)	1.14(0.76,1.73)					
GENUS_6	Firmicutes;D_2__Clostridia;D_3__Clostridiales;D_4__Lachnospiraceae;D_5__Coprococcus 3 (Combined: 25)						1.21(0.32,4.58)	1.15(0.32,4.14)	0.95(0.38,2.34)	0.88(0.25,3.15)	1.07(0.43,2.65)					
GENUS_7	Firmicutes;D_2__Clostridia;D_3__Clostridiales;D_4__Lachnospiraceae;D_5__Anaerostipes (Combined: 33)	2.38(1.19, 4.76);p=0.01467	2.47(1.29, 4.74);p=0.00643	1.04(0.66, 1.65)	0.42(0.22, 0.82);p=0.01047	1.00(0.63,1.61)	2.28(1.14,4.54);p=0.0194	2.16(1.11,4.21);p=0.02345	0.95(0.59,1.52)	0.46(0.24,0.90);p=0.02251	1.05(0.66,1.69)	↑	↑		↓	
GENUS_8	Bacteroidetes;D_2__Bacteroidia;D_3__Bacteroidales;D_4__Bacteroidaceae;D_5__Bacteroides (Combined: 282)	1.06(0.43, 2.62)	2.05(0.88, 4.80)	1.94(1.06, 3.54);p=0.0307	1.49(0.63, 3.52)	1.58(0.85,2.92)	0.95(0.39,2.31)	1.82(0.77,4.29)	1.92(1.04,3.52);p=0.03607	1.54(0.66,3.62)	1.46(0.79,2.69)			↑		
GENUS_9	Firmicutes;D_2__Clostridia;D_3__Clostridiales;D_4__Lachnospiraceae;D_5__Dorea (Combined: 34)	0.98(0.43, 2.24)	0.82(0.38, 1.79)	0.84(0.49, 1.46)	1.21(0.55, 2.66)	1.19(0.68,2.08)	0.96(0.42,2.19)	0.85(0.38,1.89)	0.89(0.50,1.56)	1.24(0.56,2.74)	1.18(0.67,2.08)					
GENUS_10	Firmicutes;D_2__Clostridia;D_3__Clostridiales;D_4__Lachnospiraceae;D_5__[Eubacterium] hallii group (Combined: 67)	1.23(0.64, 2.37)	1.27(0.69, 2.34)	1.03(0.67, 1.59)	0.73(0.39, 1.36)	0.90(0.58,1.40)	1.28(0.67,2.46)	1.50(0.80,2.80)	1.16(0.75,1.81)	0.64(0.34,1.20)	0.83(0.53,1.29)					
GENU	Firmicutes;D_2__Clostridia;D_3__Clostridiales;D_4__Christensenellaceae;D_5__C	3.08(0.97, 9.73)	2.48(0.84, 7.27)	0.80(0.38, 1.72)	0.27(0.09, 0.79);p=0.01752	0.82(0.38,1.79)	3.08(0.98,9.69)	2.75(0.91,8.32)	0.89(0.41,1.95)	0.24(0.08,0.71);p=0.01006	0.72(0.33,1.59)				↓	

S_13	hristensenellaceae R-7 group (Combined: 135)															
GENUS_14	Bacteroidetes;D_2__Bacteroidia;D_3__Bacteroidales;D_4__Rikenellaceae;D_5__Alistipes (Combined: 48)	1.83(0.59,5.69)	2.05(0.71,5.92)	1.12(0.53,2.37)	0.53(0.18,1.55)	0.97(0.45,2.09)	1.83(0.59,5.72)	1.91(0.64,5.72)	1.04(0.48,2.26)	0.55(0.19,1.65)	1.01(0.46,2.20)					
GENUS_15	Actinobacteria;D_2__Actinobacteria;D_3__Bifidobacteriales;D_4__Bifidobacteriaceae;D_5__Bifidobacterium (Combined: 39)	1.28(0.41,4.03)	1.24(0.42,3.64)	0.97(0.45,2.08)	0.30(0.10,0.89);p=0.02941	0.38(0.17,0.83);p=0.01531	1.56(0.50,4.87)	1.73(0.57,5.18)	1.11(0.51,2.41)	0.24(0.08,0.72);p=0.0109	0.38(0.17,0.82);p=0.01398				↓	↓
GENUS_17	Firmicutes;D_2__Clostridia;D_3__Clostridiales;D_4__Ruminococcaceae;D_5__Ruminococcaceae UCG-014 (Combined: 60)	0.25(0.01,5.10)	0.33(0.02,5.65)	1.34(0.18,10.01)	3.20(0.18,56.40)	0.79(0.10,6.16)	0.26(0.01,5.32)	0.42(0.02,7.87)	1.64(0.21,13.01)	2.72(0.15,49.97)	0.70(0.09,5.57)					
GENUS_18	Firmicutes;D_2__Clostridia;D_3__Clostridiales;D_4__Ruminococcaceae;D_5__Ruminiclostridium 5 (Combined: 79)	3.08(1.34,7.04);p=0.00781	2.64(1.22,5.73);p=0.01409	0.86(0.50,1.49)	0.33(0.15,0.73);p=0.00579	1.02(0.58,1.79)	3.00(1.32,6.81);p=0.00868	2.27(1.03,5.02);p=0.04211	0.76(0.43,1.33)	0.40(0.18,0.87);p=0.02137	1.19(0.68,2.08)	↑	↑		↓	
GENUS_19	Actinobacteria;D_2__Coriobacteriia;D_3__Coriobacteriales;D_4__Coriobacteriaceae;D_5__Collinsella (Combined: 22)	0.59(0.14,2.44)	0.99(0.26,3.73)	1.67(0.65,4.28)	0.83(0.22,3.18)	0.49(0.19,1.28)	0.56(0.13,2.29)	0.99(0.25,3.87)	1.78(0.68,4.66)	0.87(0.22,3.40)	0.49(0.18,1.28)					
GENUS_20	Firmicutes;D_2__Clostridia;D_3__Clostridiales;D_4__Lachnospiraceae;D_5__uncultured (Combined: 56)	0.54(0.28,1.02)	1.15(0.63,2.09)	2.13(1.39,3.26);p=0.00049	1.49(0.81,2.73)	0.80(0.52,1.24)	0.58(0.31,1.07)	1.45(0.79,2.63)	2.51(1.64,3.83);p=0.00002;q=0.00729	1.30(0.71,2.35)	0.75(0.49,1.14)			↑*		
GENUS_21	Firmicutes;D_2__Clostridia;D_3__Clostridiales;D_4__Ruminococcaceae;D_5__Ruminococcaceae NK4A214 group (Combined: 28)	1.96(0.40,9.62)	1.89(0.43,8.39)	0.97(0.34,2.77)	0.12(0.03,0.52);p=0.00505	0.23(0.08,0.67);p=0.00702	2.34(0.49,11.25)	2.34(0.51,10.65)	1.00(0.34,2.92)	0.10(0.02,0.47);p=0.00314	0.24(0.08,0.71);p=0.00943				↓	↓
GENUS_23	Firmicutes;D_2__Clostridia;D_3__Clostridiales;D_4__Ruminococcaceae;D_5__[Eubacterium] coprostanoligenes group (Combined: 79)	1.06(0.31,3.66)	0.77(0.24,2.45)	0.72(0.32,1.64)	0.94(0.29,3.04)	1.00(0.43,2.32)	1.04(0.30,3.59)	0.73(0.22,2.41)	0.70(0.30,1.63)	0.97(0.30,3.19)	1.01(0.43,2.36)					
GENUS_24	Firmicutes;D_2__Negativicutes;D_3__Selenomonadales;D_4__Veillonellaceae;D_5__Dialister (Combined: 22)	0.42(0.08,2.26)	0.59(0.12,2.81)	1.39(0.46,4.20)	1.74(0.36,8.48)	0.74(0.24,2.30)	0.48(0.09,2.58)	0.68(0.14,3.41)	1.40(0.45,4.39)	1.65(0.33,8.20)	0.80(0.25,2.51)					
GENUS_25	Firmicutes;D_2__Clostridia;D_3__Clostridiales;D_4__Lachnospiraceae;D_5__[Ruminococcus] torques group (Combined: 64)	0.74(0.36,1.50)	0.74(0.38,1.45)	1.01(0.63,1.62)	1.37(0.70,2.68)	1.01(0.62,1.63)	0.76(0.37,1.54)	0.76(0.38,1.50)	1.00(0.61,1.62)	1.32(0.67,2.61)	1.00(0.62,1.63)					
GENUS_26	Firmicutes;D_2__Clostridia;D_3__Clostridiales;D_4__Ruminococcaceae;D_5__Ruminococcus 1 (Combined: 51)	1.11(0.27,4.52)	1.73(0.47,6.44)	1.56(0.61,3.95)	0.61(0.16,2.29)	0.67(0.26,1.74)	0.83(0.21,3.29)	1.16(0.31,4.42)	1.41(0.55,3.62)	0.75(0.20,2.83)	0.62(0.24,1.59)					

GENUS_27	Firmicutes;D_2__Clostridia;D_3__Clostridiales;D_4__Lachnospiraceae;D_5__Marvinbryantia (Combined: 10)	1.67(0.25,10.96)	2.32(0.40,13.51)	1.39(0.40,4.83)	0.46(0.08,2.74)	0.77(0.22,2.76)	2.08(0.32,13.33)	2.52(0.42,15.18)	1.21(0.34,4.31)	0.36(0.06,2.14)	0.75(0.21,2.66)					
GENUS_28	Firmicutes;D_2__Clostridia;D_3__Clostridiales;D_4__Ruminococcaceae;D_5__Ruminococcaceae UCG-005 (Combined: 62)	0.59(0.16,2.18)	0.80(0.24,2.71)	1.35(0.57,3.20)	0.93(0.27,3.19)	0.55(0.23,1.33)	0.60(0.16,2.20)	0.84(0.24,2.95)	1.40(0.58,3.41)	0.93(0.27,3.26)	0.56(0.23,1.36)					
GENUS_30	Firmicutes;D_2__Negativicutes;D_3__Selenomonadales;D_4__Acidaminococcaceae;D_5__Phascolarctobacterium (Combined: 18)	0.66(0.04,10.99)	3.84(0.28,53.19)	5.79(0.90,37.19)	0.83(0.06,11.89)	0.55(0.08,3.71)	0.73(0.04,12.21)	3.65(0.24,55.34)	5.00(0.73,34.12)	0.79(0.05,11.75)	0.58(0.08,3.96)					
GENUS_31	Firmicutes;D_2__Clostridia;D_3__Clostridiales;D_4__Lachnospiraceae;D_5__Agathobacter (Combined: 51)	1.40(0.43,4.52)	0.44(0.15,1.30)	0.31(0.14,0.68);p=0.00317	1.70(0.56,5.16)	2.38(1.07,5.28);p=0.03264	0.75(0.24,2.33)	0.33(0.11,0.99);p=0.0475	0.44(0.20,0.96);p=0.03894	2.60(0.87,7.74)	1.94(0.89,4.22)		↓	↓		
GENUS_32	Firmicutes;D_2__Clostridia;D_3__Clostridiales;D_4__Ruminococcaceae;D_5__Faecalibacterium (Combined: 131)	1.03(0.44,2.40)	1.36(0.62,3.01)	1.32(0.76,2.32)	0.51(0.23,1.15)	0.53(0.30,0.94);p=0.02962	0.98(0.42,2.30)	1.16(0.51,2.64)	1.18(0.66,2.11)	0.56(0.25,1.27)	0.56(0.31,0.99);p=0.04746					↓
GENUS_33	Firmicutes;D_2__Clostridia;D_3__Clostridiales;D_4__Ruminococcaceae;D_5__Intestinimonas (Combined: 17)	2.15(0.43,10.73)	1.39(0.31,6.28)	0.65(0.22,1.87)	0.80(0.17,3.70)	1.72(0.58,5.11)	1.98(0.40,9.79)	1.12(0.24,5.24)	0.56(0.19,1.67)	0.92(0.20,4.27)	1.82(0.61,5.41)					
GENUS_34	Firmicutes;D_2__Clostridia;D_3__Clostridiales;D_4__Lachnospiraceae;D_5__[Ruminococcus] gauvreauii group (Combined: 24)	3.70(1.05,13.04);p=0.04186	2.56(0.79,8.34)	0.69(0.30,1.60)	0.32(0.10,1.05)	1.17(0.50,2.76)	3.95(1.12,13.85);p=0.03214	2.48(0.74,8.32)	0.63(0.27,1.48)	0.31(0.09,1.04)	1.23(0.52,2.90)	↑				
GENUS_35	Firmicutes;D_2__Clostridia;D_3__Clostridiales;D_4__Ruminococcaceae;D_5__Subdoligranulum (Combined: 61)	1.40(0.56,3.51)	0.82(0.35,1.92)	0.58(0.32,1.07)	0.81(0.34,1.93)	1.13(0.61,2.11)	1.34(0.54,3.35)	0.75(0.31,1.83)	0.56(0.30,1.05)	0.86(0.36,2.06)	1.15(0.61,2.15)					
GENUS_36	Bacteroidetes;D_2__Bacteroidia;D_3__Bacteroidales;D_4__Tannerellaceae;D_5__Parabacteroides (Combined: 75)	0.51(0.17,1.55)	0.86(0.30,2.42)	1.67(0.80,3.48)	1.05(0.37,2.99)	0.54(0.25,1.14)	0.47(0.16,1.42)	0.95(0.33,2.73)	2.00(0.95,4.24)	1.02(0.35,2.92)	0.48(0.23,1.02)					
GENUS_38	Firmicutes;D_2__Clostridia;D_3__Clostridiales;D_4__Family XIII;D_5__[Eubacterium] nodatum group (Combined: 12)	1.39(0.18,10.46)	0.87(0.13,5.78)	0.63(0.16,2.40)	2.01(0.29,13.78)	2.79(0.70,11.10)	1.19(0.17,8.40)	0.58(0.09,3.86)	0.49(0.13,1.87)	2.82(0.43,18.68)	3.35(0.86,13.02)					
GENUS_41	Firmicutes;D_2__Clostridia;D_3__Clostridiales;D_4__Ruminococcaceae;D_5__uncultured (Combined: 88)	0.42(0.14,1.23)	2.07(0.76,5.66)	4.94(2.42,10.08);p=0.00001	1.54(0.56,4.26)	0.64(0.31,1.34)	0.43(0.15,1.25)	1.91(0.68,5.36)	4.46(2.14,9.27);p=0.00006;q=0.01176	1.60(0.57,4.47)	0.68(0.33,1.42)			↑*		
GENUS_42	Firmicutes;D_2__Clostridia;D_3__Clostridiales;D_4__Family XIII;D_5__Family XIII AD3011 group (Combined: 40)	1.11(0.42,2.92)	1.97(0.79,4.89)	1.78(0.94,3.39)	0.56(0.22,1.41)	0.62(0.32,1.20)	1.12(0.42,2.94)	1.85(0.73,4.70)	1.65(0.86,3.20)	0.59(0.23,1.48)	0.66(0.34,1.27)					

GENUS_43	Firmicutes;D_2__Clostridia;D_3__Clostridiales;D_4__Lachnospiraceae;D_5__Eisenbergiella (Combined: 14)	2.95(0.57,15.26)	0.98(0.21,4.56)	0.33(0.11,0.98);p=0.04677	1.07(0.22,5.08)	3.14(1.03,9.59);p=0.04433	2.65(0.52,13.49)	1.02(0.21,4.92)	0.39(0.13,1.17)	1.08(0.23,5.18)	2.87(0.94,8.73)					
GENUS_44	Firmicutes;D_2__Bacilli;D_3__Bacillales;D_4__Family XI;D_5__Gemella (Combined: 3)	1.58(0.32,7.77)	0.53(0.12,2.37)	0.33(0.12,0.97);p=0.04325	0.92(0.20,4.18)	1.46(0.50,4.29)	1.29(0.27,6.09)	0.43(0.09,1.93)	0.33(0.11,0.96);p=0.04252	1.23(0.28,5.48)	1.58(0.55,4.59)			↓		
GENUS_45	Firmicutes;D_2__Clostridia;D_3__Clostridiales;D_4__Lachnospiraceae;D_5__Fusicatenibacter (Combined: 27)	0.86(0.36,2.02)	0.99(0.44,2.21)	1.15(0.65,2.03)	0.81(0.36,1.83)	0.70(0.39,1.25)	0.85(0.36,2.01)	0.97(0.42,2.21)	1.14(0.63,2.04)	0.82(0.36,1.88)	0.70(0.39,1.26)					
GENUS_47	Firmicutes;D_2__Clostridia;D_3__Clostridiales;D_4__Ruminococcaceae;D_5__Ruminiclostridium 9 (Combined: 29)	0.83(0.26,2.67)	1.12(0.37,3.36)	1.36(0.62,2.95)	0.74(0.24,2.24)	0.61(0.27,1.35)	0.73(0.23,2.33)	0.88(0.28,2.71)	1.21(0.55,2.69)	0.87(0.28,2.67)	0.63(0.28,1.41)					
GENUS_48	Firmicutes;D_2__Clostridia;D_3__Clostridiales;D_4__Peptococcaceae;D_5__Peptococcus (Combined: 13)	0.56(0.03,10.06)	0.43(0.03,6.41)	0.77(0.11,5.30)	0.30(0.02,4.48)	0.17(0.02,1.18)	1.05(0.06,18.58)	0.82(0.05,13.12)	0.78(0.11,5.59)	0.24(0.02,3.74)	0.25(0.04,1.78)					
GENUS_49	Firmicutes;D_2__Clostridia;D_3__Clostridiales;D_4__Peptostreptococcaceae;D_5__Romboutsia (Combined: 4)	1.80(0.58,5.54)	0.90(0.31,2.57)	0.50(0.24,1.05)	0.84(0.29,2.43)	1.50(0.70,3.23)	1.93(0.63,5.94)	1.05(0.36,3.12)	0.55(0.25,1.17)	0.74(0.25,2.16)	1.42(0.66,3.07)					
GENUS_50	Bacteroidetes;D_2__Bacteroidia;D_3__Bacteroidales;D_4__Marinifilaceae;D_5__Odoribacter (Combined: 16)	0.46(0.11,1.98)	0.69(0.18,2.71)	1.50(0.57,3.94)	1.15(0.29,4.58)	0.53(0.20,1.43)	0.47(0.11,2.01)	0.64(0.16,2.59)	1.35(0.50,3.64)	1.32(0.33,5.32)	0.62(0.23,1.68)					
GENUS_51	Firmicutes;D_2__Clostridia;D_3__Clostridiales;D_4__Lachnospiraceae;D_5__Roseburia (Combined: 32)	1.92(0.55,6.77)	3.22(0.99,10.47)	1.68(0.73,3.86)	0.22(0.07,0.74);p=0.01417	0.43(0.18,1.02)	1.80(0.51,6.29)	3.07(0.92,10.32)	1.71(0.73,4.03)	0.24(0.07,0.80);p=0.02008	0.43(0.18,1.02)				↓	
GENUS_52	Firmicutes;D_2__Clostridia;D_3__Clostridiales;D_4__Ruminococcaceae;D_5__Ruminococcaceae UCG-002 (Combined: 24)	0.52(0.15,1.87)	0.74(0.22,2.44)	1.41(0.61,3.29)	1.24(0.37,4.14)	0.65(0.27,1.54)	0.55(0.15,1.93)	0.92(0.27,3.10)	1.68(0.71,3.98)	1.00(0.30,3.34)	0.54(0.23,1.29)					
GENUS_53	Firmicutes;D_2__Clostridia;D_3__Clostridiales;D_4__Ruminococcaceae;D_5__UBA1819 (Combined: 7)	0.83(0.29,2.41)	0.72(0.26,1.94)	0.86(0.43,1.75)	1.26(0.46,3.44)	1.04(0.51,2.15)	0.84(0.29,2.40)	0.76(0.28,2.09)	0.90(0.44,1.85)	1.26(0.46,3.45)	1.06(0.52,2.17)					
GENUS_54	Firmicutes;D_2__Erysipelotrichia;D_3__Erysipelotrichales;D_4__Erysipelotrichaceae;D_5__[Clostridium] innocuum group (Combined: 5)	0.86(0.27,2.80)	1.28(0.43,3.86)	1.49(0.68,3.25)	1.39(0.46,4.25)	1.20(0.54,2.68)	0.82(0.25,2.67)	1.24(0.40,3.86)	1.51(0.67,3.37)	1.36(0.44,4.21)	1.12(0.50,2.51)					
GENUS_55	Actinobacteria;D_2__Coriobacteriia;D_3__Coriobacteriales;D_4__Eggerthellaceae;D_5__Slackia (Combined: 7)	2.33(0.08,66.71)	1.46(0.06,33.73)	0.63(0.07,5.78)	0.21(0.01,5.13)	0.50(0.05,4.83)										

GENUS_56	Firmicutes;D_2__Clostridia;D_3__Clostridiales;D_4__Ruminococcaceae;D_5__Ruminococcaceae UCG-013 (Combined: 53)	1.51(0.62, 3.67)	1.89(0.82, 4.34)	1.25(0.70, 2.26)	0.65(0.28, 1.50)	0.97(0.53,1.78)	1.62(0.67,3.89)	2.29(0.98,5.35)	1.42(0.78,2.58)	0.57(0.25,1.33)	0.92(0.51,1.68)					
GENUS_57	Firmicutes;D_2__Clostridia;D_3__Clostridiales;D_4__Lachnospiraceae;D_5__Hungatella (Combined: 6)	1.10(0.19, 6.48)	1.19(0.23, 6.23)	1.08(0.33, 3.49)	1.87(0.35, 10.05)	2.06(0.62,6.87)	0.78(0.14,4.53)	0.66(0.12,3.57)	0.84(0.25,2.77)	2.66(0.49,14.33)	2.09(0.63,6.95)					
GENUS_58	Firmicutes;D_2__Clostridia;D_3__Clostridiales;D_4__Lachnospiraceae;D_5__Lachnospiraceae FCS020 group (Combined: 16)	0.74(0.27, 2.03)	0.56(0.22, 1.45)	0.76(0.39, 1.48)	1.40(0.54, 3.62)	1.04(0.53,2.06)	0.76(0.28,2.07)	0.60(0.23,1.57)	0.79(0.40,1.57)	1.36(0.52,3.57)	1.03(0.52,2.05)					
GENUS_59	Firmicutes;D_2__Negativicutes;D_3__Selenomonadales;D_4__Veillonellaceae;D_5__Veillonella (Combined: 18)	2.04(0.46, 9.03)	3.53(0.87, 14.24)	1.73(0.65, 4.64)	0.17(0.04, 0.69);p=0.01301	0.34(0.12,0.93);p=0.03596	1.47(0.35,6.18)	1.93(0.48,7.75)	1.32(0.49,3.51)	0.19(0.05,0.76);p=0.01841	0.28(0.10,0.75);p=0.01086				↓	↓
GENUS_60	Firmicutes;D_2__Clostridia;D_3__Clostridiales;D_4__Lachnospiraceae;D_5__GCA-900066575 (Combined: 21)	1.06(0.33, 3.44)	1.25(0.41, 3.76)	1.18(0.54, 2.57)	0.78(0.25, 2.37)	0.82(0.37,1.83)	0.94(0.29,3.03)	1.00(0.32,3.10)	1.07(0.48,2.37)	0.89(0.29,2.72)	0.83(0.37,1.86)					
GENUS_61	Firmicutes;D_2__Clostridia;D_3__Clostridiales;D_4__Ruminococcaceae;D_5__Ruminiclostridium 6 (Combined: 10)	1.97(0.29, 13.51)	3.96(0.65, 23.94)	2.00(0.56, 7.14)	0.43(0.07, 2.67)	0.85(0.23,3.13)	1.02(0.18,5.89)	1.50(0.28,8.11)	1.47(0.45,4.84)	1.08(0.20,5.79)	1.10(0.33,3.64)					
GENUS_62	Firmicutes;D_2__Clostridia;D_3__Clostridiales;D_4__Lachnospiraceae;D_5__CAG-56 (Combined: 16)	0.44(0.05, 3.55)	0.28(0.04, 2.00)	0.65(0.16, 2.57)	2.71(0.38, 19.64)	1.19(0.29,4.93)	0.34(0.04,2.67)	0.35(0.05,2.58)	1.04(0.25,4.23)	2.46(0.34,17.79)	0.84(0.20,3.43)					
GENUS_63	Firmicutes;D_2__Clostridia;D_3__Clostridiales;D_4__Ruminococcaceae;D_5__Negativibacillus (Combined: 8)	1.19(0.13, 10.94)	0.60(0.08, 4.84)	0.51(0.12, 2.22)	0.97(0.12, 8.01)	1.16(0.26,5.22)										
GENUS_65	Firmicutes;D_2__Clostridia;D_3__Clostridiales;D_4__Ruminococcaceae (Combined: 126)	0.80(0.25, 2.56)	0.50(0.17, 1.49)	0.62(0.29, 1.35)	1.29(0.43, 3.88)	1.03(0.47,2.27)	0.68(0.21,2.15)	0.52(0.17,1.59)	0.77(0.35,1.70)	1.40(0.46,4.23)	0.95(0.43,2.09)					
GENUS_66	Firmicutes;D_2__Clostridia;D_3__Clostridiales;D_4__Family XIII;D_5__Family XIII UCG-001 (Combined: 9)	2.96(0.55, 15.90)	3.33(0.69, 16.08)	1.12(0.37, 3.39)	0.47(0.10, 2.31)	1.39(0.45,4.31)	3.64(0.67,19.64)	3.57(0.70,18.21)	0.98(0.31,3.06)	0.41(0.08,2.06)	1.48(0.47,4.62)					
GENUS_67	Firmicutes;D_2__Clostridia;D_3__Clostridiales;D_4__Lachnospiraceae (Combined: 215)	1.34(0.47, 3.83)	0.61(0.23, 1.62)	0.45(0.23, 0.91);p=0.02611	2.15(0.79, 5.84)	2.88(1.41,5.88);p=0.0037	1.36(0.47,3.91)	0.64(0.23,1.77)	0.47(0.23,0.96);p=0.03895	2.07(0.75,5.70)	2.82(1.37,5.80);p=0.00488			↓		↑
GENUS_68	Proteobacteria;D_2__Deltaproteobacteria;D_3__Desulfovibrionales;D_4__Desulfovibrionaceae;D_5__Bilophila (Combined: 20)	0.90(0.23, 3.61)	1.08(0.29, 3.95)	1.19(0.48, 3.00)	1.43(0.38, 5.33)	1.29(0.50,3.31)	1.02(0.26,4.09)	1.46(0.38,5.56)	1.43(0.55,3.67)	1.28(0.34,4.84)	1.31(0.51,3.39)					

GENUS_69	Firmicutes;D_2__Clostridia;D_3__Clostridiales;D_4__Peptococcaceae;D_5__uncultured (Combined: 26)	0.52(0.06, 4.56)	1.46(0.19, 10.99)	2.79(0.66, 11.77)	0.50(0.06, 3.83)	0.26(0.06,1.14)	0.46(0.05,3.92)	1.48(0.19,11.53)	3.20(0.74,13.95)	0.49(0.06,3.80)	0.23(0.05,0.99);p=0.04837					↓
GENUS_70	Actinobacteria;D_2__Actinobacteria;D_3__Actinomycetales;D_4__Actinomycetaceae;D_5__Actinomyces (Combined: 17)	1.70(0.61, 4.76)	0.71(0.27, 1.87)	0.42(0.21, 0.83);p=0.01228	0.54(0.20, 1.42)	0.91(0.46,1.83)	2.04(0.74,5.59)	0.63(0.24,1.68)	0.31(0.16,0.62);p=0.00085	0.65(0.25,1.71)	1.32(0.66,2.62)			↓		
GENUS_71	Firmicutes;D_2__Clostridia;D_3__Clostridiales;D_4__Family XIII;D_5__[Eubacterium] brachy group (Combined: 10)	1.40(0.31, 6.28)	1.34(0.33, 5.47)	0.96(0.35, 2.59)	0.59(0.14, 2.43)	0.82(0.30,2.27)	1.36(0.30,6.12)	1.35(0.32,5.74)	0.99(0.36,2.75)	0.61(0.14,2.58)	0.83(0.30,2.31)					
GENUS_72	Firmicutes;D_2__Clostridia;D_3__Clostridiales;D_4__Lachnospiraceae;D_5__Lachnoclostridium (Combined: 69)	0.61(0.26, 1.40)	1.08(0.49, 2.35)	1.77(1.02, 3.07);p=0.04367	1.55(0.70, 3.42)	0.94(0.54,1.67)	0.77(0.35,1.72)	1.53(0.71,3.31)	1.98(1.15,3.41);p=0.01395	1.22(0.57,2.61)	0.94(0.55,1.63)			↑		
GENUS_73	Firmicutes;D_2__Clostridia;D_3__Clostridiales;D_4__Lachnospiraceae;D_5__[Eubacterium] eligens group (Combined: 28)	3.80(0.58, 24.76)	2.44(0.42, 14.10)	0.64(0.19, 2.22)	0.05(0.01, 0.28);p=0.00082	0.18(0.05,0.65);p=0.00892	3.56(0.55,23.19)	2.90(0.47,17.72)	0.81(0.23,2.93)	0.04(0.01,0.27);p=0.00073	0.16(0.04,0.58);p=0.0051				↓	↓
GENUS_74	Firmicutes;D_2__Bacilli;D_3__Lactobacillales;D_4__Carnobacteriaceae;D_5__Granulicatella (Combined: 3)	1.19(0.32, 4.47)	0.50(0.14, 1.72)	0.42(0.17, 1.01)	1.17(0.33, 4.09)	1.40(0.57,3.43)	1.17(0.31,4.37)	0.47(0.13,1.68)	0.40(0.16,0.99);p=0.0479	1.23(0.34,4.37)	1.43(0.58,3.54)			↓		
GENUS_75	Firmicutes;D_2__Clostridia;D_3__Clostridiales;D_4__Ruminococcaceae;D_5__Ruminococcaceae UCG-010 (Combined: 65)	3.92(0.52, 29.65)	7.94(1.19, 52.87);p=0.03211	2.03(0.54, 7.66)	0.13(0.02, 0.89);p=0.03742	0.51(0.13,2.00)	4.28(0.56,32.59)	8.80(1.24,62.52);p=0.02966	2.06(0.52,8.12)	0.12(0.02,0.85);p=0.03377	0.52(0.13,2.05)		↑		↓	
GENUS_76	Firmicutes;D_2__Bacilli;D_3__Lactobacillales;D_4__Streptococcaceae;D_5__Lactococcus (Combined: 9)	2.03(0.37, 11.23)	0.54(0.11, 2.70)	0.27(0.09, 0.83);p=0.02263	2.49(0.49, 12.61)	5.07(1.59,16.18);p=0.00619	2.15(0.40,11.57)	0.93(0.18,4.71)	0.43(0.14,1.36)	1.88(0.37,9.46)	4.03(1.27,12.77);p=0.01788					↑
GENUS_77	Firmicutes;D_2__Clostridia;D_3__Clostridiales;D_4__Clostridiales vadinBB60 group;D_5__uncultured organism (Combined: 4)	0.39(0.01, 10.43)	5.53(0.26, 118.47)	14.18(1.60,125.81);p=0.01727	0.45(0.02, 10.12)	0.18(0.02,1.66)	0.25(0.01,6.70)	3.03(0.13,70.26)	11.98(1.27,113.34);p=0.03036	0.38(0.02,8.66)	0.10(0.01,0.92);p=0.04183			↑		↓
GENUS_78	Firmicutes;D_2__Clostridia;D_3__Clostridiales;D_4__Lachnospiraceae;D_5__Lachnospiraceae NK4A136 group (Combined: 44)	0.92(0.32, 2.61)	1.55(0.58, 4.14)	1.70(0.85, 3.40)	0.69(0.26, 1.86)	0.63(0.31,1.29)	0.80(0.28,2.28)	1.36(0.50,3.72)	1.70(0.83,3.46)	0.81(0.30,2.22)	0.65(0.32,1.33)					
GENUS_79	Firmicutes;D_2__Erysipelotrichia;D_3__Erysipelotrichales;D_4__Erysipelotrichaceae;D_5__Erysipelotrichaceae UCG-003 (Combined: 11)	3.23(0.83, 12.63)	3.13(0.87, 11.22)	0.97(0.39, 2.39)	0.24(0.07, 0.87);p=0.02932	0.77(0.31,1.94)	3.56(0.92,13.77)	4.12(1.12,15.21);p=0.0333	1.16(0.46,2.91)	0.20(0.05,0.73);p=0.01448	0.71(0.28,1.78)		↑		↓	
GENUS_81	Firmicutes;D_2__Clostridia;D_3__Clostridiales;D_4__Peptostreptococcaceae;D_5__Peptostreptococcus (Combined: 2)	0.16(0.01, 2.95)	0.14(0.01, 2.13)	0.88(0.12, 6.52)	6.93(0.44, 108.58)	1.11(0.14,8.66)										

GENUS_82	Firmicutes;D_2__Clostridia;D_3__Clostridiales;D_4__Defluviitaleaceae;D_5__Defluviitaleaceae UCG-011 (Combined: 12)	1.41(0.33, 6.03)	1.36(0.35, 5.32)	0.97(0.37, 2.53)	0.80(0.20, 3.18)	1.12(0.42,3.02)	1.12(0.27,4.69)	1.26(0.32,5.03)	1.13(0.42,3.00)	0.83(0.21,3.27)	0.92(0.35,2.46)					
GENUS_83	Firmicutes;D_2__Clostridia;D_3__Clostridiales;D_4__Clostridiales vadinBB60 group;D_5__uncultured Thermoanaerobacterales bacterium (Combined: 2)	1.92(0.02, 204.89)	1.20(0.02, 95.27)	0.62(0.03, 13.78)	0.21(0.00, 17.44)	0.40(0.02,9.48)										
GENUS_84	Firmicutes;D_2__Erysipelotrichia;D_3__Erysipelotrichales;D_4__Erysipelotrichaceae;D_5__Holdemania (Combined: 12)	0.77(0.29, 2.08)	0.71(0.28, 1.81)	0.92(0.48, 1.79)	1.82(0.71, 4.67)	1.41(0.71,2.78)	0.75(0.28,2.03)	0.64(0.24,1.66)	0.85(0.43,1.68)	1.93(0.74,5.00)	1.45(0.73,2.88)					
GENUS_85	Firmicutes;D_2__Clostridia;D_3__Clostridiales;D_4__Lachnospiraceae;D_5__Sellimonas (Combined: 13)	0.52(0.08, 3.44)	0.81(0.14, 4.76)	1.56(0.45, 5.46)	4.51(0.75, 27.04)	2.34(0.65,8.47)										
GENUS_86	Firmicutes;D_2__Clostridia;D_3__Clostridiales;D_4__Christensenellaceae;D_5__uncultured (Combined: 32)	0.38(0.10, 1.51)	0.63(0.17, 2.31)	1.68(0.67, 4.20)	1.34(0.36, 4.94)	0.51(0.20,1.30)	0.30(0.08,1.16)	0.44(0.12,1.63)	1.47(0.58,3.73)	1.76(0.48,6.47)	0.53(0.21,1.34)					
GENUS_88	Firmicutes;D_2__Clostridia;D_3__Clostridiales;D_4__Ruminococcaceae;D_5__DTU089 (Combined: 4)	1.24(0.37, 4.14)	1.63(0.53, 5.03)	1.31(0.59, 2.91)	1.23(0.39, 3.84)	1.52(0.67,3.45)										
GENUS_89	Actinobacteria;D_2__Coriobacteriia;D_3__Coriobacteriales;D_4__Coriobacteriales Incertae Sedis;D_5__uncultured (Combined: 11)	3.03(0.28, 32.82)	1.87(0.20, 17.37)	0.62(0.13, 2.98)	0.72(0.08, 6.90)	2.18(0.43,11.00)										
GENUS_90	Firmicutes;D_2__Clostridia;D_3__Clostridiales;D_4__Ruminococcaceae;D_5__Anaerotruncus (Combined: 8)	0.67(0.20, 2.25)	1.13(0.36, 3.49)	1.69(0.75, 3.82)	1.23(0.39, 3.87)	0.82(0.35,1.89)	0.67(0.20,2.26)	1.08(0.34,3.47)	1.62(0.70,3.73)	1.19(0.37,3.79)	0.80(0.34,1.85)					
GENUS_91	Actinobacteria;D_2__Coriobacteriia;D_3__Coriobacteriales;D_4__Eggerthellaceae (Combined: 8)	0.58(0.11, 2.98)	1.47(0.32, 6.79)	2.54(0.86, 7.56)	1.21(0.26, 5.69)	0.70(0.23,2.14)										
GENUS_92	Firmicutes;D_2__Clostridia;D_3__Clostridiales;D_4__Lachnospiraceae;D_5__[Eubacterium] ventriosum group (Combined: 30)	1.60(0.49, 5.23)	1.19(0.39, 3.62)	0.75(0.34, 1.63)	0.56(0.18, 1.73)	0.90(0.40,2.02)	1.29(0.40,4.20)	1.00(0.32,3.13)	0.78(0.35,1.74)	0.62(0.20,1.93)	0.80(0.36,1.80)					
GENUS_93	Proteobacteria;D_2__Gammaproteobacteria;D_3__Pseudomonadales;D_4__Pseudomonadaceae;D_5__Pseudomonas (Combined: 9)	29.10(3.35,252.47);p=0.00223	1.91(0.24, 15.17)	0.07(0.02, 0.25);p=0.00007	0.15(0.02, 1.22)	4.44(1.16,17.08);p=0.02995	76.13(8.38,691.28);p=0.00012;q=0.01566	3.06(0.34,27.29)	0.04(0.01,0.15);p=0;q=0.00257	0.12(0.01,1.02)	8.95(2.41,33.17);p=0.00105	↑*		↓*		↑
GENUS_94	Firmicutes;D_2__Clostridia;D_3__Clostridiales;D_4__Ruminococcaceae;D_5__Fournierella (Combined: 13)	0.37(0.05, 3.02)	1.38(0.20, 9.72)	3.71(0.92, 14.90)	1.01(0.14, 7.31)	0.38(0.09,1.57)										

GENUS_95	Firmicutes;D_2__Clostridia;D_3__Clostridiales;D_4__Ruminococcaceae;D_5__Angelakisella (Combined: 6)	0.50(0.11, 2.26)	0.57(0.14, 2.32)	1.13(0.42, 3.10)	1.39(0.34, 5.75)	0.70(0.25,1.95)										
GENUS_96	Firmicutes;D_2__Erysipelotrichia;D_3__Erysipelotrichales;D_4__Erysipelotrichaceae;D_5__Erysipelatoclostridium (Combined: 9)	1.49(0.51, 4.38)	1.00(0.36, 2.76)	0.67(0.33, 1.38)	1.58(0.57, 4.40)	2.36(1.13,4.91);p=0.02198	1.46(0.50,4.31)	1.02(0.36,2.90)	0.70(0.33,1.46)	1.63(0.58,4.60)	2.38(1.14,5.00);p=0.02137					↑
GENUS_97	Actinobacteria;D_2__Coriobacteriia;D_3__Coriobacteriales;D_4__Eggerthellaceae;D_5__Gordonibacter (Combined: 10)	1.07(0.39, 2.93)	0.81(0.32, 2.08)	0.76(0.39, 1.47)	1.00(0.39, 2.60)	1.08(0.54,2.13)	1.01(0.37,2.74)	0.70(0.27,1.84)	0.70(0.35,1.37)	1.10(0.43,2.87)	1.12(0.57,2.21)					
GENUS_98	Firmicutes;D_2__Clostridia;D_3__Clostridiales;D_4__Lachnospiraceae;D_5__Lachnospiraceae UCG-010 (Combined: 18)	0.27(0.05, 1.51)	0.80(0.16, 3.94)	2.93(0.94, 9.11)	0.99(0.20, 4.97)	0.27(0.09,0.87);p=0.02794	0.34(0.06,1.86)	1.27(0.25,6.52)	3.74(1.17,11.97);p=0.0261	0.91(0.18,4.63)	0.31(0.10,0.99);p=0.04856			↑		↓
GENUS_99	Firmicutes;D_2__Clostridia;D_3__Clostridiales;D_4__Lachnospiraceae;D_5__[Eubacterium] xylanophilum group (Combined: 20)	0.89(0.11, 7.30)	0.93(0.13, 6.61)	1.03(0.26, 4.16)	0.64(0.09, 4.64)	0.57(0.14,2.36)	0.88(0.11,7.13)	0.73(0.10,5.53)	0.83(0.20,3.49)	0.71(0.09,5.28)	0.62(0.15,2.60)					
GENUS_100	Firmicutes;D_2__Clostridia;D_3__Clostridiales;D_4__Lachnospiraceae;D_5__GCA-900066755 (Combined: 2)	0.72(0.11, 4.77)	1.43(0.25, 8.24)	1.98(0.56, 6.95)	1.51(0.25, 8.97)	1.09(0.30,4.02)	0.49(0.08,3.12)	0.85(0.14,5.02)	1.75(0.49,6.34)	2.11(0.36,12.43)	1.02(0.28,3.78)					
GENUS_101	Actinobacteria;D_2__Coriobacteriia;D_3__Coriobacteriales;D_4__Eggerthellaceae;D_5__Adlercreutzia (Combined: 2)	0.85(0.22, 3.30)	0.85(0.24, 3.01)	0.99(0.40, 2.44)	1.39(0.38, 5.03)	1.18(0.47,2.97)										
GENUS_102	Firmicutes;D_2__Clostridia;D_3__DTU014;D_4__uncultured bacterium;D_5__ (Combined: 2)	0.96(0.07, 12.98)	1.91(0.17, 21.46)	1.98(0.36, 11.06)	0.20(0.02, 2.31)	0.19(0.03,1.11)										
GENUS_103	Firmicutes;D_2__Clostridia;D_3__Clostridiales;D_4__Ruminococcaceae;D_5__Ruminococcaceae UCG-003 (Combined: 12)	1.94(0.43, 8.87)	3.88(0.94, 16.06)	1.99(0.73, 5.44)	0.15(0.04, 0.65);p=0.01094	0.30(0.11,0.84);p=0.02195	2.31(0.51,10.39)	5.60(1.31,23.96);p=0.02014	2.43(0.87,6.77)	0.12(0.03,0.49);p=0.00343	0.27(0.10,0.75);p=0.01175		↑		↓	↓
GENUS_104	Firmicutes;D_2__Clostridia;D_3__Clostridiales;D_4__Ruminococcaceae;D_5__Hydrogenoanaerobacterium (Combined: 5)	1.82(0.11, 30.17)	2.89(0.21, 39.80)	1.59(0.27, 9.19)	0.19(0.01, 2.62)	0.34(0.06,2.02)	1.95(0.11,33.01)	2.92(0.19,44.37)	1.50(0.25,9.14)	0.18(0.01,2.68)	0.35(0.06,2.12)					
GENUS_105	Firmicutes;D_2__Clostridia;D_3__Clostridiales;D_4__Christensenellaceae;D_5__Catabacter (Combined: 6)	1.18(0.16, 8.69)	1.15(0.18, 7.48)	0.98(0.26, 3.67)	0.50(0.08, 3.27)	0.59(0.15,2.25)	1.55(0.23,10.44)	1.43(0.23,9.07)	0.93(0.26,3.35)	0.59(0.09,3.70)	0.92(0.25,3.32)					
GENUS_106	Firmicutes;D_2__Clostridia;D_3__Clostridiales;D_4__Ruminococcaceae;D_5__Ruminococcaceae UCG-007 (Combined: 2)	0.50(0.03, 9.08)	0.16(0.01, 2.52)	0.32(0.04, 2.48)	1.13(0.07, 17.33)	0.56(0.08,4.15)	0.39(0.02,6.68)	0.07(0.00,1.18)	0.18(0.02,1.50)	1.32(0.09,19.59)	0.51(0.07,3.71)					

GENUS_108	Proteobacteria;D_2__Deltaproteobacteria;D_3__Desulfovibrionales;D_4__Desulfovibrionaceae;D_5__Desulfovibrio (Combined: 11)	30.17(1.20,757.65);p=0.03832	8.80(0.43,180.30)	0.29(0.03,2.45)	0.07(0.00,1.47)	2.08(0.24,18.41)	32.27(1.39,751.46);p=0.03053	10.64(0.51,223.19)	0.33(0.04,2.79)	0.03(0.00,0.62);p=0.0232	0.97(0.11,8.26)	↑			↓	
GENUS_109	Firmicutes;D_2__Erysipelotrichia;D_3__Erysipelotrichales;D_4__Erysipelotrichaceae;D_5__Dielma (Combined: 5)	1.58(0.25,10.05)	2.91(0.51,16.47)	1.85(0.54,6.26)	2.32(0.39,13.59)	3.65(1.02,13.01);p=0.04603	1.98(0.32,12.37)	4.81(0.82,28.23)	2.43(0.70,8.40)	0.92(0.16,5.36)	1.81(0.52,6.37)					
GENUS_110	Firmicutes;D_2__Clostridia;D_3__Clostridiales;D_4__Lachnospiraceae;D_5__Lachnospira (Combined: 35)	1.57(0.43,5.72)	1.63(0.49,5.48)	1.04(0.44,2.45)	0.41(0.12,1.38)	0.64(0.26,1.53)	1.82(0.50,6.64)	2.16(0.62,7.54)	1.19(0.49,2.87)	0.33(0.09,1.13)	0.59(0.24,1.44)					
GENUS_111	Firmicutes;D_2__Bacilli;D_3__Lactobacillales;D_4__Leuconostocaceae;D_5__Leuconostoc (Combined: 7)	2.06(0.09,48.97)	2.03(0.10,39.49)	0.99(0.12,8.03)	0.47(0.02,9.47)	0.97(0.11,8.27)	0.71(0.03,14.65)	1.95(0.10,36.16)	2.75(0.34,22.26)	0.49(0.03,9.00)	0.35(0.04,2.82)					
GENUS_112	Firmicutes;D_2__Bacilli;D_3__Bacillales;D_4__Staphylococcaceae;D_5__Staphylococcus (Combined: 4)	1.30(0.11,14.91)	1.08(0.11,10.63)	0.83(0.16,4.18)	0.62(0.06,6.25)	0.81(0.16,4.19)	1.36(0.12,15.47)	0.95(0.09,10.00)	0.70(0.13,3.66)	0.70(0.07,7.23)	0.96(0.18,5.00)					
GENUS_113	Firmicutes;D_2__Clostridia;D_3__Clostridiales;D_4__Ruminococcaceae;D_5__Candidatus Soleaferrea (Combined: 20)	0.44(0.18,1.12)	0.83(0.35,1.97)	1.88(1.01,3.50);p=0.04744	1.66(0.69,3.96)	0.74(0.39,1.40)	0.43(0.17,1.08)	0.70(0.29,1.70)	1.65(0.87,3.12)	1.81(0.75,4.36)	0.77(0.41,1.47)					
GENUS_114	Firmicutes;D_2__Clostridia;D_3__Clostridiales;D_4__Family XIII;D_5__Mogibacterium (Combined: 5)	93.15(4.01,2161.13);p=0.00471	3.25(0.16,64.10)	0.03(0.00,0.25);p=0.00094	0.19(0.01,3.88)	17.84(2.35,135.50);p=0.00534	93.37(3.96,2202.42);p=0.00491	4.58(0.21,100.36)	0.05(0.01,0.38);p=0.00367	0.17(0.01,3.66)	15.90(2.08,121.69);p=0.00771	↑		↓		↑
GENUS_115	Firmicutes;D_2__Clostridia;D_3__Clostridiales;D_4__Clostridiales vadinBB60 group;D_5__uncultured bacterium (Combined: 38)	1.41(0.09,22.85)	0.98(0.07,13.24)	0.69(0.11,4.37)	0.12(0.01,1.67)	0.17(0.03,1.11)										
GENUS_116	Patescibacteria;D_2__Saccharimonadia;D_3__Saccharimonadales;D_4__Saccharimonadaceae (Combined: 1)	1.49(0.19,11.39)	0.34(0.05,2.38)	0.23(0.06,0.92);p=0.03759	0.82(0.12,5.65)	1.21(0.31,4.82)	1.17(0.16,8.58)	0.25(0.04,1.77)	0.21(0.05,0.87);p=0.03062	1.10(0.16,7.49)	1.29(0.33,5.05)			↓		
GENUS_117	Actinobacteria;D_2__Coriobacteriia;D_3__Coriobacteriales;D_4__Eggerthellaceae;D_5__Enterorhabdus (Combined: 9)	0.71(0.05,10.16)	0.03(0.00,0.44);p=0.00951	0.05(0.01,0.31);p=0.00128	1.78(0.14,22.30)	1.26(0.21,7.74)	0.67(0.05,9.37)	0.03(0.00,0.39);p=0.00776	0.04(0.01,0.29);p=0.00107	2.65(0.21,33.40)	1.78(0.29,10.88)		↓	↓		
GENUS_119	Proteobacteria;D_2__Gammaproteobacteria;D_3__Betaproteobacteriales;D_4__Burkholderiaceae;D_5__Parasutterella (Combined: 15)	0.91(0.12,7.21)	1.11(0.16,7.70)	1.22(0.31,4.80)	1.63(0.23,11.55)	1.48(0.36,6.03)	1.13(0.14,8.86)	1.80(0.25,13.18)	1.60(0.39,6.53)	1.04(0.14,7.54)	1.17(0.29,4.81)					
GENUS_120	Firmicutes;D_2__Clostridia;D_3__Clostridiales;D_4__Lachnospiraceae;D_5__Lachnospiraceae ND3007 group (Combined: 16)	0.53(0.12,2.25)	1.32(0.34,5.11)	2.50(0.96,6.50)	0.89(0.23,3.51)	0.47(0.18,1.26)	0.54(0.13,2.27)	1.48(0.37,5.94)	2.75(1.03,7.37);p=0.04384	0.84(0.21,3.34)	0.45(0.17,1.20)			↑		

GENUS_121	Bacteroidetes;D_2__Bacteroidia;D_3__Bacteroidales;D_4__Barnesiellaceae;D_5__Coprobacter (Combined: 7)						0.25(0.00,17.19)	0.33(0.01,19.36)	1.30(0.07,23.59)	0.59(0.01,33.41)	0.15(0.01,2.68)					
GENUS_122	Firmicutes;D_2__Clostridia;D_3__Clostridiales;D_4__Ruminococcaceae;D_5__Butyricicoccus (Combined: 41)	0.73(0.26,1.99)	0.63(0.24,1.61)	0.87(0.44,1.69)	0.99(0.38,2.56)	0.72(0.36,1.42)	0.63(0.23,1.73)	0.54(0.20,1.42)	0.85(0.43,1.70)	1.09(0.41,2.86)	0.69(0.34,1.37)					
GENUS_124	Firmicutes;D_2__Clostridia;D_3__Clostridiales;D_4__Ruminococcaceae;D_5__Oscillibacter (Combined: 28)	0.32(0.11,0.95);p=0.04023	0.51(0.18,1.41)	1.59(0.77,3.29)	2.52(0.89,7.08)	0.80(0.38,1.69)	0.35(0.12,1.05)	0.62(0.22,1.76)	1.75(0.83,3.68)	2.19(0.77,6.22)	0.77(0.37,1.63)					
GENUS_126	Firmicutes;D_2__Clostridia;D_3__Clostridiales;D_4__Lachnospiraceae;D_5__Lachnospiraceae UCG-008 (Combined: 6)	0.54(0.12,2.40)	0.99(0.25,4.01)	1.84(0.68,4.97)	0.64(0.16,2.61)	0.34(0.12,0.95);p=0.03977	0.53(0.12,2.31)	1.37(0.33,5.66)	2.59(0.94,7.13)	0.52(0.13,2.14)	0.28(0.10,0.76);p=0.01307					↓
GENUS_127	Firmicutes;D_2__Clostridia;D_3__Clostridiales;D_4__Lachnospiraceae;D_5__Lachnospiraceae UCG-001 (Combined: 23)	5.01(0.51,49.08)	4.93(0.58,41.75)	0.98(0.22,4.45)	0.51(0.06,4.45)	2.56(0.55,12.03)	5.53(0.56,54.41)	5.58(0.61,50.75)	1.01(0.21,4.79)	0.49(0.05,4.43)	2.73(0.57,13.02)					
GENUS_128	Firmicutes;D_2__Clostridia;D_3__Clostridiales;D_4__Lachnospiraceae;D_5__Lachnospiraceae UCG-004 (Combined: 29)	0.51(0.12,2.13)	1.21(0.32,4.63)	2.39(0.92,6.17)	0.59(0.15,2.28)	0.30(0.11,0.79);p=0.01498	0.64(0.15,2.63)	1.88(0.48,7.39)	2.95(1.12,7.77);p=0.02876	0.41(0.11,1.61)	0.26(0.10,0.70);p=0.00713			↑		↓
GENUS_129	Firmicutes;D_2__Clostridia;D_3__Clostridiales;D_4__Clostridiales vadinBB60 group;D_5__gut metagenome (Combined: 14)	6.57(0.69,62.68)	37.05(4.46,308.01);p=0.00083	5.64(1.33,23.80);p=0.01863	0.04(0.00,0.36);p=0.00379	0.28(0.06,1.22)	10.14(1.04,99.04);p=0.04631	117.99(12.92,1077.71);p=0.00002;q=0.00729	11.63(2.70,50.07);p=0.00098	0.02(0.00,0.17);p=0.00043;q=0.04435	0.20(0.05,0.84);p=0.02871	↑	↑*	↑	↓*	↓
GENUS_131	Firmicutes;D_2__Clostridia;D_3__Clostridiales;D_4__Ruminococcaceae;D_5__Flavonifractor (Combined: 17)	0.79(0.31,2.00)	0.77(0.32,1.83)	0.97(0.53,1.79)	2.38(0.99,5.71)	1.88(1.00,3.53);p=0.04858	0.69(0.28,1.70)	0.75(0.31,1.80)	1.10(0.59,2.03)	2.23(0.93,5.30)	1.53(0.82,2.85)					
GENUS_134	Firmicutes;D_2__Clostridia;D_3__Clostridiales;D_4__Lachnospiraceae;D_5__Moryella (Combined: 3)	4.09(0.73,22.96)	3.45(0.68,17.49)	0.85(0.28,2.59)	0.21(0.04,1.09)	0.87(0.28,2.73)	4.54(0.84,24.40)	4.67(0.91,23.82)	1.03(0.34,3.16)	0.21(0.04,1.04)	0.93(0.30,2.87)					
GENUS_135	Firmicutes;D_2__Clostridia;D_3__Clostridiales;D_4__Ruminococcaceae;D_5__Ruminococcaceae UCG-009 (Combined: 13)	1.10(0.10,11.83)	3.79(0.42,34.49)	3.43(0.72,16.31)	0.37(0.04,3.46)	0.41(0.08,2.02)	1.20(0.11,12.98)	3.69(0.38,36.33)	3.08(0.62,15.33)	0.37(0.04,3.56)	0.44(0.09,2.20)					
GENUS_136	Actinobacteria;D_2__Coriobacteriia;D_3__Coriobacteriales;D_4__Atopobiaceae;D_5__uncultured (Combined: 5)	5.00(0.16,151.62)	5.48(0.22,135.09)	1.10(0.12,10.00)	0.04(0.00,0.96);p=0.0475	0.19(0.02,1.81)	4.54(0.15,133.83)	5.42(0.21,142.94)	1.19(0.12,11.46)	0.04(0.00,0.96);p=0.04737	0.17(0.02,1.62)				↓	
GENUS_137	Proteobacteria;D_2__Gammaproteobacteria;D_3__Pasteurellales;D_4__Pasteurellaceae;D_5__Haemophilus (Combined: 3)	0.78(0.15,4.09)	0.78(0.17,3.68)	1.00(0.33,2.98)	1.46(0.31,6.97)	1.14(0.37,3.51)	0.78(0.15,4.04)	1.04(0.21,5.09)	1.33(0.43,4.08)	1.11(0.23,5.34)	0.87(0.28,2.66)					

GENUS_139	Firmicutes;D_2__Clostridia;D_3__Clostridiales;D_4__Ruminococcaceae;D_5__Oscillospira (Combined: 12)	0.62(0.12,3.14)	1.05(0.23,4.73)	1.68(0.57,4.91)	1.15(0.25,5.28)	0.72(0.24,2.16)	0.71(0.14,3.55)	1.33(0.28,6.24)	1.86(0.62,5.60)	0.95(0.20,4.39)	0.68(0.22,2.04)					
GENUS_140	Firmicutes;D_2__Clostridia;D_3__Clostridiales;D_4__Clostridiaceae 1;D_5__Clostridium sensu stricto 1 (Combined: 22)	3.11(0.83,11.69)	1.49(0.43,5.15)	0.48(0.20,1.15)	0.30(0.09,1.05)	0.93(0.38,2.28)	4.11(1.12,15.12);p=0.03368	2.15(0.61,7.58)	0.52(0.22,1.28)	0.27(0.08,0.93);p=0.03777	1.09(0.45,2.66)	↑			↓	
GENUS_141	Firmicutes;D_2__Clostridia;D_3__Clostridiales;D_4__Ruminococcaceae;D_5__Phocea (Combined: 3)	0.74(0.17,3.20)	0.87(0.22,3.42)	1.18(0.44,3.17)	1.19(0.30,4.77)	0.88(0.32,2.41)	0.76(0.18,3.33)	1.00(0.24,4.10)	1.31(0.47,3.60)	1.18(0.29,4.82)	0.90(0.33,2.50)					
GENUS_142	Proteobacteria;D_2__Gammaproteobacteria;D_3__Enterobacteriales;D_4__Enterobacteriaceae;D_5__Escherichia-Shigella (Combined: 10)	0.24(0.04,1.47)	0.24(0.04,1.36)	1.04(0.31,3.50)	2.25(0.40,12.81)	0.53(0.15,1.84)	0.06(0.01,0.38);p=0.00253	0.13(0.02,0.75);p=0.02209	2.09(0.62,7.03)	6.22(1.13,34.34);p=0.0359	0.40(0.12,1.36)	↓	↓		↑	
GENUS_143	Firmicutes;D_2__Clostridia;D_3__Clostridiales (Combined: 29)	60.32(1.40,2605.10);p=0.03286	27.00(0.77,947.95)	0.45(0.04,4.63)	0.01(0.00,0.41);p=0.01469	0.69(0.06,7.50)	80.93(1.92,3408.88);p=0.02133	13.13(0.35,495.95)	0.16(0.01,1.78)	0.02(0.00,0.83);p=0.03915	1.81(0.17,19.85)	↑			↓	
GENUS_144	Actinobacteria;D_2__Coriobacteriia;D_3__Coriobacteriales;D_4__Eggerthellaceae;D_5__Eggerthella (Combined: 3)	1.08(0.36,3.22)	1.12(0.40,3.12)	1.04(0.50,2.15)	1.34(0.48,3.79)	1.45(0.69,3.04)	1.13(0.38,3.35)	1.23(0.43,3.51)	1.09(0.52,2.29)	1.12(0.39,3.20)	1.27(0.60,2.67)					
GENUS_145	Firmicutes;D_2__Clostridia;D_3__Clostridiales;D_4__Ruminococcaceae;D_5__Ruminiclostridium (Combined: 7)						1.08(0.17,7.04)	1.04(0.17,6.33)	0.96(0.26,3.49)	0.65(0.11,3.88)	0.70(0.19,2.51)					
GENUS_146	Firmicutes;D_2__Clostridia;D_3__Clostridiales;D_4__Lachnospiraceae;D_5__Anaerosporobacter (Combined: 3)	1200632531.21(0.00,Inf)	12158704076.20(0.00,Inf)	10.13(0.62,165.30)	0.00(0.00,Inf)	0.02(0.00,0.41);p=0.00997	14343709213417848.00(0.00,Inf)	641604538219601536.00(0.00,Inf)	44.73(2.09,956.98);p=0.01502	0.00(0.00,Inf)	0.00(0.00,0.04);p=0.00005;q=0.01056			↑		↓*
GENUS_147	Firmicutes;D_2__Clostridia;D_3__Clostridiales;D_4__Ruminococcaceae;D_5__GCA-900066225 (Combined: 20)	0.68(0.15,3.20)	0.56(0.13,2.39)	0.82(0.29,2.31)	0.83(0.19,3.59)	0.57(0.20,1.63)	0.72(0.15,3.37)	0.57(0.13,2.55)	0.80(0.27,2.31)	0.83(0.19,3.65)	0.60(0.21,1.72)					
GENUS_148	Firmicutes;D_2__Clostridia;D_3__Clostridiales;D_4__Ruminococcaceae;D_5__Pseudoflavonifractor (Combined: 3)	1.06(0.06,19.68)	1.46(0.10,22.26)	1.37(0.20,9.49)	2.01(0.12,32.84)	2.13(0.28,16.25)	1.02(0.06,18.58)	1.58(0.10,25.90)	1.56(0.21,11.36)	2.07(0.12,34.75)	2.10(0.27,16.31)					
GENUS_149	Firmicutes;D_2__Clostridia;D_3__Clostridiales;D_4__Lachnospiraceae;D_5__Tyzzerella (Combined: 7)	6.06(0.34,107.71)	6.55(0.44,97.20)	1.08(0.16,7.19)	2.11(0.14,32.83)	12.80(1.80,90.88);p=0.0108	4.88(0.28,85.19)	4.36(0.27,69.04)	0.89(0.13,6.22)	2.60(0.16,41.12)	12.69(1.78,90.65);p=0.01132					↑
GENUS_152	Proteobacteria;D_2__Gammaproteobacteria;D_3__Betaproteobacteriales;D_4__Burkholderiaceae;D_5__Noviherbaspirillum (Combined: 4)	1.31(0.03,61.46)	0.72(0.02,27.30)	0.55(0.04,7.26)	0.30(0.01,11.35)	0.39(0.03,5.15)										

GENUS_153	Proteobacteria;D_2__Gammaproteobacteria;D_3__Betaproteobacteriales;D_4__Burkholderiaceae;D_5__Sutterella (Combined: 37)	0.69(0.07,7.39)	2.53(0.28,23.18)	3.65(0.76,17.51)	0.23(0.02,2.12)	0.16(0.03,0.78);p=0.02347	0.65(0.06,6.94)	2.53(0.26,25.01)	3.91(0.77,19.76)	0.23(0.02,2.27)	0.15(0.03,0.76);p=0.02216					↓
GENUS_154	Firmicutes;D_2__Clostridia;D_3__Clostridiales;D_4__Peptostreptococcaceae;D_5__Intestinibacter (Combined: 5)	1.62(0.53,4.89)	0.78(0.28,2.20)	0.48(0.23,1.01)	0.87(0.30,2.48)	1.41(0.66,2.98)	1.53(0.51,4.56)	0.67(0.23,1.93)	0.44(0.21,0.92);p=0.03017	0.96(0.34,2.73)	1.47(0.69,3.09)			↓		
GENUS_155	Cyanobacteria;D_2__Oxyphotobacteria;D_3__Chloroplast (Combined: 12)	0.06(0.00,1.27)	2.20(0.14,35.53)	35.47(4.63,271.93);p=0.00059	2.99(0.18,50.28)	0.18(0.02,1.50)										
GENUS_156	Firmicutes;D_2__Clostridia;D_3__Clostridiales;D_4__Peptostreptococcaceae;D_5__Terrisporobacter (Combined: 2)	5.46(0.87,34.09)	2.21(0.40,12.27)	0.40(0.12,1.36)	0.11(0.02,0.64);p=0.01342	0.61(0.18,2.11)										
GENUS_157	Proteobacteria;D_2__Alphaproteobacteria;D_3__Caulobacterales;D_4__Caulobacteraceae;D_5__Brevundimonas (Combined: 1)	3245133348.26(0.00,Inf)	227410180.20(0.00,Inf)	0.07(0.01,0.81);p=0.03326	0.00(0.00,Inf)	2.99(0.34,26.18)	5672774314329615.00(0.00,Inf)	452872687431494.00(0.00,Inf)	0.08(0.01,0.85);p=0.03593	0.00(0.00,Inf)	5.30(0.61,46.28)			↓		
GENUS_159	Firmicutes;D_2__Bacilli;D_3__Lactobacillales;D_4__Lactobacillaceae;D_5__Pediococcus (Combined: 3)	10767741225.08(0.00,Inf)	1207390748.81(0.00,Inf)	0.11(0.01,2.07)	0.00(0.00,Inf)	0.64(0.03,12.26)	99378500058806304.00(0.00,Inf)	15019032761008232.00(0.00,Inf)	0.15(0.01,2.93)	0.00(0.00,Inf)	1.61(0.08,30.76)					
GENUS_160	Actinobacteria;D_2__Actinobacteria;D_3__Micrococcales;D_4__Micrococcaceae;D_5__Rothia (Combined: 9)	1.93(0.34,11.04)	1.23(0.24,6.32)	0.64(0.20,2.01)	0.64(0.12,3.36)	1.24(0.38,4.01)	1.86(0.32,10.66)	1.06(0.20,5.75)	0.57(0.17,1.87)	0.71(0.13,3.83)	1.33(0.40,4.35)					
GENUS_162	Proteobacteria;D_2__Gammaproteobacteria;D_3__Pasteurellales;D_4__Pasteurellaceae (Combined: 1)	4.56(0.09,233.14)	1.14(0.03,45.44)	0.25(0.02,3.38)	0.39(0.01,16.17)	1.77(0.12,25.46)	4.09(0.08,197.94)	1.34(0.03,56.78)	0.33(0.02,4.61)	0.25(0.01,10.19)	1.00(0.07,14.20)					
GENUS_163	Firmicutes;D_2__Clostridia;D_3__Clostridiales;D_4__Lachnospiraceae;D_5__[Ruminococcus] gnavus group (Combined: 9)	0.15(0.02,0.98);p=0.048	0.41(0.07,2.35)	2.69(0.78,9.27)	2.77(0.47,16.22)	0.42(0.12,1.50)	0.13(0.02,0.85);p=0.03264	0.43(0.07,2.60)	3.29(0.92,11.77)	2.92(0.49,17.50)	0.38(0.11,1.37)	↓				
GENUS_168	Firmicutes;D_2__Erysipelotrichia;D_3__Erysipelotrichales;D_4__Erysipelotrichaceae;D_5__Turicibacter (Combined: 20)	4.54(1.32,15.65);p=0.01669	1.50(0.47,4.80)	0.33(0.15,0.75);p=0.00834	0.24(0.07,0.78);p=0.01725	1.09(0.47,2.52)	5.50(1.60,18.86);p=0.00672	1.93(0.59,6.34)	0.35(0.15,0.81);p=0.01462	0.18(0.05,0.57);p=0.00396	0.97(0.42,2.24)	↑		↓	↓	
GENUS_169	Firmicutes;D_2__Clostridia;D_3__Clostridiales;D_4__Lachnospiraceae;D_5__Tyzzerella 3 (Combined: 5)	1.21(0.02,61.28)	0.52(0.01,20.36)	0.43(0.03,5.75)	2.55(0.06,105.33)	3.09(0.21,44.37)										
GENUS_170	Firmicutes;D_2__Clostridia;D_3__Clostridiales;D_4__Lachnospiraceae;D_5__[Eubacterium] ruminantium group (Combined: 7)	10.07(0.22,456.88)	11.88(0.33,422.97)	1.18(0.09,14.72)	0.09(0.00,3.39)	0.92(0.07,12.18)										

GENUS_174	Firmicutes;D_2__Erysipelotrichia;D_3__Erysipelotrichales;D_4__Erysipelotrichaceae;D_5__Merdibacter (Combined: 3)	0.22(0.02, 2.79)	1.24(0.12, 13.35)	5.75(1.04, 31.90);p=0.04529	1.87(0.17, 20.77)	0.40(0.07,2.34)										
GENUS_175	Firmicutes;D_2__Clostridia;D_3__Clostridiales;D_4__Ruminococcaceae;D_5__Anaerofilum (Combined: 8)	1.33(0.12, 14.41)	0.91(0.10, 8.46)	0.68(0.14, 3.30)	0.35(0.04, 3.32)	0.47(0.09,2.31)	1.24(0.12,12.93)	0.65(0.07,6.34)	0.53(0.11,2.64)	0.49(0.05,4.68)	0.61(0.12,3.01)					
GENUS_176	Actinobacteria;D_2__Coriobacteriia;D_3__Coriobacteriales;D_4__Coriobacteriales Incertae Sedis;D_5__Raoultibacter (Combined: 2)	132798649498.60(0.00,Inf)	51744242664.77(0.00,Inf)	0.39(0.05, 3.24)	0.00(0.00, Inf)	1.11(0.13,9.45)	16098510720766396.00(0.00,Inf)	5739290196032651.00(0.00,Inf)	0.36(0.04,3.12)	0.00(0.00,Inf)	0.52(0.06,4.25)					
GENUS_177	Firmicutes;D_2__Clostridia;D_3__Clostridiales;D_4__Eubacteriaceae;D_5__Eubacterium (Combined: 2)	0.57(0.02, 16.79)	0.08(0.00, 2.10)	0.15(0.01, 1.51)	6.14(0.24, 154.55)	3.52(0.34,36.74)	0.86(0.03,21.20)	0.04(0.00,0.95);p=0.04637	0.04(0.00,0.46);p=0.0093	11.19(0.48,261.91)	9.62(0.97,95.05)		↓	↓		
GENUS_182	Firmicutes;D_2__Clostridia;D_3__Clostridiales;D_4__Lachnospiraceae;D_5__[Eubacterium] fissicatena group (Combined: 8)	1.72(0.44, 6.71)	1.40(0.39, 5.03)	0.82(0.33, 2.00)	0.80(0.22, 2.90)	1.37(0.55,3.44)	1.66(0.43,6.45)	1.19(0.32,4.42)	0.72(0.29,1.80)	0.92(0.25,3.39)	1.52(0.60,3.84)					
GENUS_183	Firmicutes;D_2__Clostridia;D_3__Clostridiales;D_4__Lachnospiraceae;D_5__UC5-1-2E3 (Combined: 1)	0.28(0.02, 5.15)	0.87(0.06, 12.95)	3.07(0.44, 21.31)	2.96(0.19, 45.90)	0.84(0.11,6.16)										
GENUS_189	Firmicutes;D_2__Clostridia;D_3__Clostridiales;D_4__Peptostreptococcaceae;D_5__Paeniclostridium (Combined: 2)	496273611192.90(0.00,Inf)	39565411362.18(0.00,Inf)	0.08(0.01, 0.69);p=0.02198	0.00(0.00, Inf)	5.76(0.65,51.13)	105551341084980656.00(0.00,Inf)	4608512028605506.00(0.00,Inf)	0.04(0.00,0.38);p=0.00472	0.00(0.00,Inf)	7.87(0.95,65.11)			↓		
GENUS_190	Firmicutes;D_2__Bacilli;D_3__Lactobacillales;D_4__Enterococcaceae;D_5__Enterococcus (Combined: 4)	4.05(0.06, 270.10)	0.36(0.01, 18.51)	0.09(0.01, 1.45)	0.47(0.01, 25.24)	1.91(0.11,33.02)										
GENUS_192	Actinobacteria;D_2__Actinobacteria;D_3__Bifidobacteriales;D_4__Bifidobacteriaceae;D_5__Alloscardovia (Combined: 1)	1.01(0.01, 125.55)	0.79(0.01, 72.97)	0.79(0.03, 19.89)	0.63(0.01, 59.83)	0.64(0.02,16.72)										
GENUS_193	Firmicutes;D_2__Erysipelotrichia;D_3__Erysipelotrichales;D_4__Erysipelotrichaceae;D_5__Faecalitalea (Combined: 5)	0.68(0.08, 5.61)	1.02(0.14, 7.37)	1.50(0.37, 6.08)	2.46(0.33, 18.17)	1.68(0.40,7.04)	0.69(0.08,5.71)	0.98(0.13,7.46)	1.41(0.33,5.93)	2.28(0.30,17.26)	1.59(0.37,6.72)					
GENUS_195	Bacteroidetes;D_2__Bacteroidia;D_3__Bacteroidales;D_4__Porphyromonadaceae;D_5__Porphyromonas (Combined: 17)	3.56(0.20, 62.76)	2.74(0.19, 40.51)	0.77(0.12, 5.04)	0.91(0.06, 14.04)	3.25(0.47,22.57)	0.70(0.05,9.57)	0.81(0.07,10.06)	1.16(0.19,6.98)	3.34(0.26,42.12)	2.34(0.37,14.76)					
GENUS_196	Actinobacteria;D_2__Actinobacteria;D_3__Corynebacteriales;D_4__Corynebacteriaceae;D_5__Corynebacterium 1 (Combined: 8)	0.45(0.03, 6.12)	0.06(0.01, 0.76);p=0.02934	0.14(0.02, 0.85);p=0.033	11.16(0.91,136.66)	5.03(0.80,31.46)	0.16(0.02,1.66)	0.04(0.00,0.43);p=0.00748	0.26(0.04,1.63)	12.55(1.32,119.32);p=0.02769	2.06(0.35,11.95)		↓		↑	

GENUS_197	Firmicutes;D_2__Clostridia;D_3__Clostridiales;D_4__Family XI;D_5__Ezakiella (Combined: 4)	0.14(0.01, 2.10)	1.24(0.11, 14.12)	8.94(1.34, 59.48);p= 0.02355	0.49(0.04, 5.74)	0.07(0.01,0.47);p=0.00623	0.07(0.00,0.96);p=0.04663	0.35(0.03,3.92)	5.14(0.77,34.20)	1.86(0.17,20.26)	0.13(0.02,0.86);p=0.03421	↓				↓
GENUS_199	Firmicutes;D_2__Clostridia;D_3__Clostridiales;D_4__Lachnospiraceae;D_5__uncultured organism (Combined: 1)						0.67(0.01,37.05)	5.49(0.12,243.23)	8.16(0.50,133.68)	0.75(0.02,33.05)	0.50(0.03,8.44)					
GENUS_200	Bacteroidetes;D_2__Bacteroidia;D_3__Bacteroidales;D_4__Prevotellaceae;D_5__Prevotella (Combined: 11)	0.03(0.00, 1.01)	1.52(0.08, 30.65)	43.60(4.15,457.61);p=0.00165	4.53(0.21, 96.16)	0.16(0.01,1.77)										
GENUS_201	Proteobacteria;D_2__Alphaproteobacteria;D_3__Rhizobiales;D_4__Rhizobiaceae;D_5__Allorhizobium-Neorhizobium-Pararhizobium-Rhizobium (Combined: 2)	2.84(0.15, 53.54)	0.17(0.01, 3.83)	0.06(0.01, 0.60);p=0.01675	1.75(0.10, 31.99)	4.98(0.66,37.79)	2.73(0.14,52.72)	0.12(0.00,3.43)	0.04(0.00,0.52);p=0.0131	1.67(0.08,33.53)	4.57(0.60,34.74)			↓		
GENUS_202	Proteobacteria;D_2__Gammaproteobacteria;D_3__Betaproteobacteriales;D_4__Burkholderiaceae (Combined: 7)	45947547698.18(0.00,Inf)	44476999717.64(0.00,Inf)	0.97(0.05, 19.95)	0.00(0.00, Inf)	0.31(0.02,6.38)										
GENUS_203	Firmicutes;D_2__Clostridia;D_3__Clostridiales;D_4__Family XI;D_5__Peptoniphilus (Combined: 8)	0.27(0.01, 6.31)	0.23(0.01, 4.42)	0.86(0.10, 7.07)	7.13(0.36, 143.22)	1.90(0.22,16.57)	0.38(0.02,8.91)	0.21(0.01,4.37)	0.55(0.06,4.78)	8.02(0.38,168.38)	3.02(0.34,26.80)					
GENUS_204	Firmicutes;D_2__Erysipelotrichia;D_3__Erysipelotrichales;D_4__Erysipelotrichaceae;D_5__Candidatus Stoquefichus (Combined: 3)	0.02(0.00, 0.61);p=0.02367	0.30(0.02, 5.96)	12.35(1.43,106.55);p=0.02225	28.24(1.37,582.02);p=0.03046	0.70(0.08,6.43)	0.03(0.00,0.61);p=0.02272	0.11(0.01,2.03)	3.83(0.45,32.20)	50.56(2.63,971.73);p=0.00929	1.40(0.16,12.22)	↓			↑	
GENUS_205	Firmicutes;D_2__Clostridia;D_3__Clostridiales;D_4__Ruminococcaceae;D_5__Ruminococcaceae UCG-008 (Combined: 7)	0.54(0.01, 25.62)	1.76(0.05, 64.84)	3.29(0.25, 43.02)	2.07(0.05, 80.82)	1.11(0.08,15.76)	0.83(0.03,27.39)	0.56(0.02,16.68)	0.67(0.06,7.84)	4.15(0.14,124.84)	3.45(0.28,41.89)					
GENUS_206	Actinobacteria;D_2__Coriobacteriia;D_3__Coriobacteriales;D_4__Atopobiaceae;D_5__Atopobium (Combined: 8)	1.13(0.05, 25.05)	0.32(0.02, 6.09)	0.28(0.03, 2.36)	0.77(0.04, 14.51)	0.87(0.11,7.14)										
GENUS_212	Firmicutes;D_2__Clostridia;D_3__Clostridiales;D_4__Lachnospiraceae;D_5__Shuttleworthia (Combined: 6)						2.70(0.13,55.96)	4.89(0.26,91.68)	1.81(0.23,13.98)	0.77(0.04,14.41)	2.08(0.26,16.44)					
GENUS_214	Firmicutes;D_2__Clostridia;D_3__Clostridiales;D_4__Eubacteriaceae;D_5__Anaerofustis (Combined: 6)	1.24(0.30, 5.05)	0.71(0.19, 2.65)	0.57(0.22, 1.46)	1.92(0.50, 7.36)	2.38(0.91,6.28)	0.95(0.24,3.76)	0.49(0.13,1.85)	0.51(0.20,1.33)	2.75(0.72,10.46)	2.60(0.99,6.84)					
GENUS_218	Firmicutes;D_2__Clostridia;D_3__Clostridiales;D_4__Peptostreptococcaceae (Combined: 6)	9.79(0.05, 1875.90)	3.80(0.03, 540.70)	0.39(0.01, 11.30)	0.16(0.00, 23.18)	1.52(0.05,47.48)										

GENUS_231	Firmicutes;D_2__Clostridia;D_3__Clostridiales;D_4__Family XI;D_5__Parvimonas (Combined: 6)	0.90(0.09,8.67)	0.32(0.04,2.71)	0.36(0.08,1.63)	1.34(0.16,11.48)	1.21(0.26,5.68)	0.96(0.10,9.27)	0.36(0.04,3.25)	0.38(0.08,1.80)	1.39(0.16,12.25)	1.33(0.28,6.33)					
GENUS_234	Firmicutes;D_2__Clostridia;D_3__Clostridiales;D_4__Family XI;D_5__Finegoldia (Combined: 1)						0.26(0.01,7.05)	0.07(0.00,1.77)	0.28(0.03,2.80)	9.19(0.38,224.20)	2.35(0.23,23.73)					
GENUS_236	Firmicutes;D_2__Clostridia;D_3__Clostridiales;D_4__Family XI;D_5__Anaerococcus (Combined: 6)	0.14(0.01,3.42)	0.32(0.02,6.36)	2.33(0.27,20.39)	3.03(0.15,62.25)	0.42(0.05,3.84)	0.25(0.01,6.05)	0.34(0.02,7.20)	1.35(0.15,12.14)	3.75(0.18,78.61)	0.94(0.10,8.60)					
GENUS_237	Actinobacteria;D_2__Actinobacteria;D_3__Corynebacteriales;D_4__Corynebacteriaceae;D_5__Corynebacterium (Combined: 1)	1.88(0.44,7.98)	1.86(0.48,7.24)	0.99(0.39,2.51)	0.45(0.12,1.79)	0.85(0.33,2.20)	1.63(0.40,6.61)	1.56(0.40,6.06)	0.96(0.38,2.43)	0.55(0.14,2.13)	0.90(0.35,2.29)					
GENUS_238	Firmicutes;D_2__Bacilli;D_3__Lactobacillales;D_4__Aerococcaceae;D_5__Abiotrophia (Combined: 1)						5903849737287102.00(0.00,Inf)	1511579655086675.00(0.00,Inf)	0.26(0.03,1.95)	0.00(0.00,Inf)	4.90(0.62,38.76)					
GENUS_244	Firmicutes;D_2__Clostridia;D_3__Clostridiales;D_4__Lachnospiraceae;D_5__Lactonifactor (Combined: 2)	27.41(0.69,1086.13)	2.87(0.09,97.00)	0.10(0.01,0.99);p=0.04923	0.44(0.01,15.66)	12.13(1.19,123.44);p=0.03506										
GENUS_245	Firmicutes;D_2__Clostridia;D_3__Clostridiales;D_4__Lachnospiraceae;D_5__Lachnospiraceae NC2004 group (Combined: 7)	35.88(0.58,2205.18)	8.20(0.15,437.40)	0.23(0.03,2.05)	0.05(0.00,2.46)	1.63(0.18,15.01)	44.51(0.68,2920.35)	9.76(0.16,608.45)	0.22(0.02,2.09)	0.04(0.00,2.50)	1.85(0.20,17.22)					
GENUS_248	Firmicutes;D_2__Erysipelotrichia;D_3__Erysipelotrichales;D_4__Erysipelotrichaceae;D_5__Coprobacillus (Combined: 1)	0.04(0.00,0.63);p=0.02113	0.25(0.02,2.84)	5.69(0.92,35.26)	1.20(0.10,13.77)	0.05(0.01,0.34);p=0.00194	0.05(0.00,0.70);p=0.02582	0.29(0.02,3.49)	5.85(0.91,37.78)	1.34(0.11,15.70)	0.07(0.01,0.43);p=0.00431	↓				↓
GENUS_252	Actinobacteria;D_2__Actinobacteria;D_3__Corynebacteriales;D_4__Corynebacteriaceae;D_5__Lawsonella (Combined: 1)	4538453567.37(0.00,Inf)	3233422940.45(0.00,Inf)	0.71(0.04,12.26)	0.00(0.00,Inf)	0.27(0.02,4.13)	1468967813729974.00(0.00,Inf)	1110464012871734.00(0.00,Inf)	0.76(0.04,15.03)	0.00(0.00,Inf)	0.20(0.01,3.31)					
GENUS_256	Firmicutes;D_2__Clostridia;D_3__Clostridiales;D_4__Ruminococcaceae;D_5__Ruminiclostridium 1 (Combined: 9)						0.38(0.01,13.60)	0.21(0.01,6.58)	0.54(0.04,6.62)	2.61(0.09,79.40)	1.00(0.08,12.03)					
GENUS_258	Firmicutes;D_2__Clostridia;D_3__Clostridiales;D_4__Ruminococcaceae;D_5__Papillibacter (Combined: 2)	0.58(0.07,4.94)	0.51(0.07,3.71)	0.87(0.20,3.79)	0.85(0.12,6.26)	0.50(0.11,2.18)	0.52(0.06,4.35)	0.50(0.06,3.81)	0.96(0.21,4.32)	1.01(0.14,7.51)	0.52(0.12,2.32)					
GENUS_259	Actinobacteria;D_2__Actinobacteria;D_3__Actinomycetales;D_4__Actinomycetaceae;D_5__Varibaculum (Combined: 2)	0.66(0.04,11.26)	2.81(0.21,37.38)	4.24(0.64,27.98)	0.23(0.02,3.15)	0.15(0.02,1.05)	0.73(0.04,12.65)	3.16(0.22,46.25)	4.31(0.62,29.88)	0.23(0.02,3.25)	0.17(0.02,1.15)					

GENUS_261	Proteobacteria;D_2__Gammaproteobacteria;D_3__Xanthomonadales;D_4__Xanthomonadaceae;D_5__Stenotrophomonas (Combined: 5)	5.86(0.13,266.49)	0.56(0.01,23.66)	0.10(0.01,1.21)	0.55(0.01,21.86)	3.22(0.28,37.41)										
GENUS_262	Firmicutes;D_2__Clostridia;D_3__Clostridiales;D_4__Ruminococcaceae;D_5__uncultured bacterium (Combined: 1)	0.00(0.00,Inf)	0.28(0.03,2.65)	111343142700.06(0.00,Inf)	2.30(0.25,21.53)	0.00(0.00,Inf)	0.00(0.00,Inf)	0.10(0.01,1.00)	1324269907901246.00(0.00,Inf)	4.38(0.48,39.69)	0.00(0.00,Inf)					
GENUS_263	Firmicutes;D_2__Clostridia;D_3__Clostridiales;D_4__Lachnospiraceae;D_5__Oribacterium (Combined: 8)	0.79(0.04,14.15)	1.10(0.08,15.37)	1.39(0.20,9.80)	0.63(0.04,8.87)	0.50(0.07,3.58)	1.33(0.06,28.50)	2.69(0.14,50.44)	2.02(0.24,16.84)	0.13(0.01,2.27)	0.17(0.02,1.31)					
GENUS_267	Actinobacteria;D_2__Actinobacteria;D_3__Actinomycetales;D_4__Actinomycetaceae;D_5__F0332 (Combined: 2)	0.52(0.03,8.59)	1.58(0.12,20.05)	3.04(0.45,20.50)	0.58(0.04,7.55)	0.30(0.04,2.11)	1.00(0.07,14.07)	1.54(0.12,19.36)	1.54(0.25,9.34)	0.53(0.04,6.55)	0.53(0.09,3.21)					
GENUS_271	Patescibacteria;D_2__Saccharimonadia;D_3__Saccharimonadales;D_4__Saccharimonadaceae;D_5__uncultured Candidatus Saccharibacteria bacterium (Combined: 1)	3.53(0.23,54.85)	0.64(0.04,9.45)	0.18(0.03,1.12)	1.22(0.08,18.10)	4.30(0.69,26.82)	3.79(0.24,59.23)	0.74(0.05,11.79)	0.19(0.03,1.27)	1.02(0.07,15.80)	3.88(0.61,24.56)					
GENUS_302	Proteobacteria;D_2__Gammaproteobacteria;D_3__Pseudomonadales;D_4__Moraxellaceae;D_5__Acinetobacter (Combined: 8)						2814715401364155.00(0.00,Inf)	781623598654501.00(0.00,Inf)	0.28(0.02,4.04)	0.00(0.00,Inf)	0.51(0.04,5.83)					
GENUS_309	Proteobacteria;D_2__Alphaproteobacteria;D_3__Azospirillales;D_4__Azospirillaceae;D_5__Azospirillum (Combined: 2)	1.21(0.00,Inf)	36049177741585.90(0.00,Inf)	29827223890356.40(0.00,Inf)	0.00(0.00,Inf)	0.00(0.00,Inf)										
GENUS_320	Proteobacteria;D_2__Gammaproteobacteria;D_3__Pasteurellales;D_4__Pasteurellaceae;D_5__Aggregatibacter (Combined: 2)	0.00(0.00,Inf)	0.07(0.00,7.71)	135757849222087.00(0.00,Inf)	30.78(0.22,4334.78)	0.00(0.00,Inf)										
GENUS_351	Firmicutes;D_2__Clostridia;D_3__Clostridiales;D_4__Peptostreptococcaceae;D_5__uncultured (Combined: 4)	2119863074744.59(0.00,Inf)	218131588559.42(0.00,Inf)	0.10(0.00,6.50)	0.00(0.00,Inf)	146.42(2.02,10613.82);p=0.02251										
GENUS_365	Firmicutes;D_2__Clostridia;D_3__Clostridiales;D_4__Christensenellaceae (Combined: 4)	0.28(0.01,8.26)	0.33(0.02,6.89)	1.16(0.09,14.40)	0.67(0.03,13.13)	0.19(0.02,2.18)										

Table 3.1: Species-level differences in the gut microbial communities: participants with MS, and monophasic demyelinating disease compared to controls for individual ASVs

		Rate ratios (95%CIs) unadjusted			Rate ratios (95%CIs) age and sex adjusted					
Taxa Identifier (for internal use)	**Species-level findings (shown as: Phylum;Class;Order;Family;Genus;Species)**	**MS cases (vs ADS)**	**MS cases (vs controls)**	**ADS (vs controls)**	**MS cases (vs ADS)**	**MS cases (vs controls)**	**ADS (vs controls)**	*MS cases (vs ADS)*	*MS cases (vs controls)*	*ADS (vs controls)*
ASV_3	Firmicutes;D_2__Bacilli;D_3__Lactobacillales;D_4__Streptococcaceae;D_5__Streptococcus;D_6__Streptococcus salivarius subsp. thermophilus;D_7__;D_8__;D_9__;D_10__;D_11__;D_12__;D_13__;D_14__	1.71(0.91,3.21)	1.30(0.68,2.49)	0.76(0.41,1.40)	1.82(0.94,3.51)	1.36(0.70,2.63)	0.75(0.40,1.39)			
ASV_4	Firmicutes;D_2__Clostridia;D_3__Clostridiales;D_4__Lachnospiraceae;D_5__Blautia	1.05(0.60,1.82)	0.99(0.56,1.75)	0.94(0.55,1.61)	0.98(0.55,1.75)	0.95(0.53,1.70)	0.97(0.57,1.67)			
ASV_5	Firmicutes;D_2__Clostridia;D_3__Clostridiales;D_4__Lachnospiraceae;D_5__Blautia	0.82(0.50,1.35)	0.88(0.52,1.47)	1.07(0.66,1.74)	0.86(0.51,1.45)	0.95(0.56,1.61)	1.11(0.68,1.81)			
ASV_7	Firmicutes;D_2__Clostridia;D_3__Clostridiales;D_4__Lachnospiraceae;D_5__Blautia	1.19(0.79,1.82)	1.01(0.65,1.55)	0.84(0.56,1.27)	1.27(0.82,1.96)	1.03(0.66,1.59)	0.81(0.54,1.22)			
ASV_8	Firmicutes;D_2__Clostridia;D_3__Clostridiales;D_4__Lachnospiraceae;D_5__Coprococcus 3	1.04(0.44,2.47)	0.94(0.39,2.29)	0.90(0.39,2.08)	1.00(0.41,2.45)	1.01(0.42,2.47)	1.01(0.44,2.32)			
ASV_9	Firmicutes;D_2__Clostridia;D_3__Clostridiales;D_4__Lachnospiraceae;D_5__Anaerostipes	0.87(0.50,1.49)	0.96(0.55,1.67)	1.11(0.65,1.87)	0.95(0.54,1.68)	1.03(0.58,1.80)	1.08(0.64,1.82)			
ASV_10	Bacteroidetes;D_2__Bacteroidia;D_3__Bacteroidales;D_4__Bacteroidaceae;D_5__Bacteroides	0.56(0.21,1.46)	1.65(0.61,4.45)	2.97(1.17,7.55); p=0.02251	0.44(0.16,1.19)	1.44(0.54,3.89)	3.27(1.30,8.26);p=0.01199			↑
ASV_11	Firmicutes;D_2__Clostridia;D_3__Clostridiales;D_4__Lachnospiraceae;D_5__Dorea	1.40(0.72,2.71)	1.45(0.73,2.88)	1.04(0.55,1.98)	1.29(0.64,2.58)	1.43(0.72,2.86)	1.11(0.58,2.12)			
ASV_13	Firmicutes;D_2__Bacilli;D_3__Lactobacillales;D_4__Streptococcaceae;D_5__Streptococcus	2.62(1.20,5.68);p=0.01519	0.82(0.37,1.83)	0.31(0.15,0.67); p=0.00255	2.92(1.30,6.59); p=0.00971	0.95(0.42,2.14)	0.33(0.15,0.70);p=0.00371	↑		↓
ASV_14	Firmicutes;D_2__Clostridia;D_3__Clostridiales;D_4__Lachnospiraceae;D_5__Blautia	0.80(0.44,1.44)	1.29(0.70,2.36)	1.60(0.91,2.84)	0.75(0.40,1.38)	1.25(0.68,2.32)	1.68(0.95,2.98)			
ASV_15	Firmicutes;D_2__Clostridia;D_3__Clostridiales;D_4__Lachnospiraceae;D_5__[Eubacterium] hallii group	1.66(0.80,3.42)	2.31(1.09,4.87);p=0.02815	1.39(0.69,2.81)	1.91(0.89,4.07)	2.52(1.18,5.38); p=0.01656	1.32(0.65,2.68)		↑	
ASV_18	Firmicutes;D_2__Clostridia;D_3__Clostridiales;D_4__Christensenellaceae;D_5__Christensenellaceae R-7 group;D_6__uncultured organism;D_7__;D_8__;D_9__;D_10__;D_11__;D_12__;D_13__;D_14__	1.46(0.15,13.77)	0.38(0.04,3.80)	0.26(0.03,2.27)						
ASV_24	Bacteroidetes;D_2__Bacteroidia;D_3__Bacteroidales;D_4__Bacteroidaceae;D_5__Bacteroides	0.49(0.13,1.89)	0.69(0.17,2.79)	1.42(0.38,5.24)	0.47(0.12,1.88)	0.57(0.14,2.28)	1.22(0.33,4.47)			
ASV_25	Firmicutes;D_2__Clostridia;D_3__Clostridiales;D_4__Lachnospiraceae;D_5__Blautia	0.71(0.18,2.69)	0.66(0.17,2.61)	0.93(0.26,3.41)	0.61(0.16,2.39)	0.35(0.09,1.35)	0.56(0.16,2.01)			
ASV_26	Firmicutes;D_2__Clostridia;D_3__Clostridiales;D_4__Ruminococcaceae;D_5__Ruminiclostridium 5	0.98(0.37,2.58)	0.74(0.27,2.02)	0.76(0.30,1.95)	1.05(0.38,2.88)	0.88(0.32,2.41)	0.84(0.33,2.14)			
ASV_27	Actinobacteria;D_2__Coriobacteriia;D_3__Coriobacteriales;D_4__Coriobacteriaceae;D_5__Collinsella	0.72(0.26,2.03)	0.53(0.19,1.54)	0.74(0.27,2.00)	0.70(0.24,2.03)	0.55(0.19,1.61)	0.79(0.29,2.15)			
ASV_28	Firmicutes;D_2__Clostridia;D_3__Clostridiales;D_4__Lachnospiraceae;D_5__uncultured;D_6__uncultured Eubacterium sp.;D_7__;D_8__;D_9__;D_10__;D_11__;D_12__;D_13__;D_14__	0.38(0.16,0.91);p=0.0305	0.69(0.28,1.73)	1.84(0.78,4.33)	0.27(0.11,0.66); p=0.00394	0.43(0.18,1.05)	1.61(0.70,3.69)	↓		
ASV_31	Firmicutes;D_2__Clostridia;D_3__Clostridiales;D_4__Ruminococcaceae;D_5__[Eubacterium] coprostanoligenes group;D_6__uncultured organism;D_7__;D_8__;D_9__;D_10__;D_11__;D_12__;D_13__;D_14__	0.92(0.07,11.52)	0.82(0.06,11.04)	0.89(0.08,10.27)						

ASV_32	Firmicutes;D_2__Clostridia;D_3__Clostridiales;D_4__Christensenellaceae;D_5__Christensenellaceae R-7 group	0.14(0.02,1.01)	0.28(0.04,2.08)	1.94(0.30,12.69)	0.12(0.02,0.92); p=0.04177	0.15(0.02,1.13)	1.23(0.19,8.06)	↓		
ASV_33	Bacteroidetes;D_2__Bacteroidia;D_3__Bacteroidales;D_4__Rikenellaceae;D_5__Alistipes;D_6__Alistipes indistinctus YIT 12060;D_7__;D_8__;D_9__;D_10__;D_11__;D_12__;D_13__;D_14__	0.86(0.12,6.16)	1.33(0.18,10.08)	1.54(0.23,10.33)						
ASV_34	Firmicutes;D_2__Negativicutes;D_3__Selenomonadales;D_4__Veillonellaceae;D_5__Dialister	1.59(0.42,6.04)	1.56(0.39,6.17)	0.98(0.27,3.58)	1.47(0.36,5.96)	1.43(0.35,5.80)	0.98(0.26,3.60)			
ASV_35	Firmicutes;D_2__Clostridia;D_3__Clostridiales;D_4__Christensenellaceae;D_5__Christensenellaceae R-7 group	0.47(0.04,5.88)	0.41(0.03,5.54)	0.87(0.08,10.12)	0.21(0.02,2.66)	0.12(0.01,1.57)	0.60(0.06,6.36)			
ASV_36	Bacteroidetes;D_2__Bacteroidia;D_3__Bacteroidales;D_4__Bacteroidaceae;D_5__Bacteroides;D_6__Bacteroides thetaiotaomicron;D_7__;D_8__;D_9__;D_10__;D_11__;D_12__;D_13__;D_14__	0.99(0.39,2.48)	0.70(0.27,1.79)	0.70(0.29,1.71)	0.91(0.35,2.38)	0.67(0.25,1.75)	0.73(0.30,1.80)			
ASV_37	Firmicutes;D_2__Clostridia;D_3__Clostridiales;D_4__Lachnospiraceae;D_5__[Ruminococcus] torques group;D_6__uncultured organism;D_7__;D_8__;D_9__;D_10__;D_11__;D_12__;D_13__;D_14__	1.41(0.54,3.64)	2.31(0.87,6.14)	1.64(0.65,4.11)	1.87(0.70,4.96)	1.95(0.73,5.17)	1.04(0.42,2.60)			
ASV_38	Firmicutes;D_2__Clostridia;D_3__Clostridiales;D_4__Ruminococcaceae;D_5__Ruminococcus 1;D_6__uncultured organism;D_7__;D_8__;D_9__;D_10__;D_11__;D_12__;D_13__;D_14__	0.62(0.21,1.84)	0.57(0.19,1.77)	0.93(0.32,2.69)	0.79(0.25,2.46)	0.69(0.22,2.14)	0.87(0.30,2.51)			
ASV_39	Bacteroidetes;D_2__Bacteroidia;D_3__Bacteroidales;D_4__Rikenellaceae;D_5__Alistipes;D_6__uncultured organism;D_7__;D_8__;D_9__;D_10__;D_11__;D_12__;D_13__;D_14__	0.23(0.07,0.76);p=0.01663	0.44(0.13,1.52)	1.91(0.59,6.15)	0.24(0.07,0.84); p=0.02616	0.45(0.13,1.58)	1.88(0.58,6.11)	↓		
ASV_40	Firmicutes;D_2__Clostridia;D_3__Clostridiales;D_4__Lachnospiraceae;D_5__[Eubacterium] hallii group	2.02(0.54,7.58)	1.14(0.29,4.47)	0.57(0.16,2.04)	1.54(0.39,6.12)	0.87(0.22,3.46)	0.57(0.16,2.05)			
ASV_41	Firmicutes;D_2__Clostridia;D_3__Clostridiales;D_4__Lachnospiraceae;D_5__Marvinbryantia	0.21(0.03,1.51)	0.38(0.05,2.96)	1.85(0.27,12.58)						
ASV_43	Firmicutes;D_2__Clostridia;D_3__Clostridiales;D_4__Ruminococcaceae;D_5__Ruminococcaceae UCG-005;D_6__uncultured organism;D_7__;D_8__;D_9__;D_10__;D_11__;D_12__;D_13__;D_14__	0.97(0.28,3.35)	0.79(0.22,2.80)	0.81(0.24,2.67)	0.96(0.27,3.43)	0.61(0.17,2.17)	0.63(0.19,2.08)			
ASV_46	Firmicutes;D_2__Clostridia;D_3__Clostridiales;D_4__Lachnospiraceae;D_5__Agathobacter	1.73(0.76,3.92)	1.25(0.54,2.90)	0.72(0.33,1.59)	1.73(0.74,4.03)	1.33(0.57,3.11)	0.77(0.35,1.70)			
ASV_47	Firmicutes;D_2__Clostridia;D_3__Clostridiales;D_4__Ruminococcaceae;D_5__Faecalibacterium	1.01(0.61,1.68)	0.75(0.44,1.26)	0.74(0.45,1.21)	1.06(0.62,1.81)	0.76(0.45,1.30)	0.72(0.44,1.19)			
ASV_48	Firmicutes;D_2__Clostridia;D_3__Clostridiales;D_4__Ruminococcaceae;D_5__Intestinimonas;D_6__uncultured bacterium;D_7__;D_8__;D_9__;D_10__;D_11__;D_12__;D_13__;D_14__	0.68(0.16,2.93)	0.67(0.15,2.98)	0.98(0.24,3.96)	0.50(0.11,2.26)	0.61(0.13,2.75)	1.22(0.30,4.91)			
ASV_49	Bacteroidetes;D_2__Bacteroidia;D_3__Bacteroidales;D_4__Rikenellaceae;D_5__Alistipes	0.54(0.16,1.79)	1.02(0.30,3.51)	1.89(0.59,6.04)						
ASV_50	Firmicutes;D_2__Clostridia;D_3__Clostridiales;D_4__Lachnospiraceae;D_5__[Ruminococcus] gauvreauii group	1.13(0.42,3.01)	0.91(0.33,2.51)	0.81(0.31,2.09)	1.28(0.46,3.56)	0.95(0.34,2.65)	0.75(0.29,1.94)			
ASV_51	Firmicutes;D_2__Clostridia;D_3__Clostridiales;D_4__Ruminococcaceae;D_5__Subdoligranulum	1.15(0.57,2.34)	0.85(0.41,1.77)	0.74(0.37,1.47)	1.20(0.58,2.48)	0.84(0.41,1.73)	0.70(0.36,1.37)			
ASV_52	Firmicutes;D_2__Clostridia;D_3__Clostridiales;D_4__Ruminococcaceae;D_5__Ruminiclostridium 5	1.22(0.19,7.78)	0.43(0.06,2.87)	0.35(0.06,2.11)	1.32(0.19,9.12)	0.52(0.08,3.59)	0.40(0.07,2.39)			
ASV_53	Firmicutes;D_2__Clostridia;D_3__Clostridiales;D_4__Lachnospiraceae;D_5__[Ruminococcus] torques group	0.92(0.42,2.00)	0.87(0.39,1.96)	0.95(0.45,2.03)	0.66(0.29,1.49)	0.72(0.32,1.62)	1.09(0.51,2.32)			
ASV_56	Firmicutes;D_2__Bacilli;D_3__Lactobacillales;D_4__Leuconostocaceae;D_5__Weissella	1.79(0.09,34.32)	0.60(0.03,12.34)	0.33(0.02,5.78)						
ASV_60	Firmicutes;D_2__Clostridia;D_3__Clostridiales;D_4__Lachnospiraceae;D_5__Blautia;D_6__uncultured Blautia sp.;D_7__;D_8__;D_9__;D_10__;D_11__;D_12__;D_13__;D_14__	1.06(0.43,2.60)	0.81(0.32,2.05)	0.77(0.32,1.83)	0.97(0.38,2.46)	0.68(0.27,1.72)	0.70(0.29,1.68)			
ASV_62	Firmicutes;D_2__Clostridia;D_3__Clostridiales;D_4__Lachnospiraceae;D_5__Agathobacter;D_6__Eubacterium ramulus;D_7__;D_8__;D_9__;D_10__;D_11__;D_12__;D_13__;D_14__	1.58(0.49,5.15)	1.59(0.47,5.37)	1.01(0.32,3.16)	1.68(0.49,5.79)	1.61(0.47,5.53)	0.96(0.30,3.03)			
ASV_65	Firmicutes;D_2__Clostridia;D_3__Clostridiales;D_4__Ruminococcaceae;D_5__uncultured;D_6__uncultured Clostridium sp.;D_7__;D_8__;D_9__;D_10__;D_11__;D_12__;D_13__;D_14__	0.95(0.25,3.52)	0.70(0.18,2.70)	0.74(0.21,2.63)	0.78(0.20,3.05)	0.75(0.19,2.91)	0.96(0.27,3.41)			
ASV_66	Firmicutes;D_2__Clostridia;D_3__Clostridiales;D_4__Ruminococcaceae;D_5__Subdoligranulum	2.25(1.02,4.97);p=0.04514	2.19(0.97,4.96)	0.97(0.45,2.10)	2.36(1.03,5.42); p=0.04276	2.40(1.05,5.50); p=0.03868	1.02(0.47,2.20)	↑	↑	
ASV_68	Firmicutes;D_2__Clostridia;D_3__Clostridiales;D_4__Family XIII;D_5__Family XIII AD3011 group;D_6__uncultured Eubacterium sp.;D_7__;D_8__;D_9__;D_10__;D_11__;D_12__;D_13__;D_14__	1.17(0.32,4.24)	0.45(0.12,1.67)	0.38(0.11,1.32)	1.27(0.33,4.89)	0.46(0.12,1.74)	0.36(0.10,1.25)			
ASV_69	Bacteroidetes;D_2__Bacteroidia;D_3__Bacteroidales;D_4__Rikenellaceae;D_5__Alistipes;D_6__Alistipes sp. N15.MGS-157;D_7__;D_8__;D_9__;D_10__;D_11__;D_12__;D_13__;D_14__	1.03(0.20,5.19)	0.20(0.04,1.08)	0.20(0.04,0.95); p=0.04314	1.11(0.20,6.05)	0.19(0.04,1.06)	0.18(0.04,0.85);p=0.03071			↓
ASV_70	Firmicutes;D_2__Bacilli;D_3__Lactobacillales;D_4__Streptococcaceae;D_5__Streptococcus;D_6__Streptococcus anginosus subsp. anginosus;D_7__;D_8__;D_9__;D_10__;D_11__;D_12__;D_13__;D_14__	1.49(0.25,8.72)	0.72(0.12,4.32)	0.48(0.09,2.65)	1.39(0.23,8.58)	0.92(0.15,5.50)	0.66(0.12,3.56)			
ASV_71	Firmicutes;D_2__Clostridia;D_3__Clostridiales;D_4__Christensenellaceae;D_5__Christensenellaceae R-7 group	0.24(0.02,3.30)	0.83(0.05,12.48)	3.49(0.27,44.59)						

ASV _72	Firmicutes;D_2__Clostridia;D_3__Clostridiales;D_4__Lachnospiraceae;D_5__Eisenbergiella;D_6__uncultured organism;D_7__;D_8__;D_9__;D_10__;D_11__;D_12__;D_13__;D_14__	0.94(0.28,3.20)	0.52(0.15,1.83)	0.55(0.17,1.79)	1.19(0.33,4.24)	0.58(0.16,2.07)	0.49(0.15,1.60)			
ASV _73	Firmicutes;D_2__Bacilli;D_3__Lactobacillales;D_4__Streptococcaceae;D_5__Streptococcus	3.39(1.11,10.32);p=0.03156	1.71(0.54,5.35)	0.50(0.17,1.48)	3.35(1.04,10.76);p=0.04278	1.80(0.56,5.75)	0.54(0.18,1.60)	↑		
ASV _74	Firmicutes;D_2__Bacilli;D_3__Bacillales;D_4__Family XI;D_5__Gemella	3.24(1.16,9.05);p=0.02463	1.24(0.44,3.52)	0.38(0.14,1.03)	3.33(1.16,9.59); p=0.02554	1.46(0.51,4.13)	0.44(0.16,1.17)	↑		
ASV _75	Firmicutes;D_2__Clostridia;D_3__Clostridiales;D_4__Lachnospiraceae;D_5__Fusicatenibacter	0.91(0.53,1.55)	0.81(0.47,1.41)	0.89(0.53,1.49)	0.90(0.51,1.58)	0.81(0.46,1.42)	0.90(0.53,1.52)			
ASV _76	Firmicutes;D_2__Clostridia;D_3__Clostridiales;D_4__Ruminococcaceae;D_5__Ruminococcaceae NK4A214 group;D_6__uncultured organism;D_7__;D_8__;D_9__;D_10__;D_11__;D_12__;D_13__;D_14__	1.05(0.08,14.07)	0.42(0.03,5.96)	0.40(0.03,4.82)	1.30(0.09,18.90)	0.38(0.03,4.93)	0.29(0.03,3.29)			
ASV _80	Firmicutes;D_2__Clostridia;D_3__Clostridiales;D_4__Peptostreptococcaceae;D_5__Romboutsia	2.03(1.00,4.13)	1.29(0.62,2.68)	0.64(0.32,1.27)	1.85(0.88,3.89)	1.23(0.59,2.59)	0.67(0.33,1.34)			
ASV _81	Bacteroidetes;D_2__Bacteroidia;D_3__Bacteroidales;D_4__Tannerellaceae;D_5__Parabacteroides;D_6__Parabacteroides distasonis;D_7__;D_8__;D_9__;D_10__;D_11__;D_12__;D_13__;D_14__	0.91(0.16,5.04)	0.93(0.16,5.42)	1.02(0.19,5.38)	0.91(0.15,5.38)	1.49(0.25,8.78)	1.64(0.31,8.54)			
ASV _83	Firmicutes;D_2__Clostridia;D_3__Clostridiales;D_4__Lachnospiraceae;D_5__Roseburia;D_6__gut metagenome;D_7__;D_8__;D_9__;D_10__;D_11__;D_12__;D_13__;D_14__	0.55(0.18,1.68)	0.21(0.07,0.65);p=0.00723	0.38(0.13,1.10)	0.56(0.18,1.80)	0.21(0.06,0.66); p=0.00797	0.37(0.13,1.09)		↓	
ASV _85	Firmicutes;D_2__Clostridia;D_3__Clostridiales;D_4__Ruminococcaceae;D_5__Ruminococcaceae UCG-002;D_6__uncultured rumen bacterium;D_7__;D_8__;D_9__;D_10__;D_11__;D_12__;D_13__;D_14__	0.34(0.08,1.48)	0.47(0.10,2.16)	1.39(0.33,5.86)	0.35(0.07,1.60)	0.35(0.08,1.60)	1.00(0.24,4.17)			
ASV _86	Firmicutes;D_2__Clostridia;D_3__Clostridiales;D_4__Ruminococcaceae;D_5__Ruminococcaceae UCG-005;D_6__human gut metagenome;D_7__;D_8__;D_9__;D_10__;D_11__;D_12__;D_13__;D_14__	0.88(0.28,2.72)	0.59(0.19,1.90)	0.68(0.23,2.02)	1.29(0.40,4.18)	0.61(0.19,1.98)	0.48(0.16,1.43)			
ASV _87	Firmicutes;D_2__Clostridia;D_3__Clostridiales;D_4__Ruminococcaceae;D_5__UBA1819;D_6__uncultured organism;D_7__;D_8__;D_9__;D_10__;D_11__;D_12__;D_13__;D_14__	1.13(0.56,2.25)	1.19(0.58,2.42)	1.05(0.54,2.06)	1.15(0.56,2.37)	1.18(0.57,2.42)	1.02(0.52,2.00)			
ASV _88	Firmicutes;D_2__Erysipelotrichia;D_3__Erysipelotrichales;D_4__Erysipelotrichaceae;D_5__[Clostridium] innocuum group	0.63(0.27,1.48)	1.15(0.48,2.77)	1.83(0.80,4.15)	0.85(0.35,2.04)	1.44(0.60,3.47)	1.70(0.75,3.86)			
ASV _89	Firmicutes;D_2__Clostridia;D_3__Clostridiales;D_4__Family XIII;D_5__Family XIII AD3011 group;D_6__uncultured bacterium;D_7__;D_8__;D_9__;D_10__;D_11__;D_12__;D_13__;D_14__	0.59(0.26,1.37)	0.54(0.23,1.28)	0.91(0.41,2.04)	0.66(0.27,1.57)	0.60(0.25,1.44)	0.92(0.41,2.06)			
ASV _90	Firmicutes;D_2__Clostridia;D_3__Clostridiales;D_4__Ruminococcaceae;D_5__Faecalibacterium	0.78(0.31,1.98)	0.70(0.27,1.83)	0.90(0.36,2.22)	0.62(0.24,1.64)	0.65(0.25,1.72)	1.05(0.43,2.58)			
ASV _91	Bacteroidetes;D_2__Bacteroidia;D_3__Bacteroidales;D_4__Rikenellaceae;D_5__Alistipes;D_6__Alistipes obesi;D_7__;D_8__;D_9__;D_10__;D_11__;D_12__;D_13__;D_14__	1.13(0.30,4.28)	0.51(0.13,2.00)	0.45(0.12,1.62)						
ASV _92	Actinobacteria;D_2__Coriobacteriia;D_3__Coriobacteriales;D_4__Eggerthellaceae;D_5__Slackia	0.42(0.03,5.49)	0.13(0.01,1.83)	0.31(0.03,3.70)						
ASV _93	Firmicutes;D_2__Clostridia;D_3__Clostridiales;D_4__Lachnospiraceae;D_5__Roseburia;D_6__metagenome;D_7__;D_8__;D_9__;D_10__;D_11__;D_12__;D_13__;D_14__	1.42(0.19,10.42)	0.09(0.01,0.68);p=0.01969	0.06(0.01,0.42); p=0.0046						
ASV _95	Bacteroidetes;D_2__Bacteroidia;D_3__Bacteroidales;D_4__Rikenellaceae;D_5__Alistipes	0.72(0.25,2.05)	0.50(0.17,1.47)	0.69(0.25,1.92)	0.85(0.28,2.57)	0.53(0.18,1.60)	0.62(0.22,1.74)			
ASV _96	Firmicutes;D_2__Clostridia;D_3__Clostridiales;D_4__Lachnospiraceae;D_5__Hungatella	0.43(0.10,1.73)	1.22(0.29,5.21)	2.87(0.74,11.18)	0.37(0.09,1.63)	0.71(0.16,3.11)	1.91(0.49,7.49)			
ASV _98	Firmicutes;D_2__Clostridia;D_3__Clostridiales;D_4__Lachnospiraceae;D_5__Lachnospiraceae FCS020 group;D_6__uncultured Clostridium sp.;D_7__;D_8__;D_9__;D_10__;D_11__;D_12__;D_13__;D_14__	1.74(0.44,6.84)	1.56(0.38,6.40)	0.90(0.24,3.38)						
ASV _99	Firmicutes;D_2__Clostridia;D_3__Clostridiales;D_4__Lachnospiraceae;D_5__Hungatella;D_6__uncultured bacterium;D_7__;D_8__;D_9__;D_10__;D_11__;D_12__;D_13__;D_14__	1.59(0.39,6.40)	1.24(0.30,5.22)	0.78(0.20,3.02)	2.58(0.62,10.78)	2.24(0.54,9.32)	0.87(0.23,3.29)			
ASV _100	Firmicutes;D_2__Clostridia;D_3__Clostridiales;D_4__Family XIII;D_5__Family XIII AD3011 group	0.31(0.05,1.92)	0.53(0.08,3.40)	1.67(0.30,9.44)	0.86(0.15,5.04)	0.69(0.12,4.06)	0.81(0.16,4.15)			
ASV _101	Firmicutes;D_2__Clostridia;D_3__Clostridiales;D_4__Lachnospiraceae;D_5__Dorea	0.89(0.46,1.71)	0.60(0.31,1.17)	0.67(0.36,1.26)	0.89(0.45,1.76)	0.62(0.31,1.22)	0.69(0.37,1.31)			
ASV _102	Firmicutes;D_2__Negativicutes;D_3__Selenomonadales;D_4__Veillonellaceae;D_5__Veillonella	0.72(0.22,2.36)	0.35(0.10,1.18)	0.48(0.15,1.52)	0.80(0.24,2.66)	0.26(0.08,0.86); p=0.02787	0.33(0.11,1.00)		↓	
ASV _103	Firmicutes;D_2__Clostridia;D_3__Clostridiales;D_4__Lachnospiraceae;D_5__GCA-900066575;D_6__uncultured bacterium;D_7__;D_8__;D_9__;D_10__;D_11__;D_12__;D_13__;D_14__	6.11(0.13,281.86)	5.85(0.11,302.94)	0.96(0.02,41.76)						
ASV _104	Bacteroidetes;D_2__Bacteroidia;D_3__Bacteroidales;D_4__Marinifilaceae;D_5__Odoribacter	0.74(0.29,1.91)	0.56(0.21,1.47)	0.75(0.30,1.86)	0.78(0.29,2.09)	0.63(0.24,1.68)	0.80(0.32,2.01)			
ASV _106	Firmicutes;D_2__Clostridia;D_3__Clostridiales;D_4__Ruminococcaceae;D_5__Ruminiclostridium 6;D_6__uncultured bacterium;D_7__;D_8__;D_9__;D_10__;D_11__;D_12__;D_13__;D_14__	0.45(0.12,1.74)	0.66(0.16,2.64)	1.46(0.39,5.38)	1.04(0.32,3.45)	2.16(0.65,7.19)	2.07(0.68,6.30)			

ASV_107	Firmicutes;D_2__Clostridia;D_3__Clostridiales;D_4__Lachnospiraceae;D_5__CAG-56;D_6__uncultured bacterium;D_7__;D_8__;D_9__;D_10__;D_11__;D_12__;D_13__;D_14__	1.61(0.43,5.98)	1.35(0.35,5.24)	0.84(0.24,3.01)	1.37(0.35,5.38)	1.39(0.35,5.44)	1.01(0.28,3.62)			
ASV_109	Firmicutes;D_2__Clostridia;D_3__Clostridiales;D_4__Lachnospiraceae;D_5__Lachnospiraceae FCS020 group;D_6__uncultured organism;D_7__;D_8__;D_9__;D_10__;D_11__;D_12__;D_13__;D_14__	1.33(0.58,3.04)	0.97(0.41,2.27)	0.73(0.33,1.62)	1.19(0.50,2.82)	0.88(0.37,2.09)	0.74(0.33,1.66)			
ASV_112	Firmicutes;D_2__Clostridia;D_3__Clostridiales;D_4__Ruminococcaceae	1.77(0.08,39.46)	0.68(0.03,16.51)	0.38(0.02,7.74)						
ASV_119	Bacteroidetes;D_2__Bacteroidia;D_3__Bacteroidales;D_4__Bacteroidaceae;D_5__Bacteroides	0.51(0.11,2.32)	0.64(0.14,3.06)	1.26(0.29,5.47)	0.45(0.09,2.23)	0.57(0.12,2.77)	1.25(0.28,5.48)			
ASV_122	Firmicutes;D_2__Clostridia;D_3__Clostridiales;D_4__Lachnospiraceae;D_5__Eisenbergiella	0.18(0.01,6.39)	0.62(0.01,27.68)	3.35(0.14,82.18)						
ASV_123	Firmicutes;D_2__Clostridia;D_3__Clostridiales;D_4__Ruminococcaceae;D_5__uncultured				960467039155816.00(0.00,Inf)	0.47(0.03,7.34)	0.00(0.00,Inf)			
ASV_124	Proteobacteria;D_2__Deltaproteobacteria;D_3__Desulfovibrionales;D_4__Desulfovibrionaceae;D_5__Bilophila;D_6__uncultured bacterium;D_7__;D_8__;D_9__;D_10__;D_11__;D_12__;D_13__;D_14__	0.78(0.29,2.12)	1.09(0.39,3.07)	1.40(0.53,3.70)	0.65(0.23,1.85)	1.13(0.40,3.21)	1.74(0.66,4.60)			
ASV_125	Firmicutes;D_2__Clostridia;D_3__Clostridiales;D_4__Peptococcaceae;D_5__uncultured;D_6__uncultured Peptococcaceae bacterium;D_7__;D_8__;D_9__;D_10__;D_11__;D_12__;D_13__;D_14__				40.18(2.12,761.25);p=0.01386	6.88(0.48,99.30)	0.17(0.01,2.65)	↑		
ASV_126	Firmicutes;D_2__Clostridia;D_3__Clostridiales;D_4__Christensenellaceae;D_5__Christensenellaceae R-7 group	0.04(0.00,0.42);p=0.00678	0.09(0.01,0.94);p=0.04436	2.11(0.25,17.85)						
ASV_127	Actinobacteria;D_2__Actinobacteria;D_3__Actinomycetales;D_4__Actinomycetaceae;D_5__Actinomyces	3.24(1.52,6.92);p=0.00239	1.16(0.53,2.51)	0.36(0.17,0.74);p=0.00604						
ASV_128	Firmicutes;D_2__Clostridia;D_3__Clostridiales;D_4__Family XIII;D_5__[Eubacterium] brachy group;D_6__uncultured bacterium;D_7__;D_8__;D_9__;D_10__;D_11__;D_12__;D_13__;D_14__	1.35(0.46,3.96)	0.76(0.25,2.30)	0.57(0.20,1.60)	1.66(0.55,5.02)	0.82(0.27,2.46)	0.49(0.18,1.38)			
ASV_131	Firmicutes;D_2__Clostridia;D_3__Clostridiales;D_4__Lachnospiraceae;D_5__Lachnoclostridium	0.92(0.41,2.05)	1.33(0.58,3.04)	1.45(0.66,3.16)	0.72(0.31,1.68)	1.16(0.50,2.68)	1.60(0.73,3.49)			
ASV_132	Firmicutes;D_2__Clostridia;D_3__Clostridiales;D_4__Lachnospiraceae;D_5__[Eubacterium] eligens group	0.65(0.19,2.19)	0.25(0.07,0.87);p=0.03	0.38(0.12,1.25)	0.72(0.20,2.61)	0.26(0.07,0.93);p=0.03763	0.36(0.11,1.17)		↓	
ASV_134	Firmicutes;D_2__Clostridia;D_3__Clostridiales;D_4__Ruminococcaceae;D_5__Ruminococcaceae UCG-013;D_6__uncultured organism;D_7__;D_8__;D_9__;D_10__;D_11__;D_12__;D_13__;D_14__	0.65(0.34,1.25)	0.91(0.46,1.79)	1.40(0.74,2.64)	0.57(0.29,1.13)	0.83(0.42,1.64)	1.45(0.76,2.74)			
ASV_135	Firmicutes;D_2__Clostridia;D_3__Clostridiales;D_4__Lachnospiraceae;D_5__Roseburia	0.80(0.29,2.15)	0.33(0.12,0.91);p=0.03276	0.41(0.16,1.08)	0.92(0.33,2.55)	0.37(0.14,1.03)	0.40(0.16,1.04)			
ASV_136	Firmicutes;D_2__Clostridia;D_3__Clostridiales;D_4__Ruminococcaceae;D_5__Ruminiclostridium 5;D_6__uncultured organism;D_7__;D_8__;D_9__;D_10__;D_11__;D_12__;D_13__;D_14__	1.14(0.60,2.17)	1.32(0.68,2.57)	1.16(0.62,2.16)	1.21(0.62,2.38)	1.35(0.69,2.65)	1.11(0.59,2.08)			
ASV_137	Firmicutes;D_2__Negativicutes;D_3__Selenomonadales;D_4__Veillonellaceae;D_5__Veillonella	0.28(0.09,0.87);p=0.02818	0.57(0.18,1.88)	2.08(0.69,6.29)	0.40(0.12,1.31)	0.59(0.18,1.95)	1.48(0.49,4.44)			
ASV_141	Firmicutes;D_2__Clostridia;D_3__Clostridiales;D_4__Ruminococcaceae;D_5__Ruminiclostridium 5;D_6__gut metagenome;D_7__;D_8__;D_9__;D_10__;D_11__;D_12__;D_13__;D_14__	0.76(0.33,1.75)	0.81(0.35,1.92)	1.07(0.48,2.40)	0.82(0.34,1.98)	0.85(0.36,2.04)	1.03(0.46,2.34)			
ASV_144	Firmicutes;D_2__Bacilli;D_3__Lactobacillales;D_4__Carnobacteriaceae;D_5__Granulicatella	2.62(1.14,6.04);p=0.02349	1.31(0.56,3.07)	0.50(0.22,1.12)	2.67(1.11,6.40);p=0.02813	1.37(0.57,3.27)	0.51(0.23,1.16)	↑		
ASV_145	Bacteroidetes;D_2__Bacteroidia;D_3__Bacteroidales;D_4__Rikenellaceae;D_5__Alistipes;D_6__Faecalibacterium prausnitzii;D_7__;D_8__;D_9__;D_10__;D_11__;D_12__;D_13__;D_14__				0.01(0.00,0.17);p=0.00108	0.28(0.02,3.62)	22.16(2.09,235.58);p=0.01019	↓		↑
ASV_146	Firmicutes;D_2__Negativicutes;D_3__Selenomonadales;D_4__Veillonellaceae;D_5__Veillonella	0.78(0.25,2.50)	0.15(0.05,0.50);p=0.00186	0.19(0.06,0.59);p=0.00383	0.76(0.23,2.53)	0.14(0.04,0.45);p=0.00107	0.18(0.06,0.54);p=0.00244		↓	↓
ASV_148	Firmicutes;D_2__Clostridia;D_3__Clostridiales;D_4__Ruminococcaceae	1.25(0.40,3.93)	0.85(0.26,2.75)	0.68(0.23,2.05)	1.17(0.36,3.81)	0.62(0.19,1.98)	0.53(0.18,1.57)			
ASV_150	Firmicutes;D_2__Bacilli;D_3__Lactobacillales;D_4__Streptococcaceae;D_5__Lactococcus	3.99(1.34,11.89);p=0.01309	5.68(1.84,17.53);p=0.00249	1.43(0.49,4.11)	2.38(0.77,7.31)	4.76(1.55,14.61);p=0.00649	2.00(0.70,5.71)		↑	
ASV_153	Firmicutes;D_2__Clostridia;D_3__Clostridiales;D_4__Lachnospiraceae;D_5__Lachnospiraceae NK4A136 group;D_6__uncultured organism;D_7__;D_8__;D_9__;D_10__;D_11__;D_12__;D_13__;D_14__	0.51(0.18,1.41)	0.58(0.20,1.66)	1.14(0.42,3.04)	0.47(0.16,1.36)	0.75(0.26,2.16)	1.58(0.59,4.26)			
ASV_154	Bacteroidetes;D_2__Bacteroidia;D_3__Bacteroidales;D_4__Bacteroidaceae;D_5__Bacteroides	1.10(0.27,4.47)	1.44(0.34,6.13)	1.32(0.34,5.13)	1.25(0.29,5.40)	2.03(0.47,8.76)	1.63(0.42,6.38)			
ASV_155	Firmicutes;D_2__Erysipelotrichia;D_3__Erysipelotrichales;D_4__Erysipelotrichaceae;D_5__Erysipelotrichaceae UCG-003	0.99(0.41,2.38)	0.67(0.27,1.65)	0.67(0.29,1.57)	0.81(0.33,2.02)	0.58(0.24,1.45)	0.72(0.31,1.67)			
ASV_157	Firmicutes;D_2__Clostridia;D_3__Clostridiales;D_4__Peptostreptococcaceae;D_5__Peptostreptococcus;D_6__uncultured organism;D_7__;D_8__;D_9__;D_10__;D_11__;D_12__;D_13__;D_14__	3.01(0.42,21.51)	2.14(0.29,16.05)	0.71(0.10,4.89)						
ASV_158	Firmicutes;D_2__Clostridia;D_3__Clostridiales;D_4__Lachnospiraceae;D_5__Lachnoclostridium;D_6__human gut metagenome;D_7__;D_8__;D_9__;D_10__;D_11__;D_12__;D_13__;D_14__	0.78(0.34,1.78)	1.31(0.56,3.07)	1.68(0.76,3.73)	0.62(0.26,1.46)	1.01(0.43,2.38)	1.63(0.73,3.65)			

ASV _160	Firmicutes;D_2__Clostridia;D_3__Clostridiales;D_4__Ruminococcaceae	0.57(0.05,6.23)	0.30(0.03,3.46)	0.53(0.06,5.02)						
ASV _161	Firmicutes;D_2__Clostridia;D_3__Clostridiales;D_4__Defluviitaleaceae;D_5__Defluviitaleaceae UCG-011;D_6__uncultured bacterium;D_7__;D_8__;D_9__;D_10__;D_11__;D_12__;D_13__;D_14__	0.37(0.07,1.87)	0.38(0.07,2.03)	1.04(0.22,4.92)	0.38(0.07,2.03)	0.40(0.07,2.18)	1.07(0.23,5.09)			
ASV _163	Firmicutes;D_2__Clostridia;D_3__Clostridiales;D_4__Ruminococcaceae	2.02(0.26,15.77)	0.79(0.10,6.42)	0.39(0.05,2.86)						
ASV _167	Firmicutes;D_2__Clostridia;D_3__Clostridiales;D_4__Clostridiales vadinBB60 group;D_5__uncultured Thermoanaerobacterales bacterium;D_6__;D_7__;D_8__;D_9__;D_10__;D_11__;D_12__;D_13__;D_14__	8.57(0.28,262.35)	0.32(0.01,10.65)	0.04(0.00,1.03)						
ASV _168	Firmicutes;D_2__Clostridia;D_3__Clostridiales;D_4__Lachnospiraceae	0.48(0.16,1.47)	0.27(0.09,0.85);p=0.02538	0.56(0.19,1.64)	0.27(0.09,0.87); p=0.02892	0.17(0.05,0.56); p=0.00319	0.64(0.22,1.86)	↓	↓	
ASV _169	Firmicutes;D_2__Clostridia;D_3__Clostridiales;D_4__Lachnospiraceae;D_5__[Eubacterium] eligens group	1.52(0.34,6.75)	0.28(0.06,1.28)	0.18(0.04,0.77); p=0.02091	1.79(0.37,8.54)	0.35(0.07,1.68)	0.20(0.05,0.85);p=0.02919			↓
ASV _170	Firmicutes;D_2__Clostridia;D_3__Clostridiales;D_4__Lachnospiraceae;D_5__Lachnospiraceae NK4A136 group	0.37(0.14,0.97);p=0.04256	0.20(0.07,0.53);p=0.00123	0.53(0.21,1.34)	0.42(0.16,1.11)	0.21(0.08,0.56); p=0.00179	0.51(0.20,1.25)		↓	
ASV _171	Firmicutes;D_2__Clostridia;D_3__Clostridiales;D_4__Ruminococcaceae;D_5__Ruminococcaceae NK4A214 group;D_6__uncultured organism;D_7__;D_8__;D_9__;D_10__;D_11__;D_12__;D_13__;D_14__	1.11(0.16,7.65)	0.99(0.14,7.23)	0.89(0.14,5.79)						
ASV _172	Firmicutes;D_2__Erysipelotrichia;D_3__Erysipelotrichales;D_4__Erysipelotrichaceae;D_5__Holdemania	1.34(0.47,3.85)	2.91(0.97,8.70)	2.17(0.77,6.10)	2.14(0.72,6.36)	4.28(1.43,12.86);p=0.00949	2.00(0.72,5.61)		↑	
ASV _173	Firmicutes;D_2__Clostridia;D_3__Clostridiales;D_4__Lachnospiraceae;D_5__Sellimonas;D_6__uncultured bacterium;D_7__;D_8__;D_9__;D_10__;D_11__;D_12__;D_13__;D_14__	0.30(0.02,4.67)	5.04(0.26,98.41)	16.54(1.02,267.92);p=0.04827	0.22(0.02,2.78)	15.15(0.45,509.10)	70.29(2.87,1720.26);p=0.00914			↑
ASV _174	Firmicutes;D_2__Clostridia;D_3__Clostridiales;D_4__Lachnospiraceae;D_5__uncultured;D_6__uncultured organism;D_7__;D_8__;D_9__;D_10__;D_11__;D_12__;D_13__;D_14__	1.79(0.52,6.25)	2.19(0.60,7.91)	1.22(0.36,4.09)	1.58(0.43,5.85)	2.16(0.59,7.98)	1.37(0.40,4.63)			
ASV _175	Firmicutes;D_2__Clostridia;D_3__Clostridiales;D_4__Christensenellaceae;D_5__uncultured;D_6__uncultured bacterium;D_7__;D_8__;D_9__;D_10__;D_11__;D_12__;D_13__;D_14__	0.34(0.08,1.40)	0.25(0.06,1.05)	0.72(0.19,2.73)	0.29(0.07,1.28)	0.26(0.06,1.11)	0.87(0.23,3.32)			
ASV _177	Firmicutes;D_2__Clostridia;D_3__Clostridiales;D_4__Christensenellaceae;D_5__Christensenellaceae R-7 group	0.75(0.05,12.08)	2.76(0.15,49.23)	3.66(0.24,54.81)						
ASV _179	Firmicutes;D_2__Clostridia;D_3__Clostridiales;D_4__Ruminococcaceae;D_5__DTU089	0.80(0.34,1.87)	1.47(0.61,3.52)	1.83(0.81,4.16)						
ASV _180	Actinobacteria;D_2__Coriobacteriia;D_3__Coriobacteriales;D_4__Coriobacteriales Incertae Sedis;D_5__uncultured;D_6__gut metagenome;D_7__;D_8__;D_9__;D_10__;D_11__;D_12__;D_13__;D_14__	0.83(0.15,4.59)	1.24(0.21,7.20)	1.49(0.29,7.77)	1.02(0.17,6.06)	1.20(0.20,7.12)	1.18(0.22,6.20)			
ASV _181	Firmicutes;D_2__Clostridia;D_3__Clostridiales;D_4__Ruminococcaceae;D_5__Anaerotruncus;D_6__Anaerotruncus sp. AT3;D_7__;D_8__;D_9__;D_10__;D_11__;D_12__;D_13__;D_14__	0.34(0.04,3.29)	0.66(0.06,6.93)	1.94(0.22,16.87)						
ASV _182	Firmicutes;D_2__Clostridia;D_3__Clostridiales;D_4__Lachnospiraceae	1.77(0.92,3.40)	1.33(0.68,2.60)	0.75(0.40,1.43)	1.87(0.94,3.71)	1.40(0.70,2.76)	0.75(0.39,1.42)			
ASV _183	Actinobacteria;D_2__Coriobacteriia;D_3__Coriobacteriales;D_4__Eggerthellaceae	0.83(0.31,2.18)	1.01(0.37,2.73)	1.21(0.48,3.10)	0.81(0.29,2.24)	0.97(0.35,2.68)	1.19(0.46,3.07)			
ASV _184	Firmicutes;D_2__Clostridia;D_3__Clostridiales;D_4__Ruminococcaceae;D_5__uncultured	0.66(0.14,3.12)	0.32(0.06,1.58)	0.49(0.11,2.18)						
ASV _185	Firmicutes;D_2__Clostridia;D_3__Clostridiales;D_4__Ruminococcaceae;D_5__Anaerotruncus;D_6__Anaerotruncus rubiinfantis;D_7__;D_8__;D_9__;D_10__;D_11__;D_12__;D_13__;D_14__	1.36(0.27,6.84)	0.52(0.10,2.59)	0.38(0.08,1.75)	0.87(0.16,4.80)	0.37(0.07,1.92)	0.42(0.09,1.93)			
ASV _187	Firmicutes;D_2__Clostridia;D_3__Clostridiales;D_4__Christensenellaceae;D_5__uncultured	0.41(0.03,5.63)	0.70(0.05,10.83)	1.70(0.15,19.53)						
ASV _189	Firmicutes;D_2__Clostridia;D_3__Clostridiales;D_4__Lachnospiraceae;D_5__[Eubacterium] ventriosum group	1.41(0.54,3.73)	0.68(0.25,1.86)	0.48(0.19,1.24)	1.24(0.45,3.39)	0.52(0.19,1.41)	0.42(0.16,1.07)			
ASV _191	Proteobacteria;D_2__Gammaproteobacteria;D_3__Pseudomonadales;D_4__Pseudomonadaceae;D_5__Pseudomonas	10.59(2.57,43.62);p=0.00109	2.95(0.70,12.37)	0.28(0.07,1.11)						
ASV _192	Firmicutes;D_2__Clostridia;D_3__Clostridiales;D_4__Lachnospiraceae;D_5__GCA-900066575	0.55(0.07,4.24)	0.71(0.09,5.89)	1.30(0.18,9.34)						
ASV _196	Firmicutes;D_2__Clostridia;D_3__Clostridiales;D_4__Ruminococcaceae;D_5__Angelakisella;D_6__uncultured bacterium;D_7__;D_8__;D_9__;D_10__;D_11__;D_12__;D_13__;D_14__				2.34(0.31,17.50)	0.87(0.12,6.45)	0.37(0.06,2.43)			
ASV _197	Firmicutes;D_2__Erysipelotrichia;D_3__Erysipelotrichales;D_4__Erysipelotrichaceae;D_5__Erysipelatoclostridium;D_6__unidentified;D_7__;D_8__;D_9__;D_10__;D_11__;D_12__;D_13__;D_14__	0.57(0.02,13.68)	1.86(0.07,50.41)	3.29(0.15,72.73)						
ASV _198	Actinobacteria;D_2__Coriobacteriia;D_3__Coriobacteriales;D_4__Eggerthellaceae;D_5__Gordonibacter;D_6__unidentified;D_7__;D_8__;D_9__;D_10__;D_11__;D_12__;D_13__;D_14__	1.27(0.61,2.64)	0.89(0.42,1.87)	0.70(0.34,1.41)	1.37(0.64,2.93)	0.94(0.44,2.00)	0.68(0.34,1.39)			

ASV_199	Firmicutes;D_2__Clostridia;D_3__Clostridiales;D_4__Lachnospiraceae;D_5__Lachnospiraceae UCG-010;D_6__uncultured organism;D_7__;D_8__;D_9__;D_10__;D_11__;D_12__;D_13__;D_14__	0.59(0.15,2.38)	0.45(0.11,1.87)	0.75(0.20,2.91)	0.43(0.10,1.81)	0.48(0.11,2.05)	1.13(0.29,4.36)			
ASV_202	Firmicutes;D_2__Clostridia;D_3__Clostridiales;D_4__Lachnospiraceae;D_5__GCA-900066755;D_6__uncultured bacterium;D_7__;D_8__;D_9__;D_10__;D_11__;D_12__;D_13__;D_14__	0.58(0.14,2.48)	1.14(0.25,5.15)	1.97(0.48,7.99)	0.76(0.17,3.44)	1.26(0.28,5.73)	1.65(0.41,6.70)			
ASV_203	Firmicutes;D_2__Clostridia;D_3__Clostridiales;D_4__Christensenellaceae;D_5__Christensenellaceae R-7 group;D_6__uncultured soil bacterium;D_7__;D_8__;D_9__;D_10__;D_11__;D_12__;D_13__;D_14__	3.11(0.47,20.61)	1.74(0.26,11.69)	0.56(0.09,3.59)	5.26(0.71,38.84)	2.28(0.33,15.60)	0.43(0.07,2.86)			
ASV_206	Actinobacteria;D_2__Coriobacteriia;D_3__Coriobacteriales;D_4__Eggerthellaceae;D_5__Gordonibacter;D_6__uncultured bacterium;D_7__;D_8__;D_9__;D_10__;D_11__;D_12__;D_13__;D_14__	3.11(0.35,27.72)	3.62(0.38,34.67)	1.16(0.14,9.94)	4.57(0.50,41.98)	5.19(0.56,47.76)	1.13(0.14,9.29)			
ASV_207	Firmicutes;D_2__Clostridia;D_3__Clostridiales;D_4__Lachnospiraceae;D_5__Lachnoclostridium	1.34(0.07,26.43)	0.32(0.02,5.84)	0.24(0.02,3.75)	0.54(0.02,13.43)	0.19(0.01,4.04)	0.34(0.02,5.23)			
ASV_209	Firmicutes;D_2__Clostridia;D_3__Clostridiales;D_4__Lachnospiraceae;D_5__GCA-900066575;D_6__uncultured organism;D_7__;D_8__;D_9__;D_10__;D_11__;D_12__;D_13__;D_14__	1.50(0.31,7.10)	0.63(0.13,3.12)	0.42(0.09,1.91)	2.17(0.43,10.89)	1.17(0.24,5.78)	0.54(0.12,2.41)			
ASV_210	Firmicutes;D_2__Clostridia;D_3__Clostridiales;D_4__Christensenellaceae;D_5__Christensenellaceae R-7 group	4.01(0.29,55.71)	1.32(0.09,18.97)	0.33(0.03,4.25)						
ASV_211	Firmicutes;D_2__Clostridia;D_3__Clostridiales;D_4__Christensenellaceae;D_5__uncultured;D_6__uncultured bacterium;D_7__;D_8__;D_9__;D_10__;D_11__;D_12__;D_13__;D_14__	0.29(0.02,3.99)	0.15(0.01,2.18)	0.51(0.04,6.37)	1.46(0.10,21.86)	0.62(0.04,9.08)	0.42(0.03,5.21)			
ASV_212	Actinobacteria;D_2__Coriobacteriia;D_3__Coriobacteriales;D_4__Eggerthellaceae;D_5__Adlercreutzia;D_6__uncultured bacterium;D_7__;D_8__;D_9__;D_10__;D_11__;D_12__;D_13__;D_14__	1.09(0.46,2.55)	1.19(0.49,2.85)	1.09(0.48,2.49)	1.11(0.45,2.71)	1.18(0.48,2.88)	1.07(0.46,2.45)			
ASV_214	Actinobacteria;D_2__Actinobacteria;D_3__Actinomycetales;D_4__Actinomycetaceae;D_5__Actinomyces	2.99(0.12,75.34)	2.99(0.11,83.93)	1.00(0.04,27.71)						
ASV_215	Firmicutes;D_2__Clostridia;D_3__Clostridiales;D_4__Lachnospiraceae;D_5__Lachnoclostridium	1.13(0.39,3.33)	1.57(0.52,4.77)	1.39(0.49,3.94)	1.14(0.37,3.49)	1.40(0.46,4.29)	1.23(0.43,3.50)			
ASV_216	Firmicutes;D_2__Clostridia;D_3__Clostridiales;D_4__Lachnospiraceae;D_5__Lachnospiraceae NK4A136 group;D_6__uncultured organism;D_7__;D_8__;D_9__;D_10__;D_11__;D_12__;D_13__;D_14__	0.51(0.17,1.53)	0.52(0.17,1.62)	1.02(0.35,2.98)	0.65(0.21,2.03)	0.82(0.26,2.55)	1.26(0.44,3.65)			
ASV_218	Firmicutes;D_2__Clostridia;D_3__Clostridiales;D_4__Ruminococcaceae;D_5__Ruminococcaceae UCG-003;D_6__uncultured organism;D_7__;D_8__;D_9__;D_10__;D_11__;D_12__;D_13__;D_14__	0.23(0.08,0.71);p=0.01039	0.15(0.05,0.47);p=0.00113	0.64(0.22,1.87)	0.22(0.07,0.72); p=0.01173	0.15(0.05,0.48); p=0.00133	0.67(0.23,1.97)	↓	↓	
ASV_219	Firmicutes;D_2__Clostridia;D_3__Clostridiales;D_4__Christensenellaceae;D_5__Christensenellaceae R-7 group	1.30(0.20,8.47)	0.56(0.08,3.80)	0.43(0.07,2.62)						
ASV_220	Firmicutes;D_2__Clostridia;D_3__Clostridiales;D_4__Ruminococcaceae;D_5__Hydrogenoanaerobacterium	0.66(0.04,10.65)	0.08(0.01,1.23)	0.12(0.01,1.39)						
ASV_222	Firmicutes;D_2__Clostridia;D_3__Clostridiales;D_4__Lachnospiraceae;D_5__uncultured;D_6__intestinal bacterium CG19-1;D_7__;D_8__;D_9__;D_10__;D_11__;D_12__;D_13__;D_14__	0.81(0.12,5.38)	0.73(0.10,5.15)	0.91(0.15,5.66)	0.74(0.10,5.20)	0.84(0.12,5.91)	1.14(0.19,6.98)			
ASV_223	Firmicutes;D_2__Clostridia;D_3__Clostridiales;D_4__Christensenellaceae;D_5__Catabacter;D_6__Christensenella massiliensis;D_7__;D_8__;D_9__;D_10__;D_11__;D_12__;D_13__;D_14__	0.33(0.02,5.99)	0.21(0.01,4.26)	0.66(0.04,10.51)						
ASV_224	Firmicutes;D_2__Clostridia;D_3__Clostridiales;D_4__Ruminococcaceae;D_5__Ruminococcaceae UCG-007	3.67(0.43,31.26)	0.44(0.06,3.53)	0.12(0.02,0.94); p=0.04354						
ASV_225	Firmicutes;D_2__Clostridia;D_3__Clostridiales;D_4__Lachnospiraceae;D_5__uncultured	0.82(0.13,5.08)	0.61(0.09,3.99)	0.74(0.13,4.34)	1.36(0.20,9.06)	0.78(0.12,5.14)	0.57(0.10,3.33)			
ASV_227	Firmicutes;D_2__Clostridia;D_3__Clostridiales;D_4__Lachnospiraceae;D_5__Lachnospiraceae NK4A136 group;D_6__uncultured organism;D_7__;D_8__;D_9__;D_10__;D_11__;D_12__;D_13__;D_14__	0.64(0.26,1.62)	0.77(0.30,2.00)	1.20(0.49,2.93)	0.69(0.26,1.82)	0.89(0.34,2.34)	1.29(0.53,3.17)			
ASV_231	Firmicutes;D_2__Clostridia;D_3__Clostridiales;D_4__Lachnospiraceae;D_5__GCA-900066575;D_6__uncultured bacterium;D_7__;D_8__;D_9__;D_10__;D_11__;D_12__;D_13__;D_14__	2.17(0.46,10.30)	2.64(0.53,13.23)	1.22(0.26,5.61)	2.84(0.56,14.53)	3.08(0.60,15.76)	1.08(0.23,5.04)			
ASV_233	Proteobacteria;D_2__Deltaproteobacteria;D_3__Desulfovibrionales;D_4__Desulfovibrionaceae;D_5__Desulfovibrio;D_6__Desulfovibrio desulfuricans;D_7__;D_8__;D_9__;D_10__;D_11__;D_12__;D_13__;D_14__	3.57(0.15,82.84)	0.50(0.02,12.59)	0.14(0.01,2.94)	1.55(0.06,37.33)	0.39(0.02,9.27)	0.25(0.01,4.90)			
ASV_234	Firmicutes;D_2__Clostridia;D_3__Clostridiales;D_4__Ruminococcaceae;D_5__Ruminiclostridium 5;D_6__uncultured bacterium;D_7__;D_8__;D_9__;D_10__;D_11__;D_12__;D_13__;D_14__	3.88(0.20,74.32)	3.40(0.16,71.09)	0.88(0.05,15.73)						
ASV_235	Firmicutes;D_2__Clostridia;D_3__Clostridiales;D_4__Christensenellaceae;D_5__Christensenellaceae R-7 group;D_6__uncultured prokaryote;D_7__;D_8__;D_9__;D_10__;D_11__;D_12__;D_13__;D_14__	1.04(0.40,2.67)	1.04(0.39,2.75)	1.00(0.40,2.50)	1.20(0.44,3.22)	1.19(0.44,3.20)	0.99(0.40,2.50)			
ASV_237	Firmicutes;D_2__Clostridia;D_3__Clostridiales;D_4__Lachnospiraceae;D_5__Lachnospira	1.44(0.39,5.31)	1.54(0.40,5.94)	1.07(0.30,3.81)	0.41(0.11,1.53)	1.00(0.27,3.73)	2.45(0.72,8.37)			

ASV_238	Firmicutes;D_2__Clostridia;D_3__Clostridiales;D_4__Ruminococcaceae	23.99(2.40,240.30);p=0.00687	3.05(0.42,22.01)	0.13(0.01,1.26)	174.56(10.94,2785.26);p=0.00026	11.34(1.57,81.69);p=0.01594	0.06(0.00,0.86);p=0.03787	↑	↑	↓
ASV_240	Firmicutes;D_2__Clostridia;D_3__Clostridiales;D_4__Christensenellaceae;D_5__Christensenellaceae R-7 group;D_6__uncultured bacterium;D_7__;D_8__;D_9__;D_10__;D_11__;D_12__;D_13__;D_14__	1.40(0.17,11.74)	0.78(0.09,6.84)	0.56(0.07,4.32)	0.85(0.09,8.08)	0.33(0.04,2.98)	0.38(0.05,2.98)			
ASV_241	Firmicutes;D_2__Clostridia;D_3__Clostridiales;D_4__Ruminococcaceae;D_5__uncultured	1.65(0.15,17.86)	0.39(0.04,4.30)	0.24(0.02,2.31)	1.64(0.14,18.85)	0.46(0.04,4.95)	0.28(0.03,2.64)			
ASV_242	Firmicutes;D_2__Bacilli;D_3__Bacillales;D_4__Staphylococcaceae;D_5__Staphylococcus	1.24(0.27,5.76)	0.88(0.18,4.24)	0.71(0.16,3.12)	1.40(0.28,6.96)	1.02(0.21,5.05)	0.73(0.16,3.25)			
ASV_245	Firmicutes;D_2__Clostridia;D_3__Clostridiales;D_4__Peptococcaceae;D_5__uncultured;D_6__metagenome;D_7__;D_8__;D_9__;D_10__;D_11__;D_12__;D_13__;D_14__	0.47(0.05,4.27)	0.46(0.05,4.40)	0.97(0.12,7.55)	0.63(0.06,6.49)	0.21(0.02,1.87)	0.33(0.04,2.45)			
ASV_246	Firmicutes;D_2__Clostridia;D_3__Clostridiales;D_4__Lachnospiraceae	1.33(0.14,12.75)	2.10(0.19,23.08)	1.57(0.16,15.51)	3.42(0.31,37.36)	3.01(0.29,31.28)	0.88(0.08,9.39)			
ASV_249	Firmicutes;D_2__Clostridia;D_3__Clostridiales;D_4__Lachnospiraceae;D_5__Lachnoclostridium	0.67(0.21,2.15)	2.02(0.60,6.84)	3.03(0.97,9.49)	0.49(0.15,1.63)	1.37(0.41,4.63)	2.81(0.91,8.70)			
ASV_252	Firmicutes;D_2__Erysipelotrichia;D_3__Erysipelotrichales;D_4__Erysipelotrichaceae;D_5__Erysipelatoclostridium	0.28(0.07,1.16)	2.64(0.60,11.55)	9.41(2.36,37.59);p=0.00151	0.30(0.07,1.32)	2.23(0.50,9.98)	7.49(1.86,30.20);p=0.00463			↑
ASV_253	Firmicutes;D_2__Clostridia;D_3__Clostridiales;D_4__Family XIII;D_5__Mogibacterium	2.91(0.51,16.54)	0.37(0.07,2.02)	0.13(0.02,0.67);p=0.01474						
ASV_254	Firmicutes;D_2__Clostridia;D_3__Clostridiales;D_4__Lachnospiraceae	2.85(0.34,23.99)	1.61(0.18,14.37)	0.56(0.07,4.44)	3.78(0.45,31.98)	1.54(0.18,12.92)	0.41(0.06,3.00)			
ASV_255	Actinobacteria;D_2__Actinobacteria;D_3__Actinomycetales;D_4__Actinomycetaceae;D_5__Actinomyces	0.68(0.29,1.63)	0.47(0.19,1.14)	0.69(0.30,1.56)	0.83(0.34,2.03)	0.48(0.20,1.17)	0.58(0.26,1.31)			
ASV_256	Firmicutes;D_2__Clostridia;D_3__Clostridiales;D_4__Family XIII;D_5__Family XIII AD3011 group;D_6__uncultured bacterium;D_7__;D_8__;D_9__;D_10__;D_11__;D_12__;D_13__;D_14__	2.93(0.53,16.12)	1.09(0.19,6.20)	0.37(0.07,1.94)	4.19(0.72,24.41)	0.70(0.13,3.86)	0.17(0.03,0.87);p=0.03304			↓
ASV_258	Firmicutes;D_2__Clostridia;D_3__Clostridiales;D_4__Defluviitaleaceae;D_5__Defluviitaleaceae UCG-011;D_6__uncultured bacterium;D_7__;D_8__;D_9__;D_10__;D_11__;D_12__;D_13__;D_14__	2.13(0.43,10.61)	0.86(0.17,4.41)	0.40(0.09,1.91)	1.40(0.27,7.25)	0.69(0.14,3.53)	0.50(0.11,2.27)			
ASV_259	Patescibacteria;D_2__Saccharimonadia;D_3__Saccharimonadales;D_4__Saccharimonadaceae	4.39(1.16,16.61);p=0.02918	1.06(0.28,4.04)	0.24(0.07,0.88);p=0.03086	5.05(1.28,19.93);p=0.0208	1.26(0.33,4.78)	0.25(0.07,0.90);p=0.03331	↑		↓
ASV_260	Actinobacteria;D_2__Coriobacteriia;D_3__Coriobacteriales;D_4__Eggerthellaceae;D_5__Enterorhabdus;D_6__uncultured bacterium;D_7__;D_8__;D_9__;D_10__;D_11__;D_12__;D_13__;D_14__	6.45(0.89,46.68)	0.36(0.05,2.48)	0.06(0.01,0.38);p=0.00313	9.84(1.11,87.29);p=0.03998	0.17(0.03,1.11)	0.02(0.00,0.13);p=0.00007;q=0.04705	↑		↓*
ASV_262	Firmicutes;D_2__Clostridia;D_3__Clostridiales;D_4__Defluviitaleaceae;D_5__Defluviitaleaceae UCG-011;D_6__uncultured bacterium;D_7__;D_8__;D_9__;D_10__;D_11__;D_12__;D_13__;D_14__	0.00(0.00,Inf)	0.00(0.00,Inf)	5.30(0.26,107.86)						
ASV_264	Firmicutes;D_2__Clostridia;D_3__Clostridiales;D_4__Ruminococcaceae;D_5__Ruminiclostridium 5;D_6__uncultured bacterium;D_7__;D_8__;D_9__;D_10__;D_11__;D_12__;D_13__;D_14__	1.30(0.37,4.56)	0.69(0.19,2.45)	0.53(0.16,1.75)	1.19(0.35,4.02)	0.91(0.27,3.08)	0.77(0.25,2.37)			
ASV_265	Firmicutes;D_2__Clostridia;D_3__Clostridiales;D_4__Lachnospiraceae;D_5__[Ruminococcus] torques group	5.40(0.56,52.49)	4.68(0.45,48.63)	0.87(0.09,7.98)						
ASV_268	Proteobacteria;D_2__Gammaproteobacteria;D_3__Betaproteobacteriales;D_4__Burkholderiaceae;D_5__Parasutterella	0.83(0.23,3.00)	1.39(0.37,5.25)	1.68(0.48,5.86)	0.57(0.15,2.16)	0.94(0.25,3.56)	1.65(0.48,5.74)			
ASV_270	Bacteroidetes;D_2__Bacteroidia;D_3__Bacteroidales;D_4__Bacteroidaceae;D_5__Bacteroides;D_6__Bacteroides uniformis;D_7__;D_8__;D_9__;D_10__;D_11__;D_12__;D_13__;D_14__	1.41(0.30,6.77)	1.70(0.34,8.53)	1.20(0.26,5.47)						
ASV_274	Firmicutes;D_2__Clostridia;D_3__Clostridiales;D_4__Christensenellaceae;D_5__Christensenellaceae R-7 group	0.27(0.05,1.53)	0.14(0.02,0.86);p=0.03335	0.53(0.10,2.86)	0.24(0.04,1.47)	0.13(0.02,0.81);p=0.0283	0.55(0.10,2.98)		↓	
ASV_277	Firmicutes;D_2__Clostridia;D_3__Clostridiales;D_4__Ruminococcaceae;D_5__Faecalibacterium;D_6__metagenome;D_7__;D_8__;D_9__;D_10__;D_11__;D_12__;D_13__;D_14__	0.58(0.12,2.68)	0.42(0.09,2.06)	0.73(0.17,3.25)	0.64(0.13,3.22)	0.46(0.09,2.29)	0.71(0.16,3.20)			
ASV_278	Bacteroidetes;D_2__Bacteroidia;D_3__Bacteroidales;D_4__Bacteroidaceae;D_5__Bacteroides;D_6__Bacteroides stercoris ATCC 43183;D_7__;D_8__;D_9__;D_10__;D_11__;D_12__;D_13__;D_14__	0.75(0.10,5.65)	0.47(0.06,3.78)	0.63(0.09,4.48)	1.20(0.14,9.99)	0.51(0.06,4.26)	0.43(0.06,3.08)			
ASV_280	Firmicutes;D_2__Clostridia;D_3__Clostridiales;D_4__Ruminococcaceae;D_5__uncultured;D_6__gut metagenome;D_7__;D_8__;D_9__;D_10__;D_11__;D_12__;D_13__;D_14__				0.46(0.02,11.22)	0.53(0.02,12.73)	1.14(0.06,22.17)			
ASV_281	Firmicutes;D_2__Clostridia;D_3__Clostridiales;D_4__Lachnospiraceae;D_5__Lachnospiraceae ND3007 group;D_6__metagenome;D_7__;D_8__;D_9__;D_10__;D_11__;D_12__;D_13__;D_14__	0.43(0.17,1.09)	0.47(0.18,1.21)	1.08(0.44,2.66)	0.40(0.15,1.06)	0.44(0.17,1.17)	1.11(0.45,2.73)			
ASV_283	Firmicutes;D_2__Clostridia;D_3__Clostridiales;D_4__Ruminococcaceae;D_5__Ruminococcaceae UCG-002	1.14(0.32,4.03)	0.53(0.14,1.94)	0.46(0.14,1.57)	2.06(0.55,7.62)	0.60(0.16,2.23)	0.29(0.09,0.99);p=0.04902			↓
ASV_287	Bacteroidetes;D_2__Bacteroidia;D_3__Bacteroidales;D_4__Tannerellaceae;D_5__Parabacteroides;D_6__Parabacteroides distasonis;D_7__;D_8__;D_9__;D_10__;D_11__;D_12__;D_13__;D_14__	0.20(0.03,1.18)	0.39(0.06,2.48)	1.99(0.35,11.20)						

ASV _288	Firmicutes;D_2__Clostridia;D_3__Clostridiales;D_4__Lachnospiraceae;D_5__[Eubacterium] hallii group	0.66(0.36,1.22)	0.56(0.30,1.05)	0.84(0.47,1.53)	0.59(0.31,1.11)	0.50(0.27,0.95); p=0.03475	0.86(0.47,1.56)		↓	
ASV _289	Bacteroidetes;D_2__Bacteroidia;D_3__Bacteroidales;D_4__Bacteroidaceae;D_5__Bacteroides	0.40(0.08,2.14)	1.62(0.29,8.99)	4.00(0.80,20.06)	0.23(0.04,1.26)	1.05(0.19,5.85)	4.64(0.94,23.06)			
ASV _290	Firmicutes;D_2__Clostridia;D_3__Clostridiales;D_4__Ruminococcaceae;D_5__Ruminiclostridium 6	0.16(0.01,2.29)	0.34(0.02,5.32)	2.14(0.16,27.72)						
ASV _291	Firmicutes;D_2__Clostridia;D_3__Clostridiales;D_4__Lachnospiraceae;D_5__Lachnospira	0.51(0.15,1.74)	0.42(0.12,1.50)	0.83(0.25,2.73)	0.57(0.16,2.06)	0.48(0.13,1.75)	0.85(0.26,2.83)			
ASV _292	Firmicutes;D_2__Clostridia;D_3__Clostridiales;D_4__Ruminococcaceae;D_5__Butyricicoccus	1.24(0.52,2.96)	0.86(0.35,2.10)	0.69(0.30,1.60)	1.25(0.50,3.09)	0.84(0.34,2.07)	0.67(0.29,1.56)			
ASV _293	Bacteroidetes;D_2__Bacteroidia;D_3__Bacteroidales;D_4__Rikenellaceae;D_5__Alistipes	0.20(0.02,1.90)	0.22(0.02,2.18)	1.08(0.13,9.19)	0.30(0.03,2.87)	0.27(0.03,2.50)	0.87(0.11,6.73)			
ASV _295	Firmicutes;D_2__Clostridia;D_3__Clostridiales;D_4__Lachnospiraceae;D_5__[Eubacterium] xylanophilum group				0.05(0.00,0.64); p=0.02143	0.03(0.00,0.38); p=0.00653	0.59(0.06,6.08)	↓	↓	
ASV _296	Firmicutes;D_2__Clostridia;D_3__Clostridiales;D_4__Lachnospiraceae	0.44(0.19,1.04)	0.58(0.24,1.39)	1.30(0.57,2.96)	0.41(0.17,1.00)	0.61(0.25,1.48)	1.48(0.65,3.39)			
ASV _297	Bacteroidetes;D_2__Bacteroidia;D_3__Bacteroidales;D_4__Tannerellaceae;D_5__Parabacteroides;D _6__Parabacteroides distasonis;D_7__;D_8__;D_9__;D_10__;D_11__;D_12__;D_13__;D_14__	0.35(0.04,3.31)	0.25(0.02,2.49)	0.70(0.08,6.16)	0.07(0.01,0.67); p=0.02161	0.22(0.02,2.26)	3.39(0.39,29.65)	↓		
ASV _298	Firmicutes;D_2__Clostridia;D_3__Clostridiales;D_4__Ruminococcaceae;D_5__Oscillibacter;D_6__ uncultured organism;D_7__;D_8__;D_9__;D_10__;D_11__;D_12__;D_13__;D_14__	1.20(0.60,2.39)	1.31(0.64,2.66)	1.09(0.56,2.13)	1.10(0.53,2.26)	1.26(0.61,2.59)	1.15(0.59,2.25)			
ASV _299	Firmicutes;D_2__Clostridia;D_3__Clostridiales;D_4__Ruminococcaceae;D_5__Ruminococcaceae UCG-005	0.62(0.09,4.32)	0.36(0.05,2.62)	0.57(0.09,3.74)	0.68(0.09,5.10)	0.38(0.05,2.86)	0.56(0.09,3.68)			
ASV _301	Firmicutes;D_2__Clostridia;D_3__Clostridiales;D_4__Lachnospiraceae;D_5__Lachnospiraceae UCG-008;D_6__uncultured organism;D_7__;D_8__;D_9__;D_10__;D_11__;D_12__;D_13__;D_14__	0.70(0.23,2.11)	0.46(0.15,1.43)	0.66(0.23,1.90)	0.57(0.18,1.78)	0.40(0.13,1.26)	0.71(0.24,2.06)			
ASV _302	Firmicutes;D_2__Clostridia;D_3__Clostridiales;D_4__Lachnospiraceae;D_5__uncultured	0.37(0.14,1.00) ;p=0.04897	0.34(0.12,0.95) ;p=0.03868	0.92(0.35,2.41)	0.36(0.13,1.03)	0.34(0.12,0.96); p=0.04102	0.93(0.35,2.45)		↓	
ASV _304	Firmicutes;D_2__Clostridia;D_3__Clostridiales;D_4__Lachnospiraceae;D_5__Lachnospiraceae UCG-010;D_6__uncultured organism;D_7__;D_8__;D_9__;D_10__;D_11__;D_12__;D_13__;D_14__	0.11(0.01,2.11)	0.12(0.01,2.61)	1.13(0.07,19.56)	0.04(0.00,0.93); p=0.04522	0.03(0.00,0.79); p=0.03557	0.87(0.05,14.08)	↓	↓	
ASV _306	Firmicutes;D_2__Clostridia;D_3__Clostridiales;D_4__Lachnospiraceae;D_5__Lachnospiraceae UCG-001	0.63(0.12,3.39)	0.80(0.14,4.50)	1.26(0.25,6.42)						
ASV _309	Firmicutes;D_2__Clostridia;D_3__Clostridiales;D_4__Lachnospiraceae;D_5__Lachnoclostridium;D _6__uncultured Firmicutes bacterium;D_7__;D_8__;D_9__;D_10__;D_11__;D_12__;D_13__;D_14__	0.42(0.16,1.14)	0.70(0.25,1.93)	1.65(0.63,4.32)						
ASV _313	Firmicutes;D_2__Clostridia;D_3__Clostridiales;D_4__Ruminococcaceae;D_5__Ruminiclostridium 9	0.48(0.12,1.93)	0.31(0.07,1.32)	0.66(0.17,2.54)	0.37(0.09,1.59)	0.25(0.06,1.07)	0.67(0.17,2.61)			
ASV _314	Firmicutes;D_2__Clostridia;D_3__Clostridiales;D_4__Lachnospiraceae;D_5__Lachnospiraceae UCG-004	0.49(0.15,1.69)	0.21(0.06,0.74) ;p=0.01512	0.42(0.13,1.38)	0.41(0.11,1.48)	0.19(0.05,0.69); p=0.01163	0.47(0.14,1.54)		↓	
ASV _315	Firmicutes;D_2__Clostridia;D_3__Clostridiales;D_4__Lachnospiraceae;D_5__Lachnospiraceae UCG-004	0.51(0.19,1.36)	0.67(0.24,1.83)	1.30(0.51,3.35)	0.32(0.12,0.89); p=0.02876	0.44(0.16,1.21)	1.36(0.53,3.50)	↓		
ASV _317	Firmicutes;D_2__Clostridia;D_3__Clostridiales;D_4__Ruminococcaceae;D_5__[Eubacterium] coprostanoligenes group	0.40(0.04,4.18)	0.36(0.03,4.03)	0.90(0.09,8.70)	0.31(0.03,3.44)	0.15(0.01,1.65)	0.48(0.05,4.50)			
ASV _318	Firmicutes;D_2__Clostridia;D_3__Clostridiales;D_4__Lachnospiraceae	0.88(0.32,2.41)	0.96(0.34,2.72)	1.10(0.41,2.92)	0.58(0.21,1.65)	0.89(0.31,2.51)	1.52(0.58,4.01)			
ASV _322	Firmicutes;D_2__Clostridia;D_3__Clostridiales;D_4__Ruminococcaceae;D_5__Ruminococcaceae UCG-005;D_6__uncultured organism;D_7__;D_8__;D_9__;D_10__;D_11__;D_12__;D_13__;D_14__	0.24(0.06,1.03)	0.10(0.02,0.46) ;p=0.00307	0.43(0.11,1.75)	0.20(0.05,0.92); p=0.03814	0.11(0.02,0.50); p=0.00414	0.55(0.14,2.19)	↓	↓	
ASV _323	Firmicutes;D_2__Clostridia;D_3__Clostridiales;D_4__Ruminococcaceae;D_5__Ruminococcaceae UCG-010;D_6__metagenome;D_7__;D_8__;D_9__;D_10__;D_11__;D_12__;D_13__;D_14__	0.11(0.01,1.05)	0.17(0.02,1.84)	1.64(0.18,14.70)						
ASV _325	Firmicutes;D_2__Clostridia;D_3__Clostridiales;D_4__Clostridiales vadinBB60 group;D_5__gut metagenome;D_6__;D_7__;D_8__;D_9__;D_10__;D_11__;D_12__;D_13__;D_14__				0.19(0.01,2.82)	0.02(0.00,0.33); p=0.00561	0.12(0.01,1.35)		↓	
ASV _327	Firmicutes;D_2__Clostridia;D_3__Clostridiales;D_4__Lachnospiraceae;D_5__GCA-900066575	0.71(0.23,2.24)	0.61(0.19,1.98)	0.85(0.28,2.59)	0.60(0.18,2.01)	0.60(0.18,1.97)	0.98(0.32,2.99)			
ASV _331	Firmicutes;D_2__Clostridia;D_3__Clostridiales;D_4__Ruminococcaceae;D_5__Ruminococcaceae UCG-005	0.66(0.04,10.5 3)	5.23(0.24,113. 88)	7.88(0.43,143.5 5)	0.43(0.02,7.45)	3.84(0.19,79.21)	8.91(0.46,171.84)			
ASV _334	Firmicutes;D_2__Clostridia;D_3__Clostridiales;D_4__Lachnospiraceae;D_5__uncultured;D_6__unc ultured bacterium adhufec382;D_7__;D_8__;D_9__;D_10__;D_11__;D_12__;D_13__;D_14__	1.05(0.37,2.96)	0.80(0.27,2.30)	0.76(0.28,2.05)	1.07(0.36,3.17)	0.83(0.28,2.46)	0.78(0.28,2.14)			
ASV _335	Firmicutes;D_2__Clostridia;D_3__Clostridiales;D_4__Christensenellaceae;D_5__uncultured;D_6__ uncultured bacterium;D_7__;D_8__;D_9__;D_10__;D_11__;D_12__;D_13__;D_14__				1.34(0.09,20.63)	0.52(0.03,7.91)	0.39(0.03,4.93)			

ASV _336	Firmicutes;D_2__Clostridia;D_3__Clostridiales;D_4__Family XIII;D_5__Family XIII UCG-001;D_6__uncultured bacterium;D_7__;D_8__;D_9__;D_10__;D_11__;D_12__;D_13__;D_14__	0.02(0.00,0.98);p=0.04853	0.04(0.00,1.79)	1.70(0.10,28.00)						
ASV _340	Firmicutes;D_2__Clostridia;D_3__Clostridiales;D_4__Ruminococcaceae;D_5__Flavonifractor;D_6__uncultured bacterium;D_7__;D_8__;D_9__;D_10__;D_11__;D_12__;D_13__;D_14__	1.62(0.44,5.95)	1.29(0.34,4.92)	0.80(0.23,2.81)	1.51(0.39,5.83)	1.39(0.36,5.37)	0.92(0.26,3.26)			
ASV _341	Tenericutes;D_2__Mollicutes;D_3__Izimaplasmatales;D_4__uncultured organism;D_5__;D_6__;D_7__;D_8__;D_9__;D_10__;D_11__;D_12__;D_13__;D_14__	0.25(0.01,9.25)	0.09(0.00,3.60)	0.36(0.01,9.96)						
ASV _344	Firmicutes;D_2__Clostridia;D_3__Clostridiales;D_4__Defluviitaleaceae;D_5__Defluviitaleaceae UCG-011;D_6__uncultured bacterium;D_7__;D_8__;D_9__;D_10__;D_11__;D_12__;D_13__;D_14__	1.21(0.10,15.04)	1.48(0.11,19.91)	1.22(0.11,14.11)	1.38(0.10,18.76)	2.61(0.19,35.84)	1.90(0.16,21.96)			
ASV _345	Firmicutes;D_2__Clostridia;D_3__Clostridiales;D_4__Lachnospiraceae;D_5__Lachnospira	0.60(0.15,2.49)	0.15(0.04,0.66);p=0.01197	0.26(0.07,1.00)	0.62(0.14,2.73)	0.16(0.04,0.70); p=0.01518	0.26(0.06,1.03)		↓	
ASV _348	Firmicutes;D_2__Clostridia;D_3__Clostridiales;D_4__Lachnospiraceae;D_5__Moryella;D_6__human gut metagenome;D_7__;D_8__;D_9__;D_10__;D_11__;D_12__;D_13__;D_14__	0.74(0.26,2.11)	0.56(0.19,1.65)	0.76(0.28,2.08)	0.71(0.24,2.06)	0.66(0.23,1.91)	0.93(0.34,2.51)			
ASV _349	Firmicutes;D_2__Clostridia;D_3__Clostridiales;D_4__Ruminococcaceae;D_5__Flavonifractor	1.15(0.62,2.12)	1.99(1.05,3.75);p=0.03376	1.73(0.95,3.15)	1.08(0.57,2.04)	1.68(0.89,3.19)	1.56(0.86,2.83)			
ASV _351	Firmicutes;D_2__Clostridia;D_3__Clostridiales;D_4__Ruminococcaceae;D_5__uncultured;D_6__Clostridium phoceensis;D_7__;D_8__;D_9__;D_10__;D_11__;D_12__;D_13__;D_14__	0.00(0.00,Inf)	0.00(0.00,Inf)	0.68(0.05,8.59)	0.00(0.00,Inf)	0.00(0.00,Inf)	0.39(0.03,5.06)			
ASV _353	Firmicutes;D_2__Clostridia;D_3__Clostridiales;D_4__Ruminococcaceae;D_5__Ruminococcaceae UCG-010	0.53(0.12,2.25)	0.68(0.15,3.02)	1.28(0.32,5.19)	0.63(0.14,2.84)	0.90(0.20,4.03)	1.42(0.35,5.75)			
ASV _354	Firmicutes;D_2__Clostridia;D_3__Clostridiales;D_4__Lachnospiraceae;D_5__Lachnospiraceae FCS020 group	0.57(0.19,1.73)	0.86(0.27,2.71)	1.50(0.52,4.36)	0.59(0.18,1.87)	0.84(0.26,2.68)	1.43(0.49,4.19)			
ASV _355	Firmicutes;D_2__Clostridia;D_3__Clostridiales;D_4__Ruminococcaceae;D_5__Ruminococcaceae UCG-009;D_6__uncultured bacterium;D_7__;D_8__;D_9__;D_10__;D_11__;D_12__;D_13__;D_14__	0.31(0.04,2.64)	0.68(0.07,6.30)	2.21(0.28,17.57)	0.32(0.04,2.85)	0.37(0.04,3.32)	1.15(0.16,8.37)			
ASV _356	Firmicutes;D_2__Clostridia;D_3__Clostridiales;D_4__Lachnospiraceae;D_5__uncultured;D_6__uncultured organism;D_7__;D_8__;D_9__;D_10__;D_11__;D_12__;D_13__;D_14__	0.15(0.01,2.91)	0.12(0.01,2.47)	0.79(0.05,11.89)	0.15(0.01,3.35)	0.13(0.01,2.77)	0.84(0.05,12.96)			
ASV _357	Firmicutes;D_2__Clostridia;D_3__Clostridiales;D_4__Ruminococcaceae;D_5__Oscillibacter;D_6__human gut metagenome;D_7__;D_8__;D_9__;D_10__;D_11__;D_12__;D_13__;D_14__	0.56(0.25,1.27)	0.64(0.28,1.47)	1.13(0.52,2.49)	0.53(0.23,1.25)	0.61(0.26,1.43)	1.15(0.52,2.53)			
ASV _358	Actinobacteria;D_2__Coriobacteriia;D_3__Coriobacteriales;D_4__Atopobiaceae;D_5__uncultured;D_6__uncultured bacterium;D_7__;D_8__;D_9__;D_10__;D_11__;D_12__;D_13__;D_14__	0.31(0.03,3.34)	0.23(0.02,2.65)	0.74(0.08,7.22)	0.27(0.02,3.30)	0.24(0.02,2.88)	0.88(0.09,8.72)			
ASV _360	Firmicutes;D_2__Clostridia;D_3__Clostridiales;D_4__Ruminococcaceae;D_5__Ruminiclostridium 9;D_6__uncultured organism;D_7__;D_8__;D_9__;D_10__;D_11__;D_12__;D_13__;D_14__	1.23(0.48,3.14)	0.65(0.25,1.69)	0.53(0.21,1.30)	1.17(0.44,3.14)	0.65(0.24,1.71)	0.55(0.22,1.37)			
ASV _364	Firmicutes;D_2__Clostridia;D_3__Clostridiales;D_4__Lachnospiraceae;D_5__Roseburia;D_6__gut metagenome;D_7__;D_8__;D_9__;D_10__;D_11__;D_12__;D_13__;D_14__	0.39(0.02,6.22)	3.09(0.16,58.34)	7.90(0.51,123.37)						
ASV _365	Firmicutes;D_2__Clostridia;D_3__Clostridiales;D_4__Ruminococcaceae;D_5__Intestinimonas	0.12(0.01,1.08)	0.76(0.08,7.45)	6.10(0.79,47.36)	0.45(0.06,3.66)	1.35(0.15,11.76)	2.96(0.42,20.78)			
ASV _366	Proteobacteria;D_2__Gammaproteobacteria;D_3__Pasteurellales;D_4__Pasteurellaceae;D_5__Haemophilus	1.11(0.40,3.05)	1.16(0.41,3.31)	1.05(0.39,2.81)	0.84(0.29,2.41)	0.94(0.33,2.70)	1.12(0.42,3.01)			
ASV _368	Firmicutes;D_2__Clostridia;D_3__Clostridiales;D_4__Ruminococcaceae;D_5__Ruminococcaceae UCG-013;D_6__gut metagenome;D_7__;D_8__;D_9__;D_10__;D_11__;D_12__;D_13__;D_14__	0.91(0.23,3.64)	0.59(0.14,2.45)	0.65(0.17,2.48)	0.98(0.23,4.22)	0.55(0.13,2.37)	0.56(0.14,2.19)			
ASV _369	Firmicutes;D_2__Clostridia;D_3__Clostridiales;D_4__Ruminococcaceae;D_5__Ruminococcaceae UCG-003;D_6__uncultured bacterium;D_7__;D_8__;D_9__;D_10__;D_11__;D_12__;D_13__;D_14__	0.37(0.09,1.52)	0.26(0.06,1.10)	0.70(0.18,2.74)	0.34(0.08,1.51)	0.21(0.05,0.90); p=0.03608	0.60(0.15,2.38)		↓	
ASV _370	Firmicutes;D_2__Bacilli;D_3__Lactobacillales;D_4__Lactobacillaceae;D_5__Lactobacillus;D_6__Lactobacillus fermentum;D_7__;D_8__;D_9__;D_10__;D_11__;D_12__;D_13__;D_14__	1.33(0.07,26.02)	1.51(0.07,32.34)	1.14(0.06,20.30)						
ASV _371	Firmicutes;D_2__Clostridia;D_3__Clostridiales;D_4__Ruminococcaceae;D_5__uncultured;D_6__Clostridium phoceensis;D_7__;D_8__;D_9__;D_10__;D_11__;D_12__;D_13__;D_14__	1.16(0.49,2.77)	1.97(0.80,4.84)	1.69(0.73,3.94)	1.12(0.45,2.77)	1.96(0.79,4.85)	1.75(0.75,4.08)			
ASV _373	Firmicutes;D_2__Clostridia;D_3__Clostridiales;D_4__Ruminococcaceae;D_5__Oscillospira;D_6__uncultured bacterium;D_7__;D_8__;D_9__;D_10__;D_11__;D_12__;D_13__;D_14__	0.76(0.03,23.02)	3.52(0.08,158.64)	4.64(0.13,166.18)	0.41(0.01,15.55)	3.35(0.06,196.33)	8.27(0.19,367.21)			
ASV _374	Firmicutes;D_2__Clostridia;D_3__Clostridiales;D_4__Clostridiaceae 1;D_5__Clostridium sensu stricto 1	1.43(0.31,6.53)	0.37(0.08,1.76)	0.26(0.06,1.12)						
ASV _376	Firmicutes;D_2__Clostridia;D_3__Clostridiales;D_4__Clostridiales vadinBB60 group;D_5__gut metagenome;D_6__;D_7__;D_8__;D_9__;D_10__;D_11__;D_12__;D_13__;D_14__	0.78(0.04,15.48)	0.73(0.03,15.93)	0.94(0.05,16.84)						
ASV _377	Firmicutes;D_2__Clostridia;D_3__Clostridiales;D_4__Ruminococcaceae;D_5__Phocea;D_6__uncultured bacterium;D_7__;D_8__;D_9__;D_10__;D_11__;D_12__;D_13__;D_14__	0.75(0.29,1.94)	0.76(0.29,2.04)	1.02(0.41,2.54)	0.65(0.24,1.77)	0.73(0.27,1.99)	1.13(0.45,2.82)			
ASV _378	Proteobacteria;D_2__Gammaproteobacteria;D_3__Enterobacteriales;D_4__Enterobacteriaceae;D_5__Escherichia-Shigella	1.82(0.58,5.70)	0.98(0.30,3.17)	0.54(0.18,1.62)	1.30(0.40,4.19)	0.89(0.28,2.87)	0.69(0.23,2.04)			
ASV _380	Firmicutes;D_2__Clostridia;D_3__Clostridiales;D_4__Ruminococcaceae;D_5__Oscillibacter;D_6__Oscillibacter sp. PC13;D_7__;D_8__;D_9__;D_10__;D_11__;D_12__;D_13__;D_14__	0.14(0.01,2.19)	0.11(0.01,1.84)	0.79(0.07,8.85)						

ASV_383	Firmicutes;D_2__Clostridia;D_3__Clostridiales;D_4__Clostridiales vadinBB60 group;D_5__uncultured bacterium;D_6__;D_7__;D_8__;D_9__;D_10__;D_11__;D_12__;D_13__;D_14__				1.30(0.18,9.18)	0.67(0.10,4.65)	0.51(0.08,3.12)			
ASV_384	Firmicutes;D_2__Clostridia;D_3__Clostridiales;D_4__Ruminococcaceae;D_5__Ruminococcaceae UCG-005	0.85(0.11,6.49)	0.41(0.05,3.25)	0.48(0.07,3.33)	0.66(0.08,5.59)	0.41(0.05,3.41)	0.62(0.09,4.34)			
ASV_386	Firmicutes;D_2__Clostridia;D_3__Clostridiales;D_4__Lachnospiraceae	0.72(0.05,10.25)	0.92(0.06,14.26)	1.27(0.10,16.22)	0.70(0.04,11.27)	0.95(0.06,15.66)	1.36(0.10,17.59)			
ASV_388	Firmicutes;D_2__Clostridia;D_3__Clostridiales;D_4__Ruminococcaceae;D_5__Oscillospira	0.74(0.26,2.10)	0.80(0.27,2.35)	1.09(0.40,2.98)	0.63(0.21,1.85)	0.70(0.24,2.07)	1.12(0.41,3.07)			
ASV_392	Actinobacteria;D_2__Coriobacteriia;D_3__Coriobacteriales;D_4__Eggerthellaceae;D_5__Eggerthella	1.11(0.56,2.21)	1.33(0.65,2.70)	1.20(0.61,2.33)	1.07(0.52,2.19)	1.18(0.57,2.42)	1.11(0.56,2.17)			
ASV_393	Firmicutes;D_2__Clostridia;D_3__Clostridiales;D_4__Ruminococcaceae;D_5__Ruminococcaceae UCG-005;D_6__uncultured organism;D_7__;D_8__;D_9__;D_10__;D_11__;D_12__;D_13__;D_14__	0.88(0.15,5.06)	0.43(0.07,2.61)	0.49(0.09,2.65)	0.55(0.09,3.29)	0.39(0.06,2.31)	0.71(0.13,3.72)			
ASV_397	Firmicutes;D_2__Clostridia;D_3__Clostridiales;D_4__Family XIII;D_5__Family XIII AD3011 group;D_6__uncultured organism;D_7__;D_8__;D_9__;D_10__;D_11__;D_12__;D_13__;D_14__	0.43(0.11,1.65)	0.44(0.11,1.73)	1.01(0.28,3.63)	0.32(0.08,1.26)	0.41(0.10,1.60)	1.29(0.37,4.48)			
ASV_401	Firmicutes;D_2__Clostridia;D_3__Clostridiales;D_4__Ruminococcaceae;D_5__uncultured;D_6__uncultured Ruminococcus sp.;D_7__;D_8__;D_9__;D_10__;D_11__;D_12__;D_13__;D_14__	0.79(0.26,2.39)	0.74(0.24,2.32)	0.94(0.32,2.73)	1.00(0.32,3.13)	0.96(0.31,3.02)	0.97(0.33,2.80)			
ASV_402	Firmicutes;D_2__Clostridia;D_3__Clostridiales;D_4__Ruminococcaceae;D_5__Ruminiclostridium 9;D_6__uncultured bacterium;D_7__;D_8__;D_9__;D_10__;D_11__;D_12__;D_13__;D_14__	0.00(0.00,Inf)	0.00(0.00,Inf)	5.16(0.20,131.09)						
ASV_405	Actinobacteria;D_2__Actinobacteria;D_3__Actinomycetales;D_4__Actinomycetaceae;D_5__Actinomyces	3.07(0.74,12.72)	0.34(0.09,1.35)	0.11(0.03,0.43); p=0.0015	2.88(0.65,12.71)	0.26(0.06,1.02)	0.09(0.02,0.35);p=0.00049			↓
ASV_406	Firmicutes;D_2__Clostridia;D_3__Clostridiales;D_4__Ruminococcaceae	0.00(0.00,Inf)	0.00(0.00,Inf)	0.17(0.02,1.90)						
ASV_410	Firmicutes;D_2__Clostridia;D_3__Clostridiales;D_4__Lachnospiraceae;D_5__Anaerosporobacter;D_6__uncultured organism;D_7__;D_8__;D_9__;D_10__;D_11__;D_12__;D_13__;D_14__	0.05(0.00,0.62);p=0.02068	0.02(0.00,0.22);p=0.00223	0.33(0.03,4.01)	0.01(0.00,0.17); p=0.00153	0.00(0.00,0.02); p=0;q=0.00275	0.11(0.01,1.28)	↓	↓*	
ASV_411	Firmicutes;D_2__Clostridia;D_3__Clostridiales;D_4__Ruminococcaceae;D_5__Ruminiclostridium 9;D_6__uncultured bacterium;D_7__;D_8__;D_9__;D_10__;D_11__;D_12__;D_13__;D_14__	0.94(0.06,15.17)	0.96(0.05,16.99)	1.03(0.07,15.22)						
ASV_413	Firmicutes;D_2__Clostridia;D_3__Clostridiales;D_4__Lachnospiraceae;D_5__Lachnospiraceae UCG-010;D_6__uncultured bacterium;D_7__;D_8__;D_9__;D_10__;D_11__;D_12__;D_13__;D_14__	1.36(0.35,5.21)	1.02(0.26,4.07)	0.75(0.20,2.77)	0.90(0.22,3.65)	1.00(0.25,4.01)	1.10(0.30,4.03)			
ASV_414	Firmicutes;D_2__Clostridia;D_3__Clostridiales;D_4__Ruminococcaceae;D_5__GCA-900066225;D_6__uncultured bacterium;D_7__;D_8__;D_9__;D_10__;D_11__;D_12__;D_13__;D_14__	0.96(0.09,10.35)	2.08(0.17,24.80)	2.17(0.21,22.36)	0.46(0.04,5.93)	0.37(0.03,4.42)	0.80(0.08,8.01)			
ASV_415	Firmicutes;D_2__Clostridia;D_3__Clostridiales;D_4__Ruminococcaceae;D_5__Pseudoflavonifractor;D_6__uncultured bacterium;D_7__;D_8__;D_9__;D_10__;D_11__;D_12__;D_13__;D_14__	1.15(0.19,6.87)	2.07(0.32,13.47)	1.80(0.31,10.58)	1.11(0.17,7.29)	2.17(0.32,14.62)	1.95(0.33,11.62)			
ASV_418	Firmicutes;D_2__Clostridia;D_3__Clostridiales;D_4__Christensenellaceae;D_5__uncultured;D_6__uncultured bacterium;D_7__;D_8__;D_9__;D_10__;D_11__;D_12__;D_13__;D_14__	0.31(0.01,9.38)	0.61(0.02,20.29)	1.93(0.07,50.27)						
ASV_420	Firmicutes;D_2__Clostridia;D_3__Clostridiales;D_4__Ruminococcaceae;D_5__Fournierella;D_6__uncultured organism;D_7__;D_8__;D_9__;D_10__;D_11__;D_12__;D_13__;D_14__	1.72(0.49,6.07)	0.42(0.12,1.46)	0.24(0.07,0.81); p=0.02068	1.77(0.47,6.67)	0.35(0.10,1.23)	0.20(0.06,0.66);p=0.00837			↓
ASV_421	Firmicutes;D_2__Clostridia;D_3__Clostridiales;D_4__Lachnospiraceae;D_5__Agathobacter	1.40(0.05,35.67)	0.09(0.01,1.45)	0.07(0.00,0.96); p=0.04658	2.27(0.09,59.33)	0.17(0.01,2.29)	0.08(0.01,1.10)			
ASV_422	Firmicutes;D_2__Clostridia;D_3__Clostridiales;D_4__Ruminococcaceae;D_5__Ruminococcaceae UCG-007	2.90(0.08,108.90)	1.83(0.05,71.74)	0.63(0.02,22.54)						
ASV_423	Firmicutes;D_2__Clostridia;D_3__Clostridiales;D_4__Lachnospiraceae;D_5__Lachnoclostridium	1.70(0.52,5.51)	1.08(0.32,3.58)	0.63(0.20,1.98)	1.26(0.37,4.29)	1.02(0.30,3.42)	0.81(0.26,2.51)			
ASV_424	Firmicutes;D_2__Clostridia;D_3__Clostridiales;D_4__Lachnospiraceae	0.33(0.07,1.63)	0.41(0.08,2.13)	1.25(0.27,5.85)	0.38(0.07,2.03)	0.34(0.06,1.77)	0.88(0.19,4.09)			
ASV_426	Firmicutes;D_2__Clostridia;D_3__Clostridiales;D_4__Lachnospiraceae;D_5__[Ruminococcus] torques group	0.97(0.26,3.65)	1.76(0.44,6.96)	1.82(0.50,6.62)	1.10(0.27,4.39)	1.70(0.42,6.83)	1.55(0.42,5.67)			
ASV_429	Firmicutes;D_2__Clostridia;D_3__Clostridiales;D_4__Ruminococcaceae;D_5__uncultured;D_6__uncultured Clostridium sp.;D_7__;D_8__;D_9__;D_10__;D_11__;D_12__;D_13__;D_14__	0.69(0.09,5.30)	0.38(0.05,3.06)	0.55(0.08,3.86)	0.46(0.05,4.08)	0.20(0.02,1.71)	0.43(0.06,3.06)			
ASV_430	Firmicutes;D_2__Clostridia;D_3__Clostridiales;D_4__Ruminococcaceae;D_5__Ruminococcaceae UCG-013;D_6__uncultured Clostridiaceae bacterium;D_7__;D_8__;D_9__;D_10__;D_11__;D_12__;D_13__;D_14__	0.15(0.01,2.87)	0.10(0.00,1.89)	0.62(0.05,7.49)						
ASV_431	Firmicutes;D_2__Clostridia;D_3__Clostridiales;D_4__Ruminococcaceae	1.34(0.40,4.53)	0.93(0.27,3.24)	0.69(0.21,2.24)	1.74(0.49,6.17)	1.24(0.35,4.36)	0.71(0.22,2.31)			
ASV_432	Firmicutes;D_2__Erysipelotrichia;D_3__Erysipelotrichales;D_4__Erysipelotrichaceae;D_5__Holdemania	1.23(0.27,5.67)	1.29(0.27,6.24)	1.05(0.24,4.62)	1.76(0.36,8.67)	1.40(0.29,6.84)	0.79(0.18,3.50)			

ASV_437	Firmicutes;D_2__Clostridia;D_3__Clostridiales;D_4__Ruminococcaceae;D_5__Candidatus Soleaferrea;D_6__uncultured bacterium;D_7__;D_8__;D_9__;D_10__;D_11__;D_12__;D_13__;D_14__	0.71(0.16,3.22)	0.82(0.17,3.93)	1.16(0.27,4.94)	0.76(0.16,3.68)	0.89(0.18,4.36)	1.17(0.27,5.00)			
ASV_439	Firmicutes;D_2__Clostridia;D_3__Clostridiales;D_4__Peptococcaceae;D_5__uncultured;D_6__metagenome;D_7__;D_8__;D_9__;D_10__;D_11__;D_12__;D_13__;D_14__	0.55(0.02,15.83)	0.33(0.01,9.95)	0.60(0.03,13.08)						
ASV_445	Proteobacteria;D_2__Gammaproteobacteria;D_3__Betaproteobacteriales;D_4__Burkholderiaceae;D_5__Sutterella	0.16(0.02,1.18)	0.09(0.01,0.69);p=0.02074	0.55(0.08,3.75)	0.16(0.02,1.31)	0.10(0.01,0.76); p=0.02689	0.59(0.08,4.06)		↓	
ASV_448	Actinobacteria;D_2__Actinobacteria;D_3__Bifidobacteriales;D_4__Bifidobacteriaceae;D_5__Bifidobacterium	1.59(0.52,4.88)	1.15(0.36,3.66)	0.73(0.24,2.15)	1.28(0.40,4.13)	1.00(0.31,3.23)	0.79(0.26,2.34)			
ASV_456	Firmicutes;D_2__Clostridia;D_3__Clostridiales;D_4__Peptostreptococcaceae;D_5__Intestinibacter	2.22(1.09,4.50);p=0.0278	1.31(0.63,2.71)	0.59(0.30,1.17)	2.45(1.17,5.11); p=0.0174	1.43(0.69,2.99)	0.59(0.29,1.16)	↑		
ASV_457	Firmicutes;D_2__Clostridia;D_3__Clostridiales;D_4__Lachnospiraceae;D_5__uncultured	0.36(0.08,1.64)	0.50(0.10,2.36)	1.37(0.33,5.67)	0.19(0.04,0.95); p=0.0436	0.43(0.09,2.16)	2.27(0.54,9.43)	↓		
ASV_460	Firmicutes;D_2__Clostridia;D_3__Clostridiales;D_4__Lachnospiraceae	0.83(0.21,3.22)	0.71(0.18,2.88)	0.86(0.23,3.19)						
ASV_462	Firmicutes;D_2__Clostridia;D_3__Clostridiales;D_4__Lachnospiraceae;D_5__uncultured;D_6__uncultured bacterium adhufec382;D_7__;D_8__;D_9__;D_10__;D_11__;D_12__;D_13__;D_14__	0.05(0.01,0.30);p=0.00096	0.21(0.03,1.35)	4.24(0.91,19.79)	0.05(0.01,0.29); p=0.001	0.25(0.04,1.61)	5.59(1.21,25.87);p=0.02766	↓		↑
ASV_464	Firmicutes;D_2__Clostridia;D_3__Clostridiales;D_4__Lachnospiraceae;D_5__Agathobacter				0.17(0.02,1.47)	0.31(0.04,2.67)	1.86(0.26,13.32)			
ASV_465	Firmicutes;D_2__Bacilli;D_3__Lactobacillales;D_4__Lactobacillaceae;D_5__Lactobacillus	0.12(0.01,1.55)	1.08(0.08,14.83)	8.78(0.75,102.64)	0.06(0.00,0.94); p=0.04486	0.23(0.02,3.13)	3.52(0.32,39.21)	↓		
ASV_466	Firmicutes;D_2__Clostridia;D_3__Clostridiales;D_4__Lachnospiraceae;D_5__uncultured	0.00(0.00,Inf)	0.00(0.00,Inf)	0.02(0.00,0.74); p=0.03299						
ASV_467	Firmicutes;D_2__Bacilli;D_3__Lactobacillales;D_4__Lactobacillaceae;D_5__Lactobacillus	0.41(0.04,4.19)	1.88(0.17,20.90)	4.57(0.48,43.61)	0.34(0.03,3.54)	2.73(0.25,29.49)	7.92(0.88,71.01)			
ASV_471	Firmicutes;D_2__Bacilli;D_3__Lactobacillales;D_4__Streptococcaceae;D_5__Streptococcus	1.67(0.48,5.81)	1.38(0.38,4.98)	0.83(0.25,2.76)	2.71(0.79,9.36)	1.25(0.37,4.30)	0.46(0.15,1.46)			
ASV_472	Firmicutes;D_2__Erysipelotrichia;D_3__Erysipelotrichales;D_4__Erysipelotrichaceae;D_5__Dielma	0.49(0.14,1.63)	2.82(0.80,9.98)	5.82(1.78,19.04);p=0.00358	0.37(0.11,1.31)	1.30(0.37,4.57)	3.46(1.07,11.15);p=0.03753			↑
ASV_474	Firmicutes;D_2__Bacilli;D_3__Lactobacillales;D_4__Leuconostocaceae;D_5__Leuconostoc	0.24(0.01,7.16)	0.63(0.02,20.56)	2.59(0.10,67.95)						
ASV_475	Firmicutes;D_2__Negativicutes;D_3__Selenomonadales;D_4__Veillonellaceae;D_5__Veillonella	0.57(0.10,3.34)	0.19(0.03,1.12)	0.33(0.07,1.64)						
ASV_476	Cyanobacteria;D_2__Oxyphotobacteria;D_3__Chloroplast	6.40(0.38,107.68)	8.37(0.44,157.82)	1.31(0.08,22.21)						
ASV_477	Firmicutes;D_2__Clostridia;D_3__Clostridiales;D_4__Peptostreptococcaceae;D_5__Terrisporobacter;D_6__uncultured bacterium;D_7__;D_8__;D_9__;D_10__;D_11__;D_12__;D_13__;D_14__	2.34(0.74,7.35)	0.49(0.15,1.61)	0.21(0.07,0.64); p=0.006						
ASV_481	Proteobacteria;D_2__Alphaproteobacteria;D_3__Caulobacterales;D_4__Caulobacteraceae;D_5__Brevundimonas	10.38(0.85,126.98)	2.64(0.26,26.64)	0.25(0.02,3.12)						
ASV_482	Firmicutes;D_2__Negativicutes;D_3__Selenomonadales;D_4__Veillonellaceae;D_5__Megasphaera;D_6__unidentified;D_7__;D_8__;D_9__;D_10__;D_11__;D_12__;D_13__;D_14__	0.96(0.02,37.99)	0.61(0.01,25.49)	0.63(0.02,20.72)						
ASV_483	Firmicutes;D_2__Bacilli;D_3__Lactobacillales;D_4__Streptococcaceae;D_5__Lactococcus	0.07(0.00,3.70)	0.11(0.00,6.43)	1.55(0.04,62.90)						
ASV_484	Firmicutes;D_2__Bacilli;D_3__Lactobacillales;D_4__Lactobacillaceae;D_5__Pediococcus	0.20(0.01,5.18)	0.01(0.00,0.35);p=0.00969	0.06(0.00,1.24)						
ASV_485	Actinobacteria;D_2__Actinobacteria;D_3__Micrococcales;D_4__Micrococcaceae;D_5__Rothia;D_6__uncultured organism;D_7__;D_8__;D_9__;D_10__;D_11__;D_12__;D_13__;D_14__	1.79(0.36,8.83)	0.76(0.15,3.85)	0.42(0.09,1.98)	1.75(0.34,9.15)	0.96(0.19,4.95)	0.55(0.12,2.56)			
ASV_488	Proteobacteria;D_2__Gammaproteobacteria;D_3__Pasteurellales;D_4__Pasteurellaceae	4.18(0.38,46.36)	1.53(0.13,18.20)	0.37(0.04,3.76)	3.97(0.33,47.78)	0.92(0.08,10.99)	0.23(0.02,2.35)			
ASV_489	Actinobacteria;D_2__Actinobacteria;D_3__Actinomycetales;D_4__Actinomycetaceae;D_5__Actinomyces;D_6__Actinomyces graevenitzii F0530;D_7__;D_8__;D_9__;D_10__;D_11__;D_12__;D_13__;D_14__	2.75(0.78,9.71)	1.65(0.46,5.97)	0.60(0.18,2.06)	2.95(0.79,11.00)	1.78(0.49,6.54)	0.60(0.18,2.08)			
ASV_490	Firmicutes;D_2__Clostridia;D_3__Clostridiales;D_4__Lachnospiraceae;D_5__[Ruminococcus] gnavus group	1.05(0.35,3.12)	2.61(0.85,8.01)	2.48(0.86,7.13)	1.01(0.33,3.12)	3.39(1.10,10.46);p=0.03409	3.36(1.17,9.61);p=0.02417		↑	↑
ASV_494	Proteobacteria;D_2__Gammaproteobacteria;D_3__Betaproteobacteriales;D_4__Neisseriaceae;D_5__Neisseria;D_6__uncultured bacterium;D_7__;D_8__;D_9__;D_10__;D_11__;D_12__;D_13__;D_14__	0.00(0.00,Inf)	0.00(0.00,Inf)	1.26(0.08,19.37)	0.00(0.00,Inf)	0.00(0.00,Inf)	0.92(0.06,13.37)			
ASV_502	Bacteroidetes;D_2__Bacteroidia;D_3__Bacteroidales;D_4__Bacteroidaceae;D_5__Bacteroides;D_6__Bacteroides fragilis;D_7__;D_8__;D_9__;D_10__;D_11__;D_12__;D_13__;D_14__	1.35(0.34,5.32)	0.49(0.12,2.03)	0.37(0.10,1.38)	1.78(0.42,7.49)	0.52(0.13,2.20)	0.29(0.08,1.12)			

ASV_504	Firmicutes;D_2__Erysipelotrichia;D_3__Erysipelotrichales;D_4__Erysipelotrichaceae;D_5__Turicibacter	2.44(1.04,5.74);p=0.04063	0.76(0.31,1.82)	0.31(0.14,0.71);p=0.00552	2.40(0.98,5.88)	0.70(0.29,1.71)	0.29(0.13,0.67);p=0.00371			↓
ASV_505	Firmicutes;D_2__Clostridia;D_3__Clostridiales;D_4__Lachnospiraceae;D_5__Tyzzerella 3;D_6__unidentified;D_7__;D_8__;D_9__;D_10__;D_11__;D_12__;D_13__;D_14__	2.38(0.22,25.90)	3.08(0.26,36.07)	1.30(0.13,13.11)						
ASV_506	Firmicutes;D_2__Clostridia;D_3__Clostridiales;D_4__Lachnospiraceae	0.00(0.00,Inf)	0.00(0.00,Inf)	10.54(0.77,144.24)						
ASV_508	Firmicutes;D_2__Clostridia;D_3__Clostridiales;D_4__Ruminococcaceae;D_5__Ruminococcaceae UCG-005	0.37(0.07,1.88)	0.44(0.08,2.34)	1.19(0.25,5.65)	0.65(0.13,3.36)	0.58(0.11,2.99)	0.89(0.20,4.08)			
ASV_509	Firmicutes;D_2__Clostridia;D_3__Clostridiales;D_4__Lachnospiraceae;D_5__[Eubacterium] ruminantium group;D_6__uncultured bacterium;D_7__;D_8__;D_9__;D_10__;D_11__;D_12__;D_13__;D_14__	0.66(0.06,6.86)	1.29(0.12,14.48)	1.97(0.20,19.10)						
ASV_510	Firmicutes;D_2__Clostridia;D_3__Clostridiales;D_4__Lachnospiraceae;D_5__Blautia	0.41(0.04,4.33)	1.44(0.13,16.08)	3.46(0.36,33.51)	0.36(0.03,3.99)	2.62(0.24,28.95)	7.27(0.77,68.38)			
ASV_512	Firmicutes;D_2__Clostridia;D_3__Clostridiales;D_4__Lachnospiraceae;D_5__Lachnoclostridium	0.58(0.07,4.52)	2.73(0.32,23.06)	4.72(0.64,34.95)	2.49(0.33,18.93)	5.16(0.66,40.24)	2.08(0.31,14.11)			
ASV_516	Firmicutes;D_2__Clostridia;D_3__Clostridiales;D_4__Ruminococcaceae;D_5__Negativibacillus;D_6__uncultured bacterium;D_7__;D_8__;D_9__;D_10__;D_11__;D_12__;D_13__;D_14__	0.82(0.07,10.06)	0.60(0.05,7.93)	0.73(0.06,8.29)						
ASV_518	Firmicutes;D_2__Clostridia;D_3__Clostridiales;D_4__Peptostreptococcaceae;D_5__Romboutsia	1.76(0.05,66.93)	0.09(0.00,3.71)	0.05(0.00,1.72)						
ASV_519	Bacteroidetes;D_2__Bacteroidia;D_3__Bacteroidales;D_4__Barnesiellaceae;D_5__Coprobacter	3.72(0.18,76.42)	0.70(0.03,15.75)	0.19(0.01,3.52)						
ASV_524	Firmicutes;D_2__Clostridia;D_3__Clostridiales;D_4__Ruminococcaceae;D_5__Butyricicoccus	1.55(0.46,5.23)	0.86(0.25,3.00)	0.55(0.17,1.80)	1.54(0.43,5.52)	0.80(0.23,2.86)	0.52(0.16,1.70)			
ASV_526	Firmicutes;D_2__Clostridia;D_3__Clostridiales;D_4__Lachnospiraceae;D_5__uncultured;D_6__metagenome;D_7__;D_8__;D_9__;D_10__;D_11__;D_12__;D_13__;D_14__	0.46(0.11,2.00)	0.60(0.13,2.74)	1.31(0.32,5.39)	0.34(0.07,1.62)	0.47(0.10,2.21)	1.37(0.33,5.68)			
ASV_529	Firmicutes;D_2__Clostridia;D_3__Clostridiales;D_4__Ruminococcaceae;D_5__Ruminiclostridium 9	0.51(0.10,2.63)	3.92(0.72,21.27)	7.67(1.56,37.64);p=0.01206						
ASV_536	Firmicutes;D_2__Clostridia;D_3__Clostridiales;D_4__Clostridiaceae 1;D_5__Clostridium sensu stricto 1	1.69(0.70,4.08)	0.90(0.36,2.24)	0.53(0.23,1.26)	1.77(0.70,4.42)	0.98(0.39,2.46)	0.56(0.24,1.31)			
ASV_537	Firmicutes;D_2__Clostridia;D_3__Clostridiales;D_4__Ruminococcaceae;D_5__Ruminiclostridium 5	0.50(0.02,11.77)	0.13(0.01,3.36)	0.26(0.01,5.38)	0.03(0.00,1.09)	0.07(0.00,2.04)	2.01(0.11,36.77)			
ASV_541	Firmicutes;D_2__Clostridia;D_3__Clostridiales;D_4__Ruminococcaceae;D_5__uncultured;D_6__unidentified;D_7__;D_8__;D_9__;D_10__;D_11__;D_12__;D_13__;D_14__	1.48(0.21,10.54)	1.70(0.22,12.89)	1.15(0.17,7.76)	3.42(0.46,25.37)	1.77(0.25,12.72)	0.52(0.08,3.37)			
ASV_545	Firmicutes;D_2__Clostridia;D_3__Clostridiales;D_4__Lachnospiraceae;D_5__[Eubacterium] ventriosum group;D_6__uncultured bacterium;D_7__;D_8__;D_9__;D_10__;D_11__;D_12__;D_13__;D_14__	0.38(0.02,6.65)	2.57(0.13,49.78)	6.85(0.42,110.82)	0.08(0.00,1.43)	1.58(0.09,28.52)	20.17(1.38,295.58);p=0.02831			↑
ASV_547	Firmicutes;D_2__Clostridia;D_3__Clostridiales;D_4__Ruminococcaceae;D_5__Oscillibacter;D_6__Oscillibacter sp. Marseille-P3260;D_7__;D_8__;D_9__;D_10__;D_11__;D_12__;D_13__;D_14__	0.11(0.01,1.45)	0.21(0.01,2.92)	1.85(0.18,19.24)						
ASV_549	Firmicutes;D_2__Clostridia;D_3__Clostridiales;D_4__Lachnospiraceae	0.73(0.17,3.17)	1.12(0.25,5.10)	1.53(0.37,6.36)	0.46(0.10,1.99)	0.72(0.16,3.14)	1.58(0.40,6.18)			
ASV_551	Firmicutes;D_2__Clostridia;D_3__Clostridiales;D_4__Lachnospiraceae;D_5__[Eubacterium] xylanophilum group	1.16(0.30,4.55)	0.60(0.15,2.44)	0.51(0.14,1.93)	1.97(0.48,8.08)	0.72(0.18,2.93)	0.36(0.10,1.36)			
ASV_552	Firmicutes;D_2__Clostridia;D_3__Clostridiales;D_4__Christensenellaceae;D_5__Christensenellaceae R-7 group	0.40(0.05,3.14)	0.41(0.05,3.42)	1.03(0.15,7.20)						
ASV_553	Cyanobacteria;D_2__Oxyphotobacteria;D_3__Chloroplast	1.97(0.13,30.85)	0.78(0.05,13.05)	0.40(0.03,5.70)	16.54(0.98,278.48)	3.35(0.27,41.69)	0.20(0.01,3.26)			
ASV_557	Firmicutes;D_2__Clostridia;D_3__Clostridiales;D_4__Christensenellaceae;D_5__uncultured;D_6__uncultured bacterium;D_7__;D_8__;D_9__;D_10__;D_11__;D_12__;D_13__;D_14__	1.92(0.57,6.41)	1.58(0.46,5.45)	0.82(0.25,2.67)	1.83(0.51,6.47)	1.48(0.42,5.23)	0.81(0.25,2.66)			
ASV_560	Firmicutes;D_2__Clostridia;D_3__Clostridiales;D_4__Lachnospiraceae;D_5__Anaerostipes;D_6__unidentified;D_7__;D_8__;D_9__;D_10__;D_11__;D_12__;D_13__;D_14__				3.08(0.32,29.31)	3.24(0.34,30.72)	1.05(0.13,8.68)			
ASV_561	Actinobacteria;D_2__Coriobacteriia;D_3__Coriobacteriales;D_4__Coriobacteriales Incertae Sedis;D_5__Raoultibacter;D_6__Raoultibacter timonensis;D_7__;D_8__;D_9__;D_10__;D_11__;D_12__;D_13__;D_14__	2.30(0.25,21.29)	0.75(0.08,7.11)	0.33(0.04,2.79)	2.64(0.26,27.17)	0.38(0.04,3.58)	0.15(0.02,1.26)			
ASV_564	Firmicutes;D_2__Clostridia;D_3__Clostridiales;D_4__Ruminococcaceae	10.70(0.49,233.72)	7.68(0.35,170.49)	0.72(0.03,18.04)						
ASV_566	Firmicutes;D_2__Clostridia;D_3__Clostridiales;D_4__Ruminococcaceae	6.21(1.43,26.93);p=0.01473	5.57(1.23,25.22);p=0.02598	0.90(0.22,3.72)	5.13(1.11,23.74);p=0.03634	6.18(1.34,28.54);p=0.01968	1.20(0.29,5.04)	↑	↑	
ASV_568	Firmicutes;D_2__Clostridia;D_3__Clostridiales;D_4__Clostridiales vadinBB60 group;D_5__gut metagenome;D_6__;D_7__;D_8__;D_9__;D_10__;D_11__;D_12__;D_13__;D_14__	0.24(0.03,1.77)	0.18(0.02,1.39)	0.74(0.11,4.94)	0.69(0.09,5.52)	0.18(0.02,1.43)	0.27(0.04,1.78)			

ASV_570	Firmicutes;D_2__Clostridia;D_3__Clostridiales;D_4__Peptococcaceae;D_5__uncultured;D_6__uncultured bacterium;D_7__;D_8__;D_9__;D_10__;D_11__;D_12__;D_13__;D_14__	1.12(0.05,27.64)	0.27(0.01,6.00)	0.24(0.01,4.38)						
ASV_572	Firmicutes;D_2__Clostridia;D_3__Clostridiales;D_4__Christensenellaceae;D_5__Christensenellaceae R-7 group;D_6__uncultured marine bacterium;D_7__;D_8__;D_9__;D_10__;D_11__;D_12__;D_13__;D_14__	1.37(0.57,3.32)	1.73(0.69,4.30)	1.26(0.53,2.97)	1.80(0.72,4.49)	1.78(0.71,4.45)	0.99(0.42,2.34)			
ASV_573	Firmicutes;D_2__Bacilli;D_3__Lactobacillales;D_4__Streptococcaceae;D_5__Streptococcus;D_6__Streptococcus mutans;D_7__;D_8__;D_9__;D_10__;D_11__;D_12__;D_13__;D_14__	1.96(0.57,6.75)	0.46(0.13,1.62)	0.23(0.07,0.77); p=0.01672	1.48(0.41,5.37)	0.34(0.09,1.24)	0.23(0.07,0.77);p=0.01704			↓
ASV_576	Actinobacteria;D_2__Actinobacteria;D_3__Actinomycetales;D_4__Actinomycetaceae;D_5__Actinomyces	1.92(0.14,27.20)	0.69(0.05,9.45)	0.36(0.03,4.55)						
ASV_580	Firmicutes;D_2__Clostridia;D_3__Clostridiales;D_4__Ruminococcaceae;D_5__Anaerotruncus;D_6__uncultured bacterium;D_7__;D_8__;D_9__;D_10__;D_11__;D_12__;D_13__;D_14__	1.76(0.11,28.96)	0.54(0.03,8.61)	0.30(0.02,4.37)	1.42(0.07,28.18)	0.37(0.02,5.85)	0.26(0.02,4.49)			
ASV_581	Firmicutes;D_2__Erysipelotrichia;D_3__Erysipelotrichales;D_4__Erysipelotrichaceae;D_5__Solobacterium	2.30(0.45,11.76)	1.28(0.24,6.66)	0.56(0.11,2.72)						
ASV_582	Firmicutes;D_2__Clostridia;D_3__Clostridiales;D_4__Family XIII;D_5__Family XIII AD3011 group;D_6__gut metagenome;D_7__;D_8__;D_9__;D_10__;D_11__;D_12__;D_13__;D_14__	0.45(0.03,7.56)	13.21(0.52,333.76)	29.25(1.37,622.21);p=0.03046						
ASV_585	Firmicutes;D_2__Clostridia;D_3__Clostridiales;D_4__Ruminococcaceae;D_5__Ruminiclostridium 5;D_6__uncultured bacterium;D_7__;D_8__;D_9__;D_10__;D_11__;D_12__;D_13__;D_14__	0.76(0.14,4.07)	1.35(0.24,7.66)	1.77(0.35,8.99)						
ASV_586	Firmicutes;D_2__Clostridia;D_3__Clostridiales;D_4__Ruminococcaceae;D_5__Anaerotruncus;D_6__uncultured organism;D_7__;D_8__;D_9__;D_10__;D_11__;D_12__;D_13__;D_14__	0.57(0.22,1.46)	0.77(0.29,2.03)	1.35(0.55,3.33)	0.60(0.22,1.62)	0.76(0.28,2.04)	1.26(0.51,3.13)			
ASV_588	Firmicutes;D_2__Erysipelotrichia;D_3__Erysipelotrichales;D_4__Erysipelotrichaceae;D_5__uncultured;D_6__Clostridiales bacterium 60-7e;D_7__;D_8__;D_9__;D_10__;D_11__;D_12__;D_13__;D_14__	45.24(3.55,577.11);p=0.00334	4.23(0.31,57.15)	0.09(0.01,1.10)						
ASV_590	Firmicutes;D_2__Clostridia;D_3__Clostridiales;D_4__Lachnospiraceae	2.40(0.39,14.68)	0.74(0.13,4.15)	0.31(0.05,1.73)						
ASV_591	Firmicutes;D_2__Clostridia;D_3__Clostridiales;D_4__Clostridiales vadinBB60 group;D_5__gut metagenome;D_6__;D_7__;D_8__;D_9__;D_10__;D_11__;D_12__;D_13__;D_14__				1.53(0.17,14.17)	0.33(0.04,2.93)	0.21(0.03,1.66)			
ASV_594	Firmicutes;D_2__Clostridia;D_3__Clostridiales;D_4__Ruminococcaceae;D_5__Candidatus Soleaferrea;D_6__Bittarella massiliensis;D_7__;D_8__;D_9__;D_10__;D_11__;D_12__;D_13__;D_14__	0.77(0.34,1.77)	0.90(0.38,2.11)	1.16(0.52,2.58)	0.89(0.38,2.11)	1.07(0.45,2.55)	1.20(0.54,2.69)			
ASV_595	Firmicutes;D_2__Clostridia;D_3__Clostridiales;D_4__Lachnospiraceae	0.25(0.03,2.11)	0.22(0.02,1.95)	0.87(0.12,6.50)	0.27(0.03,2.47)	0.33(0.04,2.95)	1.20(0.16,8.93)			
ASV_596	Firmicutes;D_2__Clostridia;D_3__Clostridiales;D_4__Lachnospiraceae;D_5__[Eubacterium] fissicatena group	1.30(0.53,3.22)	1.10(0.43,2.79)	0.84(0.35,2.03)	1.45(0.56,3.75)	1.22(0.47,3.14)	0.84(0.35,2.03)			
ASV_597	Firmicutes;D_2__Clostridia;D_3__Clostridiales;D_4__Lachnospiraceae;D_5__Lachnoclostridium;D_6__[Clostridium] scindens;D_7__;D_8__;D_9__;D_10__;D_11__;D_12__;D_13__;D_14__	0.89(0.18,4.36)	5.09(0.99,26.28)	5.70(1.22,26.67);p=0.02718	0.87(0.16,4.59)	5.10(0.96,27.11)	5.88(1.24,27.96);p=0.02592			↑
ASV_599	Firmicutes;D_2__Clostridia;D_3__Clostridiales;D_4__Ruminococcaceae;D_5__Ruminiclostridium 9	0.40(0.13,1.23)	0.33(0.10,1.02)	0.81(0.28,2.34)	0.52(0.16,1.67)	0.34(0.11,1.10)	0.66(0.23,1.92)			
ASV_600	Firmicutes;D_2__Clostridia;D_3__Clostridiales;D_4__Lachnospiraceae;D_5__UC5-1-2E3;D_6__uncultured bacterium;D_7__;D_8__;D_9__;D_10__;D_11__;D_12__;D_13__;D_14__	0.53(0.09,3.26)	1.29(0.20,8.40)	2.42(0.42,14.00)						
ASV_601	Firmicutes;D_2__Clostridia;D_3__Clostridiales;D_4__Lachnospiraceae	1.21(0.29,5.09)	3.09(0.69,13.75)	2.55(0.62,10.40)	0.83(0.19,3.62)	2.57(0.58,11.38)	3.08(0.77,12.41)			
ASV_609	Bacteroidetes;D_2__Bacteroidia;D_3__Bacteroidales;D_4__Bacteroidaceae;D_5__Bacteroides;D_6__Bacteroides ovatus;D_7__;D_8__;D_9__;D_10__;D_11__;D_12__;D_13__;D_14__	1.58(0.12,20.30)	0.72(0.05,9.96)	0.45(0.04,5.37)	0.70(0.05,9.45)	1.20(0.09,16.08)	1.71(0.15,19.25)			
ASV_613	Firmicutes;D_2__Erysipelotrichia;D_3__Erysipelotrichales;D_4__Erysipelotrichaceae;D_5__Turicibacter	2.41(0.49,11.95)	1.86(0.36,9.66)	0.77(0.16,3.63)						
ASV_618	Actinobacteria;D_2__Coriobacteriia;D_3__Coriobacteriales;D_4__Eggerthellaceae;D_5__Paraeggerthella;D_6__Eggerthellaceae bacterium;D_7__;D_8__;D_9__;D_10__;D_11__;D_12__;D_13__;D_14__	1.13(0.04,28.62)	0.50(0.02,13.21)	0.44(0.02,9.58)						
ASV_620	Firmicutes;D_2__Clostridia;D_3__Clostridiales;D_4__Lachnospiraceae;D_5__uncultured;D_6__uncultured organism;D_7__;D_8__;D_9__;D_10__;D_11__;D_12__;D_13__;D_14__	0.73(0.14,3.76)	1.52(0.28,8.34)	2.10(0.43,10.38)	1.12(0.20,6.15)	1.51(0.27,8.34)	1.35(0.28,6.64)			
ASV_621	Firmicutes;D_2__Clostridia;D_3__Clostridiales;D_4__Ruminococcaceae;D_5__Intestinimonas;D_6__Intestinimonas butyriciproducens;D_7__;D_8__;D_9__;D_10__;D_11__;D_12__;D_13__;D_14__	1.04(0.32,3.43)	1.21(0.35,4.13)	1.16(0.37,3.68)	1.36(0.40,4.59)	1.22(0.36,4.14)	0.90(0.29,2.82)			
ASV_623	Firmicutes;D_2__Clostridia;D_3__Clostridiales;D_4__Lachnospiraceae;D_5__[Ruminococcus] torques group;D_6__uncultured bacterium;D_7__;D_8__;D_9__;D_10__;D_11__;D_12__;D_13__;D_14__	0.63(0.14,2.83)	1.53(0.33,7.17)	2.42(0.57,10.31)						
ASV_628	Firmicutes;D_2__Clostridia;D_3__Clostridiales;D_4__Ruminococcaceae;D_5__Butyricicoccus	0.80(0.30,2.11)	1.45(0.53,3.98)	1.81(0.71,4.67)	1.12(0.42,2.98)	2.22(0.82,6.00)	1.99(0.79,5.01)			
ASV_630	Firmicutes;D_2__Clostridia;D_3__Clostridiales;D_4__Clostridiaceae 1;D_5__Clostridium sensu stricto 1	0.50(0.05,5.22)	0.17(0.02,1.86)	0.34(0.04,3.14)	0.95(0.11,8.50)	0.94(0.11,8.28)	0.99(0.13,7.50)			

ASV_637	Firmicutes;D_2__Clostridia;D_3__Clostridiales;D_4__Ruminococcaceae;D_5__Ruminococcaceae UCG-005	2.05(0.33,12.85)	1.07(0.17,6.73)	0.52(0.09,3.09)	1.31(0.20,8.38)	0.78(0.13,4.57)	0.59(0.11,3.35)			
ASV_641	Actinobacteria;D_2__Coriobacteriia;D_3__Coriobacteriales;D_4__Coriobacteriaceae;D_5__Collinsella;D_6__uncultured bacterium;D_7__;D_8__;D_9__;D_10__;D_11__;D_12__;D_13__;D_14__	0.47(0.06,3.62)	0.48(0.06,3.96)	1.03(0.14,7.33)	0.78(0.10,6.11)	0.83(0.11,6.44)	1.06(0.16,7.09)			
ASV_642	Firmicutes;D_2__Clostridia;D_3__Clostridiales;D_4__Lachnospiraceae	0.27(0.05,1.48)	0.39(0.07,2.26)	1.45(0.28,7.48)	0.41(0.07,2.41)	0.36(0.06,2.12)	0.88(0.17,4.49)			
ASV_643	Firmicutes;D_2__Clostridia;D_3__Clostridiales;D_4__Ruminococcaceae;D_5__Candidatus Soleaferrea;D_6__uncultured Anaerotruncus sp.;D_7__;D_8__;D_9__;D_10__;D_11__;D_12__;D_13__;D_14__	0.40(0.12,1.32)	0.91(0.26,3.15)	2.27(0.72,7.18)	0.86(0.25,2.91)	0.83(0.25,2.83)	0.97(0.32,3.00)			
ASV_644	Firmicutes;D_2__Clostridia;D_3__Clostridiales;D_4__Lachnospiraceae;D_5__Lachnospiraceae UCG-008;D_6__uncultured organism;D_7__;D_8__;D_9__;D_10__;D_11__;D_12__;D_13__;D_14__	1.61(0.28,9.26)	3.02(0.49,18.64)	1.88(0.34,10.48)	0.72(0.12,4.17)	0.98(0.17,5.59)	1.36(0.27,6.94)			
ASV_646	Firmicutes;D_2__Clostridia;D_3__Clostridiales;D_4__Ruminococcaceae	0.41(0.06,2.66)	0.24(0.04,1.58)	0.59(0.12,2.93)	0.26(0.04,1.80)	0.21(0.03,1.38)	0.80(0.17,3.80)			
ASV_647	Firmicutes;D_2__Clostridia;D_3__Clostridiales;D_4__Ruminococcaceae;D_5__Acetanaerobacterium;D_6__uncultured rumen bacterium;D_7__;D_8__;D_9__;D_10__;D_11__;D_12__;D_13__;D_14__	1.98(0.57,6.93)	3.66(0.99,13.55)	1.84(0.53,6.40)	2.07(0.55,7.70)	3.74(0.99,14.21)	1.81(0.52,6.37)			
ASV_648	Firmicutes;D_2__Clostridia;D_3__Clostridiales;D_4__Lachnospiraceae;D_5__Lachnospiraceae UCG-004;D_6__uncultured organism;D_7__;D_8__;D_9__;D_10__;D_11__;D_12__;D_13__;D_14__	0.00(0.00,Inf)	0.00(0.00,Inf)	0.22(0.02,2.77)	0.00(0.00,Inf)	0.00(0.00,Inf)	0.84(0.07,9.59)			
ASV_649	Firmicutes;D_2__Clostridia;D_3__Clostridiales;D_4__Clostridiaceae 1;D_5__Clostridium sensu stricto 1	0.26(0.01,4.93)	0.84(0.04,18.22)	3.26(0.19,55.37)						
ASV_650	Firmicutes;D_2__Clostridia;D_3__Clostridiales;D_4__Christensenellaceae;D_5__Catabacter	1.25(0.32,4.94)	0.91(0.22,3.71)	0.73(0.19,2.73)	2.14(0.54,8.49)	1.14(0.30,4.41)	0.53(0.15,1.92)			
ASV_651	Actinobacteria;D_2__Actinobacteria;D_3__Micrococcales;D_4__Micrococcaceae;D_5__Rothia;D_6__uncultured organism;D_7__;D_8__;D_9__;D_10__;D_11__;D_12__;D_13__;D_14__	7.28(0.33,162.70)	19.19(0.71,520.31)	2.63(0.11,62.57)						
ASV_652	Proteobacteria;D_2__Gammaproteobacteria;D_3__Betaproteobacteriales;D_4__Burkholderiaceae;D_5__Noviherbaspirillum	0.30(0.01,8.83)	0.10(0.00,3.02)	0.33(0.02,6.79)						
ASV_653	Firmicutes;D_2__Clostridia;D_3__Clostridiales;D_4__Ruminococcaceae;D_5__GCA-900066225;D_6__Massilimaliae massiliensis;D_7__;D_8__;D_9__;D_10__;D_11__;D_12__;D_13__;D_14__	1.20(0.18,8.16)	1.26(0.17,9.05)	1.04(0.16,6.73)	1.46(0.20,10.93)	1.34(0.18,9.92)	0.91(0.14,6.01)			
ASV_656	Firmicutes;D_2__Clostridia;D_3__Clostridiales;D_4__Family XIII;D_5__Family XIII AD3011 group;D_6__uncultured bacterium;D_7__;D_8__;D_9__;D_10__;D_11__;D_12__;D_13__;D_14__	0.80(0.19,3.37)	5.13(1.10,23.87);p=0.03719	6.39(1.50,27.23);p=0.01209	2.41(0.58,10.03)	8.09(1.79,36.56);p=0.0066	3.36(0.82,13.77)		↑	
ASV_658	Firmicutes;D_2__Clostridia;D_3__Clostridiales;D_4__Ruminococcaceae;D_5__Flavonifractor;D_6__uncultured bacterium;D_7__;D_8__;D_9__;D_10__;D_11__;D_12__;D_13__;D_14__	0.42(0.14,1.27)	0.83(0.26,2.64)	1.99(0.68,5.85)	0.41(0.13,1.33)	0.62(0.19,1.99)	1.49(0.51,4.39)			
ASV_669	Firmicutes;D_2__Clostridia;D_3__Clostridiales;D_4__Lachnospiraceae;D_5__[Eubacterium] hallii group	0.00(0.00,Inf)	0.00(0.00,Inf)	431.17(19.87,9354.21);p=0.00011	0.00(0.00,Inf)	0.00(0.00,Inf)	270.00(13.67,5334.08);p=0.00024			↑
ASV_672	Firmicutes;D_2__Bacilli;D_3__Lactobacillales;D_4__Lactobacillaceae;D_5__Lactobacillus	2.04(0.09,45.08)	1.02(0.04,23.18)	0.50(0.02,9.97)	7.27(0.31,172.79)	6.74(0.29,157.43)	0.93(0.04,20.31)			
ASV_674	Firmicutes;D_2__Clostridia;D_3__Clostridiales;D_4__Clostridiaceae 1;D_5__Clostridium sensu stricto 1;D_6__Clostridium perfringens;D_7__;D_8__;D_9__;D_10__;D_11__;D_12__;D_13__;D_14__	0.49(0.04,5.47)	2.86(0.23,35.62)	5.86(0.55,62.44)	0.78(0.06,9.75)	3.24(0.25,41.68)	4.18(0.39,45.17)			
ASV_676	Firmicutes;D_2__Bacilli;D_3__Lactobacillales;D_4__Lactobacillaceae;D_5__Lactobacillus	3.63(0.57,23.32)	29.23(4.29,199.11);p=0.00057	8.05(1.32,48.92);p=0.02348	4.02(0.64,25.23)	11.30(1.80,70.85);p=0.00964	2.81(0.50,15.66)		↑	
ASV_678	Firmicutes;D_2__Erysipelotrichia;D_3__Erysipelotrichales;D_4__Erysipelotrichaceae;D_5__Erysipelatoclostridium	2.88(1.22,6.77);p=0.01534	2.33(0.97,5.63)	0.81(0.35,1.86)	2.72(1.12,6.65);p=0.02764	2.24(0.92,5.45)	0.82(0.36,1.89)	↑		
ASV_679	Firmicutes;D_2__Clostridia;D_3__Clostridiales;D_4__Peptostreptococcaceae;D_5__Paeniclostridium;D_6__uncultured bacterium;D_7__;D_8__;D_9__;D_10__;D_11__;D_12__;D_13__;D_14__	13.51(1.31,139.13);p=0.02867	6.13(0.58,64.28)	0.45(0.05,4.56)						
ASV_680	Firmicutes;D_2__Clostridia;D_3__Clostridiales;D_4__Ruminococcaceae;D_5__Butyricicoccus	0.55(0.05,6.52)	0.09(0.01,1.08)	0.15(0.01,1.68)	0.59(0.05,7.59)	0.09(0.01,1.11)	0.15(0.01,1.58)			
ASV_681	Firmicutes;D_2__Clostridia;D_3__Clostridiales;D_4__Lachnospiraceae;D_5__uncultured				0.29(0.05,1.74)	0.15(0.03,0.89);p=0.03702	0.52(0.10,2.57)		↓	
ASV_683	Firmicutes;D_2__Clostridia;D_3__Clostridiales;D_4__Ruminococcaceae;D_5__Ruminiclostridium 5;D_6__uncultured organism;D_7__;D_8__;D_9__;D_10__;D_11__;D_12__;D_13__;D_14__	0.89(0.33,2.43)	1.18(0.42,3.31)	1.32(0.50,3.47)	1.19(0.43,3.29)	1.20(0.43,3.32)	1.01(0.39,2.61)			
ASV_685	Firmicutes;D_2__Clostridia;D_3__Clostridiales;D_4__Lachnospiraceae;D_5__[Ruminococcus] torques group	2.36(0.33,16.85)	1.62(0.21,12.23)	0.69(0.10,4.60)						
ASV_688	Firmicutes;D_2__Clostridia;D_3__Clostridiales;D_4__Ruminococcaceae;D_5__uncultured	0.18(0.01,6.18)	0.28(0.01,10.34)	1.50(0.05,42.68)						

ASV_692	Actinobacteria;D_2__Actinobacteria;D_3__Bifidobacteriales;D_4__Bifidobacteriaceae;D_5__Alloscardovia;D_6__Bifidobacterium longum subsp. longum;D_7__;D_8__;D_9__;D_10__;D_11__;D_12__;D_13__;D_14__	1.48(0.08,28.15)	0.81(0.04,16.18)	0.55(0.03,9.32)						
ASV_695	Actinobacteria;D_2__Actinobacteria;D_3__Micrococcales;D_4__Micrococcaceae;D_5__Rothia	1.76(0.30,10.21)	1.21(0.20,7.33)	0.69(0.12,3.77)						
ASV_696	Actinobacteria;D_2__Actinobacteria;D_3__Actinomycetales;D_4__Actinomycetaceae;D_5__Actinomyces	1.34(0.24,7.36)	0.85(0.15,4.79)	0.64(0.12,3.27)	0.88(0.16,4.94)	0.84(0.15,4.70)	0.96(0.19,4.75)			
ASV_697	Firmicutes;D_2__Erysipelotrichia;D_3__Erysipelotrichales;D_4__Erysipelotrichaceae;D_5__Faecalitalea	0.72(0.18,2.90)	1.64(0.39,6.93)	2.29(0.59,8.85)	0.91(0.21,3.86)	2.33(0.55,9.88)	2.55(0.66,9.85)			
ASV_698	Firmicutes;D_2__Clostridia;D_3__Clostridiales;D_4__Lachnospiraceae;D_5__Sellimonas;D_6__Lachnoclostridium phocaeense;D_7__;D_8__;D_9__;D_10__;D_11__;D_12__;D_13__;D_14__	0.09(0.01,1.49)	0.54(0.03,9.56)	5.77(0.41,80.89)						
ASV_699	Bacteroidetes;D_2__Bacteroidia;D_3__Bacteroidales;D_4__Prevotellaceae;D_5__Prevotella 6;D_6__uncultured bacterium;D_7__;D_8__;D_9__;D_10__;D_11__;D_12__;D_13__;D_14__	1.19(0.04,35.15)	0.21(0.01,5.75)	0.18(0.01,3.96)						
ASV_702	Firmicutes;D_2__Clostridia;D_3__Clostridiales;D_4__Ruminococcaceae;D_5__uncultured;D_6__human gut metagenome;D_7__;D_8__;D_9__;D_10__;D_11__;D_12__;D_13__;D_14__	0.70(0.08,5.93)	1.29(0.14,11.76)	1.84(0.23,14.69)						
ASV_703	Firmicutes;D_2__Clostridia;D_3__Clostridiales;D_4__Christensenellaceae;D_5__Christensenellaceae R-7 group;D_6__bacterium YE57;D_7__;D_8__;D_9__;D_10__;D_11__;D_12__;D_13__;D_14__	8.55(0.41,177.02)	0.69(0.03,14.81)	0.08(0.00,1.51)	11.31(0.47,270.50)	0.37(0.02,7.19)	0.03(0.00,0.63);p=0.02353			↓
ASV_704	Firmicutes;D_2__Clostridia;D_3__Clostridiales;D_4__Ruminococcaceae;D_5__uncultured;D_6__uncultured bacterium;D_7__;D_8__;D_9__;D_10__;D_11__;D_12__;D_13__;D_14__	1.14(0.20,6.50)	0.58(0.10,3.41)	0.51(0.10,2.69)	1.23(0.20,7.58)	0.51(0.09,3.05)	0.42(0.08,2.18)			
ASV_705	Firmicutes;D_2__Clostridia;D_3__Clostridiales;D_4__Family XI;D_5__Ezakiella;D_6__Fenollaria timonensis;D_7__;D_8__;D_9__;D_10__;D_11__;D_12__;D_13__;D_14__	0.39(0.07,2.13)	0.20(0.03,1.11)	0.50(0.10,2.46)	0.75(0.13,4.29)	0.39(0.07,2.18)	0.51(0.10,2.53)			
ASV_708	Firmicutes;D_2__Clostridia;D_3__Clostridiales;D_4__Ruminococcaceae;D_5__Hydrogenoanaerobacterium;D_6__uncultured bacterium;D_7__;D_8__;D_9__;D_10__;D_11__;D_12__;D_13__;D_14__	0.63(0.04,9.30)	0.84(0.05,13.51)	1.32(0.10,17.40)						
ASV_711	Firmicutes;D_2__Clostridia;D_3__Clostridiales;D_4__Lachnospiraceae;D_5__uncultured organism;D_6__;D_7__;D_8__;D_9__;D_10__;D_11__;D_12__;D_13__;D_14__				0.19(0.02,2.45)	0.56(0.04,7.17)	2.91(0.29,28.67)			
ASV_713	Firmicutes;D_2__Clostridia;D_3__Clostridiales;D_4__Lachnospiraceae;D_5__Tyzzerella;D_6__uncultured bacterium;D_7__;D_8__;D_9__;D_10__;D_11__;D_12__;D_13__;D_14__	0.54(0.06,4.77)	0.85(0.09,8.21)	1.59(0.19,13.01)	0.14(0.01,1.42)	0.93(0.09,9.64)	6.47(0.78,53.62)			
ASV_714	Firmicutes;D_2__Clostridia;D_3__Clostridiales;D_4__Lachnospiraceae;D_5__Lachnoclostridium	1.65(0.15,18.14)	0.69(0.06,7.76)	0.42(0.04,4.18)						
ASV_717	Firmicutes;D_2__Clostridia;D_3__Clostridiales;D_4__Family XI;D_5__Peptoniphilus;D_6__uncultured organism;D_7__;D_8__;D_9__;D_10__;D_11__;D_12__;D_13__;D_14__	10.94(1.04,115.01);p=0.04627	77.51(4.73,1269.43);p=0.00229	7.09(0.46,108.37)	10.94(0.91,131.50)	78.09(4.55,1339.81);p=0.00266	7.14(0.46,111.51)		↑	
ASV_721	Actinobacteria;D_2__Actinobacteria;D_3__Actinomycetales;D_4__Actinomycetaceae;D_5__Actinomyces	2.65(0.58,12.10)	0.30(0.06,1.39)	0.11(0.03,0.48);p=0.00333						
ASV_722	Firmicutes;D_2__Erysipelotrichia;D_3__Erysipelotrichales;D_4__Erysipelotrichaceae;D_5__Erysipelatoclostridium;D_6__Massiliomicrobiota timonensis;D_7__;D_8__;D_9__;D_10__;D_11__;D_12__;D_13__;D_14__	2.77(0.51,15.07)	1.53(0.27,8.64)	0.55(0.11,2.87)						
ASV_723	Firmicutes;D_2__Clostridia;D_3__Clostridiales;D_4__Ruminococcaceae;D_5__Ruminiclostridium;D_6__Massilioclostridium coli;D_7__;D_8__;D_9__;D_10__;D_11__;D_12__;D_13__;D_14__				0.92(0.14,6.04)	0.50(0.08,3.21)	0.54(0.10,3.05)			
ASV_726	Firmicutes;D_2__Negativicutes;D_3__Selenomonadales;D_4__Veillonellaceae;D_5__Veillonella	1.26(0.14,11.56)	0.17(0.02,1.57)	0.13(0.02,1.09)	1.40(0.13,14.56)	0.10(0.01,0.96);p=0.04606	0.07(0.01,0.60);p=0.01466		↓	↓
ASV_733	Firmicutes;D_2__Clostridia;D_3__Clostridiales;D_4__Clostridiaceae 1;D_5__Clostridium sensu stricto 1	3.37(0.15,76.36)	4.82(0.19,120.16)	1.43(0.07,29.46)	1.77(0.07,45.82)	5.67(0.22,145.82)	3.20(0.15,66.21)			
ASV_734	Firmicutes;D_2__Clostridia;D_3__Clostridiales;D_4__Lachnospiraceae;D_5__Eisenbergiella;D_6__uncultured organism;D_7__;D_8__;D_9__;D_10__;D_11__;D_12__;D_13__;D_14__	1.75(0.50,6.12)	4.43(1.21,16.26);p=0.02487	2.53(0.74,8.63)	1.72(0.47,6.32)	4.25(1.14,15.78);p=0.03069	2.47(0.72,8.45)		↑	
ASV_737	Firmicutes;D_2__Clostridia;D_3__Clostridiales;D_4__Lachnospiraceae;D_5__Tyzzerella	0.81(0.04,18.57)	13.13(0.52,333.82)	16.16(0.77,338.94)						
ASV_738	Firmicutes;D_2__Clostridia;D_3__Clostridiales;D_4__Ruminococcaceae;D_5__Ruminiclostridium 9;D_6__uncultured bacterium;D_7__;D_8__;D_9__;D_10__;D_11__;D_12__;D_13__;D_14__	0.67(0.04,11.88)	0.03(0.00,0.56);p=0.01865	0.05(0.00,0.67);p=0.02425	1.75(0.09,33.52)	0.08(0.00,1.39)	0.05(0.00,0.67);p=0.02388			↓
ASV_740	Firmicutes;D_2__Clostridia;D_3__Clostridiales;D_4__Lachnospiraceae;D_5__Lachnospiraceae UCG-004;D_6__uncultured organism;D_7__;D_8__;D_9__;D_10__;D_11__;D_12__;D_13__;D_14__	0.34(0.05,2.52)	0.52(0.07,4.05)	1.51(0.22,10.40)	0.21(0.03,1.56)	0.22(0.03,1.63)	1.06(0.17,6.64)			
ASV_741	Firmicutes;D_2__Clostridia;D_3__Clostridiales;D_4__Lachnospiraceae;D_5__Lachnoclostridium;D_6__uncultured organism;D_7__;D_8__;D_9__;D_10__;D_11__;D_12__;D_13__;D_14__	3.04(0.23,39.51)	30.77(1.21,783.31);p=0.03802	10.13(0.43,236.85)	1.99(0.14,27.25)	14.58(0.66,321.39)	7.34(0.31,171.90)			
ASV_743	Firmicutes;D_2__Clostridia;D_3__Clostridiales;D_4__Ruminococcaceae;D_5__Ruminococcaceae UCG-005;D_6__metagenome;D_7__;D_8__;D_9__;D_10__;D_11__;D_12__;D_13__;D_14__	0.21(0.02,2.40)	0.24(0.02,2.93)	1.13(0.11,11.82)	0.18(0.01,2.37)	0.03(0.00,0.43);p=0.00899	0.19(0.02,1.77)		↓	
ASV_747	Firmicutes;D_2__Clostridia;D_3__Clostridiales;D_4__Lachnospiraceae;D_5__Sellimonas;D_6__uncultured bacterium;D_7__;D_8__;D_9__;D_10__;D_11__;D_12__;D_13__;D_14__	1.09(0.30,4.01)	6.81(1.77,26.17);p=0.00519	6.25(1.76,22.17);p=0.00453						

ASV_749	Firmicutes;D_2__Erysipelotrichia;D_3__Erysipelotrichales;D_4__Erysipelotrichaceae;D_5__Candidatus Stoquefichus	0.78(0.09,6.81)	5.85(0.62,55.18)	7.48(0.91,61.71)	2.28(0.26,20.30)	8.96(0.99,81.12)	3.93(0.50,30.65)			
ASV_752	Firmicutes;D_2__Clostridia;D_3__Clostridiales;D_4__Ruminococcaceae;D_5__Negativibacillus;D_6__uncultured bacterium;D_7__;D_8__;D_9__;D_10__;D_11__;D_12__;D_13__;D_14__	1.79(0.13,25.50)	0.73(0.05,11.18)	0.41(0.03,5.33)	4.58(0.34,60.85)	1.91(0.15,24.73)	0.42(0.04,4.68)			
ASV_753	Firmicutes;D_2__Clostridia;D_3__Clostridiales;D_4__Ruminococcaceae;D_5__Oscillibacter	0.55(0.01,20.60)	0.18(0.01,6.36)	0.33(0.02,7.00)						
ASV_755	Firmicutes;D_2__Clostridia;D_3__Clostridiales;D_4__Ruminococcaceae;D_5__Ruminococcaceae UCG-008;D_6__uncultured bacterium;D_7__;D_8__;D_9__;D_10__;D_11__;D_12__;D_13__;D_14__	8312456365.25(0.00,Inf)	0.23(0.01,4.65)	0.00(0.00,Inf)						
ASV_756	Firmicutes;D_2__Clostridia;D_3__Clostridiales;D_4__Lachnospiraceae	0.13(0.01,2.01)	0.47(0.03,7.91)	3.57(0.26,48.59)						
ASV_757	Firmicutes;D_2__Erysipelotrichia;D_3__Erysipelotrichales;D_4__Erysipelotrichaceae;D_5__Faecalitalea;D_6__[Eubacterium] dolichum;D_7__;D_8__;D_9__;D_10__;D_11__;D_12__;D_13__;D_14__	14.45(1.00,209.38)	29.01(1.64,512.59);p=0.02155	2.01(0.12,33.71)						
ASV_760	Firmicutes;D_2__Clostridia;D_3__Clostridiales;D_4__Family XIII;D_5__[Eubacterium] nodatum group	70224450062.04(0.00,Inf)	13.38(0.59,301.79)	0.00(0.00,Inf)						
ASV_761	Firmicutes;D_2__Erysipelotrichia;D_3__Erysipelotrichales;D_4__Erysipelotrichaceae;D_5__Merdibacter;D_6__uncultured bacterium;D_7__;D_8__;D_9__;D_10__;D_11__;D_12__;D_13__;D_14__	2.12(0.10,43.94)	0.79(0.04,16.19)	0.37(0.02,6.84)						
ASV_767	Firmicutes;D_2__Erysipelotrichia;D_3__Erysipelotrichales;D_4__Erysipelotrichaceae;D_5__[Clostridium] innocuum group	0.85(0.06,12.24)	0.44(0.03,6.91)	0.52(0.04,6.87)						
ASV_768	Firmicutes;D_2__Clostridia;D_3__Clostridiales;D_4__Lachnospiraceae				0.00(0.00,Inf)	0.00(0.00,Inf)	1.63(0.18,14.32)			
ASV_771	Firmicutes;D_2__Clostridia;D_3__Clostridiales;D_4__Family XIII;D_5__[Eubacterium] brachy group;D_6__uncultured bacterium;D_7__;D_8__;D_9__;D_10__;D_11__;D_12__;D_13__;D_14__	1.11(0.04,29.58)	0.85(0.03,24.87)	0.76(0.03,18.33)	1.00(0.03,31.45)	0.18(0.01,5.58)	0.18(0.01,4.44)			
ASV_773	Firmicutes;D_2__Clostridia;D_3__Clostridiales;D_4__Ruminococcaceae;D_5__Ruminiclostridium	3.23(0.49,21.48)	2.65(0.38,18.30)	0.82(0.12,5.57)	3.37(0.47,24.12)	3.10(0.44,22.04)	0.92(0.14,6.22)			
ASV_778	Bacteroidetes;D_2__Bacteroidia;D_3__Bacteroidales;D_4__Bacteroidaceae;D_5__Bacteroides	0.00(0.00,Inf)	0.00(0.00,Inf)	1.45(0.07,31.28)						
ASV_779	Firmicutes;D_2__Clostridia;D_3__Clostridiales;D_4__Lachnospiraceae;D_5__Lachnoclostridium	0.53(0.07,3.95)	1.14(0.14,9.23)	2.16(0.31,15.26)	0.49(0.06,3.97)	0.93(0.11,7.66)	1.92(0.27,13.49)			
ASV_780	Actinobacteria;D_2__Coriobacteriia;D_3__Coriobacteriales;D_4__Eggerthellaceae;D_5__Gordonibacter;D_6__unidentified;D_7__;D_8__;D_9__;D_10__;D_11__;D_12__;D_13__;D_14__	3.85(0.13,110.05)	33.98(0.92,1258.27)	8.82(0.29,272.30)						
ASV_792	Firmicutes;D_2__Clostridia;D_3__Clostridiales;D_4__Lachnospiraceae;D_5__Lachnospiraceae UCG-001;D_6__Lachnospiraceae bacterium TF01-11;D_7__;D_8__;D_9__;D_10__;D_11__;D_12__;D_13__;D_14__	0.17(0.01,2.72)	1.75(0.10,29.79)	10.07(0.70,144.36)	0.06(0.00,0.98); p=0.04844	1.33(0.07,23.59)	24.11(1.65,352.25);p=0.02001	↓		↑
ASV_794	Firmicutes;D_2__Clostridia;D_3__Clostridiales;D_4__Lachnospiraceae;D_5__[Ruminococcus] torques group	4.73(0.40,56.26)	3.28(0.27,39.96)	0.69(0.06,8.44)						
ASV_795	Firmicutes;D_2__Clostridia;D_3__Clostridiales;D_4__Lachnospiraceae;D_5__[Ruminococcus] gauvreauii group;D_6__uncultured bacterium;D_7__;D_8__;D_9__;D_10__;D_11__;D_12__;D_13__;D_14__	1.55(0.12,20.59)	1.02(0.07,14.57)	0.66(0.05,8.05)						
ASV_798	Actinobacteria;D_2__Coriobacteriia;D_3__Coriobacteriales;D_4__Eggerthellaceae;D_5__Eggerthella;D_6__uncultured bacterium;D_7__;D_8__;D_9__;D_10__;D_11__;D_12__;D_13__;D_14__	0.45(0.04,4.70)	1.72(0.15,19.37)	3.79(0.39,36.75)	0.24(0.02,2.43)	1.18(0.12,12.00)	4.90(0.57,41.89)			
ASV_804	Firmicutes;D_2__Clostridia;D_3__Clostridiales;D_4__Lachnospiraceae;D_5__Lachnospiraceae NK4A136 group;D_6__uncultured Clostridium sp.;D_7__;D_8__;D_9__;D_10__;D_11__;D_12__;D_13__;D_14__	0.27(0.05,1.37)	0.47(0.09,2.51)	1.73(0.37,8.08)	0.28(0.05,1.52)	0.44(0.08,2.43)	1.59(0.34,7.51)			
ASV_811	Firmicutes;D_2__Clostridia;D_3__Clostridiales;D_4__Ruminococcaceae;D_5__Ruminococcaceae UCG-010;D_6__uncultured bacterium;D_7__;D_8__;D_9__;D_10__;D_11__;D_12__;D_13__;D_14__	0.00(0.00,Inf)	0.00(0.00,Inf)	6.38(0.39,105.54)						
ASV_814	Firmicutes;D_2__Clostridia;D_3__Clostridiales;D_4__Peptococcaceae;D_5__uncultured	0.96(0.07,12.47)	1.61(0.11,22.85)	1.68(0.14,20.36)						
ASV_817	Firmicutes;D_2__Clostridia;D_3__Clostridiales;D_4__Ruminococcaceae	0.91(0.21,4.02)	0.63(0.14,2.88)	0.69(0.17,2.87)	0.84(0.18,3.93)	0.63(0.14,2.95)	0.76(0.18,3.13)			
ASV_819	Firmicutes;D_2__Clostridia;D_3__Clostridiales;D_4__Ruminococcaceae	0.87(0.07,10.29)	0.21(0.02,2.40)	0.24(0.03,2.26)						
ASV_820	Firmicutes;D_2__Clostridia;D_3__Clostridiales;D_4__Lachnospiraceae;D_5__GCA-900066575;D_6__uncultured bacterium;D_7__;D_8__;D_9__;D_10__;D_11__;D_12__;D_13__;D_14__	1.11(0.12,10.62)	0.86(0.08,8.65)	0.77(0.09,6.76)						
ASV_822	Firmicutes;D_2__Clostridia;D_3__Clostridiales;D_4__Family XIII;D_5__Family XIII AD3011 group	0.91(0.05,17.19)	1.30(0.06,27.31)	1.42(0.08,24.79)						
ASV_823	Firmicutes;D_2__Clostridia;D_3__Clostridiales;D_4__Ruminococcaceae;D_5__Ruminococcaceae UCG-010	0.46(0.02,10.40)	0.08(0.00,1.93)	0.18(0.01,3.21)						

ASV_826	Firmicutes;D_2__Clostridia;D_3__Clostridiales;D_4__Christensenellaceae;D_5__Christensenellaceae R-7 group	1.05(0.24,4.57)	0.51(0.12,2.29)	0.49(0.12,1.99)	1.06(0.24,4.67)	0.59(0.14,2.51)	0.56(0.14,2.16)			
ASV_827	Firmicutes;D_2__Clostridia;D_3__Clostridiales;D_4__Ruminococcaceae;D_5__Caproiciproducens;D_6__uncultured bacterium;D_7__;D_8__;D_9__;D_10__;D_11__;D_12__;D_13__;D_14__	1.25(0.03,55.29)	0.05(0.00,2.12)	0.04(0.00,1.37)						
ASV_828	Firmicutes;D_2__Clostridia;D_3__Clostridiales;D_4__Family XIII;D_5__Family XIII AD3011 group	1.21(0.09,16.85)	0.52(0.04,7.56)	0.43(0.03,5.34)	1.02(0.06,16.18)	0.50(0.03,7.62)	0.49(0.04,6.19)			
ASV_829	Firmicutes;D_2__Clostridia;D_3__Clostridiales;D_4__Christensenellaceae;D_5__Christensenellaceae R-7 group;D_6__uncultured Clostridium sp.;D_7__;D_8__;D_9__;D_10__;D_11__;D_12__;D_13__;D_14__	0.22(0.03,1.62)	0.22(0.03,1.73)	1.01(0.17,6.13)	0.18(0.02,1.50)	0.16(0.02,1.32)	0.90(0.15,5.53)			
ASV_830	Firmicutes;D_2__Clostridia;D_3__Clostridiales;D_4__Eubacteriaceae;D_5__Anaerofustis;D_6__unidentified;D_7__;D_8__;D_9__;D_10__;D_11__;D_12__;D_13__;D_14__	1.59(0.30,8.54)	3.94(0.67,23.16)	2.48(0.46,13.27)	3.22(0.59,17.49)	8.41(1.42,49.63);p=0.01874	2.61(0.49,13.91)		↑	
ASV_831	Firmicutes;D_2__Clostridia;D_3__Clostridiales;D_4__Lachnospiraceae;D_5__GCA-900066755;D_6__uncultured bacterium;D_7__;D_8__;D_9__;D_10__;D_11__;D_12__;D_13__;D_14__	0.44(0.04,5.29)	0.65(0.05,8.42)	1.46(0.14,15.55)	0.53(0.04,6.63)	0.96(0.07,12.49)	1.82(0.18,18.89)			
ASV_832	Firmicutes;D_2__Clostridia;D_3__Clostridiales	0.40(0.04,4.42)	0.28(0.02,3.31)	0.71(0.08,6.44)	0.33(0.03,4.29)	0.23(0.02,2.89)	0.70(0.08,6.39)			
ASV_836	Firmicutes;D_2__Clostridia;D_3__Clostridiales;D_4__Peptococcaceae;D_5__uncultured	0.41(0.02,11.04)	1.23(0.03,43.71)	2.96(0.12,73.09)						
ASV_838	Firmicutes;D_2__Clostridia;D_3__Clostridiales;D_4__Christensenellaceae;D_5__uncultured;D_6__uncultured bacterium;D_7__;D_8__;D_9__;D_10__;D_11__;D_12__;D_13__;D_14__	2.41(0.29,20.13)	1.33(0.16,11.10)	0.55(0.07,4.43)						
ASV_839	Firmicutes;D_2__Clostridia;D_3__Clostridiales;D_4__Ruminococcaceae;D_5__Ruminococcaceae UCG-005	1.85(0.15,22.39)	1.34(0.11,16.99)	0.73(0.06,8.32)	2.46(0.17,35.32)	1.12(0.09,13.93)	0.46(0.04,5.65)			
ASV_843	Firmicutes;D_2__Clostridia;D_3__Clostridiales;D_4__Christensenellaceae;D_5__Catabacter;D_6__Christensenella timonensis;D_7__;D_8__;D_9__;D_10__;D_11__;D_12__;D_13__;D_14__	1.65(0.03,80.29)	0.19(0.00,8.30)	0.12(0.00,4.33)						
ASV_845	Firmicutes;D_2__Clostridia;D_3__Clostridiales;D_4__Ruminococcaceae;D_5__Harryflintia	1.92(0.18,20.66)	1.55(0.14,17.51)	0.81(0.08,8.42)						
ASV_846	Firmicutes;D_2__Clostridia;D_3__Clostridiales;D_4__Ruminococcaceae;D_5__Candidatus Soleaferrea;D_6__uncultured bacterium;D_7__;D_8__;D_9__;D_10__;D_11__;D_12__;D_13__;D_14__	0.34(0.03,4.50)	0.44(0.03,6.26)	1.28(0.12,13.43)	0.22(0.02,3.19)	0.46(0.03,6.59)	2.06(0.21,20.60)			
ASV_849	Firmicutes;D_2__Clostridia;D_3__Clostridiales;D_4__Clostridiales vadinBB60 group;D_5__uncultured organism;D_6__;D_7__;D_8__;D_9__;D_10__;D_11__;D_12__;D_13__;D_14__	1.30(0.23,7.40)	1.18(0.20,7.07)	0.91(0.17,4.88)	1.08(0.18,6.63)	0.94(0.15,5.75)	0.87(0.16,4.72)			
ASV_850	Firmicutes;D_2__Clostridia;D_3__Clostridiales;D_4__Christensenellaceae;D_5__Christensenella;D_6__Christensenella minuta;D_7__;D_8__;D_9__;D_10__;D_11__;D_12__;D_13__;D_14__	2.71(0.44,16.51)	1.91(0.30,12.24)	0.71(0.12,4.11)	2.00(0.30,13.21)	0.83(0.13,5.32)	0.41(0.07,2.40)			
ASV_851	Firmicutes;D_2__Clostridia;D_3__Clostridiales;D_4__Ruminococcaceae				0.00(0.00,Inf)	0.00(0.00,Inf)	1.13(0.11,11.45)			
ASV_854	Firmicutes;D_2__Clostridia;D_3__Clostridiales;D_4__Peptostreptococcaceae	312109950028.48(0.00,Inf)	0.66(0.04,10.77)	0.00(0.00,Inf)						
ASV_855	Firmicutes;D_2__Clostridia;D_3__Clostridiales;D_4__Family XIII;D_5__[Eubacterium] nodatum group	0.45(0.05,4.24)	0.98(0.09,10.20)	2.18(0.25,19.08)						
ASV_869	Firmicutes;D_2__Clostridia;D_3__Clostridiales;D_4__Lachnospiraceae;D_5__Coprococcus 2	0.44(0.04,5.34)	0.48(0.04,6.18)	1.07(0.10,11.96)	6.02(0.47,76.42)	1.92(0.15,24.23)	0.32(0.03,3.41)			
ASV_908	Bacteroidetes;D_2__Bacteroidia;D_3__Bacteroidales;D_4__Bacteroidaceae;D_5__Bacteroides	0.24(0.01,5.12)	0.11(0.00,2.47)	0.44(0.02,8.46)						
ASV_920	Firmicutes;D_2__Clostridia;D_3__Clostridiales;D_4__Christensenellaceae;D_5__uncultured;D_6__uncultured bacterium;D_7__;D_8__;D_9__;D_10__;D_11__;D_12__;D_13__;D_14__	4.85(0.27,88.84)	19.91(0.88,448.66)	4.10(0.21,80.29)						
ASV_921	Firmicutes;D_2__Clostridia;D_3__Clostridiales;D_4__Lachnospiraceae;D_5__Dorea	1.65(0.34,8.04)	0.89(0.17,4.54)	0.54(0.12,2.50)						
ASV_922	Firmicutes;D_2__Clostridia;D_3__Clostridiales;D_4__Ruminococcaceae;D_5__Angelakisella;D_6__uncultured bacterium;D_7__;D_8__;D_9__;D_10__;D_11__;D_12__;D_13__;D_14__	1.28(0.22,7.47)	2.41(0.39,14.94)	1.89(0.34,10.51)	0.84(0.14,5.19)	1.52(0.25,9.42)	1.82(0.33,9.98)			
ASV_925	Firmicutes;D_2__Clostridia;D_3__Clostridiales;D_4__Ruminococcaceae;D_5__Ruminococcaceae UCG-009	0.45(0.05,4.31)	0.41(0.04,4.20)	0.91(0.10,8.00)	0.58(0.05,6.16)	0.10(0.01,1.03)	0.17(0.02,1.48)			
ASV_927	Proteobacteria;D_2__Gammaproteobacteria;D_3__Betaproteobacteriales;D_4__Burkholderiaceae;D_5__Sutterella				3.37(0.25,45.72)	1.25(0.09,16.83)	0.37(0.03,4.20)			
ASV_931	Firmicutes;D_2__Clostridia;D_3__Clostridiales;D_4__Ruminococcaceae				0.35(0.03,4.59)	0.27(0.02,3.44)	0.80(0.08,7.83)			
ASV_932	Actinobacteria;D_2__Actinobacteria;D_3__Actinomycetales;D_4__Actinomycetaceae;D_5__Actinomyces	1.09(0.05,26.50)	10.95(0.31,381.86)	10.01(0.34,291.25)						
ASV_937	Firmicutes;D_2__Clostridia;D_3__Clostridiales;D_4__Family XI;D_5__Parvimonas	2.72(0.59,12.66)	1.35(0.28,6.46)	0.49(0.11,2.20)	2.33(0.47,11.64)	1.27(0.26,6.24)	0.54(0.12,2.44)			

ASV_941	Firmicutes;D_2__Clostridia;D_3__Clostridiales;D_4__Ruminococcaceae;D_5__[Eubacterium] coprostanoligenes group;D_6__uncultured rumen bacterium;D_7__;D_8__;D_9__;D_10__;D_11__;D_12__;D_13__;D_14__	2.03(0.06,64.55)	0.39(0.01,12.87)	0.19(0.01,5.34)	1.82(0.04,74.93)	0.11(0.00,3.94)	0.06(0.00,1.84)			
ASV_949	Firmicutes;D_2__Clostridia;D_3__Clostridiales;D_4__Family XI;D_5__Peptoniphilus;D_6__uncultured organism;D_7__;D_8__;D_9__;D_10__;D_11__;D_12__;D_13__;D_14__	2.35(0.22,24.98)	1.34(0.12,15.17)	0.57(0.06,5.66)						
ASV_951	Firmicutes;D_2__Clostridia;D_3__Clostridiales;D_4__Ruminococcaceae	0.05(0.00,0.63);p=0.02022	0.09(0.01,1.17)	1.74(0.21,14.33)						
ASV_953	Firmicutes;D_2__Clostridia;D_3__Clostridiales;D_4__Ruminococcaceae;D_5__Subdoligranulum	1.52(0.25,9.21)	0.62(0.10,4.00)	0.41(0.07,2.36)	1.18(0.18,7.54)	0.73(0.11,4.63)	0.62(0.11,3.46)			
ASV_955	Bacteroidetes;D_2__Bacteroidia;D_3__Bacteroidales;D_4__Porphyromonadaceae;D_5__Porphyromonas	1.38(0.06,31.53)	7.45(0.29,192.29)	5.40(0.25,115.20)	1.34(0.07,24.11)	6.36(0.31,132.37)	4.74(0.28,79.56)			
ASV_956	Firmicutes;D_2__Clostridia;D_3__Clostridiales;D_4__Family XI;D_5__Finegoldia;D_6__uncultured bacterium;D_7__;D_8__;D_9__;D_10__;D_11__;D_12__;D_13__;D_14__	9.41(0.98,89.84)	2.73(0.27,27.34)	0.29(0.03,2.60)	6.27(0.69,56.68)	4.37(0.49,38.82)	0.70(0.09,5.50)			
ASV_959	Firmicutes;D_2__Clostridia;D_3__Clostridiales;D_4__Eubacteriaceae;D_5__Anaerofustis;D_6__Anaerofustis sp. Marseille-P2441;D_7__;D_8__;D_9__;D_10__;D_11__;D_12__;D_13__;D_14__	2.10(0.30,14.87)	3.93(0.51,30.37)	1.87(0.27,13.04)						
ASV_964	Firmicutes;D_2__Clostridia;D_3__Clostridiales;D_4__Clostridiales vadinBB60 group;D_5__gut metagenome;D_6__;D_7__;D_8__;D_9__;D_10__;D_11__;D_12__;D_13__;D_14__	0.28(0.04,2.13)	0.07(0.01,0.58);p=0.01342	0.26(0.04,1.79)	0.59(0.08,4.29)	0.29(0.04,2.10)	0.50(0.08,3.10)			
ASV_965	Firmicutes;D_2__Negativicutes;D_3__Selenomonadales;D_4__Veillonellaceae;D_5__Dialister;D_6__uncultured bacterium;D_7__;D_8__;D_9__;D_10__;D_11__;D_12__;D_13__;D_14__	2.87(0.11,77.91)	17674864049.96(0.00,Inf)	6161036662.62(0.00,Inf)	2.90(0.07,121.08)	33888463097286.40(0.00,Inf)	11681231467672.10(0.00,Inf)			
ASV_966	Firmicutes;D_2__Clostridia;D_3__Clostridiales;D_4__Ruminococcaceae;D_5__Ruminococcaceae UCG-005;D_6__uncultured rumen bacterium;D_7__;D_8__;D_9__;D_10__;D_11__;D_12__;D_13__;D_14__	0.46(0.08,2.85)	0.89(0.14,5.82)	1.92(0.33,11.13)	0.49(0.08,3.23)	0.94(0.14,6.17)	1.91(0.33,10.90)			
ASV_969	Firmicutes;D_2__Clostridia;D_3__Clostridiales;D_4__Ruminococcaceae;D_5__Ruminococcaceae UCG-010	0.34(0.01,7.93)	0.45(0.02,11.39)	1.31(0.06,26.97)						
ASV_972	Firmicutes;D_2__Erysipelotrichia;D_3__Erysipelotrichales;D_4__Erysipelotrichaceae;D_5__Holdemania	0.00(0.00,Inf)	0.00(0.00,Inf)	0.06(0.00,2.29)						
ASV_981	Firmicutes;D_2__Clostridia;D_3__Clostridiales;D_4__Family XI;D_5__Anaerococcus	2.09(0.17,25.61)	0.66(0.05,8.56)	0.32(0.03,3.56)	1.17(0.09,15.51)	1.00(0.08,13.08)	0.85(0.08,9.37)			
ASV_984	Firmicutes;D_2__Clostridia;D_3__Clostridiales;D_4__Lachnospiraceae;D_5__Marvinbryantia;D_6__uncultured bacterium;D_7__;D_8__;D_9__;D_10__;D_11__;D_12__;D_13__;D_14__	3.46(0.30,40.09)	2.33(0.19,28.96)	0.67(0.06,7.27)						
ASV_986	Firmicutes;D_2__Clostridia;D_3__Clostridiales;D_4__Ruminococcaceae;D_5__Ruminiclostridium 5;D_6__uncultured bacterium;D_7__;D_8__;D_9__;D_10__;D_11__;D_12__;D_13__;D_14__	0.65(0.15,2.81)	0.55(0.12,2.48)	0.85(0.21,3.40)	0.61(0.13,2.84)	0.51(0.11,2.34)	0.83(0.21,3.37)			
ASV_1003	Firmicutes;D_2__Clostridia;D_3__Clostridiales;D_4__Ruminococcaceae;D_5__Fournierella;D_6__uncultured organism;D_7__;D_8__;D_9__;D_10__;D_11__;D_12__;D_13__;D_14__	0.46(0.03,6.63)	0.29(0.02,4.53)	0.64(0.05,7.98)	0.69(0.04,12.08)	0.10(0.01,1.63)	0.15(0.01,1.88)			
ASV_1005	Firmicutes;D_2__Clostridia;D_3__Clostridiales;D_4__Ruminococcaceae				0.00(0.00,Inf)	0.00(0.00,Inf)	0.50(0.11,2.36)			
ASV_1009	Firmicutes;D_2__Clostridia;D_3__Clostridiales;D_4__Ruminococcaceae;D_5__GCA-900066225;D_6__uncultured bacterium;D_7__;D_8__;D_9__;D_10__;D_11__;D_12__;D_13__;D_14__	3.47(0.33,36.79)	0.87(0.08,9.32)	0.25(0.03,2.45)	7.77(0.63,96.67)	0.78(0.07,8.08)	0.10(0.01,1.02)			
ASV_1010	Firmicutes;D_2__Clostridia;D_3__Clostridiales;D_4__Ruminococcaceae;D_5__Ruminiclostridium 5;D_6__uncultured organism;D_7__;D_8__;D_9__;D_10__;D_11__;D_12__;D_13__;D_14__	5.00(0.40,62.73)	8.01(0.58,111.45)	1.60(0.13,19.76)	6.73(0.51,89.57)	6.38(0.49,83.62)	0.95(0.08,11.35)			
ASV_1013	Actinobacteria;D_2__Actinobacteria;D_3__Corynebacteriales;D_4__Corynebacteriaceae;D_5__Corynebacterium;D_6__Corynebacterium durum;D_7__;D_8__;D_9__;D_10__;D_11__;D_12__;D_13__;D_14__	0.97(0.39,2.39)	0.76(0.30,1.91)	0.78(0.33,1.86)	1.00(0.40,2.54)	0.83(0.33,2.10)	0.83(0.35,1.96)			
ASV_1014	Firmicutes;D_2__Clostridia;D_3__Clostridiales;D_4__Lachnospiraceae;D_5__[Ruminococcus] gauvreauii group				28.56(1.21,676.43);p=0.0379	0.20(0.01,4.26)	0.01(0.00,0.13);p=0.00094	↑		↓
ASV_1016	Firmicutes;D_2__Clostridia;D_3__Clostridiales;D_4__Lachnospiraceae;D_5__Lachnoclostridium				0.00(0.00,Inf)	0.00(0.00,Inf)	0.81(0.03,21.70)			
ASV_1018	Firmicutes;D_2__Clostridia;D_3__Clostridiales;D_4__Christensenellaceae;D_5__Christensenellaceae R-7 group;D_6__uncultured bacterium;D_7__;D_8__;D_9__;D_10__;D_11__;D_12__;D_13__;D_14__	1.11(0.16,7.70)	1.01(0.14,7.41)	0.91(0.14,5.95)	0.70(0.09,5.33)	0.49(0.07,3.58)	0.69(0.11,4.34)			
ASV_1020	Firmicutes;D_2__Erysipelotrichia;D_3__Erysipelotrichales;D_4__Erysipelotrichaceae;D_5__Erysipelatoclostridium;D_6__Massiliomicrobiota timonensis;D_7__;D_8__;D_9__;D_10__;D_11__;D_12__;D_13__;D_14__	0.54(0.06,4.87)	1.60(0.16,15.82)	2.99(0.35,25.58)	0.32(0.03,3.14)	0.90(0.09,8.82)	2.80(0.34,23.17)			

ASV_1023	Firmicutes;D_2__Clostridia;D_3__Clostridiales;D_4__Ruminococcaceae;D_5__Oscillospira;D_6__uncultured bacterium;D_7__;D_8__;D_9__;D_10__;D_11__;D_12__;D_13__;D_14__	0.00(0.00,Inf)	0.00(0.00,Inf)	0.30(0.01,7.49)						
ASV_1024	Firmicutes;D_2__Clostridia;D_3__Clostridiales;D_4__Lachnospiraceae	1.64(0.19,14.57)	1.20(0.13,11.17)	0.73(0.09,6.08)						
ASV_1025	Firmicutes;D_2__Bacilli;D_3__Lactobacillales;D_4__Aerococcaceae;D_5__Abiotrophia;D_6__uncultured bacterium;D_7__;D_8__;D_9__;D_10__;D_11__;D_12__;D_13__;D_14__	2.93(0.38,22.83)	2.75(0.33,22.76)	0.94(0.12,7.17)	2.88(0.35,23.45)	3.30(0.40,27.30)	1.15(0.16,8.48)			
ASV_1029	Firmicutes;D_2__Clostridia;D_3__Clostridiales;D_4__Ruminococcaceae;D_5__uncultured;D_6__uncultured Firmicutes bacterium;D_7__;D_8__;D_9__;D_10__;D_11__;D_12__;D_13__;D_14__	0.29(0.03,3.26)	0.52(0.04,6.55)	1.79(0.20,15.86)	0.26(0.02,3.14)	0.57(0.04,7.49)	2.22(0.25,19.66)			
ASV_1030	Firmicutes;D_2__Clostridia;D_3__Clostridiales;D_4__Ruminococcaceae	1.75(0.29,10.46)	1.40(0.22,8.74)	0.80(0.14,4.56)	1.51(0.23,9.73)	1.29(0.20,8.30)	0.86(0.15,4.92)			
ASV_1031	Actinobacteria;D_2__Actinobacteria;D_3__Bifidobacteriales;D_4__Bifidobacteriaceae;D_5__Scardovia;D_6__unidentified;D_7__;D_8__;D_9__;D_10__;D_11__;D_12__;D_13__;D_14__	4.01(0.34,47.21)	1.09(0.09,12.70)	0.27(0.02,2.97)						
ASV_1033	Firmicutes;D_2__Clostridia;D_3__Clostridiales;D_4__Lachnospiraceae	0.00(0.00,Inf)	0.00(0.00,Inf)	0.21(0.01,3.87)						
ASV_1040	Firmicutes;D_2__Bacilli;D_3__Lactobacillales;D_4__Lactobacillaceae;D_5__Lactobacillus	0.59(0.02,21.98)	0.06(0.00,2.64)	0.11(0.00,3.55)						
ASV_1049	Firmicutes;D_2__Bacilli;D_3__Lactobacillales;D_4__Lactobacillaceae;D_5__Lactobacillus;D_6__Lactobacillus rhamnosus;D_7__;D_8__;D_9__;D_10__;D_11__;D_12__;D_13__;D_14__	0.08(0.01,1.03)	4.31(0.28,65.40)	51.08(4.04,646.58);p=0.00239	0.11(0.01,1.46)	4.61(0.28,75.36)	42.45(3.24,556.74);p=0.00431			↑
ASV_1052	Firmicutes;D_2__Clostridia;D_3__Clostridiales;D_4__Ruminococcaceae;D_5__Anaerofilum;D_6__uncultured bacterium;D_7__;D_8__;D_9__;D_10__;D_11__;D_12__;D_13__;D_14__	2.54(0.55,11.79)	1.38(0.29,6.57)	0.54(0.12,2.40)	2.13(0.44,10.36)	1.55(0.32,7.45)	0.73(0.17,3.20)			
ASV_1055	Firmicutes;D_2__Clostridia;D_3__Clostridiales;D_4__Ruminococcaceae;D_5__GCA-900066225;D_6__uncultured bacterium;D_7__;D_8__;D_9__;D_10__;D_11__;D_12__;D_13__;D_14__	2.92(0.22,39.55)	0.09(0.01,1.17)	0.03(0.00,0.36); p=0.0057						
ASV_1057	Actinobacteria;D_2__Coriobacteriia;D_3__Coriobacteriales;D_4__Coriobacteriaceae;D_5__Collinsella;D_6__uncultured bacterium;D_7__;D_8__;D_9__;D_10__;D_11__;D_12__;D_13__;D_14__	0.84(0.22,3.23)	0.65(0.16,2.58)	0.77(0.21,2.81)	0.63(0.16,2.58)	0.60(0.15,2.42)	0.94(0.26,3.42)			
ASV_1062	Firmicutes;D_2__Clostridia;D_3__Clostridiales;D_4__Ruminococcaceae;D_5__GCA-900066225;D_6__uncultured Ruminococcus sp.;D_7__;D_8__;D_9__;D_10__;D_11__;D_12__;D_13__;D_14__	0.89(0.24,3.31)	1.15(0.29,4.49)	1.29(0.36,4.62)	0.89(0.23,3.55)	1.17(0.29,4.69)	1.31(0.36,4.74)			
ASV_1066	Actinobacteria;D_2__Actinobacteria;D_3__Corynebacteriales;D_4__Corynebacteriaceae;D_5__Corynebacterium 1;D_6__Corynebacterium pseudodiphtheriticum;D_7__;D_8__;D_9__;D_10__;D_11__;D_12__;D_13__;D_14__	0.74(0.01,47.16)	0.98(0.01,73.41)	1.32(0.02,72.09)						
ASV_1067	Firmicutes;D_2__Clostridia;D_3__Clostridiales;D_4__Ruminococcaceae;D_5__Ruminococcaceae UCG-005;D_6__uncultured Clostridiales bacterium;D_7__;D_8__;D_9__;D_10__;D_11__;D_12__;D_13__;D_14__	0.19(0.00,10.55)	0.98(0.01,68.11)	5.13(0.11,244.07)	0.31(0.01,15.16)	0.19(0.00,8.18)	0.61(0.02,20.93)			
ASV_1070	Firmicutes;D_2__Clostridia;D_3__Clostridiales;D_4__Lachnospiraceae;D_5__Lactonifactor	2.34(0.19,29.35)	1.98(0.15,26.44)	0.84(0.07,10.11)						
ASV_1072	Firmicutes;D_2__Clostridia;D_3__Clostridiales;D_4__Lachnospiraceae;D_5__Lachnoclostridium	0.18(0.02,1.36)	0.32(0.04,2.64)	1.82(0.27,12.43)	0.30(0.04,2.45)	0.33(0.04,2.65)	1.09(0.16,7.33)			
ASV_1091	Firmicutes;D_2__Clostridia;D_3__Clostridiales;D_4__Lachnospiraceae;D_5__[Eubacterium] hallii group	0.00(0.00,Inf)	0.00(0.00,Inf)	2.50(0.09,67.93)	0.00(0.00,Inf)	0.00(0.00,Inf)	0.86(0.03,22.74)			
ASV_1092	Firmicutes;D_2__Clostridia;D_3__Clostridiales;D_4__Lachnospiraceae;D_5__[Eubacterium] hallii group	0.12(0.00,3.09)	2.53(0.06,115.72)	20.75(0.67,646.63)						
ASV_1096	Firmicutes;D_2__Clostridia;D_3__Clostridiales;D_4__Lachnospiraceae;D_5__Lachnospiraceae UCG-004	1.91(0.10,36.81)	0.31(0.02,5.67)	0.16(0.01,2.65)	8.86(0.31,250.05)	0.37(0.02,6.82)	0.04(0.00,0.88);p=0.04099			↓

ASV_1097	Firmicutes;D_2__Clostridia;D_3__Clostridiales;D_4__Lachnospiraceae	0.00(0.00,Inf)	0.00(0.00,Inf)	0.78(0.07,9.04)						
ASV_1106	Firmicutes;D_2__Clostridia;D_3__Clostridiales;D_4__Ruminococcaceae;D_5__Faecalibacterium;D_6__uncultured bacterium;D_7__;D_8__;D_9__;D_10__;D_11__;D_12__;D_13__;D_14__	28924338907867.00(0.00,Inf)	0.61(0.02,19.81)	0.00(0.00,Inf)							
ASV_1108	Firmicutes;D_2__Clostridia;D_3__Clostridiales;D_4__Lachnospiraceae;D_5__Lachnospiraceae UCG-004	0.68(0.02,30.21)	104074133394481.00(0.00,Inf)	152371488318117.00(0.00,Inf)							
ASV_1112	Firmicutes;D_2__Clostridia;D_3__Clostridiales;D_4__Lachnospiraceae;D_5__Anaerostipes;D_6__uncultured bacterium;D_7__;D_8__;D_9__;D_10__;D_11__;D_12__;D_13__;D_14__	4.30(0.22,84.35)	1.55(0.12,19.54)	0.36(0.02,7.30)							
ASV_1132	Firmicutes;D_2__Bacilli;D_3__Lactobacillales;D_4__Streptococcaceae;D_5__Streptococcus	98.75(4.46,2186.85);p=0.00366	16.61(0.86,321.30)	0.17(0.01,3.61)							
ASV_1133	Firmicutes;D_2__Clostridia;D_3__Clostridiales;D_4__Family XI;D_5__Parvimonas	4.13(0.28,60.58)	0.36(0.03,4.87)	0.09(0.01,1.14)	9.64(0.54,173.50)	0.62(0.05,8.55)	0.06(0.00,0.93);p=0.04397			↓
ASV_1134	Firmicutes;D_2__Clostridia;D_3__Clostridiales;D_4__Family XI;D_5__Ezakiella	0.26(0.01,7.91)	0.15(0.00,4.85)	0.57(0.02,13.07)							
ASV_1142	Firmicutes;D_2__Clostridia;D_3__Clostridiales;D_4__Christensenellaceae;D_5__Christensenellaceae R-7 group;D_6__uncultured bacterium;D_7__;D_8__;D_9__;D_10__;D_11__;D_12__;D_13__;D_14__	0.21(0.01,6.61)	0.98(0.03,37.82)	4.63(0.17,125.69)							
ASV_1143	Firmicutes;D_2__Erysipelotrichia;D_3__Erysipelotrichales;D_4__Erysipelotrichaceae;D_5__Coprobacillus;D_6__uncultured bacterium;D_7__;D_8__;D_9__;D_10__;D_11__;D_12__;D_13__;D_14__	1.06(0.19,5.81)	0.27(0.05,1.55)	0.26(0.05,1.32)	0.90(0.15,5.25)	0.31(0.05,1.82)	0.35(0.07,1.79)			
ASV_1145	Bacteroidetes;D_2__Bacteroidia;D_3__Bacteroidales;D_4__Porphyromonadaceae;D_5__Porphyromonas;D_6__unidentified;D_7__;D_8__;D_9__;D_10__;D_11__;D_12__;D_13__;D_14__				0.00(0.00,Inf)	0.00(0.00,Inf)	0.76(0.04,13.11)			
ASV_1147	Firmicutes;D_2__Bacilli;D_3__Lactobacillales;D_4__Streptococcaceae;D_5__Streptococcus	2.52(0.24,26.74)	3.18(0.28,36.47)	1.26(0.13,12.65)							
ASV_1149	Firmicutes;D_2__Clostridia;D_3__Clostridiales;D_4__Ruminococcaceae	1.76(0.05,59.57)	0.42(0.01,13.98)	0.24(0.01,6.76)	16.60(0.48,573.59)	7.68(0.27,221.83)	0.46(0.02,12.29)			
ASV_1151	Firmicutes;D_2__Negativicutes;D_3__Selenomonadales;D_4__Veillonellaceae;D_5__Dialister;D_6__uncultured bacterium;D_7__;D_8__;D_9__;D_10__;D_11__;D_12__;D_13__;D_14__	0.11(0.00,8.26)	1.02(0.01,119.40)	9.35(0.15,587.13)							
ASV_1155	Actinobacteria;D_2__Actinobacteria;D_3__Corynebacteriales;D_4__Corynebacteriaceae;D_5__Lawsonella;D_6__uncultured bacterium;D_7__;D_8__;D_9__;D_10__;D_11__;D_12__;D_13__;D_14__	1.10(0.07,17.33)	0.19(0.01,2.69)	0.17(0.01,2.05)	1.04(0.05,20.30)	0.13(0.01,2.13)	0.13(0.01,1.63)			
ASV_1160	Firmicutes;D_2__Clostridia;D_3__Clostridiales;D_4__Ruminococcaceae;D_5__Candidatus Soleaferrea;D_6__uncultured bacterium;D_7__;D_8__;D_9__;D_10__;D_11__;D_12__;D_13__;D_14__	1.37(0.21,9.15)	1.23(0.18,8.59)	0.89(0.14,5.67)	1.97(0.27,14.34)	1.22(0.17,8.54)	0.62(0.10,3.94)			
ASV_1162	Firmicutes;D_2__Clostridia;D_3__Clostridiales;D_4__Ruminococcaceae;D_5__[Eubacterium] coprostanoligenes group;D_6__uncultured organism;D_7__;D_8__;D_9__;D_10__;D_11__;D_12__;D_13__;D_14__	3.00(0.13,67.06)	0.64(0.03,13.42)	0.21(0.01,4.20)							
ASV_1164	Actinobacteria;D_2__Actinobacteria;D_3__Actinomycetales;D_4__Actinomycetaceae;D_5__Actinomyces	11.22(0.88,142.36)	1.55(0.14,17.76)	0.14(0.01,1.65)	11.51(0.83,159.75)	2.25(0.19,26.68)	0.20(0.02,2.31)			
ASV_1189	Firmicutes;D_2__Clostridia;D_3__Clostridiales;D_4__Ruminococcaceae	0.37(0.02,6.78)	0.18(0.01,3.19)	0.48(0.05,4.97)	0.28(0.01,6.52)	0.14(0.01,2.77)	0.48(0.04,5.17)			
ASV_1193	Firmicutes;D_2__Clostridia;D_3__Clostridiales;D_4__Ruminococcaceae;D_5__Ruminococcaceae UCG-004;D_6__uncultured bacterium;D_7__;D_8__;D_9__;D_10__;D_11__;D_12__;D_13__;D_14__	1.45(0.04,49.18)	2.74(0.07,109.40)	1.89(0.06,62.08)							
ASV_1196	Firmicutes;D_2__Clostridia;D_3__Clostridiales;D_4__Ruminococcaceae;D_5__uncultured				3.13(0.24,41.02)	0.75(0.07,8.57)	0.24(0.02,2.59)			

ASV_1201	Cyanobacteria;D_2__Oxyphotobacteria;D_3__Chloroplast	0.08(0.00,2.62)	1.81(0.05,67.71)	23.00(0.77,688.05)						
ASV_1205	Firmicutes;D_2__Clostridia;D_3__Clostridiales;D_4__Ruminococcaceae	7.37(0.15,359.53)	3.57(0.08,151.14)	0.48(0.01,26.08)						
ASV_1207	Firmicutes;D_2__Clostridia;D_3__Clostridiales;D_4__Family XIII;D_5__[Eubacterium] brachy group;D_6__uncultured Eubacteriaceae bacterium;D_7__;D_8__;D_9__;D_10__;D_11__;D_12__;D_13__;D_14__	0.18(0.01,3.02)	0.11(0.01,1.95)	0.62(0.06,6.63)	0.11(0.01,2.29)	0.09(0.00,1.83)	0.83(0.08,8.70)			
ASV_1208	Firmicutes;D_2__Erysipelotrichia;D_3__Erysipelotrichales;D_4__Erysipelotrichaceae;D_5__[Clostridium] innocuum group	0.00(0.00,Inf)	0.00(0.00,Inf)	3.96(0.47,33.21)	0.00(0.00,Inf)	0.00(0.00,Inf)	2.72(0.35,21.08)			
ASV_1209	Firmicutes;D_2__Clostridia;D_3__Clostridiales;D_4__Ruminococcaceae;D_5__Papillibacter;D_6__uncultured bacterium;D_7__;D_8__;D_9__;D_10__;D_11__;D_12__;D_13__;D_14__	2.11(0.53,8.37)	0.92(0.23,3.62)	0.44(0.12,1.65)	2.09(0.49,8.90)	0.85(0.21,3.41)	0.41(0.11,1.56)			
ASV_1210	Firmicutes;D_2__Clostridia;D_3__Clostridiales;D_4__Family XIII;D_5__[Eubacterium] nodatum group;D_6__[Eubacterium] sulci;D_7__;D_8__;D_9__;D_10__;D_11__;D_12__;D_13__;D_14__				0.48(0.07,3.49)	0.35(0.05,2.48)	0.73(0.13,4.21)			
ASV_1211	Firmicutes;D_2__Clostridia;D_3__Clostridiales;D_4__Ruminococcaceae;D_5__Candidatus Soleaferrea;D_6__uncultured bacterium;D_7__;D_8__;D_9__;D_10__;D_11__;D_12__;D_13__;D_14__	2.06(0.24,17.72)	1.96(0.21,17.95)	0.95(0.11,8.06)	1.96(0.20,19.12)	0.96(0.11,8.41)	0.49(0.06,4.11)			
ASV_1213	Firmicutes;D_2__Clostridia;D_3__Clostridiales;D_4__Ruminococcaceae;D_5__Butyricicoccus	20.93(0.59,739.28)	1008684855838.46(0.00,Inf)	48204369615.07(0.00,Inf)						
ASV_1214	Actinobacteria;D_2__Actinobacteria;D_3__Actinomycetales;D_4__Actinomycetaceae;D_5__Varibaculum	0.52(0.06,4.93)	0.36(0.04,3.57)	0.69(0.08,5.63)	0.41(0.04,4.28)	0.19(0.02,1.91)	0.47(0.06,3.60)			
ASV_1215	Firmicutes;D_2__Clostridia;D_3__Clostridiales;D_4__Lachnospiraceae;D_5__Lachnospiraceae FCS020 group;D_6__uncultured bacterium;D_7__;D_8__;D_9__;D_10__;D_11__;D_12__;D_13__;D_14__	0.95(0.06,16.40)	2.76(0.13,56.68)	2.90(0.17,49.95)	0.66(0.03,13.04)	2.50(0.11,55.83)	3.78(0.21,66.90)			
ASV_1216	Firmicutes;D_2__Clostridia;D_3__Clostridiales;D_4__Ruminococcaceae	0.00(0.00,Inf)	0.00(0.00,Inf)	3.36(0.26,42.65)						
ASV_1217	Firmicutes;D_2__Clostridia;D_3__Clostridiales;D_4__Christensenellaceae;D_5__Christensenellaceae R-7 group	2.07(0.36,11.93)	2.66(0.42,16.84)	1.28(0.21,7.84)	2.03(0.32,12.84)	2.60(0.40,16.99)	1.28(0.21,7.84)			
ASV_1220	Bacteroidetes;D_2__Bacteroidia;D_3__Bacteroidales;D_4__Prevotellaceae;D_5__Prevotella;D_6__Chlamydia trachomatis;D_7__;D_8__;D_9__;D_10__;D_11__;D_12__;D_13__;D_14__	0.02(0.00,0.33);p=0.00633	0.38(0.02,7.07)	18.87(1.38,257.82);p=0.02768						
ASV_1222	Firmicutes;D_2__Bacilli;D_3__Lactobacillales;D_4__Lactobacillaceae;D_5__Lactobacillus;D_6__Lactobacillus rhamnosus;D_7__;D_8__;D_9__;D_10__;D_11__;D_12__;D_13__;D_14__	0.34(0.01,8.98)	2.86(0.06,135.93)	8.40(0.25,287.68)	0.22(0.01,7.74)	1.86(0.04,92.05)	8.43(0.25,283.41)			
ASV_1223	Firmicutes;D_2__Clostridia;D_3__Clostridiales;D_4__Ruminococcaceae	0.75(0.03,16.82)	0.61(0.03,14.74)	0.81(0.04,15.65)						
ASV_1226	Firmicutes;D_2__Clostridia;D_3__Clostridiales;D_4__Christensenellaceae;D_5__Christensenellaceae R-7 group	2.79(0.18,43.38)	0.20(0.01,3.08)	0.07(0.01,0.99);p=0.04885	2.55(0.14,44.96)	0.21(0.01,3.43)	0.08(0.01,1.16)			
ASV_1227	Firmicutes;D_2__Clostridia;D_3__Clostridiales;D_4__Ruminococcaceae;D_5__Ruminiclostridium 5;D_6__uncultured bacterium;D_7__;D_8__;D_9__;D_10__;D_11__;D_12__;D_13__;D_14__	3.52(0.42,29.57)	0.30(0.04,2.10)	0.09(0.01,0.64);p=0.01623						
ASV_1228	Firmicutes;D_2__Clostridia;D_3__Clostridiales;D_4__Christensenellaceae;D_5__uncultured;D_6__uncultured bacterium;D_7__;D_8__;D_9__;D_10__;D_11__;D_12__;D_13__;D_14__	0.41(0.04,3.96)	0.25(0.02,2.47)	0.60(0.08,4.67)						
ASV_1229	Firmicutes;D_2__Clostridia;D_3__Clostridiales;D_4__Ruminococcaceae;D_5__uncultured bacterium;D_6__;D_7__;D_8__;D_9__;D_10__;D_11__;D_12__;D_13__;D_14__	0.99(0.14,6.85)	0.60(0.09,4.17)	0.61(0.10,3.67)	1.39(0.19,10.42)	0.75(0.11,5.22)	0.54(0.09,3.33)			
ASV_1231	Firmicutes;D_2__Clostridia;D_3__Clostridiales;D_4__Lachnospiraceae	11.50(0.92,144.47)	20154294236.46(0.00,Inf)	1752047847.92(0.00,Inf)						

ASV_1258	Firmicutes;D_2__Clostridia;D_3__Clostridiales;D_4__Ruminococcaceae;D_5__Hydrogenoanaerobacterium;D_6__uncultured bacterium;D_7__;D_8__;D_9__;D_10__;D_11__;D_12__;D_13__;D_14__	0.63(0.04,9.58)	1.44(0.07,28.49)	2.29(0.15,34.79)	0.94(0.05,16.44)	1.12(0.06,19.49)	1.19(0.08,17.78)			
ASV_1259	Firmicutes;D_2__Clostridia;D_3__Clostridiales;D_4__Ruminococcaceae;D_5__GCA-900066225;D_6__uncultured bacterium;D_7__;D_8__;D_9__;D_10__;D_11__;D_12__;D_13__;D_14__	2.24(0.06,83.40)	2.00(0.05,80.81)	0.89(0.02,34.79)						
ASV_1282	Firmicutes;D_2__Clostridia;D_3__Clostridiales;D_4__Lachnospiraceae	0.48(0.08,2.98)	0.39(0.06,2.51)	0.80(0.14,4.54)	1.34(0.20,8.79)	0.49(0.08,3.18)	0.37(0.07,2.09)			
ASV_1284	Firmicutes;D_2__Clostridia;D_3__Clostridiales;D_4__Family XIII;D_5__[Eubacterium] brachy group;D_6__Eubacterium brachy ATCC 33089;D_7__;D_8__;D_9__;D_10__;D_11__;D_12__;D_13__;D_14__	4.28(0.58,31.79)	0.94(0.13,6.86)	0.22(0.03,1.53)						
ASV_1297	Firmicutes;D_2__Clostridia;D_3__Clostridiales;D_4__Peptococcaceae;D_5__Peptococcus;D_6__uncultured organism;D_7__;D_8__;D_9__;D_10__;D_11__;D_12__;D_13__;D_14__	0.00(0.00,Inf)	0.00(0.00,Inf)	0.25(0.01,5.81)						
ASV_1301	Firmicutes;D_2__Clostridia;D_3__Clostridiales;D_4__Ruminococcaceae;D_5__Candidatus Soleaferrea	2.51(0.20,31.29)	2.42(0.18,32.51)	0.96(0.08,11.85)						
ASV_1303	Firmicutes;D_2__Clostridia;D_3__Clostridiales;D_4__Ruminococcaceae;D_5__uncultured	2.23(0.34,14.85)	2.09(0.30,14.70)	0.94(0.15,5.96)	1.66(0.23,11.81)	1.77(0.25,12.53)	1.07(0.17,6.74)			
ASV_1306	Actinobacteria;D_2__Actinobacteria;D_3__Actinomycetales;D_4__Actinomycetaceae;D_5__F0332;D_6__uncultured bacterium;D_7__;D_8__;D_9__;D_10__;D_11__;D_12__;D_13__;D_14__	0.30(0.05,1.67)	0.31(0.05,1.77)	1.01(0.22,4.78)	0.48(0.09,2.61)	0.48(0.09,2.59)	1.00(0.22,4.50)			
ASV_1323	Firmicutes;D_2__Clostridia;D_3__Clostridiales;D_4__Lachnospiraceae;D_5__[Eubacterium] eligens group	0.19(0.01,5.41)	0.05(0.00,1.68)	0.28(0.01,7.36)	0.06(0.00,1.91)	0.01(0.00,0.35);p=0.01012	0.19(0.01,4.08)		↓	
ASV_1329	Patescibacteria;D_2__Saccharimonadia;D_3__Saccharimonadales;D_4__Saccharimonadaceae;D_5__uncultured Candidatus Saccharibacteria bacterium;D_6__;D_7__;D_8__;D_9__;D_10__;D_11__;D_12__;D_13__;D_14__	5.37(0.88,32.71)	3.42(0.56,20.75)	0.64(0.10,4.02)	4.94(0.74,32.83)	3.19(0.51,19.92)	0.65(0.10,4.15)			
ASV_1333	Actinobacteria;D_2__Actinobacteria;D_3__Bifidobacteriales;D_4__Bifidobacteriaceae;D_5__Bifidobacterium	1.26(0.19,8.18)	2.30(0.32,16.71)	1.83(0.28,11.98)						
ASV_1336	Actinobacteria;D_2__Actinobacteria;D_3__Corynebacteriales;D_4__Corynebacteriaceae;D_5__Corynebacterium 1	59.61(1.99,1785.60);p=0.01844	8.83(0.41,190.62)	0.15(0.00,4.42)						
ASV_1343	Firmicutes;D_2__Clostridia;D_3__Clostridiales;D_4__Lachnospiraceae;D_5__Dorea	5.42(0.17,175.23)	697319976202.32(0.00,Inf)	128539823903.78(0.00,Inf)						
ASV_1359	Firmicutes;D_2__Clostridia;D_3__Clostridiales;D_4__Ruminococcaceae;D_5__Ruminococcaceae UCG-010;D_6__uncultured bacterium;D_7__;D_8__;D_9__;D_10__;D_11__;D_12__;D_13__;D_14__	1.85(0.05,74.37)	0.27(0.01,11.44)	0.15(0.00,5.07)						
ASV_1361	Firmicutes;D_2__Clostridia;D_3__Clostridiales;D_4__Lachnospiraceae;D_5__Lachnoclostridium;D_6__Eubacterium sp. Marseille-P3202;D_7__;D_8__;D_9__;D_10__;D_11__;D_12__;D_13__;D_14__	0.02(0.00,0.35);p=0.00711	0.08(0.00,1.35)	3.52(0.29,43.15)	0.02(0.00,0.32);p=0.00709	0.04(0.00,0.78);p=0.03387	2.56(0.22,29.82)	↓	↓	
ASV_1362	Actinobacteria;D_2__Actinobacteria;D_3__Micrococcales;D_4__Micrococcaceae;D_5__Rothia	0.32(0.04,2.95)	0.48(0.05,4.68)	1.48(0.18,12.25)	0.80(0.09,7.16)	1.62(0.17,14.98)	2.03(0.26,15.98)			
ASV_1363	Firmicutes;D_2__Clostridia;D_3__Clostridiales;D_4__Christensenellaceae;D_5__Christensenellaceae R-7 group	5.78(0.39,86.32)	2.32(0.15,37.20)	0.40(0.03,5.54)						
ASV_1372	Firmicutes;D_2__Clostridia;D_3__Clostridiales;D_4__Ruminococcaceae;D_5__Subdoligranulum	0.80(0.04,17.73)	1.04(0.04,26.52)	1.29(0.07,25.29)	0.96(0.03,36.82)	0.28(0.01,8.03)	0.30(0.02,5.75)			
ASV_1378	Firmicutes;D_2__Erysipelotrichia;D_3__Erysipelotrichales;D_4__Erysipelotrichaceae;D_5__Merdibacter;D_6__uncultured bacterium;D_7__;D_8__;D_9__;D_10__;D_11__;D_12__;D_13__;D_14__	0.96(0.18,5.26)	0.62(0.11,3.56)	0.64(0.13,3.33)						
ASV_1393	Firmicutes;D_2__Clostridia;D_3__Clostridiales;D_4__Ruminococcaceae;D_5__Intestinimonas;D_6__uncultured Clostridiales bacterium;D_7__;D_8__;D_9__;D_10__;D_11__;D_12__;D_13__;D_14__	0.96(0.03,32.36)	0.09(0.00,3.24)	0.10(0.00,2.68)	0.69(0.02,29.28)	0.06(0.00,2.20)	0.08(0.00,2.32)			

ASV_1399	Firmicutes;D_2__Clostridia;D_3__Clostridiales;D_4__Ruminococcaceae;D_5__GCA-900066225;D_6__uncultured bacterium;D_7__;D_8__;D_9__;D_10__;D_11__;D_12__;D_13__;D_14__	1.34(0.06,32.35)	1.26(0.05,33.61)	0.94(0.04,20.70)						
ASV_1408	Firmicutes;D_2__Clostridia;D_3__Clostridiales;D_4__Lachnospiraceae	0.59(0.04,8.22)	0.83(0.05,12.70)	1.40(0.11,17.34)						
ASV_1409	Firmicutes;D_2__Clostridia;D_3__Clostridiales;D_4__Ruminococcaceae	1.50(0.05,41.81)	0.46(0.02,10.96)	0.31(0.01,6.56)						
ASV_1416	Firmicutes;D_2__Clostridia;D_3__Clostridiales;D_4__Ruminococcaceae;D_5__[Eubacterium] coprostanoligenes group;D_6__uncultured organism;D_7__;D_8__;D_9__;D_10__;D_11__;D_12__;D_13__;D_14__	0.43(0.03,5.87)	0.27(0.02,3.97)	0.63(0.05,7.63)	1.04(0.07,14.98)	0.21(0.02,2.74)	0.20(0.02,2.23)			
ASV_1418	Firmicutes;D_2__Clostridia;D_3__Clostridiales;D_4__Lachnospiraceae;D_5__Lachnospiraceae FCS020 group;D_6__metagenome;D_7__;D_8__;D_9__;D_10__;D_11__;D_12__;D_13__;D_14__	0.20(0.01,2.68)	0.11(0.01,1.51)	0.56(0.08,3.97)	0.14(0.01,2.39)	0.08(0.01,1.29)	0.58(0.08,4.20)			
ASV_1420	Firmicutes;D_2__Clostridia;D_3__Clostridiales;D_4__Christensenellaceae;D_5__Christensenellaceae R-7 group;D_6__uncultured organism;D_7__;D_8__;D_9__;D_10__;D_11__;D_12__;D_13__;D_14__				0.00(0.00,Inf)	0.00(0.00,Inf)	0.76(0.03,18.50)			
ASV_1421	Firmicutes;D_2__Clostridia;D_3__Clostridiales;D_4__Ruminococcaceae;D_5__Butyricicoccus	1.30(0.02,97.14)	0.48(0.01,35.71)	0.37(0.01,21.71)						
ASV_1422	Firmicutes;D_2__Clostridia;D_3__Clostridiales;D_4__Lachnospiraceae;D_5__Lachnoclostridium	0.40(0.02,10.46)	0.30(0.01,8.61)	0.75(0.03,16.85)	3.30(0.15,71.37)	6.06(0.25,144.64)	1.84(0.08,39.99)			
ASV_1464	Firmicutes;D_2__Clostridia;D_3__Clostridiales;D_4__Ruminococcaceae;D_5__Subdoligranulum;D_6__uncultured bacterium;D_7__;D_8__;D_9__;D_10__;D_11__;D_12__;D_13__;D_14__	2788517933921.80(0.00,Inf)	0.54(0.04,6.65)	0.00(0.00,Inf)	1045489958304624.00(0.00,Inf)	0.53(0.04,7.19)	0.00(0.00,Inf)			
ASV_1493	Firmicutes;D_2__Clostridia;D_3__Clostridiales;D_4__Ruminococcaceae;D_5__Oscillibacter;D_6__uncultured bacterium;D_7__;D_8__;D_9__;D_10__;D_11__;D_12__;D_13__;D_14__	0.48(0.03,7.59)	0.14(0.01,2.14)	0.28(0.02,3.27)						
ASV_1500	Firmicutes;D_2__Clostridia;D_3__Clostridiales;D_4__Ruminococcaceae	0.14(0.00,4.52)	0.48(0.01,18.70)	3.47(0.14,85.45)						
ASV_1503	Firmicutes;D_2__Clostridia;D_3__Clostridiales;D_4__Christensenellaceae;D_5__Christensenellaceae R-7 group	4.64(0.09,236.61)	0.78(0.02,35.85)	0.17(0.00,7.51)						
ASV_1510	Epsilonbacteraeota;D_2__Campylobacteria;D_3__Campylobacterales;D_4__Campylobacteraceae;D_5__Campylobacter;D_6__unidentified;D_7__;D_8__;D_9__;D_10__;D_11__;D_12__;D_13__;D_14__	0.00(0.00,Inf)	0.00(0.00,Inf)	0.72(0.04,14.32)	0.00(0.00,Inf)	0.00(0.00,Inf)	0.44(0.02,9.60)			
ASV_1512	Firmicutes;D_2__Clostridia;D_3__Clostridiales;D_4__Christensenellaceae;D_5__Christensenellaceae R-7 group;D_6__Blattella germanica (German cockroach);D_7__;D_8__;D_9__;D_10__;D_11__;D_12__;D_13__;D_14__	0.69(0.05,10.07)	1.18(0.07,19.25)	1.71(0.13,23.05)						
ASV_1520	Actinobacteria;D_2__Actinobacteria;D_3__Bifidobacteriales;D_4__Bifidobacteriaceae;D_5__Bifidobacterium	0.74(0.09,6.15)	0.22(0.03,1.63)	0.30(0.05,1.68)	0.64(0.06,6.27)	0.16(0.02,1.31)	0.25(0.04,1.49)			
ASV_1526	Firmicutes;D_2__Clostridia;D_3__Clostridiales;D_4__Lachnospiraceae;D_5__uncultured;D_6__intestinal bacterium CG19-1;D_7__;D_8__;D_9__;D_10__;D_11__;D_12__;D_13__;D_14__	1.42(0.18,11.15)	1.92(0.23,16.30)	1.36(0.18,10.22)						
ASV_1528	Firmicutes;D_2__Clostridia;D_3__Clostridiales;D_4__Ruminococcaceae;D_5__Ruminiclostridium 9;D_6__Colidextribacter massiliensis;D_7__;D_8__;D_9__;D_10__;D_11__;D_12__;D_13__;D_14__	3.96(0.33,46.95)	0.80(0.07,9.51)	0.20(0.02,2.21)						
ASV_1532	Bacteroidetes;D_2__Bacteroidia;D_3__Bacteroidales;D_4__Bacteroidaceae;D_5__Bacteroides	0.00(0.00,Inf)	0.00(0.00,Inf)	0.36(0.01,10.33)						
ASV_1543	Proteobacteria;D_2__Gammaproteobacteria;D_3__Xanthomonadales;D_4__Xanthomonadaceae;D_5__Stenotrophomonas	8.50(0.61,119.13)	2.81(0.21,37.32)	0.33(0.02,4.51)						
ASV_1548	Proteobacteria;D_2__Alphaproteobacteria;D_3__Rhizobiales;D_4__Rhizobiaceae;D_5__Allorhizobium-Neorhizobium-Pararhizobium-Rhizobium	1304410241808.97(0.00,Inf)	12.25(1.48,101.31);p=0.02012	0.00(0.00,Inf)	13089477662639464.00(0.00,Inf)	21.49(2.17,212.95);p=0.00875	0.00(0.00,Inf)		↑	

ASV_1550	Firmicutes;D_2__Bacilli;D_3__Bacillales;D_4__Alicyclobacillaceae;D_5__Effusibacillus;D_6__uncultured organism;D_7__;D_8__;D_9__;D_10__;D_11__;D_12__;D_13__;D_14__	70804990340854.91(0.00,Inf)	0.26(0.01,5.37)	0.00(0.00,Inf)						
ASV_1552	Firmicutes;D_2__Clostridia;D_3__Clostridiales;D_4__Lachnospiraceae;D_5__Blautia				0.03(0.00,0.46);p=0.01196	0.63(0.04,9.88)	21.40(1.68,272.36);p=0.01826	↓		↑
ASV_1556	Firmicutes;D_2__Clostridia;D_3__Clostridiales;D_4__Lachnospiraceae;D_5__Lachnoclostridium	3.19(0.33,30.57)	23.10(1.79,298.75);p=0.0162	7.24(0.62,84.64)	4.69(0.60,36.81)	7.06(0.81,61.59)	1.50(0.19,11.82)			
ASV_1569	Firmicutes;D_2__Bacilli;D_3__Lactobacillales;D_4__Leuconostocaceae;D_5__Leuconostoc;D_6__uncultured bacterium;D_7__;D_8__;D_9__;D_10__;D_11__;D_12__;D_13__;D_14__	1.24(0.07,21.49)	0.05(0.00,0.75);p=0.03102	0.04(0.00,0.52);p=0.01421	10.60(0.49,229.00)	0.33(0.03,4.42)	0.03(0.00,0.52);p=0.01532			↓
ASV_1570	Bacteroidetes;D_2__Bacteroidia;D_3__Bacteroidales;D_4__Bacteroidaceae;D_5__Bacteroides	0.88(0.18,4.41)	8083552476.25(0.00,Inf)	9197549454.34(0.00,Inf)	0.57(0.10,3.24)	1750069557594987.00(0.00,Inf)	3054974664527147.00(0.00,Inf)			
ASV_1575	Firmicutes;D_2__Clostridia;D_3__Clostridiales;D_4__Lachnospiraceae;D_5__Blautia	1.20(0.10,14.94)	776952752676385.00(0.00,Inf)	648389706546647.00(0.00,Inf)	0.55(0.03,9.24)	366328733310483.00(0.00,Inf)	669376484803630.00(0.00,Inf)			
ASV_1576	Firmicutes;D_2__Clostridia;D_3__Clostridiales;D_4__Lachnospiraceae;D_5__Tyzzerella 4;D_6__uncultured organism;D_7__;D_8__;D_9__;D_10__;D_11__;D_12__;D_13__;D_14__	1.48(0.20,11.29)	1.56(0.19,12.61)	1.05(0.15,7.50)	1.49(0.18,12.52)	1.57(0.19,13.19)	1.06(0.14,7.70)			
ASV_1585	Firmicutes;D_2__Bacilli;D_3__Lactobacillales;D_4__Streptococcaceae;D_5__Streptococcus;D_6__Streptococcus agalactiae;D_7__;D_8__;D_9__;D_10__;D_11__;D_12__;D_13__;D_14__	0.35(0.02,7.32)	0.03(0.00,0.56);p=0.01912	0.09(0.01,0.85);p=0.03545						
ASV_1591	Firmicutes;D_2__Clostridia;D_3__Clostridiales;D_4__Ruminococcaceae;D_5__Faecalibacterium				0.04(0.00,1.19)	0.14(0.01,3.65)	3.20(0.22,47.25)			
ASV_1642	Firmicutes;D_2__Clostridia;D_3__Clostridiales;D_4__Family XI;D_5__Anaerococcus	17.70(0.42,743.61)	2.67(0.07,100.30)	0.15(0.00,5.88)						
ASV_1657	Firmicutes;D_2__Clostridia;D_3__Clostridiales;D_4__Family XI;D_5__Peptoniphilus;D_6__Peptoniphilus sp. KHD5;D_7__;D_8__;D_9__;D_10__;D_11__;D_12__;D_13__;D_14__	0.32(0.01,9.68)	0.19(0.01,6.10)	0.58(0.02,14.30)	0.06(0.00,2.39)	0.14(0.00,5.70)	2.34(0.11,52.21)			
ASV_1662	Firmicutes;D_2__Clostridia;D_3__Clostridiales;D_4__Ruminococcaceae;D_5__Candidatus Soleaferrea;D_6__uncultured bacterium;D_7__;D_8__;D_9__;D_10__;D_11__;D_12__;D_13__;D_14__	0.42(0.03,5.33)	0.37(0.03,4.96)	0.87(0.08,9.19)	0.33(0.03,3.95)	0.47(0.04,5.48)	1.41(0.16,12.23)			
ASV_1666	Firmicutes;D_2__Clostridia;D_3__Clostridiales;D_4__Ruminococcaceae;D_5__Ruminococcaceae UCG-014;D_6__uncultured bacterium;D_7__;D_8__;D_9__;D_10__;D_11__;D_12__;D_13__;D_14__	0.20(0.01,7.47)	1.91(0.03,107.06)	9.36(0.24,358.77)						
ASV_1711	Firmicutes;D_2__Clostridia;D_3__Clostridiales;D_4__Ruminococcaceae;D_5__Ruminococcaceae UCG-013;D_6__uncultured bacterium;D_7__;D_8__;D_9__;D_10__;D_11__;D_12__;D_13__;D_14__	3.89(0.25,60.66)	11.44(0.60,216.95)	2.94(0.17,49.51)	4.93(0.28,88.15)	22.70(1.05,492.67);p=0.04672	4.61(0.25,84.97)		↑	
ASV_1714	Firmicutes;D_2__Clostridia;D_3__Clostridiales;D_4__Ruminococcaceae	0.60(0.11,3.29)	0.66(0.11,3.86)	1.11(0.22,5.68)	0.27(0.05,1.63)	0.30(0.05,1.74)	1.10(0.23,5.27)			
ASV_1719	Actinobacteria;D_2__Actinobacteria;D_3__Micrococcales;D_4__Micrococcaceae;D_5__Rothia;D_6__uncultured bacterium;D_7__;D_8__;D_9__;D_10__;D_11__;D_12__;D_13__;D_14__	3.10(0.60,15.85)	1.18(0.24,5.75)	0.38(0.08,1.87)	2.04(0.36,11.53)	0.54(0.11,2.67)	0.27(0.05,1.34)			
ASV_1721	Actinobacteria;D_2__Coriobacteriia;D_3__Coriobacteriales;D_4__Atopobiaceae;D_5__Atopobium;D_6__uncultured bacterium;D_7__;D_8__;D_9__;D_10__;D_11__;D_12__;D_13__;D_14__	2.33(0.27,20.42)	0.48(0.06,4.10)	0.21(0.03,1.64)	1.77(0.18,17.61)	0.32(0.04,2.91)	0.18(0.02,1.48)			
ASV_1725	Firmicutes;D_2__Clostridia;D_3__Clostridiales;D_4__Ruminococcaceae	0.68(0.04,11.76)	4.71(0.22,100.60)	6.97(0.39,124.16)						
ASV_1727	Firmicutes;D_2__Clostridia;D_3__Clostridiales;D_4__Lachnospiraceae	0.03(0.00,0.89);p=0.04283	0.04(0.00,1.34)	1.39(0.08,23.12)						
ASV_1739	Firmicutes;D_2__Clostridia;D_3__Clostridiales;D_4__Ruminococcaceae;D_5__Subdoligranulum;D_6__uncultured bacterium;D_7__;D_8__;D_9__;D_10__;D_11__;D_12__;D_13__;D_14__	0.50(0.06,3.93)	3.26(0.34,31.52)	6.53(0.79,54.24)	0.40(0.05,3.55)	3.26(0.32,33.27)	8.12(0.95,69.70)			

ASV_1742	Firmicutes;D_2__Clostridia;D_3__Clostridiales;D_4__Ruminococcaceae;D_5__Subdoligranulum;D_6__uncultured bacterium;D_7__;D_8__;D_9__;D_10__;D_11__;D_12__;D_13__;D_14__	0.00(0.00,Inf)	0.00(0.00,Inf)	1.15(0.06,22.26)						
ASV_1744	Firmicutes;D_2__Clostridia;D_3__Clostridiales;D_4__Peptococcaceae;D_5__Peptococcus	0.22(0.01,4.10)	0.15(0.01,3.01)	0.70(0.06,8.56)						
ASV_1755	Actinobacteria;D_2__Actinobacteria;D_3__Corynebacteriales;D_4__Corynebacteriaceae;D_5__Corynebacterium 1	2.76(0.18,43.18)	2.69(0.16,45.93)	0.98(0.06,15.76)						
ASV_1780	Firmicutes;D_2__Clostridia;D_3__Clostridiales;D_4__Ruminococcaceae;D_5__Anaerotruncus	0.79(0.07,8.64)	1.02(0.08,12.45)	1.29(0.13,12.91)	0.62(0.05,8.15)	0.55(0.04,6.95)	0.89(0.10,7.63)			
ASV_1811	Actinobacteria;D_2__Coriobacteriia;D_3__Coriobacteriales;D_4__Coriobacteriaceae;D_5__Collinsella	0.16(0.01,2.47)	0.31(0.02,5.13)	1.88(0.17,20.88)						
ASV_1813	Actinobacteria;D_2__Actinobacteria;D_3__Actinomycetales;D_4__Actinomycetaceae;D_5__Varibaculum	0.18(0.01,2.96)	0.07(0.00,1.30)	0.41(0.03,5.42)	0.11(0.01,2.00)	0.27(0.01,4.79)	2.34(0.19,28.18)			
ASV_1815	Firmicutes;D_2__Clostridia;D_3__Clostridiales;D_4__Lachnospiraceae;D_5__Lachnoclostridium	1.22(0.06,24.09)	9255423750.15(0.00,Inf)	7607332219.09(0.00,Inf)						
ASV_1816	Firmicutes;D_2__Clostridia;D_3__Clostridiales;D_4__Lachnospiraceae;D_5__Tyzzerella	0.57(0.02,15.76)	0.80(0.03,25.42)	1.39(0.06,31.53)	0.62(0.01,34.12)	0.17(0.00,7.35)	0.27(0.01,6.35)			
ASV_1817	Firmicutes;D_2__Clostridia;D_3__Clostridiales;D_4__Lachnospiraceae	0.00(0.00,Inf)	0.00(0.00,Inf)	0.40(0.04,4.17)						
ASV_1819	Firmicutes;D_2__Clostridia;D_3__Clostridiales;D_4__Lachnospiraceae	0.58(0.02,15.15)	0.20(0.01,5.71)	0.35(0.02,7.76)						
ASV_1856	Proteobacteria;D_2__Alphaproteobacteria;D_3__Caulobacterales;D_4__Caulobacteraceae;D_5__PMMR1;D_6__uncultured bacterium;D_7__;D_8__;D_9__;D_10__;D_11__;D_12__;D_13__;D_14__	0.67(0.01,35.11)	0.44(0.01,24.83)	0.65(0.02,27.30)						
ASV_1863	Firmicutes;D_2__Clostridia;D_3__Clostridiales;D_4__Ruminococcaceae;D_5__Ruminiclostridium 5;D_6__uncultured bacterium;D_7__;D_8__;D_9__;D_10__;D_11__;D_12__;D_13__;D_14__	0.33(0.02,4.71)	0.20(0.01,2.82)	0.60(0.07,4.89)						
ASV_1872	Firmicutes;D_2__Clostridia;D_3__Clostridiales;D_4__Peptococcaceae;D_5__uncultured;D_6__uncultured organism;D_7__;D_8__;D_9__;D_10__;D_11__;D_12__;D_13__;D_14__	0.05(0.00,3.07)	0.41(0.00,34.45)	8.80(0.21,367.88)						
ASV_1875	Firmicutes;D_2__Clostridia;D_3__Clostridiales;D_4__Ruminococcaceae;D_5__Ruminococcaceae UCG-004;D_6__uncultured bacterium;D_7__;D_8__;D_9__;D_10__;D_11__;D_12__;D_13__;D_14__	0.00(0.00,Inf)	0.00(0.00,Inf)	0.22(0.01,3.46)						
ASV_1878	Firmicutes;D_2__Clostridia;D_3__Clostridiales;D_4__Ruminococcaceae;D_5__Subdoligranulum	0.00(0.00,Inf)	0.00(0.00,Inf)	0.54(0.02,12.68)						
ASV_1880	Actinobacteria;D_2__Coriobacteriia;D_3__Coriobacteriales;D_4__Coriobacteriaceae;D_5__Collinsella	0.32(0.01,19.45)	0.60(0.01,44.68)	1.88(0.04,83.34)						
ASV_1899	Firmicutes;D_2__Clostridia;D_3__Clostridiales;D_4__Lachnospiraceae;D_5__Tyzzerella;D_6__uncultured Firmicutes bacterium;D_7__;D_8__;D_9__;D_10__;D_11__;D_12__;D_13__;D_14__	0.91(0.04,19.93)	3.30(0.11,97.12)	3.63(0.15,87.99)						
ASV_2008	Firmicutes;D_2__Clostridia;D_3__Clostridiales;D_4__Ruminococcaceae;D_5__Faecalibacterium;D_6__uncultured bacterium;D_7__;D_8__;D_9__;D_10__;D_11__;D_12__;D_13__;D_14__	0.62(0.01,28.27)	0.47(0.01,23.44)	0.76(0.02,27.54)						
ASV_2043	Firmicutes;D_2__Clostridia;D_3__Clostridiales;D_4__Lachnospiraceae;D_5__Lachnospiraceae NC2004 group;D_6__uncultured bacterium;D_7__;D_8__;D_9__;D_10__;D_11__;D_12__;D_13__;D_14__	0.00(0.00,Inf)	0.00(0.00,Inf)	1.08(0.11,10.85)	0.00(0.00,Inf)	0.00(0.00,Inf)	1.16(0.11,11.90)			
ASV_2044	Firmicutes;D_2__Clostridia;D_3__Clostridiales;D_4__Lachnospiraceae;D_5__Lachnospiraceae NC2004 group;D_6__uncultured bacterium;D_7__;D_8__;D_9__;D_10__;D_11__;D_12__;D_13__;D_14__	3.88(0.17,91.33)	3.49(0.14,85.77)	0.90(0.03,30.16)						

ASV_2081	Firmicutes;D_2__Clostridia;D_3__Clostridiales;D_4__Lachnospiraceae;D_5__Agathobacter;D_6__uncultured bacterium;D_7__;D_8__;D_9__;D_10__;D_11__;D_12__;D_13__;D_14__				6.00(0.39,92.95)	2.41(0.20,28.96)	0.40(0.03,5.84)			
ASV_2103	Bacteroidetes;D_2__Bacteroidia;D_3__Bacteroidales;D_4__Bacteroidaceae;D_5__Bacteroides	0.46(0.03,8.05)	4024272549293.01(0.00,Inf)	8735427389017.53(0.00,Inf)	0.77(0.05,12.78)	22943917150378.40(0.00,Inf)	29624994034344.10(0.00,Inf)			
ASV_2107	Firmicutes;D_2__Clostridia;D_3__Clostridiales;D_4__Lachnospiraceae	0.00(0.00,Inf)	0.00(0.00,Inf)	1.34(0.10,17.87)	0.00(0.00,Inf)	0.00(0.00,Inf)	0.92(0.07,11.84)			
ASV_2109	Firmicutes;D_2__Clostridia;D_3__Clostridiales;D_4__Ruminococcaceae;D_5__Butyricicoccus;D_6__uncultured bacterium;D_7__;D_8__;D_9__;D_10__;D_11__;D_12__;D_13__;D_14__	11.84(0.32,435.36)	2.08(0.06,77.11)	0.18(0.01,5.86)						
ASV_2110	Firmicutes;D_2__Negativicutes;D_3__Selenomonadales;D_4__Veillonellaceae;D_5__Veillonella	0.01(0.00,0.69);p=0.03189	0.06(0.00,3.29)	4.26(0.11,161.15)						
ASV_2114	Firmicutes;D_2__Clostridia;D_3__Clostridiales;D_4__Eubacteriaceae;D_5__Anaerofustis;D_6__unidentified;D_7__;D_8__;D_9__;D_10__;D_11__;D_12__;D_13__;D_14__	0.36(0.01,9.00)	0.83(0.03,24.15)	2.33(0.11,51.50)	0.81(0.03,20.98)	9.75(0.22,423.24)	12.03(0.36,406.59)			
ASV_2115	Firmicutes;D_2__Clostridia;D_3__Clostridiales;D_4__Lachnospiraceae;D_5__Blautia	0.08(0.00,4.28)	0.50(0.01,34.01)	6.18(0.17,221.62)						
ASV_2117	Bacteroidetes;D_2__Bacteroidia;D_3__Bacteroidales;D_4__Tannerellaceae;D_5__Parabacteroides	0.00(0.00,Inf)	0.00(0.00,Inf)	4.31(0.20,91.33)						
ASV_2134	Firmicutes;D_2__Clostridia;D_3__Clostridiales;D_4__Lachnospiraceae;D_5__Oribacterium;D_6__Oribacterium sinus;D_7__;D_8__;D_9__;D_10__;D_11__;D_12__;D_13__;D_14__	0.78(0.09,6.68)	0.57(0.06,4.99)	0.73(0.10,5.30)						
ASV_2169	Firmicutes;D_2__Clostridia;D_3__Clostridiales;D_4__Lachnospiraceae	0.06(0.00,3.48)	2.15(0.02,193.81)	33.83(0.57,1991.09)						
ASV_2185	Firmicutes;D_2__Clostridia;D_3__Clostridiales;D_4__Lachnospiraceae	0.48(0.02,10.03)	0.53(0.02,12.10)	1.10(0.06,20.00)						
ASV_2186	Firmicutes;D_2__Clostridia;D_3__Clostridiales;D_4__Lachnospiraceae;D_5__Blautia;D_6__Blautia sp. Marseille-P3087;D_7__;D_8__;D_9__;D_10__;D_11__;D_12__;D_13__;D_14__	288760487381389.00(0.00,Inf)	1.67(0.07,39.16)	0.00(0.00,Inf)						
ASV_2198	Firmicutes;D_2__Clostridia;D_3__Clostridiales;D_4__Ruminococcaceae;D_5__Ruminococcaceae UCG-010;D_6__uncultured organism;D_7__;D_8__;D_9__;D_10__;D_11__;D_12__;D_13__;D_14__	1.19(0.18,8.09)	0.18(0.03,1.25)	0.15(0.02,0.94); p=0.04243	1.25(0.17,9.38)	0.15(0.02,1.03)	0.12(0.02,0.73);p=0.02123			↓
ASV_2218	Firmicutes;D_2__Clostridia;D_3__Clostridiales;D_4__Family XIII;D_5__[Eubacterium] nodatum group;D_6__Eubacterium infirmum F0142;D_7__;D_8__;D_9__;D_10__;D_11__;D_12__;D_13__;D_14__	1.11(0.03,45.04)	0.25(0.01,9.37)	0.23(0.01,6.66)						
ASV_2234	Firmicutes;D_2__Clostridia;D_3__Clostridiales;D_4__Peptococcaceae;D_5__Peptococcus				0.14(0.00,6.64)	0.20(0.00,8.71)	1.42(0.05,38.01)			
ASV_2236	Firmicutes;D_2__Clostridia;D_3__Clostridiales;D_4__Lachnospiraceae;D_5__Lachnospiraceae NC2004 group;D_6__uncultured bacterium;D_7__;D_8__;D_9__;D_10__;D_11__;D_12__;D_13__;D_14__	7.75(0.21,280.35)	346212839725.74(0.00,Inf)	44693565601.07(0.00,Inf)						
ASV_2240	Firmicutes;D_2__Clostridia;D_3__Clostridiales;D_4__Ruminococcaceae;D_5__Ruminiclostridium;D_6__metagenome;D_7__;D_8__;D_9__;D_10__;D_11__;D_12__;D_13__;D_14__	0.40(0.03,4.98)	0.36(0.03,4.60)	0.89(0.10,7.54)	0.39(0.03,5.60)	0.34(0.02,4.67)	0.87(0.10,7.52)			
ASV_2241	Proteobacteria;D_2__Gammaproteobacteria;D_3__Betaproteobacteriales;D_4__Burkholderiaceae;D_5__Lautropia;D_6__uncultured bacterium;D_7__;D_8__;D_9__;D_10__;D_11__;D_12__;D_13__;D_14__	10.60(0.16,714.93)	5.31(0.08,338.95)	0.50(0.01,35.95)						
ASV_2249	Firmicutes;D_2__Clostridia;D_3__Clostridiales;D_4__Lachnospiraceae;D_5__Lachnospiraceae FCS020 group;D_6__uncultured organism;D_7__;D_8__;D_9__;D_10__;D_11__;D_12__;D_13__;D_14__	0.27(0.03,2.86)	0.66(0.06,7.95)	2.43(0.27,21.63)	0.17(0.01,2.14)	0.56(0.04,7.55)	3.35(0.36,31.00)			
ASV_2258	Bacteroidetes;D_2__Bacteroidia;D_3__Bacteroidales;D_4__Porphyromonadaceae;D_5__Porphyromonas;D_6__Porphyromonas bennonis;D_7__;D_8__;D_9__;D_10__;D_11__;D_12__;D_13__;D_14__	0.81(0.08,8.71)	2.39(0.19,30.49)	2.95(0.27,32.12)	0.67(0.06,8.13)	2.34(0.17,31.98)	3.48(0.31,39.09)			

ASV_2265	Firmicutes;D_2__Clostridia;D_3__Clostridiales;D_4__Lachnospiraceae;D_5__[Ruminococcus] torques group;D_6__Ruminococcus sp. AT10;D_7__;D_8__;D_9__;D_10__;D_11__;D_12__;D_13__;D_14__	1.47(0.05,42.64)	0.48(0.02,14.99)	0.33(0.01,8.39)						
ASV_2311	Firmicutes;D_2__Clostridia;D_3__Clostridiales;D_4__Family XI;D_5__Anaerococcus	0.13(0.01,2.15)	1849365162.14 (0.00,Inf)	13787883475.05 (0.00,Inf)						
ASV_2314	Firmicutes;D_2__Clostridia;D_3__Clostridiales;D_4__Lachnospiraceae;D_5__[Ruminococcus] torques group	5.08(0.23,112.50)	7.89(0.29,213.36)	1.55(0.06,40.38)						
ASV_2319	Firmicutes;D_2__Clostridia;D_3__Clostridiales;D_4__Ruminococcaceae;D_5__Faecalibacterium;D_6__uncultured Clostridiaceae bacterium;D_7__;D_8__;D_9__;D_10__;D_11__;D_12__;D_13__;D_14__	3.80(0.08,170.78)	0.08(0.00,2.55)	0.02(0.00,0.76); p=0.03464						
ASV_2320	Firmicutes;D_2__Clostridia;D_3__Clostridiales;D_4__Ruminococcaceae;D_5__Faecalibacterium	1.18(0.04,35.40)	0.44(0.01,13.89)	0.37(0.01,9.58)						
ASV_2333	Firmicutes;D_2__Clostridia;D_3__Clostridiales;D_4__Defluviitaleaceae;D_5__Defluviitaleaceae UCG-011;D_6__uncultured bacterium;D_7__;D_8__;D_9__;D_10__;D_11__;D_12__;D_13__;D_14__	3.65(0.09,141.20)	1.25(0.03,49.64)	0.34(0.01,11.88)						
ASV_2342	Firmicutes;D_2__Clostridia;D_3__Clostridiales;D_4__Family XIII;D_5__Family XIII AD3011 group	0.42(0.01,16.11)	0.06(0.00,2.40)	0.15(0.01,3.50)						
ASV_2360	Firmicutes;D_2__Clostridia;D_3__Clostridiales;D_4__Ruminococcaceae;D_5__Oscillibacter	2.64(0.14,49.18)	692785519939.78(0.00,Inf)	262892425040.99(0.00,Inf)						
ASV_2386	Firmicutes;D_2__Clostridia;D_3__Clostridiales;D_4__Clostridiales vadinBB60 group;D_5__uncultured bacterium;D_6__;D_7__;D_8__;D_9__;D_10__;D_11__;D_12__;D_13__;D_14__				2.92(0.11,78.94)	0.96(0.04,23.35)	0.33(0.02,6.70)			
ASV_2483	Firmicutes;D_2__Clostridia;D_3__Clostridiales;D_4__Peptococcaceae;D_5__uncultured;D_6__metagenome;D_7__;D_8__;D_9__;D_10__;D_11__;D_12__;D_13__;D_14__	0.00(0.00,Inf)	0.00(0.00,Inf)	0.32(0.05,1.94)	0.00(0.00,Inf)	0.00(0.00,Inf)	0.20(0.03,1.27)			
ASV_2484	Firmicutes;D_2__Clostridia;D_3__Clostridiales;D_4__Ruminococcaceae;D_5__CAG-352;D_6__uncultured bacterium;D_7__;D_8__;D_9__;D_10__;D_11__;D_12__;D_13__;D_14__	0.53(0.03,9.22)	0.38(0.02,7.08)	0.72(0.05,10.26)						
ASV_2485	Proteobacteria;D_2__Gammaproteobacteria;D_3__Betaproteobacteriales;D_4__Burkholderiaceae;D_5__Noviherbaspirillum	1.81(0.07,44.30)	0.38(0.02,8.10)	0.21(0.01,4.09)						
ASV_2490	Firmicutes;D_2__Clostridia;D_3__Clostridiales	1.36(0.04,41.57)	1.31(0.04,43.89)	0.96(0.03,27.10)	2.25(0.04,119.13)	0.90(0.02,39.15)	0.40(0.01,13.14)			
ASV_2491	Firmicutes;D_2__Clostridia;D_3__Clostridiales;D_4__Lachnospiraceae	10.42(0.24,461.06)	1.43(0.03,59.92)	0.14(0.00,5.46)	4.73(0.09,257.65)	0.79(0.02,36.38)	0.17(0.00,6.98)			
ASV_2518	Firmicutes;D_2__Clostridia;D_3__Clostridiales;D_4__Lachnospiraceae	0.44(0.02,8.58)	1.02(0.04,24.30)	2.30(0.13,39.50)						
ASV_2520	Firmicutes;D_2__Clostridia;D_3__Clostridiales;D_4__Lachnospiraceae;D_5__Roseburia	0.00(0.00,Inf)	0.00(0.00,Inf)	0.17(0.01,3.64)						
ASV_2574	Actinobacteria;D_2__Actinobacteria;D_3__Propionibacteriales;D_4__Propionibacteriaceae;D_5__Propionibacterium;D_6__Propionibacterium freudenreichii;D_7__;D_8__;D_9__;D_10__;D_11__;D_12__;D_13__;D_14__	0.00(0.00,Inf)	0.00(0.00,Inf)	0.28(0.02,5.03)						
ASV_2579	Firmicutes;D_2__Clostridia;D_3__Clostridiales;D_4__Ruminococcaceae;D_5__Subdoligranulum;D_6__uncultured bacterium;D_7__;D_8__;D_9__;D_10__;D_11__;D_12__;D_13__;D_14__	5.06(0.31,82.21)	1.63(0.12,22.58)	0.32(0.02,5.11)	47.50(1.18,1906.90);p=0.04044	2.25(0.15,34.11)	0.05(0.00,1.39)	↑		
ASV_2582	Firmicutes;D_2__Clostridia;D_3__Clostridiales;D_4__Lachnospiraceae;D_5__Blautia	1.17(0.03,43.80)	0.07(0.00,1.57)	0.06(0.00,1.08)	1.28(0.03,55.40)	0.07(0.00,1.61)	0.05(0.00,1.15)			
ASV_2593	Firmicutes;D_2__Clostridia;D_3__Clostridiales;D_4__Family XIII;D_5__Family XIII AD3011 group;D_6__uncultured Eubacterium sp.;D_7__;D_8__;D_9__;D_10__;D_11__;D_12__;D_13__;D_14__	2.06(0.11,37.61)	0.30(0.02,5.38)	0.14(0.01,2.29)						

ASV_2594	Firmicutes;D_2__Clostridia;D_3__Clostridiales;D_4__Family XIII;D_5__Family XIII AD3011 group	0.41(0.01,23.67)	0.56(0.01,37.22)	1.37(0.03,65.35)						
ASV_2599	Firmicutes;D_2__Clostridia;D_3__Clostridiales;D_4__Ruminococcaceae;D_5__Subdoligranulum;D_6__uncultured bacterium;D_7__;D_8__;D_9__;D_10__;D_11__;D_12__;D_13__;D_14__	0.00(0.00,Inf)	0.00(0.00,Inf)	9.50(1.07,84.05);p=0.04296						
ASV_2603	Firmicutes;D_2__Clostridia;D_3__Clostridiales;D_4__Ruminococcaceae;D_5__DTU089	4.75(0.19,118.54)	2.69(0.11,67.68)	0.57(0.02,13.94)	4.54(0.14,144.84)	1.23(0.05,31.09)	0.27(0.01,7.38)			
ASV_2611	Firmicutes;D_2__Clostridia;D_3__Clostridiales;D_4__Lachnospiraceae;D_5__[Ruminococcus] gauvreauii group;D_6__Ruminococcus gauvreauii;D_7__;D_8__;D_9__;D_10__;D_11__;D_12__;D_13__;D_14__	1.88(0.16,21.65)	7.50(0.56,100.13)	3.98(0.34,46.46)						
ASV_2616	Firmicutes;D_2__Clostridia;D_3__Clostridiales;D_4__Peptostreptococcaceae;D_5__uncultured;D_6__uncultured bacterium;D_7__;D_8__;D_9__;D_10__;D_11__;D_12__;D_13__;D_14__	1.30(0.03,56.85)	0.43(0.01,15.41)	0.33(0.01,10.03)						
ASV_2636	Firmicutes;D_2__Clostridia;D_3__Clostridiales;D_4__Ruminococcaceae	0.00(0.00,Inf)	0.00(0.00,Inf)	0.23(0.01,4.32)						
ASV_2640	Proteobacteria;D_2__Deltaproteobacteria;D_3__Desulfovibrionales;D_4__Desulfovibrionaceae;D_5__Bilophila	3.30(0.19,58.48)	4.29(0.20,93.63)	1.30(0.05,30.90)						
ASV_2641	Firmicutes;D_2__Clostridia;D_3__Clostridiales;D_4__Lachnospiraceae;D_5__[Eubacterium] fissicatena group;D_6__uncultured bacterium;D_7__;D_8__;D_9__;D_10__;D_11__;D_12__;D_13__;D_14__	0.38(0.02,8.46)	0.24(0.01,5.75)	0.65(0.04,10.51)	0.16(0.00,5.13)	0.05(0.00,1.36)	0.30(0.02,4.94)			
ASV_2652	Proteobacteria;D_2__Gammaproteobacteria;D_3__Betaproteobacteriales;D_4__Burkholderiaceae;D_5__Parasutterella;D_6__uncultured bacterium;D_7__;D_8__;D_9__;D_10__;D_11__;D_12__;D_13__;D_14__	9.55(0.52,175.86)	16.00(0.53,479.90)	1.68(0.04,70.04)	14.15(0.59,339.82)	28.97(0.63,1324.80)	2.05(0.04,117.12)			
ASV_2662	Firmicutes;D_2__Clostridia;D_3__Clostridiales;D_4__Ruminococcaceae;D_5__Faecalibacterium	62623812346783.10(0.00,Inf)	3.33(0.11,102.71)	0.00(0.00,Inf)						
ASV_2664	Firmicutes;D_2__Clostridia;D_3__Clostridiales;D_4__Lachnospiraceae;D_5__Blautia;D_6__Blautia sp. Marseille-P3087;D_7__;D_8__;D_9__;D_10__;D_11__;D_12__;D_13__;D_14__	1.07(0.04,30.61)	0.81(0.03,22.97)	0.76(0.03,17.20)						
ASV_2665	Patescibacteria;D_2__Saccharimonadia;D_3__Saccharimonadales;D_4__TM7 phylum sp. oral clone FR058;D_5__;D_6__;D_7__;D_8__;D_9__;D_10__;D_11__;D_12__;D_13__;D_14__	2.08(0.25,17.65)	2.75(0.30,25.44)	1.32(0.16,11.05)						
ASV_2667	Firmicutes;D_2__Clostridia;D_3__Clostridiales;D_4__Family XIII;D_5__[Eubacterium] brachy group;D_6__uncultured bacterium;D_7__;D_8__;D_9__;D_10__;D_11__;D_12__;D_13__;D_14__	0.00(0.00,Inf)	0.00(0.00,Inf)	0.76(0.04,13.22)						
ASV_2683	Firmicutes;D_2__Clostridia;D_3__Clostridiales;D_4__Ruminococcaceae;D_5__[Eubacterium] coprostanoligenes group;D_6__unidentified;D_7__;D_8__;D_9__;D_10__;D_11__;D_12__;D_13__;D_14__	28972209152.72(0.00,Inf)	4.07(0.10,169.02)	0.00(0.00,Inf)						
ASV_2684	Firmicutes;D_2__Clostridia;D_3__Clostridiales;D_4__Lachnospiraceae	2.80(0.20,40.05)	4.10(0.24,69.15)	1.46(0.09,23.50)	1.68(0.10,27.84)	3.29(0.18,60.06)	1.96(0.12,32.27)			
ASV_2685	Firmicutes;D_2__Clostridia;D_3__Clostridiales;D_4__Lachnospiraceae	0.84(0.08,8.37)	4.91(0.27,90.18)	5.83(0.37,92.51)	0.73(0.06,8.31)	6.35(0.28,143.99)	8.71(0.44,174.50)			
ASV_2687	Actinobacteria;D_2__Actinobacteria;D_3__Actinomycetales;D_4__Actinomycetaceae;D_5__Actinomyces	0.44(0.03,5.76)	1.00(0.06,16.33)	2.30(0.20,26.97)						
ASV_2732	Firmicutes;D_2__Clostridia;D_3__Clostridiales;D_4__Lachnospiraceae;D_5__Blautia	0.61(0.02,21.71)	212942161606839.00(0.00,Inf)	350791134765962.00(0.00,Inf)						
ASV_2760	Proteobacteria;D_2__Gammaproteobacteria;D_3__Pseudomonadales;D_4__Moraxellaceae;D_5__Acinetobacter				1606216137480483.00(0.00,Inf)	2.11(0.12,37.48)	0.00(0.00,Inf)			
ASV_2778	Firmicutes;D_2__Clostridia;D_3__Clostridiales;D_4__Lachnospiraceae;D_5__GCA-900066575;D_6__uncultured bacterium;D_7__;D_8__;D_9__;D_10__;D_11__;D_12__;D_13__;D_14__	4.94(0.47,51.77)	4.28(0.38,47.98)	0.87(0.09,8.81)	10.52(0.91,122.01)	8.85(0.77,101.17)	0.84(0.08,8.71)			

ASV_2847	Actinobacteria;D_2__Actinobacteria;D_3__Corynebacteriales;D_4__Corynebacteriaceae;D_5__Corynebacterium 1	0.00(0.00,Inf)	0.00(0.00,Inf)	0.25(0.02,3.26)	0.00(0.00,Inf)	0.00(0.00,Inf)	0.37(0.03,5.10)			
ASV_2889	Firmicutes;D_2__Clostridia;D_3__Clostridiales;D_4__Ruminococcaceae;D_5__Ruminococcaceae UCG-003;D_6__uncultured bacterium;D_7__;D_8__;D_9__;D_10__;D_11__;D_12__;D_13__;D_14__	0.18(0.00,6.32)	83565148315.99(0.00,Inf)	473434434438.53(0.00,Inf)						
ASV_2902	Firmicutes;D_2__Clostridia;D_3__Clostridiales;D_4__Lachnospiraceae	0.19(0.01,2.71)	1.73(0.09,34.61)	9.32(0.63,137.90)						
ASV_2903	Firmicutes;D_2__Clostridia;D_3__Clostridiales;D_4__Ruminococcaceae	0.49(0.03,7.31)	2.81(0.13,60.45)	5.75(0.34,98.58)	0.44(0.03,6.69)	4.61(0.16,136.53)	10.51(0.57,194.28)			
ASV_2904	Firmicutes;D_2__Clostridia;D_3__Clostridiales;D_4__Lachnospiraceae;D_5__Fusicatenibacter;D_6__uncultured bacterium;D_7__;D_8__;D_9__;D_10__;D_11__;D_12__;D_13__;D_14__	0.00(0.00,Inf)	0.00(0.00,Inf)	0.30(0.03,3.11)						
ASV_2920	Firmicutes;D_2__Clostridia;D_3__Clostridiales;D_4__Lachnospiraceae;D_5__Oribacterium;D_6__Oribacterium parvum ACB1;D_7__;D_8__;D_9__;D_10__;D_11__;D_12__;D_13__;D_14__	0.00(0.00,Inf)	0.00(0.00,Inf)	1.27(0.04,37.59)	0.00(0.00,Inf)	0.00(0.00,Inf)	1.20(0.04,35.36)			
ASV_2921	Actinobacteria;D_2__Coriobacteriia;D_3__Coriobacteriales;D_4__Eggerthellaceae;D_5__Slackia;D_6__Slackia sp. S4-C6;D_7__;D_8__;D_9__;D_10__;D_11__;D_12__;D_13__;D_14__	0.88(0.04,19.09)	0.36(0.02,7.35)	0.40(0.03,6.46)	0.84(0.03,23.82)	0.23(0.01,5.31)	0.27(0.02,4.65)			
ASV_2931	Firmicutes;D_2__Clostridia;D_3__Clostridiales;D_4__Christensenellaceae	1.56(0.16,14.88)	0.32(0.04,2.84)	0.21(0.03,1.67)	1.93(0.17,22.18)	0.27(0.03,2.52)	0.14(0.02,1.16)			
ASV_2943	Firmicutes;D_2__Clostridia;D_3__Clostridiales;D_4__Lachnospiraceae;D_5__Coprococcus 3	1.50(0.03,76.77)	0.34(0.01,12.84)	0.22(0.01,7.66)						
ASV_2955	Firmicutes;D_2__Clostridia;D_3__Clostridiales;D_4__Family XIII;D_5__uncultured;D_6__uncultured bacterium;D_7__;D_8__;D_9__;D_10__;D_11__;D_12__;D_13__;D_14__	0.00(0.00,Inf)	0.00(0.00,Inf)	0.26(0.01,6.84)						
ASV_2974	Firmicutes;D_2__Clostridia;D_3__Clostridiales;D_4__Lachnospiraceae;D_5__Blautia	1.76(0.02,156.20)	0.14(0.00,8.67)	0.08(0.00,4.47)						
ASV_2984	Firmicutes;D_2__Clostridia;D_3__Clostridiales;D_4__Ruminococcaceae;D_5__Ruminiclostridium 5;D_6__uncultured bacterium;D_7__;D_8__;D_9__;D_10__;D_11__;D_12__;D_13__;D_14__	3.33(0.10,106.93)	433676826541.15(0.00,Inf)	130360171801.91(0.00,Inf)						
ASV_2998	Firmicutes;D_2__Clostridia;D_3__Clostridiales;D_4__Lachnospiraceae;D_5__CHKCI001;D_6__uncultured organism;D_7__;D_8__;D_9__;D_10__;D_11__;D_12__;D_13__;D_14__	2.03(0.03,131.86)	2.11(0.03,158.05)	1.04(0.02,65.72)						
ASV_3031	Firmicutes;D_2__Clostridia;D_3__Clostridiales;D_4__Family XIII;D_5__Family XIII AD3011 group;D_6__uncultured bacterium;D_7__;D_8__;D_9__;D_10__;D_11__;D_12__;D_13__;D_14__	0.00(0.00,Inf)	0.00(0.00,Inf)	4.25(0.21,87.19)						
ASV_3033	Firmicutes;D_2__Clostridia;D_3__Clostridiales;D_4__Lachnospiraceae;D_5__Blautia	0.00(0.00,Inf)	0.00(0.00,Inf)	1.68(0.05,53.36)						
ASV_3048	Firmicutes;D_2__Clostridia;D_3__Clostridiales;D_4__Lachnospiraceae	2.95(0.06,150.54)	0.89(0.02,37.00)	0.30(0.01,13.39)	1.01(0.01,73.05)	0.38(0.01,21.33)	0.37(0.01,17.07)			
ASV_3052	Firmicutes;D_2__Clostridia;D_3__Clostridiales;D_4__Lachnospiraceae;D_5__Agathobacter	0.75(0.02,27.85)	0.62(0.02,24.65)	0.82(0.03,24.51)						
ASV_3073	Proteobacteria;D_2__Gammaproteobacteria;D_3__Pasteurellales;D_4__Pasteurellaceae;D_5__Aggregatibacter				3.41(0.00,Inf)	0.00(0.00,Inf)	0.00(0.00,Inf)			
ASV_3084	Firmicutes;D_2__Clostridia;D_3__Clostridiales;D_4__Ruminococcaceae	7.52(0.35,162.98)	0.94(0.05,18.69)	0.12(0.01,2.47)	10.63(0.43,260.35)	2.34(0.11,48.49)	0.22(0.01,4.46)			
ASV_3086	Firmicutes;D_2__Clostridia;D_3__Clostridiales;D_4__Lachnospiraceae;D_5__[Eubacterium] hallii group	0.00(0.00,Inf)	0.00(0.00,Inf)	0.06(0.00,2.33)						

ASV_3095	Firmicutes;D_2__Clostridia;D_3__Clostridiales;D_4__Lachnospiraceae;D_5__Anaerostipes	0.94(0.10,9.05)	2.70(0.17,43.60)	2.88(0.21,39.91)							
ASV_3137	Firmicutes;D_2__Clostridia;D_3__Clostridiales;D_4__Lachnospiraceae;D_5__Lachnoclostridium				0.34(0.01,18.41)	0.21(0.00,10.46)	0.61(0.02,20.62)				
ASV_3165	Firmicutes;D_2__Clostridia;D_3__Clostridiales;D_4__Ruminococcaceae;D_5__Ruminiclostridium 5				0.53(0.01,37.48)	0.17(0.00,11.10)	0.31(0.01,14.42)				
ASV_3168	Firmicutes;D_2__Bacilli;D_3__Lactobacillales;D_4__Streptococcaceae;D_5__Streptococcus	1.04(0.10,10.43)	3.15(0.21,46.30)	3.03(0.24,39.07)							
ASV_3171	Firmicutes;D_2__Clostridia;D_3__Clostridiales;D_4__Lachnospiraceae;D_5__uncultured	0.86(0.03,21.89)	2.22(0.06,76.25)	2.59(0.09,71.00)							
ASV_3180	Firmicutes;D_2__Clostridia;D_3__Clostridiales;D_4__Lachnospiraceae	1.75(0.06,55.24)	0.49(0.02,13.59)	0.28(0.01,7.12)							
ASV_3189	Firmicutes;D_2__Clostridia;D_3__Clostridiales;D_4__Lachnospiraceae	0.00(0.00,Inf)	0.00(0.00,Inf)	0.32(0.03,3.77)							
ASV_3250	Firmicutes;D_2__Clostridia;D_3__Clostridiales;D_4__Ruminococcaceae;D_5__Hydrogenoanaerobacterium;D_6__uncultured bacterium;D_7__;D_8__;D_9__;D_10__;D_11__;D_12__;D_13__;D_14__	0.88(0.02,33.42)	0.49(0.01,19.48)	0.56(0.02,16.94)							
ASV_3272	Firmicutes;D_2__Clostridia;D_3__Clostridiales;D_4__Ruminococcaceae;D_5__Subdoligranulum;D_6__uncultured bacterium;D_7__;D_8__;D_9__;D_10__;D_11__;D_12__;D_13__;D_14__	195869638128.08(0.00,Inf)	0.23(0.01,5.54)	0.00(0.00,Inf)							
ASV_3323	Firmicutes;D_2__Clostridia;D_3__Clostridiales;D_4__Ruminococcaceae;D_5__uncultured;D_6__uncultured bacterium;D_7__;D_8__;D_9__;D_10__;D_11__;D_12__;D_13__;D_14__	0.96(0.04,21.10)	3.78(0.13,114.33)	3.94(0.16,98.70)	1.35(0.06,31.97)	2.09(0.09,50.91)	1.55(0.06,38.92)				
ASV_3342	Firmicutes;D_2__Clostridia;D_3__Clostridiales;D_4__Lachnospiraceae	0.00(0.00,Inf)	0.00(0.00,Inf)	0.38(0.02,7.00)							
ASV_3368	Actinobacteria;D_2__Coriobacteriia;D_3__Coriobacteriales;D_4__Coriobacteriaceae;D_5__Collinsella;D_6__uncultured bacterium;D_7__;D_8__;D_9__;D_10__;D_11__;D_12__;D_13__;D_14__	1.89(0.11,33.41)	0.51(0.04,7.10)	0.27(0.02,3.83)	1.14(0.04,32.99)	0.17(0.01,3.10)	0.15(0.01,2.90)				
ASV_3395	Firmicutes;D_2__Clostridia;D_3__Clostridiales;D_4__Lachnospiraceae;D_5__Lachnospiraceae NC2004 group;D_6__uncultured bacterium;D_7__;D_8__;D_9__;D_10__;D_11__;D_12__;D_13__;D_14__	956856147529602.00(0.00,Inf)	969457566615515.00(0.00,Inf)	1.01(0.00,Inf)							
ASV_3680	Firmicutes;D_2__Clostridia;D_3__Clostridiales;D_4__Lachnospiraceae;D_5__Lachnospiraceae UCG-004	0.49(0.03,8.63)	10.61(0.30,375.36)	21.57(0.73,636.37)							
ASV_3748	Firmicutes;D_2__Clostridia;D_3__Clostridiales;D_4__Ruminococcaceae;D_5__Subdoligranulum;D_6__uncultured bacterium;D_7__;D_8__;D_9__;D_10__;D_11__;D_12__;D_13__;D_14__	0.49(0.05,4.66)	2.93(0.16,52.67)	5.93(0.43,82.77)	0.71(0.07,6.87)	3.63(0.19,69.07)	5.15(0.39,67.92)				
ASV_3939	Firmicutes;D_2__Clostridia;D_3__Clostridiales;D_4__Ruminococcaceae;D_5__Faecalibacterium	0.00(0.00,Inf)	0.00(0.00,Inf)	0.04(0.00,1.93)							
ASV_3940	Firmicutes;D_2__Bacilli;D_3__Lactobacillales;D_4__Streptococcaceae;D_5__Streptococcus	0.00(0.00,Inf)	0.00(0.00,Inf)	2.61(0.08,81.28)							
ASV_4462	Firmicutes;D_2__Clostridia;D_3__Clostridiales;D_4__Lachnospiraceae;D_5__[Eubacterium] hallii group	1.09(0.00,Inf)	0.00(0.00,Inf)	0.00(0.00,Inf)							

Table 3.2: Species-level differences in the gut microbial communities: participants with MS (DMD naïve and exposed), and monophasic demyelinating disease compared to controls for individual ASVs

		Rate ratios (95%CIs) unadjusted					Rate ratios (95%CIs) age and sex adjusted									
		Ref.: MS cases DMD-		Ref.: MS cases DMD+	Ref.: Controls		Ref.: MS cases DMD-		Ref.: MS cases DMD+	Ref.: Controls						
Taxa Identifier (for internal use)	Species-level findings (shown as: Phylum;Class;Order;Family;Genus;Species) i.e., D_6= species	MS cases DMD+	ADS	ADS	MS cases DMD-	MS cases DMD+	MS cases DMD+	ADS	ADS	MS cases DMD-	MS cases DMD+	MSDMD+ (vs MSDMD-)	ADS (vs MSDMD-)	ADS (vs MSDMD+)	MSDMD- (vs control)	MSDMD+ (vs control)
ASV_3	Firmicutes;D_2__Bacilli;D_3__Lactobacillales;D_4__Streptococcaceae;D_5__Streptococcus;D_6__Streptococcus salivarius subsp. thermophilus;D_7__;D_8__;D_9__;D_10__;D_11__;D_12__;D_13__;D_14__	1.20(0.42, 3.43)	0.67(0.25, 1.78)	0.56(0.28, 1.11)	1.13(0.42, 3.07)	1.36(0.67, 2.78)	1.22(0.43, 3.48)	0.64(0.23, 1.76)	0.52(0.26,1.07)	1.18(0.43, 3.22)	1.43(0.70,2.94)					
ASV_4	Firmicutes;D_2__Clostridia;D_3__Clostridiales;D_4__Lachnospiraceae;D_5__Blautia	1.15(0.46, 2.91)	1.06(0.45, 2.52)	0.92(0.50, 1.69)	0.89(0.37, 2.13)	1.03(0.55, 1.92)	1.21(0.48, 3.03)	1.17(0.48, 2.86)	0.97(0.52,1.83)	0.83(0.34, 2.01)	1.00(0.53,1.88)					
ASV_5	Firmicutes;D_2__Clostridia;D_3__Clostridiales;D_4__Lachnospiraceae;D_5__Blautia	1.30(0.56, 3.00)	1.49(0.68, 3.25)	1.14(0.66, 1.99)	0.72(0.33, 1.59)	0.94(0.53, 1.65)	1.32(0.57, 3.03)	1.44(0.64, 3.21)	1.09(0.62,1.93)	0.77(0.35, 1.72)	1.02(0.58,1.80)					
ASV_7	Firmicutes;D_2__Clostridia;D_3__Clostridiales;D_4__Lachnospiraceae;D_5__Blautia	0.85(0.42, 1.70)	0.74(0.39, 1.43)	0.88(0.55, 1.40)	1.13(0.58, 2.20)	0.96(0.60, 1.54)	0.81(0.40, 1.62)	0.68(0.35, 1.33)	0.84(0.52,1.35)	1.20(0.61, 2.34)	0.97(0.60,1.56)					
ASV_8	Firmicutes;D_2__Clostridia;D_3__Clostridiales;D_4__Lachnospiraceae;D_5__Coprococcus 3	1.08(0.26, 4.54)	1.01(0.26, 3.90)	0.94(0.36, 2.44)	0.89(0.23, 3.49)	0.96(0.36, 2.55)	1.09(0.26, 4.53)	1.07(0.27, 4.21)	0.97(0.37,2.57)	0.95(0.24, 3.71)	1.04(0.39,2.74)					
ASV_9	Firmicutes;D_2__Clostridia;D_3__Clostridiales;D_4__Lachnospiraceae;D_5__Anaerostipes	2.61(1.07, 6.35);p=0.03449	2.49(1.08, 5.73);p=0.03155	0.96(0.53, 1.72)	0.44(0.19, 1.03)	1.16(0.63, 2.12)	2.49(1.03, 6.06);p=0.0436	2.20(0.93, 5.18)	0.88(0.48,1.61)	0.49(0.21, 1.15)	1.23(0.67,2.25)	↑				
ASV_10	Bacteroidetes;D_2__Bacteroidia;D_3__Bacteroidales;D_4__Bacteroidaceae;D_5__Bacteroides	0.89(0.18, 4.45)	1.66(0.37, 7.48)	1.86(0.64, 5.41)	1.79(0.39, 8.21)	1.59(0.53, 4.74)	0.97(0.20, 4.70)	2.21(0.48, 10.19)	2.29(0.78,6.74)	1.48(0.32, 6.75)	1.43(0.48,4.22)					
ASV_11	Firmicutes;D_2__Clostridia;D_3__Clostridiales;D_4__Lachnospiraceae;D_5__Dorea	1.06(0.35, 3.21)	0.75(0.26, 2.11)	0.70(0.34, 1.47)	1.39(0.49, 3.98)	1.48(0.70, 3.13)	1.00(0.33, 3.03)	0.78(0.27, 2.26)	0.78(0.37,1.65)	1.43(0.49, 4.11)	1.43(0.67,3.05)					
ASV_13	Firmicutes;D_2__Bacilli;D_3__Lactobacillales;D_4__Streptococcaceae;D_5__Streptococcus	1.79(0.49, 6.50)	0.60(0.18, 2.01)	0.33(0.14, 0.79);p=0.01208	0.52(0.15, 1.78)	0.94(0.39, 2.25)	2.22(0.61, 8.04)	0.62(0.18, 2.16)	0.28(0.12,0.67);p=0.00454	0.53(0.15, 1.82)	1.17(0.49,2.82)			↓		
ASV_14	Firmicutes;D_2__Clostridia;D_3__Clostridiales;D_4__Lachnospiraceae;D_5__Blautia	1.32(0.50, 3.53)	1.54(0.61, 3.85)	1.16(0.61, 2.23)	1.04(0.41, 2.64)	1.38(0.71, 2.69)	1.27(0.48, 3.37)	1.60(0.62, 4.10)	1.26(0.65,2.46)	1.05(0.41, 2.69)	1.33(0.68,2.60)					
ASV_15	Firmicutes;D_2__Clostridia;D_3__Clostridiales;D_4__Lachnospiraceae;D_5__[Eubacterium] hallii group	1.61(0.48, 5.37)	0.87(0.28, 2.68)	0.54(0.24, 1.20)	1.60(0.51, 5.03)	2.58(1.14, 5.86);p=0.02307	1.49(0.45, 4.96)	0.72(0.23, 2.31)	0.48(0.21,1.10)	1.84(0.58, 5.86)	2.74(1.20,6.25);p=0.01667					↑
ASV_18	Firmicutes;D_2__Clostridia;D_3__Clostridiales;D_4__Christensenellaceae;D_5__Christensenellaceae R-7 group;D_6__uncultured organism;D_7__;D_8__;D_9__;D_10__;D_11__;D_12__;D_13__;D_14__	3.31(0.08, 138.52)	1.83(0.06, 60.34)	0.55(0.05, 6.52)	0.14(0.00, 4.88)	0.47(0.04, 5.88)										
ASV_24	Bacteroidetes;D_2__Bacteroidia;D_3__Bacteroidales;D_4__Bacteroidaceae;D_5__Bacteroides	0.62(0.07, 5.88)	1.48(0.18, 12.21)	2.40(0.54, 10.65)	0.95(0.11, 8.06)	0.59(0.13, 2.72)	0.83(0.09, 7.60)	1.90(0.22, 16.13)	2.29(0.50,10.40)	0.65(0.08, 5.42)	0.53(0.12,2.44)					

ASV_25	Firmicutes;D_2__Clostridia;D_3__Clostridiales;D_4__Lachnospiraceae;D_5__Blautia	0.92(0.10, 8.61)	1.34(0.17, 10.83)	1.45(0.33, 6.37)	0.70(0.08, 5.78)	0.64(0.14, 2.93)	1.14(0.13, 9.93)	1.80(0.22, 14.54)	1.58(0.36,6.92)	0.31(0.04, 2.51)	0.36(0.08,1.57)					
ASV_26	Firmicutes;D_2__Clostridia;D_3__Clostridiales;D_4__Ruminococcaceae;D_5__Ruminiclostridium 5	4.34(0.87, 21.55)	3.48(0.78, 15.61)	0.80(0.28, 2.32)	0.22(0.05, 1.00);p=0.04995	0.95(0.32, 2.82)	4.19(0.85, 20.68)	3.21(0.69, 14.98)	0.76(0.26,2.27)	0.26(0.06, 1.22)	1.10(0.37,3.28)					
ASV_27	Actinobacteria;D_2__Coriobacteriia;D_3__Coriobacteriales;D_4__Coriobacteriaceae;D_5__Collinsella	0.60(0.11, 3.33)	0.98(0.20, 4.90)	1.64(0.53, 5.10)	0.75(0.15, 3.81)	0.45(0.14, 1.44)										
ASV_28	Firmicutes;D_2__Clostridia;D_3__Clostridiales;D_4__Lachnospiraceae;D_5__uncultured;D_6__uncultured Eubacterium sp.;D_7__;D_8__;D_9__;D_10__;D_11__;D_12__;D_13__;D_14__	0.32(0.08, 1.39)	1.37(0.35, 5.33)	4.21(1.60, 11.07);p=0.00355	1.35(0.34, 5.34)	0.44(0.16, 1.18)	0.35(0.09, 1.43)	1.96(0.51, 7.61)	5.61(2.14,14.76);p=0.00047	0.82(0.21, 3.14)	0.29(0.11,0.75);p=0.01125			↑		↓
ASV_31	Firmicutes;D_2__Clostridia;D_3__Clostridiales;D_4__Ruminococcaceae;D_5__[Eubacterium] coprostanoligenes group;D_6__uncultured organism;D_7__;D_8__;D_9__;D_10__;D_11__;D_12__;D_13__;D_14__	3.90(0.06, 259.65)	3.35(0.07, 170.85)	0.86(0.05, 13.88)	0.27(0.00, 14.18)	1.03(0.06, 17.86)										
ASV_32	Firmicutes;D_2__Clostridia;D_3__Clostridiales;D_4__Christensenellaceae;D_5__Christensenellaceae R-7 group	0.29(0.01, 7.38)	3.41(0.17, 69.16)	11.60(1.37,98.52); p=0.02476	0.57(0.03, 12.00)	0.17(0.02, 1.50)	0.18(0.01, 4.48)	3.45(0.16, 74.66)	18.84(2.07, 171.73);p=0.00923	0.30(0.01, 6.26)	0.05(0.01,0.49);p=0.00966			↑		↓
ASV_33	Bacteroidetes;D_2__Bacteroidia;D_3__Bacteroidales;D_4__Rikenellaceae;D_5__Alistipes;D_6__Alistipes indistinctus YIT 12060;D_7__;D_8__;D_9__;D_10__;D_11__;D_12__;D_13__;D_14__	9423897861.19(0.00,Inf)	7840350785.19(0.00,Inf)	0.83(0.11, 6.49)	0.00(0.00, Inf)	1.85(0.23, 15.20)										
ASV_34	Firmicutes;D_2__Negativicutes;D_3__Selenomonadales;D_4__Veillonellaceae;D_5__Dialister	0.35(0.04, 3.23)	0.34(0.04, 2.68)	0.96(0.22, 4.15)	2.92(0.36, 23.84)	1.03(0.23, 4.63)	0.38(0.04, 3.51)	0.37(0.04, 3.16)	0.97(0.21,4.44)	2.60(0.31, 21.90)	0.99(0.22,4.52)					
ASV_35	Firmicutes;D_2__Clostridia;D_3__Clostridiales;D_4__Christensenellaceae;D_5__Christensenellaceae R-7 group	19164821048.69(0.00,Inf)	29438810305.22(0.00,Inf)	1.54(0.11, 22.10)	0.00(0.00, Inf)	0.57(0.04, 8.73)										
ASV_36	Bacteroidetes;D_2__Bacteroidia;D_3__Bacteroidales;D_4__Bacteroidaceae;D_5__Bacteroides;D_6__Bacteroides thetaiotaomicron;D_7__;D_8__;D_9__;D_10__;D_11__;D_12__;D_13__;D_14__	1.10(0.24, 5.09)	1.08(0.26, 4.55)	0.98(0.36, 2.72)	0.65(0.15, 2.77)	0.71(0.25, 2.02)	1.04(0.22, 4.85)	1.13(0.26, 5.00)	1.09(0.38,3.10)	0.65(0.15, 2.82)	0.67(0.24,1.93)					
ASV_37	Firmicutes;D_2__Clostridia;D_3__Clostridiales;D_4__Lachnospiraceae;D_5__[Ruminococcus] torques group;D_6__uncultured organism;D_7__;D_8__;D_9__;D_10__;D_11__;D_12__;D_13__;D_14__	0.17(0.04, 0.80);p=0.025	0.29(0.07, 1.22)	1.68(0.60, 4.69)	5.71(1.32, 24.73);p=0.0198	0.97(0.34, 2.79)	0.21(0.05, 0.97);p=0.04586	0.25(0.06, 1.08)	1.18(0.41,3.34)	4.39(1.01, 19.05);p=0.04812	0.93(0.32,2.64)	↓			↑	
ASV_38	Firmicutes;D_2__Clostridia;D_3__Clostridiales;D_4__Ruminococcaceae;D_5__Ruminococcus 1;D_6__uncultured organism;D_7__;D_8__;D_9__;D_10__;D_11__;D_12__;D_13__;D_14__	0.41(0.07, 2.53)	0.94(0.17, 5.13)	2.28(0.68, 7.58)	0.99(0.18, 5.55)	0.41(0.12, 1.40)	0.39(0.06, 2.35)	0.70(0.12, 4.01)	1.82(0.53,6.22)	1.23(0.22, 6.97)	0.48(0.14,1.64)					
ASV_39	Bacteroidetes;D_2__Bacteroidia;D_3__Bacteroidales;D_4__Rikenellaceae;D_5__Alistipes;D_6__uncultured organism;D_7__;D_8__;D_9__;D_10__;D_11__;D_12__;D_13__;D_14__	0.81(0.11, 6.07)	3.78(0.57, 24.89)	4.66(1.23, 17.70);p=0.02371	0.51(0.07, 3.41)	0.41(0.10, 1.61)	0.72(0.10, 5.44)	3.34(0.48, 23.41)	4.62(1.17,18.29);p=0.02945	0.56(0.08, 3.88)	0.41(0.10,1.61)			↑		
ASV_40	Firmicutes;D_2__Clostridia;D_3__Clostridiales;D_4__Lachnospiraceae;D_5__[Eubacterium] hallii group	2.03(0.22, 18.35)	0.86(0.11, 6.79)	0.43(0.10, 1.83)	0.66(0.08, 5.31)	1.33(0.30, 5.95)	2.03(0.23, 18.31)	1.14(0.14, 9.48)	0.56(0.12,2.50)	0.50(0.06, 4.09)	1.01(0.22,4.54)					
ASV_41	Firmicutes;D_2__Clostridia;D_3__Clostridiales;D_4__Lachnospiraceae;D_5__Marvinbryantia	2.87(0.10, 78.33)	11.26(0.51,249.35)	3.93(0.44, 34.86)	0.16(0.01, 3.77)	0.47(0.05, 4.40)										
ASV_43	Firmicutes;D_2__Clostridia;D_3__Clostridiales;D_4__Ruminococcaceae;D_5__Ruminococcaceae UCG-005;D_6__uncultured organism;D_7__;D_8__;D_9__;D_10__;D_11__;D_12__;D_13__;D_14__	0.81(0.10, 6.36)	0.89(0.13, 6.11)	1.10(0.28, 4.29)	0.91(0.13, 6.40)	0.74(0.18, 2.98)	0.92(0.12, 7.02)	0.98(0.14, 6.98)	1.07(0.27,4.26)	0.64(0.09, 4.51)	0.60(0.15,2.39)					
ASV_46	Firmicutes;D_2__Clostridia;D_3__Clostridiales;D_4__Lachnospiraceae;D_5__Agathobacter	0.93(0.24, 3.62)	0.55(0.15, 1.96)	0.59(0.24, 1.46)	1.32(0.36, 4.81)	1.22(0.48, 3.08)	0.67(0.17, 2.57)	0.44(0.12, 1.62)	0.66(0.26,1.66)	1.75(0.48, 6.39)	1.17(0.47,2.94)					

ASV_47	Firmicutes;D_2__Clostridia;D_3__Clostridiales;D_4__Ruminococcaceae;D_5__Faecalibacterium	1.00(0.43, 2.35)	0.99(0.45, 2.19)	0.99(0.56, 1.73)	0.74(0.33, 1.66)	0.75(0.42, 1.33)	1.00(0.43, 2.33)	0.94(0.41, 2.14)	0.94(0.53,1.69)	0.77(0.34, 1.73)	0.76(0.43,1.37)					
ASV_48	Firmicutes;D_2__Clostridia;D_3__Clostridiales;D_4__Ruminococcaceae;D_5__Intestinimonas;D_6__uncultured bacterium;D_7__;D_8__;D_9__;D_10__;D_11__;D_12__;D_13__;D_14__	0.43(0.04, 4.78)	0.87(0.09, 8.09)	2.02(0.40, 10.18)	1.13(0.12, 10.84)	0.48(0.09, 2.54)	0.25(0.02, 2.66)	0.98(0.10, 9.25)	3.92(0.73,21.10)	1.33(0.14, 12.45)	0.33(0.06,1.79)					
ASV_49	Bacteroidetes;D_2__Bacteroidia;D_3__Bacteroidales;D_4__Rikenellaceae;D_5__Alistipes	0.65(0.09, 4.81)	1.39(0.21, 9.03)	2.13(0.57, 8.03)	1.36(0.20, 9.06)	0.89(0.23, 3.44)										
ASV_50	Firmicutes;D_2__Clostridia;D_3__Clostridiales;D_4__Lachnospiraceae;D_5__[Ruminococcus] gauvreauii group	3.58(0.71, 18.12)	2.53(0.55, 11.55)	0.71(0.24, 2.07)	0.32(0.07, 1.49)	1.15(0.38, 3.44)	4.04(0.80, 20.38)	2.47(0.52, 11.80)	0.61(0.20,1.85)	0.30(0.06, 1.41)	1.21(0.40,3.65)					
ASV_51	Firmicutes;D_2__Clostridia;D_3__Clostridiales;D_4__Ruminococcaceae;D_5__Subdoligranulum	2.48(0.77, 8.00)	1.79(0.60, 5.36)	0.72(0.33, 1.57)	0.41(0.14, 1.26)	1.02(0.46, 2.27)	1.90(0.60, 5.99)	1.38(0.45, 4.18)	0.73(0.33,1.59)	0.51(0.17, 1.54)	0.97(0.44,2.13)					
ASV_52	Firmicutes;D_2__Clostridia;D_3__Clostridiales;D_4__Ruminococcaceae;D_5__Ruminiclostridium 5	1.20(0.05, 26.31)	0.94(0.05, 16.92)	0.78(0.10, 6.07)	0.37(0.02, 6.99)	0.45(0.05, 3.64)	0.83(0.04, 17.92)	0.66(0.03, 12.86)	0.80(0.10,6.51)	0.60(0.03, 11.46)	0.50(0.06,4.08)					
ASV_53	Firmicutes;D_2__Clostridia;D_3__Clostridiales;D_4__Lachnospiraceae;D_5__[Ruminococcus] torques group	1.77(0.48, 6.51)	1.70(0.50, 5.74)	0.96(0.40, 2.27)	0.56(0.16, 1.93)	1.00(0.41, 2.41)	1.81(0.50, 6.58)	2.40(0.69, 8.35)	1.33(0.55,3.21)	0.45(0.13, 1.57)	0.82(0.34,1.98)					
ASV_56	Firmicutes;D_2__Bacilli;D_3__Lactobacillales;D_4__Leuconostocaceae;D_5__Weissella	0.17(0.00, 21.15)	0.23(0.00, 20.42)	1.33(0.05, 33.49)	1.47(0.02, 140.21)	0.25(0.01, 6.75)										
ASV_60	Firmicutes;D_2__Clostridia;D_3__Clostridiales;D_4__Lachnospiraceae;D_5__Blautia;D_6__uncultured Blautia sp.;D_7__;D_8__;D_9__;D_10__;D_11__;D_12__;D_13__;D_14__	2.49(0.56, 11.06)	1.96(0.48, 7.91)	0.79(0.29, 2.11)	0.39(0.10, 1.62)	0.98(0.36, 2.68)	2.69(0.61, 11.87)	2.31(0.55, 9.68)	0.86(0.31,2.36)	0.30(0.07, 1.26)	0.81(0.29,2.23)					
ASV_62	Firmicutes;D_2__Clostridia;D_3__Clostridiales;D_4__Lachnospiraceae;D_5__Agathobacter;D_6__Eubacterium ramulus;D_7__;D_8__;D_9__;D_10__;D_11__;D_12__;D_13__;D_14__	4.99(0.71, 35.07)	2.44(0.39, 15.18)	0.49(0.13, 1.78)	0.41(0.06, 2.62)	2.05(0.55, 7.70)	5.51(0.79, 38.65)	2.55(0.39, 16.70)	0.46(0.12,1.74)	0.39(0.06, 2.51)	2.14(0.56,8.08)					
ASV_65	Firmicutes;D_2__Clostridia;D_3__Clostridiales;D_4__Ruminococcaceae;D_5__uncultured;D_6__uncultured Clostridium sp.;D_7__;D_8__;D_9__;D_10__;D_11__;D_12__;D_13__;D_14__	0.27(0.03, 2.34)	0.50(0.07, 3.76)	1.86(0.44, 7.89)	1.47(0.19, 11.24)	0.40(0.09, 1.74)	0.34(0.04, 2.93)	0.65(0.08, 5.12)	1.89(0.43,8.32)	1.40(0.18, 10.86)	0.48(0.11,2.11)					
ASV_66	Firmicutes;D_2__Clostridia;D_3__Clostridiales;D_4__Ruminococcaceae;D_5__Subdoligranulum	0.94(0.25, 3.54)	0.43(0.12, 1.47)	0.45(0.19, 1.09)	2.28(0.65, 7.99)	2.16(0.88, 5.29)	0.90(0.24, 3.38)	0.39(0.11, 1.41)	0.44(0.18,1.08)	2.58(0.73, 9.19)	2.33(0.94,5.76)					
ASV_68	Firmicutes;D_2__Clostridia;D_3__Clostridiales;D_4__Family XIII;D_5__Family XIII AD3011 group;D_6__uncultured Eubacterium sp.;D_7__;D_8__;D_9__;D_10__;D_11__;D_12__;D_13__;D_14__	0.82(0.10, 6.97)	0.74(0.10, 5.52)	0.91(0.22, 3.77)	0.51(0.07, 3.88)	0.42(0.10, 1.80)	0.84(0.10, 7.14)	0.70(0.09, 5.50)	0.83(0.19,3.59)	0.52(0.07, 4.02)	0.43(0.10,1.88)					
ASV_69	Bacteroidetes;D_2__Bacteroidia;D_3__Bacteroidales;D_4__Rikenellaceae;D_5__Alistipes;D_6__Alistipes sp. N15.MGS-157;D_7__;D_8__;D_9__;D_10__;D_11__;D_12__;D_13__;D_14__	7.13(0.48, 105.78)	5.27(0.42, 66.10)	0.74(0.13, 4.34)	0.04(0.00, 0.49);p=0.01197	0.27(0.04, 1.64)	6.06(0.41, 89.70)	4.21(0.31, 56.97)	0.70(0.11,4.30)	0.04(0.00, 0.58);p=0.01746	0.26(0.04,1.63)				↓	
ASV_70	Firmicutes;D_2__Bacilli;D_3__Lactobacillales;D_4__Streptococcaceae;D_5__Streptococcus;D_6__Streptococcus anginosus subsp. anginosus;D_7__;D_8__;D_9__;D_10__;D_11__;D_12__;D_13__;D_14__	0.46(0.03, 8.17)	0.41(0.03, 6.01)	0.89(0.13, 6.35)	1.18(0.08, 17.62)	0.54(0.07, 3.94)	0.64(0.04, 10.66)	0.53(0.03, 8.09)	0.83(0.11,6.09)	1.18(0.08, 17.43)	0.75(0.11,5.34)					
ASV_72	Firmicutes;D_2__Clostridia;D_3__Clostridiales;D_4__Lachnospiraceae;D_5__Eisenbergiella;D_6__uncultured organism;D_7__;D_8__;D_9__;D_10__;D_11__;D_12__;D_13__;D_14__	2.50(0.33, 19.11)	2.20(0.33, 14.79)	0.88(0.23, 3.36)	0.25(0.04, 1.73)	0.63(0.16, 2.47)	2.23(0.29, 16.85)	1.53(0.22, 10.83)	0.69(0.17,2.72)	0.31(0.04, 2.18)	0.70(0.18,2.76)					
ASV_73	Firmicutes;D_2__Bacilli;D_3__Lactobacillales;D_4__Streptococcaceae;D_5__Streptococcus	1.72(0.27, 10.90)	0.45(0.08, 2.53)	0.26(0.08, 0.89);p=0.03131	1.13(0.20, 6.48)	1.94(0.55, 6.78)	1.69(0.26, 10.73)	0.44(0.07, 2.62)	0.26(0.07,0.92);p=0.03685	1.18(0.20, 7.01)	2.00(0.56,7.08)			↓		

ASV_74	Firmicutes;D_2__Bacilli;D_3__Bacillales;D_4__Family XI;D_5__Gemella	1.44(0.27,7.81)	0.41(0.08,1.99)	0.28(0.09,0.87);p=0.02782	0.94(0.19,4.68)	1.36(0.43,4.27)	1.20(0.23,6.31)	0.34(0.07,1.72)	0.28(0.09,0.89);p=0.03143	1.27(0.26,6.26)	1.52(0.49,4.74)			↓		
ASV_75	Firmicutes;D_2__Clostridia;D_3__Clostridiales;D_4__Lachnospiraceae;D_5__Fusicatenibacter	0.89(0.36,2.16)	1.01(0.44,2.32)	1.14(0.63,2.05)	0.88(0.38,2.06)	0.78(0.43,1.43)	0.90(0.37,2.19)	1.03(0.43,2.43)	1.15(0.62,2.11)	0.87(0.37,2.06)	0.78(0.43,1.44)					
ASV_76	Firmicutes;D_2__Clostridia;D_3__Clostridiales;D_4__Ruminococcaceae;D_5__Ruminococcaceae NK4A214 group;D_6__uncultured organism;D_7__;D_8__;D_9__;D_10__;D_11__;D_12__;D_13__;D_14__	2892179653.56(0.00,Inf)	1975641178.90(0.00,Inf)	0.68(0.04,10.49)	0.00(0.00,Inf)	0.59(0.04,9.46)										
ASV_80	Firmicutes;D_2__Clostridia;D_3__Clostridiales;D_4__Peptostreptococcaceae;D_5__Romboutsia	1.81(0.56,5.91)	0.78(0.26,2.36)	0.43(0.20,0.94);p=0.03527	0.81(0.27,2.50)	1.48(0.66,3.29)	1.88(0.58,6.14)	0.89(0.28,2.77)	0.47(0.21,1.05)	0.74(0.24,2.31)	1.40(0.62,3.14)					
ASV_81	Bacteroidetes;D_2__Bacteroidia;D_3__Bacteroidales;D_4__Tannerellaceae;D_5__Parabacteroides;D_6__Parabacteroides distasonis;D_7__;D_8__;D_9__;D_10__;D_11__;D_12__;D_13__;D_14__	4.07(0.24,70.28)	3.54(0.25,50.99)	0.87(0.13,5.73)	0.29(0.02,4.29)	1.17(0.17,8.11)	3.86(0.23,64.21)	3.36(0.22,50.69)	0.87(0.13,5.91)	0.49(0.03,7.23)	1.88(0.28,12.83)					
ASV_83	Firmicutes;D_2__Clostridia;D_3__Clostridiales;D_4__Lachnospiraceae;D_5__Roseburia;D_6__gut metagenome;D_7__;D_8__;D_9__;D_10__;D_11__;D_12__;D_13__;D_14__	0.80(0.13,5.13)	1.55(0.27,8.80)	1.93(0.57,6.62)	0.24(0.04,1.41)	0.19(0.06,0.69);p=0.01085	0.75(0.12,4.81)	1.45(0.24,8.68)	1.93(0.54,6.84)	0.25(0.04,1.49)	0.19(0.05,0.67);p=0.01007					↓
ASV_85	Firmicutes;D_2__Clostridia;D_3__Clostridiales;D_4__Ruminococcaceae;D_5__Ruminococcaceae UCG-002;D_6__uncultured rumen bacterium;D_7__;D_8__;D_9__;D_10__;D_11__;D_12__;D_13__;D_14__	0.12(0.01,1.32)	1.09(0.11,10.46)	9.28(1.86,46.38);p=0.00663	1.28(0.13,12.69)	0.15(0.03,0.78);p=0.02411	0.14(0.01,1.56)	1.05(0.10,10.65)	7.43(1.44,38.43);p=0.01673	0.95(0.10,9.43)	0.13(0.03,0.70);p=0.01684			↑		↓
ASV_86	Firmicutes;D_2__Clostridia;D_3__Clostridiales;D_4__Ruminococcaceae;D_5__Ruminococcaceae UCG-005;D_6__human gut metagenome;D_7__;D_8__;D_9__;D_10__;D_11__;D_12__;D_13__;D_14__	0.74(0.11,4.83)	0.92(0.16,5.36)	1.25(0.36,4.35)	0.73(0.12,4.35)	0.54(0.15,1.94)	0.71(0.11,4.64)	0.62(0.10,3.77)	0.87(0.24,3.12)	0.77(0.13,4.66)	0.55(0.15,1.98)					
ASV_87	Firmicutes;D_2__Clostridia;D_3__Clostridiales;D_4__Ruminococcaceae;D_5__UBA1819;D_6__uncultured organism;D_7__;D_8__;D_9__;D_10__;D_11__;D_12__;D_13__;D_14__	1.00(0.32,3.17)	0.89(0.30,2.61)	0.89(0.41,1.91)	1.19(0.40,3.54)	1.19(0.54,2.60)	0.99(0.32,3.12)	0.86(0.29,2.60)	0.87(0.40,1.90)	1.18(0.39,3.56)	1.18(0.54,2.57)					
ASV_88	Firmicutes;D_2__Erysipelotrichia;D_3__Erysipelotrichales;D_4__Erysipelotrichaceae;D_5__[Clostridium] innocuum group	2.00(0.48,8.26)	2.72(0.72,10.27)	1.36(0.54,3.46)	0.67(0.17,2.58)	1.34(0.52,3.50)	1.83(0.45,7.42)	1.89(0.49,7.31)	1.03(0.40,2.67)	0.94(0.24,3.60)	1.71(0.66,4.45)					
ASV_89	Firmicutes;D_2__Clostridia;D_3__Clostridiales;D_4__Family XIII;D_5__Family XIII AD3011 group;D_6__uncultured bacterium;D_7__;D_8__;D_9__;D_10__;D_11__;D_12__;D_13__;D_14__	1.07(0.27,4.32)	1.77(0.48,6.52)	1.65(0.66,4.15)	0.52(0.14,1.93)	0.55(0.22,1.42)	1.06(0.26,4.26)	1.59(0.41,6.09)	1.50(0.58,3.88)	0.58(0.15,2.19)	0.61(0.24,1.58)					
ASV_90	Firmicutes;D_2__Clostridia;D_3__Clostridiales;D_4__Ruminococcaceae;D_5__Faecalibacterium	0.73(0.15,3.45)	1.03(0.24,4.41)	1.41(0.51,3.95)	0.87(0.20,3.79)	0.64(0.22,1.82)	0.74(0.16,3.46)	1.30(0.29,5.73)	1.75(0.61,4.99)	0.81(0.18,3.52)	0.60(0.21,1.72)					
ASV_92	Actinobacteria;D_2__Coriobacteriia;D_3__Coriobacteriales;D_4__Eggerthellaceae;D_5__Slackia	0.75(0.01,54.48)	1.95(0.04,107.48)	2.60(0.15,44.36)	0.16(0.00,9.14)	0.12(0.01,2.17)										
ASV_93	Firmicutes;D_2__Clostridia;D_3__Clostridiales;D_4__Lachnospiraceae;D_5__Roseburia;D_6__metagenome;D_7__;D_8__;D_9__;D_10__;D_11__;D_12__;D_13__;D_14__	0.39(0.01,10.80)	0.40(0.02,8.84)	1.01(0.11,9.11)	0.15(0.01,3.55)	0.06(0.01,0.58);p=0.01461										
ASV_95	Bacteroidetes;D_2__Bacteroidia;D_3__Bacteroidales;D_4__Rikenellaceae;D_5__Alistipes	14.07(2.53,78.31);p=0.00254	14.51(2.91,72.46);p=0.00111	1.03(0.33,3.21)	0.05(0.01,0.24);p=0.00025	0.67(0.21,2.15)	16.35(2.94,91.04);p=0.00142	13.06(2.49,68.56);p=0.00239	0.80(0.25,2.57)	0.05(0.01,0.24);p=0.00024	0.74(0.23,2.40)	↑	↑		↓	
ASV_96	Firmicutes;D_2__Clostridia;D_3__Clostridiales;D_4__Lachnospiraceae;D_5__Hungatella	1.58(0.15,16.61)	3.34(0.37,30.07)	2.11(0.45,9.91)	0.86(0.09,8.00)	1.36(0.28,6.69)	1.52(0.14,15.94)	3.69(0.38,35.66)	2.43(0.49,11.99)	0.53(0.06,5.04)	0.80(0.16,3.98)					
ASV_99	Firmicutes;D_2__Clostridia;D_3__Clostridiales;D_4__Lachnospiraceae;D_5__Hungatella;D_6__uncultured bacterium;D_7__;D_8__;D_9__;D_10__;D_11__;D_12__;D_13__;D_14__	0.86(0.08,8.77)	0.57(0.06,4.98)	0.66(0.14,3.07)	1.38(0.15,12.43)	1.19(0.25,5.76)	0.42(0.04,4.06)	0.22(0.02,1.94)	0.52(0.11,2.43)	3.85(0.44,33.69)	1.64(0.35,7.73)					

ASV_100	Firmicutes;D_2__Clostridia;D_3__Clostridiales;D_4__Family XIII;D_5__Family XIII AD3011 group	0.97(0.05,20.41)	3.12(0.18,53.04)	3.20(0.43,23.76)	0.54(0.03,9.46)	0.52(0.07,4.08)	1.28(0.07,21.96)	1.39(0.09,21.65)	1.09(0.16,7.42)	0.58(0.04,8.85)	0.74(0.11,5.07)					
ASV_101	Firmicutes;D_2__Clostridia;D_3__Clostridiales;D_4__Lachnospiraceae;D_5__Dorea	1.43(0.48,4.23)	1.47(0.53,4.07)	1.03(0.50,2.11)	0.46(0.16,1.28)	0.65(0.31,1.36)	1.45(0.49,4.31)	1.49(0.52,4.26)	1.03(0.49,2.16)	0.46(0.16,1.32)	0.67(0.32,1.42)					
ASV_102	Firmicutes;D_2__Negativicutes;D_3__Selenomonadales;D_4__Veillonellaceae;D_5__Veillonella	1.84(0.25,13.34)	2.23(0.35,14.23)	1.21(0.33,4.48)	0.22(0.03,1.41)	0.40(0.10,1.52)	1.22(0.18,8.36)	1.47(0.23,9.41)	1.20(0.32,4.46)	0.22(0.04,1.41)	0.27(0.07,1.01)					
ASV_103	Firmicutes;D_2__Clostridia;D_3__Clostridiales;D_4__Lachnospiraceae;D_5__GCA-900066575;D_6__uncultured bacterium;D_7__;D_8__;D_9__;D_10__;D_11__;D_12__;D_13__;D_14__	58542955274.43(0.00,Inf)	6856709216.90(0.00,Inf)	0.12(0.00,5.91)	0.00(0.00,Inf)	8.13(0.15,453.04)										
ASV_104	Bacteroidetes;D_2__Bacteroidia;D_3__Bacteroidales;D_4__Marinifilaceae;D_5__Odoribacter	0.66(0.14,3.17)	1.02(0.24,4.40)	1.53(0.54,4.33)	0.74(0.17,3.24)	0.49(0.17,1.41)	0.77(0.16,3.65)	1.06(0.24,4.77)	1.38(0.48,4.00)	0.76(0.17,3.38)	0.58(0.20,1.69)					
ASV_106	Firmicutes;D_2__Clostridia;D_3__Clostridiales;D_4__Ruminococcaceae;D_5__Ruminiclostridium 6;D_6__uncultured bacterium;D_7__;D_8__;D_9__;D_10__;D_11__;D_12__;D_13__;D_14__	2.60(0.27,24.60)	4.76(0.58,39.14)	1.84(0.42,8.11)	0.31(0.04,2.58)	0.79(0.17,3.64)	1.00(0.15,6.76)	0.96(0.15,6.04)	0.96(0.26,3.51)	2.17(0.34,13.62)	2.16(0.58,8.01)					
ASV_107	Firmicutes;D_2__Clostridia;D_3__Clostridiales;D_4__Lachnospiraceae;D_5__CAG-56;D_6__uncultured bacterium;D_7__;D_8__;D_9__;D_10__;D_11__;D_12__;D_13__;D_14__	0.17(0.02,1.44)	0.25(0.03,1.88)	1.50(0.36,6.28)	3.38(0.44,26.08)	0.56(0.13,2.43)										
ASV_109	Firmicutes;D_2__Clostridia;D_3__Clostridiales;D_4__Lachnospiraceae;D_5__Lachnospiraceae FCS020 group;D_6__uncultured organism;D_7__;D_8__;D_9__;D_10__;D_11__;D_12__;D_13__;D_14__	0.50(0.13,1.97)	0.48(0.13,1.73)	0.96(0.39,2.38)	1.51(0.41,5.51)	0.76(0.30,1.92)	0.54(0.14,2.11)	0.55(0.15,2.07)	1.03(0.40,2.62)	1.34(0.36,4.96)	0.72(0.28,1.83)					
ASV_112	Firmicutes;D_2__Clostridia;D_3__Clostridiales;D_4__Ruminococcaceae	2.01(0.01,353.77)	0.98(0.01,124.07)	0.49(0.02,14.95)	0.39(0.00,52.78)	0.79(0.02,26.21)										
ASV_119	Bacteroidetes;D_2__Bacteroidia;D_3__Bacteroidales;D_4__Bacteroidaceae;D_5__Bacteroides	0.26(0.02,3.15)	0.91(0.09,9.54)	3.55(0.67,18.67)	1.38(0.13,14.86)	0.36(0.06,1.95)	0.21(0.02,2.63)	0.98(0.09,11.07)	4.59(0.83,25.40)	1.16(0.10,12.84)	0.25(0.04,1.38)					
ASV_122	Firmicutes;D_2__Clostridia;D_3__Clostridiales;D_4__Lachnospiraceae;D_5__Eisenbergiella	1817625072.86(0.00,Inf)	6935251064.03(0.00,Inf)	3.82(0.09,162.31)	0.00(0.00,Inf)	0.88(0.02,47.50)										
ASV_123	Firmicutes;D_2__Clostridia;D_3__Clostridiales;D_4__Ruminococcaceae;D_5__uncultured						1.28(0.01,174.88)	0.00(0.00,Inf)	0.00(0.00,Inf)	0.40(0.00,39.11)	0.51(0.03,10.28)					
ASV_124	Proteobacteria;D_2__Deltaproteobacteria;D_3__Desulfovibrionales;D_4__Desulfovibrionaceae;D_5__Bilophila;D_6__uncultured bacterium;D_7__;D_8__;D_9__;D_10__;D_11__;D_12__;D_13__;D_14__	1.33(0.25,7.08)	1.59(0.33,7.59)	1.19(0.39,3.60)	0.88(0.18,4.30)	1.18(0.38,3.66)	1.54(0.29,8.14)	2.14(0.43,10.66)	1.39(0.45,4.31)	0.82(0.17,4.03)	1.26(0.40,3.94)					
ASV_126	Firmicutes;D_2__Clostridia;D_3__Clostridiales;D_4__Christensenellaceae;D_5__Christensenellaceae R-7 group	740338946.89(0.00,Inf)	12605554635.58(0.00,Inf)	17.03(1.44,201.83);p=0.02464	0.00(0.00,Inf)	0.12(0.01,1.56)										
ASV_127	Actinobacteria;D_2__Actinobacteria;D_3__Actinomycetales;D_4__Actinomycetaceae;D_5__Actinomyces	2.26(0.65,7.91)	0.59(0.18,1.91)	0.26(0.11,0.60);p=0.00149	0.61(0.19,1.99)	1.37(0.59,3.20)										

ASV_128	Firmicutes;D_2__Clostridia;D_3__Clostridiales;D_4__Family XIII;D_5__[Eubacterium] brachy group;D_6__uncultured bacterium;D_7__;D_8__;D_9__;D_10__;D_11__;D_12__;D_13__;D_14__	0.77(0.13, 4.62)	0.62(0.12, 3.32)	0.81(0.24, 2.65)	0.91(0.17, 4.94)	0.70(0.21, 2.37)	0.63(0.11, 3.63)	0.44(0.08, 2.38)	0.69(0.21,2.31)	1.12(0.21, 5.95)	0.71(0.21,2.35)					
ASV_129	Bacteroidetes;D_2__Bacteroidia;D_3__Bacteroidales;D_4__Tannerellaceae;D_5__Parabacteroides;D_6__Parabacteroides distasonis;D_7__;D_8__;D_9__;D_10__;D_11__;D_12__;D_13__;D_14__	0.29(0.01, 14.11)	0.56(0.01, 21.63)	1.97(0.15, 26.11)	1.40(0.03, 56.31)	0.40(0.03, 5.64)										
ASV_131	Firmicutes;D_2__Clostridia;D_3__Clostridiales;D_4__Lachnospiraceae;D_5__Lachnoclostridium	0.42(0.11, 1.57)	0.63(0.18, 2.20)	1.52(0.63, 3.68)	2.28(0.65, 8.03)	0.95(0.38, 2.35)	0.46(0.12, 1.73)	0.82(0.23, 2.95)	1.79(0.72,4.45)	1.91(0.54, 6.83)	0.88(0.35,2.18)					
ASV_132	Firmicutes;D_2__Clostridia;D_3__Clostridiales;D_4__Lachnospiraceae;D_5__[Eubacterium] eligens group	1.35(0.18, 10.35)	1.94(0.29, 13.04)	1.43(0.37, 5.53)	0.20(0.03, 1.37)	0.27(0.07, 1.07)	1.42(0.18, 10.93)	1.79(0.25, 12.85)	1.26(0.31,5.08)	0.20(0.03, 1.40)	0.28(0.07,1.14)					
ASV_134	Firmicutes;D_2__Clostridia;D_3__Clostridiales;D_4__Ruminococcaceae;D_5__Ruminococcaceae UCG-013;D_6__uncultured organism;D_7__;D_8__;D_9__;D_10__;D_11__;D_12__;D_13__;D_14__	1.27(0.43, 3.80)	1.84(0.66, 5.11)	1.44(0.70, 2.98)	0.76(0.27, 2.15)	0.97(0.46, 2.04)	1.31(0.44, 3.91)	2.15(0.75, 6.16)	1.64(0.78,3.45)	0.67(0.24, 1.92)	0.89(0.42,1.87)					
ASV_135	Firmicutes;D_2__Clostridia;D_3__Clostridiales;D_4__Lachnospiraceae;D_5__Roseburia	1.29(0.25, 6.72)	1.52(0.32, 7.13)	1.18(0.39, 3.52)	0.27(0.06, 1.31)	0.35(0.11, 1.08)	0.72(0.14, 3.61)	0.86(0.18, 4.07)	1.19(0.40,3.59)	0.47(0.10, 2.23)	0.34(0.11,1.03)					
ASV_136	Firmicutes;D_2__Clostridia;D_3__Clostridiales;D_4__Ruminococcaceae;D_5__Ruminiclostridium 5;D_6__uncultured organism;D_7__;D_8__;D_9__;D_10__;D_11__;D_12__;D_13__;D_14__	1.62(0.55, 4.72)	1.27(0.46, 3.45)	0.78(0.38, 1.59)	0.92(0.33, 2.53)	1.48(0.72, 3.06)	1.56(0.54, 4.55)	1.16(0.41, 3.25)	0.74(0.36,1.53)	0.97(0.35, 2.70)	1.51(0.73,3.13)					
ASV_137	Firmicutes;D_2__Negativicutes;D_3__Selenomonadales;D_4__Veillonellaceae;D_5__Veillonella	1.09(0.16, 7.48)	3.86(0.64, 23.30)	3.54(0.99, 12.60)	0.54(0.09, 3.34)	0.59(0.16, 2.16)	0.86(0.13, 5.70)	2.21(0.36, 13.67)	2.58(0.71,9.36)	0.66(0.11, 4.08)	0.57(0.16,2.08)					
ASV_141	Firmicutes;D_2__Clostridia;D_3__Clostridiales;D_4__Ruminococcaceae;D_5__Ruminiclostridium 5;D_6__gut metagenome;D_7__;D_8__;D_9__;D_10__;D_11__;D_12__;D_13__;D_14__	2.44(0.61, 9.75)	2.68(0.73, 9.80)	1.10(0.44, 2.74)	0.40(0.11, 1.49)	0.98(0.38, 2.50)	2.38(0.60, 9.54)	2.42(0.64, 9.25)	1.02(0.40,2.62)	0.43(0.11, 1.64)	1.03(0.40,2.67)					
ASV_144	Firmicutes;D_2__Bacilli;D_3__Lactobacillales;D_4__Carnobacteriaceae;D_5__Granulicatella	1.25(0.31, 4.96)	0.45(0.12, 1.64)	0.36(0.14, 0.90);p=0.02928	1.11(0.30, 4.10)	1.39(0.54, 3.53)	1.18(0.30, 4.70)	0.42(0.11, 1.62)	0.36(0.14,0.93);p=0.03455	1.21(0.32, 4.55)	1.42(0.55,3.66)			↓		
ASV_146	Firmicutes;D_2__Negativicutes;D_3__Selenomonadales;D_4__Veillonellaceae;D_5__Veillonella	3.47(0.49, 24.73)	3.54(0.56, 22.34)	1.02(0.29, 3.63)	0.05(0.01, 0.35);p=0.00218	0.19(0.05, 0.69);p=0.01189	3.21(0.46, 22.56)	3.48(0.53, 23.00)	1.09(0.30,3.98)	0.05(0.01, 0.35);p=0.00214	0.17(0.05,0.62);p=0.00748				↓	↓
ASV_148	Firmicutes;D_2__Clostridia;D_3__Clostridiales;D_4__Ruminococcaceae	0.62(0.09, 4.08)	0.58(0.10, 3.38)	0.94(0.26, 3.32)	1.17(0.20, 6.92)	0.73(0.20, 2.64)	0.59(0.09, 3.67)	0.60(0.10, 3.53)	1.03(0.28,3.72)	0.87(0.15, 4.98)	0.51(0.14,1.83)					
ASV_150	Firmicutes;D_2__Bacilli;D_3__Lactobacillales;D_4__Streptococcaceae;D_5__Lactococcus	1.84(0.30, 11.33)	0.40(0.07, 2.21)	0.22(0.07, 0.73);p=0.01331	3.54(0.63, 19.84)	6.52(1.90, 22.42);p=0.00291	1.94(0.33, 11.56)	0.71(0.13, 3.98)	0.37(0.11,1.23)	2.82(0.51, 15.64)	5.49(1.62,18.61);p=0.0063					↑
ASV_153	Firmicutes;D_2__Clostridia;D_3__Clostridiales;D_4__Lachnospiraceae;D_5__Lachnospiraceae NK4A136 group;D_6__uncultured organism;D_7__;D_8__;D_9__;D_10__;D_11__;D_12__;D_13__;D_14__	1.08(0.20, 5.91)	2.07(0.42, 10.15)	1.91(0.62, 5.89)	0.55(0.11, 2.73)	0.59(0.19, 1.88)	0.89(0.16, 4.81)	1.95(0.38, 9.97)	2.20(0.69,6.97)	0.82(0.16, 4.15)	0.72(0.23,2.31)					
ASV_154	Bacteroidetes;D_2__Bacteroidia;D_3__Bacteroidales;D_4__Bacteroidaceae;D_5__Bacteroides	4.37(0.43, 44.84)	3.12(0.35, 27.59)	0.71(0.15, 3.34)	0.42(0.05, 3.83)	1.84(0.38, 8.95)	5.89(0.58, 59.77)	3.48(0.37, 32.57)	0.59(0.12,2.87)	0.48(0.05, 4.47)	2.85(0.58,13.91)					

ASV_155	Firmicutes;D_2__Erysipelotrichia;D_3__Erysipelotrichales;D_4__Erysipelotrichaceae;D_5__Erysipelotrichaceae UCG-003	3.03(0.71,12.88)	2.48(0.64,9.61)	0.82(0.31,2.13)	0.27(0.07,1.07)	0.82(0.31,2.20)	3.27(0.78,13.73)	3.24(0.81,12.93)	0.99(0.37,2.63)	0.22(0.06,0.88);p=0.0325	0.73(0.27,1.94)				↓	
ASV_157	Firmicutes;D_2__Clostridia;D_3__Clostridiales;D_4__Peptostreptococcaceae;D_5__Peptostreptococcus;D_6__uncultured organism;D_7__;D_8__;D_9__;D_10__;D_11__;D_12__;D_13__;D_14__	0.14(0.01,3.08)	0.13(0.01,2.29)	0.92(0.11,7.78)	5.59(0.30,103.86)	0.77(0.09,6.79)	0.17(0.01,3.73)	0.25(0.01,4.81)	1.49(0.17,13.36)	3.47(0.19,64.31)	0.59(0.07,5.27)					
ASV_158	Firmicutes;D_2__Clostridia;D_3__Clostridiales;D_4__Lachnospiraceae;D_5__Lachnoclostridium;D_6__human gut metagenome;D_7__;D_8__;D_9__;D_10__;D_11__;D_12__;D_13__;D_14__	0.54(0.14,2.12)	0.86(0.24,3.09)	1.59(0.64,3.93)	1.96(0.54,7.16)	1.06(0.42,2.69)	0.56(0.14,2.19)	1.09(0.29,4.05)	1.94(0.76,4.94)	1.49(0.40,5.51)	0.83(0.33,2.12)					
ASV_160	Firmicutes;D_2__Clostridia;D_3__Clostridiales;D_4__Ruminococcaceae	0.86(0.02,47.80)	1.58(0.04,65.72)	1.84(0.13,26.35)	0.34(0.01,14.47)	0.29(0.02,4.31)	0.54(0.01,29.40)	1.53(0.03,68.70)	2.85(0.17,46.59)	0.40(0.01,17.32)	0.22(0.01,3.47)					
ASV_161	Firmicutes;D_2__Clostridia;D_3__Clostridiales;D_4__Defluviitaleaceae;D_5__Defluviitaleaceae UCG-011;D_6__uncultured bacterium;D_7__;D_8__;D_9__;D_10__;D_11__;D_12__;D_13__;D_14__	2.42(0.16,37.54)	5.47(0.42,71.43)	2.27(0.38,13.44)	0.19(0.01,2.55)	0.46(0.07,2.84)	2.35(0.15,36.31)	5.23(0.37,73.31)	2.22(0.36,13.76)	0.20(0.01,2.82)	0.48(0.08,3.00)					
ASV_163	Firmicutes;D_2__Clostridia;D_3__Clostridiales;D_4__Ruminococcaceae	0.15(0.01,4.04)	0.19(0.01,4.12)	1.30(0.14,12.41)	2.03(0.09,44.11)	0.30(0.03,2.99)										
ASV_167	Firmicutes;D_2__Clostridia;D_3__Clostridiales;D_4__Clostridiales vadinBB60 group;D_5__uncultured Thermoanaerobacterales bacterium;D_6__;D_7__;D_8__;D_9__;D_10__;D_11__;D_12__;D_13__;D_14__	2.41(0.01,695.24)	0.24(0.00,47.92)	0.10(0.00,4.21)	0.16(0.00,34.13)	0.38(0.01,17.85)										
ASV_168	Firmicutes;D_2__Clostridia;D_3__Clostridiales;D_4__Lachnospiraceae	0.39(0.06,2.45)	1.17(0.21,6.51)	3.02(0.88,10.37)	0.48(0.08,2.74)	0.19(0.05,0.65);p=0.00883	0.48(0.08,3.00)	2.19(0.37,12.77)	4.59(1.29,16.36);p=0.01874	0.29(0.05,1.67)	0.14(0.04,0.49);p=0.00221			↑		↓
ASV_169	Firmicutes;D_2__Clostridia;D_3__Clostridiales;D_4__Lachnospiraceae;D_5__[Eubacterium] eligens group	1.40(0.12,16.89)	0.85(0.08,8.75)	0.61(0.12,3.16)	0.21(0.02,2.27)	0.30(0.06,1.62)	1.19(0.10,14.37)	0.64(0.06,7.07)	0.54(0.10,2.93)	0.31(0.03,3.36)	0.37(0.07,2.02)					
ASV_170	Firmicutes;D_2__Clostridia;D_3__Clostridiales;D_4__Lachnospiraceae;D_5__Lachnospiraceae NK4A136 group	1.51(0.30,7.46)	3.68(0.82,16.42)	2.44(0.85,7.02)	0.14(0.03,0.66);p=0.0122	0.22(0.07,0.64);p=0.0057	1.39(0.29,6.58)	3.01(0.67,13.48)	2.16(0.75,6.23)	0.17(0.04,0.74);p=0.01857	0.23(0.08,0.67);p=0.00688				↓	↓
ASV_171	Firmicutes;D_2__Clostridia;D_3__Clostridiales;D_4__Ruminococcaceae;D_5__Ruminococcaceae NK4A214 group;D_6__uncultured organism;D_7__;D_8__;D_9__;D_10__;D_11__;D_12__;D_13__;D_14__	0.10(0.00,2.32)	0.32(0.02,5.82)	3.12(0.39,25.35)	2.79(0.15,52.67)	0.29(0.03,2.44)										
ASV_172	Firmicutes;D_2__Erysipelotrichia;D_3__Erysipelotrichales;D_4__Erysipelotrichaceae;D_5__Holdemania	1.43(0.25,8.30)	0.98(0.19,5.08)	0.68(0.21,2.19)	2.22(0.42,11.84)	3.17(0.96,10.55)	1.55(0.27,8.74)	0.66(0.12,3.51)	0.42(0.13,1.38)	3.07(0.58,16.31)	4.75(1.44,15.68);p=0.01048					↑
ASV_173	Firmicutes;D_2__Clostridia;D_3__Clostridiales;D_4__Lachnospiraceae;D_5__Sellimonas;D_6__uncultured bacterium;D_7__;D_8__;D_9__;D_10__;D_11__;D_12__;D_13__;D_14__	77276149084.62(0.00,Inf)	181653744249.98(0.00,Inf)	2.35(0.13,41.22)	0.00(0.00,Inf)	6.99(0.32,153.17)	1744480822546475.00(0.00,Inf)	5978728971867738.00(0.00,Inf)	3.43(0.25,47.53)	0.00(0.00,Inf)	16.48(0.52,517.99)					
ASV_174	Firmicutes;D_2__Clostridia;D_3__Clostridiales;D_4__Lachnospiraceae;D_5__uncultured;D_6__uncultured organism;D_7__;D_8__;D_9__;D_10__;D_11__;D_12__;D_13__;D_14__	2.10(0.26,16.69)	1.00(0.14,6.96)	0.48(0.12,1.88)	1.22(0.17,8.76)	2.56(0.63,10.48)	1.95(0.24,15.56)	1.06(0.14,7.86)	0.54(0.13,2.23)	1.30(0.18,9.58)	2.55(0.61,10.54)					
ASV_175	Firmicutes;D_2__Clostridia;D_3__Clostridiales;D_4__Christensenellaceae;D_5__uncultured;D_6__uncultured bacterium;D_7__;D_8__;D_9__;D_10__;D_11__;D_12__;D_13__;D_14__	1.10(0.10,11.95)	3.13(0.34,28.68)	2.84(0.60,13.48)	0.23(0.02,2.17)	0.26(0.05,1.25)	0.94(0.09,10.06)	3.28(0.34,31.80)	3.49(0.70,17.49)	0.27(0.03,2.55)	0.25(0.05,1.25)					

ASV_177	Firmicutes;D_2__Clostridia;D_3__Clostridiales;D_4__Christensenellaceae;D_5__Christensenellaceae R-7 group	0.10(0.00, 9.37)	0.47(0.01, 30.47)	4.49(0.22, 91.85)	7.77(0.11, 538.04)	0.81(0.04, 18.31)										
ASV_179	Firmicutes;D_2__Clostridia;D_3__Clostridiales;D_4__Ruminococcaceae;D_5__DTU089	1.23(0.30, 5.05)	1.45(0.39, 5.45)	1.18(0.46, 3.00)	1.26(0.33, 4.81)	1.55(0.59, 4.05)										
ASV_180	Actinobacteria;D_2__Coriobacteriia;D_3__Coriobacteriales;D_4__Coriobacteriales Incertae Sedis;D_5__uncultured;D_6__gut metagenome;D_7__;D_8__;D_9__;D_10__;D_11__;D_12__;D_13__;D_14__	1.89(0.11, 32.36)	1.97(0.14, 28.08)	1.04(0.16, 6.81)	0.76(0.05, 11.16)	1.43(0.21, 9.85)	2.80(0.17, 47.43)	2.24(0.15, 34.44)	0.80(0.12,5.52)	0.51(0.03, 7.67)	1.42(0.20,9.83)					
ASV_181	Firmicutes;D_2__Clostridia;D_3__Clostridiales;D_4__Ruminococcaceae;D_5__Anaerotruncus;D_6__Anaerotruncus sp. AT3;D_7__;D_8__;D_9__;D_10__;D_11__;D_12__;D_13__;D_14__	2901682821.88(0.00,Inf)	6066565757.00(0.00,Inf)	2.09(0.19, 22.96)	0.00(0.00, Inf)	0.93(0.08, 10.97)										
ASV_182	Firmicutes;D_2__Clostridia;D_3__Clostridiales;D_4__Lachnospiraceae	1.43(0.48, 4.23)	0.74(0.27, 2.06)	0.52(0.25, 1.06)	1.02(0.36, 2.85)	1.45(0.70, 3.03)	1.37(0.46, 4.05)	0.68(0.24, 1.96)	0.50(0.24,1.05)	1.10(0.39, 3.13)	1.50(0.72,3.15)					
ASV_183	Actinobacteria;D_2__Coriobacteriia;D_3__Coriobacteriales;D_4__Eggerthellaceae	1.38(0.27, 6.99)	1.54(0.34, 6.99)	1.11(0.38, 3.23)	0.79(0.17, 3.67)	1.09(0.37, 3.27)	1.61(0.32, 8.17)	1.80(0.38, 8.62)	1.12(0.37,3.35)	0.66(0.14, 3.15)	1.07(0.35,3.22)					
ASV_184	Firmicutes;D_2__Clostridia;D_3__Clostridiales;D_4__Ruminococcaceae;D_5__uncultured	2.51(0.19, 34.09)	3.18(0.28, 36.56)	1.26(0.23, 7.01)	0.15(0.01, 1.81)	0.38(0.07, 2.22)										
ASV_185	Firmicutes;D_2__Clostridia;D_3__Clostridiales;D_4__Ruminococcaceae;D_5__Anaerotruncus;D_6__Anaerotruncus rubiinfantis;D_7__;D_8__;D_9__;D_10__;D_11__;D_12__;D_13__;D_14__	0.84(0.06, 11.68)	0.65(0.06, 7.68)	0.78(0.13, 4.65)	0.59(0.05, 6.87)	0.49(0.08, 2.92)	0.81(0.06, 11.60)	1.00(0.08, 12.91)	1.23(0.19,8.02)	0.42(0.03, 5.19)	0.34(0.06,2.13)					
ASV_187	Firmicutes;D_2__Clostridia;D_3__Clostridiales;D_4__Christensenellaceae;D_5__uncultured	40586639377.94(0.00,Inf)	69533395690.48(0.00,Inf)	1.71(0.11, 26.69)	0.00(0.00, Inf)	0.99(0.06, 17.24)										
ASV_189	Firmicutes;D_2__Clostridia;D_3__Clostridiales;D_4__Lachnospiraceae;D_5__[Eubacterium] ventriosum group	2.20(0.44, 11.04)	1.32(0.29, 5.97)	0.60(0.21, 1.74)	0.37(0.08, 1.69)	0.81(0.27, 2.41)	1.35(0.27, 6.73)	1.01(0.21, 4.73)	0.74(0.25,2.22)	0.42(0.09, 1.95)	0.57(0.19,1.70)					
ASV_191	Proteobacteria;D_2__Gammaproteobacteria;D_3__Pseudomonadales;D_4__Pseudomonadaceae;D_5__Pseudomonas	555971289946.95(0.00,Inf)	37936468453.89(0.00,Inf)	0.07(0.02, 0.28);p=0.00023	0.00(0.00, Inf)	4.11(0.97, 17.32)	488802013255023808.00(0.00,Inf)	18940430161487368.00(0.00,Inf)	0.04(0.01,0.16);p=0.00001;q=0.02286	0.00(0.00, Inf)	8.48(2.08,34.56);p=0.00285			↓*		↑
ASV_194	Firmicutes;D_2__Clostridia;D_3__Clostridiales;D_4__Ruminococcaceae;D_5__Fournierella;D_6__uncultured bacterium;D_7__;D_8__;D_9__;D_10__;D_11__;D_12__;D_13__;D_14__	0.77(0.02, 29.32)	4.75(0.16, 142.56)	6.16(0.55, 68.54)	1.08(0.03, 33.77)	0.83(0.07, 9.84)										
ASV_196	Firmicutes;D_2__Clostridia;D_3__Clostridiales;D_4__Ruminococcaceae;D_5__Angelakisella;D_6__uncultured bacterium;D_7__;D_8__;D_9__;D_10__;D_11__;D_12__;D_13__;D_14__						0.38(0.02, 8.96)	0.24(0.01, 5.01)	0.62(0.07,5.50)	1.58(0.08, 32.74)	0.60(0.07,5.25)					
ASV_197	Firmicutes;D_2__Erysipelotrichia;D_3__Erysipelotrichales;D_4__Erysipelotrichaceae;D_5__Erysipelatoclostridium;D_6__unidentified;D_7__;D_8__;D_9__;D_10__;D_11__;D_12__;D_13__;D_14__	8698847832.87(0.00,Inf)	11046698734.51(0.00,Inf)	1.27(0.04, 36.13)	0.00(0.00, Inf)	2.60(0.08, 81.58)										
ASV_198	Actinobacteria;D_2__Coriobacteriia;D_3__Coriobacteriales;D_4__Eggerthellaceae;D_5__Gordonibacter;D_6__unidentified;D_7__;D_8__;D_9__;D_10__;D_11__;D_12__;D_13__;D_14__	0.53(0.16, 1.77)	0.52(0.17, 1.60)	0.98(0.44, 2.19)	1.34(0.43, 4.17)	0.71(0.31, 1.61)	0.56(0.17, 1.85)	0.50(0.16, 1.58)	0.89(0.39,2.02)	1.37(0.44, 4.33)	0.77(0.34,1.75)					

ASV_199	Firmicutes;D_2__Clostridia;D_3__Clostridiales;D_4__Lachnospiraceae;D_5__Lachnospiraceae UCG-010;D_6__uncultured organism;D_7__;D_8__;D_9__;D_10__;D_11__;D_12__;D_13__;D_14__	0.17(0.02,1.64)	0.68(0.08,5.74)	4.07(0.89,18.56)	1.11(0.13,9.60)	0.19(0.04,0.88);p=0.03375	0.22(0.02,2.14)	1.03(0.11,9.24)	4.70(0.99,22.38)	1.04(0.12,9.19)	0.23(0.05,1.08)					
ASV_202	Firmicutes;D_2__Clostridia;D_3__Clostridiales;D_4__Lachnospiraceae;D_5__GCA-900066755;D_6__uncultured bacterium;D_7__;D_8__;D_9__;D_10__;D_11__;D_12__;D_13__;D_14__	1.04(0.09,11.78)	1.76(0.18,17.00)	1.70(0.34,8.42)	1.11(0.11,11.13)	1.16(0.22,6.04)	0.71(0.06,7.77)	1.00(0.10,10.04)	1.42(0.28,7.32)	1.59(0.16,15.87)	1.13(0.22,5.89)					
ASV_203	Firmicutes;D_2__Clostridia;D_3__Clostridiales;D_4__Christensenellaceae;D_5__Christensenellaceae R-7 group;D_6__uncultured soil bacterium;D_7__;D_8__;D_9__;D_10__;D_11__;D_12__;D_13__;D_14__	0.58(0.03,11.71)	0.22(0.01,3.87)	0.39(0.05,3.13)	2.49(0.14,43.39)	1.44(0.18,11.69)	0.66(0.03,13.07)	0.15(0.01,2.80)	0.22(0.03,1.90)	2.98(0.17,53.09)	1.97(0.24,15.95)					
ASV_204	Firmicutes;D_2__Clostridia;D_3__Clostridiales;D_4__Christensenellaceae;D_5__uncultured;D_6__uncultured bacterium;D_7__;D_8__;D_9__;D_10__;D_11__;D_12__;D_13__;D_14__	0.19(0.01,5.57)	0.53(0.02,11.98)	2.80(0.28,27.95)	1.73(0.07,40.86)	0.32(0.03,3.42)	0.22(0.01,6.50)	0.60(0.02,15.33)	2.77(0.26,29.80)	1.42(0.06,35.41)	0.31(0.03,3.32)					
ASV_206	Actinobacteria;D_2__Coriobacteriia;D_3__Coriobacteriales;D_4__Eggerthellaceae;D_5__Gordonibacter;D_6__uncultured bacterium;D_7__;D_8__;D_9__;D_10__;D_11__;D_12__;D_13__;D_14__	15.89(0.38,672.11)	3.75(0.11,130.49)	0.24(0.02,2.44)	0.31(0.01,11.23)	4.92(0.45,54.25)	17.59(0.46,666.05)	2.95(0.08,103.19)	0.17(0.02,1.71)	0.42(0.01,14.49)	7.40(0.72,76.52)					
ASV_207	Firmicutes;D_2__Clostridia;D_3__Clostridiales;D_4__Lachnospiraceae;D_5__Lachnoclostridium	5726543717.04(0.00,Inf)	3021695396.46(0.00,Inf)	0.53(0.02,11.97)	0.00(0.00,Inf)	0.45(0.02,9.45)	1430869905656308.00(0.00,Inf)	1760610280931246.00(0.00,Inf)	1.23(0.05,33.23)	0.00(0.00,Inf)	0.27(0.01,6.50)					
ASV_209	Firmicutes;D_2__Clostridia;D_3__Clostridiales;D_4__Lachnospiraceae;D_5__GCA-900066575;D_6__uncultured organism;D_7__;D_8__;D_9__;D_10__;D_11__;D_12__;D_13__;D_14__	0.38(0.03,4.91)	0.37(0.03,4.05)	0.98(0.18,5.44)	1.14(0.10,12.79)	0.43(0.08,2.50)	0.48(0.04,6.11)	0.30(0.03,3.51)	0.63(0.11,3.60)	1.79(0.16,20.21)	0.87(0.15,4.96)					
ASV_210	Firmicutes;D_2__Clostridia;D_3__Clostridiales;D_4__Christensenellaceae;D_5__Christensenellaceae R-7 group	3.68(0.05,283.84)	0.73(0.01,44.53)	0.20(0.01,3.46)	0.45(0.01,28.27)	1.67(0.09,30.10)										
ASV_211	Firmicutes;D_2__Clostridia;D_3__Clostridiales;D_4__Christensenellaceae;D_5__uncultured;D_6__uncultured bacterium;D_7__;D_8__;D_9__;D_10__;D_11__;D_12__;D_13__;D_14__	0.24(0.00,19.04)	1.59(0.03,91.40)	6.59(0.35,122.37)	0.32(0.01,19.23)	0.08(0.00,1.53)										
ASV_212	Actinobacteria;D_2__Coriobacteriia;D_3__Coriobacteriales;D_4__Eggerthellaceae;D_5__Adlercreutzia;D_6__uncultured bacterium;D_7__;D_8__;D_9__;D_10__;D_11__;D_12__;D_13__;D_14__	0.90(0.22,3.72)	0.85(0.23,3.22)	0.95(0.37,2.43)	1.28(0.33,4.91)	1.15(0.44,3.02)	0.89(0.21,3.67)	0.83(0.21,3.26)	0.93(0.35,2.46)	1.29(0.33,5.05)	1.14(0.43,3.03)					
ASV_214	Actinobacteria;D_2__Actinobacteria;D_3__Actinomycetales;D_4__Actinomycetaceae;D_5__Actinomyces	1.68(0.01,297.00)	0.50(0.00,71.72)	0.30(0.01,9.86)	2.02(0.01,312.83)	3.39(0.09,124.89)										
ASV_215	Firmicutes;D_2__Clostridia;D_3__Clostridiales;D_4__Lachnospiraceae;D_5__Lachnoclostridium	2.22(0.37,13.33)	1.65(0.31,8.88)	0.75(0.23,2.44)	0.84(0.15,4.59)	1.86(0.55,6.26)	2.07(0.35,12.35)	1.50(0.27,8.41)	0.72(0.21,2.44)	0.81(0.14,4.48)	1.67(0.49,5.65)					
ASV_216	Firmicutes;D_2__Clostridia;D_3__Clostridiales;D_4__Lachnospiraceae;D_5__Lachnospiraceae NK4A136 group;D_6__uncultured organism;D_7__;D_8__;D_9__;D_10__;D_11__;D_12__;D_13__;D_14__	0.12(0.02,0.69);p=0.01827	0.72(0.14,3.83)	6.24(1.89,20.61);p=0.00268	1.42(0.26,7.71)	0.16(0.05,0.56);p=0.00379	0.04(0.01,0.23);p=0.0003	0.42(0.08,2.15)	10.00(3.04,32.90);p=0.00015	3.49(0.68,17.92)	0.15(0.04,0.48);p=0.00157	↓		↑		↓
ASV_218	Firmicutes;D_2__Clostridia;D_3__Clostridiales;D_4__Ruminococcaceae;D_5__Ruminococcaceae UCG-003;D_6__uncultured organism;D_7__;D_8__;D_9__;D_10__;D_11__;D_12__;D_13__;D_14__	1.40(0.22,9.03)	5.55(0.97,31.65)	3.95(1.15,13.53);p=0.02877	0.11(0.02,0.67);p=0.01619	0.16(0.05,0.57);p=0.00459	1.74(0.27,11.14)	6.85(1.14,41.04);p=0.03521	3.93(1.11,13.92);p=0.03379	0.10(0.02,0.57);p=0.00996	0.17(0.05,0.60);p=0.00573		↑	↑	↓	↓
ASV_219	Firmicutes;D_2__Clostridia;D_3__Clostridiales;D_4__Christensenellaceae;D_5__Christensenellaceae R-7 group	0.83(0.04,18.73)	0.68(0.04,12.51)	0.82(0.10,6.49)	0.63(0.03,12.05)	0.53(0.06,4.36)										

ASV_220	Firmicutes;D_2__Clostridia;D_3__Clostridiales;D_4__Ruminococcaceae;D_5__Hydrogenoanaerobacterium	0.00(0.00,Inf)	0.40(0.01,16.18)	80872897651.58(0.00,Inf)	0.30(0.01,11.57)	0.00(0.00,Inf)										
ASV_222	Firmicutes;D_2__Clostridia;D_3__Clostridiales;D_4__Lachnospiraceae;D_5__uncultured;D_6__intestinal bacterium CG19-1;D_7__;D_8__;D_9__;D_10__;D_11__;D_12__;D_13__;D_14__	0.54(0.02,12.59)	0.83(0.04,15.68)	1.54(0.19,12.61)	1.09(0.06,21.39)	0.59(0.07,5.06)	0.84(0.04,18.87)	1.20(0.06,24.03)	1.43(0.17,12.00)	0.94(0.05,18.59)	0.79(0.09,6.70)					
ASV_224	Firmicutes;D_2__Clostridia;D_3__Clostridiales;D_4__Ruminococcaceae;D_5__Ruminococcaceae UCG-007	0.93(0.03,27.57)	0.26(0.01,6.59)	0.28(0.03,2.93)	0.47(0.02,11.36)	0.43(0.04,4.27)										
ASV_225	Firmicutes;D_2__Clostridia;D_3__Clostridiales;D_4__Lachnospiraceae;D_5__uncultured	0.99(0.05,20.91)	1.22(0.07,21.04)	1.23(0.16,9.22)	0.61(0.03,10.95)	0.61(0.08,4.80)	1.77(0.09,36.37)	1.08(0.06,20.16)	0.61(0.08,4.79)	0.51(0.03,9.38)	0.91(0.12,7.10)					
ASV_227	Firmicutes;D_2__Clostridia;D_3__Clostridiales;D_4__Lachnospiraceae;D_5__Lachnospiraceae NK4A136 group;D_6__uncultured organism;D_7__;D_8__;D_9__;D_10__;D_11__;D_12__;D_13__;D_14__	0.38(0.08,1.75)	0.86(0.21,3.59)	2.27(0.82,6.25)	1.40(0.33,5.95)	0.53(0.19,1.50)	0.29(0.06,1.33)	0.70(0.16,3.04)	2.41(0.85,6.82)	1.91(0.44,8.22)	0.55(0.20,1.57)					
ASV_231	Firmicutes;D_2__Clostridia;D_3__Clostridiales;D_4__Lachnospiraceae;D_5__GCA-900066575;D_6__uncultured bacterium;D_7__;D_8__;D_9__;D_10__;D_11__;D_12__;D_13__;D_14__	3.08(0.23,41.27)	1.15(0.10,13.23)	0.37(0.07,2.05)	1.06(0.09,12.58)	3.27(0.57,18.80)	2.99(0.22,39.63)	0.86(0.07,10.62)	0.29(0.05,1.67)	1.26(0.10,15.38)	3.77(0.65,21.96)					
ASV_233	Proteobacteria;D_2__Deltaproteobacteria;D_3__Desulfovibrionales;D_4__Desulfovibrionaceae;D_5__Desulfovibrio;D_6__Desulfovibrio desulfuricans;D_7__;D_8__;D_9__;D_10__;D_11__;D_12__;D_13__;D_14__	9301026755.11(0.00,Inf)	1875011918.01(0.00,Inf)	0.20(0.01,5.46)	0.00(0.00,Inf)	0.70(0.02,20.18)										
ASV_235	Firmicutes;D_2__Clostridia;D_3__Clostridiales;D_4__Christensenellaceae;D_5__Christensenellaceae R-7 group;D_6__uncultured prokaryote;D_7__;D_8__;D_9__;D_10__;D_11__;D_12__;D_13__;D_14__	2.93(0.61,14.02)	2.30(0.53,9.98)	0.79(0.28,2.22)	0.43(0.10,1.92)	1.27(0.44,3.68)	2.68(0.56,12.85)	1.89(0.42,8.58)	0.70(0.24,2.05)	0.53(0.12,2.37)	1.41(0.48,4.11)					
ASV_237	Firmicutes;D_2__Clostridia;D_3__Clostridiales;D_4__Lachnospiraceae;D_5__Lachnospira	0.69(0.08,6.09)	0.54(0.07,4.16)	0.78(0.19,3.32)	1.99(0.25,15.67)	1.37(0.31,6.02)	0.90(0.11,7.37)	2.26(0.30,17.19)	2.51(0.60,10.52)	1.08(0.14,8.08)	0.97(0.23,4.09)					
ASV_238	Firmicutes;D_2__Clostridia;D_3__Clostridiales;D_4__Ruminococcaceae	1.56(0.07,35.66)	0.06(0.00,1.47)	0.04(0.00,0.44);p=0.00883	2.17(0.11,43.76)	3.39(0.39,29.23)	7.26(0.28,187.94)	0.02(0.00,0.90);p=0.04395	0.00(0.00,0.05);p=0.00006;q=0.04623	3.01(0.11,82.95)	21.81(2.60,182.67);p=0.00448		↓	↓*		↑
ASV_240	Firmicutes;D_2__Clostridia;D_3__Clostridiales;D_4__Christensenellaceae;D_5__Christensenellaceae R-7 group;D_6__uncultured bacterium;D_7__;D_8__;D_9__;D_10__;D_11__;D_12__;D_13__;D_14__	6727775545.57(0.00,Inf)	3449927259.97(0.00,Inf)	0.51(0.06,4.73)	0.00(0.00,Inf)	1.09(0.11,10.42)	11856721578790066.00(0.00,Inf)	9774400083212978.00(0.00,Inf)	0.82(0.08,8.33)	0.00(0.00,Inf)	0.48(0.05,4.70)					
ASV_241	Firmicutes;D_2__Clostridia;D_3__Clostridiales;D_4__Ruminococcaceae;D_5__uncultured	0.60(0.01,29.48)	0.43(0.01,16.54)	0.72(0.05,10.09)	0.55(0.01,21.07)	0.33(0.02,4.69)										
ASV_242	Firmicutes;D_2__Bacilli;D_3__Bacillales;D_4__Staphylococcaceae;D_5__Staphylococcus	1.41(0.11,18.28)	1.05(0.09,11.54)	0.74(0.14,4.02)	0.68(0.06,7.68)	0.96(0.17,5.39)	1.50(0.12,19.27)	0.96(0.08,11.42)	0.64(0.11,3.66)	0.75(0.06,8.75)	1.12(0.20,6.39)					
ASV_245	Firmicutes;D_2__Clostridia;D_3__Clostridiales;D_4__Peptococcaceae;D_5__uncultured;D_6__metagenome;D_7__;D_8__;D_9__;D_10__;D_11__;D_12__;D_13__;D_14__	0.22(0.01,8.63)	0.93(0.04,23.62)	4.18(0.31,55.71)	1.05(0.04,27.74)	0.23(0.02,3.26)	0.37(0.01,11.58)	0.93(0.04,22.65)	2.55(0.18,36.50)	0.37(0.02,8.20)	0.14(0.01,1.75)					
ASV_246	Firmicutes;D_2__Clostridia;D_3__Clostridiales;D_4__Lachnospiraceae	1.40(0.03,60.35)	0.96(0.03,33.50)	0.69(0.06,8.22)	1.64(0.04,62.53)	2.29(0.17,31.03)	2.49(0.07,89.33)	0.58(0.02,21.22)	0.23(0.02,3.00)	1.50(0.04,52.54)	3.73(0.30,46.08)					

ASV_249	Firmicutes;D_2__Clostridia;D_3__Clostridiales;D_4__Lachnospiraceae;D_5__Lachnoclostridium	0.14(0.02, 0.91);p=0.03994	0.57(0.10, 3.25)	4.11(1.15, 14.65);p=0.02932	5.30(0.90, 31.12)	0.74(0.20, 2.74)	0.14(0.02, 0.91);p=0.0389	0.74(0.13, 4.30)	5.19(1.41,19.08);p=0.01314	3.80(0.65, 22.09)	0.54(0.15,2.01)	↓		↑		
ASV_252	Firmicutes;D_2__Erysipelotrichia;D_3__Erysipelotrichales;D_4__Erysipelotrichaceae;D_5__Erysipelatoclostridium	0.41(0.04, 4.28)	2.05(0.23, 18.43)	5.04(1.05, 24.08);p=0.04266	4.59(0.49, 42.89)	1.87(0.37, 9.41)										
ASV_253	Firmicutes;D_2__Clostridia;D_3__Clostridiales;D_4__Family XIII;D_5__Mogibacterium	1.69(0.10, 28.52)	0.51(0.03, 7.59)	0.30(0.05, 2.03)	0.25(0.02, 3.56)	0.42(0.06, 2.69)										
ASV_254	Firmicutes;D_2__Clostridia;D_3__Clostridiales;D_4__Lachnospiraceae	0.47(0.01, 16.05)	0.22(0.01, 5.92)	0.46(0.04, 4.82)	2.60(0.09, 73.77)	1.22(0.11, 13.47)	0.79(0.03, 23.28)	0.23(0.01, 5.93)	0.28(0.03,2.88)	1.81(0.07, 46.52)	1.44(0.14,14.60)					
ASV_255	Actinobacteria;D_2__Actinobacteria;D_3__Actinomycetales;D_4__Actinomycetaceae;D_5__Actinomyces	1.20(0.27, 5.24)	1.67(0.42, 6.62)	1.40(0.53, 3.66)	0.41(0.10, 1.64)	0.49(0.18, 1.31)	1.30(0.31, 5.51)	1.47(0.36, 5.90)	1.13(0.43,2.97)	0.40(0.10, 1.58)	0.52(0.20,1.35)					
ASV_256	Firmicutes;D_2__Clostridia;D_3__Clostridiales;D_4__Family XIII;D_5__Family XIII AD3011 group;D_6__uncultured bacterium;D_7__;D_8__;D_9__;D_10__;D_11__;D_12__;D_13__;D_14__	1.18(0.07, 19.67)	0.39(0.03, 5.43)	0.33(0.05, 2.13)	0.97(0.07, 13.85)	1.14(0.17, 7.69)										
ASV_258	Firmicutes;D_2__Clostridia;D_3__Clostridiales;D_4__Defluviitaleaceae;D_5__Defluviitaleaceae UCG-011;D_6__uncultured bacterium;D_7__;D_8__;D_9__;D_10__;D_11__;D_12__;D_13__;D_14__	2.54(0.17, 36.87)	0.99(0.08, 12.31)	0.39(0.07, 2.25)	0.41(0.03, 5.20)	1.04(0.18, 6.18)	1.55(0.11, 21.31)	1.00(0.08, 12.73)	0.65(0.11,3.81)	0.51(0.04, 6.30)	0.79(0.13,4.61)					
ASV_259	Patescibacteria;D_2__Saccharimonadia;D_3__Saccharimonadales;D_4__Saccharimonadaceae	1.30(0.15, 11.35)	0.28(0.04, 2.15)	0.21(0.05, 0.92);p=0.03792	0.87(0.11, 6.81)	1.14(0.26, 4.93)	1.03(0.12, 8.60)	0.20(0.03, 1.62)	0.20(0.04,0.87);p=0.03168	1.23(0.16, 9.48)	1.27(0.30,5.43)			↓		
ASV_260	Actinobacteria;D_2__Coriobacteriia;D_3__Coriobacteriales;D_4__Eggerthellaceae;D_5__Enterorhabdus;D_6__uncultured bacterium;D_7__;D_8__;D_9__;D_10__;D_11__;D_12__;D_13__;D_14__	0.57(0.03, 12.73)	0.11(0.01, 2.08)	0.19(0.02, 1.65)	0.53(0.03, 9.86)	0.30(0.04, 2.48)										
ASV_262	Firmicutes;D_2__Clostridia;D_3__Clostridiales;D_4__Defluviitaleaceae;D_5__Defluviitaleaceae UCG-011;D_6__uncultured bacterium;D_7__;D_8__;D_9__;D_10__;D_11__;D_12__;D_13__;D_14__	1.12(0.00, Inf)	8587452856039.46(0.00,Inf)	7691578905488.61(0.00,Inf)	0.00(0.00, Inf)	0.00(0.00, Inf)										
ASV_264	Firmicutes;D_2__Clostridia;D_3__Clostridiales;D_4__Ruminococcaceae;D_5__Ruminiclostridium 5;D_6__uncultured bacterium;D_7__;D_8__;D_9__;D_10__;D_11__;D_12__;D_13__;D_14__	1.54(0.19, 12.50)	1.06(0.15, 7.60)	0.69(0.17, 2.73)	0.50(0.07, 3.59)	0.76(0.19, 3.08)										
ASV_265	Firmicutes;D_2__Clostridia;D_3__Clostridiales;D_4__Lachnospiraceae;D_5__[Ruminococcus] torques group	24.12(0.53,1100.42)	3.26(0.09, 120.92)	0.14(0.01, 1.53)	0.27(0.01, 10.24)	6.41(0.53, 76.80)										
ASV_268	Proteobacteria;D_2__Gammaproteobacteria;D_3__Betaproteobacteriales;D_4__Burkholderiaceae;D_5__Parasutterella	1.31(0.15, 11.23)	1.48(0.20, 11.04)	1.13(0.27, 4.67)	1.14(0.15, 8.68)	1.49(0.35, 6.41)	1.53(0.18, 12.84)	2.46(0.32, 19.20)	1.61(0.38,6.88)	0.66(0.09, 5.12)	1.02(0.24,4.36)					
ASV_270	Bacteroidetes;D_2__Bacteroidia;D_3__Bacteroidales;D_4__Bacteroidaceae;D_5__Bacteroides;D_6__Bacteroides uniformis;D_7__;D_8__;D_9__;D_10__;D_11__;D_12__;D_13__;D_14__	0.53(0.04, 7.23)	0.47(0.04, 5.39)	0.88(0.16, 4.95)	2.56(0.22, 30.24)	1.37(0.23, 8.01)										
ASV_274	Firmicutes;D_2__Clostridia;D_3__Clostridiales;D_4__Christensenellaceae;D_5__Christensenellaceae R-7 group	2.28(0.13, 40.90)	7.13(0.48, 106.24)	3.12(0.46, 21.10)	0.07(0.00, 1.15)	0.17(0.02, 1.21)	2.08(0.12, 37.05)	7.36(0.46, 118.92)	3.55(0.50,25.33)	0.07(0.00, 1.19)	0.16(0.02,1.11)					

ASV_277	Firmicutes;D_2__Clostridia;D_3__Clostridiales;D_4__Ruminococcaceae;D_5__Faecalibacterium;D_6__metagenome;D_7__;D_8__;D_9__;D_10__;D_11__;D_12__;D_13__;D_14__	15.39(1.23,192.75);p=0.03405	19.65(1.84,209.68);p=0.01368	1.28(0.24,6.79)	0.04(0.00,0.41);p=0.00717	0.57(0.10,3.18)	31.07(2.49,388.08);p=0.00765	32.26(2.81,369.78);p=0.00525	1.04(0.19,5.77)	0.02(0.00,0.24);p=0.0018	0.65(0.12,3.64)	↑	↑		↓	
ASV_278	Bacteroidetes;D_2__Bacteroidia;D_3__Bacteroidales;D_4__Bacteroidaceae;D_5__Bacteroides;D_6__Bacteroides stercoris ATCC 43183;D_7__;D_8__;D_9__;D_10__;D_11__;D_12__;D_13__;D_14__	0.27(0.01,7.69)	0.64(0.03,14.76)	2.38(0.26,22.04)	0.99(0.04,23.98)	0.26(0.03,2.59)	0.17(0.01,4.95)	0.29(0.01,7.33)	1.66(0.17,16.30)	1.29(0.05,31.99)	0.22(0.02,2.22)					
ASV_281	Firmicutes;D_2__Clostridia;D_3__Clostridiales;D_4__Lachnospiraceae;D_5__Lachnospiraceae ND3007 group;D_6__metagenome;D_7__;D_8__;D_9__;D_10__;D_11__;D_12__;D_13__;D_14__	0.43(0.09,2.01)	1.37(0.32,5.79)	3.19(1.15,8.84);p=0.02606	0.79(0.18,3.40)	0.34(0.12,0.97);p=0.04319	0.47(0.10,2.19)	1.49(0.34,6.62)	3.19(1.11,9.14);p=0.0306	0.73(0.17,3.22)	0.34(0.12,0.99);p=0.04683			↑		↓
ASV_283	Firmicutes;D_2__Clostridia;D_3__Clostridiales;D_4__Ruminococcaceae;D_5__Ruminococcaceae UCG-002	1.03(0.13,8.47)	0.90(0.13,6.43)	0.87(0.22,3.50)	0.52(0.07,3.79)	0.53(0.13,2.23)	1.72(0.21,13.86)	0.70(0.09,5.24)	0.41(0.10,1.69)	0.40(0.05,2.98)	0.69(0.17,2.89)					
ASV_287	Bacteroidetes;D_2__Bacteroidia;D_3__Bacteroidales;D_4__Tannerellaceae;D_5__Parabacteroides;D_6__Parabacteroides distasonis;D_7__;D_8__;D_9__;D_10__;D_11__;D_12__;D_13__;D_14__	1.56(0.08,30.60)	7.09(0.44,114.78)	4.53(0.63,32.50)	0.28(0.02,4.70)	0.44(0.06,3.30)										
ASV_288	Firmicutes;D_2__Clostridia;D_3__Clostridiales;D_4__Lachnospiraceae;D_5__[Eubacterium] hallii group	1.00(0.36,2.79)	1.52(0.58,3.97)	1.52(0.77,2.99)	0.55(0.21,1.46)	0.56(0.28,1.11)	1.03(0.37,2.85)	1.74(0.65,4.66)	1.69(0.85,3.40)	0.49(0.19,1.31)	0.51(0.25,1.02)					
ASV_289	Bacteroidetes;D_2__Bacteroidia;D_3__Bacteroidales;D_4__Bacteroidaceae;D_5__Bacteroides	1.63(0.10,26.06)	3.59(0.27,48.11)	2.20(0.35,13.84)	1.12(0.08,15.46)	1.82(0.28,11.93)	1.66(0.11,25.60)	6.53(0.47,91.64)	3.93(0.61,25.46)	0.71(0.05,9.82)	1.18(0.18,7.67)					
ASV_291	Firmicutes;D_2__Clostridia;D_3__Clostridiales;D_4__Lachnospiraceae;D_5__Lachnospira	8.18(1.08,62.11);p=0.04207	12.11(1.81,80.77);p=0.01001	1.48(0.39,5.66)	0.07(0.01,0.47);p=0.00629	0.56(0.14,2.22)	7.66(1.01,58.31);p=0.04926	10.37(1.46,73.63);p=0.01931	1.35(0.34,5.40)	0.08(0.01,0.58);p=0.0119	0.63(0.16,2.52)	↑	↑		↓	
ASV_292	Firmicutes;D_2__Clostridia;D_3__Clostridiales;D_4__Ruminococcaceae;D_5__Butyricicoccus	0.95(0.22,4.04)	0.78(0.20,3.01)	0.82(0.31,2.13)	0.89(0.23,3.51)	0.85(0.32,2.26)	0.71(0.17,2.98)	0.63(0.16,2.52)	0.89(0.33,2.38)	1.07(0.27,4.25)	0.75(0.28,2.02)					
ASV_293	Bacteroidetes;D_2__Bacteroidia;D_3__Bacteroidales;D_4__Rikenellaceae;D_5__Alistipes	2467963432.72(0.00,Inf)	8748884035.14(0.00,Inf)	3.54(0.33,38.46)	0.00(0.00,Inf)	0.30(0.03,3.49)										
ASV_295	Firmicutes;D_2__Clostridia;D_3__Clostridiales;D_4__Lachnospiraceae;D_5__[Eubacterium] xylanophilum group						0.90(0.02,51.19)	18.00(0.37,881.56)	20.01(1.28,313.80);p=0.03289	0.03(0.00,1.57)	0.03(0.00,0.46);p=0.01224			↑		↓
ASV_296	Firmicutes;D_2__Clostridia;D_3__Clostridiales;D_4__Lachnospiraceae	0.49(0.12,2.00)	1.42(0.38,5.32)	2.92(1.14,7.44);p=0.025	0.92(0.24,3.50)	0.45(0.17,1.16)	0.52(0.13,2.15)	1.58(0.41,6.18)	3.02(1.15,7.92);p=0.02442	0.92(0.24,3.57)	0.48(0.18,1.27)			↑		
ASV_297	Bacteroidetes;D_2__Bacteroidia;D_3__Bacteroidales;D_4__Tannerellaceae;D_5__Parabacteroides;D_6__Parabacteroides distasonis;D_7__;D_8__;D_9__;D_10__;D_11__;D_12__;D_13__;D_14__	0.81(0.02,34.13)	2.46(0.07,81.57)	3.04(0.25,36.17)	0.29(0.01,9.88)	0.23(0.02,2.93)	1.06(0.03,43.23)	16.08(0.45,576.42)	15.16(1.21,190.54);p=0.03528	0.21(0.01,7.43)	0.22(0.02,2.84)			↑		
ASV_298	Firmicutes;D_2__Clostridia;D_3__Clostridiales;D_4__Ruminococcaceae;D_5__Oscillibacter;D_6__uncultured organism;D_7__;D_8__;D_9__;D_10__;D_11__;D_12__;D_13__;D_14__	0.45(0.14,1.40)	0.50(0.17,1.46)	1.12(0.53,2.38)	2.17(0.74,6.36)	0.97(0.45,2.11)	0.46(0.15,1.43)	0.53(0.18,1.60)	1.17(0.53,2.54)	2.12(0.71,6.32)	0.97(0.44,2.12)					
ASV_299	Firmicutes;D_2__Clostridia;D_3__Clostridiales;D_4__Ruminococcaceae;D_5__Ruminococcaceae UCG-005	2.97(0.12,74.64)	3.88(0.19,79.53)	1.31(0.15,11.06)	0.15(0.01,3.14)	0.44(0.05,3.90)										

ASV_301	Firmicutes;D_2__Clostridia;D_3__Clostridiales;D_4__Lachnospiraceae;D_5__Lachnospiraceae UCG-008;D_6__uncultured organism;D_7__;D_8__;D_9__;D_10__;D_11__;D_12__;D_13__;D_14__	0.63(0.10,3.96)	1.05(0.19,5.83)	1.66(0.49,5.61)	0.63(0.11,3.54)	0.40(0.11,1.38)	0.65(0.11,4.04)	1.34(0.23,7.78)	2.06(0.59,7.18)	0.55(0.10,3.13)	0.36(0.10,1.24)					
ASV_302	Firmicutes;D_2__Clostridia;D_3__Clostridiales;D_4__Lachnospiraceae;D_5__uncultured	0.48(0.09,2.53)	1.71(0.36,8.04)	3.55(1.18,10.69);p=0.02404	0.54(0.11,2.58)	0.26(0.08,0.80);p=0.01903	0.45(0.09,2.36)	1.72(0.35,8.48)	3.83(1.23,11.93);p=0.02053	0.55(0.11,2.70)	0.25(0.08,0.77);p=0.0162			↑		↓
ASV_306	Firmicutes;D_2__Clostridia;D_3__Clostridiales;D_4__Lachnospiraceae;D_5__Lachnospiraceae UCG-001	7.13(0.44,115.62)	8.56(0.63,116.40)	1.20(0.19,7.54)	0.15(0.01,2.07)	1.05(0.16,6.90)										
ASV_309	Firmicutes;D_2__Clostridia;D_3__Clostridiales;D_4__Lachnospiraceae;D_5__Lachnoclostridium;D_6__uncultured Firmicutes bacterium;D_7__;D_8__;D_9__;D_10__;D_11__;D_12__;D_13__;D_14__	0.59(0.11,3.06)	1.67(0.36,7.83)	2.84(0.95,8.47)	0.99(0.21,4.73)	0.58(0.19,1.79)										
ASV_313	Firmicutes;D_2__Clostridia;D_3__Clostridiales;D_4__Ruminococcaceae;D_5__Ruminiclostridium 9	1.84(0.18,19.13)	3.37(0.38,30.14)	1.83(0.39,8.56)	0.20(0.02,1.80)	0.36(0.07,1.75)	2.41(0.23,24.96)	5.47(0.57,52.31)	2.27(0.47,11.07)	0.12(0.01,1.16)	0.30(0.06,1.45)					
ASV_314	Firmicutes;D_2__Clostridia;D_3__Clostridiales;D_4__Lachnospiraceae;D_5__Lachnospiraceae UCG-004	6.14(0.79,47.43)	9.48(1.39,64.53);p=0.02152	1.55(0.41,5.87)	0.04(0.01,0.31);p=0.00169	0.27(0.07,1.08)	6.07(0.79,46.87)	11.26(1.56,81.22);p=0.01633	1.85(0.47,7.32)	0.04(0.01,0.30);p=0.00151	0.25(0.06,1.00)		↑		↓	
ASV_315	Firmicutes;D_2__Clostridia;D_3__Clostridiales;D_4__Lachnospiraceae;D_5__Lachnospiraceae UCG-004	0.22(0.04,1.09)	0.86(0.19,3.81)	3.90(1.35,11.26);p=0.01194	1.52(0.34,6.87)	0.33(0.11,0.99);p=0.04833	0.25(0.05,1.22)	1.20(0.26,5.53)	4.79(1.61,14.24);p=0.00483	1.06(0.23,4.87)	0.27(0.09,0.79);p=0.01723			↑		↓
ASV_317	Firmicutes;D_2__Clostridia;D_3__Clostridiales;D_4__Ruminococcaceae;D_5__[Eubacterium] coprostanoligenes group						5.91(0.12,303.18)	14.83(0.33,674.43)	2.51(0.19,33.83)	0.03(0.00,1.43)	0.19(0.01,2.57)					
ASV_318	Firmicutes;D_2__Clostridia;D_3__Clostridiales;D_4__Lachnospiraceae	0.18(0.04,0.96);p=0.04463	0.47(0.10,2.22)	2.57(0.86,7.71)	2.32(0.48,11.08)	0.43(0.14,1.31)	0.29(0.06,1.50)	0.74(0.15,3.58)	2.53(0.82,7.77)	1.85(0.38,8.94)	0.54(0.18,1.66)					
ASV_322	Firmicutes;D_2__Clostridia;D_3__Clostridiales;D_4__Ruminococcaceae;D_5__Ruminococcaceae UCG-005;D_6__uncultured organism;D_7__;D_8__;D_9__;D_10__;D_11__;D_12__;D_13__;D_14__	1.24(0.11,14.32)	4.88(0.50,47.91)	3.93(0.78,19.67)	0.09(0.01,0.89);p=0.03971	0.11(0.02,0.57);p=0.00864	1.15(0.10,12.83)	5.43(0.53,55.36)	4.73(0.92,24.25)	0.10(0.01,1.01)	0.12(0.02,0.59);p=0.00967					↓
ASV_325	Firmicutes;D_2__Clostridia;D_3__Clostridiales;D_4__Clostridiales vadinBB60 group;D_5__gut metagenome;D_6__;D_7__;D_8__;D_9__;D_10__;D_11__;D_12__;D_13__;D_14__	878768330.45(0.00,Inf)	14827482663.75(0.00,Inf)	16.87(0.98,290.32)	0.00(0.00,Inf)	0.05(0.00,0.96);p=0.04699	7920569230516762.00(0.00,Inf)	26738846328992360.00(0.00,Inf)	3.38(0.19,59.54)	0.00(0.00,Inf)	0.03(0.00,0.55);p=0.01737					↓
ASV_327	Firmicutes;D_2__Clostridia;D_3__Clostridiales;D_4__Lachnospiraceae;D_5__GCA-900066575	1.05(0.15,7.13)	1.46(0.24,8.74)	1.39(0.39,4.93)	0.59(0.10,3.60)	0.62(0.17,2.26)	1.21(0.18,8.20)	1.92(0.30,12.11)	1.58(0.43,5.81)	0.52(0.08,3.22)	0.63(0.17,2.31)					
ASV_329	Firmicutes;D_2__Clostridia;D_3__Clostridiales;D_4__Christensenellaceae;D_5__Christensenellaceae R-7 group;D_6__uncultured organism;D_7__;D_8__;D_9__;D_10__;D_11__;D_12__;D_13__;D_14__	0.53(0.01,32.77)	0.23(0.00,11.04)	0.44(0.03,6.76)	0.63(0.01,31.28)	0.33(0.02,5.45)										
ASV_331	Firmicutes;D_2__Clostridia;D_3__Clostridiales;D_4__Ruminococcaceae;D_5__Ruminococcaceae UCG-005	8.16(0.05,1409.48)	9.27(0.07,1245.96)	1.14(0.06,21.99)	0.85(0.01,137.74)	6.96(0.27,180.72)	9.67(0.08,1158.30)	18.84(0.15,2321.90)	1.95(0.10,37.51)	0.50(0.00,67.14)	4.85(0.21,111.33)					
ASV_334	Firmicutes;D_2__Clostridia;D_3__Clostridiales;D_4__Lachnospiraceae;D_5__uncultured;D_6__uncultured bacterium adhufec382;D_7__;D_8__;D_9__;D_10__;D_11__;D_12__;D_13__;D_14__	1.94(0.35,10.88)	1.59(0.32,8.00)	0.82(0.26,2.55)	0.48(0.09,2.44)	0.92(0.29,2.95)	2.05(0.36,11.53)	1.66(0.31,8.81)	0.81(0.25,2.62)	0.48(0.09,2.52)	0.98(0.30,3.18)					

ASV_335	Firmicutes;D_2__Clostridia;D_3__Clostridiales;D_4__Christensenellaceae;D_5__uncultured;D_6__uncultured bacterium;D_7__;D_8__;D_9__;D_10__;D_11__;D_12__;D_13__;D_14__						0.31(0.00,23.76)	0.37(0.01,24.22)	1.19(0.06,22.96)	1.26(0.02,81.25)	0.39(0.02,7.65)					
ASV_336	Firmicutes;D_2__Clostridia;D_3__Clostridiales;D_4__Family XIII;D_5__Family XIII UCG-001;D_6__uncultured bacterium;D_7__;D_8__;D_9__;D_10__;D_11__;D_12__;D_13__;D_14__						0.00(0.00,Inf)	16.26(0.07,3649.49)	7379768713302509.00(0.00,Inf)	0.07(0.00,13.87)	0.00(0.00,Inf)					
ASV_340	Firmicutes;D_2__Clostridia;D_3__Clostridiales;D_4__Ruminococcaceae;D_5__Flavonifractor;D_6__uncultured bacterium;D_7__;D_8__;D_9__;D_10__;D_11__;D_12__;D_13__;D_14__	2.42(0.28,21.18)	1.25(0.16,9.55)	0.52(0.12,2.16)	0.64(0.08,4.97)	1.54(0.36,6.68)	1.69(0.20,14.50)	0.98(0.12,7.82)	0.58(0.13,2.51)	0.92(0.12,7.28)	1.55(0.36,6.75)					
ASV_344	Firmicutes;D_2__Clostridia;D_3__Clostridiales;D_4__Defluviitaleaceae;D_5__Defluviitaleaceae UCG-011;D_6__uncultured bacterium;D_7__;D_8__;D_9__;D_10__;D_11__;D_12__;D_13__;D_14__	0.05(0.00,2.72)	0.26(0.01,10.46)	5.33(0.34,82.52)	4.66(0.11,194.75)	0.23(0.01,3.79)										
ASV_345	Firmicutes;D_2__Clostridia;D_3__Clostridiales;D_4__Lachnospiraceae;D_5__Lachnospira	0.90(0.09,9.47)	1.53(0.17,13.89)	1.71(0.36,8.13)	0.17(0.02,1.56)	0.15(0.03,0.74);p=0.02004	0.91(0.09,9.63)	1.51(0.15,14.77)	1.67(0.33,8.36)	0.17(0.02,1.65)	0.15(0.03,0.78);p=0.02349					↓
ASV_348	Firmicutes;D_2__Clostridia;D_3__Clostridiales;D_4__Lachnospiraceae;D_5__Moryella;D_6__human gut metagenome;D_7__;D_8__;D_9__;D_10__;D_11__;D_12__;D_13__;D_14__	3.34(0.57,19.44)	3.61(0.69,18.87)	1.08(0.34,3.39)	0.21(0.04,1.12)	0.70(0.22,2.26)	4.23(0.76,23.61)	4.63(0.88,24.49)	1.10(0.35,3.45)	0.20(0.04,1.06)	0.86(0.27,2.71)					
ASV_349	Firmicutes;D_2__Clostridia;D_3__Clostridiales;D_4__Ruminococcaceae;D_5__Flavonifractor	0.88(0.32,2.46)	0.80(0.31,2.09)	0.90(0.46,1.78)	2.17(0.82,5.74)	1.92(0.96,3.85)	0.75(0.27,2.08)	0.76(0.29,2.03)	1.01(0.51,2.02)	2.04(0.77,5.39)	1.54(0.77,3.08)					
ASV_351	Firmicutes;D_2__Clostridia;D_3__Clostridiales;D_4__Ruminococcaceae;D_5__uncultured;D_6__Clostridium phoceensis;D_7__;D_8__;D_9__;D_10__;D_11__;D_12__;D_13__;D_14__	1.12(0.00,Inf)	59441257667.04(0.00,Inf)	53240833080.97(0.00,Inf)	0.00(0.00,Inf)	0.00(0.00,Inf)	1.03(0.00,Inf)	432344962089001.00(0.00,Inf)	419760318569950.00(0.00,Inf)	0.00(0.00,Inf)	0.00(0.00,Inf)					
ASV_353	Firmicutes;D_2__Clostridia;D_3__Clostridiales;D_4__Ruminococcaceae;D_5__Ruminococcaceae UCG-010	5.77(0.49,67.28)	8.32(0.83,83.73)	1.44(0.30,6.97)	0.15(0.01,1.60)	0.89(0.18,4.47)	8.65(0.74,100.62)	10.75(1.00,115.90)	1.24(0.25,6.16)	0.14(0.01,1.45)	1.18(0.24,5.85)					
ASV_354	Firmicutes;D_2__Clostridia;D_3__Clostridiales;D_4__Lachnospiraceae;D_5__Lachnospiraceae FCS020 group	2.54(0.38,17.15)	3.65(0.61,21.92)	1.44(0.43,4.82)	0.41(0.07,2.53)	1.05(0.30,3.64)	2.62(0.39,17.63)	3.79(0.60,23.97)	1.45(0.42,5.05)	0.39(0.06,2.42)	1.01(0.29,3.55)					
ASV_355	Firmicutes;D_2__Clostridia;D_3__Clostridiales;D_4__Ruminococcaceae;D_5__Ruminococcaceae UCG-009;D_6__uncultured bacterium;D_7__;D_8__;D_9__;D_10__;D_11__;D_12__;D_13__;D_14__						0.48(0.01,16.80)	2.07(0.07,58.95)	4.35(0.39,48.95)	0.61(0.02,17.08)	0.29(0.03,3.34)					
ASV_357	Firmicutes;D_2__Clostridia;D_3__Clostridiales;D_4__Ruminococcaceae;D_5__Oscillibacter;D_6__human gut metagenome;D_7__;D_8__;D_9__;D_10__;D_11__;D_12__;D_13__;D_14__	0.38(0.10,1.45)	0.99(0.28,3.44)	2.61(1.07,6.40);p=0.03565	1.15(0.32,4.06)	0.43(0.17,1.09)	0.40(0.10,1.53)	1.04(0.29,3.78)	2.60(1.03,6.55);p=0.0421	1.08(0.30,3.89)	0.43(0.17,1.09)			↑		
ASV_360	Firmicutes;D_2__Clostridia;D_3__Clostridiales;D_4__Ruminococcaceae;D_5__Ruminiclostridium 9;D_6__uncultured organism;D_7__;D_8__;D_9__;D_10__;D_11__;D_12__;D_13__;D_14__	1.45(0.30,6.95)	1.08(0.25,4.68)	0.74(0.26,2.09)	0.49(0.11,2.15)	0.71(0.25,2.04)	1.59(0.33,7.60)	1.21(0.27,5.49)	0.76(0.26,2.21)	0.45(0.10,2.04)	0.72(0.25,2.09)					
ASV_364	Firmicutes;D_2__Clostridia;D_3__Clostridiales;D_4__Lachnospiraceae;D_5__Roseburia;D_6__gut metagenome;D_7__;D_8__;D_9__;D_10__;D_11__;D_12__;D_13__;D_14__	3950859972.94(0.00,Inf)	7247468652.49(0.00,Inf)	1.83(0.10,33.38)	0.00(0.00,Inf)	4.31(0.20,91.81)										
ASV_365	Firmicutes;D_2__Clostridia;D_3__Clostridiales;D_4__Ruminococcaceae;D_5__Intestinimonas	0.37(0.01,14.33)	4.46(0.17,118.97)	11.99(1.01,142.67);p=0.04931	1.37(0.05,39.74)	0.51(0.04,6.76)	0.29(0.01,8.20)	1.09(0.05,24.09)	3.71(0.36,38.29)	2.82(0.12,64.82)	0.83(0.07,9.30)					

ASV_366	Proteobacteria;D_2__Gammaproteobacteria;D_3__Pasteurellales;D_4__Pasteurellaceae;D_5__Haemophilus	1.19(0.22, 6.43)	1.02(0.21, 4.99)	0.86(0.28, 2.65)	1.03(0.21, 5.10)	1.22(0.39, 3.83)	1.18(0.22, 6.34)	1.34(0.26, 6.84)	1.14(0.36,3.61)	0.83(0.17, 4.20)	0.98(0.31,3.10)					
ASV_368	Firmicutes;D_2__Clostridia;D_3__Clostridiales;D_4__Ruminococcaceae;D_5__Ruminococcaceae UCG-013;D_6__gut metagenome;D_7__;D_8__;D_9__;D_10__;D_11__;D_12__;D_13__;D_14__	0.82(0.08, 8.38)	0.96(0.11, 8.45)	1.17(0.25, 5.43)	0.67(0.07, 6.02)	0.55(0.11, 2.67)										
ASV_369	Firmicutes;D_2__Clostridia;D_3__Clostridiales;D_4__Ruminococcaceae;D_5__Ruminococcaceae UCG-003;D_6__uncultured bacterium;D_7__;D_8__;D_9__;D_10__;D_11__;D_12__;D_13__;D_14__	0.78(0.07, 8.35)	2.29(0.25, 20.99)	2.93(0.61, 14.10)	0.30(0.03, 2.86)	0.24(0.05, 1.18)	1.00(0.09, 10.59)	2.91(0.30, 28.43)	2.92(0.58,14.65)	0.21(0.02, 1.98)	0.21(0.04,1.03)					
ASV_371	Firmicutes;D_2__Clostridia;D_3__Clostridiales;D_4__Ruminococcaceae;D_5__uncultured;D_6__Clostridium phoceensis;D_7__;D_8__;D_9__;D_10__;D_11__;D_12__;D_13__;D_14__	0.62(0.15, 2.62)	0.63(0.16, 2.41)	1.01(0.39, 2.63)	2.71(0.69, 10.65)	1.68(0.63, 4.50)	0.59(0.14, 2.50)	0.62(0.16, 2.48)	1.04(0.39,2.79)	2.80(0.71, 11.09)	1.66(0.62,4.46)					
ASV_373	Firmicutes;D_2__Clostridia;D_3__Clostridiales;D_4__Ruminococcaceae;D_5__Oscillospira;D_6__uncultured bacterium;D_7__;D_8__;D_9__;D_10__;D_11__;D_12__;D_13__;D_14__	139191992310.71(0.00,Inf)	130079184334.31(0.00,Inf)	0.93(0.03, 32.40)	0.00(0.00, Inf)	4.91(0.10, 246.39)										
ASV_374	Firmicutes;D_2__Clostridia;D_3__Clostridiales;D_4__Clostridiaceae 1;D_5__Clostridium sensu stricto 1	0.53(0.04, 6.60)	0.46(0.04, 4.95)	0.88(0.16, 4.71)	0.56(0.05, 6.15)	0.29(0.05, 1.63)	1.60(0.13, 19.40)	0.91(0.08, 10.16)	0.57(0.10,3.13)	0.53(0.05, 5.82)	0.85(0.15,4.69)					
ASV_376	Firmicutes;D_2__Clostridia;D_3__Clostridiales;D_4__Clostridiales vadinBB60 group;D_5__gut metagenome;D_6__;D_7__;D_8__;D_9__;D_10__;D_11__;D_12__;D_13__;D_14__	4545755241.45(0.00,Inf)	4184137720.73(0.00,Inf)	0.92(0.04, 21.46)	0.00(0.00, Inf)	1.02(0.04, 25.77)										
ASV_377	Firmicutes;D_2__Clostridia;D_3__Clostridiales;D_4__Ruminococcaceae;D_5__Phocea;D_6__uncultured bacterium;D_7__;D_8__;D_9__;D_10__;D_11__;D_12__;D_13__;D_14__	1.21(0.24, 5.98)	1.53(0.34, 6.82)	1.27(0.44, 3.62)	0.67(0.15, 3.03)	0.80(0.27, 2.36)	1.30(0.26, 6.49)	1.88(0.40, 8.84)	1.44(0.49,4.27)	0.60(0.13, 2.79)	0.78(0.26,2.32)					
ASV_378	Proteobacteria;D_2__Gammaproteobacteria;D_3__Enterobacteriales;D_4__Enterobacteriaceae;D_5__Escherichia-Shigella	0.25(0.04, 1.64)	0.25(0.04, 1.48)	1.01(0.29, 3.53)	2.13(0.36, 12.69)	0.53(0.15, 1.91)	0.07(0.01, 0.44);p=0.00438	0.14(0.02, 0.82);p=0.0287	1.99(0.57,6.89)	5.45(0.95, 31.35)	0.39(0.11,1.34)	↓	↓			
ASV_380	Firmicutes;D_2__Clostridia;D_3__Clostridiales;D_4__Ruminococcaceae;D_5__Oscillibacter;D_6__Oscillibacter sp. PC13;D_7__;D_8__;D_9__;D_10__;D_11__;D_12__;D_13__;D_14__	1497891679.08(0.00,Inf)	7720159821.84(0.00,Inf)	5.15(0.26, 100.33)	0.00(0.00, Inf)	0.15(0.01, 3.16)										
ASV_383	Firmicutes;D_2__Clostridia;D_3__Clostridiales;D_4__Clostridiales vadinBB60 group;D_5__uncultured bacterium;D_6__;D_7__;D_8__;D_9__;D_10__;D_11__;D_12__;D_13__;D_14__						2.16(0.09, 51.54)	1.28(0.06, 27.57)	0.59(0.07,4.93)	0.36(0.02, 7.54)	0.78(0.09,6.41)					
ASV_384	Firmicutes;D_2__Clostridia;D_3__Clostridiales;D_4__Ruminococcaceae;D_5__Ruminococcaceae UCG-005	0.48(0.02, 13.81)	0.74(0.03, 16.43)	1.53(0.16, 14.98)	0.66(0.03, 14.94)	0.32(0.03, 3.17)										
ASV_386	Firmicutes;D_2__Clostridia;D_3__Clostridiales;D_4__Lachnospiraceae	0.71(0.01, 58.62)	1.09(0.02, 65.50)	1.54(0.08, 29.29)	1.16(0.02, 73.76)	0.83(0.04, 17.12)	0.71(0.01, 61.23)	1.15(0.02, 81.22)	1.63(0.08,34.05)	1.21(0.02, 84.77)	0.86(0.04,18.70)					
ASV_388	Firmicutes;D_2__Clostridia;D_3__Clostridiales;D_4__Ruminococcaceae;D_5__Oscillospira	0.76(0.13, 4.33)	1.12(0.22, 5.72)	1.48(0.47, 4.71)	0.97(0.19, 5.04)	0.73(0.22, 2.40)	0.87(0.16, 4.90)	1.45(0.27, 7.62)	1.66(0.51,5.40)	0.77(0.15, 4.04)	0.68(0.21,2.21)					
ASV_392	Actinobacteria;D_2__Coriobacteriia;D_3__Coriobacteriales;D_4__Eggerthellaceae;D_5__Eggerthella	1.13(0.36, 3.57)	0.99(0.34, 2.90)	0.87(0.41, 1.87)	1.21(0.41, 3.59)	1.37(0.63, 2.99)	1.18(0.37, 3.71)	1.06(0.35, 3.22)	0.90(0.41,1.97)	1.04(0.35, 3.14)	1.23(0.56,2.70)					

ASV_393	Firmicutes;D_2__Clostridia;D_3__Clostridiales;D_4__Ruminococcaceae;D_5__Ruminococcaceae UCG-005;D_6__uncultured organism;D_7__;D_8__;D_9__;D_10__;D_11__;D_12__;D_13__;D_14__	0.28(0.02,5.05)	0.55(0.04,8.14)	1.96(0.29,13.43)	0.89(0.06,13.43)	0.25(0.04,1.79)	0.49(0.03,8.38)	1.11(0.07,17.12)	2.27(0.32,16.09)	0.61(0.04,9.18)	0.30(0.04,2.10)					
ASV_397	Firmicutes;D_2__Clostridia;D_3__Clostridiales;D_4__Family XIII;D_5__Family XIII AD3011 group;D_6__uncultured organism;D_7__;D_8__;D_9__;D_10__;D_11__;D_12__;D_13__;D_14__	1.60(0.16,15.58)	3.31(0.40,27.70)	2.07(0.47,9.05)	0.31(0.04,2.62)	0.49(0.11,2.22)	2.00(0.22,18.22)	5.50(0.65,46.34)	2.75(0.62,12.25)	0.24(0.03,1.99)	0.48(0.11,2.12)					
ASV_401	Firmicutes;D_2__Clostridia;D_3__Clostridiales;D_4__Ruminococcaceae;D_5__uncultured;D_6__uncultured Ruminococcus sp.;D_7__;D_8__;D_9__;D_10__;D_11__;D_12__;D_13__;D_14__	1.36(0.22,8.66)	1.60(0.28,9.00)	1.17(0.35,3.96)	0.59(0.10,3.39)	0.80(0.23,2.80)	1.76(0.28,10.99)	1.55(0.26,9.07)	0.88(0.25,3.04)	0.64(0.11,3.69)	1.12(0.32,3.89)					
ASV_402	Firmicutes;D_2__Clostridia;D_3__Clostridiales;D_4__Ruminococcaceae;D_5__Ruminiclostridium 9;D_6__uncultured bacterium;D_7__;D_8__;D_9__;D_10__;D_11__;D_12__;D_13__;D_14__	1.12(0.00,Inf)	14578008617.89(0.00,Inf)	13057367044.97(0.00,Inf)	0.00(0.00,Inf)	0.00(0.00,Inf)										
ASV_405	Actinobacteria;D_2__Actinobacteria;D_3__Actinomycetales;D_4__Actinomycetaceae;D_5__Actinomyces	0.33(0.04,2.99)	0.17(0.02,1.35)	0.51(0.11,2.47)	0.66(0.09,5.14)	0.22(0.05,1.00)	0.32(0.04,2.80)	0.18(0.02,1.46)	0.55(0.11,2.73)	0.56(0.07,4.22)	0.18(0.04,0.82);p=0.027					↓
ASV_406	Firmicutes;D_2__Clostridia;D_3__Clostridiales;D_4__Ruminococcaceae	1.12(0.00,Inf)	6817882039.35(0.00,Inf)	6106704007.72(0.00,Inf)	0.00(0.00,Inf)	0.00(0.00,Inf)										
ASV_410	Firmicutes;D_2__Clostridia;D_3__Clostridiales;D_4__Lachnospiraceae;D_5__Anaerosporobacter;D_6__uncultured organism;D_7__;D_8__;D_9__;D_10__;D_11__;D_12__;D_13__;D_14__	938125661.13(0.00,Inf)	14587155204.97(0.00,Inf)	15.55(0.94,256.56)	0.00(0.00,Inf)	0.02(0.00,0.37);p=0.00854	13014424164695352.00(0.00,Inf)	730498967526387968.00(0.00,Inf)	56.13(2.54,1241.43);p=0.01079	0.00(0.00,Inf)	0.00(0.00,0.03);p=0.00003;q=0.03732			↑		↓*
ASV_411	Firmicutes;D_2__Clostridia;D_3__Clostridiales;D_4__Ruminococcaceae;D_5__Ruminiclostridium 9;D_6__uncultured bacterium;D_7__;D_8__;D_9__;D_10__;D_11__;D_12__;D_13__;D_14__	0.47(0.00,47.60)	0.66(0.01,49.07)	1.40(0.06,30.45)	1.55(0.02,121.13)	0.73(0.03,17.20)										
ASV_413	Firmicutes;D_2__Clostridia;D_3__Clostridiales;D_4__Lachnospiraceae;D_5__Lachnospiraceae UCG-010;D_6__uncultured bacterium;D_7__;D_8__;D_9__;D_10__;D_11__;D_12__;D_13__;D_14__	0.24(0.03,2.15)	0.34(0.04,2.56)	1.40(0.32,6.13)	2.23(0.29,17.31)	0.54(0.12,2.45)	0.18(0.02,1.51)	0.43(0.06,3.33)	2.43(0.53,11.15)	2.75(0.36,21.13)	0.49(0.11,2.23)					
ASV_414	Firmicutes;D_2__Clostridia;D_3__Clostridiales;D_4__Ruminococcaceae;D_5__GCA-900066225;D_6__uncultured bacterium;D_7__;D_8__;D_9__;D_10__;D_11__;D_12__;D_13__;D_14__	0.10(0.00,4.77)	0.37(0.01,12.60)	3.62(0.26,49.81)	5.79(0.16,207.62)	0.60(0.04,8.96)	0.12(0.00,5.09)	0.96(0.03,31.24)	7.91(0.44,142.39)	1.38(0.04,43.53)	0.17(0.01,2.87)					
ASV_415	Firmicutes;D_2__Clostridia;D_3__Clostridiales;D_4__Ruminococcaceae;D_5__Pseudoflavonifractor;D_6__uncultured bacterium;D_7__;D_8__;D_9__;D_10__;D_11__;D_12__;D_13__;D_14__	0.91(0.05,17.69)	0.81(0.05,13.13)	0.90(0.12,6.52)	2.22(0.13,37.75)	2.01(0.26,15.70)	0.91(0.05,17.77)	0.84(0.05,14.90)	0.93(0.12,7.17)	2.32(0.13,41.09)	2.10(0.26,16.80)					
ASV_418	Firmicutes;D_2__Clostridia;D_3__Clostridiales;D_4__Christensenellaceae;D_5__uncultured;D_6__uncultured bacterium;D_7__;D_8__;D_9__;D_10__;D_11__;D_12__;D_13__;D_14__	1.01(0.00,304.39)	3.20(0.02,654.76)	3.17(0.07,136.32)	0.60(0.00,132.90)	0.61(0.01,28.94)										
ASV_419	Actinobacteria;D_2__Coriobacteriia;D_3__Coriobacteriales;D_4__Eggerthellaceae;D_5__uncultured;D_6__uncultured Coriobacteriales bacterium;D_7__;D_8__;D_9__;D_10__;D_11__;D_12__;D_13__;D_14__	0.00(0.00,Inf)	0.01(0.00,2.36)	1655738696.04(0.00,Inf)	12.23(0.08,1857.16)	0.00(0.00,Inf)										
ASV_420	Firmicutes;D_2__Clostridia;D_3__Clostridiales;D_4__Ruminococcaceae;D_5__Fournierella;D_6__uncultured organism;D_7__;D_8__;D_9__;D_10__;D_11__;D_12__;D_13__;D_14__	0.64(0.08,4.89)	0.43(0.06,2.89)	0.67(0.17,2.71)	0.57(0.09,3.79)	0.36(0.09,1.45)	0.67(0.09,5.07)	0.44(0.06,3.12)	0.65(0.15,2.78)	0.45(0.07,3.07)	0.30(0.08,1.23)					
ASV_421	Firmicutes;D_2__Clostridia;D_3__Clostridiales;D_4__Lachnospiraceae;D_5__Agathobacter	280117481473.81(0.00,Inf)	137027874790.44(0.00,Inf)	0.49(0.02,13.82)	0.00(0.00,Inf)	0.14(0.01,2.39)	323889651511684.00(0.00,Inf)	90600337301864.91(0.00,Inf)	0.28(0.01,7.72)	0.00(0.00,Inf)	0.27(0.02,3.84)					

ASV_422	Firmicutes;D_2__Clostridia;D_3__Clostridiales;D_4__Ruminococcaceae;D_5__Ruminococcaceae UCG-007	0.00(0.00,Inf)	0.10(0.00,8.03)	6417151177.87(0.00,Inf)	6.40(0.08,524.00)	0.00(0.00,Inf)										
ASV_423	Firmicutes;D_2__Clostridia;D_3__Clostridiales;D_4__Lachnospiraceae;D_5__Lachnoclostridium	0.30(0.04,1.98)	0.30(0.05,1.72)	0.99(0.27,3.63)	2.15(0.36,12.67)	0.64(0.17,2.39)	0.37(0.06,2.45)	0.40(0.07,2.45)	1.08(0.29,4.08)	1.88(0.31,11.32)	0.70(0.19,2.62)					
ASV_424	Firmicutes;D_2__Clostridia;D_3__Clostridiales;D_4__Lachnospiraceae	11.87(0.61,230.56)	27.08(1.62,451.74);p=0.02159	2.28(0.40,12.96)	0.05(0.00,0.79);p=0.03383	0.55(0.09,3.25)	7.44(0.43,128.07)	15.45(0.97,245.15)	2.08(0.35,12.31)	0.06(0.00,1.00);p=0.04979	0.48(0.08,2.83)				↓	
ASV_426	Firmicutes;D_2__Clostridia;D_3__Clostridiales;D_4__Lachnospiraceae;D_5__[Ruminococcus] torques group	0.57(0.06,5.10)	0.71(0.09,5.56)	1.26(0.29,5.45)	2.55(0.32,20.57)	1.44(0.32,6.53)	0.47(0.05,4.15)	0.53(0.06,4.36)	1.14(0.25,5.12)	2.84(0.35,23.16)	1.32(0.29,6.00)					
ASV_428	Firmicutes;D_2__Clostridia;D_3__Clostridiales;D_4__Lachnospiraceae;D_5__Lachnoclostridium	0.05(0.00,4.26)	0.07(0.00,4.09)	1.28(0.07,23.60)	8.90(0.15,543.80)	0.49(0.02,9.59)										
ASV_429	Firmicutes;D_2__Clostridia;D_3__Clostridiales;D_4__Ruminococcaceae;D_5__uncultured;D_6__uncultured Clostridium sp.;D_7__;D_8__;D_9__;D_10__;D_11__;D_12__;D_13__;D_14__	0.47(0.02,13.87)	0.91(0.04,20.90)	1.93(0.20,18.74)	0.61(0.03,14.42)	0.29(0.03,2.89)	0.44(0.01,13.28)	1.30(0.05,33.37)	2.96(0.27,32.84)	0.33(0.01,8.15)	0.15(0.01,1.57)					
ASV_430	Firmicutes;D_2__Clostridia;D_3__Clostridiales;D_4__Ruminococcaceae;D_5__Ruminococcaceae UCG-013;D_6__uncultured Clostridiaceae bacterium;D_7__;D_8__;D_9__;D_10__;D_11__;D_12__;D_13__;D_14__						0.00(0.00,Inf)	1.03(0.02,45.26)	10184717101628638.00(0.00,Inf)	0.52(0.01,21.57)	0.00(0.00,Inf)					
ASV_431	Firmicutes;D_2__Clostridia;D_3__Clostridiales;D_4__Ruminococcaceae	0.41(0.06,3.05)	0.43(0.07,2.80)	1.05(0.27,3.99)	1.61(0.24,10.71)	0.66(0.17,2.59)	0.49(0.07,3.58)	0.37(0.05,2.54)	0.76(0.19,2.99)	1.91(0.28,12.90)	0.93(0.24,3.67)					
ASV_432	Firmicutes;D_2__Erysipelotrichia;D_3__Erysipelotrichales;D_4__Erysipelotrichaceae;D_5__Holdemania	0.23(0.02,2.72)	0.36(0.04,3.67)	1.60(0.30,8.53)	2.90(0.28,30.38)	0.66(0.12,3.65)	0.16(0.01,1.82)	0.20(0.02,2.16)	1.31(0.24,7.26)	3.68(0.35,38.38)	0.57(0.10,3.17)					
ASV_437	Firmicutes;D_2__Clostridia;D_3__Clostridiales;D_4__Ruminococcaceae;D_5__Candidatus Soleaferrea;D_6__uncultured bacterium;D_7__;D_8__;D_9__;D_10__;D_11__;D_12__;D_13__;D_14__	0.44(0.04,5.28)	0.84(0.09,8.25)	1.92(0.35,10.52)	1.38(0.14,14.09)	0.60(0.11,3.47)	0.26(0.02,3.17)	0.60(0.06,6.12)	2.27(0.39,13.34)	2.08(0.20,21.12)	0.55(0.09,3.28)					
ASV_439	Firmicutes;D_2__Clostridia;D_3__Clostridiales;D_4__Peptococcaceae;D_5__uncultured;D_6__metagenome;D_7__;D_8__;D_9__;D_10__;D_11__;D_12__;D_13__;D_14__	225500510629.14(0.00,Inf)	289629232851.30(0.00,Inf)	1.28(0.04,44.80)	0.00(0.00,Inf)	0.47(0.01,17.00)										
ASV_445	Proteobacteria;D_2__Gammaproteobacteria;D_3__Betaproteobacteriales;D_4__Burkholderiaceae;D_5__Sutterella						0.65(0.02,17.82)	4.52(0.19,110.01)	6.92(0.72,66.11)	0.13(0.01,3.08)	0.08(0.01,0.81);p=0.03201					↓
ASV_448	Actinobacteria;D_2__Actinobacteria;D_3__Bifidobacteriales;D_4__Bifidobacteriaceae;D_5__Bifidobacterium	0.35(0.05,2.28)	0.34(0.06,1.93)	0.95(0.28,3.28)	2.15(0.37,12.56)	0.76(0.21,2.69)	0.35(0.06,2.25)	0.40(0.07,2.41)	1.14(0.32,4.04)	1.95(0.33,11.58)	0.69(0.19,2.45)					
ASV_456	Firmicutes;D_2__Clostridia;D_3__Clostridiales;D_4__Peptostreptococcaceae;D_5__Intestinibacter	1.37(0.42,4.47)	0.57(0.19,1.73)	0.42(0.19,0.91);p=0.02825	1.03(0.34,3.15)	1.42(0.64,3.15)	1.31(0.41,4.24)	0.50(0.16,1.56)	0.38(0.17,0.85);p=0.01866	1.16(0.38,3.58)	1.53(0.68,3.41)			↓		
ASV_457	Firmicutes;D_2__Clostridia;D_3__Clostridiales;D_4__Lachnospiraceae;D_5__uncultured	29742687263.58(0.00,Inf)	58580533539.39(0.00,Inf)	1.97(0.40,9.66)	0.00(0.00,Inf)	0.69(0.14,3.57)	8052357500309444.00(0.00,Inf)	30073494621296992.00(0.00,Inf)	3.73(0.71,19.71)	0.00(0.00,Inf)	0.62(0.12,3.27)					

ASV_460	Firmicutes;D_2__Clostridia;D_3__Clostridiales;D_4__Lachnospiraceae	1.80(0.19,17.27)	1.90(0.23,15.77)	1.05(0.24,4.70)	0.45(0.05,3.86)	0.82(0.18,3.77)										
ASV_462	Firmicutes;D_2__Clostridia;D_3__Clostridiales;D_4__Lachnospiraceae;D_5__uncultured;D_6__uncultured bacterium adhufec382;D_7__;D_8__;D_9__;D_10__;D_11__;D_12__;D_13__;D_14__	2.01(0.07,61.77)	34.06(1.44,805.48);p=0.02881	16.95(2.39,120.22);p=0.00463	0.12(0.01,3.07)	0.25(0.03,1.89)	2.52(0.08,74.67)	45.17(1.79,1143.22);p=0.02081	17.93(2.45,131.04);p=0.00444	0.13(0.01,3.18)	0.32(0.04,2.36)		↑	↑		
ASV_464	Firmicutes;D_2__Clostridia;D_3__Clostridiales;D_4__Lachnospiraceae;D_5__Agathobacter						0.53(0.02,15.60)	4.18(0.16,106.66)	7.83(0.72,84.59)	0.47(0.02,11.82)	0.25(0.02,2.69)					
ASV_465	Firmicutes;D_2__Bacilli;D_3__Lactobacillales;D_4__Lactobacillaceae;D_5__Lactobacillus	23.83(0.27,2077.14)	141.93(2.09,9659.34);p=0.02137	5.96(0.38,92.56)	0.06(0.00,4.42)	1.48(0.09,24.70)	221.58(0.75,65116.63)	2481.74(8.30,742152.83);p=0.0072	11.20(0.65,192.18)	0.00(0.00,0.43);p=0.02428	0.34(0.02,5.56)		↑		↓	
ASV_466	Firmicutes;D_2__Clostridia;D_3__Clostridiales;D_4__Lachnospiraceae;D_5__uncultured	1.12(0.00,Inf)	16531056843.47(0.00,Inf)	14806779934.06(0.00,Inf)	0.00(0.00,Inf)	0.00(0.00,Inf)										
ASV_467	Firmicutes;D_2__Bacilli;D_3__Lactobacillales;D_4__Lactobacillaceae;D_5__Lactobacillus	4850675098.08(0.00,Inf)	8448574767.43(0.00,Inf)	1.74(0.15,19.92)	0.00(0.00,Inf)	2.62(0.21,32.42)	16034209472528100.00(0.00,Inf)	34941489290715900.00(0.00,Inf)	2.18(0.19,24.73)	0.00(0.00,Inf)	3.74(0.31,44.82)					
ASV_471	Firmicutes;D_2__Bacilli;D_3__Lactobacillales;D_4__Streptococcaceae;D_5__Streptococcus	1.74(0.22,13.82)	0.92(0.13,6.38)	0.53(0.13,2.08)	0.90(0.13,6.43)	1.57(0.39,6.40)	0.42(0.06,2.99)	0.20(0.03,1.32)	0.47(0.12,1.81)	2.19(0.34,14.28)	0.93(0.24,3.55)					
ASV_472	Firmicutes;D_2__Erysipelotrichia;D_3__Erysipelotrichales;D_4__Erysipelotrichaceae;D_5__Dielma	2.28(0.30,17.36)	3.96(0.59,26.45)	1.73(0.46,6.56)	1.47(0.21,10.17)	3.36(0.85,13.32)	2.80(0.38,20.84)	6.22(0.89,43.36)	2.22(0.58,8.60)	0.56(0.08,3.89)	1.57(0.40,6.15)					
ASV_474	Firmicutes;D_2__Bacilli;D_3__Lactobacillales;D_4__Leuconostocaceae;D_5__Leuconostoc	0.18(0.00,48.08)	1.69(0.01,306.24)	9.28(0.23,379.21)	1.53(0.01,296.88)	0.28(0.01,12.51)										
ASV_475	Firmicutes;D_2__Negativicutes;D_3__Selenomonadales;D_4__Veillonellaceae;D_5__Veillonella	0.60(0.03,11.48)	1.25(0.09,18.42)	2.08(0.28,15.27)	0.26(0.02,3.90)	0.16(0.02,1.18)										
ASV_476	Cyanobacteria;D_2__Oxyphotobacteria;D_3__Chloroplast	0.03(0.00,1.70)	0.05(0.00,2.14)	1.83(0.09,35.63)	27.79(0.58,1332.94)	0.73(0.03,15.53)										
ASV_477	Firmicutes;D_2__Clostridia;D_3__Clostridiales;D_4__Peptostreptococcaceae;D_5__Terrisporobacter;D_6__uncultured bacterium;D_7__;D_8__;D_9__;D_10__;D_11__;D_12__;D_13__;D_14__	6.37(0.96,42.06)	2.08(0.35,12.17)	0.33(0.09,1.14)	0.10(0.02,0.61);p=0.01238	0.65(0.18,2.33)										
ASV_481	Proteobacteria;D_2__Alphaproteobacteria;D_3__Caulobacterales;D_4__Caulobacteraceae;D_5__Brevundimonas	211939718954.77(0.00,Inf)	14354510276.57(0.00,Inf)	0.07(0.01,0.82);p=0.03466	0.00(0.00,Inf)	3.68(0.37,36.23)	7094238560567908.00(0.00,Inf)	558617984858965.00(0.00,Inf)	0.08(0.01,0.89);p=0.04021	0.00(0.00,Inf)	6.91(0.70,67.86)			↓		
ASV_482	Firmicutes;D_2__Negativicutes;D_3__Selenomonadales;D_4__Veillonellaceae;D_5__Megasphaera;D_6__unidentified;D_7__;D_8__;D_9__;D_10__;D_11__;D_12__;D_13__;D_14__	0.00(0.00,Inf)	0.30(0.00,36.10)	3329498167.04(0.00,Inf)	2.13(0.02,265.04)	0.00(0.00,Inf)										
ASV_484	Firmicutes;D_2__Bacilli;D_3__Lactobacillales;D_4__Lactobacillaceae;D_5__Pediococcus	318896157.56(0.00,Inf)	1120008262.93(0.00,Inf)	3.51(0.11,115.28)	0.00(0.00,Inf)	0.02(0.00,0.62);p=0.02631										

ASV_485	Actinobacteria;D_2__Actinobacteria;D_3__Micrococcales;D_4__Micrococcaceae;D_5__Rothia;D_6__uncultured organism;D_7__;D_8__;D_9__;D_10__;D_11__;D_12__;D_13__;D_14__	2.09(0.15, 29.87)	0.99(0.08, 12.19)	0.48(0.08, 2.74)	0.43(0.03, 5.31)	0.89(0.15, 5.25)	2.24(0.16, 31.24)	1.11(0.09, 14.38)	0.50(0.08,2.94)	0.50(0.04, 6.29)	1.11(0.19,6.54)					
ASV_488	Proteobacteria;D_2__Gammaproteobacteria;D_3__Pasteurellales;D_4__Pasteurellaceae	6.03(0.11, 323.35)	1.10(0.03, 46.03)	0.18(0.01, 2.56)	0.33(0.01, 14.46)	2.00(0.13, 29.72)	5.30(0.10, 271.15)	1.21(0.03, 54.21)	0.23(0.02,3.34)	0.23(0.01, 10.07)	1.22(0.08,17.91)					
ASV_489	Actinobacteria;D_2__Actinobacteria;D_3__Actinomycetales;D_4__Actinomycetaceae;D_5__Actinomyces;D_6__Actinomyces graevenitzii F0530;D_7__;D_8__;D_9__;D_10__;D_11__;D_12__;D_13__;D_14__	0.86(0.11, 6.73)	0.33(0.05, 2.27)	0.38(0.09, 1.53)	1.84(0.26, 12.96)	1.58(0.39, 6.48)	0.69(0.09, 5.32)	0.26(0.04, 1.91)	0.38(0.09,1.58)	2.33(0.33, 16.67)	1.61(0.39,6.65)					
ASV_490	Firmicutes;D_2__Clostridia;D_3__Clostridiales;D_4__Lachnospiraceae;D_5__[Ruminococcus] gnavus group	0.17(0.03, 0.97);p=0.04666	0.38(0.07, 2.00)	2.31(0.71, 7.48)	6.52(1.21, 35.05);p=0.02886	1.08(0.32, 3.60)	0.21(0.04, 1.25)	0.42(0.08, 2.33)	1.98(0.59,6.63)	7.37(1.35, 40.22);p=0.02101	1.57(0.47,5.29)				↑	
ASV_494	Proteobacteria;D_2__Gammaproteobacteria;D_3__Betaproteobacteriales;D_4__Neisseriaceae;D_5__Neisseria;D_6__uncultured bacterium;D_7__;D_8__;D_9__;D_10__;D_11__;D_12__;D_13__;D_14__	1.12(0.00, Inf)	2571299133093.16(0.00,Inf)	2303674369234.77(0.00,Inf)	0.00(0.00, Inf)	0.00(0.00, Inf)	0.99(0.00,Inf)	462234419997601.00(0.00,Inf)	469592707532129.00(0.00,Inf)	0.00(0.00, Inf)	0.00(0.00,Inf)					
ASV_502	Bacteroidetes;D_2__Bacteroidia;D_3__Bacteroidales;D_4__Bacteroidaceae;D_5__Bacteroides;D_6__Bacteroides fragilis;D_7__;D_8__;D_9__;D_10__;D_11__;D_12__;D_13__;D_14__	0.61(0.06, 5.96)	0.53(0.06, 4.51)	0.88(0.19, 3.98)	0.69(0.08, 6.01)	0.42(0.09, 1.97)										
ASV_504	Firmicutes;D_2__Erysipelotrichia;D_3__Erysipelotrichales;D_4__Erysipelotrichaceae;D_5__Turicibacter	6.31(1.55, 25.64);p=0.01	1.97(0.53, 7.33)	0.31(0.12, 0.79);p=0.01406	0.16(0.04, 0.59);p=0.00633	0.99(0.38, 2.57)	7.26(1.79, 29.45);p=0.00551	2.39(0.62, 9.22)	0.33(0.13,0.85);p=0.02229	0.12(0.03, 0.46);p=0.00204	0.88(0.34,2.28)	↑		↓	↓	
ASV_505	Firmicutes;D_2__Clostridia;D_3__Clostridiales;D_4__Lachnospiraceae;D_5__Tyzzerella 3;D_6__unidentified;D_7__;D_8__;D_9__;D_10__;D_11__;D_12__;D_13__;D_14__	0.99(0.02, 52.82)	0.42(0.01, 17.33)	0.42(0.03, 5.91)	3.11(0.07, 135.56)	3.07(0.21, 45.80)										
ASV_506	Firmicutes;D_2__Clostridia;D_3__Clostridiales;D_4__Lachnospiraceae	1.12(0.00, Inf)	48831248208.73(0.00,Inf)	43737626770.86(0.00,Inf)	0.00(0.00, Inf)	0.00(0.00, Inf)										
ASV_508	Firmicutes;D_2__Clostridia;D_3__Clostridiales;D_4__Ruminococcaceae;D_5__Ruminococcaceae UCG-005	0.23(0.02, 3.31)	1.19(0.10, 14.33)	5.18(0.88, 30.56)	0.99(0.08, 12.30)	0.23(0.04, 1.41)	0.26(0.02, 3.46)	0.71(0.06, 8.45)	2.69(0.46,15.81)	1.25(0.11, 14.61)	0.33(0.06,1.93)					
ASV_509	Firmicutes;D_2__Clostridia;D_3__Clostridiales;D_4__Lachnospiraceae;D_5__[Eubacterium] ruminantium group;D_6__uncultured bacterium;D_7__;D_8__;D_9__;D_10__;D_11__;D_12__;D_13__;D_14__	7.04(0.14, 343.25)	8.15(0.21, 310.08)	1.16(0.09, 15.16)	0.24(0.01, 9.62)	1.70(0.12, 23.75)										
ASV_510	Firmicutes;D_2__Clostridia;D_3__Clostridiales;D_4__Lachnospiraceae;D_5__Blautia	4.62(0.09, 226.83)	8.68(0.23, 332.78)	1.88(0.14, 24.65)	0.40(0.01, 16.01)	1.84(0.13, 25.76)	12.82(0.29,568.33)	25.48(0.65,993.85)	1.99(0.15,26.08)	0.30(0.01, 11.37)	3.81(0.29,50.47)					
ASV_512	Firmicutes;D_2__Clostridia;D_3__Clostridiales;D_4__Lachnospiraceae;D_5__Lachnoclostridium	9.55(0.29, 311.98)	12.33(0.46,327.22)	1.29(0.14, 11.93)	0.38(0.01, 10.67)	3.66(0.37, 36.33)	8.50(0.31, 232.35)	2.41(0.10, 59.94)	0.28(0.03,2.46)	0.84(0.03, 20.98)	7.17(0.80,64.01)					
ASV_515	Firmicutes;D_2__Clostridia;D_3__Clostridiales;D_4__Ruminococcaceae;D_5__Ruminiclostridium 9	0.19(0.00, 33.67)	0.31(0.00, 39.22)	1.62(0.05, 50.15)	1.11(0.01, 148.90)	0.21(0.01, 7.11)										
ASV_	Firmicutes;D_2__Clostridia;D_3__Clostridiales;D_4__Ruminococcaceae;D_5__Negativibacillus;D_6__uncultured	0.21(0.00, 13.12)	0.53(0.01, 24.99)	2.52(0.16, 39.62)	1.38(0.03, 68.09)	0.29(0.02, 4.89)										

516	bacterium;D_7__;D_8__;D_9__;D_10__;D_11__;D_12__;D_13__;D_14__															
ASV_518	Firmicutes;D_2__Clostridia;D_3__Clostridiales;D_4__Peptostreptococcaceae;D_5__Romboutsia	2.15(0.00, 931.14)	1.04(0.00, 311.83)	0.48(0.01, 26.21)	0.05(0.00, 15.72)	0.11(0.00, 6.23)										
ASV_519	Bacteroidetes;D_2__Bacteroidia;D_3__Bacteroidales;D_4__Barnesiellaceae;D_5__Coprobacter	1.30(0.01, 199.43)	0.33(0.00, 36.39)	0.25(0.01, 7.06)	0.58(0.00, 68.16)	0.75(0.02, 22.89)										
ASV_524	Firmicutes;D_2__Clostridia;D_3__Clostridiales;D_4__Ruminococcaceae;D_5__Butyricicoccus	1.46(0.19, 11.02)	0.86(0.13, 5.69)	0.59(0.15, 2.24)	0.65(0.10, 4.40)	0.94(0.24, 3.72)	1.23(0.16, 9.30)	0.75(0.11, 5.32)	0.61(0.15,2.44)	0.69(0.10, 4.85)	0.85(0.21,3.40)					
ASV_526	Firmicutes;D_2__Clostridia;D_3__Clostridiales;D_4__Lachnospiraceae;D_5__uncultured;D_6__metagenome;D_7__;D_8__;D_9__;D_10__;D_11__;D_12__;D_13__;D_14__	15.61(0.92,263.72)	24.91(1.68,369.03);p=0.0194	1.60(0.33, 7.74)	0.05(0.00, 0.80);p=0.0337	0.82(0.16, 4.14)	18.73(1.00,351.19)	41.81(2.36,741.01);p=0.01092	2.23(0.44,11.36)	0.03(0.00, 0.59);p=0.0201	0.63(0.12,3.22)		↑		↓	
ASV_529	Firmicutes;D_2__Clostridia;D_3__Clostridiales;D_4__Ruminococcaceae;D_5__Ruminiclostridium 9	0.84(0.05, 12.85)	1.73(0.13, 22.29)	2.07(0.34, 12.62)	4.44(0.33, 59.18)	3.71(0.58, 23.81)										
ASV_533	Firmicutes;D_2__Clostridia;D_3__Clostridiales;D_4__Lachnospiraceae;D_5__Marvinbryantia						0.93(0.08, 10.29)	2.12(0.21, 21.52)	2.28(0.44,11.76)	0.42(0.04, 4.25)	0.39(0.08,2.05)					
ASV_536	Firmicutes;D_2__Clostridia;D_3__Clostridiales;D_4__Clostridiaceae 1;D_5__Clostridium sensu stricto 1	9.86(2.33, 41.76);p=0.00189	4.37(1.13, 16.87);p=0.03264	0.44(0.17, 1.15)	0.12(0.03, 0.48);p=0.00262	1.21(0.45, 3.21)	8.12(1.93, 34.19);p=0.00431	3.51(0.88, 14.09)	0.43(0.16,1.15)	0.16(0.04, 0.63);p=0.00877	1.28(0.48,3.42)	↑			↓	
ASV_537	Firmicutes;D_2__Clostridia;D_3__Clostridiales;D_4__Ruminococcaceae;D_5__Ruminiclostridium 5						4650063628071557.00(0.00,Inf)	88624629376968688.00(0.00,Inf)	19.06(0.50, 722.70)	0.00(0.00, Inf)	0.10(0.00,3.48)					
ASV_541	Firmicutes;D_2__Clostridia;D_3__Clostridiales;D_4__Ruminococcaceae;D_5__uncultured;D_6__unidentified;D_7__;D_8__;D_9__;D_10__;D_11__;D_12__;D_13__;D_14__	1.17(0.04, 30.65)	0.76(0.04, 16.18)	0.65(0.07, 5.65)	1.51(0.07, 33.57)	1.78(0.19, 16.39)	1.08(0.05, 24.64)	0.31(0.01, 6.48)	0.29(0.03,2.50)	1.68(0.08, 34.02)	1.82(0.21,15.58)					
ASV_543	Firmicutes;D_2__Clostridia;D_3__Clostridiales;D_4__Christensenellaceae;D_5__Christensenellaceae R-7 group	0.60(0.02, 23.01)	0.22(0.01, 6.82)	0.37(0.03, 4.33)	1.49(0.05, 47.23)	0.88(0.07, 10.71)	0.34(0.01, 12.43)	0.19(0.01, 6.22)	0.56(0.04,7.04)	1.32(0.04, 40.89)	0.45(0.04,5.46)					
ASV_545	Firmicutes;D_2__Clostridia;D_3__Clostridiales;D_4__Lachnospiraceae;D_5__[Eubacterium] ventriosum group;D_6__uncultured bacterium;D_7__;D_8__;D_9__;D_10__;D_11__;D_12__;D_13__;D_14__	1.52(0.01, 182.10)	3.65(0.04, 322.65)	2.41(0.10, 57.34)	1.88(0.02, 176.09)	2.85(0.11, 73.59)	0.69(0.01, 65.32)	11.01(0.14,889.56)	16.02(0.66, 386.69)	2.05(0.03, 161.06)	1.41(0.06,33.81)					
ASV_547	Firmicutes;D_2__Clostridia;D_3__Clostridiales;D_4__Ruminococcaceae;D_5__Oscillibacter;D_6__Oscillibacter sp. Marseille-P3260;D_7__;D_8__;D_9__;D_10__;D_11__;D_12__;D_13__;D_14__	0.52(0.01, 40.87)	5.91(0.11, 306.43)	11.35(0.62,208.83)	0.31(0.01, 17.12)	0.16(0.01, 3.22)										
ASV_549	Firmicutes;D_2__Clostridia;D_3__Clostridiales;D_4__Lachnospiraceae	2.05(0.18, 24.05)	2.41(0.24, 24.11)	1.17(0.23, 5.90)	0.64(0.06, 6.58)	1.31(0.25, 6.88)	2.16(0.20, 22.95)	4.05(0.41, 39.92)	1.88(0.38,9.23)	0.39(0.04, 3.84)	0.85(0.17,4.21)					
ASV_551	Firmicutes;D_2__Clostridia;D_3__Clostridiales;D_4__Lachnospiraceae;D_5__[Eubacterium] xylanophilum group	0.34(0.04, 3.26)	0.45(0.05, 3.77)	1.34(0.30, 6.00)	1.14(0.13, 9.72)	0.38(0.08, 1.79)	0.31(0.03, 2.87)	0.26(0.03, 2.23)	0.84(0.18,3.85)	1.45(0.17, 12.30)	0.45(0.10,2.05)					
ASV_	Cyanobacteria;D_2__Oxyphotobacteria;D_3__Chloroplast	0.00(0.00, Inf)	0.14(0.00, 5.29)	5798426835.96(0.00,Inf)	2.77(0.07, 105.37)	0.00(0.00, Inf)										

553																
ASV_557	Firmicutes;D_2__Clostridia;D_3__Clostridiales;D_4__Christensenellaceae;D_5__uncultured;D_6__uncultured bacterium;D_7__;D_8__;D_9__;D_10__;D_11__;D_12__;D_13__;D_14__	0.51(0.07,3.72)	0.34(0.05,2.17)	0.66(0.17,2.51)	2.42(0.37,15.73)	1.24(0.32,4.86)	0.55(0.08,4.00)	0.36(0.05,2.47)	0.66(0.17,2.61)	2.20(0.33,14.72)	1.22(0.31,4.80)					
ASV_560	Firmicutes;D_2__Clostridia;D_3__Clostridiales;D_4__Lachnospiraceae;D_5__Anaerostipes;D_6__unidentified;D_7__;D_8__;D_9__;D_10__;D_11__;D_12__;D_13__;D_14__						0.27(0.01,9.40)	0.14(0.00,4.23)	0.51(0.04,5.74)	7.50(0.25,223.51)	2.06(0.18,23.52)					
ASV_561	Actinobacteria;D_2__Coriobacteriia;D_3__Coriobacteriales;D_4__Coriobacteriales Incertae Sedis;D_5__Raoultibacter;D_6__Raoultibacter timonensis;D_7__;D_8__;D_9__;D_10__;D_11__;D_12__;D_13__;D_14__	5948776308.75(0.00,Inf)	1853914646.96(0.00,Inf)	0.31(0.03,3.15)	0.00(0.00,Inf)	1.04(0.10,10.74)	15491816281510906.00(0.00,Inf)	4134265171864758.00(0.00,Inf)	0.27(0.02,2.88)	0.00(0.00,Inf)	0.53(0.05,5.20)					
ASV_564	Firmicutes;D_2__Clostridia;D_3__Clostridiales;D_4__Ruminococcaceae	1.19(0.01,124.21)	0.11(0.00,10.03)	0.09(0.00,2.54)	6.77(0.07,647.76)	8.05(0.28,232.31)										
ASV_565	Firmicutes;D_2__Clostridia;D_3__Clostridiales;D_4__Ruminococcaceae;D_5__Oscillibacter;D_6__uncultured bacterium;D_7__;D_8__;D_9__;D_10__;D_11__;D_12__;D_13__;D_14__	0.00(0.00,Inf)	0.05(0.00,9.19)	338639721629.05(0.00,Inf)	4.38(0.03,760.63)	0.00(0.00,Inf)										
ASV_566	Firmicutes;D_2__Clostridia;D_3__Clostridiales;D_4__Ruminococcaceae	4.73(0.42,53.29)	0.59(0.06,5.74)	0.13(0.03,0.62);p=0.01122	1.51(0.15,15.08)	7.15(1.38,37.03);p=0.01904	4.96(0.44,55.48)	0.75(0.07,7.77)	0.15(0.03,0.79);p=0.02483	1.62(0.16,16.49)	8.03(1.54,41.92);p=0.01346			↓		↑
ASV_567	Firmicutes;D_2__Clostridia;D_3__Clostridiales;D_4__Ruminococcaceae;D_5__Butyricicoccus;D_6__uncultured bacterium;D_7__;D_8__;D_9__;D_10__;D_11__;D_12__;D_13__;D_14__	0.20(0.01,8.19)	0.17(0.01,5.39)	0.83(0.07,9.66)	2.36(0.07,78.52)	0.48(0.04,5.92)										
ASV_568	Firmicutes;D_2__Clostridia;D_3__Clostridiales;D_4__Clostridiales vadinBB60 group;D_5__gut metagenome;D_6__;D_7__;D_8__;D_9__;D_10__;D_11__;D_12__;D_13__;D_14__	1445410934.93(0.00,Inf)	4323942160.80(0.00,Inf)	2.99(0.35,25.30)	0.00(0.00,Inf)	0.25(0.03,2.21)	21385215667351500.00(0.00,Inf)	18254632570369308.00(0.00,Inf)	0.85(0.10,7.49)	0.00(0.00,Inf)	0.26(0.03,2.27)					
ASV_570	Firmicutes;D_2__Clostridia;D_3__Clostridiales;D_4__Peptococcaceae;D_5__uncultured;D_6__uncultured bacterium;D_7__;D_8__;D_9__;D_10__;D_11__;D_12__;D_13__;D_14__	4166247068302.62(0.00,Inf)	2655762868003.35(0.00,Inf)	0.64(0.02,18.39)	0.00(0.00,Inf)	0.38(0.01,9.94)										
ASV_572	Firmicutes;D_2__Clostridia;D_3__Clostridiales;D_4__Christensenellaceae;D_5__Christensenellaceae R-7 group;D_6__uncultured marine bacterium;D_7__;D_8__;D_9__;D_10__;D_11__;D_12__;D_13__;D_14__	0.63(0.15,2.70)	0.53(0.14,2.10)	0.85(0.32,2.26)	2.36(0.59,9.45)	1.48(0.54,4.02)	0.66(0.16,2.82)	0.43(0.11,1.72)	0.64(0.24,1.73)	2.35(0.58,9.43)	1.56(0.57,4.22)					
ASV_573	Firmicutes;D_2__Bacilli;D_3__Lactobacillales;D_4__Streptococcaceae;D_5__Streptococcus;D_6__Streptococcus mutans;D_7__;D_8__;D_9__;D_10__;D_11__;D_12__;D_13__;D_14__	0.52(0.07,4.01)	0.33(0.05,2.26)	0.64(0.16,2.51)	0.70(0.10,4.83)	0.36(0.09,1.45)	0.57(0.07,4.42)	0.46(0.06,3.29)	0.80(0.20,3.25)	0.52(0.07,3.65)	0.30(0.07,1.20)					
ASV_576	Actinobacteria;D_2__Actinobacteria;D_3__Actinomycetales;D_4__Actinomycetaceae;D_5__Actinomyces	205538075683.89(0.00,Inf)	75633178405.34(0.00,Inf)	0.37(0.02,5.76)	0.00(0.00,Inf)	0.98(0.07,14.74)										
ASV_580	Firmicutes;D_2__Clostridia;D_3__Clostridiales;D_4__Ruminococcaceae;D_5__Anaerotruncus;D_6__uncultured bacterium;D_7__;D_8__;D_9__;D_10__;D_11__;D_12__;D_13__;D_14__	0.21(0.00,17.38)	0.24(0.00,14.05)	1.17(0.05,27.83)	1.26(0.02,71.97)	0.26(0.01,6.08)										
ASV_581	Firmicutes;D_2__Erysipelotrichia;D_3__Erysipelotrichales;D_4__Erysipelotrichaceae;D_5__Solobacterium	1.06(0.07,15.10)	0.45(0.04,5.57)	0.43(0.07,2.58)	1.23(0.10,15.32)	1.30(0.21,7.96)										

ASV_582	Firmicutes;D_2__Clostridia;D_3__Clostridiales;D_4__Family XIII;D_5__Family XIII AD3011 group;D_6__gut metagenome;D_7__;D_8__;D_9__;D_10__;D_11__;D_12__;D_13__;D_14__	0.11(0.00,10.66)	0.80(0.01,52.43)	7.16(0.32,158.13)	36.37(0.42,3146.73)	4.09(0.13,130.93)										
ASV_585	Firmicutes;D_2__Clostridia;D_3__Clostridiales;D_4__Ruminococcaceae;D_5__Ruminiclostridium 5;D_6__uncultured bacterium;D_7__;D_8__;D_9__;D_10__;D_11__;D_12__;D_13__;D_14__	0.30(0.02,4.71)	0.65(0.05,8.41)	2.17(0.34,13.77)	2.70(0.20,36.14)	0.82(0.12,5.46)	0.32(0.02,4.86)	0.74(0.05,10.07)	2.32(0.35,15.47)	2.38(0.18,32.19)	0.76(0.11,5.08)					
ASV_586	Firmicutes;D_2__Clostridia;D_3__Clostridiales;D_4__Ruminococcaceae;D_5__Anaerotruncus;D_6__uncultured organism;D_7__;D_8__;D_9__;D_10__;D_11__;D_12__;D_13__;D_14__	0.54(0.11,2.59)	1.19(0.28,5.05)	2.19(0.77,6.23)	1.14(0.26,4.94)	0.62(0.21,1.81)	0.53(0.11,2.55)	1.09(0.24,4.89)	2.06(0.70,6.05)	1.14(0.26,5.04)	0.60(0.20,1.79)					
ASV_588	Firmicutes;D_2__Erysipelotrichia;D_3__Erysipelotrichales;D_4__Erysipelotrichaceae;D_5__uncultured;D_6__Clostridiales bacterium 60-7e;D_7__;D_8__;D_9__;D_10__;D_11__;D_12__;D_13__;D_14__	0.19(0.00,12.07)	0.01(0.00,0.46);p=0.01861	0.05(0.00,0.78);p=0.03272	10.14(0.20,520.51)	1.92(0.11,32.33)										
ASV_590	Firmicutes;D_2__Clostridia;D_3__Clostridiales;D_4__Lachnospiraceae	0.10(0.01,1.49)	0.15(0.01,1.56)	1.53(0.17,13.85)	2.12(0.21,21.28)	0.20(0.02,1.72)										
ASV_594	Firmicutes;D_2__Clostridia;D_3__Clostridiales;D_4__Ruminococcaceae;D_5__Candidatus Soleaferrea;D_6__Bittarella massiliensis;D_7__;D_8__;D_9__;D_10__;D_11__;D_12__;D_13__;D_14__	0.41(0.11,1.61)	0.75(0.21,2.66)	1.82(0.73,4.55)	1.55(0.43,5.58)	0.64(0.25,1.62)	0.39(0.10,1.52)	0.61(0.17,2.25)	1.56(0.61,3.97)	1.94(0.53,7.07)	0.76(0.30,1.95)					
ASV_595	Firmicutes;D_2__Clostridia;D_3__Clostridiales;D_4__Lachnospiraceae						10377552945231754.00(0.00,Inf)	26484922573212692.00(0.00,Inf)	2.55(0.25,25.62)	0.00(0.00,Inf)	0.48(0.05,4.81)					
ASV_596	Firmicutes;D_2__Clostridia;D_3__Clostridiales;D_4__Lachnospiraceae;D_5__[Eubacterium] fissicatena group	1.96(0.43,8.88)	1.29(0.31,5.35)	0.66(0.24,1.79)	0.65(0.15,2.73)	1.27(0.46,3.53)	2.00(0.44,9.09)	1.17(0.27,5.06)	0.59(0.21,1.63)	0.71(0.17,3.04)	1.42(0.51,3.98)					
ASV_597	Firmicutes;D_2__Clostridia;D_3__Clostridiales;D_4__Lachnospiraceae;D_5__Lachnoclostridium;D_6__[Clostridium] scindens;D_7__;D_8__;D_9__;D_10__;D_11__;D_12__;D_13__;D_14__	1.35(0.10,18.93)	1.40(0.12,16.61)	1.04(0.18,5.98)	4.08(0.33,50.24)	5.49(0.91,33.23)	1.23(0.09,17.39)	1.34(0.10,17.27)	1.09(0.18,6.64)	4.38(0.34,56.02)	5.37(0.87,33.17)					
ASV_599	Firmicutes;D_2__Clostridia;D_3__Clostridiales;D_4__Ruminococcaceae;D_5__Ruminiclostridium 9	1.00(0.15,6.55)	2.48(0.43,14.27)	2.48(0.72,8.54)	0.33(0.06,1.91)	0.33(0.09,1.15)	0.79(0.12,5.11)	1.60(0.27,9.61)	2.03(0.57,7.23)	0.41(0.07,2.40)	0.32(0.09,1.14)					
ASV_600	Firmicutes;D_2__Clostridia;D_3__Clostridiales;D_4__Lachnospiraceae;D_5__UC5-1-2E3;D_6__uncultured bacterium;D_7__;D_8__;D_9__;D_10__;D_11__;D_12__;D_13__;D_14__	0.29(0.01,5.79)	0.92(0.06,14.92)	3.16(0.43,23.16)	2.63(0.16,44.40)	0.77(0.10,5.95)										
ASV_601	Firmicutes;D_2__Clostridia;D_3__Clostridiales;D_4__Lachnospiraceae	3.83(0.35,42.25)	2.50(0.26,23.83)	0.65(0.14,3.13)	1.02(0.10,10.07)	3.90(0.77,19.69)	4.50(0.42,48.18)	3.91(0.39,38.96)	0.87(0.18,4.19)	0.76(0.08,7.61)	3.44(0.70,16.98)					
ASV_609	Bacteroidetes;D_2__Bacteroidia;D_3__Bacteroidales;D_4__Bacteroidaceae;D_5__Bacteroides;D_6__Bacteroides ovatus;D_7__;D_8__;D_9__;D_10__;D_11__;D_12__;D_13__;D_14__	1.12(0.02,78.98)	0.69(0.01,36.84)	0.61(0.04,10.23)	0.66(0.01,37.17)	0.74(0.04,13.33)	0.49(0.01,30.28)	0.94(0.02,50.86)	1.94(0.12,32.55)	1.96(0.04,103.50)	0.95(0.06,16.13)					
ASV_613	Firmicutes;D_2__Erysipelotrichia;D_3__Erysipelotrichales;D_4__Erysipelotrichaceae;D_5__Turicibacter	2.25(0.16,32.31)	0.79(0.07,9.54)	0.35(0.06,2.04)	0.98(0.08,12.18)	2.20(0.36,13.42)	4.81(0.35,65.93)	1.27(0.10,15.96)	0.26(0.04,1.58)	0.66(0.05,8.16)	3.17(0.53,19.03)					
ASV_620	Firmicutes;D_2__Clostridia;D_3__Clostridiales;D_4__Lachnospiraceae;D_5__uncultured;D_6__uncultured organism;D_7__;D_8__;D_9__;D_10__;D_11__;D_12__;D_13__;D_14__	0.21(0.01,3.13)	0.60(0.05,7.41)	2.84(0.47,17.21)	3.52(0.27,45.29)	0.74(0.12,4.70)	0.26(0.02,3.83)	0.45(0.03,5.88)	1.70(0.27,10.69)	3.08(0.24,40.17)	0.81(0.13,5.14)					

ASV_621	Firmicutes;D_2__Clostridia;D_3__Clostridiales;D_4__Ruminococcaceae;D_5__Intestinimonas;D_6__Intestinimonas butyriciproducens;D_7__;D_8__;D_9__;D_10__;D_11__;D_12__;D_13__;D_14__	1.27(0.17, 9.30)	1.15(0.18, 7.37)	0.90(0.24, 3.34)	1.01(0.15, 6.66)	1.29(0.33, 4.96)	1.09(0.16, 7.58)	0.79(0.12, 5.13)	0.72(0.19,2.71)	1.15(0.18, 7.43)	1.25(0.33,4.73)					
ASV_623	Firmicutes;D_2__Clostridia;D_3__Clostridiales;D_4__Lachnospiraceae;D_5__[Ruminococcus] torques group;D_6__uncultured bacterium;D_7__;D_8__;D_9__;D_10__;D_11__;D_12__;D_13__;D_14__	0.25(0.02, 2.95)	0.73(0.07, 7.32)	2.91(0.57, 14.97)	3.31(0.32, 34.20)	0.83(0.15, 4.46)	0.44(0.04, 5.03)	1.19(0.11, 12.56)	2.72(0.51,14.49)	2.95(0.28, 30.78)	1.29(0.24,6.91)					
ASV_628	Firmicutes;D_2__Clostridia;D_3__Clostridiales;D_4__Ruminococcaceae;D_5__Butyricicoccus	1.34(0.26, 6.84)	1.56(0.34, 7.15)	1.16(0.40, 3.38)	1.16(0.25, 5.46)	1.56(0.52, 4.72)	1.19(0.25, 5.69)	1.01(0.22, 4.60)	0.85(0.29,2.47)	1.96(0.43, 8.90)	2.33(0.79,6.86)					
ASV_630	Firmicutes;D_2__Clostridia;D_3__Clostridiales;D_4__Clostridiaceae 1;D_5__Clostridium sensu stricto 1	1.14(0.02, 58.42)	2.19(0.06, 86.25)	1.92(0.14, 25.55)	0.15(0.00, 6.29)	0.18(0.01, 2.46)										
ASV_637	Firmicutes;D_2__Clostridia;D_3__Clostridiales;D_4__Ruminococcaceae;D_5__Ruminococcaceae UCG-005	0.07(0.00, 1.18)	0.17(0.01, 1.92)	2.43(0.27, 21.67)	3.14(0.27, 36.18)	0.21(0.02, 1.91)	0.10(0.01, 1.30)	0.29(0.03, 2.86)	2.98(0.33,26.73)	2.11(0.23, 19.64)	0.21(0.02,1.73)					
ASV_641	Actinobacteria;D_2__Coriobacteriia;D_3__Coriobacteriales;D_4__Coriobacteriaceae;D_5__Collinsella;D_6__uncultured bacterium;D_7__;D_8__;D_9__;D_10__;D_11__;D_12__;D_13__;D_14__	0.25(0.01, 7.24)	0.99(0.04, 22.43)	4.05(0.41, 39.81)	1.03(0.04, 24.25)	0.25(0.02, 2.64)	0.24(0.01, 5.93)	0.56(0.03, 11.84)	2.34(0.25,22.21)	1.87(0.09, 39.08)	0.44(0.05,4.24)					
ASV_642	Firmicutes;D_2__Clostridia;D_3__Clostridiales;D_4__Lachnospiraceae	1.25(0.07, 22.21)	4.40(0.30, 64.73)	3.53(0.53, 23.43)	0.33(0.02, 5.01)	0.41(0.06, 2.85)	1.17(0.07, 19.98)	2.70(0.18, 41.45)	2.31(0.34,15.71)	0.32(0.02, 4.89)	0.38(0.06,2.60)					
ASV_643	Firmicutes;D_2__Clostridia;D_3__Clostridiales;D_4__Ruminococcaceae;D_5__Candidatus Soleaferrea;D_6__uncultured Anaerotruncus sp.;D_7__;D_8__;D_9__;D_10__;D_11__;D_12__;D_13__;D_14__	1.05(0.14, 7.88)	2.58(0.39, 16.92)	2.47(0.66, 9.30)	0.88(0.13, 5.93)	0.92(0.23, 3.61)	1.00(0.14, 7.18)	1.17(0.18, 7.75)	1.17(0.31,4.41)	0.83(0.13, 5.47)	0.83(0.22,3.18)					
ASV_644	Firmicutes;D_2__Clostridia;D_3__Clostridiales;D_4__Lachnospiraceae;D_5__Lachnospiraceae UCG-008;D_6__uncultured organism;D_7__;D_8__;D_9__;D_10__;D_11__;D_12__;D_13__;D_14__	0.60(0.03, 10.94)	0.44(0.03, 6.71)	0.74(0.11, 5.10)	4.23(0.27, 66.71)	2.55(0.35, 18.74)	0.43(0.03, 6.31)	0.84(0.06, 11.16)	1.96(0.28,13.63)	1.61(0.12, 21.09)	0.69(0.10,4.73)					
ASV_646	Firmicutes;D_2__Clostridia;D_3__Clostridiales;D_4__Ruminococcaceae	0.46(0.02, 10.60)	1.50(0.09, 23.65)	3.27(0.37, 29.01)	0.39(0.02, 6.23)	0.18(0.02, 1.61)	0.50(0.02, 10.46)	2.51(0.16, 39.85)	5.03(0.54,46.86)	0.32(0.02, 4.86)	0.16(0.02,1.42)					
ASV_647	Firmicutes;D_2__Clostridia;D_3__Clostridiales;D_4__Ruminococcaceae;D_5__Acetanaerobacterium;D_6__uncultured rumen bacterium;D_7__;D_8__;D_9__;D_10__;D_11__;D_12__;D_13__;D_14__	2.65(0.33, 21.21)	1.10(0.15, 7.83)	0.42(0.11, 1.63)	1.67(0.23, 12.35)	4.43(1.07, 18.27);p=0.03973	2.61(0.32, 21.08)	1.06(0.14, 8.09)	0.41(0.10,1.67)	1.72(0.23, 13.09)	4.49(1.07,18.82);p=0.03996					↑
ASV_648	Firmicutes;D_2__Clostridia;D_3__Clostridiales;D_4__Lachnospiraceae;D_5__Lachnospiraceae UCG-004;D_6__uncultured organism;D_7__;D_8__;D_9__;D_10__;D_11__;D_12__;D_13__;D_14__	1.12(0.00, Inf)	2884785181.49(0.00,Inf)	2583870973.64(0.00,Inf)	0.00(0.00, Inf)	0.00(0.00, Inf)	1.40(0.00,Inf)	1900023995063915.00(0.00,Inf)	1354292271422123.00(0.00,Inf)	0.00(0.00, Inf)	0.00(0.00,Inf)					
ASV_649	Firmicutes;D_2__Clostridia;D_3__Clostridiales;D_4__Clostridiaceae 1;D_5__Clostridium sensu stricto 1	3.30(0.02, 615.06)	10.19(0.08,1379.49)	3.09(0.12, 77.90)	0.32(0.00, 46.68)	1.05(0.04, 29.83)										
ASV_650	Firmicutes;D_2__Clostridia;D_3__Clostridiales;D_4__Christensenellaceae;D_5__Catabacter	1.17(0.12, 11.38)	0.89(0.11, 7.55)	0.76(0.17, 3.47)	0.82(0.09, 7.05)	0.95(0.20, 4.44)	1.92(0.21, 17.15)	0.75(0.09, 6.44)	0.39(0.09,1.72)	0.70(0.08, 5.83)	1.35(0.31,5.81)					
ASV_651	Actinobacteria;D_2__Actinobacteria;D_3__Micrococcales;D_4__Micrococcaceae;D_5__Rothia;D_6__uncultured organism;D_7__;D_8__;D_9__;D_10__;D_11__;D_12__;D_13__;D_14__	17.07(0.10,2952.23)	1.73(0.01, 228.91)	0.10(0.00, 2.73)	1.54(0.01, 230.10)	26.22(0.81,844.95)										

ASV_652	Proteobacteria;D_2__Gammaproteobacteria;D_3__Betaproteobacteriales;D_4__Burkholderiaceae;D_5__Noviherbaspirillum	72016310094.10(0.00,Inf)	177421877003.08(0.00,Inf)	2.46(0.07,91.98)	0.00(0.00,Inf)	0.13(0.00,5.14)										
ASV_653	Firmicutes;D_2__Clostridia;D_3__Clostridiales;D_4__Ruminococcaceae;D_5__GCA-900066225;D_6__Massilimaliae massiliensis;D_7__;D_8__;D_9__;D_10__;D_11__;D_12__;D_13__;D_14__	0.16(0.01,3.49)	0.34(0.02,5.41)	2.05(0.24,17.72)	3.08(0.19,51.31)	0.51(0.06,4.60)	0.12(0.01,2.45)	0.25(0.01,4.36)	2.16(0.23,20.30)	3.70(0.22,62.49)	0.43(0.05,4.06)					
ASV_656	Firmicutes;D_2__Clostridia;D_3__Clostridiales;D_4__Family XIII;D_5__Family XIII AD3011 group;D_6__uncultured bacterium;D_7__;D_8__;D_9__;D_10__;D_11__;D_12__;D_13__;D_14__	1.11(0.10,12.15)	1.34(0.14,12.62)	1.21(0.25,5.92)	4.76(0.47,47.95)	5.27(0.98,28.25)	1.26(0.13,11.91)	0.49(0.05,4.35)	0.39(0.08,1.81)	6.84(0.73,63.89)	8.61(1.70,43.67);p=0.00933					↑
ASV_658	Firmicutes;D_2__Clostridia;D_3__Clostridiales;D_4__Ruminococcaceae;D_5__Flavonifractor;D_6__uncultured bacterium;D_7__;D_8__;D_9__;D_10__;D_11__;D_12__;D_13__;D_14__	1.83(0.27,12.35)	3.85(0.65,22.88)	2.10(0.61,7.19)	0.52(0.08,3.15)	0.95(0.27,3.38)	2.02(0.30,13.68)	4.09(0.65,25.84)	2.02(0.57,7.15)	0.36(0.06,2.26)	0.73(0.20,2.60)					
ASV_669	Firmicutes;D_2__Clostridia;D_3__Clostridiales;D_4__Lachnospiraceae;D_5__[Eubacterium] hallii group	1.12(0.00,Inf)	169210660661.55(0.00,Inf)	151560188602.34(0.00,Inf)	0.00(0.00,Inf)	0.00(0.00,Inf)	0.99(0.00,Inf)	95243657714741184.00(0.00,Inf)	97798843999772592.00(0.00,Inf)	0.00(0.00,Inf)	0.00(0.00,Inf)					
ASV_672	Firmicutes;D_2__Bacilli;D_3__Lactobacillales;D_4__Lactobacillaceae;D_5__Lactobacillus	1.92(0.01,324.55)	0.81(0.01,104.94)	0.42(0.01,12.50)	0.61(0.00,80.36)	1.17(0.04,35.56)										
ASV_676	Firmicutes;D_2__Bacilli;D_3__Lactobacillales;D_4__Lactobacillaceae;D_5__Lactobacillus	101.02(4.91,2079.15);p=0.00278	20.08(1.18,341.73);p=0.03808	0.20(0.03,1.45)	0.40(0.02,7.08)	40.52(5.26,312.30);p=0.00038	93.16(5.37,1615.15);p=0.00184	16.29(1.03,257.78);p=0.04759	0.17(0.03,1.20)	0.18(0.01,2.84)	16.97(2.46,117.17);p=0.00407	↑	↑			↑
ASV_678	Firmicutes;D_2__Erysipelotrichia;D_3__Erysipelotrichales;D_4__Erysipelotrichaceae;D_5__Erysipelatoclostridium	2.14(0.52,8.84)	0.63(0.17,2.39)	0.30(0.12,0.76);p=0.01091	1.28(0.33,4.92)	2.75(1.05,7.18);p=0.03964	2.12(0.52,8.70)	0.66(0.17,2.59)	0.31(0.12,0.82);p=0.01801	1.24(0.32,4.82)	2.63(1.00,6.91);p=0.04975			↓		↑
ASV_679	Firmicutes;D_2__Clostridia;D_3__Clostridiales;D_4__Peptostreptococcaceae;D_5__Paeniclostridium;D_6__uncultured bacterium;D_7__;D_8__;D_9__;D_10__;D_11__;D_12__;D_13__;D_14__	401101428041.61(0.00,Inf)	21553921064.02(0.00,Inf)	0.05(0.01,0.56);p=0.01461	0.00(0.00,Inf)	8.55(0.81,90.17)										
ASV_680	Firmicutes;D_2__Clostridia;D_3__Clostridiales;D_4__Ruminococcaceae;D_5__Butyricicoccus	0.20(0.00,12.03)	0.77(0.02,34.95)	3.80(0.25,57.14)	0.20(0.00,9.42)	0.04(0.00,0.65);p=0.02365	0.20(0.00,11.01)	0.71(0.01,34.31)	3.62(0.23,57.31)	0.21(0.00,9.87)	0.04(0.00,0.65);p=0.02332					↓
ASV_683	Firmicutes;D_2__Clostridia;D_3__Clostridiales;D_4__Ruminococcaceae;D_5__Ruminiclostridium 5;D_6__uncultured organism;D_7__;D_8__;D_9__;D_10__;D_11__;D_12__;D_13__;D_14__	1.15(0.22,6.14)	1.24(0.26,5.94)	1.08(0.36,3.25)	1.06(0.22,5.17)	1.22(0.39,3.80)	1.10(0.22,5.61)	0.91(0.19,4.36)	0.82(0.27,2.49)	1.12(0.23,5.32)	1.23(0.41,3.74)					
ASV_685	Firmicutes;D_2__Clostridia;D_3__Clostridiales;D_4__Lachnospiraceae;D_5__[Ruminococcus] torques group	0.04(0.00,0.81);p=0.03637	0.13(0.01,2.33)	3.57(0.45,28.68)	5.26(0.28,97.38)	0.19(0.02,1.61)	0.02(0.00,0.41);p=0.01165	0.08(0.00,1.59)	4.43(0.51,38.42)	6.76(0.35,128.89)	0.12(0.01,1.08)	↓				
ASV_692	Actinobacteria;D_2__Actinobacteria;D_3__Bifidobacteriales;D_4__Bifidobacteriaceae;D_5__Alloscardovia;D_6__Bifidobacterium longum subsp. longum;D_7__;D_8__;D_9__;D_10__;D_11__;D_12__;D_13__;D_14__	0.89(0.01,114.40)	0.62(0.01,59.00)	0.70(0.03,18.07)	0.88(0.01,85.78)	0.78(0.03,21.23)										
ASV_695	Actinobacteria;D_2__Actinobacteria;D_3__Micrococcales;D_4__Micrococcaceae;D_5__Rothia	3.91(0.20,74.89)	1.75(0.11,28.21)	0.45(0.07,3.04)	0.39(0.02,6.50)	1.53(0.22,10.86)	2.64(0.14,49.67)	1.77(0.10,30.39)	0.67(0.09,4.81)	0.37(0.02,6.27)	0.99(0.14,7.04)					
ASV_696	Actinobacteria;D_2__Actinobacteria;D_3__Actinomycetales;D_4__Actinomycetaceae;D_5__Actinomyces	0.79(0.05,12.81)	0.64(0.05,8.61)	0.80(0.12,5.32)	1.00(0.07,13.72)	0.79(0.12,5.34)	0.86(0.06,12.66)	1.03(0.08,13.87)	1.19(0.18,7.85)	0.93(0.07,12.26)	0.80(0.12,5.26)					

ASV_697	Firmicutes;D_2__Erysipelotrichia;D_3__Erysipelotrichales;D_4__Erysipelotrichaceae;D_5__Faecalitalea	1.26(0.12,12.90)	1.65(0.19,14.58)	1.31(0.28,6.12)	1.39(0.15,12.58)	1.75(0.36,8.48)	1.69(0.17,16.84)	1.61(0.18,14.89)	0.96(0.20,4.59)	1.61(0.18,14.65)	2.71(0.56,13.08)					
ASV_698	Firmicutes;D_2__Clostridia;D_3__Clostridiales;D_4__Lachnospiraceae;D_5__Sellimonas;D_6__Lachnoclostridium phocaeense;D_7__;D_8__;D_9__;D_10__;D_11__;D_12__;D_13__;D_14__	0.22(0.00,21.72)	4.59(0.07,309.14)	21.14(0.94,476.77)	1.26(0.02,90.59)	0.27(0.01,6.75)										
ASV_699	Bacteroidetes;D_2__Bacteroidia;D_3__Bacteroidales;D_4__Prevotellaceae;D_5__Prevotella 6;D_6__uncultured bacterium;D_7__;D_8__;D_9__;D_10__;D_11__;D_12__;D_13__;D_14__	0.00(0.00,Inf)	0.24(0.00,20.84)	907326702.63(0.00,Inf)	0.72(0.01,57.17)	0.00(0.00,Inf)										
ASV_702	Firmicutes;D_2__Clostridia;D_3__Clostridiales;D_4__Ruminococcaceae;D_5__uncultured;D_6__human gut metagenome;D_7__;D_8__;D_9__;D_10__;D_11__;D_12__;D_13__;D_14__	0.18(0.01,5.88)	0.59(0.02,15.21)	3.33(0.32,34.85)	3.12(0.12,83.97)	0.55(0.05,6.20)										
ASV_703	Firmicutes;D_2__Clostridia;D_3__Clostridiales;D_4__Christensenellaceae;D_5__Christensenellaceae R-7 group;D_6__bacterium YE57;D_7__;D_8__;D_9__;D_10__;D_11__;D_12__;D_13__;D_14__	2.94(0.02,423.78)	0.28(0.00,30.43)	0.10(0.00,2.64)	0.29(0.00,31.97)	0.84(0.03,24.18)										
ASV_704	Firmicutes;D_2__Clostridia;D_3__Clostridiales;D_4__Ruminococcaceae;D_5__uncultured;D_6__uncultured bacterium;D_7__;D_8__;D_9__;D_10__;D_11__;D_12__;D_13__;D_14__	0.25(0.02,4.22)	0.41(0.03,5.41)	1.62(0.23,11.46)	1.25(0.09,16.74)	0.32(0.04,2.28)	0.26(0.02,4.21)	0.33(0.02,4.65)	1.28(0.17,9.53)	1.29(0.10,17.56)	0.33(0.05,2.39)					
ASV_705	Firmicutes;D_2__Clostridia;D_3__Clostridiales;D_4__Family XI;D_5__Ezakiella;D_6__Fenollaria timonensis;D_7__;D_8__;D_9__;D_10__;D_11__;D_12__;D_13__;D_14__	0.22(0.01,3.62)	1.14(0.09,14.59)	5.20(0.76,35.47)	0.44(0.03,5.72)	0.10(0.01,0.68);p=0.01881	0.12(0.01,1.86)	0.40(0.03,5.00)	3.23(0.47,22.19)	1.38(0.11,17.06)	0.17(0.02,1.17)					
ASV_708	Firmicutes;D_2__Clostridia;D_3__Clostridiales;D_4__Ruminococcaceae;D_5__Hydrogenoanaerobacterium;D_6__uncultured bacterium;D_7__;D_8__;D_9__;D_10__;D_11__;D_12__;D_13__;D_14__	90639928245.49(0.00,Inf)	102280098194.16(0.00,Inf)	1.13(0.07,19.02)	0.00(0.00,Inf)	1.18(0.06,21.60)										
ASV_710	Firmicutes;D_2__Clostridia;D_3__Clostridiales;D_4__Lachnospiraceae;D_5__Lachnospiraceae UCG-008;D_6__metagenome;D_7__;D_8__;D_9__;D_10__;D_11__;D_12__;D_13__;D_14__	0.08(0.00,5.02)	0.77(0.02,32.96)	9.58(0.56,164.60)	1.06(0.02,47.60)	0.09(0.00,1.56)										
ASV_711	Firmicutes;D_2__Clostridia;D_3__Clostridiales;D_4__Lachnospiraceae;D_5__uncultured organism;D_6__;D_7__;D_8__;D_9__;D_10__;D_11__;D_12__;D_13__;D_14__						0.75(0.01,41.77)	4.08(0.09,187.30)	5.46(0.34,88.46)	0.70(0.02,31.96)	0.52(0.03,8.63)					
ASV_712	Bacteroidetes;D_2__Bacteroidia;D_3__Bacteroidales;D_4__Prevotellaceae;D_5__Prevotella;D_6__Prevotella disiens JCM 6334 = ATCC 29426;D_7__;D_8__;D_9__;D_10__;D_11__;D_12__;D_13__;D_14__	0.02(0.00,4.74)	0.01(0.00,2.01)	0.61(0.01,34.64)	13.03(0.08,2139.98)	0.24(0.00,12.46)										
ASV_713	Firmicutes;D_2__Clostridia;D_3__Clostridiales;D_4__Lachnospiraceae;D_5__Tyzzerella;D_6__uncultured bacterium;D_7__;D_8__;D_9__;D_10__;D_11__;D_12__;D_13__;D_14__	0.31(0.01,11.40)	0.95(0.03,26.17)	3.06(0.27,35.21)	1.67(0.06,48.46)	0.52(0.04,6.40)										
ASV_714	Firmicutes;D_2__Clostridia;D_3__Clostridiales;D_4__Lachnospiraceae;D_5__Lachnoclostridium	0.63(0.01,31.33)	0.45(0.01,17.22)	0.70(0.05,10.01)	0.94(0.02,36.89)	0.60(0.04,8.62)										
ASV_717	Firmicutes;D_2__Clostridia;D_3__Clostridiales;D_4__Family XI;D_5__Peptoniphilus;D_6__uncultured organism;D_7__;D_8__;D_9__;D_10__;D_11__;D_12__;D_13__;D_14__	1.46(0.03,66.75)	0.12(0.00,4.53)	0.08(0.01,1.10)	58.30(1.16,2939.45);p=0.04209	85.07(4.27,1694.97);p=0.00361	1.58(0.03,72.91)	0.12(0.00,5.12)	0.08(0.01,1.10)	55.89(1.04,2992.42);p=0.04759	88.05(4.33,1791.56);p=0.00358				↑	↑
ASV_721	Actinobacteria;D_2__Actinobacteria;D_3__Actinomycetales;D_4__Actinomycetaceae;D_5__Actinomyces	9.57(0.72,126.57)	2.70(0.24,31.04)	0.28(0.05,1.45)	0.04(0.00,0.48);p=0.011	0.40(0.07,2.09)										

ASV_722	Firmicutes;D_2__Erysipelotrichia;D_3__Erysipelotrichales;D_4__Erysipelotrichaceae;D_5__Erysipelatoclostridium;D_6__Massiliomicrobiota timonensis;D_7__;D_8__;D_9__;D_10__;D_11__;D_12__;D_13__;D_14__	2.90(0.17,48.12)	0.85(0.06,12.08)	0.29(0.05,1.87)	0.65(0.04,9.39)	1.88(0.29,12.35)										
ASV_723	Firmicutes;D_2__Clostridia;D_3__Clostridiales;D_4__Ruminococcaceae;D_5__Ruminiclostridium;D_6__Massilioclostridium coli;D_7__;D_8__;D_9__;D_10__;D_11__;D_12__;D_13__;D_14__	0.52(0.03,10.25)	0.77(0.05,12.32)	1.49(0.20,11.22)	0.65(0.04,10.52)	0.33(0.04,2.58)	0.69(0.04,13.33)	0.83(0.05,14.26)	1.20(0.15,9.36)	0.64(0.04,10.79)	0.44(0.06,3.41)					
ASV_726	Firmicutes;D_2__Negativicutes;D_3__Selenomonadales;D_4__Veillonellaceae;D_5__Veillonella	2.99(0.07,136.87)	1.92(0.05,71.32)	0.64(0.06,7.22)	0.07(0.00,2.62)	0.21(0.02,2.38)	2.04(0.05,91.93)	1.27(0.03,52.20)	0.63(0.05,7.73)	0.06(0.00,2.27)	0.12(0.01,1.40)					
ASV_733	Firmicutes;D_2__Clostridia;D_3__Clostridiales;D_4__Clostridiaceae 1;D_5__Clostridium sensu stricto 1	215529323793.92(0.00,Inf)	46025138861.76(0.00,Inf)	0.21(0.01,5.55)	0.00(0.00,Inf)	6.71(0.24,188.96)	20334843695529276.00(0.00,Inf)	8414117975005874.00(0.00,Inf)	0.41(0.01,11.72)	0.00(0.00,Inf)	7.59(0.27,216.27)					
ASV_734	Firmicutes;D_2__Clostridia;D_3__Clostridiales;D_4__Lachnospiraceae;D_5__Eisenbergiella;D_6__uncultured organism;D_7__;D_8__;D_9__;D_10__;D_11__;D_12__;D_13__;D_14__	0.41(0.05,3.27)	0.33(0.05,2.29)	0.80(0.20,3.17)	7.64(1.07,54.51);p=0.04251	3.17(0.77,13.09)	0.36(0.05,2.75)	0.30(0.04,2.18)	0.85(0.21,3.46)	8.08(1.13,57.96);p=0.03775	2.87(0.70,11.88)				↑	
ASV_737	Firmicutes;D_2__Clostridia;D_3__Clostridiales;D_4__Lachnospiraceae;D_5__Tyzzerella	45.50(0.26,8007.67)	40.59(0.32,5194.15)	0.89(0.03,25.95)	0.40(0.00,54.44)	18.11(0.57,579.26)										
ASV_738	Firmicutes;D_2__Clostridia;D_3__Clostridiales;D_4__Ruminococcaceae;D_5__Ruminiclostridium 9;D_6__uncultured bacterium;D_7__;D_8__;D_9__;D_10__;D_11__;D_12__;D_13__;D_14__	0.19(0.00,21.52)	0.61(0.01,44.62)	3.28(0.12,90.64)	0.08(0.00,5.64)	0.01(0.00,0.39);p=0.01219	0.02(0.00,3.68)	0.09(0.00,6.29)	4.20(0.08,228.22)	0.44(0.01,25.43)	0.01(0.00,0.49);p=0.02044					↓
ASV_740	Firmicutes;D_2__Clostridia;D_3__Clostridiales;D_4__Lachnospiraceae;D_5__Lachnospiraceae UCG-004;D_6__uncultured organism;D_7__;D_8__;D_9__;D_10__;D_11__;D_12__;D_13__;D_14__	1.67(0.06,47.78)	4.34(0.19,100.08)	2.60(0.29,23.62)	0.35(0.01,8.33)	0.58(0.06,5.58)	1.42(0.06,35.68)	6.25(0.28,139.65)	4.39(0.49,39.30)	0.17(0.01,3.80)	0.25(0.03,2.21)					
ASV_741	Firmicutes;D_2__Clostridia;D_3__Clostridiales;D_4__Lachnospiraceae;D_5__Lachnoclostridium;D_6__uncultured organism;D_7__;D_8__;D_9__;D_10__;D_11__;D_12__;D_13__;D_14__	5550487483.45(0.00,Inf)	1316717997.32(0.00,Inf)	0.24(0.02,3.08)	0.00(0.00,Inf)	43.05(1.72,1079.93);p=0.02211	16047871852583042.00(0.00,Inf)	4536792258326757.00(0.00,Inf)	0.28(0.02,3.53)	0.00(0.00,Inf)	23.68(1.18,477.25);p=0.03888					↑
ASV_743	Firmicutes;D_2__Clostridia;D_3__Clostridiales;D_4__Ruminococcaceae;D_5__Ruminococcaceae UCG-005;D_6__metagenome;D_7__;D_8__;D_9__;D_10__;D_11__;D_12__;D_13__;D_14__	0.16(0.00,9.00)	1.92(0.05,79.12)	12.14(0.79,185.88)	0.59(0.01,25.53)	0.09(0.01,1.52)	0.17(0.00,9.40)	2.48(0.06,103.64)	14.25(0.76,268.32)	0.08(0.00,3.37)	0.01(0.00,0.27);p=0.00431					↓
ASV_747	Firmicutes;D_2__Clostridia;D_3__Clostridiales;D_4__Lachnospiraceae;D_5__Sellimonas;D_6__uncultured bacterium;D_7__;D_8__;D_9__;D_10__;D_11__;D_12__;D_13__;D_14__	0.52(0.06,4.55)	0.60(0.08,4.57)	1.15(0.27,4.85)	10.38(1.33,80.99);p=0.02565	5.42(1.24,23.68);p=0.0247										
ASV_749	Firmicutes;D_2__Erysipelotrichia;D_3__Erysipelotrichales;D_4__Erysipelotrichaceae;D_5__Candidatus Stoquefichus	0.04(0.00,1.34)	0.40(0.02,9.80)	9.29(0.93,92.79)	18.70(0.72,482.68)	0.80(0.08,8.60)	0.05(0.00,1.38)	0.14(0.01,3.32)	2.75(0.28,26.89)	32.36(1.34,778.66);p=0.03216	1.62(0.16,16.33)				↑	
ASV_752	Firmicutes;D_2__Clostridia;D_3__Clostridiales;D_4__Ruminococcaceae;D_5__Negativibacillus;D_6__uncultured bacterium;D_7__;D_8__;D_9__;D_10__;D_11__;D_12__;D_13__;D_14__	0.14(0.00,10.90)	0.21(0.00,12.43)	1.52(0.08,27.38)	1.90(0.03,115.63)	0.27(0.01,5.18)	0.90(0.02,52.60)	0.20(0.00,10.50)	0.23(0.01,3.75)	2.03(0.04,100.62)	1.83(0.11,29.87)					
ASV_753	Firmicutes;D_2__Clostridia;D_3__Clostridiales;D_4__Ruminococcaceae;D_5__Oscillibacter	0.00(0.00,Inf)	0.52(0.01,52.91)	106288560033.93(0.00,Inf)	0.65(0.01,61.48)	0.00(0.00,Inf)										
ASV_757	Firmicutes;D_2__Erysipelotrichia;D_3__Erysipelotrichales;D_4__Erysipelotrichaceae;D_5__Faecalitalea;D_6__[Eubacterium] dolichum;D_7__;D_8__;D_9__;D_10__;D_11__;D_12__;D_13__;D_14__	0.10(0.00,5.44)	0.02(0.00,1.10)	0.25(0.02,4.06)	82.35(1.61,4223.90);p=0.02811	8.08(0.41,157.59)										

ASV_760	Firmicutes;D_2__Clostridia;D_3__Clostridiales;D_4__Family XIII;D_5__[Eubacterium] nodatum group	36046657065.65(0.00,Inf)	1.00(0.00,Inf)	0.00(0.00,Inf)	0.00(0.00,Inf)	18.66(0.95,364.73)										
ASV_761	Firmicutes;D_2__Erysipelotrichia;D_3__Erysipelotrichales;D_4__Erysipelotrichaceae;D_5__Merdibacter;D_6__uncultured bacterium;D_7__;D_8__;D_9__;D_10__;D_11__;D_12__;D_13__;D_14__	0.00(0.00,Inf)	0.13(0.00,5.76)	182879718648.24(0.00,Inf)	2.76(0.06,120.26)	0.00(0.00,Inf)										
ASV_767	Firmicutes;D_2__Erysipelotrichia;D_3__Erysipelotrichales;D_4__Erysipelotrichaceae;D_5__[Clostridium] innocuum group	17.30(0.19,1577.08)	14.94(0.21,1042.15)	0.86(0.05,15.53)	0.03(0.00,2.56)	0.60(0.03,11.64)										
ASV_768	Firmicutes;D_2__Clostridia;D_3__Clostridiales;D_4__Lachnospiraceae						1.10(0.00,Inf)	1114066471127471.00(0.00,Inf)	1009380157066080.00(0.00,Inf)	0.00(0.00,Inf)	0.00(0.00,Inf)					
ASV_773	Firmicutes;D_2__Clostridia;D_3__Clostridiales;D_4__Ruminococcaceae;D_5__Ruminiclostridium	4.07(0.17,97.65)	0.98(0.05,21.20)	0.24(0.03,1.83)	0.83(0.04,18.34)	3.39(0.43,26.46)	3.25(0.14,74.14)	0.78(0.03,17.46)	0.24(0.03,1.91)	1.17(0.05,25.77)	3.81(0.48,30.33)					
ASV_778	Bacteroidetes;D_2__Bacteroidia;D_3__Bacteroidales;D_4__Bacteroidaceae;D_5__Bacteroides	1.12(0.00,Inf)	504518315416.39(0.00,Inf)	451895577731.94(0.00,Inf)	0.00(0.00,Inf)	0.00(0.00,Inf)										
ASV_779	Firmicutes;D_2__Clostridia;D_3__Clostridiales;D_4__Lachnospiraceae;D_5__Lachnoclostridium	0.19(0.01,5.28)	0.81(0.04,16.93)	4.15(0.44,38.91)	2.68(0.12,59.18)	0.52(0.05,5.21)										
ASV_780	Actinobacteria;D_2__Coriobacteriia;D_3__Coriobacteriales;D_4__Eggerthellaceae;D_5__Gordonibacter;D_6__unidentified;D_7__;D_8__;D_9__;D_10__;D_11__;D_12__;D_13__;D_14__	1124066519072.93(0.00,Inf)	209666900919.48(0.00,Inf)	0.19(0.01,5.70)	0.00(0.00,Inf)	48.22(1.24,1879.66);p=0.0381										
ASV_792	Firmicutes;D_2__Clostridia;D_3__Clostridiales;D_4__Lachnospiraceae;D_5__Lachnospiraceae UCG-001;D_6__Lachnospiraceae bacterium TF01-11;D_7__;D_8__;D_9__;D_10__;D_11__;D_12__;D_13__;D_14__	0.52(0.01,50.33)	3.76(0.05,272.26)	7.26(0.35,150.51)	2.68(0.04,204.90)	1.39(0.06,31.11)	0.42(0.00,40.59)	12.04(0.15,988.45)	28.59(1.26,651.16);p=0.03551	2.20(0.03,176.49)	0.93(0.04,21.32)			↑		
ASV_794	Firmicutes;D_2__Clostridia;D_3__Clostridiales;D_4__Lachnospiraceae;D_5__[Ruminococcus] torques group	196481431967.02(0.00,Inf)	29472089120.45(0.00,Inf)	0.15(0.01,1.82)	0.00(0.00,Inf)	4.60(0.37,56.75)										
ASV_798	Actinobacteria;D_2__Coriobacteriia;D_3__Coriobacteriales;D_4__Eggerthellaceae;D_5__Eggerthella;D_6__uncultured bacterium;D_7__;D_8__;D_9__;D_10__;D_11__;D_12__;D_13__;D_14__	2.80(0.06,141.51)	5.05(0.13,199.41)	1.81(0.14,23.59)	0.75(0.02,31.24)	2.10(0.15,29.57)	0.44(0.01,15.92)	2.60(0.08,81.57)	5.89(0.45,76.79)	2.10(0.07,65.76)	0.93(0.07,12.23)					
ASV_804	Firmicutes;D_2__Clostridia;D_3__Clostridiales;D_4__Lachnospiraceae;D_5__Lachnospiraceae NK4A136 group;D_6__uncultured Clostridium sp.;D_7__;D_8__;D_9__;D_10__;D_11__;D_12__;D_13__;D_14__	5144062462.62(0.00,Inf)	13557562805.87(0.00,Inf)	2.64(0.48,14.62)	0.00(0.00,Inf)	0.66(0.11,3.84)	108718998377951.00(0.00,Inf)	286981687667415.00(0.00,Inf)	2.64(0.45,15.56)	0.00(0.00,Inf)	0.63(0.11,3.76)					
ASV_810	Firmicutes;D_2__Clostridia;D_3__Clostridiales;D_4__Lachnospiraceae;D_5__Marvinbryantia	0.62(0.01,52.43)	0.45(0.01,28.63)	0.72(0.04,13.84)	1.01(0.02,67.93)	0.63(0.03,12.80)										
ASV_811	Firmicutes;D_2__Clostridia;D_3__Clostridiales;D_4__Ruminococcaceae;D_5__Ruminococcaceae UCG-010;D_6__uncultured bacterium;D_7__;D_8__;D_9__;D_10__;D_11__;D_12__;D_13__;D_14__	1.12(0.00,Inf)	43292849151.18(0.00,Inf)	38776945216.37(0.00,Inf)	0.00(0.00,Inf)	0.00(0.00,Inf)										
ASV_	Firmicutes;D_2__Erysipelotrichia;D_3__Erysipelotrichales;D_4__Erysipelotrichaceae;D_5__[Clostridium] innocuum group						0.14(0.01,1.46)	0.15(0.02,1.47)	1.10(0.21,5.65)	14.07(1.46,135.69);p=0.02222	1.95(0.37,10.19)				↑	

812																
ASV_814	Firmicutes;D_2__Clostridia;D_3__Clostridiales;D_4__Peptococcaceae;D_5__uncultured	0.04(0.00, 2.62)	0.33(0.01, 13.50)	7.46(0.44, 125.82)	5.17(0.12, 226.60)	0.23(0.01, 4.08)										
ASV_817	Firmicutes;D_2__Clostridia;D_3__Clostridiales;D_4__Ruminococcaceae	1.33(0.11, 16.20)	1.36(0.13, 14.12)	1.02(0.20, 5.26)	0.51(0.05, 5.37)	0.68(0.13, 3.60)	1.02(0.08, 12.30)	1.21(0.11, 13.29)	1.19(0.22,6.42)	0.62(0.06, 6.72)	0.64(0.12,3.43)					
ASV_819	Firmicutes;D_2__Clostridia;D_3__Clostridiales;D_4__Ruminococcaceae	0.00(0.00, Inf)	0.34(0.01, 8.60)	1971529667.63(0.00,Inf)	0.72(0.03, 17.65)	0.00(0.00, Inf)										
ASV_820	Firmicutes;D_2__Clostridia;D_3__Clostridiales;D_4__Lachnospiraceae;D_5__GCA-900066575;D_6__uncultured bacterium;D_7__;D_8__;D_9__;D_10__;D_11__;D_12__;D_13__;D_14__	0.91(0.02, 38.54)	0.84(0.03, 27.90)	0.92(0.08, 11.17)	0.91(0.03, 31.44)	0.83(0.07, 10.64)										
ASV_822	Firmicutes;D_2__Clostridia;D_3__Clostridiales;D_4__Family XIII;D_5__Family XIII AD3011 group	115170491236.04(0.00,Inf)	89949432152.84(0.00,Inf)	0.78(0.04, 16.88)	0.00(0.00, Inf)	1.81(0.08, 43.24)										
ASV_826	Firmicutes;D_2__Clostridia;D_3__Clostridiales;D_4__Christensenellaceae;D_5__Christensenellaceae R-7 group	0.88(0.08, 10.10)	0.87(0.09, 8.50)	0.99(0.19, 5.04)	0.56(0.06, 5.57)	0.49(0.10, 2.58)	1.07(0.11, 10.74)	0.99(0.11, 9.25)	0.93(0.18,4.69)	0.56(0.06, 5.09)	0.60(0.12,2.94)					
ASV_827	Firmicutes;D_2__Clostridia;D_3__Clostridiales;D_4__Ruminococcaceae;D_5__Caproiciproducens;D_6__uncultured bacterium;D_7__;D_8__;D_9__;D_10__;D_11__;D_12__;D_13__;D_14__	81760177896.94(0.00,Inf)	46576866800.90(0.00,Inf)	0.57(0.01, 31.75)	0.00(0.00, Inf)	0.08(0.00, 3.79)										
ASV_828	Firmicutes;D_2__Clostridia;D_3__Clostridiales;D_4__Family XIII;D_5__Family XIII AD3011 group	0.49(0.01, 36.98)	0.53(0.01, 29.18)	1.06(0.06, 19.84)	0.82(0.01, 46.71)	0.41(0.02, 7.84)	0.37(0.01, 27.57)	0.57(0.01, 34.96)	1.52(0.07,32.04)	0.91(0.02, 53.48)	0.34(0.02,6.97)					
ASV_830	Firmicutes;D_2__Clostridia;D_3__Clostridiales;D_4__Eubacteriaceae;D_5__Anaerofustis;D_6__unidentified;D_7__;D_8__;D_9__;D_10__;D_11__;D_12__;D_13__;D_14__	4744552825.46(0.00,Inf)	2137912974.30(0.00,Inf)	0.45(0.08, 2.51)	0.00(0.00, Inf)	5.48(0.91, 33.22)	14067156364005460.00(0.00,Inf)	2989680494542553.00(0.00,Inf)	0.21(0.04,1.16)	0.00(0.00, Inf)	11.88(2.01, 70.08);p=0.00629					↑
ASV_831	Firmicutes;D_2__Clostridia;D_3__Clostridiales;D_4__Lachnospiraceae;D_5__GCA-900066755;D_6__uncultured bacterium;D_7__;D_8__;D_9__;D_10__;D_11__;D_12__;D_13__;D_14__	0.34(0.01, 20.75)	1.19(0.03, 51.42)	3.51(0.21, 57.43)	1.23(0.03, 56.04)	0.42(0.02, 7.33)										
ASV_832	Firmicutes;D_2__Clostridia;D_3__Clostridiales	0.34(0.01, 18.71)	1.30(0.04, 47.41)	3.84(0.24, 61.71)	0.55(0.01, 20.62)	0.19(0.01, 3.10)	0.37(0.01, 21.70)	1.66(0.04, 71.23)	4.46(0.24,81.84)	0.42(0.01, 17.16)	0.16(0.01,2.78)					
ASV_836	Firmicutes;D_2__Clostridia;D_3__Clostridiales;D_4__Peptococcaceae;D_5__uncultured	0.00(0.00, Inf)	0.71(0.01, 48.43)	4809739019.40(0.00,Inf)	4.17(0.05, 352.12)	0.00(0.00, Inf)										
ASV_838	Firmicutes;D_2__Clostridia;D_3__Clostridiales;D_4__Christensenellaceae;D_5__uncultured;D_6__uncultured bacterium;D_7__;D_8__;D_9__;D_10__;D_11__;D_12__;D_13__;D_14__	1.18(0.04, 35.70)	0.47(0.02, 12.09)	0.40(0.04, 4.08)	1.18(0.05, 30.16)	1.39(0.14, 14.20)										
ASV_839	Firmicutes;D_2__Clostridia;D_3__Clostridiales;D_4__Ruminococcaceae;D_5__Ruminococcaceae UCG-005	116649323419.46(0.00,Inf)	44915356063.62(0.00,Inf)	0.39(0.03, 5.05)	0.00(0.00, Inf)	1.88(0.14, 25.48)	8432343957195731.00(0.00,Inf)	2315688329793266.00(0.00,Inf)	0.27(0.02,4.03)	0.00(0.00, Inf)	1.65(0.13,21.05)					
ASV_	Firmicutes;D_2__Clostridia;D_3__Clostridiales;D_4__Christensenellaceae;D_5__Catabacter;D_6__Christensenella	0.00(0.00, Inf)	0.18(0.00, 28.63)	588400942.72(0.00, Inf)	0.66(0.00, 95.76)	0.00(0.00, Inf)										

843	timonensis;D_7__;D_8__;D_9__;D_10__;D_11__;D_12__;D_13__;D_14__															
ASV_845	Firmicutes;D_2__Clostridia;D_3__Clostridiales;D_4__Ruminococcaceae;D_5__Harryflintia	1.72(0.03,87.20)	0.79(0.02,32.86)	0.46(0.03,6.13)	1.03(0.02,44.32)	1.77(0.13,24.70)										
ASV_846	Firmicutes;D_2__Clostridia;D_3__Clostridiales;D_4__Ruminococcaceae;D_5__Candidatus Soleaferrea;D_6__uncultured bacterium;D_7__;D_8__;D_9__;D_10__;D_11__;D_12__;D_13__;D_14__						0.00(0.00,Inf)	1.25(0.04,35.51)	13968398414431.80(0.00,Inf)	1.57(0.06,44.51)	0.00(0.00,Inf)					
ASV_849	Firmicutes;D_2__Clostridia;D_3__Clostridiales;D_4__Clostridiales vadinBB60 group;D_5__uncultured organism;D_6__;D_7__;D_8__;D_9__;D_10__;D_11__;D_12__;D_13__;D_14__	0.41(0.02,7.18)	0.44(0.03,6.42)	1.08(0.16,7.32)	2.05(0.14,30.81)	0.84(0.12,5.96)	0.41(0.02,7.09)	0.53(0.03,8.35)	1.31(0.18,9.37)	1.62(0.11,24.90)	0.66(0.09,4.76)					
ASV_850	Firmicutes;D_2__Clostridia;D_3__Clostridiales;D_4__Christensenellaceae;D_5__Christensenella;D_6__Christensenella minuta;D_7__;D_8__;D_9__;D_10__;D_11__;D_12__;D_13__;D_14__	4.47(0.22,90.79)	1.29(0.08,22.04)	0.29(0.04,2.06)	0.55(0.03,9.63)	2.45(0.33,18.27)	4.37(0.22,87.18)	1.72(0.09,31.70)	0.39(0.05,2.98)	0.24(0.01,4.33)	1.06(0.14,7.87)					
ASV_851	Firmicutes;D_2__Clostridia;D_3__Clostridiales;D_4__Ruminococcaceae						1.12(0.00,Inf)	2694771603864426.00(0.00,Inf)	2524732902389718.00(0.00,Inf)	0.00(0.00,Inf)	0.00(0.00,Inf)					
ASV_855	Firmicutes;D_2__Clostridia;D_3__Clostridiales;D_4__Family XIII;D_5__[Eubacterium] nodatum group	0.24(0.01,9.71)	1.01(0.03,29.76)	4.26(0.34,53.76)	2.15(0.07,67.30)	0.51(0.04,7.05)										
ASV_863	Firmicutes;D_2__Clostridia;D_3__Clostridiales;D_4__Ruminococcaceae;D_5__Ruminococcus 1						0.19(0.01,6.50)	0.13(0.00,4.01)	0.70(0.06,7.77)	1.16(0.04,34.19)	0.22(0.02,2.47)					
ASV_869	Firmicutes;D_2__Clostridia;D_3__Clostridiales;D_4__Lachnospiraceae;D_5__Coprococcus 2	0.14(0.00,8.35)	0.86(0.02,39.71)	6.19(0.41,93.73)	1.25(0.03,60.28)	0.17(0.01,2.80)	0.14(0.00,7.61)	0.06(0.00,2.94)	0.44(0.03,6.74)	5.10(0.11,232.55)	0.72(0.05,11.08)					
ASV_908	Bacteroidetes;D_2__Bacteroidia;D_3__Bacteroidales;D_4__Bacteroidaceae;D_5__Bacteroides	104.79(0.56,19678.09)	312.98(2.25,43532.08);p=0.02249	2.99(0.11,80.29)	0.00(0.00,0.21);p=0.00989	0.15(0.01,4.29)										
ASV_920	Firmicutes;D_2__Clostridia;D_3__Clostridiales;D_4__Christensenellaceae;D_5__uncultured;D_6__uncultured bacterium;D_7__;D_8__;D_9__;D_10__;D_11__;D_12__;D_13__;D_14__	0.03(0.00,2.77)	0.06(0.00,3.64)	1.84(0.09,37.18)	65.77(0.99,4350.03)	2.27(0.09,55.53)	0.04(0.00,2.22)	0.04(0.00,2.40)	1.27(0.06,27.94)	192.80(2.75,13537.25);p=0.01528	6.77(0.22,210.45)				↑	
ASV_922	Firmicutes;D_2__Clostridia;D_3__Clostridiales;D_4__Ruminococcaceae;D_5__Angelakisella;D_6__uncultured bacterium;D_7__;D_8__;D_9__;D_10__;D_11__;D_12__;D_13__;D_14__	0.55(0.03,10.43)	0.53(0.03,8.30)	0.96(0.14,6.75)	3.54(0.22,57.15)	1.96(0.26,14.53)	0.63(0.04,11.21)	0.92(0.06,14.74)	1.45(0.20,10.58)	2.09(0.13,33.15)	1.32(0.18,9.62)					
ASV_925	Firmicutes;D_2__Clostridia;D_3__Clostridiales;D_4__Ruminococcaceae;D_5__Ruminococcaceae UCG-009	2516381170.84(0.00,Inf)	4014166795.83(0.00,Inf)	1.60(0.15,17.44)	0.00(0.00,Inf)	0.57(0.05,6.62)	10984212276750794.00(0.00,Inf)	13935663357457906.00(0.00,Inf)	1.27(0.11,14.76)	0.00(0.00,Inf)	0.15(0.01,1.73)					
ASV_927	Proteobacteria;D_2__Gammaproteobacteria;D_3__Betaproteobacteriales;D_4__Burkholderiaceae;D_5__Sutterella						1.21(0.02,76.90)	0.32(0.01,17.84)	0.27(0.02,4.55)	1.07(0.02,57.53)	1.30(0.08,22.21)					
ASV_931	Firmicutes;D_2__Clostridia;D_3__Clostridiales;D_4__Ruminococcaceae	0.03(0.00,1.52)	0.20(0.01,5.98)	7.37(0.36,149.37)	2.40(0.08,72.34)	0.07(0.00,1.38)	0.07(0.00,3.21)	0.69(0.02,20.73)	10.32(0.47,225.68)	0.90(0.03,25.75)	0.06(0.00,1.23)					
ASV_	Actinobacteria;D_2__Actinobacteria;D_3__Actinomycetales;D_4__Actinomycetaceae;D_5__Actinomyces	0.00(0.00,Inf)	0.26(0.00,14.27)	6378853348.21(0.00,Inf)	38.72(0.54,2793.42)	0.00(0.00,Inf)										

932																
ASV_937	Firmicutes;D_2__Clostridia;D_3__Clostridiales;D_4__Family XI;D_5__Parvimonas	0.61(0.05,7.55)	0.26(0.02,2.81)	0.43(0.08,2.36)	1.87(0.17,20.37)	1.14(0.20,6.37)	0.60(0.05,7.51)	0.30(0.03,3.49)	0.50(0.09,2.88)	1.72(0.15,19.25)	1.04(0.18,5.90)					
ASV_949	Firmicutes;D_2__Clostridia;D_3__Clostridiales;D_4__Family XI;D_5__Peptoniphilus;D_6__uncultured organism;D_7__;D_8__;D_9__;D_10__;D_11__;D_12__;D_13__;D_14__	0.03(0.00,1.15)	0.13(0.00,3.70)	4.57(0.34,60.87)	4.41(0.15,130.26)	0.13(0.01,1.75)										
ASV_951	Firmicutes;D_2__Clostridia;D_3__Clostridiales;D_4__Ruminococcaceae	509541275.84(0.00,Inf)	7161021011.87(0.00,Inf)	14.05(0.94,209.62)	0.00(0.00,Inf)	0.12(0.01,1.96)										
ASV_953	Firmicutes;D_2__Clostridia;D_3__Clostridiales;D_4__Ruminococcaceae;D_5__Subdoligranulum	3.20(0.16,64.12)	1.70(0.10,28.19)	0.53(0.07,3.87)	0.24(0.01,4.14)	0.77(0.10,5.91)	1.25(0.07,23.92)	0.99(0.06,17.15)	0.79(0.11,5.95)	0.62(0.04,10.50)	0.77(0.10,5.81)					
ASV_955	Bacteroidetes;D_2__Bacteroidia;D_3__Bacteroidales;D_4__Porphyromonadaceae;D_5__Porphyromonas	24.93(0.13,4856.38)	13.17(0.09,1884.70)	0.53(0.02,15.25)	0.41(0.00,63.18)	10.21(0.32,328.94)										
ASV_956	Firmicutes;D_2__Clostridia;D_3__Clostridiales;D_4__Family XI;D_5__Finegoldia;D_6__uncultured bacterium;D_7__;D_8__;D_9__;D_10__;D_11__;D_12__;D_13__;D_14__	0.06(0.00,2.20)	0.03(0.00,0.96);p=0.04752	0.55(0.05,6.01)	8.35(0.29,238.27)	0.53(0.05,6.01)	0.33(0.01,9.88)	0.08(0.00,2.14)	0.24(0.02,2.55)	7.64(0.29,202.16)	2.51(0.24,26.56)					
ASV_959	Firmicutes;D_2__Clostridia;D_3__Clostridiales;D_4__Eubacteriaceae;D_5__Anaerofustis;D_6__Anaerofustis sp. Marseille-P2441;D_7__;D_8__;D_9__;D_10__;D_11__;D_12__;D_13__;D_14__	2.08(0.08,53.59)	0.84(0.04,17.95)	0.41(0.05,3.48)	2.22(0.10,49.81)	4.61(0.50,42.69)										
ASV_964	Firmicutes;D_2__Clostridia;D_3__Clostridiales;D_4__Clostridiales vadinBB60 group;D_5__gut metagenome;D_6__;D_7__;D_8__;D_9__;D_10__;D_11__;D_12__;D_13__;D_14__	872152912.93(0.00,Inf)	2233701936.73(0.00,Inf)	2.56(0.29,22.45)	0.00(0.00,Inf)	0.10(0.01,0.92);p=0.04228	19049064044260480.00(0.00,Inf)	21822357929694496.00(0.00,Inf)	1.15(0.14,9.08)	0.00(0.00,Inf)	0.43(0.06,3.42)					
ASV_965	Firmicutes;D_2__Negativicutes;D_3__Selenomonadales;D_4__Veillonellaceae;D_5__Dialister;D_6__uncultured bacterium;D_7__;D_8__;D_9__;D_10__;D_11__;D_12__;D_13__;D_14__	24465228341.48(0.00,Inf)	6045422581.76(0.00,Inf)	0.25(0.01,6.28)	1.02(0.00,Inf)	24903176369.23(0.00,Inf)	75367273269274.41(0.00,Inf)	20384286239318.70(0.00,Inf)	0.27(0.01,9.27)	0.58(0.00,Inf)	44119931386517.80(0.00,Inf)					
ASV_966	Firmicutes;D_2__Clostridia;D_3__Clostridiales;D_4__Ruminococcaceae;D_5__Ruminococcaceae UCG-005;D_6__uncultured rumen bacterium;D_7__;D_8__;D_9__;D_10__;D_11__;D_12__;D_13__;D_14__	2.28(0.11,48.88)	4.15(0.23,73.32)	1.82(0.25,13.45)	0.46(0.03,8.51)	1.05(0.14,8.23)	2.02(0.10,40.86)	3.44(0.19,62.65)	1.70(0.22,12.97)	0.55(0.03,9.96)	1.12(0.15,8.58)					
ASV_969	Firmicutes;D_2__Clostridia;D_3__Clostridiales;D_4__Ruminococcaceae;D_5__Ruminococcaceae UCG-010	0.79(0.00,149.66)	2.48(0.02,332.32)	3.14(0.10,101.21)	0.53(0.00,75.35)	0.42(0.01,14.63)										
ASV_972	Firmicutes;D_2__Erysipelotrichia;D_3__Erysipelotrichales;D_4__Erysipelotrichaceae;D_5__Holdemania	1.12(0.00,Inf)	2235086040.17(0.00,Inf)	2001942284.65(0.00,Inf)	0.00(0.00,Inf)	0.00(0.00,Inf)										
ASV_981	Firmicutes;D_2__Clostridia;D_3__Clostridiales;D_4__Family XI;D_5__Anaerococcus	0.21(0.00,12.53)	0.21(0.00,9.36)	0.98(0.06,15.26)	1.52(0.03,71.21)	0.32(0.02,5.29)										
ASV_984	Firmicutes;D_2__Clostridia;D_3__Clostridiales;D_4__Lachnospiraceae;D_5__Marvinbryantia;D_6__uncultured bacterium;D_7__;D_8__;D_9__;D_10__;D_11__;D_12__;D_13__;D_14__	1.22(0.02,71.42)	0.34(0.01,15.20)	0.27(0.02,4.09)	2.01(0.04,95.39)	2.46(0.15,39.07)										
ASV_	Firmicutes;D_2__Clostridia;D_3__Clostridiales;D_4__Ruminococcaceae;D_5__Ruminiclostridium 5;D_6__uncultured	0.33(0.03,3.64)	0.81(0.09,7.29)	2.47(0.47,12.94)	1.05(0.11,9.60)	0.34(0.06,1.86)	0.33(0.03,3.66)	0.89(0.09,8.64)	2.72(0.49,15.17)	0.94(0.10,8.99)	0.31(0.06,1.70)					

986	bacterium;D_7__;D_8__;D_9__;D_10__;D_11__;D_12__;D_13__;D_14__															
ASV_1003	Firmicutes;D_2__Clostridia;D_3__Clostridiales;D_4__Ruminococcaceae;D_5__Fournierella;D_6__uncultured organism;D_7__;D_8__;D_9__;D_10__;D_11__;D_12__;D_13__;D_14__	0.38(0.00, 32.08)	1.22(0.02, 72.01)	3.19(0.16, 63.23)	0.52(0.01, 32.37)	0.20(0.01, 4.21)	0.42(0.00, 36.71)	0.87(0.01, 62.03)	2.07(0.09,48.49)	0.17(0.00, 11.33)	0.07(0.00,1.61)					
ASV_1005	Firmicutes;D_2__Clostridia;D_3__Clostridiales;D_4__Ruminococcaceae						1.00(0.00,Inf)	3425837981062057.00(0.00,Inf)	3433264678246714.00 (0.00,Inf)	0.00(0.00, Inf)	0.00(0.00,Inf)					
ASV_1009	Firmicutes;D_2__Clostridia;D_3__Clostridiales;D_4__Ruminococcaceae;D_5__GCA-900066225;D_6__uncultured bacterium;D_7__;D_8__;D_9__;D_10__;D_11__;D_12__;D_13__;D_14__	4.98(0.10, 258.89)	1.11(0.03, 47.55)	0.22(0.02, 2.88)	0.22(0.01, 9.68)	1.12(0.09, 14.56)	6.18(0.11, 356.82)	0.59(0.01, 33.84)	0.09(0.01,1.37)	0.16(0.00, 8.06)	0.99(0.08,12.16)					
ASV_1010	Firmicutes;D_2__Clostridia;D_3__Clostridiales;D_4__Ruminococcaceae;D_5__Ruminiclostridium 5;D_6__uncultured organism;D_7__;D_8__;D_9__;D_10__;D_11__;D_12__;D_13__;D_14__	0.04(0.00, 1.75)	0.06(0.00, 2.18)	1.67(0.12, 23.71)	25.93(0.69,970.98)	0.96(0.06, 14.86)										
ASV_1013	Actinobacteria;D_2__Actinobacteria;D_3__Corynebacteriales;D_4__Corynebacteriaceae;D_5__Corynebacterium;D_6__Corynebacterium durum;D_7__;D_8__;D_9__;D_10__;D_11__;D_12__;D_13__;D_14__	2.00(0.43, 9.29)	1.78(0.42, 7.54)	0.89(0.33, 2.39)	0.44(0.10, 1.89)	0.88(0.32, 2.41)	1.72(0.38, 7.70)	1.52(0.35, 6.51)	0.88(0.33,2.40)	0.55(0.13, 2.31)	0.94(0.34,2.55)					
ASV_1014	Firmicutes;D_2__Clostridia;D_3__Clostridiales;D_4__Lachnospiraceae;D_5__[Ruminococcus] gauvreauii group						0.16(0.00, 18.93)	0.01(0.00, 1.50)	0.09(0.00,2.56)	0.48(0.00, 46.58)	0.08(0.00,2.01)					
ASV_1016	Firmicutes;D_2__Clostridia;D_3__Clostridiales;D_4__Lachnospiraceae;D_5__Lachnoclostridium						1.65(0.00,Inf)	2374067042997492.00(0.00,Inf)	1386836819350152.00 (0.00,Inf)	0.00(0.00, Inf)	0.00(0.00,Inf)					
ASV_1018	Firmicutes;D_2__Clostridia;D_3__Clostridiales;D_4__Christensenellaceae;D_5__Christensenellaceae R-7 group;D_6__uncultured bacterium;D_7__;D_8__;D_9__;D_10__;D_11__;D_12__;D_13__;D_14__	0.25(0.01, 5.72)	0.41(0.02, 7.40)	1.67(0.19, 14.71)	2.20(0.12, 40.63)	0.55(0.06, 5.02)	0.24(0.01, 5.24)	0.66(0.04, 11.76)	2.70(0.27,26.67)	1.01(0.06, 17.44)	0.25(0.03,2.36)					
ASV_1020	Firmicutes;D_2__Erysipelotrichia;D_3__Erysipelotrichales;D_4__Erysipelotrichaceae;D_5__Erysipelatoclostridium;D_6__Massiliomicrobiota timonensis;D_7__;D_8__;D_9__;D_10__;D_11__;D_12__;D_13__;D_14__	0.64(0.02, 25.47)	1.39(0.04, 43.05)	2.16(0.19, 24.79)	2.15(0.07, 70.31)	1.39(0.11, 17.16)	0.67(0.02, 24.68)	2.40(0.07, 77.35)	3.60(0.30,43.28)	1.22(0.04, 38.66)	0.81(0.07,9.90)					
ASV_1023	Firmicutes;D_2__Clostridia;D_3__Clostridiales;D_4__Ruminococcaceae;D_5__Oscillospira;D_6__uncultured bacterium;D_7__;D_8__;D_9__;D_10__;D_11__;D_12__;D_13__;D_14__	1.12(0.00, Inf)	1956601082292.56(0.00,Inf)	1752356824657.34(0.00,Inf)	0.00(0.00, Inf)	0.00(0.00, Inf)										
ASV_1024	Firmicutes;D_2__Clostridia;D_3__Clostridiales;D_4__Lachnospiraceae	0.13(0.00, 4.13)	0.23(0.01, 5.48)	1.80(0.16, 20.53)	3.14(0.13, 76.25)	0.41(0.03, 4.84)										
ASV_1025	Firmicutes;D_2__Bacilli;D_3__Lactobacillales;D_4__Aerococcaceae;D_5__Abiotrophia;D_6__uncultured bacterium;D_7__;D_8__;D_9__;D_10__;D_11__;D_12__;D_13__;D_14__						789249264746557.00 (0.00,Inf)	194861448889186.00 (0.00,Inf)	0.25(0.03,2.05)	0.00(0.00, Inf)	4.43(0.52,37.44)					
ASV_1029	Firmicutes;D_2__Clostridia;D_3__Clostridiales;D_4__Ruminococcaceae;D_5__uncultured;D_6__uncultured Firmicutes bacterium;D_7__;D_8__;D_9__;D_10__;D_11__;D_12__;D_13__;D_14__	0.00(0.00, Inf)	1.05(0.04, 24.62)	194620358679.26(0.00,Inf)	1.74(0.07, 44.32)	0.00(0.00, Inf)	0.00(0.00,Inf)	0.80(0.03, 18.77)	2494206169029429.00 (0.00,Inf)	2.13(0.09, 50.45)	0.00(0.00,Inf)					
ASV_1030	Firmicutes;D_2__Clostridia;D_3__Clostridiales;D_4__Ruminococcaceae	2491028429.19(0.00,Inf)	1017566502.48(0.00,Inf)	0.41(0.06, 2.57)	0.00(0.00, Inf)	1.95(0.30, 12.77)										

ASV_1031	Actinobacteria;D_2__Actinobacteria;D_3__Bifidobacteriales;D_4__Bifidobacteriaceae;D_5__Scardovia;D_6__unidentified;D_7__;D_8__;D_9__;D_10__;D_11__;D_12__;D_13__;D_14__	0.36(0.01, 18.22)	0.14(0.00, 5.47)	0.37(0.02, 5.60)	2.00(0.05, 79.61)	0.73(0.05, 10.81)										
ASV_1033	Firmicutes;D_2__Clostridia;D_3__Clostridiales;D_4__Lachnospiraceae	1.14(0.00, Inf)	311055464408061.00(0.00,Inf)	264522269670455.00(0.00,Inf)	0.00(0.00, Inf)	0.00(0.00, Inf)										
ASV_1040	Firmicutes;D_2__Bacilli;D_3__Lactobacillales;D_4__Lactobacillaceae;D_5__Lactobacillus	0.16(0.00, 59.96)	0.66(0.00, 173.22)	4.26(0.08, 222.86)	0.16(0.00, 45.72)	0.03(0.00, 1.46)										
ASV_1049	Firmicutes;D_2__Bacilli;D_3__Lactobacillales;D_4__Lactobacillaceae;D_5__Lactobacillus;D_6__Lactobacillus rhamnosus;D_7__;D_8__;D_9__;D_10__;D_11__;D_12__;D_13__;D_14__	0.12(0.00, 7.65)	4.37(0.10, 186.42)	35.18(2.12,583.24);p=0.01295	11.69(0.24,577.30)	1.45(0.07, 29.22)	0.16(0.00, 9.41)	3.99(0.08, 189.43)	25.60(1.46, 450.16);p=0.02662	11.07(0.21,583.75)	1.72(0.08,35.83)			↑		
ASV_1052	Firmicutes;D_2__Clostridia;D_3__Clostridiales;D_4__Ruminococcaceae;D_5__Anaerofilum;D_6__uncultured bacterium;D_7__;D_8__;D_9__;D_10__;D_11__;D_12__;D_13__;D_14__	1.15(0.09, 14.32)	0.44(0.04, 4.68)	0.38(0.07, 2.05)	1.24(0.11, 13.61)	1.43(0.26, 7.95)	1.01(0.08, 12.24)	0.47(0.04, 5.32)	0.47(0.08,2.61)	1.53(0.14, 16.85)	1.56(0.28,8.62)					
ASV_1055	Firmicutes;D_2__Clostridia;D_3__Clostridiales;D_4__Ruminococcaceae;D_5__GCA-900066225;D_6__uncultured bacterium;D_7__;D_8__;D_9__;D_10__;D_11__;D_12__;D_13__;D_14__	4.53(0.06, 367.24)	1.20(0.02, 78.25)	0.27(0.02, 4.52)	0.02(0.00, 1.60)	0.11(0.01, 1.89)										
ASV_1057	Actinobacteria;D_2__Coriobacteriia;D_3__Coriobacteriales;D_4__Coriobacteriaceae;D_5__Collinsella;D_6__uncultured bacterium;D_7__;D_8__;D_9__;D_10__;D_11__;D_12__;D_13__;D_14__	0.56(0.06, 5.24)	0.82(0.10, 6.52)	1.46(0.33, 6.52)	0.94(0.12, 7.65)	0.53(0.11, 2.44)	0.45(0.05, 4.10)	0.96(0.12, 7.99)	2.16(0.46,10.11)	1.02(0.13, 8.36)	0.46(0.10,2.14)					
ASV_1061	Firmicutes;D_2__Clostridia;D_3__Clostridiales;D_4__Ruminococcaceae;D_5__uncultured;D_6__uncultured Clostridium sp.;D_7__;D_8__;D_9__;D_10__;D_11__;D_12__;D_13__;D_14__	0.00(0.00, Inf)	0.08(0.00, 19.90)	60858183789.06(0.00,Inf)	2.69(0.01, 676.32)	0.00(0.00, Inf)										
ASV_1062	Firmicutes;D_2__Clostridia;D_3__Clostridiales;D_4__Ruminococcaceae;D_5__GCA-900066225;D_6__uncultured Ruminococcus sp.;D_7__;D_8__;D_9__;D_10__;D_11__;D_12__;D_13__;D_14__	1.94(0.21, 18.24)	1.87(0.23, 15.39)	0.97(0.23, 4.07)	0.69(0.08, 5.84)	1.34(0.30, 5.89)	1.81(0.19, 17.02)	1.72(0.20, 15.00)	0.95(0.21,4.18)	0.74(0.09, 6.46)	1.35(0.30,6.04)					
ASV_1063	Firmicutes;D_2__Clostridia;D_3__Clostridiales;D_4__Ruminococcaceae;D_5__Ruminiclostridium 5;D_6__uncultured bacterium;D_7__;D_8__;D_9__;D_10__;D_11__;D_12__;D_13__;D_14__	0.14(0.00, 15.68)	0.15(0.00, 11.64)	1.11(0.04, 34.30)	1.85(0.03, 136.07)	0.26(0.01, 7.63)										
ASV_1066	Actinobacteria;D_2__Actinobacteria;D_3__Corynebacteriales;D_4__Corynebacteriaceae;D_5__Corynebacterium 1;D_6__Corynebacterium pseudodiphtheriticum;D_7__;D_8__;D_9__;D_10__;D_11__;D_12__;D_13__;D_14__	202670572591.18(0.00,Inf)	194978172753.13(0.00,Inf)	0.96(0.01, 74.50)	0.00(0.00, Inf)	1.38(0.02, 124.13)										
ASV_1067	Firmicutes;D_2__Clostridia;D_3__Clostridiales;D_4__Ruminococcaceae;D_5__Ruminococcaceae UCG-005;D_6__uncultured Clostridiales bacterium;D_7__;D_8__;D_9__;D_10__;D_11__;D_12__;D_13__;D_14__	249169507287.03(0.00,Inf)	926514993328.74(0.00,Inf)	3.72(0.05, 264.78)	0.00(0.00, Inf)	1.39(0.02, 122.24)	2306072612233243.00(0.00,Inf)	5260802233726419.00(0.00,Inf)	2.28(0.05,111.77)	0.00(0.00, Inf)	0.27(0.01,11.58)					
ASV_1070	Firmicutes;D_2__Clostridia;D_3__Clostridiales;D_4__Lachnospiraceae;D_5__Lactonifactor	11092691790.65(0.00,Inf)	3390203310.03(0.00,Inf)	0.31(0.02, 4.13)	0.00(0.00, Inf)	2.76(0.19, 39.40)										
ASV_1072	Firmicutes;D_2__Clostridia;D_3__Clostridiales;D_4__Lachnospiraceae;D_5__Lachnoclostridium	0.32(0.01, 9.51)	2.84(0.13, 63.59)	8.90(0.90, 87.81)	0.64(0.03, 14.95)	0.20(0.02, 2.13)	0.35(0.01, 9.47)	1.75(0.08, 40.36)	5.04(0.50,50.86)	0.64(0.03, 14.43)	0.22(0.02,2.22)					
ASV_	Firmicutes;D_2__Clostridia;D_3__Clostridiales;D_4__Lachnospiraceae;D_5__[Eubacterium] hallii group	1.12(0.00, Inf)	2120322231135.28(0.00,Inf)	1899206062347.06(0.00,Inf)	0.00(0.00, Inf)	0.00(0.00, Inf)	0.99(0.00,Inf)	731482503430519.00(0.00,Inf)	695532931052864.00(0.00,Inf)	0.00(0.00, Inf)	0.00(0.00,Inf)					

1091																
ASV_1092	Firmicutes;D_2__Clostridia;D_3__Clostridiales;D_4__Lachnospiraceae;D_5__[Eubacterium] hallii group	70960379848.57(0.00,Inf)	413750393808.38(0.00,Inf)	5.83(0.19,180.54)	0.00(0.00,Inf)	3.56(0.07,192.37)										
ASV_1096	Firmicutes;D_2__Clostridia;D_3__Clostridiales;D_4__Lachnospiraceae;D_5__Lachnospiraceae UCG-004	79324463600.83(0.00,Inf)	29698506803.43(0.00,Inf)	0.37(0.02,8.32)	0.00(0.00,Inf)	0.43(0.02,9.05)	3399463982248148.00(0.00,Inf)	277511408844481.00(0.00,Inf)	0.08(0.00,2.50)	0.00(0.00,Inf)	0.52(0.03,10.92)					
ASV_1097	Firmicutes;D_2__Clostridia;D_3__Clostridiales;D_4__Lachnospiraceae	1.12(0.00,Inf)	11257310919573.80(0.00,Inf)	10076375702502.80(0.00,Inf)	0.00(0.00,Inf)	0.00(0.00,Inf)										
ASV_1106	Firmicutes;D_2__Clostridia;D_3__Clostridiales;D_4__Ruminococcaceae;D_5__Faecalibacterium;D_6__uncultured bacterium;D_7__;D_8__;D_9__;D_10__;D_11__;D_12__;D_13__;D_14__	595475010054030.00(0.00,Inf)	0.97(0.00,Inf)	0.00(0.00,Inf)	0.00(0.00,Inf)	0.84(0.02,28.89)										
ASV_1108	Firmicutes;D_2__Clostridia;D_3__Clostridiales;D_4__Lachnospiraceae;D_5__Lachnospiraceae UCG-004	140926517720720.00(0.00,Inf)	148332177764739.00(0.00,Inf)	1.05(0.02,50.58)	1.02(0.00,Inf)	142724308796918.00(0.00,Inf)										
ASV_1112	Firmicutes;D_2__Clostridia;D_3__Clostridiales;D_4__Lachnospiraceae;D_5__Anaerostipes;D_6__uncultured bacterium;D_7__;D_8__;D_9__;D_10__;D_11__;D_12__;D_13__;D_14__	634037573320.22(0.00,Inf)	106856490775.01(0.00,Inf)	0.17(0.01,3.23)	0.00(0.00,Inf)	2.20(0.18,27.29)										
ASV_1132	Firmicutes;D_2__Bacilli;D_3__Lactobacillales;D_4__Streptococcaceae;D_5__Streptococcus	0.02(0.00,1.60)	0.00(0.00,0.17);p=0.00495	0.12(0.01,2.58)	55.80(1.08,2871.87);p=0.04549	1.39(0.08,25.75)	0.07(0.00,4.16)	0.00(0.00,0.08);p=0.00265	0.01(0.00,0.44);p=0.01671	50.95(0.94,2755.47)	3.53(0.19,65.60)		↓	↓		
ASV_1133	Firmicutes;D_2__Clostridia;D_3__Clostridiales;D_4__Family XI;D_5__Parvimonas	3085224598.54(0.00,Inf)	531114425.61(0.00,Inf)	0.17(0.01,2.81)	0.00(0.00,Inf)	0.50(0.03,7.52)	7588010475028923.00(0.00,Inf)	563099339208904.00(0.00,Inf)	0.07(0.00,1.42)	0.00(0.00,Inf)	0.87(0.06,12.92)					
ASV_1143	Firmicutes;D_2__Erysipelotrichia;D_3__Erysipelotrichales;D_4__Erysipelotrichaceae;D_5__Coprobacillus;D_6__uncultured bacterium;D_7__;D_8__;D_9__;D_10__;D_11__;D_12__;D_13__;D_14__	0.06(0.00,0.89);p=0.04079	0.31(0.03,3.75)	5.29(0.82,34.07)	0.84(0.07,10.51)	0.05(0.01,0.32);p=0.00177	0.07(0.00,1.01)	0.37(0.03,4.91)	5.61(0.83,37.98)	0.86(0.07,11.13)	0.06(0.01,0.39);p=0.00332					↓
ASV_1145	Bacteroidetes;D_2__Bacteroidia;D_3__Bacteroidales;D_4__Porphyromonadaceae;D_5__Porphyromonas;D_6__unidentified;D_7__;D_8__;D_9__;D_10__;D_11__;D_12__;D_13__;D_14__						1.14(0.00,Inf)	1679219760473545.00(0.00,Inf)	1509107551731771.00(0.00,Inf)	0.00(0.00,Inf)	0.00(0.00,Inf)					
ASV_1147	Firmicutes;D_2__Bacilli;D_3__Lactobacillales;D_4__Streptococcaceae;D_5__Streptococcus	9476185406.21(0.00,Inf)	2710501372.13(0.00,Inf)	0.29(0.02,3.31)	0.00(0.00,Inf)	4.43(0.36,54.69)										
ASV_1149	Firmicutes;D_2__Clostridia;D_3__Clostridiales;D_4__Ruminococcaceae	3296109245.53(0.00,Inf)	1334009835.52(0.00,Inf)	0.40(0.01,16.23)	0.00(0.00,Inf)	0.59(0.01,23.12)										
ASV_1151	Firmicutes;D_2__Negativicutes;D_3__Selenomonadales;D_4__Veillonellaceae;D_5__Dialister;D_6__uncultured bacterium;D_7__;D_8__;D_9__;D_10__;D_11__;D_12__;D_13__;D_14__	602345040.03(0.00,Inf)	3948099568.81(0.00,Inf)	6.55(0.07,656.00)	0.00(0.00,Inf)	1.43(0.01,215.32)										
ASV_1155	Actinobacteria;D_2__Actinobacteria;D_3__Corynebacteriales;D_4__Corynebacteriaceae;D_5__Lawsonella;D_6__uncultured bacterium;D_7__;D_8__;D_9__;D_10__;D_11__;D_12__;D_13__;D_14__	663925206.79(0.00,Inf)	434383728.40(0.00,Inf)	0.65(0.04,11.89)	0.00(0.00,Inf)	0.26(0.02,4.33)	1427053721939521.00(0.00,Inf)	995144900309256.00(0.00,Inf)	0.70(0.03,14.78)	0.00(0.00,Inf)	0.20(0.01,3.54)					
ASV_	Firmicutes;D_2__Clostridia;D_3__Clostridiales;D_4__Ruminococcaceae;D_5__Candidatus Soleaferrea;D_6__uncultured	0.43(0.02,9.19)	0.43(0.03,7.35)	1.00(0.12,8.27)	2.07(0.12,36.32)	0.90(0.10,7.75)	0.35(0.02,6.98)	0.28(0.02,5.03)	0.81(0.09,7.08)	2.34(0.14,40.15)	0.81(0.09,6.94)					

1160	bacterium;D_7__;D_8__;D_9__;D_10__;D_11__;D_12__;D_13__;D_14__															
ASV_1162	Firmicutes;D_2__Clostridia;D_3__Clostridiales;D_4__Ruminococcaceae;D_5__[Eubacterium] coprostanoligenes group;D_6__uncultured organism;D_7__;D_8__;D_9__;D_10__;D_11__;D_12__;D_13__;D_14__	0.31(0.00,40.14)	0.17(0.00,16.07)	0.54(0.02,16.65)	1.27(0.01,117.69)	0.40(0.01,11.65)										
ASV_1164	Actinobacteria;D_2__Actinobacteria;D_3__Actinomycetales;D_4__Actinomycetaceae;D_5__Actinomyces	0.26(0.01,12.10)	0.04(0.00,1.68)	0.16(0.01,2.52)	3.30(0.09,124.18)	0.86(0.06,12.21)	0.19(0.00,8.40)	0.04(0.00,1.52)	0.19(0.01,3.00)	6.63(0.18,247.65)	1.28(0.09,18.18)					
ASV_1173	Firmicutes;D_2__Clostridia;D_3__Clostridiales;D_4__Lachnospiraceae;D_5__Roseburia;D_6__uncultured bacterium;D_7__;D_8__;D_9__;D_10__;D_11__;D_12__;D_13__;D_14__						27.02(0.09,8420.61)	9.87(0.04,2597.02)	0.37(0.01,10.26)	0.38(0.00,101.80)	10.20(0.34,308.46)					
ASV_1189	Firmicutes;D_2__Clostridia;D_3__Clostridiales;D_4__Ruminococcaceae	37898272881.31(0.00,Inf)	77002409383.51(0.00,Inf)	2.03(0.09,44.12)	0.00(0.00,Inf)	0.24(0.01,5.02)	598933210731999.00(0.00,Inf)	1559086823352269.00(0.00,Inf)	2.60(0.10,68.97)	0.00(0.00,Inf)	0.18(0.01,4.37)					
ASV_1193	Firmicutes;D_2__Clostridia;D_3__Clostridiales;D_4__Ruminococcaceae;D_5__Ruminococcaceae UCG-004;D_6__uncultured bacterium;D_7__;D_8__;D_9__;D_10__;D_11__;D_12__;D_13__;D_14__	1.42(0.00,504.24)	0.90(0.00,222.88)	0.63(0.01,30.81)	2.10(0.01,576.28)	2.99(0.05,168.42)										
ASV_1196	Firmicutes;D_2__Clostridia;D_3__Clostridiales;D_4__Ruminococcaceae;D_5__uncultured						0.53(0.01,24.83)	0.22(0.01,9.36)	0.41(0.02,6.72)	1.12(0.03,43.45)	0.60(0.04,8.75)					
ASV_1201	Cyanobacteria;D_2__Oxyphotobacteria;D_3__Chloroplast	0.00(0.00,Inf)	3.58(0.03,469.66)	114764385299.40(0.00,Inf)	6.43(0.05,904.48)	0.00(0.00,Inf)										
ASV_1205	Firmicutes;D_2__Clostridia;D_3__Clostridiales;D_4__Ruminococcaceae	0.00(0.00,Inf)	0.04(0.00,2.31)	768012689.11(0.00,Inf)	12.31(0.24,624.28)	0.00(0.00,Inf)										
ASV_1208	Firmicutes;D_2__Erysipelotrichia;D_3__Erysipelotrichales;D_4__Erysipelotrichaceae;D_5__[Clostridium] innocuum group	1.12(0.00,Inf)	157374346885.24(0.00,Inf)	140960719658.42(0.00,Inf)	0.00(0.00,Inf)	0.00(0.00,Inf)	0.90(0.00,Inf)	1529532467049454.00(0.00,Inf)	1647600323072498.00(0.00,Inf)	0.00(0.00,Inf)	0.00(0.00,Inf)					
ASV_1209	Firmicutes;D_2__Clostridia;D_3__Clostridiales;D_4__Ruminococcaceae;D_5__Papillibacter;D_6__uncultured bacterium;D_7__;D_8__;D_9__;D_10__;D_11__;D_12__;D_13__;D_14__	0.57(0.06,5.03)	0.33(0.04,2.56)	0.58(0.13,2.66)	1.33(0.17,10.35)	0.75(0.17,3.44)	0.63(0.07,5.62)	0.36(0.04,2.99)	0.56(0.12,2.72)	1.16(0.14,9.29)	0.73(0.16,3.38)					
ASV_1210	Firmicutes;D_2__Clostridia;D_3__Clostridiales;D_4__Family XIII;D_5__[Eubacterium] nodatum group;D_6__[Eubacterium] sulci;D_7__;D_8__;D_9__;D_10__;D_11__;D_12__;D_13__;D_14__	0.48(0.02,11.15)	1.35(0.08,23.56)	2.81(0.33,23.91)	0.58(0.03,10.46)	0.28(0.03,2.45)	0.48(0.02,10.97)	1.29(0.07,24.61)	2.70(0.30,24.22)	0.61(0.03,11.41)	0.29(0.03,2.62)					
ASV_1211	Firmicutes;D_2__Clostridia;D_3__Clostridiales;D_4__Ruminococcaceae;D_5__Candidatus Soleaferrea;D_6__uncultured bacterium;D_7__;D_8__;D_9__;D_10__;D_11__;D_12__;D_13__;D_14__	0.55(0.02,17.21)	0.33(0.01,8.37)	0.60(0.06,6.47)	2.88(0.11,76.23)	1.59(0.14,18.10)	0.62(0.02,17.22)	0.37(0.01,9.62)	0.60(0.05,7.21)	1.32(0.06,31.44)	0.82(0.08,9.01)					
ASV_1213	Firmicutes;D_2__Clostridia;D_3__Clostridiales;D_4__Ruminococcaceae;D_5__Butyricicoccus	0.48(0.00,65.89)	0.03(0.00,4.33)	0.06(0.00,2.83)	1609079797217.53(0.00,Inf)	772799952518.27(0.00,Inf)										
ASV_1214	Actinobacteria;D_2__Actinobacteria;D_3__Actinomycetales;D_4__Actinomycetaceae;D_5__Varibaculum	1949353805.50(0.00,Inf)	2641887751.89(0.00,Inf)	1.36(0.13,14.49)	0.00(0.00,Inf)	0.51(0.05,5.67)	3579568137202264.00(0.00,Inf)	6251059825772203.00(0.00,Inf)	1.75(0.15,19.72)	0.00(0.00,Inf)	0.27(0.02,2.94)					
ASV_	Firmicutes;D_2__Clostridia;D_3__Clostridiales;D_4__Lachnospiraceae;D_5__Lachnospiraceae FCS020 group;D_6__uncultured	0.99(0.01,113.94)	1.04(0.01,88.19)	1.06(0.05,24.59)	2.78(0.03,263.34)	2.75(0.10,75.16)	0.46(0.00,53.34)	0.98(0.01,91.31)	2.13(0.08,56.90)	3.79(0.04,375.68)	1.75(0.06,53.04)					

1215	bacterium;D_7__;D_8__;D_9__;D_10__;D_11__;D_12__;D_13__;D_14__															
ASV_1216	Firmicutes;D_2__Clostridia;D_3__Clostridiales;D_4__Ruminococcaceae	1.12(0.00, Inf)	28373097804.66(0.00,Inf)	25413459552.28(0.00,Inf)	0.00(0.00, Inf)	0.00(0.00, Inf)										
ASV_1217	Firmicutes;D_2__Clostridia;D_3__Clostridiales;D_4__Christensenellaceae;D_5__Christensenellaceae R-7 group	0.51(0.03, 7.85)	0.31(0.02, 4.10)	0.62(0.09, 4.30)	4.10(0.29, 57.20)	2.09(0.28, 15.83)	0.54(0.03, 8.42)	0.34(0.02, 4.90)	0.62(0.08,4.66)	3.88(0.26, 57.25)	2.09(0.27,16.22)					
ASV_1220	Bacteroidetes;D_2__Bacteroidia;D_3__Bacteroidales;D_4__Prevotellaceae;D_5__Prevotella;D_6__Chlamydia trachomatis;D_7__;D_8__;D_9__;D_10__;D_11__;D_12__;D_13__;D_14__	0.39(0.00, 45.51)	28.33(0.38,2086.43)	72.35(2.96,1771.01);p=0.00868	0.67(0.01, 53.06)	0.26(0.01, 7.09)										
ASV_1222	Firmicutes;D_2__Bacilli;D_3__Lactobacillales;D_4__Lactobacillaceae;D_5__Lactobacillus;D_6__Lactobacillus rhamnosus;D_7__;D_8__;D_9__;D_10__;D_11__;D_12__;D_13__;D_14__	1347713156.24(0.00,Inf)	2815667453.87(0.00,Inf)	2.09(0.07, 64.90)	0.00(0.00, Inf)	4.01(0.07, 217.75)	1172129938862041.00(0.00,Inf)	3781223101515184.00(0.00,Inf)	3.23(0.08,126.55)	0.00(0.00, Inf)	2.71(0.05,152.53)					
ASV_1223	Firmicutes;D_2__Clostridia;D_3__Clostridiales;D_4__Ruminococcaceae	3083497308.38(0.00,Inf)	2935734744.22(0.00,Inf)	0.95(0.04, 25.21)	0.00(0.00, Inf)	0.85(0.03, 24.12)										
ASV_1226	Firmicutes;D_2__Clostridia;D_3__Clostridiales;D_4__Christensenellaceae;D_5__Christensenellaceae R-7 group	0.61(0.01, 51.92)	0.26(0.00, 16.89)	0.42(0.02, 8.70)	0.28(0.00, 18.33)	0.17(0.01, 3.47)										
ASV_1227	Firmicutes;D_2__Clostridia;D_3__Clostridiales;D_4__Ruminococcaceae;D_5__Ruminiclostridium 5;D_6__uncultured bacterium;D_7__;D_8__;D_9__;D_10__;D_11__;D_12__;D_13__;D_14__	0.31(0.01, 7.14)	0.14(0.01, 2.80)	0.46(0.04, 4.92)	0.61(0.03, 10.71)	0.19(0.02, 1.71)										
ASV_1228	Firmicutes;D_2__Clostridia;D_3__Clostridiales;D_4__Christensenellaceae;D_5__uncultured;D_6__uncultured bacterium;D_7__;D_8__;D_9__;D_10__;D_11__;D_12__;D_13__;D_14__	0.34(0.01, 14.85)	1.26(0.04, 36.62)	3.72(0.27, 51.49)	0.48(0.02, 14.15)	0.16(0.01, 2.31)										
ASV_1229	Firmicutes;D_2__Clostridia;D_3__Clostridiales;D_4__Ruminococcaceae;D_5__uncultured bacterium;D_6__;D_7__;D_8__;D_9__;D_10__;D_11__;D_12__;D_13__;D_14__	0.00(0.00, Inf)	0.30(0.03, 3.20)	6147872310.01(0.00,Inf)	1.99(0.19, 20.75)	0.00(0.00, Inf)	0.00(0.00,Inf)	0.11(0.01, 1.28)	936135137468493.00(0.00,Inf)	3.61(0.36, 36.59)	0.00(0.00,Inf)					
ASV_1231	Firmicutes;D_2__Clostridia;D_3__Clostridiales;D_4__Lachnospiraceae	0.00(0.00, Inf)	0.02(0.00, 0.20);p=0.00058	4034752480.94(0.00,Inf)	193092653662.74(0.00,Inf)	1.14(0.00, Inf)										
ASV_1240	Firmicutes;D_2__Clostridia;D_3__Clostridiales;D_4__Ruminococcaceae;D_5__Ruminiclostridium 9;D_6__Colidextribacter massiliensis;D_7__;D_8__;D_9__;D_10__;D_11__;D_12__;D_13__;D_14__	0.03(0.00, 5.67)	0.18(0.00, 18.62)	6.14(0.14, 272.37)	0.75(0.01, 80.08)	0.02(0.00, 1.00)										
ASV_1258	Firmicutes;D_2__Clostridia;D_3__Clostridiales;D_4__Ruminococcaceae;D_5__Hydrogenoanaerobacterium;D_6__uncultured bacterium;D_7__;D_8__;D_9__;D_10__;D_11__;D_12__;D_13__;D_14__	4029212982.52(0.00,Inf)	4509430209.80(0.00,Inf)	1.12(0.07, 19.01)	0.00(0.00, Inf)	2.06(0.09, 44.61)	1579089380639486.00(0.00,Inf)	1107049222470294.00(0.00,Inf)	0.70(0.04,12.80)	0.00(0.00, Inf)	1.72(0.09,31.67)					
ASV_1259	Firmicutes;D_2__Clostridia;D_3__Clostridiales;D_4__Ruminococcaceae;D_5__GCA-900066225;D_6__uncultured bacterium;D_7__;D_8__;D_9__;D_10__;D_11__;D_12__;D_13__;D_14__	0.00(0.00, Inf)	0.12(0.00, 8.11)	72970113785.49(0.00,Inf)	7.19(0.11, 489.43)	0.00(0.00, Inf)										
ASV_1282	Firmicutes;D_2__Clostridia;D_3__Clostridiales;D_4__Lachnospiraceae	5.69(0.22, 148.68)	9.02(0.41, 196.82)	1.59(0.22, 11.43)	0.09(0.00, 1.99)	0.50(0.07, 3.80)	9.32(0.35, 247.70)	3.87(0.16, 95.05)	0.42(0.06,3.07)	0.09(0.00, 2.22)	0.87(0.12,6.33)					
ASV_	Firmicutes;D_2__Clostridia;D_3__Clostridiales;D_4__Family XIII;D_5__[Eubacterium] brachy group;D_6__Eubacterium brachy ATCC	1.33(0.05, 33.73)	0.29(0.01, 6.24)	0.22(0.02, 1.95)	0.76(0.04, 16.22)	1.01(0.11, 8.97)										

1284	33089;D_7__;D_8__;D_9__;D_10__;D_11__;D_12__;D_13__;D_14__															
ASV_1297	Firmicutes;D_2__Clostridia;D_3__Clostridiales;D_4__Peptococcaceae;D_5__Peptococcus;D_6__uncultured organism;D_7__;D_8__;D_9__;D_10__;D_11__;D_12__;D_13__;D_14__	1.12(0.00,Inf)	4387792882391.49(0.00,Inf)	3927123666487.38(0.00,Inf)	0.00(0.00,Inf)	0.00(0.00,Inf)										
ASV_1301	Firmicutes;D_2__Clostridia;D_3__Clostridiales;D_4__Ruminococcaceae;D_5__Candidatus Soleaferrea	2.10(0.03,134.56)	0.71(0.01,37.03)	0.34(0.02,5.33)	1.35(0.02,73.46)	2.83(0.17,47.58)										
ASV_1303	Firmicutes;D_2__Clostridia;D_3__Clostridiales;D_4__Ruminococcaceae;D_5__uncultured	0.11(0.01,2.15)	0.16(0.01,2.59)	1.51(0.19,11.75)	5.82(0.35,97.02)	0.62(0.08,5.09)	0.08(0.00,1.61)	0.21(0.01,3.44)	2.46(0.29,20.61)	6.08(0.37,98.65)	0.51(0.06,4.29)					
ASV_1306	Actinobacteria;D_2__Actinobacteria;D_3__Actinomycetales;D_4__Actinomycetaceae;D_5__F0332;D_6__uncultured bacterium;D_7__;D_8__;D_9__;D_10__;D_11__;D_12__;D_13__;D_14__	1.15(0.06,23.13)	3.68(0.23,58.01)	3.20(0.47,21.55)	0.28(0.02,4.48)	0.32(0.05,2.23)	2.10(0.12,37.97)	3.54(0.21,58.57)	1.69(0.27,10.44)	0.28(0.02,4.52)	0.58(0.09,3.61)					
ASV_1323	Firmicutes;D_2__Clostridia;D_3__Clostridiales;D_4__Lachnospiraceae;D_5__[Eubacterium] eligens group	1.89(0.01,522.59)	8.84(0.05,1707.80)	4.68(0.11,193.42)	0.03(0.00,6.58)	0.06(0.00,2.72)	2.20(0.01,434.27)	31.43(0.20,5056.41)	14.30(0.33,623.35)	0.01(0.00,1.02)	0.01(0.00,0.61);p=0.02611					↓
ASV_1329	Patescibacteria;D_2__Saccharimonadia;D_3__Saccharimonadales;D_4__Saccharimonadaceae;D_5__uncultured Candidatus Saccharibacteria bacterium;D_6__;D_7__;D_8__;D_9__;D_10__;D_11__;D_12__;D_13__;D_14__	3.00(0.17,52.20)	0.45(0.03,7.33)	0.15(0.02,1.04)	1.41(0.09,22.74)	4.22(0.61,29.04)	3.05(0.18,52.67)	0.50(0.03,8.82)	0.16(0.02,1.21)	1.26(0.08,20.95)	3.85(0.55,26.89)					
ASV_1333	Actinobacteria;D_2__Actinobacteria;D_3__Bifidobacteriales;D_4__Bifidobacteriaceae;D_5__Bifidobacterium	3617620076.24(0.00,Inf)	2059646383.46(0.00,Inf)	0.57(0.08,3.89)	0.00(0.00,Inf)	3.20(0.42,24.24)										
ASV_1336	Actinobacteria;D_2__Actinobacteria;D_3__Corynebacteriales;D_4__Corynebacteriaceae;D_5__Corynebacterium 1	0.43(0.00,51.07)	0.01(0.00,1.22)	0.02(0.00,0.89);p=0.04299	15.02(0.15,1474.48)	6.39(0.23,180.23)										
ASV_1343	Firmicutes;D_2__Clostridia;D_3__Clostridiales;D_4__Lachnospiraceae;D_5__Dorea	0.00(0.00,Inf)	0.05(0.00,1.33)	2050066459393.89(0.00,Inf)	49641247927239.70(0.00,Inf)	1.14(0.00,Inf)										
ASV_1361	Firmicutes;D_2__Clostridia;D_3__Clostridiales;D_4__Lachnospiraceae;D_5__Lachnoclostridium;D_6__Eubacterium sp. Marseille-P3202;D_7__;D_8__;D_9__;D_10__;D_11__;D_12__;D_13__;D_14__	0.69(0.01,85.13)	35.76(0.46,2805.70)	51.46(2.21,1197.10);p=0.01411	0.10(0.00,8.14)	0.07(0.00,1.71)										
ASV_1362	Actinobacteria;D_2__Actinobacteria;D_3__Micrococcales;D_4__Micrococcaceae;D_5__Rothia	1.17(0.03,48.02)	3.45(0.11,110.49)	2.96(0.26,33.93)	0.43(0.01,14.37)	0.50(0.04,6.12)										
ASV_1363	Firmicutes;D_2__Clostridia;D_3__Clostridiales;D_4__Christensenellaceae;D_5__Christensenellaceae R-7 group	0.03(0.00,1.69)	0.05(0.00,2.56)	2.04(0.12,34.93)	7.75(0.15,399.47)	0.20(0.01,3.56)										
ASV_1372	Firmicutes;D_2__Clostridia;D_3__Clostridiales;D_4__Ruminococcaceae;D_5__Subdoligranulum	0.00(0.00,Inf)	0.36(0.01,15.24)	6690023042.71(0.00,Inf)	3.60(0.08,169.08)	0.00(0.00,Inf)										
ASV_1378	Firmicutes;D_2__Erysipelotrichia;D_3__Erysipelotrichales;D_4__Erysipelotrichaceae;D_5__Merdibacter;D_6__uncultured bacterium;D_7__;D_8__;D_9__;D_10__;D_11__;D_12__;D_13__;D_14__	0.25(0.02,4.15)	0.48(0.04,6.50)	1.89(0.29,12.25)	1.34(0.10,18.71)	0.34(0.05,2.30)										

ASV_1391	Firmicutes;D_2__Clostridia;D_3__Clostridiales;D_4__Defluviitaleaceae;D_5__Defluviitaleaceae UCG-011;D_6__uncultured bacterium;D_7__;D_8__;D_9__;D_10__;D_11__;D_12__;D_13__;D_14__	0.00(0.00,Inf)	0.14(0.00,4.40)	143173880004.49(0.00,Inf)	1154999811688.15(0.00,Inf)	1.14(0.00,Inf)										
ASV_1393	Firmicutes;D_2__Clostridia;D_3__Clostridiales;D_4__Ruminococcaceae;D_5__Intestinimonas;D_6__uncultured Clostridiales bacterium;D_7__;D_8__;D_9__;D_10__;D_11__;D_12__;D_13__;D_14__						2411730226552677.00(0.00,Inf)	1991066820487674.00(0.00,Inf)	0.83(0.02,41.48)	0.00(0.00,Inf)	0.08(0.00,3.78)					
ASV_1399	Firmicutes;D_2__Clostridia;D_3__Clostridiales;D_4__Ruminococcaceae;D_5__GCA-900066225;D_6__uncultured bacterium;D_7__;D_8__;D_9__;D_10__;D_11__;D_12__;D_13__;D_14__	0.29(0.00,55.65)	0.37(0.00,49.54)	1.26(0.04,41.62)	2.58(0.02,371.35)	0.75(0.02,27.17)										
ASV_1408	Firmicutes;D_2__Clostridia;D_3__Clostridiales;D_4__Lachnospiraceae	78851617366.31(0.00,Inf)	94217903576.41(0.00,Inf)	1.19(0.08,18.95)	0.00(0.00,Inf)	1.17(0.07,20.24)										
ASV_1409	Firmicutes;D_2__Clostridia;D_3__Clostridiales;D_4__Ruminococcaceae	0.00(0.00,Inf)	0.20(0.00,11.41)	1345798853.62(0.00,Inf)	1.58(0.03,78.92)	0.00(0.00,Inf)										
ASV_1416	Firmicutes;D_2__Clostridia;D_3__Clostridiales;D_4__Ruminococcaceae;D_5__[Eubacterium] coprostanoligenes group;D_6__uncultured organism;D_7__;D_8__;D_9__;D_10__;D_11__;D_12__;D_13__;D_14__	51942940204.23(0.00,Inf)	87616785188.07(0.00,Inf)	1.69(0.10,27.47)	0.00(0.00,Inf)	0.37(0.02,6.48)	15485125928102630.00(0.00,Inf)	8443504549357148.00(0.00,Inf)	0.55(0.03,8.70)	0.00(0.00,Inf)	0.32(0.02,4.58)					
ASV_1418	Firmicutes;D_2__Clostridia;D_3__Clostridiales;D_4__Lachnospiraceae;D_5__Lachnospiraceae FCS020 group;D_6__metagenome;D_7__;D_8__;D_9__;D_10__;D_11__;D_12__;D_13__;D_14__	2169323006528.86(0.00,Inf)	8220399438490.44(0.00,Inf)	3.79(0.24,60.66)	0.00(0.00,Inf)	0.15(0.01,2.38)	711930576175573.00(0.00,Inf)	3725585276056270.00(0.00,Inf)	5.23(0.27,100.16)	0.00(0.00,Inf)	0.11(0.01,2.05)					
ASV_1420	Firmicutes;D_2__Clostridia;D_3__Clostridiales;D_4__Christensenellaceae;D_5__Christensenellaceae R-7 group;D_6__uncultured organism;D_7__;D_8__;D_9__;D_10__;D_11__;D_12__;D_13__;D_14__						1.48(0.00,Inf)	969767462232523.00(0.00,Inf)	764392257757924.00(0.00,Inf)	0.00(0.00,Inf)	0.00(0.00,Inf)					
ASV_1421	Firmicutes;D_2__Clostridia;D_3__Clostridiales;D_4__Ruminococcaceae;D_5__Butyricicoccus	10421305766.10(0.00,Inf)	5708121989.60(0.00,Inf)	0.55(0.01,50.43)	0.00(0.00,Inf)	0.67(0.01,61.02)										
ASV_1422	Firmicutes;D_2__Clostridia;D_3__Clostridiales;D_4__Lachnospiraceae;D_5__Lachnoclostridium	8.95(0.02,3494.68)	16.43(0.06,4686.13)	1.84(0.05,62.68)	0.05(0.00,13.74)	0.41(0.01,15.16)	18.35(0.11,3189.77)	3.54(0.02,606.23)	0.19(0.01,4.95)	0.45(0.00,77.35)	8.25(0.29,231.47)					
ASV_1464	Firmicutes;D_2__Clostridia;D_3__Clostridiales;D_4__Ruminococcaceae;D_5__Subdoligranulum;D_6__uncultured bacterium;D_7__;D_8__;D_9__;D_10__;D_11__;D_12__;D_13__;D_14__	3974198818495.86(0.00,Inf)	1.00(0.00,Inf)	0.00(0.00,Inf)	0.00(0.00,Inf)	0.77(0.06,10.12)	1042457155738006.00(0.00,Inf)	0.77(0.00,Inf)	0.00(0.00,Inf)	0.00(0.00,Inf)	0.79(0.06,10.72)					
ASV_1493	Firmicutes;D_2__Clostridia;D_3__Clostridiales;D_4__Ruminococcaceae;D_5__Oscillibacter;D_6__uncultured bacterium;D_7__;D_8__;D_9__;D_10__;D_11__;D_12__;D_13__;D_14__	690506727.54(0.00,Inf)	1020194412.42(0.00,Inf)	1.48(0.08,27.47)	0.00(0.00,Inf)	0.19(0.01,3.53)										
ASV_1503	Firmicutes;D_2__Clostridia;D_3__Clostridiales;D_4__Christensenellaceae;D_5__Christensenellaceae R-7 group	4272899746.18(0.00,Inf)	657562668.69(0.00,Inf)	0.15(0.00,8.95)	0.00(0.00,Inf)	1.09(0.02,56.77)										
ASV_1510	Epsilonbacteraeota;D_2__Campylobacteria;D_3__Campylobacterales;D_4__Campylobacteraceae;D_5__Campylobacter;D_6__unidentified;D_7__;D_8__;D_9__;D_10__;D_11__;D_12__;D_13__;D_14__	1.12(0.00,Inf)	6535709518.92(0.00,Inf)	5853966831.24(0.00,Inf)	0.00(0.00,Inf)	0.00(0.00,Inf)	0.99(0.00,Inf)	492960092331116.00(0.00,Inf)	488013251568278.00(0.00,Inf)	0.00(0.00,Inf)	0.00(0.00,Inf)					
ASV_1512	Firmicutes;D_2__Clostridia;D_3__Clostridiales;D_4__Christensenellaceae;D_5__Christensenellaceae R-7 group;D_6__Blattella germanica (German	0.14(0.00,11.36)	0.56(0.01,28.71)	3.92(0.18,84.13)	3.05(0.06,167.55)	0.44(0.02,10.30)										

	cockroach);D_7__;D_8__;D_9__;D_10__;D_11__;D_12__;D_13__;D_14__															
ASV_1520	Actinobacteria;D_2__Actinobacteria;D_3__Bifidobacteriales;D_4__Bifidobacteriaceae;D_5__Bifidobacterium	579197444290.70(0.00,Inf)	535211936699.96(0.00,Inf)	0.92(0.10,8.27)	0.00(0.00,Inf)	0.32(0.04,2.58)										
ASV_1526	Firmicutes;D_2__Clostridia;D_3__Clostridiales;D_4__Lachnospiraceae;D_5__uncultured;D_6__intestinal bacterium CG19-1;D_7__;D_8__;D_9__;D_10__;D_11__;D_12__;D_13__;D_14__	0.99(0.03,30.45)	0.70(0.03,17.37)	0.71(0.07,6.91)	1.94(0.07,50.25)	1.92(0.18,20.03)										
ASV_1528	Firmicutes;D_2__Clostridia;D_3__Clostridiales;D_4__Ruminococcaceae;D_5__Ruminiclostridium 9;D_6__Colidextribacter massiliensis;D_7__;D_8__;D_9__;D_10__;D_11__;D_12__;D_13__;D_14__	5.13(0.08,317.40)	1.00(0.02,50.83)	0.19(0.01,2.82)	0.20(0.00,10.26)	1.04(0.07,14.99)										
ASV_1532	Bacteroidetes;D_2__Bacteroidia;D_3__Bacteroidales;D_4__Bacteroidaceae;D_5__Bacteroides	1.12(0.00,Inf)	1007928792327.24(0.00,Inf)	902769970893.38(0.00,Inf)	0.00(0.00,Inf)	0.00(0.00,Inf)										
ASV_1541	Firmicutes;D_2__Clostridia;D_3__Clostridiales;D_4__Eubacteriaceae;D_5__Anaerofustis						0.19(0.00,9.07)	0.02(0.00,1.03)	0.11(0.01,2.16)	4.93(0.12,205.74)	0.92(0.06,14.12)					
ASV_1543	Proteobacteria;D_2__Gammaproteobacteria;D_3__Xanthomonadales;D_4__Xanthomonadaceae;D_5__Stenotrophomonas	2213541016106.22(0.00,Inf)	190473552677.06(0.00,Inf)	0.09(0.01,1.23)	0.00(0.00,Inf)	3.91(0.29,52.89)										
ASV_1548	Proteobacteria;D_2__Alphaproteobacteria;D_3__Rhizobiales;D_4__Rhizobiaceae;D_5__Allorhizobium-Neorhizobium-Pararhizobium-Rhizobium	2.28(0.15,35.23)	0.00(0.00,Inf)	0.00(0.00,Inf)	6.41(0.35,118.68)	14.62(1.61,133.02);p=0.01729	1.63(0.11,23.54)	0.00(0.00,Inf)	0.00(0.00,Inf)	14.25(0.67,302.62)	23.26(2.21,245.30);p=0.00885					↑
ASV_1552	Firmicutes;D_2__Clostridia;D_3__Clostridiales;D_4__Lachnospiraceae;D_5__Blautia						0.11(0.00,8.27)	10.22(0.17,614.38)	91.78(4.50,1873.83);p=0.00332	2.05(0.03,121.30)	0.23(0.01,4.72)			↑		
ASV_1556	Firmicutes;D_2__Clostridia;D_3__Clostridiales;D_4__Lachnospiraceae;D_5__Lachnoclostridium	6.02(0.14,256.76)	1.44(0.04,49.98)	0.24(0.02,2.72)	5.03(0.12,212.38)	30.26(2.01,455.14);p=0.0137	0.30(0.01,5.89)	0.08(0.00,1.65)	0.29(0.03,2.80)	15.69(0.78,314.09)	4.63(0.43,49.81)					
ASV_1559	Actinobacteria;D_2__Coriobacteriia;D_3__Coriobacteriales;D_4__Eggerthellaceae;D_5__Enterorhabdus;D_6__uncultured bacterium;D_7__;D_8__;D_9__;D_10__;D_11__;D_12__;D_13__;D_14__	65282753588.33(0.00,Inf)	4774472140.97(0.00,Inf)	0.07(0.00,2.31)	1.02(0.00,Inf)	66451343791.63(0.00,Inf)										
ASV_1569	Firmicutes;D_2__Bacilli;D_3__Lactobacillales;D_4__Leuconostocaceae;D_5__Leuconostoc;D_6__uncultured bacterium;D_7__;D_8__;D_9__;D_10__;D_11__;D_12__;D_13__;D_14__	23887933405.16(0.00,Inf)	13776143025.31(0.00,Inf)	0.58(0.03,11.93)	0.00(0.00,Inf)	0.06(0.00,1.26)	7029763243577099.00(0.00,Inf)	427838912362187.00(0.00,Inf)	0.06(0.00,1.45)	0.00(0.00,Inf)	0.51(0.03,7.40)					
ASV_1570	Bacteroidetes;D_2__Bacteroidia;D_3__Bacteroidales;D_4__Bacteroidaceae;D_5__Bacteroides	0.69(0.05,9.70)	0.88(0.08,9.85)	1.27(0.21,7.85)	573508300957.13(0.00,Inf)	395448226289.67(0.00,Inf)	0.72(0.05,9.32)	1.43(0.12,16.48)	2.00(0.29,13.90)	2516609275612619.00(0.00,Inf)	1801434608368288.00(0.00,Inf)					
ASV_1575	Firmicutes;D_2__Clostridia;D_3__Clostridiales;D_4__Lachnospiraceae;D_5__Blautia	1082937759790873.00(0.00,Inf)	635236879615347.00(0.00,Inf)	0.59(0.05,7.11)	0.99(0.00,Inf)	1004162898610419.00(0.00,Inf)	1214111885195059.00(0.00,Inf)	1392605926533321.00(0.00,Inf)	1.15(0.08,16.91)	0.62(0.00,Inf)	582329876989452.00(0.00,Inf)					
ASV_1576	Firmicutes;D_2__Clostridia;D_3__Clostridiales;D_4__Lachnospiraceae;D_5__Tyzzerella 4;D_6__uncultured organism;D_7__;D_8__;D_9__;D_10__;D_11__;D_12__;D_13__;D_14__	0.10(0.00,2.84)	0.24(0.01,5.35)	2.33(0.26,21.06)	4.39(0.19,102.09)	0.45(0.05,4.30)	0.10(0.00,2.65)	0.18(0.01,4.42)	1.87(0.19,18.14)	6.00(0.25,146.20)	0.57(0.06,5.57)					
ASV_	Firmicutes;D_2__Bacilli;D_3__Lactobacillales;D_4__Streptococcaceae;D_5__Streptococcus;D_6__Streptococcus	0.00(0.00,Inf)	0.82(0.02,39.39)	3060216031.78(0.00,Inf)	0.10(0.00,4.57)	0.00(0.00,Inf)	0.00(0.00,Inf)	0.62(0.01,29.99)	784271068867664.00(0.00,Inf)	0.09(0.00,3.35)	0.00(0.00,Inf)					

1585	agalactiae;D_7__;D_8__;D_9__;D_10__;D_11__;D_12__;D_13__;D_14__															
ASV_1591	Firmicutes;D_2__Clostridia;D_3__Clostridiales;D_4__Ruminococcaceae;D_5__Faecalibacterium						0.00(0.00,Inf)	5.21(0.08,355.82)	14182933547178444.00(0.00,Inf)	0.48(0.01,31.60)	0.00(0.00,Inf)					
ASV_1642	Firmicutes;D_2__Clostridia;D_3__Clostridiales;D_4__Family XI;D_5__Anaerococcus	0.03(0.00,6.48)	0.02(0.00,2.53)	0.54(0.01,27.66)	8.76(0.07,1172.19)	0.28(0.01,12.38)										
ASV_1657	Firmicutes;D_2__Clostridia;D_3__Clostridiales;D_4__Family XI;D_5__Peptoniphilus;D_6__Peptoniphilus sp. KHD5;D_7__;D_8__;D_9__;D_10__;D_11__;D_12__;D_13__;D_14__	0.00(0.00,Inf)	0.88(0.01,101.13)	2783891925.16(0.00,Inf)	0.66(0.01,79.47)	0.00(0.00,Inf)	0.00(0.00,Inf)	1.04(0.01,140.40)	4959959446596355.00(0.00,Inf)	0.73(0.01,93.50)	0.00(0.00,Inf)					
ASV_1662	Firmicutes;D_2__Clostridia;D_3__Clostridiales;D_4__Ruminococcaceae;D_5__Candidatus Soleaferrea;D_6__uncultured bacterium;D_7__;D_8__;D_9__;D_10__;D_11__;D_12__;D_13__;D_14__	0.34(0.00,23.22)	1.24(0.03,55.93)	3.67(0.20,67.85)	0.71(0.02,33.27)	0.24(0.01,4.66)										
ASV_1666	Firmicutes;D_2__Clostridia;D_3__Clostridiales;D_4__Ruminococcaceae;D_5__Ruminococcaceae UCG-014;D_6__uncultured bacterium;D_7__;D_8__;D_9__;D_10__;D_11__;D_12__;D_13__;D_14__	525605748570.55(0.00,Inf)	1891051208178.07(0.00,Inf)	3.60(0.08,162.10)	0.00(0.00,Inf)	2.63(0.04,177.47)										
ASV_1711	Firmicutes;D_2__Clostridia;D_3__Clostridiales;D_4__Ruminococcaceae;D_5__Ruminococcaceae UCG-013;D_6__uncultured bacterium;D_7__;D_8__;D_9__;D_10__;D_11__;D_12__;D_13__;D_14__	1.90(0.02,174.04)	0.42(0.01,29.99)	0.22(0.01,4.55)	6.96(0.09,560.69)	13.19(0.54,321.37)	9.48(0.09,985.91)	1.01(0.01,95.20)	0.11(0.00,2.35)	6.40(0.06,723.77)	60.69(2.05,1796.62);p=0.01754					↑
ASV_1714	Firmicutes;D_2__Clostridia;D_3__Clostridiales;D_4__Ruminococcaceae	3.64(0.17,78.21)	4.84(0.27,87.38)	1.33(0.21,8.53)	0.23(0.01,4.29)	0.84(0.12,5.64)	1.12(0.07,18.07)	3.98(0.28,57.27)	3.54(0.50,25.19)	0.28(0.02,3.87)	0.31(0.04,2.15)					
ASV_1719	Actinobacteria;D_2__Actinobacteria;D_3__Micrococcales;D_4__Micrococcaceae;D_5__Rothia;D_6__uncultured bacterium;D_7__;D_8__;D_9__;D_10__;D_11__;D_12__;D_13__;D_14__	0.41(0.03,4.96)	0.19(0.02,1.97)	0.45(0.07,2.74)	2.03(0.20,20.66)	0.84(0.14,4.88)										
ASV_1721	Actinobacteria;D_2__Coriobacteriia;D_3__Coriobacteriales;D_4__Atopobiaceae;D_5__Atopobium;D_6__uncultured bacterium;D_7__;D_8__;D_9__;D_10__;D_11__;D_12__;D_13__;D_14__	0.30(0.01,9.18)	0.21(0.01,5.24)	0.72(0.06,8.00)	0.96(0.04,23.01)	0.29(0.03,3.13)	0.28(0.01,9.09)	0.29(0.01,7.96)	1.01(0.08,12.95)	0.62(0.02,15.78)	0.17(0.01,2.05)					
ASV_1725	Firmicutes;D_2__Clostridia;D_3__Clostridiales;D_4__Ruminococcaceae	0.47(0.00,52.96)	0.92(0.01,75.16)	1.96(0.08,46.26)	7.61(0.08,715.20)	3.55(0.13,100.63)										
ASV_1739	Firmicutes;D_2__Clostridia;D_3__Clostridiales;D_4__Ruminococcaceae;D_5__Subdoligranulum;D_6__uncultured bacterium;D_7__;D_8__;D_9__;D_10__;D_11__;D_12__;D_13__;D_14__	0.05(0.00,1.48)	0.62(0.04,10.84)	12.90(1.00,167.07)	10.44(0.52,210.66)	0.50(0.03,7.62)	0.05(0.00,1.54)	0.72(0.04,14.24)	14.57(1.02,209.05);p=0.04864	10.31(0.48,221.80)	0.51(0.03,8.09)			↑		
ASV_1742	Firmicutes;D_2__Clostridia;D_3__Clostridiales;D_4__Ruminococcaceae;D_5__Subdoligranulum;D_6__uncultured bacterium;D_7__;D_8__;D_9__;D_10__;D_11__;D_12__;D_13__;D_14__	1.12(0.00,Inf)	408108477311.77(0.00,Inf)	365546993162.50(0.00,Inf)	0.00(0.00,Inf)	0.00(0.00,Inf)										
ASV_1755	Actinobacteria;D_2__Actinobacteria;D_3__Corynebacteriales;D_4__Corynebacteriaceae;D_5__Corynebacterium 1	295467983652.38(0.00,Inf)	76861905460.26(0.00,Inf)	0.26(0.02,4.24)	0.00(0.00,Inf)	3.82(0.22,67.29)										
ASV_1780	Firmicutes;D_2__Clostridia;D_3__Clostridiales;D_4__Ruminococcaceae;D_5__Anaerotruncus	0.00(0.00,Inf)	0.35(0.02,6.64)	281386572704.08(0.00,Inf)	3.77(0.18,78.14)	0.00(0.00,Inf)	0.00(0.00,Inf)	0.54(0.03,11.35)	991989497271004.00(0.00,Inf)	1.83(0.09,36.87)	0.00(0.00,Inf)					
ASV_	Actinobacteria;D_2__Coriobacteriia;D_3__Coriobacteriales;D_4__Coriobacteriaceae;D_5__Collinsella	0.00(0.00,Inf)	1.62(0.04,63.13)	325051920515.53(0.00,Inf)	1.16(0.03,48.54)	0.00(0.00,Inf)										

1811																
ASV_1813	Actinobacteria;D_2__Actinobacteria;D_3__Actinomycetales;D_4__Actinomycetaceae;D_5__Varibaculum	0.00(0.00,Inf)	1.64(0.03,86.91)	3571009306.33(0.00,Inf)	0.25(0.00,13.80)	0.00(0.00,Inf)										
ASV_1815	Firmicutes;D_2__Clostridia;D_3__Clostridiales;D_4__Lachnospiraceae;D_5__Lachnoclostridium	0.00(0.00,Inf)	0.23(0.01,6.24)	132692734222.29(0.00,Inf)	643485707717.52(0.00,Inf)	1.14(0.00,Inf)										
ASV_1816	Firmicutes;D_2__Clostridia;D_3__Clostridiales;D_4__Lachnospiraceae;D_5__Tyzzerella	0.00(0.00,Inf)	0.52(0.01,35.94)	522150647902.60(0.00,Inf)	2.72(0.04,208.84)	0.00(0.00,Inf)										
ASV_1817	Firmicutes;D_2__Clostridia;D_3__Clostridiales;D_4__Lachnospiraceae	1.12(0.00,Inf)	297666846801.84(0.00,Inf)	266615538236.99(0.00,Inf)	0.00(0.00,Inf)	0.00(0.00,Inf)										
ASV_1856	Proteobacteria;D_2__Alphaproteobacteria;D_3__Caulobacterales;D_4__Caulobacteraceae;D_5__PMMR1;D_6__uncultured bacterium;D_7__;D_8__;D_9__;D_10__;D_11__;D_12__;D_13__;D_14__	38672540886.53(0.00,Inf)	41423280804.78(0.00,Inf)	1.07(0.02,70.16)	0.00(0.00,Inf)	0.60(0.01,42.54)										
ASV_1863	Firmicutes;D_2__Clostridia;D_3__Clostridiales;D_4__Ruminococcaceae;D_5__Ruminiclostridium 5;D_6__uncultured bacterium;D_7__;D_8__;D_9__;D_10__;D_11__;D_12__;D_13__;D_14__	0.00(0.00,Inf)	1.08(0.04,31.38)	1665235910722.53(0.00,Inf)	0.57(0.02,16.37)	0.00(0.00,Inf)										
ASV_1872	Firmicutes;D_2__Clostridia;D_3__Clostridiales;D_4__Peptococcaceae;D_5__uncultured;D_6__uncultured organism;D_7__;D_8__;D_9__;D_10__;D_11__;D_12__;D_13__;D_14__	85488312495.69(0.00,Inf)	1351140977957.64(0.00,Inf)	15.80(0.18,1397.70)	0.00(0.00,Inf)	0.56(0.01,61.84)										
ASV_1875	Firmicutes;D_2__Clostridia;D_3__Clostridiales;D_4__Ruminococcaceae;D_5__Ruminococcaceae UCG-004;D_6__uncultured bacterium;D_7__;D_8__;D_9__;D_10__;D_11__;D_12__;D_13__;D_14__	1.12(0.00,Inf)	283268200956.63(0.00,Inf)	253722736938.96(0.00,Inf)	0.00(0.00,Inf)	0.00(0.00,Inf)										
ASV_1878	Firmicutes;D_2__Clostridia;D_3__Clostridiales;D_4__Ruminococcaceae;D_5__Subdoligranulum	1.12(0.00,Inf)	901663227176.07(0.00,Inf)	807854126968.07(0.00,Inf)	0.00(0.00,Inf)	0.00(0.00,Inf)										
ASV_1880	Actinobacteria;D_2__Coriobacteriia;D_3__Coriobacteriales;D_4__Coriobacteriaceae;D_5__Collinsella	277798569856.88(0.00,Inf)	641244828103.85(0.00,Inf)	2.31(0.03,178.75)	0.00(0.00,Inf)	0.82(0.01,75.89)										
ASV_1899	Firmicutes;D_2__Clostridia;D_3__Clostridiales;D_4__Lachnospiraceae;D_5__Tyzzerella;D_6__uncultured Firmicutes bacterium;D_7__;D_8__;D_9__;D_10__;D_11__;D_12__;D_13__;D_14__	0.00(0.00,Inf)	0.32(0.01,15.19)	6960085938.54(0.00,Inf)	11.47(0.20,671.03)	0.00(0.00,Inf)										
ASV_2017	Firmicutes;D_2__Clostridia;D_3__Clostridiales;D_4__Lachnospiraceae;D_5__Lachnospiraceae UCG-010;D_6__uncultured organism;D_7__;D_8__;D_9__;D_10__;D_11__;D_12__;D_13__;D_14__	73912802219904.41(0.00,Inf)	1.00(0.00,Inf)	0.00(0.00,Inf)	0.00(0.00,Inf)	7.05(0.13,377.99)										
ASV_2043	Firmicutes;D_2__Clostridia;D_3__Clostridiales;D_4__Lachnospiraceae;D_5__Lachnospiraceae NC2004 group;D_6__uncultured bacterium;D_7__;D_8__;D_9__;D_10__;D_11__;D_12__;D_13__;D_14__	1.12(0.00,Inf)	4961121076.24(0.00,Inf)	4443621473.18(0.00,Inf)	0.00(0.00,Inf)	0.00(0.00,Inf)	1.35(0.00,Inf)	2330667278440131.00(0.00,Inf)	1782941740766006.00(0.00,Inf)	0.00(0.00,Inf)	0.00(0.00,Inf)					
ASV_2044	Firmicutes;D_2__Clostridia;D_3__Clostridiales;D_4__Lachnospiraceae;D_5__Lachnospiraceae NC2004 group;D_6__uncultured bacterium;D_7__;D_8__;D_9__;D_10__;D_11__;D_12__;D_13__;D_14__	1389173353300475.00(0.00,Inf)	258304907652207.00(0.00,Inf)	0.19(0.01,4.14)	0.00(0.00,Inf)	4.85(0.21,111.50)	1868166154448276.00(0.00,Inf)	384600444532091.00(0.00,Inf)	0.21(0.01,7.18)	0.00(0.00,Inf)	3.24(0.12,85.58)					
ASV_	Firmicutes;D_2__Clostridia;D_3__Clostridiales;D_4__Lachnospiraceae;D_5__Agathobacter;D_6__uncultured						6100868402664472.00(0.00,Inf)	748294427677969.00(0.00,Inf)	0.12(0.01,1.75)	0.00(0.00,Inf)	3.61(0.31,41.36)					

2081	bacterium;D_7__;D_8__;D_9__;D_10__;D_11__;D_12__;D_13__;D_14__															
ASV_2103	Bacteroidetes;D_2__Bacteroidia;D_3__Bacteroidales;D_4__Bacteroidaceae;D_5__Bacteroides	5770999660964.94(0.00,Inf)	8541820135260.00(0.00,Inf)	1.48(0.08,28.02)	1.02(0.00,Inf)	5877315320960.07(0.00,Inf)	34314867149091.70(0.00,Inf)	31373513869642.80(0.00,Inf)	0.91(0.06,15.08)	0.95(0.00,Inf)	32687891041109.60(0.00,Inf)					
ASV_2105	Firmicutes;D_2__Clostridia;D_3__Clostridiales;D_4__Lachnospiraceae;D_5__CHKCI001;D_6__uncultured organism;D_7__;D_8__;D_9__;D_10__;D_11__;D_12__;D_13__;D_14__	0.95(0.02,56.59)	0.98(0.02,44.83)	1.03(0.07,15.59)	3.50(0.07,176.41)	3.32(0.19,57.26)										
ASV_2107	Firmicutes;D_2__Clostridia;D_3__Clostridiales;D_4__Lachnospiraceae	1.12(0.00,Inf)	633196114415.22(0.00,Inf)	567152802265.05(0.00,Inf)	0.00(0.00,Inf)	0.00(0.00,Inf)	1.39(0.00,Inf)	1715392155047103.00(0.00,Inf)	1190735456850250.00(0.00,Inf)	0.00(0.00,Inf)	0.00(0.00,Inf)					
ASV_2109	Firmicutes;D_2__Clostridia;D_3__Clostridiales;D_4__Ruminococcaceae;D_5__Butyricicoccus;D_6__uncultured bacterium;D_7__;D_8__;D_9__;D_10__;D_11__;D_12__;D_13__;D_14__	0.00(0.00,Inf)	0.02(0.00,1.99)	1388979847.79(0.00,Inf)	7.34(0.09,605.73)	0.00(0.00,Inf)										
ASV_2114	Firmicutes;D_2__Clostridia;D_3__Clostridiales;D_4__Eubacteriaceae;D_5__Anaerofustis;D_6__unidentified;D_7__;D_8__;D_9__;D_10__;D_11__;D_12__;D_13__;D_14__	3089589799.48(0.00,Inf)	6176350671.86(0.00,Inf)	2.00(0.07,60.85)	0.00(0.00,Inf)	1.17(0.03,40.38)										
ASV_2115	Firmicutes;D_2__Clostridia;D_3__Clostridiales;D_4__Lachnospiraceae;D_5__Blautia	35740494982.47(0.00,Inf)	313898383603.39(0.00,Inf)	8.78(0.13,608.05)	0.00(0.00,Inf)	0.70(0.01,61.43)										
ASV_2117	Bacteroidetes;D_2__Bacteroidia;D_3__Bacteroidales;D_4__Tannerellaceae;D_5__Parabacteroides	1.12(0.00,Inf)	27488049645.78(0.00,Inf)	24620766843.19(0.00,Inf)	0.00(0.00,Inf)	0.00(0.00,Inf)										
ASV_2134	Firmicutes;D_2__Clostridia;D_3__Clostridiales;D_4__Lachnospiraceae;D_5__Oribacterium;D_6__Oribacterium sinus;D_7__;D_8__;D_9__;D_10__;D_11__;D_12__;D_13__;D_14__	0.47(0.01,15.68)	0.82(0.03,19.46)	1.77(0.15,21.08)	0.88(0.04,21.14)	0.41(0.03,4.98)										
ASV_2169	Firmicutes;D_2__Clostridia;D_3__Clostridiales;D_4__Lachnospiraceae	0.00(0.00,Inf)	4.70(0.02,1209.39)	251941593674.13(0.00,Inf)	7.26(0.02,2675.97)	0.00(0.00,Inf)										
ASV_2185	Firmicutes;D_2__Clostridia;D_3__Clostridiales;D_4__Lachnospiraceae	2.28(0.01,411.60)	3.98(0.03,517.26)	1.75(0.06,48.78)	0.28(0.00,38.02)	0.63(0.02,19.15)										
ASV_2186	Firmicutes;D_2__Clostridia;D_3__Clostridiales;D_4__Lachnospiraceae;D_5__Blautia;D_6__Blautia sp. Marseille-P3087;D_7__;D_8__;D_9__;D_10__;D_11__;D_12__;D_13__;D_14__	870986288508184.00(0.00,Inf)	1.02(0.00,Inf)	0.00(0.00,Inf)	0.00(0.00,Inf)	2.46(0.11,54.00)										
ASV_2198	Firmicutes;D_2__Clostridia;D_3__Clostridiales;D_4__Ruminococcaceae;D_5__Ruminococcaceae UCG-010;D_6__uncultured organism;D_7__;D_8__;D_9__;D_10__;D_11__;D_12__;D_13__;D_14__						10484738253502874.00(0.00,Inf)	6000697458446384.00(0.00,Inf)	0.57(0.07,4.62)	0.00(0.00,Inf)	0.20(0.03,1.56)					
ASV_2218	Firmicutes;D_2__Clostridia;D_3__Clostridiales;D_4__Family XIII;D_5__[Eubacterium] nodatum group;D_6__Eubacterium infirmum F0142;D_7__;D_8__;D_9__;D_10__;D_11__;D_12__;D_13__;D_14__	0.00(0.00,Inf)	0.25(0.00,31.28)	103499254316.59(0.00,Inf)	0.87(0.01,99.05)	0.00(0.00,Inf)										
ASV_2234	Firmicutes;D_2__Clostridia;D_3__Clostridiales;D_4__Peptococcaceae;D_5__Peptococcus						1850006128246409.00(0.00,Inf)	9236161451580890.00(0.00,Inf)	4.99(0.09,270.90)	0.00(0.00,Inf)	0.27(0.01,13.83)					

ASV_2236	Firmicutes;D_2__Clostridia;D_3__Clostridiales;D_4__Lachnospiraceae;D_5__Lachnospiraceae NC2004 group;D_6__uncultured bacterium;D_7__;D_8__;D_9__;D_10__;D_11__;D_12__;D_13__;D_14__	3495843662527.58(0.00,Inf)	318934670620.09(0.00,Inf)	0.09(0.00,2.99)	1.02(0.00,Inf)	3558298062172.73(0.00,Inf)										
ASV_2240	Firmicutes;D_2__Clostridia;D_3__Clostridiales;D_4__Ruminococcaceae;D_5__Ruminiclostridium;D_6__metagenome;D_7__;D_8__;D_9__;D_10__;D_11__;D_12__;D_13__;D_14__	0.00(0.00,Inf)	0.76(0.03,17.84)	4733932751.16(0.00,Inf)	1.17(0.05,28.50)	0.00(0.00,Inf)	0.00(0.00,Inf)	0.70(0.03,19.05)	36187029428315.90(0.00,Inf)	1.21(0.05,31.38)	0.00(0.00,Inf)					
ASV_2241	Proteobacteria;D_2__Gammaproteobacteria;D_3__Betaproteobacteriales;D_4__Burkholderiaceae;D_5__Lautropia;D_6__uncultured bacterium;D_7__;D_8__;D_9__;D_10__;D_11__;D_12__;D_13__;D_14__	5754946616707916.00(0.00,Inf)	397762422814683.00(0.00,Inf)	0.07(0.00,4.65)	0.00(0.00,Inf)	7.43(0.12,468.82)										
ASV_2249	Firmicutes;D_2__Clostridia;D_3__Clostridiales;D_4__Lachnospiraceae;D_5__Lachnospiraceae FCS020 group;D_6__uncultured organism;D_7__;D_8__;D_9__;D_10__;D_11__;D_12__;D_13__;D_14__	119634370249.77(0.00,Inf)	319216512963.19(0.00,Inf)	2.67(0.22,32.10)	0.00(0.00,Inf)	0.91(0.07,12.39)	1676113194112641.00(0.00,Inf)	6931352042557759.00(0.00,Inf)	4.14(0.29,57.99)	0.00(0.00,Inf)	0.76(0.05,11.35)					
ASV_2258	Bacteroidetes;D_2__Bacteroidia;D_3__Bacteroidales;D_4__Porphyromonadaceae;D_5__Porphyromonas;D_6__Porphyromonas bennonis;D_7__;D_8__;D_9__;D_10__;D_11__;D_12__;D_13__;D_14__	0.13(0.00,5.97)	0.47(0.02,14.33)	3.60(0.24,54.04)	6.36(0.18,218.60)	0.83(0.05,14.37)	0.14(0.00,6.60)	0.57(0.02,19.59)	3.95(0.24,64.91)	5.48(0.15,198.93)	0.79(0.04,14.34)					
ASV_2265	Firmicutes;D_2__Clostridia;D_3__Clostridiales;D_4__Lachnospiraceae;D_5__[Ruminococcus] torques group;D_6__Ruminococcus sp. AT10;D_7__;D_8__;D_9__;D_10__;D_11__;D_12__;D_13__;D_14__	6.84(0.02,2340.22)	3.51(0.01,876.43)	0.51(0.01,19.85)	0.09(0.00,24.26)	0.64(0.02,26.23)										
ASV_2311	Firmicutes;D_2__Clostridia;D_3__Clostridiales;D_4__Family XI;D_5__Anaerococcus	2539202220.11(0.00,Inf)	13551244668.43(0.00,Inf)	5.34(0.28,100.52)	1.02(0.00,Inf)	2584655533.15(0.00,Inf)										
ASV_2314	Firmicutes;D_2__Clostridia;D_3__Clostridiales;D_4__Lachnospiraceae;D_5__[Ruminococcus] torques group	0.31(0.00,38.39)	0.10(0.00,9.57)	0.32(0.01,9.31)	15.50(0.14,1692.44)	4.86(0.14,169.50)										
ASV_2319	Firmicutes;D_2__Clostridia;D_3__Clostridiales;D_4__Ruminococcaceae;D_5__Faecalibacterium;D_6__uncultured Clostridiaceae bacterium;D_7__;D_8__;D_9__;D_10__;D_11__;D_12__;D_13__;D_14__	0.00(0.00,Inf)	0.08(0.00,10.22)	18141652992.50(0.00,Inf)	0.28(0.00,28.33)	0.00(0.00,Inf)										
ASV_2333	Firmicutes;D_2__Clostridia;D_3__Clostridiales;D_4__Defluviitaleaceae;D_5__Defluviitaleaceae UCG-011;D_6__uncultured bacterium;D_7__;D_8__;D_9__;D_10__;D_11__;D_12__;D_13__;D_14__	0.00(0.00,Inf)	0.08(0.00,7.91)	3789322188.16(0.00,Inf)	4.43(0.04,455.82)	0.00(0.00,Inf)										
ASV_2342	Firmicutes;D_2__Clostridia;D_3__Clostridiales;D_4__Family XIII;D_5__Family XIII AD3011 group	15785867848.32(0.00,Inf)	26133787513.42(0.00,Inf)	1.66(0.03,79.81)	0.00(0.00,Inf)	0.09(0.00,4.34)										
ASV_2360	Firmicutes;D_2__Clostridia;D_3__Clostridiales;D_4__Ruminococcaceae;D_5__Oscillibacter	959078571686.33(0.00,Inf)	257508666480.01(0.00,Inf)	0.27(0.02,4.80)	1.02(0.00,Inf)	976262707147.18(0.00,Inf)										
ASV_2483	Firmicutes;D_2__Clostridia;D_3__Clostridiales;D_4__Peptococcaceae;D_5__uncultured;D_6__metagenome;D_7__;D_8__;D_9__;D_10__;D_11__;D_12__;D_13__;D_14__	1.12(0.00,Inf)	350273272631.14(0.00,Inf)	313731608016.78(0.00,Inf)	0.00(0.00,Inf)	0.00(0.00,Inf)	1.40(0.00,Inf)	2319700607877396.00(0.00,Inf)	1580330711531387.00(0.00,Inf)	0.00(0.00,Inf)	0.00(0.00,Inf)					
ASV_2484	Firmicutes;D_2__Clostridia;D_3__Clostridiales;D_4__Ruminococcaceae;D_5__CAG-352;D_6__uncultured bacterium;D_7__;D_8__;D_9__;D_10__;D_11__;D_12__;D_13__;D_14__	50028755823.87(0.00,Inf)	67579820806.43(0.00,Inf)	1.35(0.07,27.59)	0.00(0.00,Inf)	0.54(0.02,11.54)										
ASV_	Proteobacteria;D_2__Gammaproteobacteria;D_3__Betaproteobacteriales;D_4__Burkholderiaceae;D_5__Noviherbaspirillum	291482309900.83(0.00,Inf)	113979532490.99(0.00,Inf)	0.39(0.01,10.91)	0.00(0.00,Inf)	0.53(0.02,12.87)										

2485																
ASV_2490	Firmicutes;D_2__Clostridia;D_3__Clostridiales	120979173017.02(0.00,Inf)	63399711110.30(0.00,Inf)	0.52(0.02,18.11)	0.00(0.00,Inf)	1.82(0.05,68.50)										
ASV_2491	Firmicutes;D_2__Clostridia;D_3__Clostridiales;D_4__Lachnospiraceae	744601320303.10(0.00,Inf)	50762307480.16(0.00,Inf)	0.07(0.00,3.31)	0.00(0.00,Inf)	1.98(0.04,89.99)	12140280097331124.00(0.00,Inf)	1658206583173492.00(0.00,Inf)	0.14(0.00,7.75)	0.00(0.00,Inf)	1.15(0.02,55.95)					
ASV_2517	Patescibacteria;D_2__Saccharimonadia;D_3__Saccharimonadales;D_4__Saccharimonadaceae;D_5__Candidatus Saccharimonas	0.26(0.00,16.11)	0.07(0.00,4.04)	0.27(0.01,7.29)	18609867298529.70(0.00,Inf)	4768596408851.07(0.00,Inf)										
ASV_2518	Firmicutes;D_2__Clostridia;D_3__Clostridiales;D_4__Lachnospiraceae	555577642334.95(0.00,Inf)	918403050077.60(0.00,Inf)	1.65(0.07,36.80)	0.00(0.00,Inf)	1.40(0.05,38.02)										
ASV_2520	Firmicutes;D_2__Clostridia;D_3__Clostridiales;D_4__Lachnospiraceae;D_5__Roseburia	1.12(0.00,Inf)	2341030868.72(0.00,Inf)	2096835468.33(0.00,Inf)	0.00(0.00,Inf)	0.00(0.00,Inf)										
ASV_2530	Firmicutes;D_2__Clostridia;D_3__Clostridiales;D_4__Ruminococcaceae;D_5__Ruminiclostridium 5	155731167860282.00(0.00,Inf)	20326628778916.20(0.00,Inf)	0.13(0.00,5.36)	1.02(0.00,Inf)	159322538641324.00(0.00,Inf)										
ASV_2574	Actinobacteria;D_2__Actinobacteria;D_3__Propionibacteriales;D_4__Propionibacteriaceae;D_5__Propionibacterium;D_6__Propionibacterium freudenreichii;D_7__;D_8__;D_9__;D_10__;D_11__;D_12__;D_13__;D_14__	1.12(0.00,Inf)	155107755889.97(0.00,Inf)	138928415377.31(0.00,Inf)	0.00(0.00,Inf)	0.00(0.00,Inf)										
ASV_2579	Firmicutes;D_2__Clostridia;D_3__Clostridiales;D_4__Ruminococcaceae;D_5__Subdoligranulum;D_6__uncultured bacterium;D_7__;D_8__;D_9__;D_10__;D_11__;D_12__;D_13__;D_14__	0.07(0.00,3.55)	0.07(0.00,2.21)	0.94(0.04,23.83)	4.96(0.17,147.18)	0.34(0.02,7.53)	0.04(0.00,2.85)	0.01(0.00,0.57);p=0.02674	0.20(0.00,8.63)	11.04(0.34,361.20)	0.43(0.01,12.63)		↓			
ASV_2582	Firmicutes;D_2__Clostridia;D_3__Clostridiales;D_4__Lachnospiraceae;D_5__Blautia	0.00(0.00,Inf)	0.26(0.00,21.41)	120561192088.46(0.00,Inf)	0.22(0.00,12.52)	0.00(0.00,Inf)	0.00(0.00,Inf)	0.19(0.00,17.57)	176644379060791.00(0.00,Inf)	0.28(0.01,13.58)	0.00(0.00,Inf)					
ASV_2593	Firmicutes;D_2__Clostridia;D_3__Clostridiales;D_4__Family XIII;D_5__Family XIII AD3011 group;D_6__uncultured Eubacterium sp.;D_7__;D_8__;D_9__;D_10__;D_11__;D_12__;D_13__;D_14__	0.36(0.00,38.57)	0.26(0.00,20.86)	0.74(0.03,18.35)	0.55(0.01,42.90)	0.20(0.01,4.86)										
ASV_2599	Firmicutes;D_2__Clostridia;D_3__Clostridiales;D_4__Ruminococcaceae;D_5__Subdoligranulum;D_6__uncultured bacterium;D_7__;D_8__;D_9__;D_10__;D_11__;D_12__;D_13__;D_14__	1.12(0.00,Inf)	622361512018.70(0.00,Inf)	557444425598.62(0.00,Inf)	0.00(0.00,Inf)	0.00(0.00,Inf)										
ASV_2603	Firmicutes;D_2__Clostridia;D_3__Clostridiales;D_4__Ruminococcaceae;D_5__DTU089	0.19(0.00,25.41)	0.09(0.00,8.93)	0.46(0.01,15.35)	6.33(0.06,635.93)	1.22(0.04,40.40)	0.21(0.00,25.65)	0.10(0.00,11.78)	0.49(0.01,20.08)	2.82(0.03,269.01)	0.59(0.02,20.25)					
ASV_2611	Firmicutes;D_2__Clostridia;D_3__Clostridiales;D_4__Lachnospiraceae;D_5__[Ruminococcus] gauvreauii group;D_6__Ruminococcus gauvreauii;D_7__;D_8__;D_9__;D_10__;D_11__;D_12__;D_13__;D_14__	0.21(0.00,11.19)	0.23(0.01,9.34)	1.09(0.08,15.57)	17.17(0.39,763.06)	3.66(0.22,60.11)										
ASV_2616	Firmicutes;D_2__Clostridia;D_3__Clostridiales;D_4__Peptostreptococcaceae;D_5__uncultured;D_6__uncultured bacterium;D_7__;D_8__;D_9__;D_10__;D_11__;D_12__;D_13__;D_14__	286937041462.23(0.00,Inf)	153317787982.76(0.00,Inf)	0.53(0.01,26.97)	0.00(0.00,Inf)	0.61(0.01,25.16)										

ASV_2636	Firmicutes;D_2__Clostridia;D_3__Clostridiales;D_4__Ruminococcaceae	1.12(0.00,Inf)	146374528084.91(0.00,Inf)	131105653847.08(0.00,Inf)	0.00(0.00,Inf)	0.00(0.00,Inf)										
ASV_2640	Proteobacteria;D_2__Deltaproteobacteria;D_3__Desulfovibrionales;D_4__Desulfovibrionaceae;D_5__Bilophila	164742121707765.00(0.00,Inf)	36054923001666.50(0.00,Inf)	0.22(0.01,3.63)	0.00(0.00,Inf)	6.32(0.30,131.53)										
ASV_2641	Firmicutes;D_2__Clostridia;D_3__Clostridiales;D_4__Lachnospiraceae;D_5__[Eubacterium] fissicatena group;D_6__uncultured bacterium;D_7__;D_8__;D_9__;D_10__;D_11__;D_12__;D_13__;D_14__	28103189762.82(0.00,Inf)	52762332942.11(0.00,Inf)	1.88(0.07,50.92)	0.00(0.00,Inf)	0.34(0.01,9.77)	1019389533627023.00(0.00,Inf)	4695486272631860.00(0.00,Inf)	4.61(0.13,165.96)	0.00(0.00,Inf)	0.07(0.00,2.17)					
ASV_2652	Proteobacteria;D_2__Gammaproteobacteria;D_3__Betaproteobacteriales;D_4__Burkholderiaceae;D_5__Parasutterella;D_6__uncultured bacterium;D_7__;D_8__;D_9__;D_10__;D_11__;D_12__;D_13__;D_14__	37892715108878.90(0.00,Inf)	2738416388848.07(0.00,Inf)	0.07(0.00,1.22)	0.00(0.00,Inf)	20.99(0.83,533.21)	3386439171337061.00(0.00,Inf)	175044919499033.00(0.00,Inf)	0.05(0.00,1.08)	0.00(0.00,Inf)	36.02(0.98,1318.52)					
ASV_2664	Firmicutes;D_2__Clostridia;D_3__Clostridiales;D_4__Lachnospiraceae;D_5__Blautia;D_6__Blautia sp. Marseille-P3087;D_7__;D_8__;D_9__;D_10__;D_11__;D_12__;D_13__;D_14__	41065066214850.70(0.00,Inf)	28506040689546.80(0.00,Inf)	0.69(0.02,21.66)	0.00(0.00,Inf)	1.10(0.04,33.92)										
ASV_2665	Patescibacteria;D_2__Saccharimonadia;D_3__Saccharimonadales;D_4__TM7 phylum sp. oral clone FR058;D_5__;D_6__;D_7__;D_8__;D_9__;D_10__;D_11__;D_12__;D_13__;D_14__	1.62(0.05,55.66)	0.69(0.02,19.46)	0.43(0.04,4.48)	1.91(0.06,56.47)	3.09(0.27,34.93)										
ASV_2667	Firmicutes;D_2__Clostridia;D_3__Clostridiales;D_4__Family XIII;D_5__[Eubacterium] brachy group;D_6__uncultured bacterium;D_7__;D_8__;D_9__;D_10__;D_11__;D_12__;D_13__;D_14__	1.12(0.00,Inf)	1870246077867.86(0.00,Inf)	1675276360624.23(0.00,Inf)	0.00(0.00,Inf)	0.00(0.00,Inf)										
ASV_2683	Firmicutes;D_2__Clostridia;D_3__Clostridiales;D_4__Ruminococcaceae;D_5__[Eubacterium] coprostanoligenes group;D_6__unidentified;D_7__;D_8__;D_9__;D_10__;D_11__;D_12__;D_13__;D_14__	40641889132.00(0.00,Inf)	1.00(0.00,Inf)	0.00(0.00,Inf)	0.00(0.00,Inf)	5.74(0.15,213.41)										
ASV_2684	Firmicutes;D_2__Clostridia;D_3__Clostridiales;D_4__Lachnospiraceae	316789871281.83(0.00,Inf)	80943660895.40(0.00,Inf)	0.26(0.02,3.73)	0.00(0.00,Inf)	5.60(0.33,94.67)	5684758185752220.00(0.00,Inf)	2203770805066833.00(0.00,Inf)	0.39(0.02,6.28)	0.00(0.00,Inf)	4.69(0.26,83.88)					
ASV_2685	Firmicutes;D_2__Clostridia;D_3__Clostridiales;D_4__Lachnospiraceae	57333651707196.00(0.00,Inf)	48491723125310.50(0.00,Inf)	0.85(0.08,8.91)	0.00(0.00,Inf)	6.71(0.36,125.81)	3165514955719046.00(0.00,Inf)	3014185936211939.00(0.00,Inf)	0.95(0.08,10.95)	0.00(0.00,Inf)	8.54(0.39,186.42)					
ASV_2687	Actinobacteria;D_2__Actinobacteria;D_3__Actinomycetales;D_4__Actinomycetaceae;D_5__Actinomyces	3910233824.72(0.00,Inf)	6279161497.37(0.00,Inf)	1.61(0.11,23.86)	0.00(0.00,Inf)	1.43(0.08,25.94)										
ASV_2732	Firmicutes;D_2__Clostridia;D_3__Clostridiales;D_4__Lachnospiraceae;D_5__Blautia	584791076741396.00(0.00,Inf)	697913618427603.00(0.00,Inf)	1.19(0.03,46.33)	1.00(0.00,Inf)	597510587665894.00(0.00,Inf)										
ASV_2778	Firmicutes;D_2__Clostridia;D_3__Clostridiales;D_4__Lachnospiraceae;D_5__GCA-900066575;D_6__uncultured bacterium;D_7__;D_8__;D_9__;D_10__;D_11__;D_12__;D_13__;D_14__	20.98(0.35,1264.06)	3.11(0.06,155.92)	0.15(0.01,1.77)	0.28(0.01,14.57)	5.87(0.46,74.35)	25.53(0.45,1440.76)	1.69(0.03,90.12)	0.07(0.01,0.83);p=0.03546	0.49(0.01,25.62)	12.59(1.01,157.55);p=0.0495			↓		↑
ASV_2847	Actinobacteria;D_2__Actinobacteria;D_3__Corynebacteriales;D_4__Corynebacteriaceae;D_5__Corynebacterium 1	1.12(0.00,Inf)	1632782659.33(0.00,Inf)	1462465770.63(0.00,Inf)	0.00(0.00,Inf)	0.00(0.00,Inf)	1.64(0.00,Inf)	1496557560687792.00(0.00,Inf)	934179304353412.00(0.00,Inf)	0.00(0.00,Inf)	0.00(0.00,Inf)					
ASV_2902	Firmicutes;D_2__Clostridia;D_3__Clostridiales;D_4__Lachnospiraceae	44141177239.66(0.00,Inf)	168646116711.31(0.00,Inf)	3.82(0.22,65.17)	0.00(0.00,Inf)	2.43(0.11,55.82)										

ASV_2903	Firmicutes;D_2__Clostridia;D_3__Clostridiales;D_4__Ruminococcaceae	0.00(0.00,Inf)	0.57(0.02,18.17)	26325354685.19(0.00,Inf)	10.02(0.24,417.14)	0.00(0.00,Inf)	0.00(0.00,Inf)	0.61(0.02,20.97)	1223576886822644.00(0.00,Inf)	15.16(0.27,854.11)	0.00(0.00,Inf)					
ASV_2904	Firmicutes;D_2__Clostridia;D_3__Clostridiales;D_4__Lachnospiraceae;D_5__Fusicatenibacter;D_6__uncultured bacterium;D_7__;D_8__;D_9__;D_10__;D_11__;D_12__;D_13__;D_14__	1.12(0.00,Inf)	243372321614.73(0.00,Inf)	217989171351.92(0.00,Inf)	0.00(0.00,Inf)	0.00(0.00,Inf)										
ASV_2920	Firmicutes;D_2__Clostridia;D_3__Clostridiales;D_4__Lachnospiraceae;D_5__Oribacterium;D_6__Oribacterium parvum ACB1;D_7__;D_8__;D_9__;D_10__;D_11__;D_12__;D_13__;D_14__	1.12(0.00,Inf)	1904825841887.23(0.00,Inf)	1706154762122.31(0.00,Inf)	0.00(0.00,Inf)	0.00(0.00,Inf)	1.38(0.00,Inf)	2677812068413738.00(0.00,Inf)	1936992100752034.00(0.00,Inf)	0.00(0.00,Inf)	0.00(0.00,Inf)					
ASV_2921	Actinobacteria;D_2__Coriobacteriia;D_3__Coriobacteriales;D_4__Eggerthellaceae;D_5__Slackia;D_6__Slackia sp. S4-C6;D_7__;D_8__;D_9__;D_10__;D_11__;D_12__;D_13__;D_14__	0.00(0.00,Inf)	0.31(0.01,14.75)	6874302006739.20(0.00,Inf)	1.24(0.03,55.71)	0.00(0.00,Inf)	0.00(0.00,Inf)	0.34(0.01,20.74)	791750611458816.00(0.00,Inf)	0.95(0.02,49.56)	0.00(0.00,Inf)					
ASV_2931	Firmicutes;D_2__Clostridia;D_3__Clostridiales;D_4__Christensenellaceae	0.23(0.01,7.91)	0.28(0.01,7.00)	1.24(0.09,16.77)	0.73(0.03,16.96)	0.17(0.01,2.12)										
ASV_2943	Firmicutes;D_2__Clostridia;D_3__Clostridiales;D_4__Lachnospiraceae;D_5__Coprococcus 3	288088412134.87(0.00,Inf)	133788077772.31(0.00,Inf)	0.46(0.01,27.63)	0.00(0.00,Inf)	0.48(0.01,21.38)	21925704160032.40(0.00,Inf)	14218847655958.50(0.00,Inf)	0.65(0.01,43.80)	0.00(0.00,Inf)	0.70(0.01,35.41)					
ASV_2951	Firmicutes;D_2__Clostridia;D_3__Clostridiales;D_4__Ruminococcaceae;D_5__Faecalibacterium	22488238426022.30(0.00,Inf)	18497962793470.60(0.00,Inf)	0.82(0.02,38.54)	1.02(0.00,Inf)	22861547351101.00(0.00,Inf)										
ASV_2955	Firmicutes;D_2__Clostridia;D_3__Clostridiales;D_4__Family XIII;D_5__uncultured;D_6__uncultured bacterium;D_7__;D_8__;D_9__;D_10__;D_11__;D_12__;D_13__;D_14__	1.12(0.00,Inf)	4851926287.07(0.00,Inf)	4345819662.96(0.00,Inf)	0.00(0.00,Inf)	0.00(0.00,Inf)										
ASV_2984	Firmicutes;D_2__Clostridia;D_3__Clostridiales;D_4__Ruminococcaceae;D_5__Ruminiclostridium 5;D_6__uncultured bacterium;D_7__;D_8__;D_9__;D_10__;D_11__;D_12__;D_13__;D_14__	1631763450332.99(0.00,Inf)	345654139447.05(0.00,Inf)	0.21(0.01,6.32)	1.02(0.00,Inf)	1660885261585.17(0.00,Inf)										
ASV_2998	Firmicutes;D_2__Clostridia;D_3__Clostridiales;D_4__Lachnospiraceae;D_5__CHKCI001;D_6__uncultured organism;D_7__;D_8__;D_9__;D_10__;D_11__;D_12__;D_13__;D_14__	407485206100.15(0.00,Inf)	142459348068.77(0.00,Inf)	0.35(0.00,25.82)	0.00(0.00,Inf)	2.93(0.04,243.30)										
ASV_3031	Firmicutes;D_2__Clostridia;D_3__Clostridiales;D_4__Family XIII;D_5__Family XIII AD3011 group;D_6__uncultured bacterium;D_7__;D_8__;D_9__;D_10__;D_11__;D_12__;D_13__;D_14__	1.12(0.00,Inf)	12537929793060.90(0.00,Inf)	11231122633430.80(0.00,Inf)	0.00(0.00,Inf)	0.00(0.00,Inf)										
ASV_3033	Firmicutes;D_2__Clostridia;D_3__Clostridiales;D_4__Lachnospiraceae;D_5__Blautia	1.12(0.00,Inf)	13092358702.60(0.00,Inf)	11726701830.98(0.00,Inf)	0.00(0.00,Inf)	0.00(0.00,Inf)										
ASV_3048	Firmicutes;D_2__Clostridia;D_3__Clostridiales;D_4__Lachnospiraceae	83157725668.59(0.00,Inf)	19799391535.53(0.00,Inf)	0.24(0.00,13.44)	0.00(0.00,Inf)	1.26(0.03,57.44)	1094988141113235.00(0.00,Inf)	672924873557281.00(0.00,Inf)	0.61(0.01,45.99)	0.00(0.00,Inf)	0.58(0.01,34.12)					
ASV_3052	Firmicutes;D_2__Clostridia;D_3__Clostridiales;D_4__Lachnospiraceae;D_5__Agathobacter	227108484535.00(0.00,Inf)	211946134687.77(0.00,Inf)	0.93(0.02,41.28)	0.00(0.00,Inf)	0.87(0.02,41.26)										
ASV_3073	Proteobacteria;D_2__Gammaproteobacteria;D_3__Pasteurellales;D_4__Pasteurellaceae;D_5__Aggregatibacter						0.02(0.00,Inf)	0.01(0.00,Inf)	0.39(0.00,Inf)	0.00(0.00,Inf)	0.00(0.00,Inf)					

ASV_3084	Firmicutes;D_2__Clostridia;D_3__Clostridiales;D_4__Ruminococcaceae	0.55(0.00,67.43)	0.09(0.00,8.98)	0.16(0.01,4.75)	1.38(0.01,130.19)	0.76(0.03,20.40)	0.61(0.01,70.60)	0.07(0.00,7.80)	0.11(0.00,3.57)	2.88(0.03,279.74)	1.75(0.07,47.20)					
ASV_3086	Firmicutes;D_2__Clostridia;D_3__Clostridiales;D_4__Lachnospiraceae;D_5__[Eubacterium] hallii group	1.12(0.00,Inf)	18146773014.06(0.00,Inf)	16253921333.78(0.00,Inf)	0.00(0.00,Inf)	0.00(0.00,Inf)										
ASV_3095	Firmicutes;D_2__Clostridia;D_3__Clostridiales;D_4__Lachnospiraceae;D_5__Anaerostipes	1502196463063.96(0.00,Inf)	1106091696311.25(0.00,Inf)	0.74(0.07,7.31)	0.00(0.00,Inf)	3.93(0.24,64.71)										
ASV_3137	Firmicutes;D_2__Clostridia;D_3__Clostridiales;D_4__Lachnospiraceae;D_5__Lachnoclostridium						2893954926907340.00(0.00,Inf)	5220875867687192.00(0.00,Inf)	1.80(0.03,109.28)	0.00(0.00,Inf)	0.31(0.01,17.82)					
ASV_3165	Firmicutes;D_2__Clostridia;D_3__Clostridiales;D_4__Ruminococcaceae;D_5__Ruminiclostridium 5						1693476712757558.00(0.00,Inf)	1993475328747998.00(0.00,Inf)	1.18(0.01,100.50)	0.00(0.00,Inf)	0.31(0.00,25.94)					
ASV_3168	Firmicutes;D_2__Bacilli;D_3__Lactobacillales;D_4__Streptococcaceae;D_5__Streptococcus	992339247497.16(0.00,Inf)	685573604556.30(0.00,Inf)	0.69(0.07,7.23)	0.00(0.00,Inf)	4.32(0.29,64.73)										
ASV_3171	Firmicutes;D_2__Clostridia;D_3__Clostridiales;D_4__Lachnospiraceae;D_5__uncultured	41503900249873.00(0.00,Inf)	34633589869163.40(0.00,Inf)	0.83(0.03,23.68)	0.00(0.00,Inf)	3.00(0.08,111.92)										
ASV_3180	Firmicutes;D_2__Clostridia;D_3__Clostridiales;D_4__Lachnospiraceae	79759017342.80(0.00,Inf)	32399351745.31(0.00,Inf)	0.41(0.01,14.65)	0.00(0.00,Inf)	0.70(0.02,21.99)	788560104436554.00(0.00,Inf)	260231304644194.00(0.00,Inf)	0.33(0.01,15.09)	0.00(0.00,Inf)	0.75(0.02,26.77)					
ASV_3189	Firmicutes;D_2__Clostridia;D_3__Clostridiales;D_4__Lachnospiraceae	1.12(0.00,Inf)	176759397896.71(0.00,Inf)	158321557490.98(0.00,Inf)	0.00(0.00,Inf)	0.00(0.00,Inf)										
ASV_3250	Firmicutes;D_2__Clostridia;D_3__Clostridiales;D_4__Ruminococcaceae;D_5__Hydrogenoanaerobacterium;D_6__uncultured bacterium;D_7__;D_8__;D_9__;D_10__;D_11__;D_12__;D_13__;D_14__	83303868943.67(0.00,Inf)	66842050536.18(0.00,Inf)	0.80(0.02,36.60)	0.00(0.00,Inf)	0.70(0.01,32.72)										
ASV_3323	Firmicutes;D_2__Clostridia;D_3__Clostridiales;D_4__Ruminococcaceae;D_5__uncultured;D_6__uncultured bacterium;D_7__;D_8__;D_9__;D_10__;D_11__;D_12__;D_13__;D_14__	0.50(0.00,80.67)	0.67(0.01,73.81)	1.32(0.04,41.28)	5.89(0.04,806.70)	2.97(0.07,123.77)										
ASV_3342	Firmicutes;D_2__Clostridia;D_3__Clostridiales;D_4__Lachnospiraceae	1.12(0.00,Inf)	81566803754.03(0.00,Inf)	73058266815.47(0.00,Inf)	0.00(0.00,Inf)	0.00(0.00,Inf)										
ASV_3368	Actinobacteria;D_2__Coriobacteriia;D_3__Coriobacteriales;D_4__Coriobacteriaceae;D_5__Collinsella;D_6__uncultured bacterium;D_7__;D_8__;D_9__;D_10__;D_11__;D_12__;D_13__;D_14__	3745201086.21(0.00,Inf)	1404163003.73(0.00,Inf)	0.37(0.02,7.20)	0.00(0.00,Inf)	0.73(0.05,11.02)										
ASV_3395	Firmicutes;D_2__Clostridia;D_3__Clostridiales;D_4__Lachnospiraceae;D_5__Lachnospiraceae NC2004 group;D_6__uncultured bacterium;D_7__;D_8__;D_9__;D_10__;D_11__;D_12__;D_13__;D_14__	0.92(0.05,18.77)	0.00(0.00,Inf)	0.00(0.00,Inf)	1038665115215407.00(0.00,Inf)	956868183809099.00(0.00,Inf)										
ASV_3560	Firmicutes;D_2__Clostridia;D_3__Clostridiales;D_4__Lachnospiraceae						0.00(0.00,Inf)	0.00(0.00,Inf)	0.76(0.00,Inf)	9442919358854.81(0.00,Inf)	0.37(0.00,Inf)					

ASV_3680	Firmicutes;D_2__Clostridia;D_3__Clostridiales;D_4__Lachnospiraceae;D_5__Lachnospiraceae UCG-004	0.69(0.01,83.21)	1.58(0.02,134.17)	2.29(0.09,55.32)	13.67(0.10,1879.15)	9.44(0.21,434.10)										
ASV_3748	Firmicutes;D_2__Clostridia;D_3__Clostridiales;D_4__Ruminococcaceae;D_5__Subdoligranulum;D_6__uncultured bacterium;D_7__;D_8__;D_9__;D_10__;D_11__;D_12__;D_13__;D_14__	0.00(0.00,Inf)	0.53(0.04,7.43)	271509052721.48(0.00,Inf)	11.30(0.46,278.24)	0.00(0.00,Inf)	0.00(0.00,Inf)	0.30(0.02,4.22)	561991701366613.00(0.00,Inf)	14.23(0.62,325.73)	0.00(0.00,Inf)					
ASV_3939	Firmicutes;D_2__Clostridia;D_3__Clostridiales;D_4__Ruminococcaceae;D_5__Faecalibacterium	1.12(0.00,Inf)	60297607329.55(0.00,Inf)	54008212516.81(0.00,Inf)	0.00(0.00,Inf)	0.00(0.00,Inf)										
ASV_3940	Firmicutes;D_2__Bacilli;D_3__Lactobacillales;D_4__Streptococcaceae;D_5__Streptococcus	1.12(0.00,Inf)	361033240903.45(0.00,Inf)	323374521978.93(0.00,Inf)	0.00(0.00,Inf)	0.00(0.00,Inf)										
ASV_4462	Firmicutes;D_2__Clostridia;D_3__Clostridiales;D_4__Lachnospiraceae;D_5__[Eubacterium] hallii group	1.06(0.00,Inf)	0.95(0.00,Inf)	0.90(0.00,Inf)	0.00(0.00,Inf)	0.00(0.00,Inf)										

Supplementary File: Metagenomic predictions (PICRUSt2)

Supplementary Table 4: The relative abundance of 193 predicted metagenomic pathways and related comparisons between the unaffected controls (n=36), ADS participants (n=41), and MS cases (all MS cases, n=32, then by DMD exposure status [exposed, n=23 or naïve, n=9])

FULL PATHWAY NAME	MS	CTR	ADS	MS vs ADS vs CTR		MS vs ADS	MS vs ADS2	MS vs CTR	MS vs CTR p-a.	ADS vs CTR	ADS vs CTR p-a.		DMD exposed MS cases	DMD naïve MS cases	DMD exposed vs naïve MS cases	DMD naïve MS cases vs. ADS	DMD exposed vs ADS	DMD naïve MS cases vs CTR	DMD exposed MS cases vs CTR
	Median abundance			p-value		p-u	p-a	p-u	p-a	p-u	p-a		Median abundance		p-u (p-a not shown; all >0.05)	p-u (p-a not shown; all >0.05)	p-u (p-a not shown; all >0.05)	p-u (1 p-a <0.05)	p-u (p-a not shown; all >0.05)
**(5Z)-dodec-5-enoate biosynthesis**	0.315	0.285	0.456	**0.036**		0.067	0.135	0.603	0.603	**0.015**	**0.044**		0.275	0.462	0.190	0.868	**0.027**	0.183	0.945
**5-aminoimidazole ribonucleotide biosynthesis I**	0.846	0.851	0.835	0.057		0.347	0.347	0.182	0.364	**0.017**	0.051		0.849	0.843	0.811	0.676	0.341	0.293	0.265
**5-aminoimidazole ribonucleotide biosynthesis II**	0.833	0.837	0.816	0.081		0.316	0.316	0.257	0.514	**0.025**	0.075		0.834	0.832	0.790	0.662	0.307	0.347	0.357
**6-hydroxymethyl-dihydropterin diphosphate biosynthesis III (Chlamydia)**	0.527	0.516	0.547	0.129		0.245	0.491	0.450	0.450	**0.045**	0.136		0.527	0.527	0.949	0.487	0.281	0.589	0.509
**8-amino-7-oxononanoate biosynthesis I**	0.318	0.275	0.382	**0.027**		0.302	0.302	0.128	0.256	**0.007**	**0.022**		0.296	0.447	0.113	0.579	0.108	**0.028**	0.466
**acetylene degradation**	0.592	0.645	0.546	**0.023**		0.344	0.344	0.101	0.203	**0.007**	**0.020**		0.604	0.570	0.538	0.893	0.263	0.125	0.216
**adenine and adenosine salvage III**	0.805	0.817	0.785	0.066		0.161	0.323	0.425	0.425	**0.022**	0.065		0.807	0.797	0.313	0.902	0.090	0.199	0.759
**adenosine deoxyribonucleotides de novo biosynthesis II**	0.893	0.887	0.849	**0.039**		0.091	0.181	0.499	0.499	**0.014**	**0.041**		0.922	0.847	0.155	0.994	**0.033**	0.129	0.979
**adenosine ribonucleotides de novo biosynthesis**	0.898	0.924	0.901	**0.023**		0.593	0.593	**0.049**	0.097	**0.008**	**0.024**		0.900	0.890	0.936	0.779	0.604	0.178	0.078
**biotin biosynthesis I**	0.323	0.263	0.379	**0.016**		0.349	0.349	0.078	0.156	**0.004**	**0.013**		0.300	0.428	0.141	0.596	0.141	**0.023**	0.320
**CDP-diacylglycerol biosynthesis I**	0.897	0.933	0.885	0.085		0.295	0.295	0.284	0.568	**0.026**	0.079		0.902	0.862	0.358	0.972	0.181	0.163	0.553
**CDP-diacylglycerol biosynthesis II**	0.897	0.933	0.885	0.085		0.295	0.295	0.284	0.568	**0.026**	0.079		0.902	0.862	0.358	0.972	0.181	0.163	0.553

**chorismate biosynthesis from 3-dehydroquinate**	0.817	0.834	0.808	0.077		0.589	0.589	0.122	0.244	**0.028**	0.083		0.817	0.816	0.499	0.863	0.437	0.128	0.259
**chorismate biosynthesis I**	0.827	0.854	0.822	0.054		0.903	0.903	0.050	0.100	**0.027**	0.081		0.831	0.796	0.355	0.527	0.615	**0.048**	0.162
**CMP-3-deoxy-D-manno-octulosonate biosynthesis I**	0.207	0.192	0.241	0.052		0.563	0.563	0.097	0.193	**0.018**	0.054		0.203	0.260	0.244	0.600	0.309	**0.049**	0.303
**fatty acid elongation -- saturated**	0.533	0.474	0.594	0.069		0.525	0.525	0.129	0.259	**0.023**	0.070		0.532	0.576	0.936	0.729	0.542	0.294	0.178
**formaldehyde assimilation II (RuMP Cycle)**	0.114	0.068	0.085	0.117		0.425	0.425	**0.041**	0.124	0.178	0.357		0.109	0.155	0.256	0.167	0.811	**0.028**	0.166
**galactose degradation I (Leloir pathway)**	0.825	0.862	0.817	0.082		0.729	0.729	0.096	0.192	**0.033**	0.100		0.837	0.817	0.488	0.756	0.543	0.107	0.220
**glycolysis III (from glucose)**	0.934	0.960	0.925	0.053		0.422	0.422	0.139	0.278	**0.016**	**0.049**		0.934	0.940	0.230	0.684	0.216	0.061	0.396
**guanosine deoxyribonucleotides de novo biosynthesis II**	0.893	0.887	0.849	**0.039**		0.091	0.181	0.499	0.499	**0.014**	**0.041**		0.922	0.847	0.155	0.994	**0.033**	0.129	0.979
**guanosine ribonucleotides de novo biosynthesis**	0.820	0.838	0.819	0.053		0.477	0.477	0.121	0.242	**0.017**	0.051		0.819	0.821	0.521	0.971	0.359	0.134	0.252
**incomplete reductive TCA cycle**	0.604	0.558	0.666	0.056		0.421	0.421	0.145	0.290	**0.017**	0.052		0.601	0.674	0.584	0.924	0.336	0.172	0.272
**inosine-5'-phosphate biosynthesis I**	0.799	0.810	0.790	**0.010**		0.194	0.194	0.114	0.227	**0.003**	**0.008**		0.800	0.796	0.976	0.393	0.245	0.313	0.146
**Kdo transfer to lipid IVA III (Chlamydia)**	0.189	0.168	0.216	0.092		0.614	0.614	0.132	0.264	**0.034**	0.101		0.185	0.240	0.590	0.928	0.493	0.164	0.251
**L-arginine biosynthesis I (via L-ornithine)**	0.609	0.645	0.597	0.129		0.566	0.566	0.189	0.379	**0.047**	0.140		0.621	0.601	0.641	0.992	0.473	0.226	0.317
**L-arginine biosynthesis IV (archaebacteria)**	0.609	0.645	0.598	0.131		0.563	0.563	0.192	0.384	**0.047**	0.141		0.620	0.603	0.629	1.000	0.466	0.224	0.323
**L-histidine degradation I**	0.195	0.164	0.229	0.056		0.356	0.356	0.176	0.351	**0.017**	**0.050**		0.194	0.202	0.572	0.874	0.282	0.190	0.318
**lipid IVA biosynthesis**	0.217	0.188	0.248	0.057		0.622	0.622	0.091	0.182	**0.021**	0.063		0.209	0.271	0.427	0.769	0.433	0.088	0.226
**L-isoleucine biosynthesis I (from threonine)**	0.896	0.915	0.888	**0.037**		0.736	0.736	0.051	0.102	**0.015**	**0.046**		0.910	0.892	0.486	0.750	0.548	0.072	0.137
**L-isoleucine biosynthesis II**	0.976	0.998	0.972	**0.038**		0.628	0.628	0.065	0.131	**0.014**	**0.042**		0.989	0.970	0.425	0.762	0.437	0.071	0.178
**L-isoleucine biosynthesis III**	0.842	0.869	0.841	**0.019**		0.842	0.842	**0.025**	0.050	**0.010**	**0.029**		0.844	0.839	0.568	0.756	0.673	0.058	0.071
**L-lysine biosynthesis III**	0.886	0.916	0.891	**0.021**		0.888	0.888	**0.024**	**0.049**	**0.011**	**0.033**		0.897	0.871	0.389	0.569	0.622	**0.034**	0.091
**L-lysine biosynthesis VI**	0.878	0.897	0.871	**0.018**		0.489	0.489	0.054	0.109	**0.006**	**0.017**		0.882	0.865	0.335	0.766	0.300	**0.047**	0.177
**L-valine biosynthesis**	0.896	0.915	0.888	**0.037**		0.736	0.736	0.051	0.102	**0.015**	**0.046**		0.910	0.892	0.486	0.750	0.548	0.072	0.137
**mannan degradation**	0.242	0.217	0.255	0.099		0.756	0.756	0.107	0.214	**0.042**	0.125		0.237	0.291	0.232	0.473	0.430	0.050	0.331
**methylerythritol phosphate pathway I**	0.800	0.830	0.803	**0.023**		0.731	0.731	**0.014**	**0.041**	**0.023**	**0.047**		0.801	0.785	0.654	0.573	0.904	0.051	**0.039**
**methylerythritol phosphate pathway II**	0.800	0.830	0.803	**0.023**		0.731	0.731	**0.014**	**0.041**	**0.023**	**0.047**		0.801	0.785	0.654	0.573	0.904	0.051	**0.039**
**mycolate biosynthesis**	0.359	0.328	0.512	**0.038**		0.069	0.139	0.605	0.605	**0.015**	**0.046**		0.317	0.516	0.191	0.873	**0.028**	0.184	0.944

**NAD salvage pathway I**	0.644	0.671	0.658	**0.031**		0.294	0.294	**0.009**	**0.028**	0.093	0.186		0.645	0.623	0.652	0.309	0.448	**0.042**	**0.029**
**O-antigen building blocks biosynthesis (E. coli)**	0.605	0.633	0.600	**0.019**		0.493	0.493	0.055	0.111	**0.006**	**0.018**		0.612	0.585	0.798	0.808	0.466	0.149	0.101
**oleate biosynthesis IV (anaerobic)**	0.352	0.320	0.500	**0.034**		0.060	0.119	0.631	0.631	**0.014**	**0.042**		0.309	0.507	0.185	0.850	**0.023**	0.187	0.911
**palmitoleate biosynthesis I (from (5Z)-dodec-5-enoate)**	0.321	0.289	0.466	**0.036**		0.064	0.129	0.622	0.622	**0.015**	**0.045**		0.279	0.473	0.190	0.858	**0.026**	0.188	0.925
**peptidoglycan biosynthesis I (meso-diaminopimelate cont.)**	0.782	0.808	0.780	**0.006**		0.633	0.633	**0.018**	**0.035**	**0.003**	**0.008**		0.786	0.772	0.577	0.903	0.504	**0.049**	0.053
**peptidoglycan biosynthesis III (mycobacteria)**	0.781	0.801	0.774	**0.003**		0.487	0.487	**0.016**	**0.032**	**0.001**	**0.003**		0.781	0.771	0.649	0.923	0.411	0.056	**0.045**
**peptidoglycan biosynthesis IV (Enterococcus faecium)**	0.528	0.554	0.509	**0.016**		0.610	0.610	**0.036**	0.073	**0.006**	**0.018**		0.535	0.465	0.350	0.696	0.390	**0.038**	0.129
**peptidoglycan maturation (meso-diaminopimelate cont.)**	0.603	0.654	0.555	0.114		0.383	0.383	0.266	0.532	**0.037**	0.112		0.607	0.585	0.826	0.696	0.377	0.373	0.357
**phosphatidylglycerol biosynthesis I (plastidic)**	0.822	0.847	0.784	**0.031**		0.336	0.336	0.125	0.251	**0.009**	**0.026**		0.831	0.782	0.362	0.934	0.208	0.091	0.309
**phosphatidylglycerol biosynthesis II (non-plastidic)**	0.822	0.847	0.784	**0.031**		0.336	0.336	0.125	0.251	**0.009**	**0.026**		0.831	0.782	0.362	0.934	0.208	0.091	0.309
**preQ0 biosynthesis**	0.327	0.318	0.363	0.119		0.291	0.582	0.363	0.363	**0.040**	0.119		0.325	0.358	0.620	0.767	0.243	0.332	0.534
**pyrimidine deoxyribonucleotide phosphorylation**	0.275	0.242	0.298	**0.017**		0.856	0.856	**0.022**	**0.045**	**0.009**	**0.027**		0.276	0.258	0.906	0.979	0.830	0.115	**0.042**
**pyrimidine deoxyribonucleotides de novo biosynthesis I**	0.293	0.260	0.287	0.053		0.983	0.983	**0.043**	0.087	**0.030**	0.090		0.299	0.276	0.819	0.871	0.907	0.136	0.081
**pyrimidine deoxyribonucleotides de novo biosynthesis III**	0.242	0.234	0.263	0.125		0.221	0.443	0.484	0.484	**0.045**	0.134		0.245	0.239	0.795	0.559	0.223	0.514	0.597
**pyruvate fermentation to acetate and lactate II**	0.811	0.878	0.811	**0.018**		0.902	0.902	**0.015**	**0.030**	**0.014**	**0.041**		0.807	0.817	0.943	0.981	0.887	0.125	**0.025**
**pyruvate fermentation to isobutanol (engineered)**	0.992	1.020	0.971	**0.039**		0.539	0.539	0.082	0.164	**0.013**	**0.039**		0.994	0.983	0.511	0.912	0.404	0.103	0.190
**pyruvate fermentation to propanoate I**	0.364	0.308	0.392	**0.030**		0.696	0.696	**0.047**	0.094	**0.012**	**0.036**		0.347	0.405	0.521	0.809	0.531	0.075	0.123
**queuosine biosynthesis**	0.397	0.376	0.435	0.137		0.468	0.468	0.246	0.492	**0.047**	0.142		0.384	0.424	0.873	0.732	0.469	0.380	0.322
**reductive TCA cycle I**	0.101	0.094	0.141	0.168		0.825	0.825	0.147	0.293	0.076	0.229		0.090	0.207	**0.016**	0.088	0.222	**0.006; p-a= 0.034**	0.746
**stearate biosynthesis II (bacteria and plants)**	0.322	0.292	0.480	**0.042**		0.065	0.131	0.662	0.662	**0.018**	0.054		0.279	0.481	0.185	0.870	**0.026**	0.197	0.880
**superpathway of β-D-glucuronide and D-glucuronate degradation**	0.228	0.225	0.251	0.142		0.062	0.185	0.628	0.628	0.157	0.314		0.226	0.229	0.524	0.479	**0.050**	0.867	0.481
**superpathway of 5-aminoimidazole ribonucleotide biosynthesis**	0.833	0.837	0.816	0.081		0.316	0.316	0.257	0.514	**0.025**	0.075		0.834	0.832	0.790	0.662	0.307	0.347	0.357
**superpathway of adenosine nucleotides de novo biosynthesis I**	0.904	0.930	0.890	**0.013**		0.385	0.385	0.058	0.116	**0.004**	**0.011**		0.909	0.856	0.206	0.679	0.186	**0.028**	0.229
**superpathway of adenosine nucleotides de novo biosynthesis II**	0.874	0.894	0.857	**0.020**		0.464	0.464	0.063	0.126	**0.006**	**0.019**		0.879	0.819	0.222	0.641	0.237	**0.033**	0.236

**superpathway of aromatic amino acid biosynthesis**	0.858	0.887	0.856	0.056		0.842	0.842	0.058	0.116	**0.026**	0.079		0.862	0.833	0.372	0.577	0.576	0.056	0.175
**superpathway of branched amino acid biosynthesis**	0.852	0.877	0.850	**0.019**		0.834	0.834	**0.025**	0.050	**0.009**	**0.028**		0.852	0.840	0.469	0.674	0.619	**0.045**	0.082
**superpathway of fatty acid biosynthesis initiation (E. coli)**	0.273	0.248	0.403	**0.038**		0.059	0.118	0.672	0.672	**0.016**	**0.049**		0.241	0.407	0.196	0.829	**0.024**	0.209	0.881
**superpathway of fucose and rhamnose degradation**	0.123	0.101	0.111	0.097		0.906	0.906	0.081	0.162	**0.048**	0.144		0.123	0.154	0.313	0.484	0.592	0.057	0.242
**superpath. GDP-mannose-derived O-antigen building blocks biosynthesis**	0.313	0.280	0.336	0.056		0.671	0.671	0.080	0.160	**0.021**	0.064		0.307	0.371	0.244	0.533	0.379	**0.043**	0.267
**superpathway of geranylgeranyl diphosphate biosynthesis II (via MEP)**	0.780	0.801	0.780	**0.031**		0.886	0.886	**0.023**	0.068	**0.023**	**0.045**		0.782	0.757	0.409	0.469	0.825	**0.035**	0.083
**superpathway of glucose and xylose degradation**	0.443	0.397	0.446	0.114		0.769	0.769	0.117	0.234	**0.049**	0.146		0.440	0.468	0.334	0.580	0.500	0.079	0.304
**superpathway of glycolysis and Entner-Doudoroff**	0.149	0.108	0.152	0.089		0.554	0.554	0.146	0.292	**0.031**	0.093		0.104	0.210	**0.043**	0.240	0.163	**0.013**	0.627
**superpathway of guanosine nucleotides de novo biosynthesis I**	0.388	0.331	0.403	**0.018**		0.897	0.897	**0.021**	**0.042**	**0.010**	**0.029**		0.394	0.375	0.808	0.918	0.826	0.091	**0.045**
**superpathway of guanosine nucleotides de novo biosynthesis II**	0.426	0.367	0.443	**0.016**		0.863	0.863	**0.021**	**0.042**	**0.008**	**0.025**		0.432	0.410	0.846	0.969	0.812	0.098	**0.043**
**superpathway of hexuronide and hexuronate degradation**	0.235	0.249	0.251	0.189		0.082	0.245	0.624	0.624	0.202	0.404		0.232	0.254	0.242	0.829	**0.038**	0.570	0.352
**superpathway of L-isoleucine biosynthesis I**	0.840	0.853	0.838	**0.040**		0.913	0.913	**0.039**	0.077	**0.021**	0.062		0.840	0.839	0.597	0.737	0.746	0.080	0.096
**superpathway of N-acetylneuraminate degradation**	0.242	0.194	0.252	0.095		0.592	0.592	0.142	0.284	**0.034**	0.103		0.191	0.309	0.054	0.256	0.192	**0.016**	0.591
**superpathway of phospholipid biosynthesis I (bacteria)**	0.850	0.877	0.828	**0.038**		0.385	0.385	0.123	0.247	**0.011**	**0.034**		0.852	0.812	0.359	0.883	0.239	0.089	0.307
**superpathway of purine deoxyribonucleosides degradation**	0.554	0.569	0.508	0.083		0.181	0.361	0.444	0.444	**0.028**	0.084		0.554	0.562	0.791	0.513	0.185	0.484	0.557
**superpathway of purine nucleotides de novo biosynthesis I**	0.509	0.459	0.530	**0.022**		0.860	0.860	**0.027**	0.053	**0.011**	**0.033**		0.507	0.510	0.786	0.924	0.783	0.099	0.057
**superpathway of purine nucleotides de novo biosynthesis II**	0.521	0.484	0.544	**0.041**		0.993	0.993	**0.034**	0.068	**0.025**	0.074		0.521	0.521	0.868	0.894	0.950	0.131	0.063
**superpathway of pyrimidine deoxyribonucleoside salvage**	0.319	0.296	0.337	0.076		0.824	0.824	0.076	0.152	**0.034**	0.103		0.322	0.316	0.635	0.825	0.687	0.129	0.156
**superpathway of pyrimidine nucleobases salvage**	0.897	0.908	0.883	**0.044**		0.478	0.478	0.105	0.210	**0.014**	**0.042**		0.897	0.891	0.824	0.776	0.461	0.220	0.166
**superpathway of pyrimidine ribonucleosides salvage**	0.434	0.356	0.439	**0.014**		0.736	0.736	**0.025**	**0.049**	**0.006**	**0.019**		0.438	0.431	0.772	0.996	0.668	0.092	0.054
**superpathway of pyrimidine ribonucleotides de novo biosynthesis**	0.500	0.436	0.519	**0.018**		0.832	0.832	**0.024**	**0.048**	**0.009**	**0.027**		0.502	0.498	0.776	0.934	0.754	0.092	0.053
**superpathway of thiamin diphosphate biosynthesis I**	0.381	0.353	0.413	0.086		0.942	0.942	0.069	0.138	**0.045**	0.134		0.381	0.447	0.678	0.786	0.809	0.134	0.138
**tRNA charging**	0.786	0.805	0.780	**0.012**		0.552	0.552	**0.035**	0.069	**0.004**	**0.013**		0.787	0.769	0.527	0.917	0.420	0.063	0.097
**UDP-N-acetyl-D-glucosamine biosynthesis I**	0.505	0.579	0.508	**0.029**		0.391	0.391	0.101	0.203	**0.009**	**0.026**		0.518	0.496	0.850	0.686	0.391	0.226	0.158
**UDP-N-acetylmuramoyl-pentapeptide biosyn.I (meso-diaminopimelate)**	0.789	0.814	0.787	**0.005**		0.638	0.638	**0.014**	**0.028**	**0.002**	**0.006**		0.790	0.785	0.638	0.952	0.531	0.050	**0.041**
**UDP-N-acetylmuramoyl-pentapeptide biosynthesis II (lysine-containing)**	0.791	0.817	0.786	**0.006**		0.609	0.609	**0.017**	**0.034**	**0.002**	**0.006**		0.797	0.786	0.696	0.978	0.529	0.064	**0.044**

**UMP biosynthesis**	0.889	0.910	0.880	**0.020**		0.562	0.562	**0.047**	0.094	**0.007**	**0.020**		0.891	0.870	0.507	0.891	0.420	0.072	0.126

Key: CTR=unaffected control; MS =multiple sclerosis; ADS = monophasic acquired demyelinating syndromes
p-u & p-a = unadjusted and adjusted p-values derived from the Kruskal−Wallis rank test. **Bold indicates p<0.05** (reached nominal significance). Two tests were performed for each pathway for the following comparisons: 1. Unaffected controls vs ADS vs MS cases (3 groups); 2. Unaffected controls vs ADS vs DMD exposed and DMD naïve MS cases (4 groups). All pairwise comparisons derived from these two tests, adjusting for the multiple pairwise comparisons made within each test, using the Holm method. P-values not shown if all p>0.05 for a single column (comparison).

Median relative pathway abundances are from metagenomic predictions, derived from a validated algorithm via PICRUSt2 (Phylogenetic Investigation of Communities by Reconstruction of Unobserved States) and summarized as metabolic pathways using MetaCyc (database of metabolic pathways and enzymes from all domains of life).

Possibility of chance findings: While adjustments were made for the multiple pairwise comparisons (within each of the two 3 or 4 group based analyses), no overall adjustments made for multiple different pathways assessed. In total, 193 metagenomic predicted pathways were assessed using two tests, totalling 193 x 2= 386 such that approximately 19 (i.e. 1 in 20) would result in a 'significant' p-value by chance alone.

Summary of findings for the predicted metabolic pathway relative abundances:

Relative to the controls, 8 pathways were lower for the MS cases (e.g., ***L-lysine biosynthesis III; methylerythritol phosphate pathway II; NAD salvage pathway I)*** and 5 were higher (these were: ***pyrimidine deoxyribonucleotide phosphorylation; superpathway of guanosine nucleotides de novo biosynthesis I &II; and superpathway of pyrimidine ribonucleosides salvage (& de novo biosynthesis);*** all adjusted p<0.05.
For the subgroup comparisons by DMD status, while 12 pathways were lower for the DMD naive MS cases (again relative to controls), none reached significance after adjustment (p-a>0.05; e.g., ****NAD salvage pathway I***). Ten pathways were higher for the DMD naïve MS cases (relative to controls), but only one reached significance after adjustment (***reductive TCA cycle I).*** Five pathways were lower for the DMD exposed MS cases (vs controls), but none reached significance after adjustments (p-a>0.05; e.g., ****NAD salvage pathway I)*** and of the one pathways was lower for the DMD exposed MS cases, but adjusted p-value was >0.05 ***(pyrimidine deoxyribonucleotide phosphorylation).***

The ***NAD salvage pathway I** was the only pathway to be lower for the MS DMD naïve and exposed patients (each relative to the controls), but the adjusted p>0.05.

For the MS cases only, for the DMD exposed and naïve comparisons, two pathways differed (although all p>0.05 after adjustment), one was lower for the DMD exposed MS cases (***reductive TCA cycle I)*** and one was higher ***(superpathway of glycolysis and Entner-Doudoroff).***

Relative to the ADS participants, there were no significant differences when compared to the MS cases (either all MS cases or the DMD naïve MS cases), although several pathways also differed when compared to the DMD exposed MS cases, (although all p>0.05 after adjustment): ***(5Z)-dodec-5-enoate biosynthesis; adenosine deoxyribonucleotides de novo biosynthesis II; guanosine deoxyribonucleotides de novo biosynthesis II; mycolate biosynthesis; oleate biosynthesis IV (anaerobic); palmitoleate biosynthesis I (from (5Z)-dodec-5-enoate); stearate biosynthesis II (bacteria and plants); superpathway of β-D-glucuronide and D-glucuronate degradation; superpathway of fatty acid biosynthesis initiation (E. coli); superpathway of hexuronide and hexuronate degradation.*** In addition, many pathways differed between the ADS and control participants (including after adjustment; p-a>0.05; see Table above).

Supplementary Table 5. Cohort characteristics and associations with the gut microbiota alpha and beta diversity metrics in the Canada-USA cohort.

Complementary, exploratory comparisons were performed on the entire cohort (all participants, regardless of diagnosis, i.e., pediatric onset multiple sclerosis (MS) cases, unaffected controls and monophasic acquired demyelinating syndromes (ADS) in the Canada-USA cohort.

**Characteristic**	**Richness** (number of observed ASVs; Margalef index)	**Evenness** (Shannon)	**Chao1**	**Beta-diversity; %** explained (derived from Rsquared; weighted UniFrac; PERMANOVA)
	median (quartiles)			
**Sex:** girl, n=68	19.4 (16.3, 22.5)	0.682 (0.634, 0.708)	226.0 (173.2, 260.0)	
Boy, n=41	19.6 (15.6, 22.8)	0.684 (0.658, 0.699)	224.5 (194.8, 258.0)	
p-value related	*p=0.675[a]*	*p=0.891[a]*	*p=0.798 [a]*	*0.676%; p=0.611[b]*
**Age at stool sample collection**, years: <12, n=22	20.0 (17.3, 22.5)	0.683 (0.673, 0.717)	225.3 (195.9, 253.1)	
12-16 , n=35	19.4 (16.5, 22.0)	0.674 (0.631, 0.710)	227.8 (165.6, 265.3)	
>16, n=52	19.4 (14.8, 22.6)	0.685 (0.647, 0.704)	213.9 (181.9, 258.5)	
p-value related	*p=0.850[a]*	*p=0.446 [a]*	*p=0.962[a]*	*1.84%; p=0.439[b]*
**Race**: White, n=61	19.7 (16.5, 22.8)	0.684 (0.659 0.715)	218.4 (185.1, 263.3)	
Non-white, n=39	18.0 (15.5, 20.5)	0.675 (0.622, 0.694)	218.4 (171.2, 245.1)	
Unknown, n=9	21.6 (18.4, 22.9)	0.687 (0.648, 0.691)	235.7 (238.1, 258.0)	
p-value (2-groups, white vs non-white)	*p=0.420 [a]*	*p=0.0616[a]*	*p=0.601 [a]*	***5.83%; p=0.001[b]***
**Country of residence** (at stool collection): Canada, n=86	19.9 (15.4, 23.1)	0.684 (0.650, 0.709)	232.1 (179.5, 263.2)	
USA, n=23	17.4 (16.5, 20.0)	0.679 (0.634, 0.697)	207.7 (173.2, 238.1)	
p-value related	*p=0.115[a]*	*p=0.299 [a]*	*p=0.158[a]*	***7.92%; p=0.001[b]***
**Bristol Stool Scale**: Hard (types 1-2), n=24	20.4 (17.7, 24.1)	0.681 (0.651, 0.694)	243.9 (195.0, 285.1)	
Medium (types 3-5), n=76	19.3 (15.5, 22.0)	0.688 (0.649, 0.710)	214.5 (173.4, 255.1)	
Loose (types 6-7), n=5	16.5 (12.7, 19.6)	0.673 (0.635, 0.680)	213.9 (141.0, 218.4)	
Unknown, n=4	22.1 (19.4, 23.9)	0.657 (0.611, 0.683)	244.8	

			(211.8, 281.2)	
p-value (3 groups: hard, medium, loose)	*p=0.110[a]*	*p=0.503[a]*	*p=0.165[a]*	*2.24%; p=0.266[b]*
**BMI**: Overweight/ obese (≥85th percentile): yes, n=16	19.8 (16.6, 23.4)	0.690 (0.683, 0.705)	220.9 (199.0, 259.4)	
No, n=91	19.4 (15.7, 22.5)	0.681 (0.637, 0.707)	226.0 (176.6, 261.0)	
Unknown, n=2	15.5 (13.6, 17.4)	0.530 (0.461, 0.600)	170.6 (151.1, 190.1)	
p-value related (2 groups: yes vs no)	*p=0.637[a]*	*p=0.212[a]*	*p=0.903[a]*	*0.413%; p=0.883[b]*
**Block Kids Screener: dietary intake per day,** < vs ≥median				
Unknown, n=19	*20.1 (18.1, 22.6)*	*0.687 (0.640, 0.705)*	*232.1 (208.0, 267.8)*	
% protein caloric intake: <median, n=45	18.4 (15.0, 22.1)	0.681 (0.649, 0.709)	214.5 (162.1, 254.2)	
≥median, n=45	19.7 (15.8, 22.8)	0.684 (0.644, 0.707)	224.5 (177.6, 263.0)	
p-value related (2 groups: < vs ≥median)	*p=0.572[a]*	*p=0.965[a]*	*p=0.628[a]*	*1.268%; p=0.305[b]*
% fat caloric intake: <median, n=45	19.4 (16.5, 22.3)	0.635 (0.392, 0.677)	218.4 (174.0, 258.0)	
≥median, n=45	19.4 (15.2, 22.6)	0.659 (0.194, 0.684)	218.4 (170.7 263.0)	
p-value related (2 groups: < vs ≥median)	*p=0.981[a]*	*p=0.319[a]*	*p=0.803[a]*	0.767%; *p=0.666[b]*
% carbohydrate caloric intake: <median, n=45	19.4 (15.2, 22.9)	0.686 (0.658, 0.715)	212.6 (171.6, 280.1)	
≥median, n=45	19.4 (15.4, 21.6)	0.677 (0.635, 0.701)	224.5 (174.0, 252.6)	
p-value (2 groups: < vs ≥median)	*p=0.660[a]*	*p=0.254[a]*	*p=0.723[a]*	0.767%; *p=0.772[b]*
Grams of fibre: <median, n=45	14.9 (17.9, 22.0)	0.687 (0.648, 0.712)	209.7 (161.7, 267.5)	
≥median, n=45	16.5 (20.0, 22.6)	0.680 (0.649, 0.705)	233.4 (196.1, 258.0)	
p-value related (2 groups: < vs ≥median)	*p=0.285[a]*	*p=0.586[a]*	*p=0.296[a]*	**2.88%; *p=0.022[b]***
**Other medications or supplement (excl. DMDs) within 30 days prior to stool sample**:[c] yes, n=71	18.2 (14.3, 22.2)	0.684 (0.641, 0.712)	213.7 (159.8, 256.1)	
No, n=38	20.4 (18.9, 22.9)	0.684 (0.647, 0.695)	233.2 (209.8, 277.0)	
p-value related	***p=0.00466[a]***	*p=0.571[a]*	***p=0.0170[a]***	***2.89%; p=0.011[b]***
Any vitamin or dietary supplement:‡ *Yes, n=63*	18.2 (14.2, 21.2)	0.686 (0.648, 0.715)	208.2 (156.9, 249.7)	
No, n=46	20.4 (17.9, 24.0)	0.681 (0.643, 0.692)	234.4 (209.8, 284.6)	
p-value related	***p=0.00205[a]***	*p=0.229[a]*	***p=0.00284[a]***	*1.87%; p=0.06[b]*

Key: ADS= acquired demyelinating syndromes; BMI=body mass index; DMD=disease modifying drugs; MS= multiple sclerosis.
grey shading = not applicable
[a]Group comparisons using the Kruskal-Wallis rank sum test
No pairwise comparisons reached significance (Dunn Kruskal-Wallis test, either with or without Holm adjustment for multiple comparisons, all $p>0.05$)
[b] derived from PERMANOVA (Permutational Multivariate Analysis of Variance, R vegan package; adonis function). % explained, derived from Rsquared
[c]Other medications shown exclude the MS DMDs. All medications were grouped into drug classes according to the World Health Organization's (WHO) Anatomical Therapeutic Chemical (ATC) classification system level 3 (details in Appendix 1).
‡The vitamins or dietary supplements were the most commonly used and the total number of classes are shown which included, by ATC group: A11A multivitamins, combinations; A11CC Vitamin D and analogues; A11E Vitamin B-complex, incl. combinations (B6-B12); A11GA Ascorbic acid (vitamin C); A11HA Vitamin E; A12AA Calcium; B03A Iron preparations; B03BA Vitamin B12 (cyanocobalamin and derivatives); B03BB Folic acid and derivatives; C10AX Other lipid modifying agents (Omega 3); uncategorized Fish Oil.
Diversity metrics shown to 3 significant figures. ASV data were rarefied.

Supplementary Tables 6 and 7. Genus (6.1, 6.2) and species-level (7.1, 7.2) differences in the gut microbial communities by individual amplicon sequence variants (ASVs) for USA-only cohort participants with: pediatric-onset multiple sclerosis (MS) [disease-modifying drug (DMD) exposed and naïve], and unaffected controls, expressed as rate ratios

Table Key: see Supplementary Tables 1-3

Table 6.1 Genus-level differences in the gut microbial communities by individual amplicon sequence variants (ASVs) for USA-only cohort participants with: pediatric-onset multiple sclerosis (MS) and unaffected controls, expressed as rate ratios

		Rate ratios (95%CIs) unadjusted	Rate ratios (95%CIs) age and sex adjusted	
Taxa Identifier (for internal use)	Genus-level findings<br>Shown as: Phylum;Class;Order;Family;Genus (combined # of ASVs)	MS cases (vs controls)	MS cases (vs controls)	MS cases (vs controls)
GENUS_1	Firmicutes;D_2__Bacilli;D_3__Lactobacillales;D_4__Lactobacillaceae;D_5__Lactobacillus (Combined: 49)	5.52(1.98,15.36);p=0.00108	4.24(1.57,11.46);p=0.00436	↑
GENUS_3	Firmicutes;D_2__Bacilli;D_3__Lactobacillales;D_4__Streptococcaceae;D_5__Streptococcus (Combined: 34)	0.83(0.50,1.36)	1.05(0.65,1.68)	
GENUS_4	Firmicutes;D_2__Clostridia;D_3__Clostridiales;D_4__Lachnospiraceae;D_5__Blautia (Combined: 99)	1.31(0.97,1.77)	1.34(0.99,1.81)	
GENUS_5	Verrucomicrobia;D_2__Verrucomicrobiae;D_3__Verrucomicrobiales;D_4__Akkermansiaceae;D_5__Akkermansia (Combined: 55)	1.80(0.48,6.78)	1.93(0.51,7.28)	
GENUS_6	Firmicutes;D_2__Clostridia;D_3__Clostridiales;D_4__Lachnospiraceae;D_5__Coprococcus 3 (Combined: 25)	0.85(0.46,1.58)	0.85(0.46,1.58)	
GENUS_7	Firmicutes;D_2__Clostridia;D_3__Clostridiales;D_4__Lachnospiraceae;D_5__Anaerostipes (Combined: 33)	0.94(0.65,1.37)	1.02(0.71,1.47)	
GENUS_8	Bacteroidetes;D_2__Bacteroidia;D_3__Bacteroidales;D_4__Bacteroidaceae;D_5__Bacteroides (Combined: 282)	0.98(0.70,1.36)	0.97(0.70,1.35)	
GENUS_9	Firmicutes;D_2__Clostridia;D_3__Clostridiales;D_4__Lachnospiraceae;D_5__Dorea (Combined: 34)	0.91(0.59,1.40)	0.93(0.60,1.42)	
GENUS_10	Firmicutes;D_2__Clostridia;D_3__Clostridiales;D_4__Lachnospiraceae;D_5__[Eubacterium] hallii group (Combined: 67)	1.31(0.82,2.10)	1.31(0.81,2.10)	
GENUS_11	Firmicutes;D_2__Clostridia;D_3__Clostridiales;D_4__Lachnospiraceae;D_5__Coprococcus 1 (Combined: 11)	0.68(0.35,1.32)	0.69(0.35,1.35)	
GENUS_12	Firmicutes;D_2__Clostridia;D_3__Clostridiales;D_4__Ruminococcaceae;D_5__Ruminococcaceae UCG-004 (Combined: 14)	0.83(0.47,1.45)	0.82(0.47,1.44)	
GENUS_13	Firmicutes;D_2__Clostridia;D_3__Clostridiales;D_4__Christensenellaceae;D_5__Christensenellaceae R-7 group (Combined: 135)	0.38(0.20,0.73);p=0.00382	0.44(0.23,0.83);p=0.01115	↓
GENUS_14	Bacteroidetes;D_2__Bacteroidia;D_3__Bacteroidales;D_4__Rikenellaceae;D_5__Alistipes (Combined: 48)	1.15(0.66,2.00)	1.20(0.69,2.09)	
GENUS_15	Actinobacteria;D_2__Actinobacteria;D_3__Bifidobacteriales;D_4__Bifidobacteriaceae;D_5__Bifidobacterium (Combined: 39)	4.36(1.90,10.01);p=0.00051		
GENUS_18	Firmicutes;D_2__Clostridia;D_3__Clostridiales;D_4__Ruminococcaceae;D_5__Ruminiclostridium 5 (Combined: 79)	1.71(0.98,3.00)	1.76(1.00,3.08);p=0.04889	↑
GENUS_19	Actinobacteria;D_2__Coriobacteriia;D_3__Coriobacteriales;D_4__Coriobacteriaceae;D_5__Collinsella (Combined: 22)	1.20(0.61,2.37)	1.12(0.57,2.20)	
GENUS_20	Firmicutes;D_2__Clostridia;D_3__Clostridiales;D_4__Lachnospiraceae;D_5__uncultured (Combined: 56)	1.00(0.68,1.45)	0.96(0.66,1.40)	
GENUS_21	Firmicutes;D_2__Clostridia;D_3__Clostridiales;D_4__Ruminococcaceae;D_5__Ruminococcaceae NK4A214 group (Combined: 28)	0.38(0.18,0.83);p=0.01473	0.38(0.18,0.82);p=0.01353	↓

GENUS_22	Firmicutes;D_2__Clostridia;D_3__Clostridiales;D_4__Ruminococcaceae;D_5__Ruminococcus 2 (Combined: 15)	1.01(0.32,3.19)	1.19(0.38,3.74)	
GENUS_23	Firmicutes;D_2__Clostridia;D_3__Clostridiales;D_4__Ruminococcaceae;D_5__[Eubacterium] coprostanoligenes group (Combined: 79)	0.75(0.35,1.58)	0.77(0.36,1.61)	
GENUS_24	Firmicutes;D_2__Negativicutes;D_3__Selenomonadales;D_4__Veillonellaceae;D_5__Dialister (Combined: 22)	2.21(0.77,6.34)	2.07(0.72,5.92)	
GENUS_25	Firmicutes;D_2__Clostridia;D_3__Clostridiales;D_4__Lachnospiraceae;D_5__[Ruminococcus] torques group (Combined: 64)	0.91(0.62,1.34)	0.93(0.65,1.35)	
GENUS_26	Firmicutes;D_2__Clostridia;D_3__Clostridiales;D_4__Ruminococcaceae;D_5__Ruminococcus 1 (Combined: 51)	0.55(0.24,1.25)		
GENUS_27	Firmicutes;D_2__Clostridia;D_3__Clostridiales;D_4__Lachnospiraceae;D_5__Marvinbryantia (Combined: 10)	0.63(0.26,1.54)	0.52(0.22,1.27)	
GENUS_28	Firmicutes;D_2__Clostridia;D_3__Clostridiales;D_4__Ruminococcaceae;D_5__Ruminococcaceae UCG-005 (Combined: 62)	0.49(0.24,1.03)	0.51(0.25,1.05)	
GENUS_30	Firmicutes;D_2__Negativicutes;D_3__Selenomonadales;D_4__Acidaminococcaceae;D_5__Phascolarctobacterium (Combined: 18)	0.86(0.27,2.75)	0.81(0.25,2.59)	
GENUS_31	Firmicutes;D_2__Clostridia;D_3__Clostridiales;D_4__Lachnospiraceae;D_5__Agathobacter (Combined: 51)	1.51(0.91,2.50)	1.52(0.92,2.52)	
GENUS_32	Firmicutes;D_2__Clostridia;D_3__Clostridiales;D_4__Ruminococcaceae;D_5__Faecalibacterium (Combined: 131)	1.44(0.93,2.22)	1.36(0.88,2.09)	
GENUS_33	Firmicutes;D_2__Clostridia;D_3__Clostridiales;D_4__Ruminococcaceae;D_5__Intestinimonas (Combined: 17)	0.79(0.30,2.06)		
GENUS_34	Firmicutes;D_2__Clostridia;D_3__Clostridiales;D_4__Lachnospiraceae;D_5__[Ruminococcus] gauvreauii group (Combined: 24)	0.65(0.34,1.26)	0.67(0.35,1.30)	
GENUS_35	Firmicutes;D_2__Clostridia;D_3__Clostridiales;D_4__Ruminococcaceae;D_5__Subdoligranulum (Combined: 61)	0.68(0.42,1.09)	0.67(0.42,1.09)	
GENUS_36	Bacteroidetes;D_2__Bacteroidia;D_3__Bacteroidales;D_4__Tannerellaceae;D_5__Parabacteroides (Combined: 75)	1.10(0.62,1.95)	1.08(0.61,1.91)	
GENUS_37	Firmicutes;D_2__Bacilli;D_3__Lactobacillales;D_4__Leuconostocaceae;D_5__Weissella (Combined: 3)		3.27(0.51,21.12)	
GENUS_38	Firmicutes;D_2__Clostridia;D_3__Clostridiales;D_4__Family XIII;D_5__[Eubacterium] nodatum group (Combined: 12)	1.79(0.50,6.35)	2.58(0.76,8.72)	
GENUS_41	Firmicutes;D_2__Clostridia;D_3__Clostridiales;D_4__Ruminococcaceae;D_5__uncultured (Combined: 88)	0.65(0.33,1.30)	0.58(0.31,1.09)	
GENUS_42	Firmicutes;D_2__Clostridia;D_3__Clostridiales;D_4__Family XIII;D_5__Family XIII AD3011 group (Combined: 40)	0.86(0.47,1.59)	0.91(0.49,1.67)	
GENUS_43	Firmicutes;D_2__Clostridia;D_3__Clostridiales;D_4__Lachnospiraceae;D_5__Eisenbergiella (Combined: 14)	0.53(0.24,1.16)		
GENUS_44	Firmicutes;D_2__Bacilli;D_3__Bacillales;D_4__Family XI;D_5__Gemella (Combined: 3)	1.15(0.51,2.59)	1.10(0.49,2.45)	
GENUS_45	Firmicutes;D_2__Clostridia;D_3__Clostridiales;D_4__Lachnospiraceae;D_5__Fusicatenibacter (Combined: 27)	0.92(0.58,1.47)	0.89(0.56,1.43)	
GENUS_46	Firmicutes;D_2__Clostridia;D_3__Clostridiales;D_4__Lachnospiraceae;D_5__Coprococcus 2 (Combined: 12)	0.85(0.14,5.26)		
GENUS_47	Firmicutes;D_2__Clostridia;D_3__Clostridiales;D_4__Ruminococcaceae;D_5__Ruminiclostridium 9 (Combined: 29)	0.98(0.63,1.54)	1.02(0.65,1.59)	
GENUS_48	Firmicutes;D_2__Clostridia;D_3__Clostridiales;D_4__Peptococcaceae;D_5__Peptococcus (Combined: 13)	4.36(0.91,20.85)	3.88(0.88,17.06)	
GENUS_49	Firmicutes;D_2__Clostridia;D_3__Clostridiales;D_4__Peptostreptococcaceae;D_5__Romboutsia (Combined: 4)	1.48(0.85,2.58)	1.63(0.93,2.83)	
GENUS_50	Bacteroidetes;D_2__Bacteroidia;D_3__Bacteroidales;D_4__Marinifilaceae;D_5__Odoribacter (Combined: 16)	1.28(0.53,3.08)	1.21(0.50,2.90)	
GENUS_51	Firmicutes;D_2__Clostridia;D_3__Clostridiales;D_4__Lachnospiraceae;D_5__Roseburia (Combined: 32)	0.64(0.37,1.14)	0.68(0.38,1.20)	
GENUS_52	Firmicutes;D_2__Clostridia;D_3__Clostridiales;D_4__Ruminococcaceae;D_5__Ruminococcaceae UCG-002 (Combined: 24)	0.57(0.30,1.09)	0.60(0.31,1.14)	
GENUS_53	Firmicutes;D_2__Clostridia;D_3__Clostridiales;D_4__Ruminococcaceae;D_5__UBA1819 (Combined: 7)	0.56(0.29,1.06)	0.56(0.30,1.05)	

GENUS_54	Firmicutes;D_2__Erysipelotrichia;D_3__Erysipelotrichales;D_4__Erysipelotrichaceae;D_5__[Clostridium] innocuum group (Combined: 5)	1.77(0.85,3.67)	1.76(0.85,3.65)	
GENUS_55	Actinobacteria;D_2__Coriobacteriia;D_3__Coriobacteriales;D_4__Eggerthellaceae;D_5__Slackia (Combined: 7)	0.91(0.19,4.30)		
GENUS_56	Firmicutes;D_2__Clostridia;D_3__Clostridiales;D_4__Ruminococcaceae;D_5__Ruminococcaceae UCG-013 (Combined: 53)	1.07(0.66,1.73)	1.08(0.67,1.75)	
GENUS_57	Firmicutes;D_2__Clostridia;D_3__Clostridiales;D_4__Lachnospiraceae;D_5__Hungatella (Combined: 6)	0.19(0.05,0.71);p=0.01358	0.38(0.11,1.39)	
GENUS_58	Firmicutes;D_2__Clostridia;D_3__Clostridiales;D_4__Lachnospiraceae;D_5__Lachnospiraceae FCS020 group (Combined: 16)	0.56(0.32,1.01)	0.53(0.30,0.94);p=0.03017	↓
GENUS_59	Firmicutes;D_2__Negativicutes;D_3__Selenomonadales;D_4__Veillonellaceae;D_5__Veillonella (Combined: 18)	1.15(0.52,2.53)	0.85(0.39,1.85)	
GENUS_60	Firmicutes;D_2__Clostridia;D_3__Clostridiales;D_4__Lachnospiraceae;D_5__GCA-900066575 (Combined: 21)	1.15(0.70,1.89)	1.20(0.73,1.97)	
GENUS_61	Firmicutes;D_2__Clostridia;D_3__Clostridiales;D_4__Ruminococcaceae;D_5__Ruminiclostridium 6 (Combined: 10)	2.42(0.84,7.01)	2.11(0.73,6.10)	
GENUS_62	Firmicutes;D_2__Clostridia;D_3__Clostridiales;D_4__Lachnospiraceae;D_5__CAG-56 (Combined: 16)	0.60(0.22,1.64)	0.63(0.23,1.71)	
GENUS_63	Firmicutes;D_2__Clostridia;D_3__Clostridiales;D_4__Ruminococcaceae;D_5__Negativibacillus (Combined: 8)	1.65(0.58,4.71)	1.68(0.59,4.75)	
GENUS_65	Firmicutes;D_2__Clostridia;D_3__Clostridiales;D_4__Ruminococcaceae (Combined: 126)	0.75(0.37,1.49)	0.72(0.36,1.44)	
GENUS_66	Firmicutes;D_2__Clostridia;D_3__Clostridiales;D_4__Family XIII;D_5__Family XIII UCG-001 (Combined: 9)	0.89(0.47,1.69)	1.01(0.55,1.86)	
GENUS_67	Firmicutes;D_2__Clostridia;D_3__Clostridiales;D_4__Lachnospiraceae (Combined: 215)	0.53(0.34,0.83);p=0.00582	0.57(0.37,0.88);p=0.01087	↓
GENUS_70	Actinobacteria;D_2__Actinobacteria;D_3__Actinomycetales;D_4__Actinomycetaceae;D_5__Actinomyces (Combined: 17)	1.19(0.72,1.97)	1.20(0.73,1.98)	
GENUS_71	Firmicutes;D_2__Clostridia;D_3__Clostridiales;D_4__Family XIII;D_5__[Eubacterium] brachy group (Combined: 10)	1.65(0.73,3.73)	1.65(0.73,3.73)	
GENUS_72	Firmicutes;D_2__Clostridia;D_3__Clostridiales;D_4__Lachnospiraceae;D_5__Lachnoclostridium (Combined: 69)	0.97(0.61,1.53)	1.08(0.70,1.68)	
GENUS_73	Firmicutes;D_2__Clostridia;D_3__Clostridiales;D_4__Lachnospiraceae;D_5__[Eubacterium] eligens group (Combined: 28)	0.91(0.42,1.98)	0.81(0.38,1.76)	
GENUS_74	Firmicutes;D_2__Bacilli;D_3__Lactobacillales;D_4__Carnobacteriaceae;D_5__Granulicatella (Combined: 3)	1.61(0.76,3.42)	1.52(0.72,3.20)	
GENUS_75	Firmicutes;D_2__Clostridia;D_3__Clostridiales;D_4__Ruminococcaceae;D_5__Ruminococcaceae UCG-010 (Combined: 65)	0.41(0.14,1.18)	0.39(0.14,1.12)	
GENUS_76	Firmicutes;D_2__Bacilli;D_3__Lactobacillales;D_4__Streptococcaceae;D_5__Lactococcus (Combined: 9)	0.86(0.43,1.70)	0.86(0.44,1.69)	
GENUS_78	Firmicutes;D_2__Clostridia;D_3__Clostridiales;D_4__Lachnospiraceae;D_5__Lachnospiraceae NK4A136 group (Combined: 44)	0.69(0.41,1.16)	0.70(0.41,1.18)	
GENUS_79	Firmicutes;D_2__Erysipelotrichia;D_3__Erysipelotrichales;D_4__Erysipelotrichaceae;D_5__Erysipelotrichaceae UCG-003 (Combined: 11)	0.74(0.38,1.44)	0.74(0.38,1.44)	
GENUS_82	Firmicutes;D_2__Clostridia;D_3__Clostridiales;D_4__Defluviitaleaceae;D_5__Defluviitaleaceae UCG-011 (Combined: 12)		1.15(0.47,2.78)	
GENUS_83	Firmicutes;D_2__Clostridia;D_3__Clostridiales;D_4__Clostridiales vadinBB60 group;D_5__uncultured Thermoanaerobacterales bacterium (Combined: 2)	5.16(0.70,38.27)		
GENUS_84	Firmicutes;D_2__Erysipelotrichia;D_3__Erysipelotrichales;D_4__Erysipelotrichaceae;D_5__Holdemania (Combined: 12)	1.00(0.61,1.64)	1.02(0.62,1.68)	
GENUS_85	Firmicutes;D_2__Clostridia;D_3__Clostridiales;D_4__Lachnospiraceae;D_5__Sellimonas (Combined: 13)	2.04(0.67,6.22)	1.63(0.54,4.97)	
GENUS_86	Firmicutes;D_2__Clostridia;D_3__Clostridiales;D_4__Christensenellaceae;D_5__uncultured (Combined: 32)	0.52(0.25,1.07)	0.68(0.35,1.34)	
GENUS_87	Proteobacteria;D_2__Gammaproteobacteria;D_3__Betaproteobacteriales;D_4__Burkholderiaceae;D_5__Oxalobacter (Combined: 4)		2.61(0.49,13.83)	

GENUS_88	Firmicutes;D_2__Clostridia;D_3__Clostridiales;D_4__Ruminococcaceae;D_5__DTU089 (Combined: 4)	0.96(0.50,1.86)	0.98(0.51,1.88)	
GENUS_89	Actinobacteria;D_2__Coriobacteriia;D_3__Coriobacteriales;D_4__Coriobacteriales Incertae Sedis;D_5__uncultured (Combined: 11)	0.66(0.23,1.87)	0.78(0.28,2.14)	
GENUS_90	Firmicutes;D_2__Clostridia;D_3__Clostridiales;D_4__Ruminococcaceae;D_5__Anaerotruncus (Combined: 8)	0.53(0.24,1.20)	0.62(0.28,1.39)	
GENUS_91	Actinobacteria;D_2__Coriobacteriia;D_3__Coriobacteriales;D_4__Eggerthellaceae (Combined: 8)	0.34(0.13,0.86);p=0.02338	0.48(0.20,1.17)	
GENUS_92	Firmicutes;D_2__Clostridia;D_3__Clostridiales;D_4__Lachnospiraceae;D_5__[Eubacterium] ventriosum group (Combined: 30)	0.97(0.54,1.74)	0.87(0.48,1.55)	
GENUS_93	Proteobacteria;D_2__Gammaproteobacteria;D_3__Pseudomonadales;D_4__Pseudomonadaceae;D_5__Pseudomonas (Combined: 9)	0.13(0.02,0.70);p=0.0176	0.16(0.03,0.85);p=0.03199	↓
GENUS_94	Firmicutes;D_2__Clostridia;D_3__Clostridiales;D_4__Ruminococcaceae;D_5__Fournierella (Combined: 13)	1.28(0.45,3.70)	1.12(0.39,3.23)	
GENUS_95	Firmicutes;D_2__Clostridia;D_3__Clostridiales;D_4__Ruminococcaceae;D_5__Angelakisella (Combined: 6)	0.56(0.25,1.27)	0.66(0.30,1.48)	
GENUS_96	Firmicutes;D_2__Erysipelotrichia;D_3__Erysipelotrichales;D_4__Erysipelotrichaceae;D_5__Erysipelatoclostridium (Combined: 9)	0.99(0.46,2.15)	0.99(0.46,2.14)	
GENUS_97	Actinobacteria;D_2__Coriobacteriia;D_3__Coriobacteriales;D_4__Eggerthellaceae;D_5__Gordonibacter (Combined: 10)	0.63(0.33,1.20)	0.65(0.35,1.24)	
GENUS_98	Firmicutes;D_2__Clostridia;D_3__Clostridiales;D_4__Lachnospiraceae;D_5__Lachnospiraceae UCG-010 (Combined: 18)	1.14(0.51,2.55)	1.22(0.55,2.70)	
GENUS_99	Firmicutes;D_2__Clostridia;D_3__Clostridiales;D_4__Lachnospiraceae;D_5__[Eubacterium] xylanophilum group (Combined: 20)	1.02(0.37,2.81)	1.29(0.47,3.56)	
GENUS_100	Firmicutes;D_2__Clostridia;D_3__Clostridiales;D_4__Lachnospiraceae;D_5__GCA-900066755 (Combined: 2)	1.01(0.34,3.01)	0.80(0.28,2.30)	
GENUS_101	Actinobacteria;D_2__Coriobacteriia;D_3__Coriobacteriales;D_4__Eggerthellaceae;D_5__Adlercreutzia (Combined: 2)	0.85(0.40,1.80)	0.73(0.34,1.56)	
GENUS_102	Firmicutes;D_2__Clostridia;D_3__DTU014;D_4__uncultured bacterium;D_5__ (Combined: 2)	1.69(0.37,7.69)	1.11(0.25,4.86)	
GENUS_103	Firmicutes;D_2__Clostridia;D_3__Clostridiales;D_4__Ruminococcaceae;D_5__Ruminococcaceae UCG-003 (Combined: 12)	0.53(0.26,1.09)	0.53(0.26,1.10)	
GENUS_104	Firmicutes;D_2__Clostridia;D_3__Clostridiales;D_4__Ruminococcaceae;D_5__Hydrogenoanaerobacterium (Combined: 5)	1.17(0.29,4.76)	1.10(0.27,4.43)	
GENUS_105	Firmicutes;D_2__Clostridia;D_3__Clostridiales;D_4__Christensenellaceae;D_5__Catabacter (Combined: 6)	0.37(0.09,1.49)	1.40(0.39,5.07)	
GENUS_106	Firmicutes;D_2__Clostridia;D_3__Clostridiales;D_4__Ruminococcaceae;D_5__Ruminococcaceae UCG-007 (Combined: 2)	1.72(0.29,10.24)	1.89(0.31,11.29)	
GENUS_108	Proteobacteria;D_2__Deltaproteobacteria;D_3__Desulfovibrionales;D_4__Desulfovibrionaceae;D_5__Desulfovibrio (Combined: 11)	2.67(0.39,18.14)		
GENUS_109	Firmicutes;D_2__Erysipelotrichia;D_3__Erysipelotrichales;D_4__Erysipelotrichaceae;D_5__Dielma (Combined: 5)	2.19(0.82,5.88)	2.13(0.81,5.63)	
GENUS_110	Firmicutes;D_2__Clostridia;D_3__Clostridiales;D_4__Lachnospiraceae;D_5__Lachnospira (Combined: 35)	0.76(0.42,1.38)	0.73(0.40,1.32)	
GENUS_111	Firmicutes;D_2__Bacilli;D_3__Lactobacillales;D_4__Leuconostocaceae;D_5__Leuconostoc (Combined: 7)	0.88(0.23,3.40)	0.84(0.22,3.25)	
GENUS_112	Firmicutes;D_2__Bacilli;D_3__Bacillales;D_4__Staphylococcaceae;D_5__Staphylococcus (Combined: 4)	3.73(0.69,20.23)	5.50(1.07,28.22);p=0.04104	↑
GENUS_113	Firmicutes;D_2__Clostridia;D_3__Clostridiales;D_4__Ruminococcaceae;D_5__Candidatus Soleaferrea (Combined: 20)	0.81(0.48,1.36)	0.78(0.47,1.31)	
GENUS_114	Firmicutes;D_2__Clostridia;D_3__Clostridiales;D_4__Family XIII;D_5__Mogibacterium (Combined: 5)	0.74(0.27,2.01)	0.72(0.27,1.94)	
GENUS_115	Firmicutes;D_2__Clostridia;D_3__Clostridiales;D_4__Clostridiales vadinBB60 group;D_5__uncultured bacterium (Combined: 38)	0.82(0.26,2.58)	0.87(0.29,2.65)	
GENUS_116	Patescibacteria;D_2__Saccharimonadia;D_3__Saccharimonadales;D_4__Saccharimonadaceae (Combined: 1)	2.96(1.11,7.88);p=0.02975	2.95(1.12,7.79);p=0.02899	↑
GENUS_117	Actinobacteria;D_2__Coriobacteriia;D_3__Coriobacteriales;D_4__Eggerthellaceae;D_5__Enterorhabdus (Combined: 9)	2.72(0.62,11.95)	2.72(0.62,11.93)	

GENUS_119	Proteobacteria;D_2__Gammaproteobacteria;D_3__Betaproteobacteriales;D_4__Burkholderiaceae;D_5__Parasutterella (Combined: 15)	0.51(0.19,1.34)	0.52(0.20,1.39)	
GENUS_120	Firmicutes;D_2__Clostridia;D_3__Clostridiales;D_4__Lachnospiraceae;D_5__Lachnospiraceae ND3007 group (Combined: 16)	0.53(0.26,1.11)		
GENUS_121	Bacteroidetes;D_2__Bacteroidia;D_3__Bacteroidales;D_4__Barnesiellaceae;D_5__Coprobacter (Combined: 7)	1.77(0.18,17.56)	1.63(0.17,16.02)	
GENUS_122	Firmicutes;D_2__Clostridia;D_3__Clostridiales;D_4__Ruminococcaceae;D_5__Butyricicoccus (Combined: 41)	1.01(0.66,1.55)	1.06(0.69,1.62)	
GENUS_124	Firmicutes;D_2__Clostridia;D_3__Clostridiales;D_4__Ruminococcaceae;D_5__Oscillibacter (Combined: 28)	1.00(0.59,1.69)	0.98(0.58,1.66)	
GENUS_126	Firmicutes;D_2__Clostridia;D_3__Clostridiales;D_4__Lachnospiraceae;D_5__Lachnospiraceae UCG-008 (Combined: 6)	1.10(0.54,2.23)	1.07(0.53,2.16)	
GENUS_127	Firmicutes;D_2__Clostridia;D_3__Clostridiales;D_4__Lachnospiraceae;D_5__Lachnospiraceae UCG-001 (Combined: 23)	0.50(0.16,1.57)	0.47(0.15,1.49)	
GENUS_128	Firmicutes;D_2__Clostridia;D_3__Clostridiales;D_4__Lachnospiraceae;D_5__Lachnospiraceae UCG-004 (Combined: 29)	1.48(0.77,2.88)	1.30(0.67,2.52)	
GENUS_129	Firmicutes;D_2__Clostridia;D_3__Clostridiales;D_4__Clostridiales vadinBB60 group;D_5__gut metagenome (Combined: 14)	0.89(0.32,2.47)	0.95(0.37,2.48)	
GENUS_130	Lentisphaerae;D_2__Lentisphaeria;D_3__Victivallales;D_4__Victivallaceae;D_5__Victivallis (Combined: 6)	4.93(0.81,30.05)		
GENUS_131	Firmicutes;D_2__Clostridia;D_3__Clostridiales;D_4__Ruminococcaceae;D_5__Flavonifractor (Combined: 17)	0.98(0.58,1.66)	0.99(0.58,1.68)	
GENUS_134	Firmicutes;D_2__Clostridia;D_3__Clostridiales;D_4__Lachnospiraceae;D_5__Moryella (Combined: 3)	0.98(0.43,2.23)	0.83(0.37,1.87)	
GENUS_135	Firmicutes;D_2__Clostridia;D_3__Clostridiales;D_4__Ruminococcaceae;D_5__Ruminococcaceae UCG-009 (Combined: 13)	1.68(0.50,5.58)	1.20(0.36,3.96)	
GENUS_137	Proteobacteria;D_2__Gammaproteobacteria;D_3__Pasteurellales;D_4__Pasteurellaceae;D_5__Haemophilus (Combined: 3)	0.79(0.31,2.01)	0.80(0.32,2.03)	
GENUS_139	Firmicutes;D_2__Clostridia;D_3__Clostridiales;D_4__Ruminococcaceae;D_5__Oscillospira (Combined: 12)	0.74(0.31,1.79)	0.66(0.28,1.58)	
GENUS_140	Firmicutes;D_2__Clostridia;D_3__Clostridiales;D_4__Clostridiaceae 1;D_5__Clostridium sensu stricto 1 (Combined: 22)	1.62(0.83,3.18)	1.56(0.80,3.06)	
GENUS_141	Firmicutes;D_2__Clostridia;D_3__Clostridiales;D_4__Ruminococcaceae;D_5__Phocea (Combined: 3)	1.44(0.56,3.66)	1.23(0.49,3.10)	
GENUS_142	Proteobacteria;D_2__Gammaproteobacteria;D_3__Enterobacteriales;D_4__Enterobacteriaceae;D_5__Escherichia-Shigella (Combined: 10)	1.08(0.39,3.05)		
GENUS_143	Firmicutes;D_2__Clostridia;D_3__Clostridiales (Combined: 29)	0.85(0.16,4.51)	0.94(0.18,4.86)	
GENUS_144	Actinobacteria;D_2__Coriobacteriia;D_3__Coriobacteriales;D_4__Eggerthellaceae;D_5__Eggerthella (Combined: 3)	1.19(0.56,2.50)	1.42(0.69,2.94)	
GENUS_145	Firmicutes;D_2__Clostridia;D_3__Clostridiales;D_4__Ruminococcaceae;D_5__Ruminiclostridium (Combined: 7)	0.94(0.33,2.66)	0.95(0.35,2.62)	
GENUS_146	Firmicutes;D_2__Clostridia;D_3__Clostridiales;D_4__Lachnospiraceae;D_5__Anaerosporobacter (Combined: 3)	1.65(0.34,7.97)	0.95(0.20,4.53)	
GENUS_147	Firmicutes;D_2__Clostridia;D_3__Clostridiales;D_4__Ruminococcaceae;D_5__GCA-900066225 (Combined: 20)	0.99(0.35,2.76)		
GENUS_148	Firmicutes;D_2__Clostridia;D_3__Clostridiales;D_4__Ruminococcaceae;D_5__Pseudoflavonifractor (Combined: 3)	0.60(0.11,3.13)	0.34(0.07,1.59)	
GENUS_149	Firmicutes;D_2__Clostridia;D_3__Clostridiales;D_4__Lachnospiraceae;D_5__Tyzzerella (Combined: 7)	7.76(1.77,34.05);p=0.00662	8.82(2.01,38.68);p=0.00389	↑
GENUS_152	Proteobacteria;D_2__Gammaproteobacteria;D_3__Betaproteobacteriales;D_4__Burkholderiaceae;D_5__Noviherbaspirillum (Combined: 4)	0.59(0.16,2.24)	0.47(0.13,1.78)	
GENUS_153	Proteobacteria;D_2__Gammaproteobacteria;D_3__Betaproteobacteriales;D_4__Burkholderiaceae;D_5__Sutterella (Combined: 37)	1.89(0.66,5.35)	1.50(0.53,4.22)	
GENUS_154	Firmicutes;D_2__Clostridia;D_3__Clostridiales;D_4__Peptostreptococcaceae;D_5__Intestinibacter (Combined: 5)	0.62(0.35,1.10)	0.77(0.45,1.32)	
GENUS_155	Cyanobacteria;D_2__Oxyphotobacteria;D_3__Chloroplast (Combined: 12)	2.44(0.45,13.31)	2.86(0.58,14.17)	

GENUS_156	Firmicutes;D_2__Clostridia;D_3__Clostridiales;D_4__Peptostreptococcaceae;D_5__Terrisporobacter (Combined: 2)	0.68(0.27,1.70)	0.84(0.34,2.05)	
GENUS_157	Proteobacteria;D_2__Alphaproteobacteria;D_3__Caulobacterales;D_4__Caulobacteraceae;D_5__Brevundimonas (Combined: 1)	0.48(0.08,2.81)	0.51(0.09,2.98)	
GENUS_160	Actinobacteria;D_2__Actinobacteria;D_3__Micrococcales;D_4__Micrococcaceae;D_5__Rothia (Combined: 9)	2.25(0.79,6.38)	2.74(1.00,7.50)	
GENUS_162	Proteobacteria;D_2__Gammaproteobacteria;D_3__Pasteurellales;D_4__Pasteurellaceae (Combined: 1)	0.39(0.07,2.24)	0.57(0.11,3.03)	
GENUS_163	Firmicutes;D_2__Clostridia;D_3__Clostridiales;D_4__Lachnospiraceae;D_5__[Ruminococcus] gnavus group (Combined: 9)	1.06(0.40,2.82)	1.18(0.45,3.10)	
GENUS_165	Proteobacteria;D_2__Gammaproteobacteria;D_3__Betaproteobacteriales;D_4__Neisseriaceae;D_5__Neisseria (Combined: 5)	0.29(0.02,3.34)		
GENUS_166	Tenericutes;D_2__Mollicutes;D_3__Mollicutes RF39 (Combined: 22)	0.06(0.00,1.45)	0.05(0.00,1.18)	
GENUS_167	Proteobacteria;D_2__Gammaproteobacteria;D_3__Enterobacteriales;D_4__Enterobacteriaceae (Combined: 15)	2.89(0.64,13.19)	3.18(0.70,14.34)	
GENUS_168	Firmicutes;D_2__Erysipelotrichia;D_3__Erysipelotrichales;D_4__Erysipelotrichaceae;D_5__Turicibacter (Combined: 20)	1.18(0.61,2.28)	1.25(0.65,2.41)	
GENUS_174	Firmicutes;D_2__Erysipelotrichia;D_3__Erysipelotrichales;D_4__Erysipelotrichaceae;D_5__Merdibacter (Combined: 3)	0.87(0.22,3.39)	0.67(0.18,2.46)	
GENUS_175	Firmicutes;D_2__Clostridia;D_3__Clostridiales;D_4__Ruminococcaceae;D_5__Anaerofilum (Combined: 8)	1.03(0.29,3.70)	0.82(0.24,2.81)	
GENUS_176	Actinobacteria;D_2__Coriobacteriia;D_3__Coriobacteriales;D_4__Coriobacteriales Incertae Sedis;D_5__Raoultibacter (Combined: 2)	0.89(0.10,7.64)	0.83(0.10,7.13)	
GENUS_177	Firmicutes;D_2__Clostridia;D_3__Clostridiales;D_4__Eubacteriaceae;D_5__Eubacterium (Combined: 2)	1.05(0.21,5.17)	1.30(0.28,6.07)	
GENUS_178	Firmicutes;D_2__Erysipelotrichia;D_3__Erysipelotrichales;D_4__Erysipelotrichaceae;D_5__Solobacterium (Combined: 2)	0.53(0.08,3.67)	1.76(0.33,9.34)	
GENUS_179	Firmicutes;D_2__Erysipelotrichia;D_3__Erysipelotrichales;D_4__Erysipelotrichaceae;D_5__uncultured (Combined: 2)	2.99(0.44,20.24)	3.22(0.50,20.88)	
GENUS_180	Proteobacteria;D_2__Gammaproteobacteria;D_3__Betaproteobacteriales;D_4__Burkholderiaceae;D_5__Variovorax (Combined: 1)		0.93(0.31,2.81)	
GENUS_182	Firmicutes;D_2__Clostridia;D_3__Clostridiales;D_4__Lachnospiraceae;D_5__[Eubacterium] fissicatena group (Combined: 8)	0.63(0.29,1.36)	0.63(0.29,1.37)	
GENUS_183	Firmicutes;D_2__Clostridia;D_3__Clostridiales;D_4__Lachnospiraceae;D_5__UC5-1-2E3 (Combined: 1)		1.22(0.19,7.89)	
GENUS_184	Bacteroidetes;D_2__Bacteroidia;D_3__Bacteroidales;D_4__Prevotellaceae;D_5__Paraprevotella (Combined: 20)	0.75(0.09,6.22)	0.90(0.11,7.48)	
GENUS_187	Firmicutes;D_2__Clostridia;D_3__Clostridiales;D_4__Ruminococcaceae;D_5__Acetanaerobacterium (Combined: 2)	0.80(0.32,1.98)	0.65(0.27,1.59)	
GENUS_188	Proteobacteria;D_2__Gammaproteobacteria;D_3__Betaproteobacteriales;D_4__Burkholderiaceae;D_5__Acidovorax (Combined: 1)	2.28(0.31,16.79)	2.27(0.31,16.77)	
GENUS_189	Firmicutes;D_2__Clostridia;D_3__Clostridiales;D_4__Peptostreptococcaceae;D_5__Paeniclostridium (Combined: 2)	7.80(0.65,94.18)		
GENUS_190	Firmicutes;D_2__Bacilli;D_3__Lactobacillales;D_4__Enterococcaceae;D_5__Enterococcus (Combined: 4)	5.19(0.79,34.02)	3.42(0.60,19.63)	
GENUS_192	Actinobacteria;D_2__Actinobacteria;D_3__Bifidobacteriales;D_4__Bifidobacteriaceae;D_5__Alloscardovia (Combined: 1)	3.61(0.23,55.96)	16.03(0.77,332.11)	
GENUS_193	Firmicutes;D_2__Erysipelotrichia;D_3__Erysipelotrichales;D_4__Erysipelotrichaceae;D_5__Faecalitalea (Combined: 5)	0.53(0.14,1.92)	0.65(0.18,2.36)	
GENUS_194	Bacteroidetes;D_2__Bacteroidia;D_3__Bacteroidales;D_4__Prevotellaceae;D_5__Prevotella 6 (Combined: 9)	16.60(2.01,136.73);p=0.00903		
GENUS_195	Bacteroidetes;D_2__Bacteroidia;D_3__Bacteroidales;D_4__Porphyromonadaceae;D_5__Porphyromonas (Combined: 17)	1.51(0.38,6.05)		
GENUS_196	Actinobacteria;D_2__Actinobacteria;D_3__Corynebacteriales;D_4__Corynebacteriaceae;D_5__Corynebacterium 1 (Combined: 8)	1.34(0.14,12.72)	1.67(0.18,15.75)	
GENUS_197	Firmicutes;D_2__Clostridia;D_3__Clostridiales;D_4__Family XI;D_5__Ezakiella (Combined: 4)	0.46(0.07,3.22)	0.51(0.08,3.07)	

GENUS_199	Firmicutes;D_2__Clostridia;D_3__Clostridiales;D_4__Lachnospiraceae;D_5__uncultured organism (Combined: 1)	0.48(0.05,4.21)	0.34(0.04,2.93)	
GENUS_200	Bacteroidetes;D_2__Bacteroidia;D_3__Bacteroidales;D_4__Prevotellaceae;D_5__Prevotella (Combined: 11)	1.15(0.28,4.75)	1.13(0.28,4.52)	
GENUS_202	Proteobacteria;D_2__Gammaproteobacteria;D_3__Betaproteobacteriales;D_4__Burkholderiaceae (Combined: 7)	1.27(0.30,5.33)	1.85(0.47,7.24)	
GENUS_203	Firmicutes;D_2__Clostridia;D_3__Clostridiales;D_4__Family XI;D_5__Peptoniphilus (Combined: 8)	0.37(0.03,4.56)		
GENUS_204	Firmicutes;D_2__Erysipelotrichia;D_3__Erysipelotrichales;D_4__Erysipelotrichaceae;D_5__Candidatus Stoquefichus (Combined: 3)	6.40(0.77,53.16)	9.05(1.09,75.45);p=0.0418	↑
GENUS_205	Firmicutes;D_2__Clostridia;D_3__Clostridiales;D_4__Ruminococcaceae;D_5__Ruminococcaceae UCG-008 (Combined: 7)	3.54(0.56,22.18)	2.96(0.48,18.30)	
GENUS_206	Actinobacteria;D_2__Coriobacteriia;D_3__Coriobacteriales;D_4__Atopobiaceae;D_5__Atopobium (Combined: 8)	1.25(0.31,5.07)	1.78(0.47,6.74)	
GENUS_208	Actinobacteria;D_2__Actinobacteria;D_3__Propionibacteriales;D_4__Propionibacteriaceae;D_5__Cutibacterium (Combined: 1)		0.00(0.00,Inf)	
GENUS_210	Firmicutes;D_2__Clostridia;D_3__Clostridiales;D_4__Lachnospiraceae;D_5__Lachnospiraceae UCG-009 (Combined: 1)	0.17(0.01,1.94)		
GENUS_212	Firmicutes;D_2__Clostridia;D_3__Clostridiales;D_4__Lachnospiraceae;D_5__Shuttleworthia (Combined: 6)	1.77(0.38,8.31)	2.02(0.46,8.76)	
GENUS_214	Firmicutes;D_2__Clostridia;D_3__Clostridiales;D_4__Eubacteriaceae;D_5__Anaerofustis (Combined: 6)	1.27(0.31,5.20)	1.41(0.35,5.71)	
GENUS_217	Firmicutes;D_2__Clostridia;D_3__Clostridiales;D_4__Christensenellaceae;D_5__Christensenella (Combined: 1)	0.34(0.07,1.75)	0.37(0.07,1.87)	
GENUS_218	Firmicutes;D_2__Clostridia;D_3__Clostridiales;D_4__Peptostreptococcaceae (Combined: 6)	0.04(0.00,0.41);p=0.00685	0.04(0.00,0.43);p=0.00746	↓
GENUS_226	Firmicutes;D_2__Bacilli;D_3__Bacillales;D_4__Bacillaceae;D_5__Bacillus (Combined: 4)	26.72(1.54,463.79);p=0.02404		
GENUS_231	Firmicutes;D_2__Clostridia;D_3__Clostridiales;D_4__Family XI;D_5__Parvimonas (Combined: 6)	0.17(0.03,0.89);p=0.03529	0.16(0.04,0.74);p=0.0187	↓
GENUS_234	Firmicutes;D_2__Clostridia;D_3__Clostridiales;D_4__Family XI;D_5__Finegoldia (Combined: 1)	5.91(0.59,59.77)	8.53(0.83,88.15)	
GENUS_235	Firmicutes;D_2__Negativicutes;D_3__Selenomonadales;D_4__Veillonellaceae (Combined: 2)	6.82(0.13,362.38)		
GENUS_236	Firmicutes;D_2__Clostridia;D_3__Clostridiales;D_4__Family XI;D_5__Anaerococcus (Combined: 6)	1.61(0.09,28.08)		
GENUS_239	Actinobacteria;D_2__Actinobacteria;D_3__Bifidobacteriales;D_4__Bifidobacteriaceae;D_5__Scardovia (Combined: 1)	2.26(0.16,31.10)		
GENUS_244	Firmicutes;D_2__Clostridia;D_3__Clostridiales;D_4__Lachnospiraceae;D_5__Lactonifactor (Combined: 2)	0.50(0.11,2.26)	0.48(0.11,2.11)	
GENUS_245	Firmicutes;D_2__Clostridia;D_3__Clostridiales;D_4__Lachnospiraceae;D_5__Lachnospiraceae NC2004 group (Combined: 7)	5.51(0.57,52.95)	4.04(0.43,37.54)	
GENUS_248	Firmicutes;D_2__Erysipelotrichia;D_3__Erysipelotrichales;D_4__Erysipelotrichaceae;D_5__Coprobacillus (Combined: 1)	0.07(0.01,1.05)		
GENUS_249	Firmicutes;D_2__Clostridia;D_3__Clostridiales;D_4__Family XIII;D_5__S5-A14a (Combined: 1)	1.19(0.08,17.16)	2.29(0.17,31.85)	
GENUS_256	Firmicutes;D_2__Clostridia;D_3__Clostridiales;D_4__Ruminococcaceae;D_5__Ruminiclostridium 1 (Combined: 9)	0.45(0.07,3.03)	0.65(0.10,4.06)	
GENUS_258	Firmicutes;D_2__Clostridia;D_3__Clostridiales;D_4__Ruminococcaceae;D_5__Papillibacter (Combined: 2)		1.85(0.51,6.68)	
GENUS_261	Proteobacteria;D_2__Gammaproteobacteria;D_3__Xanthomonadales;D_4__Xanthomonadaceae;D_5__Stenotrophomonas (Combined: 5)	0.25(0.03,2.37)	0.13(0.01,1.33)	
GENUS_262	Firmicutes;D_2__Clostridia;D_3__Clostridiales;D_4__Ruminococcaceae;D_5__uncultured bacterium (Combined: 1)		5.78(0.29,114.51)	
GENUS_263	Firmicutes;D_2__Clostridia;D_3__Clostridiales;D_4__Lachnospiraceae;D_5__Oribacterium (Combined: 8)	36.38(1.52,870.65);p=0.02653		
GENUS_267	Actinobacteria;D_2__Actinobacteria;D_3__Actinomycetales;D_4__Actinomycetaceae;D_5__F0332 (Combined: 2)	2.05(0.33,12.75)		

GENUS_268	Fusobacteria;D_2__Fusobacteriia;D_3__Fusobacteriales;D_4__Fusobacteriaceae;D_5__Fusobacterium (Combined: 13)	0.28(0.01,11.02)		
GENUS_273	Patescibacteria;D_2__Saccharimonadia;D_3__Saccharimonadales;D_4__TM7 phylum sp. oral clone FR058;D_5__ (Combined: 5)	0.87(0.09,8.77)		
GENUS_276	Proteobacteria;D_2__Gammaproteobacteria;D_3__Betaproteobacteriales;D_4__Burkholderiaceae;D_5__Aquabacterium (Combined: 1)	1.89(0.20,17.52)	2.84(0.25,32.67)	
GENUS_284	Epsilonbacteraeota;D_2__Campylobacteria;D_3__Campylobacterales;D_4__Campylobacteraceae;D_5__Campylobacter (Combined: 4)	4.14(0.54,31.61)	4.88(0.62,38.21)	
GENUS_288	Firmicutes;D_2__Clostridia;D_3__Clostridiales;D_4__Lachnospiraceae;D_5__Tyzzerella 4 (Combined: 3)	0.39(0.04,3.46)		
GENUS_289	Fusobacteria;D_2__Fusobacteriia;D_3__Fusobacteriales;D_4__Leptotrichiaceae;D_5__Sneathia (Combined: 2)	0.00(0.00,0.17);p=0.00465		
GENUS_293	Bacteroidetes;D_2__Bacteroidia;D_3__Bacteroidales;D_4__Muribaculaceae;D_5__Porphyromonadaceae bacterium C941 (Combined: 2)	1.05(0.04,30.67)		
GENUS_296	Firmicutes;D_2__Negativicutes;D_3__Selenomonadales;D_4__Veillonellaceae;D_5__Negativicoccus (Combined: 2)	1.17(0.02,67.85)		
GENUS_297	Proteobacteria;D_2__Gammaproteobacteria;D_3__Betaproteobacteriales;D_4__Burkholderiaceae;D_5__Tepidimonas (Combined: 2)	0.47(0.02,10.43)		
GENUS_303	Proteobacteria;D_2__Alphaproteobacteria;D_3__Caulobacterales;D_4__Caulobacteraceae;D_5__Phenylobacterium (Combined: 1)	1.61(0.11,24.40)	1.69(0.11,26.14)	
GENUS_323	Firmicutes;D_2__Clostridia;D_3__Clostridiales;D_4__Lachnospiraceae;D_5__Cuneatibacter (Combined: 1)	1.47(0.06,33.58)	3.53(0.13,99.46)	
GENUS_325	Firmicutes;D_2__Clostridia;D_3__Clostridiales;D_4__Family XIII (Combined: 5)	1.39(0.04,44.45)		
GENUS_330	Proteobacteria;D_2__Gammaproteobacteria;D_3__Betaproteobacteriales;D_4__Burkholderiaceae;D_5__Lautropia (Combined: 2)	11.02(0.24,505.55)		

Table 6.2. Genus-level differences in the gut microbial communities by individual amplicon sequence variants (ASVs) for USA-only cohort participants with: pediatric-onset multiple sclerosis (MS) [disease-modifying drug (DMD) exposed and naïve], and unaffected controls, expressed as rate ratios

		Rate ratios (95%CIs) unadjusted			Rate ratios (95%CIs) age and sex adjusted					
		Ref.: MS cases DMD-	Ref.: Controls		Ref.: MS cases DMD-	Ref.: Controls				
Taxa Identifier (for internal use)	Genus-level findings<br>Shown as: Phylum;Class;Order;Family;Genus (combined # of ASVs)	MS cases DMD+	MS cases DMD-	MS cases DMD+	MS cases DMD+	MS cases DMD-	MS cases DMD+	MS DMD+ (vs MS DMD-)	*MS DMD- (vs control)*	*MS DMD+ (vs control)*
GENUS_1	Firmicutes;D_2__Bacilli;D_3__Lactobacillales;D_4__Lactobacillaceae;D_5__Lactobacillus (Combined: 49)	7.36(1.81,29.93); p=0.00529	1.08(0.28,4.16)	7.94(2.61,24.16);p=0.00026	4.98(1.25,19.83);p=0.02278	1.20(0.32,4.48)	5.97(2.00,17.83);p=0.00136	↑		↑
GENUS_3	Firmicutes;D_2__Bacilli;D_3__Lactobacillales;D_4__Streptococcaceae; D_5__Streptococcus (Combined: 34)	1.31(0.65,2.63)	0.69(0.35,1.34)	0.90(0.52,1.57)	1.65(0.85,3.21)	0.75(0.40,1.41)	1.23(0.73,2.09)			
GENUS_4	Firmicutes;D_2__Clostridia;D_3__Clostridiales;D_4__Lachnospiraceae; D_5__Blautia (Combined: 99)	1.17(0.76,1.79)	1.18(0.79,1.78)	1.38(0.98,1.93)	1.13(0.74,1.73)	1.24(0.82,1.85)	1.39(1.00,1.95)			

GENUS_5	Verrucomicrobia;D_2__Verrucomicrobiae;D_3__Verrucomicrobiales;D_4__Akkermansiaceae;D_5__Akkermansia (Combined: 55)	0.91(0.14,5.86)	1.91(0.32,11.48)	1.74(0.40,7.64)	0.91(0.14,6.00)	2.03(0.34,12.23)	1.86(0.42,8.26)			
GENUS_6	Firmicutes;D_2__Clostridia;D_3__Clostridiales;D_4__Lachnospiraceae;D_5__Coprococcus 3 (Combined: 25)	1.30(0.54,3.11)	0.71(0.31,1.65)	0.93(0.47,1.85)	1.21(0.50,2.89)	0.75(0.33,1.73)	0.91(0.45,1.81)			
GENUS_7	Firmicutes;D_2__Clostridia;D_3__Clostridiales;D_4__Lachnospiraceae;D_5__Anaerostipes (Combined: 33)	1.28(0.76,2.17)	0.80(0.48,1.32)	1.02(0.67,1.55)	1.21(0.72,2.02)	0.90(0.55,1.47)	1.09(0.72,1.63)			
GENUS_8	Bacteroidetes;D_2__Bacteroidia;D_3__Bacteroidales;D_4__Bacteroidaceae;D_5__Bacteroides (Combined: 282)	1.40(0.88,2.23)	0.77(0.49,1.21)	1.09(0.75,1.57)	1.42(0.90,2.26)	0.76(0.49,1.18)	1.08(0.75,1.57)			
GENUS_9	Firmicutes;D_2__Clostridia;D_3__Clostridiales;D_4__Lachnospiraceae;D_5__Dorea (Combined: 34)	1.06(0.58,1.94)	0.87(0.49,1.57)	0.92(0.57,1.50)	1.01(0.55,1.86)	0.92(0.51,1.64)	0.93(0.57,1.51)			
GENUS_10	Firmicutes;D_2__Clostridia;D_3__Clostridiales;D_4__Lachnospiraceae;D_5__[Eubacterium] hallii group (Combined: 67)	1.98(1.03,3.80);p=0.04115	0.80(0.43,1.51)	1.59(0.95,2.67)	2.05(1.06,3.96);p=0.03336	0.79(0.42,1.48)	1.62(0.96,2.73)	↑		
GENUS_11	Firmicutes;D_2__Clostridia;D_3__Clostridiales;D_4__Lachnospiraceae;D_5__Coprococcus 1 (Combined: 11)	0.98(0.38,2.52)	0.68(0.28,1.69)	0.67(0.32,1.42)	0.96(0.37,2.47)	0.71(0.29,1.76)	0.68(0.32,1.45)			
GENUS_12	Firmicutes;D_2__Clostridia;D_3__Clostridiales;D_4__Ruminococcaceae;D_5__Ruminococcaceae UCG-004 (Combined: 14)	1.24(0.56,2.74)	0.71(0.33,1.53)	0.89(0.47,1.66)	1.22(0.55,2.70)	0.72(0.34,1.54)	0.88(0.47,1.65)			
GENUS_13	Firmicutes;D_2__Clostridia;D_3__Clostridiales;D_4__Christensenellaceae;D_5__Christensenellaceae R-7 group (Combined: 135)	0.79(0.32,1.99)	0.44(0.18,1.06)	0.35(0.17,0.72);p=0.00463	0.96(0.39,2.38)	0.45(0.19,1.06)	0.43(0.21,0.88);p=0.02126			↓
GENUS_14	Bacteroidetes;D_2__Bacteroidia;D_3__Bacteroidales;D_4__Rikenellaceae;D_5__Alistipes (Combined: 48)	1.16(0.54,2.53)	1.04(0.49,2.20)	1.21(0.65,2.25)	1.18(0.54,2.58)	1.08(0.51,2.28)	1.27(0.68,2.37)			
GENUS_15	Actinobacteria;D_2__Actinobacteria;D_3__Bifidobacteriales;D_4__Bifidobacteriaceae;D_5__Bifidobacterium (Combined: 39)	19.34(6.42,58.26);p=0	0.34(0.12,0.98);p=0.04547	6.55(2.73,15.73);p=0.00003						
GENUS_18	Firmicutes;D_2__Clostridia;D_3__Clostridiales;D_4__Ruminococcaceae;D_5__Ruminiclostridium 5 (Combined: 79)	3.86(1.80,8.25);p=0.0005	0.60(0.29,1.25)	2.32(1.27,4.24);p=0.0063	4.16(1.94,8.95);p=0.00026	0.60(0.29,1.23)	2.48(1.35,4.54);p=0.00332	↑		↑
GENUS_19	Actinobacteria;D_2__Coriobacteriia;D_3__Coriobacteriales;D_4__Coriobacteriaceae;D_5__Collinsella (Combined: 22)	1.34(0.52,3.48)	0.98(0.39,2.46)	1.32(0.62,2.82)	1.34(0.51,3.50)	0.92(0.37,2.30)	1.23(0.58,2.64)			
GENUS_20	Firmicutes;D_2__Clostridia;D_3__Clostridiales;D_4__Lachnospiraceae;D_5__uncultured (Combined: 56)	1.02(0.60,1.74)	0.98(0.59,1.64)	1.00(0.66,1.53)	0.97(0.57,1.65)	0.99(0.59,1.64)	0.95(0.63,1.45)			
GENUS_21	Firmicutes;D_2__Clostridia;D_3__Clostridiales;D_4__Ruminococcaceae;D_5__Ruminococcaceae NK4A214 group (Combined: 28)	1.19(0.40,3.54)	0.34(0.12,0.97);p=0.04311	0.40(0.17,0.96);p=0.04	1.64(0.56,4.82)	0.27(0.10,0.76);p=0.01325	0.45(0.19,1.05)		↓	
GENUS_23	Firmicutes;D_2__Clostridia;D_3__Clostridiales;D_4__Ruminococcaceae;D_5__[Eubacterium] coprostanoligenes group (Combined: 79)	0.70(0.25,2.01)	0.92(0.34,2.53)	0.65(0.28,1.50)	0.82(0.29,2.36)	0.86(0.32,2.36)	0.71(0.31,1.63)			
GENUS_24	Firmicutes;D_2__Negativicutes;D_3__Selenomonadales;D_4__Veillonellaceae;D_5__Dialister (Combined: 22)	0.13(0.03,0.53);p=0.00477	5.08(1.29,19.99);p=0.02007	0.65(0.21,2.02)	0.13(0.03,0.55);p=0.0055	4.99(1.27,19.60);p=0.02147	0.65(0.21,2.03)	↓	↑	
GENUS_25	Firmicutes;D_2__Clostridia;D_3__Clostridiales;D_4__Lachnospiraceae;D_5__[Ruminococcus] torques group (Combined: 64)	0.90(0.53,1.54)	0.97(0.58,1.63)	0.88(0.57,1.35)	0.74(0.44,1.24)	1.12(0.69,1.83)	0.83(0.55,1.25)			
GENUS_26	Firmicutes;D_2__Clostridia;D_3__Clostridiales;D_4__Ruminococcaceae;D_5__Ruminococcus 1 (Combined: 51)	1.38(0.44,4.33)	0.44(0.15,1.33)	0.61(0.25,1.52)						

GENUS_27	Firmicutes;D_2__Clostridia;D_3__Clostridiales;D_4__Lachnospiraceae;D_5__Marvinbryantia (Combined: 10)	2.01(0.58,7.02)	0.38(0.11,1.27)	0.77(0.29,2.06)	2.11(0.60,7.39)	0.32(0.10,1.04)	0.67(0.25,1.79)			
GENUS_28	Firmicutes;D_2__Clostridia;D_3__Clostridiales;D_4__Ruminococcaceae;D_5__Ruminococcaceae UCG-005 (Combined: 62)	0.88(0.31,2.47)	0.53(0.20,1.44)	0.47(0.21,1.07)	0.98(0.35,2.75)	0.52(0.19,1.37)	0.51(0.22,1.14)			
GENUS_30	Firmicutes;D_2__Negativicutes;D_3__Selenomonadales;D_4__Acidaminococcaceae;D_5__Phascolarctobacterium (Combined: 18)	1.11(0.22,5.63)	0.81(0.17,3.86)	0.89(0.25,3.25)	1.05(0.20,5.43)	0.79(0.16,3.77)	0.83(0.23,3.04)			
GENUS_31	Firmicutes;D_2__Clostridia;D_3__Clostridiales;D_4__Lachnospiraceae;D_5__Agathobacter (Combined: 51)	1.27(0.62,2.58)	1.28(0.65,2.54)	1.63(0.93,2.86)	1.25(0.61,2.54)	1.31(0.67,2.59)	1.64(0.93,2.88)			
GENUS_32	Firmicutes;D_2__Clostridia;D_3__Clostridiales;D_4__Ruminococcaceae;D_5__Faecalibacterium (Combined: 131)	0.39(0.21,0.69);p=0.00142	2.38(1.36,4.18);p=0.00248	0.92(0.58,1.46)	0.36(0.20,0.65);p=0.00064	2.44(1.39,4.27);p=0.00186	0.87(0.55,1.39)	↓	↑	
GENUS_33	Firmicutes;D_2__Clostridia;D_3__Clostridiales;D_4__Ruminococcaceae;D_5__Intestinimonas (Combined: 17)	2.77(0.72,10.60)	0.37(0.10,1.34)	1.02(0.35,2.94)						
GENUS_34	Firmicutes;D_2__Clostridia;D_3__Clostridiales;D_4__Lachnospiraceae;D_5__[Ruminococcus] gauvreauii group (Combined: 24)	0.84(0.33,2.12)	0.73(0.30,1.77)	0.61(0.29,1.28)	0.85(0.33,2.16)	0.74(0.31,1.81)	0.63(0.30,1.32)			
GENUS_35	Firmicutes;D_2__Clostridia;D_3__Clostridiales;D_4__Ruminococcaceae;D_5__Subdoligranulum (Combined: 61)	0.80(0.41,1.57)	0.78(0.41,1.49)	0.62(0.36,1.06)	0.81(0.41,1.61)	0.77(0.40,1.46)	0.62(0.36,1.07)			
GENUS_36	Bacteroidetes;D_2__Bacteroidia;D_3__Bacteroidales;D_4__Tannerellaceae;D_5__Parabacteroides (Combined: 75)	0.74(0.33,1.64)	1.33(0.62,2.86)	0.98(0.52,1.85)	0.72(0.32,1.62)	1.30(0.61,2.81)	0.94(0.50,1.79)			
GENUS_37	Firmicutes;D_2__Bacilli;D_3__Lactobacillales;D_4__Leuconostocaceae;D_5__Weissella (Combined: 3)	0.90(0.06,13.28)	4.51(0.34,60.59)	4.04(0.47,34.58)						
GENUS_38	Firmicutes;D_2__Clostridia;D_3__Clostridiales;D_4__Family XIII;D_5__[Eubacterium] nodatum group (Combined: 12)	0.74(0.13,4.36)	2.15(0.39,11.88)	1.59(0.39,6.53)	0.74(0.14,4.08)	3.09(0.61,15.73)	2.30(0.59,8.98)			
GENUS_41	Firmicutes;D_2__Clostridia;D_3__Clostridiales;D_4__Ruminococcaceae;D_5__uncultured (Combined: 88)	3.44(1.33,8.90);p=0.01082	0.25(0.10,0.63);p=0.00318	0.87(0.41,1.85)	2.14(0.88,5.20)	0.34(0.15,0.80);p=0.01348	0.73(0.36,1.48)		↓	
GENUS_42	Firmicutes;D_2__Clostridia;D_3__Clostridiales;D_4__Family XIII;D_5__Family XIII AD3011 group (Combined: 40)	1.29(0.55,3.04)	0.73(0.32,1.65)	0.94(0.48,1.85)	1.32(0.56,3.12)	0.76(0.33,1.72)	1.00(0.51,1.97)			
GENUS_43	Firmicutes;D_2__Clostridia;D_3__Clostridiales;D_4__Lachnospiraceae;D_5__Eisenbergiella (Combined: 14)	1.86(0.62,5.61)	0.34(0.12,0.98);p=0.04575	0.63(0.27,1.51)						
GENUS_44	Firmicutes;D_2__Bacilli;D_3__Bacillales;D_4__Family XI;D_5__Gemella (Combined: 3)	1.39(0.44,4.41)	0.91(0.30,2.77)	1.27(0.52,3.14)	1.43(0.45,4.49)	0.85(0.28,2.55)	1.21(0.49,2.96)			
GENUS_45	Firmicutes;D_2__Clostridia;D_3__Clostridiales;D_4__Lachnospiraceae;D_5__Fusicatenibacter (Combined: 27)	1.55(0.81,2.99)	0.68(0.36,1.27)	1.05(0.63,1.77)	1.49(0.77,2.88)	0.68(0.36,1.28)	1.02(0.60,1.72)			
GENUS_47	Firmicutes;D_2__Clostridia;D_3__Clostridiales;D_4__Ruminococcaceae;D_5__Ruminiclostridium 9 (Combined: 29)	0.77(0.41,1.45)	1.15(0.63,2.11)	0.89(0.54,1.47)	0.82(0.44,1.53)	1.16(0.64,2.10)	0.94(0.57,1.55)			
GENUS_48	Firmicutes;D_2__Clostridia;D_3__Clostridiales;D_4__Peptococcaceae;D_5__Peptococcus (Combined: 13)	0.26(0.03,2.25)	8.33(1.05,65.96);p=0.04452	2.19(0.39,12.14)	0.51(0.06,3.98)	5.68(0.79,40.88)	2.88(0.55,14.98)			
GENUS_49	Firmicutes;D_2__Clostridia;D_3__Clostridiales;D_4__Peptostreptococcaceae;D_5__Romboutsia (Combined: 4)	0.84(0.39,1.83)	1.65(0.78,3.50)	1.39(0.75,2.58)	0.93(0.42,2.03)	1.70(0.81,3.59)	1.57(0.85,2.93)			

GENUS_50	Bacteroidetes;D_2__Bacteroidia;D_3__Bacteroidales;D_4__Marinifilaceae;D_5__Odoribacter (Combined: 16)	2.06(0.60,7.03)	0.76(0.23,2.47)	1.57(0.59,4.15)	1.93(0.56,6.67)	0.78(0.24,2.54)	1.51(0.57,4.02)			
GENUS_51	Firmicutes;D_2__Clostridia;D_3__Clostridiales;D_4__Lachnospiraceae; D_5__Roseburia (Combined: 32)	0.76(0.34,1.69)	0.76(0.35,1.64)	0.58(0.31,1.09)	0.77(0.34,1.71)	0.79(0.37,1.71)	0.61(0.32,1.15)			
GENUS_52	Firmicutes;D_2__Clostridia;D_3__Clostridiales;D_4__Ruminococcaceae; D_5__Ruminococcaceae UCG-002 (Combined: 24)	0.68(0.28,1.70)	0.72(0.30,1.72)	0.49(0.24,1.01)	0.70(0.28,1.76)	0.73(0.31,1.76)	0.52(0.25,1.07)			
GENUS_53	Firmicutes;D_2__Clostridia;D_3__Clostridiales;D_4__Ruminococcaceae; D_5__UBA1819 (Combined: 7)	2.67(1.10,6.49);p=0.03003	0.27(0.11,0.63);p=0.00246	0.71(0.35,1.44)	2.36(0.97,5.73)	0.30(0.13,0.69);p=0.00479	0.70(0.35,1.40)		↓	
GENUS_54	Firmicutes;D_2__Erysipelotrichia;D_3__Erysipelotrichales;D_4__Erysipelotrichaceae;D_5__[Clostridium] innocuum group (Combined: 5)	1.10(0.39,3.06)	1.67(0.62,4.47)	1.83(0.81,4.12)	1.12(0.40,3.15)	1.63(0.61,4.36)	1.83(0.81,4.15)			
GENUS_56	Firmicutes;D_2__Clostridia;D_3__Clostridiales;D_4__Ruminococcaceae; D_5__Ruminococcaceae UCG-013 (Combined: 53)	0.73(0.37,1.44)	1.30(0.68,2.48)	0.95(0.56,1.62)	0.73(0.37,1.45)	1.30(0.68,2.49)	0.96(0.56,1.64)			
GENUS_57	Firmicutes;D_2__Clostridia;D_3__Clostridiales;D_4__Lachnospiraceae; D_5__Hungatella (Combined: 6)	2.70(0.42,17.19)	0.09(0.02,0.54);p=0.00812	0.24(0.06,1.05)						
GENUS_58	Firmicutes;D_2__Clostridia;D_3__Clostridiales;D_4__Lachnospiraceae; D_5__Lachnospiraceae FCS020 group (Combined: 16)	1.17(0.52,2.63)	0.51(0.23,1.11)	0.59(0.31,1.13)	1.03(0.46,2.33)	0.52(0.24,1.13)	0.54(0.28,1.02)			
GENUS_59	Firmicutes;D_2__Negativicutes;D_3__Selenomonadales;D_4__Veillonellaceae;D_5__Veillonella (Combined: 18)	0.43(0.14,1.30)	1.82(0.63,5.22)	0.79(0.33,1.88)	0.82(0.27,2.44)	0.99(0.35,2.80)	0.81(0.34,1.92)			
GENUS_60	Firmicutes;D_2__Clostridia;D_3__Clostridiales;D_4__Lachnospiraceae; D_5__GCA-900066575 (Combined: 21)	1.02(0.51,2.05)	1.14(0.58,2.23)	1.16(0.66,2.01)	0.94(0.47,1.89)	1.25(0.64,2.43)	1.18(0.68,2.04)			
GENUS_61	Firmicutes;D_2__Clostridia;D_3__Clostridiales;D_4__Ruminococcaceae; D_5__Ruminiclostridium 6 (Combined: 10)	0.41(0.09,1.81)	3.91(0.94,16.27)	1.61(0.50,5.22)	0.43(0.10,1.90)	3.45(0.83,14.33)	1.47(0.45,4.81)			
GENUS_62	Firmicutes;D_2__Clostridia;D_3__Clostridiales;D_4__Lachnospiraceae; D_5__CAG-56 (Combined: 16)	1.41(0.34,5.78)	0.48(0.12,1.85)	0.67(0.22,2.06)	1.34(0.32,5.56)	0.51(0.13,1.98)	0.69(0.22,2.11)			
GENUS_63	Firmicutes;D_2__Clostridia;D_3__Clostridiales;D_4__Ruminococcaceae; D_5__Negativibacillus (Combined: 8)	1.55(0.36,6.73)	1.22(0.30,5.01)	1.89(0.59,6.06)	1.35(0.31,5.91)	1.36(0.33,5.54)	1.84(0.57,5.91)			
GENUS_65	Firmicutes;D_2__Clostridia;D_3__Clostridiales;D_4__Ruminococcaceae (Combined: 126)	0.50(0.19,1.31)	1.11(0.44,2.80)	0.55(0.26,1.19)	0.52(0.20,1.37)	1.06(0.42,2.67)	0.55(0.25,1.18)			
GENUS_66	Firmicutes;D_2__Clostridia;D_3__Clostridiales;D_4__Family XIII;D_5__Family XIII UCG-001 (Combined: 9)	0.64(0.26,1.57)	1.16(0.49,2.73)	0.75(0.37,1.52)	0.67(0.28,1.59)	1.28(0.57,2.91)	0.86(0.44,1.71)			
GENUS_67	Firmicutes;D_2__Clostridia;D_3__Clostridiales;D_4__Lachnospiraceae (Combined: 215)	0.65(0.35,1.21)	0.69(0.38,1.26)	0.45(0.27,0.73);p=0.00147	0.65(0.35,1.20)	0.74(0.41,1.32)	0.48(0.30,0.78);p=0.00298			↓
GENUS_68	Proteobacteria;D_2__Deltaproteobacteria;D_3__Desulfovibrionales;D_4__Desulfovibrionaceae;D_5__Bilophila (Combined: 20)	1.32(0.42,4.15)	0.94(0.31,2.83)	1.24(0.50,3.08)	1.28(0.40,4.07)	0.98(0.33,2.95)	1.26(0.50,3.14)			
GENUS_70	Actinobacteria;D_2__Actinobacteria;D_3__Actinomycetales;D_4__Actinomycetaceae;D_5__Actinomyces (Combined: 17)	1.85(0.91,3.75)	0.77(0.39,1.52)	1.42(0.82,2.47)	2.06(1.02,4.19);p=0.04506	0.71(0.36,1.39)	1.46(0.84,2.53)	↑		
GENUS_71	Firmicutes;D_2__Clostridia;D_3__Clostridiales;D_4__Family XIII;D_5__[Eubacterium] brachy group (Combined: 10)	1.21(0.38,3.81)	1.45(0.48,4.40)	1.76(0.71,4.36)	1.23(0.39,3.88)	1.44(0.48,4.33)	1.77(0.71,4.39)			

GENUS_72	Firmicutes;D_2__Clostridia;D_3__Clostridiales;D_4__Lachnospiraceae;D_5__Lachnoclostridium (Combined: 69)	0.87(0.46,1.66)	1.06(0.57,1.97)	0.92(0.55,1.54)	0.87(0.47,1.62)	1.18(0.66,2.13)	1.03(0.63,1.68)			
GENUS_73	Firmicutes;D_2__Clostridia;D_3__Clostridiales;D_4__Lachnospiraceae;D_5__[Eubacterium] eligens group (Combined: 28)	1.80(0.61,5.31)	0.60(0.21,1.70)	1.08(0.46,2.55)	1.75(0.59,5.19)	0.55(0.20,1.55)	0.96(0.41,2.28)			
GENUS_74	Firmicutes;D_2__Bacilli;D_3__Lactobacillales;D_4__Carnobacteriaceae;D_5__Granulicatella (Combined: 3)	1.63(0.57,4.70)	1.14(0.41,3.18)	1.87(0.81,4.28)	1.94(0.67,5.60)	0.94(0.34,2.60)	1.83(0.80,4.16)			
GENUS_75	Firmicutes;D_2__Clostridia;D_3__Clostridiales;D_4__Ruminococcaceae;D_5__Ruminococcaceae UCG-010 (Combined: 65)	1.45(0.33,6.46)	0.32(0.08,1.33)	0.46(0.14,1.50)						
GENUS_76	Firmicutes;D_2__Bacilli;D_3__Lactobacillales;D_4__Streptococcaceae;D_5__Lactococcus (Combined: 9)	1.18(0.45,3.13)	0.76(0.30,1.95)	0.91(0.42,1.95)	1.41(0.54,3.65)	0.69(0.28,1.70)	0.97(0.46,2.05)			
GENUS_78	Firmicutes;D_2__Clostridia;D_3__Clostridiales;D_4__Lachnospiraceae;D_5__Lachnospiraceae NK4A136 group (Combined: 44)	1.56(0.75,3.25)	0.51(0.25,1.02)	0.79(0.44,1.41)	1.63(0.78,3.41)	0.50(0.25,1.01)	0.81(0.45,1.46)			
GENUS_79	Firmicutes;D_2__Erysipelotrichia;D_3__Erysipelotrichales;D_4__Erysipelotrichaceae;D_5__Erysipelotrichaceae UCG-003 (Combined: 11)	0.99(0.39,2.54)	0.74(0.30,1.83)	0.74(0.35,1.55)	0.98(0.38,2.53)	0.75(0.30,1.84)	0.73(0.35,1.55)			
GENUS_82	Firmicutes;D_2__Clostridia;D_3__Clostridiales;D_4__Defluviitaleaceae;D_5__Defluviitaleaceae UCG-011 (Combined: 12)				2.25(0.64,7.90)	0.63(0.19,2.10)	1.43(0.54,3.82)			
GENUS_83	Firmicutes;D_2__Clostridia;D_3__Clostridiales;D_4__Clostridiales vadinBB60 group;D_5__uncultured Thermoanaerobacterales bacterium (Combined: 2)	0.72(0.04,11.79)	6.32(0.43,93.75)	4.54(0.49,42.25)						
GENUS_84	Firmicutes;D_2__Erysipelotrichia;D_3__Erysipelotrichales;D_4__Erysipelotrichaceae;D_5__Holdemania (Combined: 12)	0.59(0.30,1.18)	1.36(0.70,2.62)	0.80(0.47,1.39)	0.60(0.30,1.20)	1.38(0.71,2.65)	0.83(0.48,1.43)			
GENUS_85	Firmicutes;D_2__Clostridia;D_3__Clostridiales;D_4__Lachnospiraceae;D_5__Sellimonas (Combined: 13)	1.70(0.36,8.14)	1.40(0.31,6.31)	2.39(0.69,8.25)	1.53(0.32,7.35)	1.23(0.27,5.50)	1.87(0.54,6.50)			
GENUS_86	Firmicutes;D_2__Clostridia;D_3__Clostridiales;D_4__Christensenellaceae;D_5__uncultured (Combined: 32)	1.56(0.56,4.33)	0.38(0.14,1.02)	0.60(0.27,1.33)	1.48(0.57,3.85)	0.53(0.21,1.32)	0.79(0.37,1.67)			
GENUS_88	Firmicutes;D_2__Clostridia;D_3__Clostridiales;D_4__Ruminococcaceae;D_5__DTU089 (Combined: 4)	1.60(0.63,4.07)	0.69(0.28,1.69)	1.11(0.53,2.31)	1.58(0.63,4.01)	0.71(0.29,1.72)	1.13(0.54,2.33)			
GENUS_89	Actinobacteria;D_2__Coriobacteriia;D_3__Coriobacteriales;D_4__Coriobacteriales Incertae Sedis;D_5__uncultured (Combined: 11)	3.29(0.77,14.05)	0.27(0.07,1.07)	0.88(0.28,2.74)	2.27(0.54,9.48)	0.42(0.11,1.64)	0.95(0.31,2.93)			
GENUS_90	Firmicutes;D_2__Clostridia;D_3__Clostridiales;D_4__Ruminococcaceae;D_5__Anaerotruncus (Combined: 8)	2.35(0.74,7.50)	0.28(0.09,0.86);p=0.0267	0.67(0.28,1.63)	3.11(0.98,9.88)	0.28(0.09,0.86);p=0.02538	0.88(0.37,2.11)		↓	
GENUS_91	Actinobacteria;D_2__Coriobacteriia;D_3__Coriobacteriales;D_4__Eggerthellaceae (Combined: 8)	1.51(0.39,5.86)	0.26(0.07,0.94);p=0.03997	0.39(0.14,1.09)	1.74(0.47,6.50)	0.33(0.09,1.15)	0.57(0.21,1.54)			
GENUS_92	Firmicutes;D_2__Clostridia;D_3__Clostridiales;D_4__Lachnospiraceae;D_5__[Eubacterium] ventriosum group (Combined: 30)	0.42(0.19,0.94);p=0.03418	1.55(0.71,3.37)	0.65(0.34,1.23)	0.38(0.17,0.86);p=0.01993	1.45(0.67,3.12)	0.56(0.29,1.05)	↓		
GENUS_93	Proteobacteria;D_2__Gammaproteobacteria;D_3__Pseudomonadales;D_4__Pseudomonadaceae;D_5__Pseudomonas (Combined: 9)	1.68(0.15,19.36)	0.09(0.01,0.90);p=0.04084	0.15(0.02,0.99);p=0.04844	1.09(0.10,12.44)	0.15(0.01,1.48)	0.16(0.02,1.07)			
GENUS_94	Firmicutes;D_2__Clostridia;D_3__Clostridiales;D_4__Ruminococcaceae;D_5__Fournierella (Combined: 13)	2.83(0.65,12.37)	0.59(0.14,2.43)	1.66(0.52,5.33)	2.69(0.61,11.92)	0.56(0.13,2.31)	1.50(0.47,4.84)			

GENUS_95	Firmicutes;D_2__Clostridia;D_3__Clostridiales;D_4__Ruminococcaceae;D_5__Angelakisella (Combined: 6)	1.15(0.36,3.66)	0.51(0.17,1.54)	0.59(0.24,1.46)	1.04(0.33,3.28)	0.64(0.22,1.92)	0.67(0.27,1.65)			
GENUS_96	Firmicutes;D_2__Erysipelotrichia;D_3__Erysipelotrichales;D_4__Erysipelotrichaceae;D_5__Erysipelatoclostridium (Combined: 9)	0.62(0.21,1.84)	1.32(0.47,3.71)	0.82(0.35,1.93)	0.64(0.22,1.90)	1.28(0.46,3.61)	0.82(0.35,1.94)			
GENUS_97	Actinobacteria;D_2__Coriobacteriia;D_3__Coriobacteriales;D_4__Eggerthellaceae;D_5__Gordonibacter (Combined: 10)	1.20(0.48,2.96)	0.56(0.24,1.34)	0.67(0.33,1.37)	1.18(0.48,2.94)	0.59(0.25,1.39)	0.70(0.34,1.42)			
GENUS_98	Firmicutes;D_2__Clostridia;D_3__Clostridiales;D_4__Lachnospiraceae;D_5__Lachnospiraceae UCG-010 (Combined: 18)	0.40(0.13,1.22)	1.87(0.64,5.44)	0.75(0.31,1.81)	0.40(0.13,1.21)	2.05(0.71,5.92)	0.82(0.34,1.98)			
GENUS_99	Firmicutes;D_2__Clostridia;D_3__Clostridiales;D_4__Lachnospiraceae;D_5__[Eubacterium] xylanophilum group (Combined: 20)	0.42(0.10,1.71)	1.64(0.42,6.36)	0.68(0.22,2.09)	0.41(0.10,1.71)	2.05(0.53,7.94)	0.85(0.27,2.61)			
GENUS_100	Firmicutes;D_2__Clostridia;D_3__Clostridiales;D_4__Lachnospiraceae;D_5__GCA-900066755 (Combined: 2)	3.67(0.74,18.27)	0.37(0.08,1.75)	1.36(0.42,4.39)	2.38(0.50,11.34)	0.44(0.10,1.99)	1.05(0.33,3.29)			
GENUS_101	Actinobacteria;D_2__Coriobacteriia;D_3__Coriobacteriales;D_4__Eggerthellaceae;D_5__Adlercreutzia (Combined: 2)	1.82(0.63,5.27)	0.55(0.20,1.53)	1.01(0.43,2.33)	1.57(0.54,4.58)	0.54(0.20,1.50)	0.85(0.37,1.99)			
GENUS_102	Firmicutes;D_2__Clostridia;D_3__DTU014;D_4__uncultured bacterium;D_5__ (Combined: 2)				2.09(0.26,17.07)	0.68(0.09,5.07)	1.42(0.27,7.34)			
GENUS_103	Firmicutes;D_2__Clostridia;D_3__Clostridiales;D_4__Ruminococcaceae;D_5__Ruminococcaceae UCG-003 (Combined: 12)	1.17(0.42,3.23)	0.48(0.18,1.27)	0.56(0.25,1.25)	1.18(0.42,3.30)	0.48(0.18,1.27)	0.56(0.25,1.27)			
GENUS_104	Firmicutes;D_2__Clostridia;D_3__Clostridiales;D_4__Ruminococcaceae;D_5__Hydrogenoanaerobacterium (Combined: 5)	0.44(0.06,3.09)	1.82(0.28,11.78)	0.80(0.17,3.81)	0.40(0.06,2.81)	1.70(0.27,10.75)	0.68(0.14,3.24)			
GENUS_105	Firmicutes;D_2__Clostridia;D_3__Clostridiales;D_4__Christensenellaceae;D_5__Catabacter (Combined: 6)	0.94(0.13,6.74)	0.39(0.06,2.56)	0.37(0.08,1.71)	0.54(0.09,3.31)	2.17(0.39,12.19)	1.18(0.28,4.99)			
GENUS_106	Firmicutes;D_2__Clostridia;D_3__Clostridiales;D_4__Ruminococcaceae;D_5__Ruminococcaceae UCG-007 (Combined: 2)	2.87(0.22,36.61)	0.78(0.06,9.36)	2.23(0.32,15.38)	3.80(0.27,53.12)	0.66(0.05,8.85)	2.51(0.36,17.32)			
GENUS_108	Proteobacteria;D_2__Deltaproteobacteria;D_3__Desulfovibrionales;D_4__Desulfovibrionaceae;D_5__Desulfovibrio (Combined: 11)	0.45(0.03,6.62)	4.14(0.31,54.60)	1.87(0.22,15.76)	0.41(0.03,6.12)	9.43(0.72,122.93)	3.90(0.46,32.97)			
GENUS_109	Firmicutes;D_2__Erysipelotrichia;D_3__Erysipelotrichales;D_4__Erysipelotrichaceae;D_5__Dielma (Combined: 5)	1.02(0.26,4.01)	2.17(0.58,8.20)	2.21(0.74,6.60)	1.01(0.26,3.93)	2.12(0.57,7.85)	2.14(0.72,6.33)			
GENUS_110	Firmicutes;D_2__Clostridia;D_3__Clostridiales;D_4__Lachnospiraceae;D_5__Lachnospira (Combined: 35)	0.79(0.34,1.84)	0.88(0.39,1.96)	0.69(0.36,1.35)	0.74(0.32,1.72)	0.87(0.39,1.94)	0.64(0.33,1.25)			
GENUS_111	Firmicutes;D_2__Bacilli;D_3__Lactobacillales;D_4__Leuconostocaceae;D_5__Leuconostoc (Combined: 7)	0.51(0.08,3.42)	1.28(0.21,7.89)	0.66(0.15,2.97)	0.35(0.05,2.34)	1.67(0.27,10.22)	0.58(0.13,2.64)			
GENUS_112	Firmicutes;D_2__Bacilli;D_3__Bacillales;D_4__Staphylococcaceae;D_5__Staphylococcus (Combined: 4)	28.68(2.47,332.59);p=0.00728	0.20(0.02,2.16)	5.64(1.00,31.70);p=0.04938	53.19(4.12,687.09);p=0.00233	0.17(0.01,2.05)	9.04(1.74,47.11);p=0.00893	↑		↑
GENUS_113	Firmicutes;D_2__Clostridia;D_3__Clostridiales;D_4__Ruminococcaceae;D_5__Candidatus Soleaferrea (Combined: 20)	1.09(0.52,2.28)	0.77(0.38,1.57)	0.84(0.47,1.49)	1.04(0.49,2.18)	0.77(0.38,1.55)	0.79(0.45,1.42)			
GENUS_114	Firmicutes;D_2__Clostridia;D_3__Clostridiales;D_4__Family XIII;D_5__Mogibacterium (Combined: 5)	1.78(0.39,8.13)	0.48(0.11,2.09)	0.86(0.29,2.56)	2.05(0.43,9.77)	0.41(0.09,1.83)	0.83(0.28,2.47)			

GENUS_115	Firmicutes;D_2__Clostridia;D_3__Clostridiales;D_4__Clostridiales vadinBB60 group;D_5__uncultured bacterium (Combined: 38)	1.19(0.24,5.93)	0.73(0.16,3.45)	0.87(0.24,3.12)	1.94(0.40,9.37)	0.54(0.12,2.41)	1.04(0.30,3.59)			
GENUS_116	Patescibacteria;D_2__Saccharimonadia;D_3__Saccharimonadales;D_4__Saccharimonadaceae (Combined: 1)	2.47(0.64,9.49)	1.51(0.40,5.67)	3.72(1.29,10.71);p=0.01504	2.29(0.60,8.77)	1.58(0.42,5.91)	3.63(1.26,10.40);p=0.01659			↑
GENUS_117	Actinobacteria;D_2__Coriobacteriia;D_3__Coriobacteriales;D_4__Eggerthellaceae;D_5__Enterorhabdus (Combined: 9)	0.75(0.09,5.94)	3.25(0.44,23.91)	2.43(0.47,12.68)	0.78(0.10,6.23)	3.24(0.44,23.65)	2.52(0.48,13.20)			
GENUS_119	Proteobacteria;D_2__Gammaproteobacteria;D_3__Betaproteobacteriales;D_4__Burkholderiaceae;D_5__Parasutterella (Combined: 15)	0.85(0.22,3.33)	0.56(0.15,2.08)	0.48(0.16,1.41)	0.87(0.22,3.45)	0.57(0.15,2.12)	0.50(0.17,1.48)			
GENUS_120	Firmicutes;D_2__Clostridia;D_3__Clostridiales;D_4__Lachnospiraceae;D_5__Lachnospiraceae ND3007 group (Combined: 16)	0.59(0.21,1.65)	0.72(0.27,1.93)	0.43(0.19,0.97);p=0.04108						
GENUS_121	Bacteroidetes;D_2__Bacteroidia;D_3__Bacteroidales;D_4__Barnesiellaceae;D_5__Coprobacter (Combined: 7)	1.66(0.07,41.61)	1.24(0.06,27.54)	2.06(0.16,26.55)	4.34(0.17,109.17)	0.54(0.02,11.63)	2.34(0.18,29.91)			
GENUS_122	Firmicutes;D_2__Clostridia;D_3__Clostridiales;D_4__Ruminococcaceae;D_5__Butyricicoccus (Combined: 41)	0.64(0.35,1.17)	1.32(0.74,2.34)	0.85(0.53,1.36)	0.63(0.35,1.15)	1.40(0.79,2.46)	0.88(0.55,1.41)			
GENUS_124	Firmicutes;D_2__Clostridia;D_3__Clostridiales;D_4__Ruminococcaceae;D_5__Oscillibacter (Combined: 28)	0.93(0.44,1.94)	1.05(0.51,2.13)	0.97(0.54,1.74)	0.94(0.45,1.98)	1.02(0.50,2.07)	0.96(0.53,1.73)			
GENUS_126	Firmicutes;D_2__Clostridia;D_3__Clostridiales;D_4__Lachnospiraceae;D_5__Lachnospiraceae UCG-008 (Combined: 6)	0.59(0.22,1.57)	1.50(0.58,3.87)	0.88(0.40,1.93)	0.61(0.22,1.64)	1.47(0.57,3.78)	0.89(0.40,1.95)			
GENUS_127	Firmicutes;D_2__Clostridia;D_3__Clostridiales;D_4__Lachnospiraceae;D_5__Lachnospiraceae UCG-001 (Combined: 23)	9.72(2.00,47.23);p=0.00479	0.07(0.02,0.34);p=0.00083	0.73(0.21,2.50)	9.47(1.94,46.30);p=0.00551	0.08(0.02,0.35);p=0.00087	0.72(0.21,2.49)	↑	↓	
GENUS_128	Firmicutes;D_2__Clostridia;D_3__Clostridiales;D_4__Lachnospiraceae;D_5__Lachnospiraceae UCG-004 (Combined: 29)	0.94(0.37,2.40)	1.54(0.63,3.76)	1.45(0.69,3.04)	1.01(0.40,2.58)	1.29(0.53,3.16)	1.31(0.62,2.75)			
GENUS_129	Firmicutes;D_2__Clostridia;D_3__Clostridiales;D_4__Clostridiales vadinBB60 group;D_5__gut metagenome (Combined: 14)	1.42(0.34,5.96)	0.70(0.18,2.78)	1.00(0.32,3.10)	1.49(0.38,5.81)	0.72(0.20,2.62)	1.07(0.37,3.13)			
GENUS_130	Lentisphaerae;D_2__Lentisphaeria;D_3__Victivallales;D_4__Victivallaceae;D_5__Victivallis (Combined: 6)	0.37(0.03,4.50)	8.31(0.75,91.60)	3.11(0.42,22.82)						
GENUS_131	Firmicutes;D_2__Clostridia;D_3__Clostridiales;D_4__Ruminococcaceae;D_5__Flavonifractor (Combined: 17)	1.26(0.60,2.64)	0.84(0.41,1.72)	1.06(0.59,1.90)	1.30(0.62,2.74)	0.83(0.41,1.70)	1.08(0.60,1.95)			
GENUS_134	Firmicutes;D_2__Clostridia;D_3__Clostridiales;D_4__Lachnospiraceae;D_5__Moryella (Combined: 3)	0.38(0.12,1.18)	1.64(0.55,4.89)	0.62(0.25,1.54)	0.37(0.12,1.14)	1.40(0.48,4.10)	0.51(0.21,1.26)			
GENUS_135	Firmicutes;D_2__Clostridia;D_3__Clostridiales;D_4__Ruminococcaceae;D_5__Ruminococcaceae UCG-009 (Combined: 13)	0.15(0.03,0.77);p=0.02305	3.71(0.79,17.31)	0.57(0.16,2.08)	0.10(0.02,0.48);p=0.00431	4.73(1.03,21.69);p=0.04546	0.45(0.13,1.64)	↓	↑	
GENUS_137	Proteobacteria;D_2__Gammaproteobacteria;D_3__Pasteurellales;D_4__Pasteurellaceae;D_5__Haemophilus (Combined: 3)	1.10(0.30,4.06)	0.75(0.21,2.62)	0.82(0.29,2.31)	0.97(0.26,3.62)	0.82(0.23,2.88)	0.79(0.28,2.25)			
GENUS_139	Firmicutes;D_2__Clostridia;D_3__Clostridiales;D_4__Ruminococcaceae;D_5__Oscillospira (Combined: 12)	1.36(0.39,4.70)	0.60(0.18,1.98)	0.82(0.31,2.18)	1.84(0.53,6.40)	0.41(0.12,1.33)	0.75(0.28,1.98)			
GENUS_140	Firmicutes;D_2__Clostridia;D_3__Clostridiales;D_4__Clostridiaceae 1;D_5__Clostridium sensu stricto 1 (Combined: 22)	2.76(1.08,7.00);p=0.03315	0.76(0.31,1.86)	2.09(1.00,4.37)	2.64(1.03,6.75);p=0.04282	0.79(0.32,1.93)	2.08(0.99,4.37)	↑		

GENUS_141	Firmicutes;D_2__Clostridia;D_3__Clostridiales;D_4__Ruminococcaceae;D_5__Phocea (Combined: 3)	1.19(0.32,4.43)	1.28(0.36,4.56)	1.52(0.54,4.31)	1.06(0.28,3.92)	1.19(0.34,4.18)	1.26(0.45,3.53)			
GENUS_142	Proteobacteria;D_2__Gammaproteobacteria;D_3__Enterobacteriales;D_4__Enterobacteriaceae;D_5__Escherichia-Shigella (Combined: 10)	1.96(0.46,8.37)	0.67(0.17,2.69)	1.31(0.42,4.15)						
GENUS_143	Firmicutes;D_2__Clostridia;D_3__Clostridiales (Combined: 29)	0.06(0.01,0.50);p=0.00965	2.26(0.30,17.25)	0.13(0.02,0.74);p=0.02183	0.01(0.00,0.09);p=0.00003;q=0.01554	10.93(1.59,74.89);p=0.01491	0.11(0.02,0.67);p=0.01626	↓*	↑	↓
GENUS_144	Actinobacteria;D_2__Coriobacteriia;D_3__Coriobacteriales;D_4__Eggerthellaceae;D_5__Eggerthella (Combined: 3)	2.01(0.71,5.73)	0.72(0.26,1.96)	1.44(0.63,3.30)						
GENUS_145	Firmicutes;D_2__Clostridia;D_3__Clostridiales;D_4__Ruminococcaceae;D_5__Ruminiclostridium (Combined: 7)	7.42(1.15,47.77);p=0.03481	0.18(0.03,1.10)	1.31(0.45,3.83)	6.76(1.05,43.53);p=0.04441	0.19(0.03,1.20)	1.30(0.46,3.69)	↑		
GENUS_146	Firmicutes;D_2__Clostridia;D_3__Clostridiales;D_4__Lachnospiraceae;D_5__Anaerosporobacter (Combined: 3)	15.57(1.79,135.04);p=0.01275	0.16(0.02,1.27)	2.47(0.46,13.15)	17.23(1.93,153.84);p=0.01083	0.11(0.01,0.87);p=0.03622	1.83(0.34,9.75)	↑	↓	
GENUS_147	Firmicutes;D_2__Clostridia;D_3__Clostridiales;D_4__Ruminococcaceae;D_5__GCA-900066225 (Combined: 20)	2.29(0.54,9.67)	0.54(0.13,2.16)	1.23(0.40,3.82)	1.46(0.36,5.92)	0.75(0.20,2.87)	1.10(0.36,3.30)			
GENUS_148	Firmicutes;D_2__Clostridia;D_3__Clostridiales;D_4__Ruminococcaceae;D_5__Pseudoflavonifractor (Combined: 3)	0.34(0.03,3.44)	1.04(0.12,9.21)	0.36(0.06,2.24)	0.91(0.09,8.86)	0.37(0.04,3.07)	0.34(0.06,1.87)			
GENUS_149	Firmicutes;D_2__Clostridia;D_3__Clostridiales;D_4__Lachnospiraceae;D_5__Tyzzerella (Combined: 7)	0.33(0.04,2.54)	13.70(1.92,97.99);p=0.00911	4.52(0.89,23.03)	0.28(0.04,2.20)	16.36(2.31,116.08);p=0.00518	4.64(0.91,23.71)		↑	
GENUS_152	Proteobacteria;D_2__Gammaproteobacteria;D_3__Betaproteobacteriales;D_4__Burkholderiaceae;D_5__Noviherbaspirillum (Combined: 4)	2.19(0.29,16.69)	0.33(0.05,2.34)	0.73(0.17,3.15)	1.92(0.25,14.96)	0.30(0.04,2.13)	0.58(0.14,2.47)			
GENUS_153	Proteobacteria;D_2__Gammaproteobacteria;D_3__Betaproteobacteriales;D_4__Burkholderiaceae;D_5__Sutterella (Combined: 37)	0.37(0.09,1.59)	3.17(0.79,12.83)	1.18(0.37,3.75)	0.43(0.10,1.85)	2.47(0.62,9.89)	1.06(0.34,3.37)			
GENUS_154	Firmicutes;D_2__Clostridia;D_3__Clostridiales;D_4__Peptostreptococcaceae;D_5__Intestinibacter (Combined: 5)	1.12(0.50,2.53)	0.57(0.26,1.25)	0.64(0.34,1.22)	1.37(0.64,2.95)	0.62(0.30,1.29)	0.85(0.46,1.56)			
GENUS_155	Cyanobacteria;D_2__Oxyphotobacteria;D_3__Chloroplast (Combined: 12)	7.47(0.72,77.51)	0.47(0.05,4.47)	3.51(0.56,22.07)	5.57(0.60,51.96)	0.78(0.09,6.65)	4.34(0.76,24.91)			
GENUS_156	Firmicutes;D_2__Clostridia;D_3__Clostridiales;D_4__Peptostreptococcaceae;D_5__Terrisporobacter (Combined: 2)	3.61(1.00,12.99);p=0.04924	0.25(0.07,0.86);p=0.0282	0.91(0.33,2.48)	3.45(0.98,12.07)	0.36(0.11,1.19)	1.24(0.46,3.31)			
GENUS_157	Proteobacteria;D_2__Alphaproteobacteria;D_3__Caulobacterales;D_4__Caulobacteraceae;D_5__Brevundimonas (Combined: 1)	2.01(0.12,32.99)	0.29(0.02,4.19)	0.58(0.08,4.03)	1.95(0.12,31.50)	0.32(0.02,4.50)	0.62(0.09,4.32)			
GENUS_160	Actinobacteria;D_2__Actinobacteria;D_3__Micrococcales;D_4__Micrococcaceae;D_5__Rothia (Combined: 9)	0.74(0.18,3.08)	2.71(0.67,10.92)	2.00(0.63,6.36)	0.96(0.24,3.81)	2.81(0.72,11.00)	2.70(0.89,8.17)			
GENUS_162	Proteobacteria;D_2__Gammaproteobacteria;D_3__Pasteurellales;D_4__Pasteurellaceae (Combined: 1)	0.50(0.04,5.97)	0.57(0.05,6.07)	0.28(0.04,2.03)	1.29(0.12,14.31)	0.49(0.05,4.81)	0.63(0.10,4.13)			
GENUS_163	Firmicutes;D_2__Clostridia;D_3__Clostridiales;D_4__Lachnospiraceae;D_5__[Ruminococcus] gnavus group (Combined: 9)	1.20(0.31,4.73)	0.94(0.25,3.51)	1.13(0.38,3.36)	0.85(0.22,3.32)	1.33(0.36,4.89)	1.12(0.38,3.32)			
GENUS_165	Proteobacteria;D_2__Gammaproteobacteria;D_3__Betaproteobacteriales;D_4__Neisseriaceae;D_5__Neisseria (Combined: 5)	1.84(0.05,70.69)	0.19(0.01,6.06)	0.34(0.02,5.18)						

GENUS_166	Tenericutes;D_2__Mollicutes;D_3__Mollicutes RF39 (Combined: 22)	0.33(0.00,25.32)	0.11(0.00,7.45)	0.04(0.00,1.18)	0.24(0.00,19.22)	0.18(0.00,11.44)	0.04(0.00,1.36)			
GENUS_167	Proteobacteria;D_2__Gammaproteobacteria;D_3__Enterobacteriales;D_4__Enterobacteriaceae (Combined: 15)	0.36(0.04,3.02)	4.92(0.64,37.59)	1.79(0.33,9.61)	0.15(0.02,1.20)	7.76(1.05,57.09);p=0.04423	1.14(0.22,6.00)		↑	
GENUS_168	Firmicutes;D_2__Erysipelotrichia;D_3__Erysipelotrichales;D_4__Erysipelotrichaceae;D_5__Turicibacter (Combined: 20)	2.59(1.04,6.46);p=0.04151	0.58(0.24,1.40)	1.51(0.73,3.11)	2.89(1.15,7.22);p=0.02337	0.57(0.24,1.36)	1.64(0.80,3.39)	↑		
GENUS_174	Firmicutes;D_2__Erysipelotrichia;D_3__Erysipelotrichales;D_4__Erysipelotrichaceae;D_5__Merdibacter (Combined: 3)	2.44(0.32,18.38)	0.45(0.06,3.16)	1.10(0.25,4.86)	1.28(0.18,9.11)	0.57(0.09,3.76)	0.72(0.17,3.03)			
GENUS_175	Firmicutes;D_2__Clostridia;D_3__Clostridiales;D_4__Ruminococcaceae;D_5__Anaerofilum (Combined: 8)	1.56(0.25,9.52)	0.76(0.13,4.34)	1.18(0.29,4.87)	0.78(0.14,4.54)	0.94(0.18,5.01)	0.74(0.19,2.90)			
GENUS_176	Actinobacteria;D_2__Coriobacteriia;D_3__Coriobacteriales;D_4__Coriobacteriales Incertae Sedis;D_5__Raoultibacter (Combined: 2)	0.14(0.01,2.61)	2.08(0.15,28.14)	0.29(0.03,3.38)	0.04(0.00,0.93);p=0.04498	3.50(0.25,49.63)	0.13(0.01,1.96)	↓		
GENUS_177	Firmicutes;D_2__Clostridia;D_3__Clostridiales;D_4__Eubacteriaceae;D_5__Eubacterium (Combined: 2)	1.17(0.12,11.08)	0.95(0.11,8.19)	1.11(0.19,6.54)	1.08(0.12,9.50)	1.25(0.16,9.94)	1.34(0.24,7.57)			
GENUS_178	Firmicutes;D_2__Erysipelotrichia;D_3__Erysipelotrichales;D_4__Erysipelotrichaceae;D_5__Solobacterium (Combined: 2)	0.07(0.01,0.94);p=0.04459	1.34(0.12,15.39)	0.10(0.01,0.75);p=0.02545						
GENUS_179	Firmicutes;D_2__Erysipelotrichia;D_3__Erysipelotrichales;D_4__Erysipelotrichaceae;D_5__uncultured (Combined: 2)	1.81(0.12,26.20)	1.97(0.15,25.89)	3.55(0.42,29.72)	1.48(0.11,20.69)	2.76(0.22,34.21)	4.09(0.50,33.34)			
GENUS_180	Proteobacteria;D_2__Gammaproteobacteria;D_3__Betaproteobacteriales;D_4__Burkholderiaceae;D_5__Variovorax (Combined: 1)				5.52(0.96,31.64)	0.24(0.04,1.30)	1.32(0.41,4.23)			
GENUS_182	Firmicutes;D_2__Clostridia;D_3__Clostridiales;D_4__Lachnospiraceae;D_5__[Eubacterium] fissicatena group (Combined: 8)	1.14(0.38,3.42)	0.58(0.20,1.67)	0.66(0.28,1.56)	1.05(0.35,3.17)	0.61(0.21,1.76)	0.64(0.27,1.52)			
GENUS_183	Firmicutes;D_2__Clostridia;D_3__Clostridiales;D_4__Lachnospiraceae;D_5__UC5-1-2E3 (Combined: 1)				2.17(0.14,33.13)	0.68(0.05,9.42)	1.48(0.19,11.58)			
GENUS_187	Firmicutes;D_2__Clostridia;D_3__Clostridiales;D_4__Ruminococcaceae;D_5__Acetanaerobacterium (Combined: 2)	1.11(0.30,4.07)	0.75(0.22,2.62)	0.83(0.30,2.27)	1.03(0.28,3.81)	0.64(0.19,2.19)	0.66(0.24,1.80)			
GENUS_188	Proteobacteria;D_2__Gammaproteobacteria;D_3__Betaproteobacteriales;D_4__Burkholderiaceae;D_5__Acidovorax (Combined: 1)	10294794638.33(0.00,Inf)	0.00(0.00,Inf)	3.46(0.51,23.64)	3690719567158151.00(0.00,Inf)	0.00(0.00,Inf)	3.68(0.53,25.66)			
GENUS_189	Firmicutes;D_2__Clostridia;D_3__Clostridiales;D_4__Peptostreptococcaceae;D_5__Paeniclostridium (Combined: 2)	7.65(0.26,222.61)	1.46(0.05,40.30)	11.20(0.80,157.45)						
GENUS_190	Firmicutes;D_2__Bacilli;D_3__Lactobacillales;D_4__Enterococcaceae;D_5__Enterococcus (Combined: 4)	0.37(0.03,5.09)	8.73(0.71,107.59)	3.27(0.41,26.14)	0.16(0.02,1.73)	7.22(0.74,70.72)	1.17(0.18,7.83)			
GENUS_192	Actinobacteria;D_2__Actinobacteria;D_3__Bifidobacteriales;D_4__Bifidobacteriaceae;D_5__Alloscardovia (Combined: 1)	39831942056.70(0.00,Inf)	0.00(0.00,Inf)	5.70(0.41,79.36)						
GENUS_193	Firmicutes;D_2__Erysipelotrichia;D_3__Erysipelotrichales;D_4__Erysipelotrichaceae;D_5__Faecalitalea (Combined: 5)	0.35(0.06,2.13)	0.90(0.16,5.03)	0.32(0.08,1.33)	0.27(0.04,1.60)	1.18(0.22,6.42)	0.31(0.08,1.31)			
GENUS_194	Bacteroidetes;D_2__Bacteroidia;D_3__Bacteroidales;D_4__Prevotellaceae;D_5__Prevotella 6 (Combined: 9)	48.00(2.89,797.63);p=0.00694	0.53(0.03,8.06)	25.40(2.88,223.81);p=0.00357						

GENUS_195	Bacteroidetes;D_2__Bacteroidia;D_3__Bacteroidales;D_4__Porphyromonadaceae;D_5__Porphyromonas (Combined: 17)	10.43(1.51,72.10);p=0.0175	0.21(0.03,1.38)	2.22(0.51,9.69)						
GENUS_196	Actinobacteria;D_2__Actinobacteria;D_3__Corynebacteriales;D_4__Corynebacteriaceae;D_5__Corynebacterium 1 (Combined: 8)	2.06(0.08,53.98)	0.79(0.03,19.29)	1.63(0.14,18.87)	3.08(0.11,88.17)	0.75(0.03,20.27)	2.31(0.20,26.40)			
GENUS_197	Firmicutes;D_2__Clostridia;D_3__Clostridiales;D_4__Family XI;D_5__Ezakiella (Combined: 4)	18.41(1.05,322.82);p=0.04626	0.04(0.00,0.60);p=0.02009	0.69(0.09,5.40)	17.08(0.97,301.43)	0.04(0.00,0.71);p=0.02738	0.75(0.11,5.04)		↓	
GENUS_199	Firmicutes;D_2__Clostridia;D_3__Clostridiales;D_4__Lachnospiraceae;D_5__uncultured organism (Combined: 1)	0.33(0.02,6.90)	0.85(0.05,15.19)	0.28(0.03,3.14)	0.44(0.02,9.37)	0.57(0.03,10.14)	0.25(0.02,2.77)			
GENUS_200	Bacteroidetes;D_2__Bacteroidia;D_3__Bacteroidales;D_4__Prevotellaceae;D_5__Prevotella (Combined: 11)	2.86(0.39,20.95)	0.52(0.08,3.54)	1.49(0.31,7.14)	2.83(0.39,20.27)	0.50(0.08,3.30)	1.42(0.30,6.65)			
GENUS_202	Proteobacteria;D_2__Gammaproteobacteria;D_3__Betaproteobacteriales;D_4__Burkholderiaceae (Combined: 7)	3.05(0.37,24.82)	0.54(0.07,4.17)	1.65(0.35,7.78)	1.09(0.16,7.41)	1.72(0.25,11.77)	1.89(0.43,8.26)			
GENUS_203	Firmicutes;D_2__Clostridia;D_3__Clostridiales;D_4__Family XI;D_5__Peptoniphilus (Combined: 8)	2.65(0.08,93.79)	0.18(0.01,5.47)	0.47(0.03,7.65)						
GENUS_204	Firmicutes;D_2__Erysipelotrichia;D_3__Erysipelotrichales;D_4__Erysipelotrichaceae;D_5__Candidatus Stoquefichus (Combined: 3)	0.08(0.01,1.23)	15.81(1.15,218.15);p=0.03928	1.27(0.14,11.52)						
GENUS_205	Firmicutes;D_2__Clostridia;D_3__Clostridiales;D_4__Ruminococcaceae;D_5__Ruminococcaceae UCG-008 (Combined: 7)	0.38(0.03,4.28)	5.91(0.55,63.12)	2.24(0.30,16.69)	0.31(0.03,3.48)	5.76(0.55,60.55)	1.77(0.24,13.09)			
GENUS_206	Actinobacteria;D_2__Coriobacteriia;D_3__Coriobacteriales;D_4__Atopobiaceae;D_5__Atopobium (Combined: 8)	1.48(0.20,10.86)	0.95(0.14,6.50)	1.41(0.30,6.65)	0.77(0.12,4.88)	2.12(0.35,12.88)	1.62(0.37,7.07)			
GENUS_208	Actinobacteria;D_2__Actinobacteria;D_3__Propionibacteriales;D_4__Propionibacteriaceae;D_5__Cutibacterium (Combined: 1)				0.35(0.00,Inf)	0.00(0.00,Inf)	0.00(0.00,Inf)			
GENUS_212	Firmicutes;D_2__Clostridia;D_3__Clostridiales;D_4__Lachnospiraceae;D_5__Shuttleworthia (Combined: 6)	1.26(0.14,11.10)	1.51(0.19,12.29)	1.91(0.34,10.68)	0.60(0.08,4.54)	2.75(0.38,19.79)	1.64(0.32,8.45)			
GENUS_214	Firmicutes;D_2__Clostridia;D_3__Clostridiales;D_4__Eubacteriaceae;D_5__Anaerofustis (Combined: 6)	0.72(0.10,5.18)	1.56(0.23,10.40)	1.12(0.23,5.41)	0.70(0.10,5.01)	1.76(0.27,11.44)	1.24(0.26,5.94)			
GENUS_215	Firmicutes;D_2__Clostridia;D_3__Clostridiales;D_4__Ruminococcaceae;D_5__Harryflintia (Combined: 3)				0.17(0.01,2.15)	1.55(0.15,15.95)	0.26(0.03,1.98)			
GENUS_217	Firmicutes;D_2__Clostridia;D_3__Clostridiales;D_4__Christensenellaceae;D_5__Christensenella (Combined: 1)	0.15(0.01,1.49)	0.77(0.10,5.85)	0.11(0.02,0.75);p=0.02445	0.08(0.01,0.88);p=0.03872	1.21(0.17,8.83)	0.10(0.01,0.72);p=0.02195	↓		↓
GENUS_218	Firmicutes;D_2__Clostridia;D_3__Clostridiales;D_4__Peptostreptococcaceae (Combined: 6)	0.43(0.01,15.89)	0.06(0.00,1.55)	0.03(0.00,0.43);p=0.0106	0.34(0.01,12.71)	0.08(0.00,1.75)	0.03(0.00,0.43);p=0.01057			↓
GENUS_226	Firmicutes;D_2__Bacilli;D_3__Bacillales;D_4__Bacillaceae;D_5__Bacillus (Combined: 4)	16.40(0.48,560.95)	2.42(0.06,100.25)	39.77(2.26,700.17);p=0.01185						
GENUS_231	Firmicutes;D_2__Clostridia;D_3__Clostridiales;D_4__Family XI;D_5__Parvimonas (Combined: 6)	3522812336.46(0.00,Inf)	0.00(0.00,Inf)	0.26(0.05,1.42)	1530140342751233.00(0.00,Inf)	0.00(0.00,Inf)	0.26(0.05,1.22)			
GENUS_234	Firmicutes;D_2__Clostridia;D_3__Clostridiales;D_4__Family XI;D_5__Finegoldia (Combined: 1)	0.73(0.03,16.29)	7.19(0.34,153.48)	5.22(0.40,67.41)	1.10(0.05,22.39)	8.04(0.37,172.21)	8.86(0.69,113.98)			

GENUS_236	Firmicutes;D_2__Clostridia;D_3__Clostridiales;D_4__Family XI;D_5__Anaerococcus (Combined: 6)	1.34(0.02,74.68)	1.32(0.03,63.17)	1.77(0.07,42.76)						
GENUS_239	Actinobacteria;D_2__Actinobacteria;D_3__Bifidobacteriales;D_4__Bifidobacteriaceae;D_5__Scardovia (Combined: 1)	2.98(0.07,122.77)	0.99(0.03,38.55)	2.94(0.17,49.74)						
GENUS_240	Firmicutes;D_2__Clostridia;D_3__Clostridiales;D_4__Lachnospiraceae;D_5__Lachnoclostridium 5 (Combined: 2)				1.49(0.06,35.19)	1690720394238479.00(0.00,Inf)	2520661685715829.00(0.00,Inf)			
GENUS_244	Firmicutes;D_2__Clostridia;D_3__Clostridiales;D_4__Lachnospiraceae;D_5__Lactonifactor (Combined: 2)	1.61(0.17,15.39)	0.36(0.04,3.09)	0.58(0.11,3.04)	1.75(0.18,17.38)	0.32(0.04,2.81)	0.56(0.11,2.87)			
GENUS_245	Firmicutes;D_2__Clostridia;D_3__Clostridiales;D_4__Lachnospiraceae;D_5__Lachnospiraceae NC2004 group (Combined: 7)	0.17(0.01,3.27)	11.11(0.64,193.24)	1.89(0.17,21.05)						
GENUS_248	Firmicutes;D_2__Erysipelotrichia;D_3__Erysipelotrichales;D_4__Erysipelotrichaceae;D_5__Coprobacillus (Combined: 1)	8.10(0.20,330.11)	0.01(0.00,0.47);p=0.01758	0.11(0.01,1.95)						
GENUS_249	Firmicutes;D_2__Clostridia;D_3__Clostridiales;D_4__Family XIII;D_5__S5-A14a (Combined: 1)	5.48(0.11,278.44)	0.30(0.01,13.80)	1.67(0.09,29.34)						
GENUS_256	Firmicutes;D_2__Clostridia;D_3__Clostridiales;D_4__Ruminococcaceae;D_5__Ruminiclostridium 1 (Combined: 9)	0.55(0.04,7.89)	0.64(0.05,8.31)	0.35(0.04,2.91)	1.31(0.10,17.92)	0.55(0.05,6.66)	0.72(0.09,5.67)			
GENUS_258	Firmicutes;D_2__Clostridia;D_3__Clostridiales;D_4__Ruminococcaceae;D_5__Papillibacter (Combined: 2)				0.67(0.12,3.82)	2.27(0.41,12.50)	1.53(0.38,6.19)			
GENUS_263	Firmicutes;D_2__Clostridia;D_3__Clostridiales;D_4__Lachnospiraceae;D_5__Oribacterium (Combined: 8)	35.75(0.58,2216.41)	1.53(0.03,93.49)	54.74(2.18,1374.62);p=0.01494						
GENUS_267	Actinobacteria;D_2__Actinobacteria;D_3__Actinomycetales;D_4__Actinomycetaceae;D_5__F0332 (Combined: 2)	5639307504.24(0.00,Inf)	0.00(0.00,Inf)	3.09(0.53,18.13)						
GENUS_273	Patescibacteria;D_2__Saccharimonadia;D_3__Saccharimonadales;D_4__TM7 phylum sp. oral clone FR058;D_5__ (Combined: 5)	1.08(0.04,30.04)	0.83(0.03,20.13)	0.89(0.07,11.64)	1.10(0.04,33.47)	0.87(0.03,23.64)	0.96(0.07,12.34)			
GENUS_276	Proteobacteria;D_2__Gammaproteobacteria;D_3__Betaproteobacteriales;D_4__Burkholderiaceae;D_5__Aquabacterium (Combined: 1)	2.14(0.09,52.20)	1.10(0.04,27.14)	2.34(0.21,25.56)	1.08(0.04,28.23)	2.68(0.08,89.77)	2.90(0.23,37.17)			
GENUS_284	Epsilonbacteraeota;D_2__Campylobacteria;D_3__Campylobacterales;D_4__Campylobacteraceae;D_5__Campylobacter (Combined: 4)	4.10(0.25,66.93)	1.37(0.09,21.40)	5.63(0.64,49.95)	5.97(0.33,108.09)	1.04(0.06,18.63)	6.19(0.71,54.31)			
GENUS_293	Bacteroidetes;D_2__Bacteroidia;D_3__Bacteroidales;D_4__Muribaculaceae;D_5__Porphyromonadaceae bacterium C941 (Combined: 2)	9873293082.95(0.00,Inf)	0.00(0.00,Inf)	1.61(0.06,47.26)						
GENUS_303	Proteobacteria;D_2__Alphaproteobacteria;D_3__Caulobacterales;D_4__Caulobacteraceae;D_5__Phenylobacterium (Combined: 1)	982555120208.25(0.00,Inf)	0.00(0.00,Inf)	2.33(0.16,33.41)	830541749552226.00(0.00,Inf)	0.00(0.00,Inf)	2.46(0.17,36.20)			
GENUS_323	Firmicutes;D_2__Clostridia;D_3__Clostridiales;D_4__Lachnospiraceae;D_5__Cuneatibacter (Combined: 1)	3719791955.83(0.00,Inf)	0.00(0.00,Inf)	2.20(0.10,48.67)						
GENUS_330	Proteobacteria;D_2__Gammaproteobacteria;D_3__Betaproteobacteriales;D_4__Burkholderiaceae;D_5__Lautropia (Combined: 2)	0.08(0.00,6.01)	26.69(0.36,2003.57)	2.04(0.04,100.38)						

Table 6.1 Species-level differences in the gut microbial communities by individual amplicon sequence variants (ASVs) for USA-only cohort participants with: pediatric-onset multiple sclerosis (MS) and unaffected controls, expressed as rate ratios

		Rate ratios (95%CIs) unadjusted	Rate ratios (95%CIs) age and sex adjusted	
Taxa Identifier (for internal use)	Species-level findings<br>(shown as: Phylum;Class;Order;Family;Genus;Species)	MS cases (vs controls)	MS cases (vs controls)	MS cases (vs controls)
ASV_3	Firmicutes;D_2__Bacilli;D_3__Lactobacillales;D_4__Streptococcaceae;D_5__Streptococcus;D_6__Streptococcus salivarius subsp. thermophilus;D_7__;D_8__;D_9__;D_10__;D_11__;D_12__;D_13__;D_14__	0.79(0.45,1.37)	1.06(0.62,1.80)	
ASV_4	Firmicutes;D_2__Clostridia;D_3__Clostridiales;D_4__Lachnospiraceae;D_5__Blautia	1.83(1.09,3.07);p=0.02241	1.85(1.10,3.11);p=0.0206	↑
ASV_5	Firmicutes;D_2__Clostridia;D_3__Clostridiales;D_4__Lachnospiraceae;D_5__Blautia	1.51(0.97,2.36)	1.58(1.02,2.47);p=0.04243	↑
ASV_7	Firmicutes;D_2__Clostridia;D_3__Clostridiales;D_4__Lachnospiraceae;D_5__Blautia	1.02(0.67,1.57)	1.03(0.67,1.58)	
ASV_8	Firmicutes;D_2__Clostridia;D_3__Clostridiales;D_4__Lachnospiraceae;D_5__Coprococcus 3	0.78(0.40,1.50)	0.78(0.40,1.50)	
ASV_9	Firmicutes;D_2__Clostridia;D_3__Clostridiales;D_4__Lachnospiraceae;D_5__Anaerostipes	1.12(0.67,1.85)	1.23(0.74,2.02)	
ASV_11	Firmicutes;D_2__Clostridia;D_3__Clostridiales;D_4__Lachnospiraceae;D_5__Dorea	0.96(0.56,1.63)	1.00(0.59,1.70)	
ASV_13	Firmicutes;D_2__Bacilli;D_3__Lactobacillales;D_4__Streptococcaceae;D_5__Streptococcus	2.20(1.22,3.97);p=0.00914	2.14(1.18,3.86);p=0.01192	↑
ASV_14	Firmicutes;D_2__Clostridia;D_3__Clostridiales;D_4__Lachnospiraceae;D_5__Blautia	1.08(0.64,1.82)	0.98(0.60,1.62)	
ASV_15	Firmicutes;D_2__Clostridia;D_3__Clostridiales;D_4__Lachnospiraceae;D_5__[Eubacterium] hallii group	1.31(0.66,2.58)	1.23(0.62,2.43)	
ASV_16	Firmicutes;D_2__Clostridia;D_3__Clostridiales;D_4__Lachnospiraceae;D_5__Coprococcus 1	0.64(0.31,1.32)	0.70(0.34,1.43)	
ASV_17	Firmicutes;D_2__Clostridia;D_3__Clostridiales;D_4__Ruminococcaceae;D_5__Ruminococcaceae UCG-004;D_6__uncultured bacterium;D_7__;D_8__;D_9__;D_10__;D_11__;D_12__;D_13__;D_14__	0.78(0.32,1.90)	0.76(0.31,1.84)	
ASV_20	Actinobacteria;D_2__Actinobacteria;D_3__Bifidobacteriales;D_4__Bifidobacteriaceae;D_5__Bifidobacterium	1.46(0.37,5.76)		
ASV_24	Bacteroidetes;D_2__Bacteroidia;D_3__Bacteroidales;D_4__Bacteroidaceae;D_5__Bacteroides	0.98(0.31,3.10)	1.34(0.43,4.16)	
ASV_25	Firmicutes;D_2__Clostridia;D_3__Clostridiales;D_4__Lachnospiraceae;D_5__Blautia	1.88(0.69,5.12)	1.86(0.69,5.01)	
ASV_26	Firmicutes;D_2__Clostridia;D_3__Clostridiales;D_4__Ruminococcaceae;D_5__Ruminiclostridium 5	1.45(0.59,3.56)	1.24(0.50,3.03)	
ASV_27	Actinobacteria;D_2__Coriobacteriia;D_3__Coriobacteriales;D_4__Coriobacteriaceae;D_5__Collinsella	1.20(0.56,2.56)	1.12(0.52,2.39)	
ASV_28	Firmicutes;D_2__Clostridia;D_3__Clostridiales;D_4__Lachnospiraceae;D_5__uncultured;D_6__uncultured Eubacterium sp.;D_7__;D_8__;D_9__;D_10__;D_11__;D_12__;D_13__;D_14__	1.17(0.50,2.71)	1.16(0.50,2.68)	
ASV_29	Firmicutes;D_2__Clostridia;D_3__Clostridiales;D_4__Ruminococcaceae;D_5__Ruminococcaceae NK4A214 group;D_6__gut metagenome;D_7__;D_8__;D_9__;D_10__;D_11__;D_12__;D_13__;D_14__	0.38(0.12,1.16)	0.43(0.14,1.29)	
ASV_31	Firmicutes;D_2__Clostridia;D_3__Clostridiales;D_4__Ruminococcaceae;D_5__[Eubacterium] coprostanoligenes group;D_6__uncultured organism;D_7__;D_8__;D_9__;D_10__;D_11__;D_12__;D_13__;D_14__		0.59(0.09,3.85)	
ASV_32	Firmicutes;D_2__Clostridia;D_3__Clostridiales;D_4__Christensenellaceae;D_5__Christensenellaceae R-7 group	4.10(0.51,32.60)		
ASV_33	Bacteroidetes;D_2__Bacteroidia;D_3__Bacteroidales;D_4__Rikenellaceae;D_5__Alistipes;D_6__Alistipes indistinctus YIT 12060;D_7__;D_8__;D_9__;D_10__;D_11__;D_12__;D_13__;D_14__	2.32(0.58,9.18)	2.23(0.57,8.79)	
ASV_34	Firmicutes;D_2__Negativicutes;D_3__Selenomonadales;D_4__Veillonellaceae;D_5__Dialister	2.59(0.69,9.71)	2.61(0.70,9.73)	

ASV_36	Bacteroidetes;D_2__Bacteroidia;D_3__Bacteroidales;D_4__Bacteroidaceae;D_5__Bacteroides;D_6__Bacteroides thetaiotaomicron;D_7__;D_8__;D_9__;D_10__;D_11__;D_12__;D_13__;D_14__	0.99(0.44,2.23)	0.93(0.41,2.10)	
ASV_37	Firmicutes;D_2__Clostridia;D_3__Clostridiales;D_4__Lachnospiraceae;D_5__[Ruminococcus] torques group;D_6__uncultured organism;D_7__;D_8__;D_9__;D_10__;D_11__;D_12__;D_13__;D_14__	0.64(0.28,1.48)	0.58(0.26,1.30)	
ASV_38	Firmicutes;D_2__Clostridia;D_3__Clostridiales;D_4__Ruminococcaceae;D_5__Ruminococcus 1;D_6__uncultured organism;D_7__;D_8__;D_9__;D_10__;D_11__;D_12__;D_13__;D_14__	0.49(0.17,1.40)		
ASV_39	Bacteroidetes;D_2__Bacteroidia;D_3__Bacteroidales;D_4__Rikenellaceae;D_5__Alistipes;D_6__uncultured organism;D_7__;D_8__;D_9__;D_10__;D_11__;D_12__;D_13__;D_14__	0.79(0.26,2.38)	0.96(0.32,2.88)	
ASV_40	Firmicutes;D_2__Clostridia;D_3__Clostridiales;D_4__Lachnospiraceae;D_5__[Eubacterium] hallii group	0.98(0.19,5.08)	1.32(0.27,6.46)	
ASV_41	Firmicutes;D_2__Clostridia;D_3__Clostridiales;D_4__Lachnospiraceae;D_5__Marvinbryantia	0.59(0.13,2.68)	0.34(0.08,1.51)	
ASV_43	Firmicutes;D_2__Clostridia;D_3__Clostridiales;D_4__Ruminococcaceae;D_5__Ruminococcaceae UCG-005;D_6__uncultured organism;D_7__;D_8__;D_9__;D_10__;D_11__;D_12__;D_13__;D_14__	1.26(0.39,4.06)	1.26(0.40,3.97)	
ASV_45	Firmicutes;D_2__Negativicutes;D_3__Selenomonadales;D_4__Acidaminococcaceae;D_5__Phascolarctobacterium		0.98(0.22,4.39)	
ASV_46	Firmicutes;D_2__Clostridia;D_3__Clostridiales;D_4__Lachnospiraceae;D_5__Agathobacter	1.48(0.82,2.67)	1.49(0.83,2.68)	
ASV_47	Firmicutes;D_2__Clostridia;D_3__Clostridiales;D_4__Ruminococcaceae;D_5__Faecalibacterium	1.22(0.76,1.94)	1.22(0.77,1.95)	
ASV_48	Firmicutes;D_2__Clostridia;D_3__Clostridiales;D_4__Ruminococcaceae;D_5__Intestinimonas;D_6__uncultured bacterium;D_7__;D_8__;D_9__;D_10__;D_11__;D_12__;D_13__;D_14__	0.63(0.12,3.32)		
ASV_50	Firmicutes;D_2__Clostridia;D_3__Clostridiales;D_4__Lachnospiraceae;D_5__[Ruminococcus] gauvreauii group	0.59(0.27,1.29)	0.62(0.28,1.36)	
ASV_51	Firmicutes;D_2__Clostridia;D_3__Clostridiales;D_4__Ruminococcaceae;D_5__Subdoligranulum	0.74(0.41,1.33)	0.74(0.41,1.33)	
ASV_52	Firmicutes;D_2__Clostridia;D_3__Clostridiales;D_4__Ruminococcaceae;D_5__Ruminiclostridium 5	1.29(0.20,8.13)	0.82(0.15,4.51)	
ASV_53	Firmicutes;D_2__Clostridia;D_3__Clostridiales;D_4__Lachnospiraceae;D_5__[Ruminococcus] torques group	1.29(0.60,2.77)	1.29(0.60,2.77)	
ASV_56	Firmicutes;D_2__Bacilli;D_3__Lactobacillales;D_4__Leuconostocaceae;D_5__Weissella	4.68(0.52,42.17)		
ASV_57	Firmicutes;D_2__Clostridia;D_3__Clostridiales;D_4__Family XIII;D_5__[Eubacterium] nodatum group;D_6__uncultured bacterium;D_7__;D_8__;D_9__;D_10__;D_11__;D_12__;D_13__;D_14__	3.21(0.13,77.30)		
ASV_58	Bacteroidetes;D_2__Bacteroidia;D_3__Bacteroidales;D_4__Rikenellaceae;D_5__Alistipes	1.47(0.64,3.38)		
ASV_60	Firmicutes;D_2__Clostridia;D_3__Clostridiales;D_4__Lachnospiraceae;D_5__Blautia;D_6__uncultured Blautia sp.;D_7__;D_8__;D_9__;D_10__;D_11__;D_12__;D_13__;D_14__	0.39(0.17,0.90);p=0.02777	0.60(0.26,1.38)	
ASV_62	Firmicutes;D_2__Clostridia;D_3__Clostridiales;D_4__Lachnospiraceae;D_5__Agathobacter;D_6__Eubacterium ramulus;D_7__;D_8__;D_9__;D_10__;D_11__;D_12__;D_13__;D_14__	0.55(0.26,1.19)	0.55(0.25,1.19)	
ASV_65	Firmicutes;D_2__Clostridia;D_3__Clostridiales;D_4__Ruminococcaceae;D_5__uncultured;D_6__uncultured Clostridium sp.;D_7__;D_8__;D_9__;D_10__;D_11__;D_12__;D_13__;D_14__	0.52(0.14,1.89)	0.31(0.09,1.12)	
ASV_66	Firmicutes;D_2__Clostridia;D_3__Clostridiales;D_4__Ruminococcaceae;D_5__Subdoligranulum	0.68(0.33,1.38)	0.70(0.34,1.43)	
ASV_68	Firmicutes;D_2__Clostridia;D_3__Clostridiales;D_4__Family XIII;D_5__Family XIII AD3011 group;D_6__uncultured Eubacterium sp.;D_7__;D_8__;D_9__;D_10__;D_11__;D_12__;D_13__;D_14__	0.31(0.10,0.98);p=0.04588	0.32(0.10,1.02)	
ASV_69	Bacteroidetes;D_2__Bacteroidia;D_3__Bacteroidales;D_4__Rikenellaceae;D_5__Alistipes;D_6__Alistipes sp. N15.MGS-157;D_7__;D_8__;D_9__;D_10__;D_11__;D_12__;D_13__;D_14__	0.64(0.13,3.20)	0.84(0.17,4.15)	
ASV_70	Firmicutes;D_2__Bacilli;D_3__Lactobacillales;D_4__Streptococcaceae;D_5__Streptococcus;D_6__Streptococcus anginosus subsp. anginosus;D_7__;D_8__;D_9__;D_10__;D_11__;D_12__;D_13__;D_14__	0.40(0.09,1.80)	0.46(0.11,2.03)	
ASV_71	Firmicutes;D_2__Clostridia;D_3__Clostridiales;D_4__Christensenellaceae;D_5__Christensenellaceae R-7 group		2.23(0.26,18.78)	
ASV_72	Firmicutes;D_2__Clostridia;D_3__Clostridiales;D_4__Lachnospiraceae;D_5__Eisenbergiella;D_6__uncultured organism;D_7__;D_8__;D_9__;D_10__;D_11__;D_12__;D_13__;D_14__	1.04(0.42,2.59)		
ASV_73	Firmicutes;D_2__Bacilli;D_3__Lactobacillales;D_4__Streptococcaceae;D_5__Streptococcus	2.20(1.10,4.39);p=0.02513	2.06(1.04,4.09);p=0.0395	↑
ASV_74	Firmicutes;D_2__Bacilli;D_3__Bacillales;D_4__Family XI;D_5__Gemella	1.24(0.53,2.90)	1.18(0.51,2.77)	
ASV_75	Firmicutes;D_2__Clostridia;D_3__Clostridiales;D_4__Lachnospiraceae;D_5__Fusicatenibacter	0.96(0.58,1.56)	0.96(0.59,1.57)	
ASV_80	Firmicutes;D_2__Clostridia;D_3__Clostridiales;D_4__Peptostreptococcaceae;D_5__Romboutsia	1.50(0.82,2.74)	1.63(0.89,2.99)	

ASV_81	Bacteroidetes;D_2__Bacteroidia;D_3__Bacteroidales;D_4__Tannerellaceae;D_5__Parabacteroides;D_6__Parabacteroides distasonis;D_7__;D_8__;D_9__;D_10__;D_11__;D_12__;D_13__;D_14__	3.19(0.75,13.52)		
ASV_83	Firmicutes;D_2__Clostridia;D_3__Clostridiales;D_4__Lachnospiraceae;D_5__Roseburia;D_6__gut metagenome;D_7__;D_8__;D_9__;D_10__;D_11__;D_12__;D_13__;D_14__	0.59(0.22,1.61)	0.62(0.23,1.65)	
ASV_85	Firmicutes;D_2__Clostridia;D_3__Clostridiales;D_4__Ruminococcaceae;D_5__Ruminococcaceae UCG-002;D_6__uncultured rumen bacterium;D_7__;D_8__;D_9__;D_10__;D_11__;D_12__;D_13__;D_14__	0.28(0.07,1.15)	0.30(0.07,1.22)	
ASV_86	Firmicutes;D_2__Clostridia;D_3__Clostridiales;D_4__Ruminococcaceae;D_5__Ruminococcaceae UCG-005;D_6__human gut metagenome;D_7__;D_8__;D_9__;D_10__;D_11__;D_12__;D_13__;D_14__	0.42(0.16,1.09)	0.40(0.16,1.03)	
ASV_87	Firmicutes;D_2__Clostridia;D_3__Clostridiales;D_4__Ruminococcaceae;D_5__UBA1819;D_6__uncultured organism;D_7__;D_8__;D_9__;D_10__;D_11__;D_12__;D_13__;D_14__	0.68(0.35,1.34)	0.66(0.34,1.29)	
ASV_88	Firmicutes;D_2__Erysipelotrichia;D_3__Erysipelotrichales;D_4__Erysipelotrichaceae;D_5__[Clostridium] innocuum group	2.10(0.91,4.89)	2.22(0.96,5.14)	
ASV_89	Firmicutes;D_2__Clostridia;D_3__Clostridiales;D_4__Family XIII;D_5__Family XIII AD3011 group;D_6__uncultured bacterium;D_7__;D_8__;D_9__;D_10__;D_11__;D_12__;D_13__;D_14__	0.60(0.29,1.27)	0.84(0.40,1.73)	
ASV_93	Firmicutes;D_2__Clostridia;D_3__Clostridiales;D_4__Lachnospiraceae;D_5__Roseburia;D_6__metagenome;D_7__;D_8__;D_9__;D_10__;D_11__;D_12__;D_13__;D_14__	1.16(0.23,5.90)	0.76(0.15,3.79)	
ASV_94	Firmicutes;D_2__Clostridia;D_3__Clostridiales;D_4__Ruminococcaceae;D_5__Ruminococcaceae UCG-013;D_6__uncultured bacterium;D_7__;D_8__;D_9__;D_10__;D_11__;D_12__;D_13__;D_14__	0.87(0.04,20.56)		
ASV_95	Bacteroidetes;D_2__Bacteroidia;D_3__Bacteroidales;D_4__Rikenellaceae;D_5__Alistipes	0.90(0.32,2.51)	0.95(0.34,2.67)	
ASV_96	Firmicutes;D_2__Clostridia;D_3__Clostridiales;D_4__Lachnospiraceae;D_5__Hungatella	1.13(0.20,6.24)	1.33(0.27,6.58)	
ASV_98	Firmicutes;D_2__Clostridia;D_3__Clostridiales;D_4__Lachnospiraceae;D_5__Lachnospiraceae FCS020 group;D_6__uncultured Clostridium sp.;D_7__;D_8__;D_9__;D_10__;D_11__;D_12__;D_13__;D_14__	0.65(0.22,1.90)	0.48(0.17,1.38)	
ASV_99	Firmicutes;D_2__Clostridia;D_3__Clostridiales;D_4__Lachnospiraceae;D_5__Hungatella;D_6__uncultured bacterium;D_7__;D_8__;D_9__;D_10__;D_11__;D_12__;D_13__;D_14__	0.12(0.02,0.65);p=0.01339	0.17(0.03,0.84);p=0.03015	↓
ASV_100	Firmicutes;D_2__Clostridia;D_3__Clostridiales;D_4__Family XIII;D_5__Family XIII AD3011 group	2.67(0.40,17.79)	2.84(0.46,17.38)	
ASV_101	Firmicutes;D_2__Clostridia;D_3__Clostridiales;D_4__Lachnospiraceae;D_5__Dorea	0.95(0.58,1.54)	0.91(0.56,1.49)	
ASV_102	Firmicutes;D_2__Negativicutes;D_3__Selenomonadales;D_4__Veillonellaceae;D_5__Veillonella	1.01(0.41,2.47)	0.58(0.25,1.36)	
ASV_104	Bacteroidetes;D_2__Bacteroidia;D_3__Bacteroidales;D_4__Marinifilaceae;D_5__Odoribacter	1.07(0.43,2.66)		
ASV_105	Firmicutes;D_2__Bacilli;D_3__Lactobacillales;D_4__Streptococcaceae;D_5__Streptococcus	2.88(0.35,24.02)		
ASV_106	Firmicutes;D_2__Clostridia;D_3__Clostridiales;D_4__Ruminococcaceae;D_5__Ruminiclostridium 6;D_6__uncultured bacterium;D_7__;D_8__;D_9__;D_10__;D_11__;D_12__;D_13__;D_14__	2.46(0.73,8.32)	2.14(0.65,6.99)	
ASV_107	Firmicutes;D_2__Clostridia;D_3__Clostridiales;D_4__Lachnospiraceae;D_5__CAG-56;D_6__uncultured bacterium;D_7__;D_8__;D_9__;D_10__;D_11__;D_12__;D_13__;D_14__	0.77(0.28,2.12)	0.80(0.29,2.21)	
ASV_110	Firmicutes;D_2__Clostridia;D_3__Clostridiales;D_4__Ruminococcaceae;D_5__Negativibacillus;D_6__uncultured bacterium;D_7__;D_8__;D_9__;D_10__;D_11__;D_12__;D_13__;D_14__	2.15(0.49,9.53)	3.16(0.74,13.56)	
ASV_113	Firmicutes;D_2__Clostridia;D_3__Clostridiales;D_4__Family XIII;D_5__Family XIII UCG-001;D_6__uncultured bacterium;D_7__;D_8__;D_9__;D_10__;D_11__;D_12__;D_13__;D_14__	28.66(2.14,384.58);p=0.01132	26.84(1.96,367.45);p=0.01372	↑
ASV_117	Firmicutes;D_2__Clostridia;D_3__Clostridiales;D_4__Lachnospiraceae		0.45(0.08,2.48)	
ASV_119	Bacteroidetes;D_2__Bacteroidia;D_3__Bacteroidales;D_4__Bacteroidaceae;D_5__Bacteroides	2.33(0.77,7.04)	1.63(0.55,4.86)	
ASV_120	Bacteroidetes;D_2__Bacteroidia;D_3__Bacteroidales;D_4__Marinifilaceae;D_5__Odoribacter;D_6__gut metagenome;D_7__;D_8__;D_9__;D_10__;D_11__;D_12__;D_13__;D_14__	9.09(0.24,338.88)		
ASV_122	Firmicutes;D_2__Clostridia;D_3__Clostridiales;D_4__Lachnospiraceae;D_5__Eisenbergiella	8.38(0.59,119.35)	31.92(1.55,659.34);p=0.02499	↑
ASV_123	Firmicutes;D_2__Clostridia;D_3__Clostridiales;D_4__Ruminococcaceae;D_5__uncultured	0.81(0.09,7.20)		
ASV_127	Actinobacteria;D_2__Actinobacteria;D_3__Actinomycetales;D_4__Actinomycetaceae;D_5__Actinomyces	1.36(0.74,2.50)	1.36(0.74,2.50)	
ASV_128	Firmicutes;D_2__Clostridia;D_3__Clostridiales;D_4__Family XIII;D_5__[Eubacterium] brachy group;D_6__uncultured bacterium;D_7__;D_8__;D_9__;D_10__;D_11__;D_12__;D_13__;D_14__	1.67(0.64,4.34)	1.57(0.61,4.06)	
ASV_129	Bacteroidetes;D_2__Bacteroidia;D_3__Bacteroidales;D_4__Tannerellaceae;D_5__Parabacteroides;D_6__Parabacteroides distasonis;D_7__;D_8__;D_9__;D_10__;D_11__;D_12__;D_13__;D_14__	5.63(0.43,73.39)	15.66(1.51,162.83);p=0.02127	↑
ASV_130	Firmicutes;D_2__Clostridia;D_3__Clostridiales;D_4__Ruminococcaceae;D_5__uncultured	0.75(0.21,2.65)		

ASV_131	Firmicutes;D_2__Clostridia;D_3__Clostridiales;D_4__Lachnospiraceae;D_5__Lachnoclostridium	0.96(0.45,2.02)	1.07(0.51,2.22)	
ASV_132	Firmicutes;D_2__Clostridia;D_3__Clostridiales;D_4__Lachnospiraceae;D_5__[Eubacterium] eligens group	1.10(0.48,2.52)	0.97(0.42,2.20)	
ASV_134	Firmicutes;D_2__Clostridia;D_3__Clostridiales;D_4__Ruminococcaceae;D_5__Ruminococcaceae UCG-013;D_6__uncultured organism;D_7__;D_8__;D_9__;D_10__;D_11__;D_12__;D_13__;D_14__	1.10(0.62,1.96)	1.12(0.63,1.97)	
ASV_135	Firmicutes;D_2__Clostridia;D_3__Clostridiales;D_4__Lachnospiraceae;D_5__Roseburia	0.54(0.25,1.15)	0.54(0.26,1.13)	
ASV_136	Firmicutes;D_2__Clostridia;D_3__Clostridiales;D_4__Ruminococcaceae;D_5__Ruminiclostridium 5;D_6__uncultured organism;D_7__;D_8__;D_9__;D_10__;D_11__;D_12__;D_13__;D_14__	0.73(0.42,1.25)	0.72(0.42,1.23)	
ASV_137	Firmicutes;D_2__Negativicutes;D_3__Selenomonadales;D_4__Veillonellaceae;D_5__Veillonella	1.63(0.63,4.21)	1.56(0.60,4.03)	
ASV_138	Firmicutes;D_2__Clostridia;D_3__Clostridiales;D_4__Christensenellaceae;D_5__Christensenellaceae R-7 group;D_6__metagenome;D_7__;D_8__;D_9__;D_10__;D_11__;D_12__;D_13__;D_14__	0.65(0.10,4.16)		
ASV_140	Firmicutes;D_2__Clostridia;D_3__Clostridiales;D_4__Family XIII;D_5__Family XIII AD3011 group;D_6__uncultured bacterium;D_7__;D_8__;D_9__;D_10__;D_11__;D_12__;D_13__;D_14__	0.62(0.03,13.41)	0.64(0.03,12.69)	
ASV_141	Firmicutes;D_2__Clostridia;D_3__Clostridiales;D_4__Ruminococcaceae;D_5__Ruminiclostridium 5;D_6__gut metagenome;D_7__;D_8__;D_9__;D_10__;D_11__;D_12__;D_13__;D_14__	1.84(0.93,3.65)	1.93(0.98,3.78)	
ASV_144	Firmicutes;D_2__Bacilli;D_3__Lactobacillales;D_4__Carnobacteriaceae;D_5__Granulicatella	1.34(0.61,2.96)	1.26(0.58,2.77)	
ASV_145	Bacteroidetes;D_2__Bacteroidia;D_3__Bacteroidales;D_4__Rikenellaceae;D_5__Alistipes;D_6__Faecalibacterium prausnitzii;D_7__;D_8__;D_9__;D_10__;D_11__;D_12__;D_13__;D_14__	0.34(0.03,4.36)		
ASV_146	Firmicutes;D_2__Negativicutes;D_3__Selenomonadales;D_4__Veillonellaceae;D_5__Veillonella	1.60(0.47,5.38)	1.03(0.31,3.45)	
ASV_147	Firmicutes;D_2__Clostridia;D_3__Clostridiales;D_4__Ruminococcaceae;D_5__Ruminococcaceae UCG-010;D_6__gut metagenome;D_7__;D_8__;D_9__;D_10__;D_11__;D_12__;D_13__;D_14__	0.31(0.01,6.60)		
ASV_148	Firmicutes;D_2__Clostridia;D_3__Clostridiales;D_4__Ruminococcaceae	1.56(0.51,4.74)	1.69(0.56,5.14)	
ASV_150	Firmicutes;D_2__Bacilli;D_3__Lactobacillales;D_4__Streptococcaceae;D_5__Lactococcus	0.81(0.38,1.72)	0.84(0.40,1.75)	
ASV_153	Firmicutes;D_2__Clostridia;D_3__Clostridiales;D_4__Lachnospiraceae;D_5__Lachnospiraceae NK4A136 group;D_6__uncultured organism;D_7__;D_8__;D_9__;D_10__;D_11__;D_12__;D_13__;D_14__	0.68(0.30,1.53)	0.73(0.33,1.62)	
ASV_154	Bacteroidetes;D_2__Bacteroidia;D_3__Bacteroidales;D_4__Bacteroidaceae;D_5__Bacteroides	1.54(0.48,4.93)	1.17(0.37,3.65)	
ASV_155	Firmicutes;D_2__Erysipelotrichia;D_3__Erysipelotrichales;D_4__Erysipelotrichaceae;D_5__Erysipelotrichaceae UCG-003	0.77(0.39,1.54)	0.77(0.39,1.54)	
ASV_157	Firmicutes;D_2__Clostridia;D_3__Clostridiales;D_4__Peptostreptococcaceae;D_5__Peptostreptococcus;D_6__uncultured organism;D_7__;D_8__;D_9__;D_10__;D_11__;D_12__;D_13__;D_14__	0.30(0.03,3.27)		
ASV_158	Firmicutes;D_2__Clostridia;D_3__Clostridiales;D_4__Lachnospiraceae;D_5__Lachnoclostridium;D_6__human gut metagenome;D_7__;D_8__;D_9__;D_10__;D_11__;D_12__;D_13__;D_14__	1.62(0.80,3.31)	1.42(0.70,2.89)	
ASV_159	Firmicutes;D_2__Clostridia;D_3__Clostridiales;D_4__Christensenellaceae;D_5__Christensenellaceae R-7 group;D_6__Clostridiales bacterium Marseille-P2846;D_7__;D_8__;D_9__;D_10__;D_11__;D_12__;D_13__;D_14__	1.17(0.05,25.85)	0.90(0.04,22.83)	
ASV_160	Firmicutes;D_2__Clostridia;D_3__Clostridiales;D_4__Ruminococcaceae	0.38(0.06,2.47)	0.50(0.08,3.11)	
ASV_161	Firmicutes;D_2__Clostridia;D_3__Clostridiales;D_4__Defluviitaleaceae;D_5__Defluviitaleaceae UCG-011;D_6__uncultured bacterium;D_7__;D_8__;D_9__;D_10__;D_11__;D_12__;D_13__;D_14__	1.99(0.37,10.64)	4.97(0.96,25.71)	
ASV_163	Firmicutes;D_2__Clostridia;D_3__Clostridiales;D_4__Ruminococcaceae	1.02(0.20,5.23)	1.16(0.23,5.84)	
ASV_165	Firmicutes;D_2__Clostridia;D_3__Clostridiales;D_4__Lachnospiraceae;D_5__Roseburia;D_6__uncultured Roseburia sp.;D_7__;D_8__;D_9__;D_10__;D_11__;D_12__;D_13__;D_14__		0.98(0.09,10.22)	
ASV_166	Firmicutes;D_2__Clostridia;D_3__Clostridiales;D_4__Lachnospiraceae;D_5__Lachnoclostridium;D_6__uncultured Clostridium sp.;D_7__;D_8__;D_9__;D_10__;D_11__;D_12__;D_13__;D_14__	4.44(0.29,67.86)		
ASV_167	Firmicutes;D_2__Clostridia;D_3__Clostridiales;D_4__Clostridiales vadinBB60 group;D_5__uncultured Thermoanaerobacterales bacterium;D_6__;D_7__;D_8__;D_9__;D_10__;D_11__;D_12__;D_13__;D_14__	8.42(0.71,99.24)		
ASV_168	Firmicutes;D_2__Clostridia;D_3__Clostridiales;D_4__Lachnospiraceae	0.59(0.21,1.66)	0.59(0.21,1.68)	
ASV_169	Firmicutes;D_2__Clostridia;D_3__Clostridiales;D_4__Lachnospiraceae;D_5__[Eubacterium] eligens group	0.68(0.21,2.19)		
ASV_170	Firmicutes;D_2__Clostridia;D_3__Clostridiales;D_4__Lachnospiraceae;D_5__Lachnospiraceae NK4A136 group	0.92(0.36,2.39)	0.74(0.29,1.89)	
ASV_171	Firmicutes;D_2__Clostridia;D_3__Clostridiales;D_4__Ruminococcaceae;D_5__Ruminococcaceae NK4A214 group;D_6__uncultured organism;D_7__;D_8__;D_9__;D_10__;D_11__;D_12__;D_13__;D_14__	1.98(0.34,11.42)	1.70(0.31,9.40)	

ASV_172	Firmicutes;D_2__Erysipelotrichia;D_3__Erysipelotrichales;D_4__Erysipelotrichaceae;D_5__Holdemania	1.00(0.44,2.25)	0.99(0.44,2.24)	
ASV_173	Firmicutes;D_2__Clostridia;D_3__Clostridiales;D_4__Lachnospiraceae;D_5__Sellimonas;D_6__uncultured bacterium;D_7__;D_8__;D_9__;D_10__;D_11__;D_12__;D_13__;D_14__	1.63(0.23,11.42)	16.20(1.71,153.36);p=0.01515	↑
ASV_174	Firmicutes;D_2__Clostridia;D_3__Clostridiales;D_4__Lachnospiraceae;D_5__uncultured;D_6__uncultured organism;D_7__;D_8__;D_9__;D_10__;D_11__;D_12__;D_13__;D_14__	2.89(0.96,8.68)	3.03(1.01,9.09);p=0.04822	↑
ASV_175	Firmicutes;D_2__Clostridia;D_3__Clostridiales;D_4__Christensenellaceae;D_5__uncultured;D_6__uncultured bacterium;D_7__;D_8__;D_9__;D_10__;D_11__;D_12__;D_13__;D_14__	0.46(0.14,1.49)	0.44(0.14,1.43)	
ASV_177	Firmicutes;D_2__Clostridia;D_3__Clostridiales;D_4__Christensenellaceae;D_5__Christensenellaceae R-7 group	0.06(0.00,0.72);p=0.02723	0.10(0.01,1.19)	
ASV_179	Firmicutes;D_2__Clostridia;D_3__Clostridiales;D_4__Ruminococcaceae;D_5__DTU089	1.20(0.56,2.58)	1.21(0.56,2.59)	
ASV_180	Actinobacteria;D_2__Coriobacteriia;D_3__Coriobacteriales;D_4__Coriobacteriales Incertae Sedis;D_5__uncultured;D_6__gut metagenome;D_7__;D_8__;D_9__;D_10__;D_11__;D_12__;D_13__;D_14__	1.25(0.37,4.22)	0.94(0.28,3.16)	
ASV_181	Firmicutes;D_2__Clostridia;D_3__Clostridiales;D_4__Ruminococcaceae;D_5__Anaerotruncus;D_6__Anaerotruncus sp. AT3;D_7__;D_8__;D_9__;D_10__;D_11__;D_12__;D_13__;D_14__	0.49(0.04,5.70)		
ASV_182	Firmicutes;D_2__Clostridia;D_3__Clostridiales;D_4__Lachnospiraceae	0.76(0.38,1.51)	0.80(0.41,1.59)	
ASV_183	Actinobacteria;D_2__Coriobacteriia;D_3__Coriobacteriales;D_4__Eggerthellaceae	0.30(0.11,0.79);p=0.01545	0.44(0.17,1.14)	
ASV_184	Firmicutes;D_2__Clostridia;D_3__Clostridiales;D_4__Ruminococcaceae;D_5__uncultured	0.27(0.05,1.39)	0.33(0.07,1.63)	
ASV_185	Firmicutes;D_2__Clostridia;D_3__Clostridiales;D_4__Ruminococcaceae;D_5__Anaerotruncus;D_6__Anaerotruncus rubiinfantis;D_7__;D_8__;D_9__;D_10__;D_11__;D_12__;D_13__;D_14__	1.55(0.37,6.59)	1.01(0.25,4.09)	
ASV_189	Firmicutes;D_2__Clostridia;D_3__Clostridiales;D_4__Lachnospiraceae;D_5__[Eubacterium] ventriosum group	1.02(0.46,2.24)	0.96(0.44,2.12)	
ASV_191	Proteobacteria;D_2__Gammaproteobacteria;D_3__Pseudomonadales;D_4__Pseudomonadaceae;D_5__Pseudomonas	1.07(0.21,5.41)	1.31(0.26,6.54)	
ASV_192	Firmicutes;D_2__Clostridia;D_3__Clostridiales;D_4__Lachnospiraceae;D_5__GCA-900066575	2.12(0.63,7.13)	2.21(0.66,7.37)	
ASV_194	Firmicutes;D_2__Clostridia;D_3__Clostridiales;D_4__Ruminococcaceae;D_5__Fournierella;D_6__uncultured bacterium;D_7__;D_8__;D_9__;D_10__;D_11__;D_12__;D_13__;D_14__	1.66(0.42,6.58)	1.21(0.31,4.70)	
ASV_195	Firmicutes;D_2__Clostridia;D_3__Clostridiales;D_4__Ruminococcaceae;D_5__Ruminiclostridium 5;D_6__gut metagenome;D_7__;D_8__;D_9__;D_10__;D_11__;D_12__;D_13__;D_14__		2995441278180776.00(0.00,Inf)	
ASV_196	Firmicutes;D_2__Clostridia;D_3__Clostridiales;D_4__Ruminococcaceae;D_5__Angelakisella;D_6__uncultured bacterium;D_7__;D_8__;D_9__;D_10__;D_11__;D_12__;D_13__;D_14__	2.92(0.59,14.41)	4.18(0.86,20.21)	
ASV_197	Firmicutes;D_2__Erysipelotrichia;D_3__Erysipelotrichales;D_4__Erysipelotrichaceae;D_5__Erysipelatoclostridium;D_6__unidentified;D_7__;D_8__;D_9__;D_10__;D_11__;D_12__;D_13__;D_14__	0.50(0.05,5.31)		
ASV_198	Actinobacteria;D_2__Coriobacteriia;D_3__Coriobacteriales;D_4__Eggerthellaceae;D_5__Gordonibacter;D_6__unidentified;D_7__;D_8__;D_9__;D_10__;D_11__;D_12__;D_13__;D_14__	0.48(0.20,1.15)	0.66(0.29,1.54)	
ASV_199	Firmicutes;D_2__Clostridia;D_3__Clostridiales;D_4__Lachnospiraceae;D_5__Lachnospiraceae UCG-010;D_6__uncultured organism;D_7__;D_8__;D_9__;D_10__;D_11__;D_12__;D_13__;D_14__	0.72(0.23,2.25)	0.85(0.28,2.63)	
ASV_202	Firmicutes;D_2__Clostridia;D_3__Clostridiales;D_4__Lachnospiraceae;D_5__GCA-900066755;D_6__uncultured bacterium;D_7__;D_8__;D_9__;D_10__;D_11__;D_12__;D_13__;D_14__	1.82(0.33,9.95)		
ASV_203	Firmicutes;D_2__Clostridia;D_3__Clostridiales;D_4__Christensenellaceae;D_5__Christensenellaceae R-7 group;D_6__uncultured soil bacterium;D_7__;D_8__;D_9__;D_10__;D_11__;D_12__;D_13__;D_14__	0.59(0.05,7.04)		
ASV_205	Firmicutes;D_2__Clostridia;D_3__Clostridiales;D_4__Christensenellaceae;D_5__Christensenellaceae R-7 group	2.23(0.36,13.83)		
ASV_206	Actinobacteria;D_2__Coriobacteriia;D_3__Coriobacteriales;D_4__Eggerthellaceae;D_5__Gordonibacter;D_6__uncultured bacterium;D_7__;D_8__;D_9__;D_10__;D_11__;D_12__;D_13__;D_14__		1.70(0.46,6.29)	
ASV_207	Firmicutes;D_2__Clostridia;D_3__Clostridiales;D_4__Lachnospiraceae;D_5__Lachnoclostridium	3.63(0.42,31.10)		
ASV_209	Firmicutes;D_2__Clostridia;D_3__Clostridiales;D_4__Lachnospiraceae;D_5__GCA-900066575;D_6__uncultured organism;D_7__;D_8__;D_9__;D_10__;D_11__;D_12__;D_13__;D_14__	1.27(0.21,7.79)		
ASV_211	Firmicutes;D_2__Clostridia;D_3__Clostridiales;D_4__Christensenellaceae;D_5__uncultured;D_6__uncultured bacterium;D_7__;D_8__;D_9__;D_10__;D_11__;D_12__;D_13__;D_14__	3.18(0.31,32.97)	2.02(0.21,19.36)	
ASV_212	Actinobacteria;D_2__Coriobacteriia;D_3__Coriobacteriales;D_4__Eggerthellaceae;D_5__Adlercreutzia;D_6__uncultured bacterium;D_7__;D_8__;D_9__;D_10__;D_11__;D_12__;D_13__;D_14__	1.01(0.46,2.21)	0.84(0.39,1.84)	
ASV_213	Firmicutes;D_2__Clostridia;D_3__DTU014;D_4__uncultured bacterium;D_5__;D_6__;D_7__;D_8__;D_9__;D_10__;D_11__;D_12__;D_13__;D_14__	2.01(0.40,10.06)	1.27(0.27,5.95)	
ASV_214	Actinobacteria;D_2__Actinobacteria;D_3__Actinomycetales;D_4__Actinomycetaceae;D_5__Actinomyces	1.05(0.10,11.53)	1.05(0.11,10.37)	

ASV_215	Firmicutes;D_2__Clostridia;D_3__Clostridiales;D_4__Lachnospiraceae;D_5__Lachnoclostridium	1.28(0.45,3.70)	1.29(0.44,3.72)	
ASV_216	Firmicutes;D_2__Clostridia;D_3__Clostridiales;D_4__Lachnospiraceae;D_5__Lachnospiraceae NK4A136 group;D_6__uncultured organism;D_7__;D_8__;D_9__;D_10__;D_11__;D_12__;D_13__;D_14__	0.39(0.17,0.92);p=0.03166	0.60(0.26,1.38)	
ASV_218	Firmicutes;D_2__Clostridia;D_3__Clostridiales;D_4__Ruminococcaceae;D_5__Ruminococcaceae UCG-003;D_6__uncultured organism;D_7__;D_8__;D_9__;D_10__;D_11__;D_12__;D_13__;D_14__	0.49(0.22,1.11)	0.49(0.22,1.11)	
ASV_219	Firmicutes;D_2__Clostridia;D_3__Clostridiales;D_4__Christensenellaceae;D_5__Christensenellaceae R-7 group	1.70(0.41,6.98)		
ASV_220	Firmicutes;D_2__Clostridia;D_3__Clostridiales;D_4__Ruminococcaceae;D_5__Hydrogenoanaerobacterium	1.31(0.10,17.00)		
ASV_222	Firmicutes;D_2__Clostridia;D_3__Clostridiales;D_4__Lachnospiraceae;D_5__uncultured;D_6__intestinal bacterium CG19-1;D_7__;D_8__;D_9__;D_10__;D_11__;D_12__;D_13__;D_14__	0.88(0.20,3.90)	1.14(0.26,4.97)	
ASV_223	Firmicutes;D_2__Clostridia;D_3__Clostridiales;D_4__Christensenellaceae;D_5__Catabacter;D_6__Christensenella massiliensis;D_7__;D_8__;D_9__;D_10__;D_11__;D_12__;D_13__;D_14__	0.20(0.02,2.34)	4.58(0.46,45.78)	
ASV_224	Firmicutes;D_2__Clostridia;D_3__Clostridiales;D_4__Ruminococcaceae;D_5__Ruminococcaceae UCG-007	2.06(0.26,16.36)	3.04(0.37,25.15)	
ASV_225	Firmicutes;D_2__Clostridia;D_3__Clostridiales;D_4__Lachnospiraceae;D_5__uncultured	0.31(0.03,2.93)	0.24(0.03,2.15)	
ASV_226	Proteobacteria;D_2__Deltaproteobacteria;D_3__Desulfovibrionales;D_4__Desulfovibrionaceae;D_5__Bilophila;D_6__uncultured bacterium;D_7__;D_8__;D_9__;D_10__;D_11__;D_12__;D_13__;D_14__	0.01(0.00,0.45);p=0.01836		
ASV_227	Firmicutes;D_2__Clostridia;D_3__Clostridiales;D_4__Lachnospiraceae;D_5__Lachnospiraceae NK4A136 group;D_6__uncultured organism;D_7__;D_8__;D_9__;D_10__;D_11__;D_12__;D_13__;D_14__	0.49(0.21,1.14)	0.52(0.22,1.22)	
ASV_229	Firmicutes;D_2__Clostridia;D_3__Clostridiales;D_4__Ruminococcaceae;D_5__[Eubacterium] coprostanoligenes group;D_6__human gut metagenome;D_7__;D_8__;D_9__;D_10__;D_11__;D_12__;D_13__;D_14__	0.91(0.09,8.80)		
ASV_234	Firmicutes;D_2__Clostridia;D_3__Clostridiales;D_4__Ruminococcaceae;D_5__Ruminiclostridium 5;D_6__uncultured bacterium;D_7__;D_8__;D_9__;D_10__;D_11__;D_12__;D_13__;D_14__	0.65(0.09,4.62)	0.45(0.06,3.16)	
ASV_236	Firmicutes;D_2__Erysipelotrichia;D_3__Erysipelotrichales;D_4__Erysipelotrichaceae;D_5__Dielma;D_6__uncultured bacterium;D_7__;D_8__;D_9__;D_10__;D_11__;D_12__;D_13__;D_14__	0.49(0.05,4.87)	0.50(0.05,4.80)	
ASV_237	Firmicutes;D_2__Clostridia;D_3__Clostridiales;D_4__Lachnospiraceae;D_5__Lachnospira	0.59(0.16,2.14)	0.53(0.15,1.83)	
ASV_238	Firmicutes;D_2__Clostridia;D_3__Clostridiales;D_4__Ruminococcaceae	0.71(0.12,4.37)	2.05(0.37,11.20)	
ASV_239	Firmicutes;D_2__Bacilli;D_3__Lactobacillales;D_4__Leuconostocaceae;D_5__Leuconostoc	0.44(0.09,2.26)	0.40(0.08,2.00)	
ASV_240	Firmicutes;D_2__Clostridia;D_3__Clostridiales;D_4__Christensenellaceae;D_5__Christensenellaceae R-7 group;D_6__uncultured bacterium;D_7__;D_8__;D_9__;D_10__;D_11__;D_12__;D_13__;D_14__	14.67(2.24,95.98);p=0.00508		
ASV_242	Firmicutes;D_2__Bacilli;D_3__Bacillales;D_4__Staphylococcaceae;D_5__Staphylococcus	3.65(0.59,22.39)	5.83(1.02,33.22);p=0.04696	↑
ASV_243	Firmicutes;D_2__Clostridia;D_3__Clostridiales;D_4__Ruminococcaceae;D_5__uncultured	13.59(0.41,451.29)		
ASV_246	Firmicutes;D_2__Clostridia;D_3__Clostridiales;D_4__Lachnospiraceae	0.28(0.05,1.43)		
ASV_249	Firmicutes;D_2__Clostridia;D_3__Clostridiales;D_4__Lachnospiraceae;D_5__Lachnoclostridium	1.70(0.60,4.86)	1.88(0.66,5.34)	
ASV_250	Firmicutes;D_2__Clostridia;D_3__Clostridiales;D_4__Christensenellaceae;D_5__Christensenellaceae R-7 group	12.10(1.67,87.85);p=0.0137		
ASV_252	Firmicutes;D_2__Erysipelotrichia;D_3__Erysipelotrichales;D_4__Erysipelotrichaceae;D_5__Erysipelatoclostridium	0.57(0.11,2.86)		
ASV_253	Firmicutes;D_2__Clostridia;D_3__Clostridiales;D_4__Family XIII;D_5__Mogibacterium	0.69(0.25,1.93)	0.66(0.24,1.83)	
ASV_255	Actinobacteria;D_2__Actinobacteria;D_3__Actinomycetales;D_4__Actinomycetaceae;D_5__Actinomyces	1.23(0.52,2.88)	1.18(0.50,2.77)	
ASV_256	Firmicutes;D_2__Clostridia;D_3__Clostridiales;D_4__Family XIII;D_5__Family XIII AD3011 group;D_6__uncultured bacterium;D_7__;D_8__;D_9__;D_10__;D_11__;D_12__;D_13__;D_14__	1.69(0.32,9.02)	1.58(0.30,8.33)	
ASV_258	Firmicutes;D_2__Clostridia;D_3__Clostridiales;D_4__Defluviitaleaceae;D_5__Defluviitaleaceae UCG-011;D_6__uncultured bacterium;D_7__;D_8__;D_9__;D_10__;D_11__;D_12__;D_13__;D_14__	0.56(0.15,2.07)	0.68(0.20,2.34)	
ASV_259	Patescibacteria;D_2__Saccharimonadia;D_3__Saccharimonadales;D_4__Saccharimonadaceae	2.72(1.00,7.36);p=0.0494	2.73(1.01,7.32);p=0.04674	↑
ASV_260	Actinobacteria;D_2__Coriobacteriia;D_3__Coriobacteriales;D_4__Eggerthellaceae;D_5__Enterorhabdus;D_6__uncultured bacterium;D_7__;D_8__;D_9__;D_10__;D_11__;D_12__;D_13__;D_14__	0.54(0.09,3.06)	0.52(0.09,2.94)	
ASV_262	Firmicutes;D_2__Clostridia;D_3__Clostridiales;D_4__Defluviitaleaceae;D_5__Defluviitaleaceae UCG-011;D_6__uncultured bacterium;D_7__;D_8__;D_9__;D_10__;D_11__;D_12__;D_13__;D_14__	0.07(0.00,1.43)		

ASV_264	Firmicutes;D_2__Clostridia;D_3__Clostridiales;D_4__Ruminococcaceae;D_5__Ruminiclostridium 5;D_6__uncultured bacterium;D_7__;D_8__;D_9__;D_10__;D_11__;D_12__;D_13__;D_14__	0.68(0.19,2.48)	0.79(0.23,2.69)	
ASV_265	Firmicutes;D_2__Clostridia;D_3__Clostridiales;D_4__Lachnospiraceae;D_5__[Ruminococcus] torques group	4.59(0.79,26.62)	3.21(0.59,17.39)	
ASV_268	Proteobacteria;D_2__Gammaproteobacteria;D_3__Betaproteobacteriales;D_4__Burkholderiaceae;D_5__Parasutterella	0.63(0.23,1.74)	0.70(0.25,1.93)	
ASV_270	Bacteroidetes;D_2__Bacteroidia;D_3__Bacteroidales;D_4__Bacteroidaceae;D_5__Bacteroides;D_6__Bacteroides uniformis;D_7__;D_8__;D_9__;D_10__;D_11__;D_12__;D_13__;D_14__	1.25(0.36,4.32)		
ASV_271	Firmicutes;D_2__Clostridia;D_3__Clostridiales;D_4__Ruminococcaceae;D_5__Ruminococcaceae UCG-002		0.50(0.07,3.60)	
ASV_274	Firmicutes;D_2__Clostridia;D_3__Clostridiales;D_4__Christensenellaceae;D_5__Christensenellaceae R-7 group	0.21(0.04,1.14)	0.25(0.05,1.29)	
ASV_275	Firmicutes;D_2__Clostridia;D_3__Clostridiales;D_4__Ruminococcaceae;D_5__Ruminococcaceae UCG-002;D_6__uncultured organism;D_7__;D_8__;D_9__;D_10__;D_11__;D_12__;D_13__;D_14__	0.89(0.34,2.36)	0.89(0.34,2.34)	
ASV_277	Firmicutes;D_2__Clostridia;D_3__Clostridiales;D_4__Ruminococcaceae;D_5__Faecalibacterium;D_6__metagenome;D_7__;D_8__;D_9__;D_10__;D_11__;D_12__;D_13__;D_14__	0.94(0.29,3.03)	0.94(0.29,3.05)	
ASV_278	Bacteroidetes;D_2__Bacteroidia;D_3__Bacteroidales;D_4__Bacteroidaceae;D_5__Bacteroides;D_6__Bacteroides stercoris ATCC 43183;D_7__;D_8__;D_9__;D_10__;D_11__;D_12__;D_13__;D_14__	1.51(0.40,5.71)	1.90(0.50,7.17)	
ASV_282	Firmicutes;D_2__Clostridia;D_3__Clostridiales;D_4__Ruminococcaceae;D_5__Faecalibacterium	0.42(0.15,1.18)	0.45(0.16,1.26)	
ASV_283	Firmicutes;D_2__Clostridia;D_3__Clostridiales;D_4__Ruminococcaceae;D_5__Ruminococcaceae UCG-002	0.74(0.22,2.49)	0.79(0.23,2.66)	
ASV_288	Firmicutes;D_2__Clostridia;D_3__Clostridiales;D_4__Lachnospiraceae;D_5__[Eubacterium] hallii group	1.25(0.72,2.19)	1.26(0.72,2.21)	
ASV_289	Bacteroidetes;D_2__Bacteroidia;D_3__Bacteroidales;D_4__Bacteroidaceae;D_5__Bacteroides	1.19(0.25,5.70)		
ASV_290	Firmicutes;D_2__Clostridia;D_3__Clostridiales;D_4__Ruminococcaceae;D_5__Ruminiclostridium 6	2.06(0.13,32.15)		
ASV_291	Firmicutes;D_2__Clostridia;D_3__Clostridiales;D_4__Lachnospiraceae;D_5__Lachnospira	0.81(0.37,1.78)	0.80(0.37,1.76)	
ASV_292	Firmicutes;D_2__Clostridia;D_3__Clostridiales;D_4__Ruminococcaceae;D_5__Butyricicoccus	0.97(0.48,1.96)	1.07(0.53,2.15)	
ASV_295	Firmicutes;D_2__Clostridia;D_3__Clostridiales;D_4__Lachnospiraceae;D_5__[Eubacterium] xylanophilum group	2.15(0.08,55.08)		
ASV_296	Firmicutes;D_2__Clostridia;D_3__Clostridiales;D_4__Lachnospiraceae	1.03(0.52,2.02)	1.01(0.51,2.00)	
ASV_298	Firmicutes;D_2__Clostridia;D_3__Clostridiales;D_4__Ruminococcaceae;D_5__Oscillibacter;D_6__uncultured organism;D_7__;D_8__;D_9__;D_10__;D_11__;D_12__;D_13__;D_14__	1.23(0.70,2.16)	1.19(0.68,2.08)	
ASV_299	Firmicutes;D_2__Clostridia;D_3__Clostridiales;D_4__Ruminococcaceae;D_5__Ruminococcaceae UCG-005		0.08(0.01,0.46);p=0.00453	↓
ASV_301	Firmicutes;D_2__Clostridia;D_3__Clostridiales;D_4__Lachnospiraceae;D_5__Lachnospiraceae UCG-008;D_6__uncultured organism;D_7__;D_8__;D_9__;D_10__;D_11__;D_12__;D_13__;D_14__	1.06(0.47,2.35)	1.02(0.46,2.27)	
ASV_302	Firmicutes;D_2__Clostridia;D_3__Clostridiales;D_4__Lachnospiraceae;D_5__uncultured	1.25(0.64,2.44)	1.26(0.64,2.46)	
ASV_303	Bacteroidetes;D_2__Bacteroidia;D_3__Bacteroidales;D_4__Marinifilaceae;D_5__Butyricimonas;D_6__uncultured organism;D_7__;D_8__;D_9__;D_10__;D_11__;D_12__;D_13__;D_14__	0.15(0.02,1.35)	0.09(0.01,0.76);p=0.02705	↓
ASV_304	Firmicutes;D_2__Clostridia;D_3__Clostridiales;D_4__Lachnospiraceae;D_5__Lachnospiraceae UCG-010;D_6__uncultured organism;D_7__;D_8__;D_9__;D_10__;D_11__;D_12__;D_13__;D_14__	2.60(0.37,18.42)	1.46(0.22,9.75)	
ASV_306	Firmicutes;D_2__Clostridia;D_3__Clostridiales;D_4__Lachnospiraceae;D_5__Lachnospiraceae UCG-001	1.21(0.30,4.95)	1.04(0.26,4.20)	
ASV_309	Firmicutes;D_2__Clostridia;D_3__Clostridiales;D_4__Lachnospiraceae;D_5__Lachnoclostridium;D_6__uncultured Firmicutes bacterium;D_7__;D_8__;D_9__;D_10__;D_11__;D_12__;D_13__;D_14__	1.04(0.44,2.47)	1.13(0.48,2.65)	
ASV_310	Bacteroidetes;D_2__Bacteroidia;D_3__Bacteroidales;D_4__Marinifilaceae;D_5__Butyricimonas;D_6__uncultured organism;D_7__;D_8__;D_9__;D_10__;D_11__;D_12__;D_13__;D_14__	0.33(0.05,2.26)	0.39(0.06,2.50)	
ASV_313	Firmicutes;D_2__Clostridia;D_3__Clostridiales;D_4__Ruminococcaceae;D_5__Ruminiclostridium 9	0.97(0.37,2.57)	0.87(0.33,2.27)	
ASV_314	Firmicutes;D_2__Clostridia;D_3__Clostridiales;D_4__Lachnospiraceae;D_5__Lachnospiraceae UCG-004	1.71(0.58,5.04)	1.59(0.54,4.72)	
ASV_315	Firmicutes;D_2__Clostridia;D_3__Clostridiales;D_4__Lachnospiraceae;D_5__Lachnospiraceae UCG-004	1.25(0.60,2.59)	1.05(0.51,2.16)	
ASV_317	Firmicutes;D_2__Clostridia;D_3__Clostridiales;D_4__Ruminococcaceae;D_5__[Eubacterium] coprostanoligenes group		0.23(0.03,1.73)	

ASV_318	Firmicutes;D_2__Clostridia;D_3__Clostridiales;D_4__Lachnospiraceae	1.08(0.54,2.14)	1.08(0.54,2.14)	
ASV_322	Firmicutes;D_2__Clostridia;D_3__Clostridiales;D_4__Ruminococcaceae;D_5__Ruminococcaceae UCG-005;D_6__uncultured organism;D_7__;D_8__;D_9__;D_10__;D_11__;D_12__;D_13__;D_14__	0.37(0.10,1.33)	0.41(0.11,1.44)	
ASV_324	Firmicutes;D_2__Clostridia;D_3__Clostridiales;D_4__Ruminococcaceae;D_5__Angelakisella;D_6__uncultured bacterium;D_7__;D_8__;D_9__;D_10__;D_11__;D_12__;D_13__;D_14__	0.85(0.13,5.60)	0.72(0.11,4.72)	
ASV_325	Firmicutes;D_2__Clostridia;D_3__Clostridiales;D_4__Clostridiales vadinBB60 group;D_5__gut metagenome;D_6__;D_7__;D_8__;D_9__;D_10__;D_11__;D_12__;D_13__;D_14__	1.58(0.10,24.88)		
ASV_326	Firmicutes;D_2__Clostridia;D_3__Clostridiales;D_4__Ruminococcaceae;D_5__Ruminococcaceae UCG-014	0.05(0.00,1.14)		
ASV_327	Firmicutes;D_2__Clostridia;D_3__Clostridiales;D_4__Lachnospiraceae;D_5__GCA-900066575	0.83(0.37,1.88)	0.86(0.38,1.95)	
ASV_333	Lentisphaerae;D_2__Lentisphaeria;D_3__Victivallales;D_4__Victivallaceae;D_5__Victivallis;D_6__uncultured bacterium;D_7__;D_8__;D_9__;D_10__;D_11__;D_12__;D_13__;D_14__	2.30(0.12,45.34)	1.99(0.14,28.39)	
ASV_334	Firmicutes;D_2__Clostridia;D_3__Clostridiales;D_4__Lachnospiraceae;D_5__uncultured;D_6__uncultured bacterium adhufec382;D_7__;D_8__;D_9__;D_10__;D_11__;D_12__;D_13__;D_14__	1.19(0.51,2.78)	0.99(0.43,2.27)	
ASV_340	Firmicutes;D_2__Clostridia;D_3__Clostridiales;D_4__Ruminococcaceae;D_5__Flavonifractor;D_6__uncultured bacterium;D_7__;D_8__;D_9__;D_10__;D_11__;D_12__;D_13__;D_14__	0.87(0.22,3.40)	1.14(0.30,4.35)	
ASV_344	Firmicutes;D_2__Clostridia;D_3__Clostridiales;D_4__Defluviitaleaceae;D_5__Defluviitaleaceae UCG-011;D_6__uncultured bacterium;D_7__;D_8__;D_9__;D_10__;D_11__;D_12__;D_13__;D_14__		0.25(0.03,2.34)	
ASV_345	Firmicutes;D_2__Clostridia;D_3__Clostridiales;D_4__Lachnospiraceae;D_5__Lachnospira		0.80(0.15,4.25)	
ASV_348	Firmicutes;D_2__Clostridia;D_3__Clostridiales;D_4__Lachnospiraceae;D_5__Moryella;D_6__human gut metagenome;D_7__;D_8__;D_9__;D_10__;D_11__;D_12__;D_13__;D_14__	0.88(0.38,2.05)	0.77(0.33,1.78)	
ASV_349	Firmicutes;D_2__Clostridia;D_3__Clostridiales;D_4__Ruminococcaceae;D_5__Flavonifractor	1.17(0.65,2.12)	1.13(0.63,2.04)	
ASV_350	Bacteroidetes;D_2__Bacteroidia;D_3__Bacteroidales;D_4__Marinifilaceae;D_5__Butyricimonas;D_6__uncultured organism;D_7__;D_8__;D_9__;D_10__;D_11__;D_12__;D_13__;D_14__	0.33(0.02,6.06)	0.43(0.03,5.93)	
ASV_354	Firmicutes;D_2__Clostridia;D_3__Clostridiales;D_4__Lachnospiraceae;D_5__Lachnospiraceae FCS020 group	0.33(0.11,0.97);p=0.04453	0.30(0.10,0.85);p=0.02342	↓
ASV_355	Firmicutes;D_2__Clostridia;D_3__Clostridiales;D_4__Ruminococcaceae;D_5__Ruminococcaceae UCG-009;D_6__uncultured bacterium;D_7__;D_8__;D_9__;D_10__;D_11__;D_12__;D_13__;D_14__	1.23(0.16,9.68)	0.56(0.08,4.15)	
ASV_357	Firmicutes;D_2__Clostridia;D_3__Clostridiales;D_4__Ruminococcaceae;D_5__Oscillibacter;D_6__human gut metagenome;D_7__;D_8__;D_9__;D_10__;D_11__;D_12__;D_13__;D_14__	0.78(0.42,1.44)	0.69(0.38,1.26)	
ASV_358	Actinobacteria;D_2__Coriobacteriia;D_3__Coriobacteriales;D_4__Atopobiaceae;D_5__uncultured;D_6__uncultured bacterium;D_7__;D_8__;D_9__;D_10__;D_11__;D_12__;D_13__;D_14__	0.34(0.06,2.05)	0.15(0.03,0.85);p=0.03246	↓
ASV_360	Firmicutes;D_2__Clostridia;D_3__Clostridiales;D_4__Ruminococcaceae;D_5__Ruminiclostridium 9;D_6__uncultured organism;D_7__;D_8__;D_9__;D_10__;D_11__;D_12__;D_13__;D_14__	1.06(0.50,2.28)	0.86(0.41,1.79)	
ASV_364	Firmicutes;D_2__Clostridia;D_3__Clostridiales;D_4__Lachnospiraceae;D_5__Roseburia;D_6__gut metagenome;D_7__;D_8__;D_9__;D_10__;D_11__;D_12__;D_13__;D_14__	0.13(0.02,1.05)		
ASV_365	Firmicutes;D_2__Clostridia;D_3__Clostridiales;D_4__Ruminococcaceae;D_5__Intestinimonas	1.60(0.35,7.24)	1.33(0.31,5.78)	
ASV_366	Proteobacteria;D_2__Gammaproteobacteria;D_3__Pasteurellales;D_4__Pasteurellaceae;D_5__Haemophilus	0.86(0.33,2.20)	0.83(0.32,2.14)	
ASV_368	Firmicutes;D_2__Clostridia;D_3__Clostridiales;D_4__Ruminococcaceae;D_5__Ruminococcaceae UCG-013;D_6__gut metagenome;D_7__;D_8__;D_9__;D_10__;D_11__;D_12__;D_13__;D_14__	1.32(0.34,5.14)	1.42(0.37,5.51)	
ASV_369	Firmicutes;D_2__Clostridia;D_3__Clostridiales;D_4__Ruminococcaceae;D_5__Ruminococcaceae UCG-003;D_6__uncultured bacterium;D_7__;D_8__;D_9__;D_10__;D_11__;D_12__;D_13__;D_14__	0.72(0.21,2.51)	0.80(0.23,2.80)	
ASV_370	Firmicutes;D_2__Bacilli;D_3__Lactobacillales;D_4__Lactobacillaceae;D_5__Lactobacillus;D_6__Lactobacillus fermentum;D_7__;D_8__;D_9__;D_10__;D_11__;D_12__;D_13__;D_14__	46.16(4.81,442.82);p=0.00089	153.18(12.03,1949.69);p=0.00011; q=0.02636	↑*
ASV_371	Firmicutes;D_2__Clostridia;D_3__Clostridiales;D_4__Ruminococcaceae;D_5__uncultured;D_6__Clostridium phoceensis;D_7__;D_8__;D_9__;D_10__;D_11__;D_12__;D_13__;D_14__	2.12(1.17,3.86);p=0.01374	1.78(0.98,3.21)	
ASV_373	Firmicutes;D_2__Clostridia;D_3__Clostridiales;D_4__Ruminococcaceae;D_5__Oscillospira;D_6__uncultured bacterium;D_7__;D_8__;D_9__;D_10__;D_11__;D_12__;D_13__;D_14__	0.23(0.03,1.95)		
ASV_374	Firmicutes;D_2__Clostridia;D_3__Clostridiales;D_4__Clostridiaceae 1;D_5__Clostridium sensu stricto 1	1.31(0.42,4.07)	2.26(0.74,6.94)	
ASV_376	Firmicutes;D_2__Clostridia;D_3__Clostridiales;D_4__Clostridiales vadinBB60 group;D_5__gut metagenome;D_6__;D_7__;D_8__;D_9__;D_10__;D_11__;D_12__;D_13__;D_14__	2.10(0.11,38.95)		
ASV_377	Firmicutes;D_2__Clostridia;D_3__Clostridiales;D_4__Ruminococcaceae;D_5__Phocea;D_6__uncultured bacterium;D_7__;D_8__;D_9__;D_10__;D_11__;D_12__;D_13__;D_14__	1.49(0.56,4.00)	1.31(0.49,3.49)	
ASV_378	Proteobacteria;D_2__Gammaproteobacteria;D_3__Enterobacteriales;D_4__Enterobacteriaceae;D_5__Escherichia-Shigella	1.21(0.44,3.34)		

ASV_380	Firmicutes;D_2__Clostridia;D_3__Clostridiales;D_4__Ruminococcaceae;D_5__Oscillibacter;D_6__Oscillibacter sp. PC13;D_7__;D_8__;D_9__;D_10__;D_11__;D_12__;D_13__;D_14__		0.81(0.09,7.62)	
ASV_383	Firmicutes;D_2__Clostridia;D_3__Clostridiales;D_4__Clostridiales vadinBB60 group;D_5__uncultured bacterium;D_6__;D_7__;D_8__;D_9__;D_10__;D_11__;D_12__;D_13__;D_14__	1.51(0.35,6.45)	1.54(0.37,6.39)	
ASV_384	Firmicutes;D_2__Clostridia;D_3__Clostridiales;D_4__Ruminococcaceae;D_5__Ruminococcaceae UCG-005	0.67(0.15,3.07)	0.67(0.15,3.01)	
ASV_387	Firmicutes;D_2__Clostridia;D_3__Clostridiales;D_4__Ruminococcaceae;D_5__Oscillospira;D_6__uncultured organism;D_7__;D_8__;D_9__;D_10__;D_11__;D_12__;D_13__;D_14__	0.44(0.03,6.76)	0.35(0.03,4.81)	
ASV_388	Firmicutes;D_2__Clostridia;D_3__Clostridiales;D_4__Ruminococcaceae;D_5__Oscillospira	0.72(0.27,1.91)	0.61(0.23,1.59)	
ASV_389	Firmicutes;D_2__Clostridia;D_3__Clostridiales	0.89(0.07,12.15)	1.54(0.13,17.78)	
ASV_392	Actinobacteria;D_2__Coriobacteriia;D_3__Coriobacteriales;D_4__Eggerthellaceae;D_5__Eggerthella	1.48(0.71,3.09)	1.53(0.74,3.17)	
ASV_393	Firmicutes;D_2__Clostridia;D_3__Clostridiales;D_4__Ruminococcaceae;D_5__Ruminococcaceae UCG-005;D_6__uncultured organism;D_7__;D_8__;D_9__;D_10__;D_11__;D_12__;D_13__;D_14__	0.80(0.20,3.21)	1.21(0.31,4.82)	
ASV_394	Firmicutes;D_2__Clostridia;D_3__Clostridiales;D_4__Family XIII;D_5__Family XIII AD3011 group;D_6__uncultured bacterium;D_7__;D_8__;D_9__;D_10__;D_11__;D_12__;D_13__;D_14__	1.01(0.19,5.40)	1.03(0.19,5.55)	
ASV_395	Firmicutes;D_2__Clostridia;D_3__Clostridiales;D_4__Lachnospiraceae	0.02(0.00,0.64);p=0.02776		
ASV_396	Firmicutes;D_2__Clostridia;D_3__Clostridiales;D_4__Lachnospiraceae;D_5__Blautia	1.16(0.08,17.33)	0.11(0.01,1.31)	
ASV_397	Firmicutes;D_2__Clostridia;D_3__Clostridiales;D_4__Family XIII;D_5__Family XIII AD3011 group;D_6__uncultured organism;D_7__;D_8__;D_9__;D_10__;D_11__;D_12__;D_13__;D_14__	0.70(0.19,2.57)	0.58(0.16,2.12)	
ASV_399	Firmicutes;D_2__Clostridia;D_3__Clostridiales;D_4__Ruminococcaceae;D_5__Ruminiclostridium;D_6__uncultured bacterium;D_7__;D_8__;D_9__;D_10__;D_11__;D_12__;D_13__;D_14__	0.55(0.03,9.06)	0.28(0.02,4.09)	
ASV_400	Firmicutes;D_2__Clostridia;D_3__Clostridiales;D_4__Christensenellaceae;D_5__Christensenellaceae R-7 group;D_6__uncultured bacterium;D_7__;D_8__;D_9__;D_10__;D_11__;D_12__;D_13__;D_14__	0.28(0.01,9.19)		
ASV_401	Firmicutes;D_2__Clostridia;D_3__Clostridiales;D_4__Ruminococcaceae;D_5__uncultured;D_6__uncultured Ruminococcus sp.;D_7__;D_8__;D_9__;D_10__;D_11__;D_12__;D_13__;D_14__	0.70(0.28,1.76)	0.82(0.33,2.04)	
ASV_405	Actinobacteria;D_2__Actinobacteria;D_3__Actinomycetales;D_4__Actinomycetaceae;D_5__Actinomyces	2.45(0.81,7.41)	2.50(0.83,7.49)	
ASV_407	Firmicutes;D_2__Clostridia;D_3__Clostridiales;D_4__Peptococcaceae;D_5__Peptococcus;D_6__uncultured organism;D_7__;D_8__;D_9__;D_10__;D_11__;D_12__;D_13__;D_14__	0.62(0.05,7.55)		
ASV_410	Firmicutes;D_2__Clostridia;D_3__Clostridiales;D_4__Lachnospiraceae;D_5__Anaerosporobacter;D_6__uncultured organism;D_7__;D_8__;D_9__;D_10__;D_11__;D_12__;D_13__;D_14__	1.14(0.24,5.39)	0.69(0.15,3.21)	
ASV_411	Firmicutes;D_2__Clostridia;D_3__Clostridiales;D_4__Ruminococcaceae;D_5__Ruminiclostridium 9;D_6__uncultured bacterium;D_7__;D_8__;D_9__;D_10__;D_11__;D_12__;D_13__;D_14__	0.50(0.04,6.02)	0.09(0.01,0.97);p=0.04709	↓
ASV_413	Firmicutes;D_2__Clostridia;D_3__Clostridiales;D_4__Lachnospiraceae;D_5__Lachnospiraceae UCG-010;D_6__uncultured bacterium;D_7__;D_8__;D_9__;D_10__;D_11__;D_12__;D_13__;D_14__	1.22(0.40,3.74)		
ASV_414	Firmicutes;D_2__Clostridia;D_3__Clostridiales;D_4__Ruminococcaceae;D_5__GCA-900066225;D_6__uncultured bacterium;D_7__;D_8__;D_9__;D_10__;D_11__;D_12__;D_13__;D_14__	0.28(0.03,2.77)		
ASV_415	Firmicutes;D_2__Clostridia;D_3__Clostridiales;D_4__Ruminococcaceae;D_5__Pseudoflavonifractor;D_6__uncultured bacterium;D_7__;D_8__;D_9__;D_10__;D_11__;D_12__;D_13__;D_14__	0.70(0.13,3.85)	0.37(0.08,1.80)	
ASV_417	Firmicutes;D_2__Clostridia;D_3__Clostridiales;D_4__Christensenellaceae;D_5__Christensenellaceae R-7 group	37.25(0.74,1879.34)		
ASV_418	Firmicutes;D_2__Clostridia;D_3__Clostridiales;D_4__Christensenellaceae;D_5__uncultured;D_6__uncultured bacterium;D_7__;D_8__;D_9__;D_10__;D_11__;D_12__;D_13__;D_14__	0.75(0.07,8.56)	0.75(0.07,8.56)	
ASV_420	Firmicutes;D_2__Clostridia;D_3__Clostridiales;D_4__Ruminococcaceae;D_5__Fournierella;D_6__uncultured organism;D_7__;D_8__;D_9__;D_10__;D_11__;D_12__;D_13__;D_14__	0.24(0.06,0.92);p=0.03733	0.31(0.08,1.17)	
ASV_422	Firmicutes;D_2__Clostridia;D_3__Clostridiales;D_4__Ruminococcaceae;D_5__Ruminococcaceae UCG-007	0.77(0.03,23.25)		
ASV_423	Firmicutes;D_2__Clostridia;D_3__Clostridiales;D_4__Lachnospiraceae;D_5__Lachnoclostridium	1.78(0.63,5.02)	2.39(0.86,6.66)	
ASV_424	Firmicutes;D_2__Clostridia;D_3__Clostridiales;D_4__Lachnospiraceae	0.70(0.12,3.98)	0.55(0.10,3.02)	
ASV_426	Firmicutes;D_2__Clostridia;D_3__Clostridiales;D_4__Lachnospiraceae;D_5__[Ruminococcus] torques group	0.45(0.14,1.45)	0.60(0.19,1.89)	
ASV_428	Firmicutes;D_2__Clostridia;D_3__Clostridiales;D_4__Lachnospiraceae;D_5__Lachnoclostridium	0.66(0.07,5.92)		
ASV_429	Firmicutes;D_2__Clostridia;D_3__Clostridiales;D_4__Ruminococcaceae;D_5__uncultured;D_6__uncultured Clostridium sp.;D_7__;D_8__;D_9__;D_10__;D_11__;D_12__;D_13__;D_14__	1.40(0.30,6.64)	0.84(0.19,3.66)	

ASV_430	Firmicutes;D_2__Clostridia;D_3__Clostridiales;D_4__Ruminococcaceae;D_5__Ruminococcaceae UCG-013;D_6__uncultured Clostridiaceae bacterium;D_7__;D_8__;D_9__;D_10__;D_11__;D_12__;D_13__;D_14__	0.98(0.13,7.70)	1.43(0.20,10.46)	
ASV_431	Firmicutes;D_2__Clostridia;D_3__Clostridiales;D_4__Ruminococcaceae	0.68(0.25,1.86)	0.75(0.28,2.01)	
ASV_432	Firmicutes;D_2__Erysipelotrichia;D_3__Erysipelotrichales;D_4__Erysipelotrichaceae;D_5__Holdemania	0.63(0.18,2.21)	0.60(0.17,2.07)	
ASV_434	Firmicutes;D_2__Clostridia;D_3__Clostridiales;D_4__Ruminococcaceae	0.45(0.04,4.95)	0.74(0.07,7.81)	
ASV_437	Firmicutes;D_2__Clostridia;D_3__Clostridiales;D_4__Ruminococcaceae;D_5__Candidatus Soleaferrea;D_6__uncultured bacterium;D_7__;D_8__;D_9__;D_10__;D_11__;D_12__;D_13__;D_14__	0.43(0.10,1.86)	0.37(0.09,1.62)	
ASV_438	Proteobacteria;D_2__Gammaproteobacteria;D_3__Betaproteobacteriales;D_4__Burkholderiaceae;D_5__Noviherbaspirillum	0.61(0.09,4.00)	0.57(0.09,3.81)	
ASV_440	Firmicutes;D_2__Clostridia;D_3__Clostridiales;D_4__Ruminococcaceae;D_5__Ruminococcaceae UCG-010;D_6__uncultured bacterium;D_7__;D_8__;D_9__;D_10__;D_11__;D_12__;D_13__;D_14__	0.40(0.04,4.37)	0.86(0.08,9.62)	
ASV_441	Firmicutes;D_2__Clostridia;D_3__Clostridiales;D_4__Lachnospiraceae;D_5__Blautia	2.06(0.08,55.46)		
ASV_442	Firmicutes;D_2__Clostridia;D_3__Clostridiales;D_4__Ruminococcaceae;D_5__uncultured	0.28(0.04,1.95)	0.18(0.02,1.35)	
ASV_445	Proteobacteria;D_2__Gammaproteobacteria;D_3__Betaproteobacteriales;D_4__Burkholderiaceae;D_5__Sutterella	2.50(0.45,13.77)	1.47(0.27,7.99)	
ASV_448	Actinobacteria;D_2__Actinobacteria;D_3__Bifidobacteriales;D_4__Bifidobacteriaceae;D_5__Bifidobacterium	0.63(0.19,2.10)	0.69(0.21,2.30)	
ASV_453	Firmicutes;D_2__Clostridia;D_3__Clostridiales;D_4__Lachnospiraceae;D_5__Anaerostipes	0.60(0.18,1.97)		
ASV_456	Firmicutes;D_2__Clostridia;D_3__Clostridiales;D_4__Peptostreptococcaceae;D_5__Intestinibacter	0.59(0.32,1.09)	0.78(0.44,1.39)	
ASV_457	Firmicutes;D_2__Clostridia;D_3__Clostridiales;D_4__Lachnospiraceae;D_5__uncultured	4.38(0.67,28.42)	4.64(0.75,28.81)	
ASV_460	Firmicutes;D_2__Clostridia;D_3__Clostridiales;D_4__Lachnospiraceae	1.78(0.58,5.51)	1.83(0.60,5.60)	
ASV_462	Firmicutes;D_2__Clostridia;D_3__Clostridiales;D_4__Lachnospiraceae;D_5__uncultured;D_6__uncultured bacterium adhufec382;D_7__;D_8__;D_9__;D_10__;D_11__;D_12__;D_13__;D_14__	1.41(0.35,5.70)	1.20(0.30,4.79)	
ASV_465	Firmicutes;D_2__Bacilli;D_3__Lactobacillales;D_4__Lactobacillaceae;D_5__Lactobacillus	2.37(0.30,18.91)	3.34(0.43,26.04)	
ASV_466	Firmicutes;D_2__Clostridia;D_3__Clostridiales;D_4__Lachnospiraceae;D_5__uncultured	14.79(1.04,209.71);p=0.04652	15.81(1.09,229.46);p=0.04315	↑
ASV_467	Firmicutes;D_2__Bacilli;D_3__Lactobacillales;D_4__Lactobacillaceae;D_5__Lactobacillus	0.26(0.06,1.23)	0.23(0.05,1.07)	
ASV_468	Firmicutes;D_2__Clostridia;D_3__Clostridiales;D_4__Lachnospiraceae;D_5__uncultured;D_6__metagenome;D_7__;D_8__;D_9__;D_10__;D_11__;D_12__;D_13__;D_14__	2.44(0.29,20.53)	1.70(0.20,14.62)	
ASV_471	Firmicutes;D_2__Bacilli;D_3__Lactobacillales;D_4__Streptococcaceae;D_5__Streptococcus	1.88(0.65,5.49)	2.02(0.70,5.86)	
ASV_472	Firmicutes;D_2__Erysipelotrichia;D_3__Erysipelotrichales;D_4__Erysipelotrichaceae;D_5__Dielma	2.21(0.77,6.34)	2.14(0.76,6.03)	
ASV_474	Firmicutes;D_2__Bacilli;D_3__Lactobacillales;D_4__Leuconostocaceae;D_5__Leuconostoc	3.52(0.51,24.23)	4.05(0.61,27.03)	
ASV_475	Firmicutes;D_2__Negativicutes;D_3__Selenomonadales;D_4__Veillonellaceae;D_5__Veillonella	1.17(0.20,7.05)	1.09(0.18,6.44)	
ASV_476	Cyanobacteria;D_2__Oxyphotobacteria;D_3__Chloroplast	8.19(0.55,122.79)	24.14(0.98,593.54)	
ASV_477	Firmicutes;D_2__Clostridia;D_3__Clostridiales;D_4__Peptostreptococcaceae;D_5__Terrisporobacter;D_6__uncultured bacterium;D_7__;D_8__;D_9__;D_10__;D_11__;D_12__;D_13__;D_14__	0.64(0.24,1.68)	1.05(0.41,2.67)	
ASV_481	Proteobacteria;D_2__Alphaproteobacteria;D_3__Caulobacterales;D_4__Caulobacteraceae;D_5__Brevundimonas	0.44(0.07,2.72)	0.52(0.08,3.19)	
ASV_483	Firmicutes;D_2__Bacilli;D_3__Lactobacillales;D_4__Streptococcaceae;D_5__Lactococcus	1.60(0.09,29.08)		
ASV_485	Actinobacteria;D_2__Actinobacteria;D_3__Micrococcales;D_4__Micrococcaceae;D_5__Rothia;D_6__uncultured organism;D_7__;D_8__;D_9__;D_10__;D_11__;D_12__;D_13__;D_14__	7.13(1.02,50.00);p=0.04811	8.43(1.13,62.78);p=0.03739	↑
ASV_488	Proteobacteria;D_2__Gammaproteobacteria;D_3__Pasteurellales;D_4__Pasteurellaceae	0.38(0.06,2.17)	0.56(0.11,2.93)	
ASV_489	Actinobacteria;D_2__Actinobacteria;D_3__Actinomycetales;D_4__Actinomycetaceae;D_5__Actinomyces;D_6__Actinomyces graevenitzii F0530;D_7__;D_8__;D_9__;D_10__;D_11__;D_12__;D_13__;D_14__	1.08(0.42,2.78)	1.10(0.43,2.86)	

ASV_490	Firmicutes;D_2__Clostridia;D_3__Clostridiales;D_4__Lachnospiraceae;D_5__[Ruminococcus] gnavus group	1.49(0.56,3.98)	1.64(0.62,4.36)	
ASV_491	Proteobacteria;D_2__Alphaproteobacteria;D_3__Sphingomonadales;D_4__Sphingomonadaceae;D_5__Sphingomonas	0.36(0.02,6.07)	0.37(0.02,5.99)	
ASV_492	Firmicutes;D_2__Bacilli;D_3__Lactobacillales;D_4__Streptococcaceae;D_5__Lactococcus;D_6__Lactococcus garvieae subsp. garvieae;D_7__;D_8__;D_9__;D_10__;D_11__;D_12__;D_13__;D_14__	2.17(0.06,83.83)		
ASV_493	Actinobacteria;D_2__Actinobacteria;D_3__Micrococcales;D_4__Micrococcaceae;D_5__Rothia;D_6__uncultured bacterium;D_7__;D_8__;D_9__;D_10__;D_11__;D_12__;D_13__;D_14__	0.87(0.08,9.68)	0.89(0.08,10.39)	
ASV_496	Firmicutes;D_2__Clostridia;D_3__Clostridiales;D_4__Peptostreptococcaceae;D_5__Terrisporobacter;D_6__uncultured bacterium;D_7__;D_8__;D_9__;D_10__;D_11__;D_12__;D_13__;D_14__	0.60(0.02,19.61)		
ASV_502	Bacteroidetes;D_2__Bacteroidia;D_3__Bacteroidales;D_4__Bacteroidaceae;D_5__Bacteroides;D_6__Bacteroides fragilis;D_7__;D_8__;D_9__;D_10__;D_11__;D_12__;D_13__;D_14__	0.31(0.06,1.74)	0.36(0.07,2.02)	
ASV_503	Proteobacteria;D_2__Gammaproteobacteria;D_3__Enterobacteriales;D_4__Enterobacteriaceae	0.89(0.14,5.63)	0.91(0.15,5.68)	
ASV_504	Firmicutes;D_2__Erysipelotrichia;D_3__Erysipelotrichales;D_4__Erysipelotrichaceae;D_5__Turicibacter	1.30(0.61,2.78)	1.42(0.66,3.04)	
ASV_506	Firmicutes;D_2__Clostridia;D_3__Clostridiales;D_4__Lachnospiraceae	0.34(0.06,1.99)	0.18(0.03,1.04)	
ASV_508	Firmicutes;D_2__Clostridia;D_3__Clostridiales;D_4__Ruminococcaceae;D_5__Ruminococcaceae UCG-005	0.43(0.13,1.47)	0.45(0.13,1.49)	
ASV_510	Firmicutes;D_2__Clostridia;D_3__Clostridiales;D_4__Lachnospiraceae;D_5__Blautia	0.10(0.01,1.07)	0.03(0.00,0.25);p=0.00154	↓
ASV_512	Firmicutes;D_2__Clostridia;D_3__Clostridiales;D_4__Lachnospiraceae;D_5__Lachnoclostridium	0.99(0.16,6.03)	1.44(0.25,8.36)	
ASV_513	Bacteroidetes;D_2__Bacteroidia;D_3__Bacteroidales;D_4__Marinifilaceae;D_5__Butyricimonas;D_6__uncultured bacterium;D_7__;D_8__;D_9__;D_10__;D_11__;D_12__;D_13__;D_14__	0.26(0.01,5.26)		
ASV_514	Firmicutes;D_2__Clostridia;D_3__Clostridiales;D_4__Ruminococcaceae;D_5__Ruminococcaceae NK4A214 group;D_6__uncultured bacterium;D_7__;D_8__;D_9__;D_10__;D_11__;D_12__;D_13__;D_14__	0.08(0.00,1.28)		
ASV_516	Firmicutes;D_2__Clostridia;D_3__Clostridiales;D_4__Ruminococcaceae;D_5__Negativibacillus;D_6__uncultured bacterium;D_7__;D_8__;D_9__;D_10__;D_11__;D_12__;D_13__;D_14__	1.62(0.20,12.96)	0.33(0.05,2.41)	
ASV_518	Firmicutes;D_2__Clostridia;D_3__Clostridiales;D_4__Peptostreptococcaceae;D_5__Romboutsia	3.58(0.38,33.84)	3.08(0.33,28.79)	
ASV_522	Firmicutes;D_2__Clostridia;D_3__Clostridiales;D_4__Family XIII;D_5__Family XIII UCG-001;D_6__uncultured bacterium;D_7__;D_8__;D_9__;D_10__;D_11__;D_12__;D_13__;D_14__	0.62(0.23,1.68)		
ASV_524	Firmicutes;D_2__Clostridia;D_3__Clostridiales;D_4__Ruminococcaceae;D_5__Butyricicoccus	1.38(0.41,4.59)		
ASV_526	Firmicutes;D_2__Clostridia;D_3__Clostridiales;D_4__Lachnospiraceae;D_5__uncultured;D_6__metagenome;D_7__;D_8__;D_9__;D_10__;D_11__;D_12__;D_13__;D_14__	2.60(0.87,7.83)	2.29(0.77,6.80)	
ASV_527	Bacteroidetes;D_2__Bacteroidia;D_3__Bacteroidales;D_4__Bacteroidaceae;D_5__Bacteroides;D_6__Bacteroides sp. HPS0048;D_7__;D_8__;D_9__;D_10__;D_11__;D_12__;D_13__;D_14__	0.91(0.07,11.98)	0.87(0.07,10.10)	
ASV_529	Firmicutes;D_2__Clostridia;D_3__Clostridiales;D_4__Ruminococcaceae;D_5__Ruminiclostridium 9	2.32(0.56,9.60)	2.35(0.56,9.79)	
ASV_531	Firmicutes;D_2__Clostridia;D_3__Clostridiales;D_4__Lachnospiraceae;D_5__Lachnoclostridium	1.13(0.10,13.19)	1.25(0.11,14.65)	
ASV_532	Firmicutes;D_2__Bacilli;D_3__Lactobacillales;D_4__Leuconostocaceae;D_5__Weissella;D_6__Weissella viridescens;D_7__;D_8__;D_9__;D_10__;D_11__;D_12__;D_13__;D_14__	0.08(0.00,1.23)	0.07(0.00,1.13)	
ASV_533	Firmicutes;D_2__Clostridia;D_3__Clostridiales;D_4__Lachnospiraceae;D_5__Marvinbryantia	0.72(0.24,2.16)		
ASV_535	Firmicutes;D_2__Clostridia;D_3__Clostridiales;D_4__Christensenellaceae;D_5__uncultured;D_6__uncultured bacterium;D_7__;D_8__;D_9__;D_10__;D_11__;D_12__;D_13__;D_14__	0.23(0.02,2.69)		
ASV_536	Firmicutes;D_2__Clostridia;D_3__Clostridiales;D_4__Clostridiaceae 1;D_5__Clostridium sensu stricto 1	1.55(0.69,3.51)	1.60(0.71,3.62)	
ASV_538	Firmicutes;D_2__Clostridia;D_3__Clostridiales;D_4__Ruminococcaceae;D_5__Ruminiclostridium 5;D_6__uncultured organism;D_7__;D_8__;D_9__;D_10__;D_11__;D_12__;D_13__;D_14__	20.18(2.48,164.25);p=0.00497		
ASV_539	Firmicutes;D_2__Clostridia;D_3__Clostridiales;D_4__Lachnospiraceae;D_5__[Eubacterium] ventriosum group	1.30(0.23,7.25)	1.38(0.25,7.64)	
ASV_545	Firmicutes;D_2__Clostridia;D_3__Clostridiales;D_4__Lachnospiraceae;D_5__[Eubacterium] ventriosum group;D_6__uncultured bacterium;D_7__;D_8__;D_9__;D_10__;D_11__;D_12__;D_13__;D_14__	0.06(0.01,0.60);p=0.01665		
ASV_547	Firmicutes;D_2__Clostridia;D_3__Clostridiales;D_4__Ruminococcaceae;D_5__Oscillibacter;D_6__Oscillibacter sp. Marseille-P3260;D_7__;D_8__;D_9__;D_10__;D_11__;D_12__;D_13__;D_14__	0.41(0.08,2.16)	0.41(0.08,2.17)	
ASV_549	Firmicutes;D_2__Clostridia;D_3__Clostridiales;D_4__Lachnospiraceae	1.11(0.43,2.84)	1.36(0.54,3.47)	

ASV_551	Firmicutes;D_2__Clostridia;D_3__Clostridiales;D_4__Lachnospiraceae;D_5__[Eubacterium] xylanophilum group	1.12(0.36,3.51)	1.20(0.38,3.76)	
ASV_552	Firmicutes;D_2__Clostridia;D_3__Clostridiales;D_4__Christensenellaceae;D_5__Christensenellaceae R-7 group	0.41(0.03,4.78)	0.39(0.03,4.45)	
ASV_556	Firmicutes;D_2__Bacilli;D_3__Lactobacillales;D_4__Leuconostocaceae;D_5__Weissella;D_6__Weissella paramesenteroides;D_7__;D_8__;D_9__;D_10__;D_11__;D_12__;D_13__;D_14__	1.64(0.04,72.66)		
ASV_557	Firmicutes;D_2__Clostridia;D_3__Clostridiales;D_4__Christensenellaceae;D_5__uncultured;D_6__uncultured bacterium;D_7__;D_8__;D_9__;D_10__;D_11__;D_12__;D_13__;D_14__	1.71(0.64,4.56)	1.21(0.46,3.15)	
ASV_559	Firmicutes;D_2__Clostridia;D_3__Clostridiales;D_4__Ruminococcaceae;D_5__Anaerofilum;D_6__uncultured bacterium;D_7__;D_8__;D_9__;D_10__;D_11__;D_12__;D_13__;D_14__	0.27(0.01,7.19)		
ASV_560	Firmicutes;D_2__Clostridia;D_3__Clostridiales;D_4__Lachnospiraceae;D_5__Anaerostipes;D_6__unidentified;D_7__;D_8__;D_9__;D_10__;D_11__;D_12__;D_13__;D_14__	0.27(0.06,1.32)	0.29(0.06,1.41)	
ASV_561	Actinobacteria;D_2__Coriobacteriia;D_3__Coriobacteriales;D_4__Coriobacteriales Incertae Sedis;D_5__Raoultibacter;D_6__Raoultibacter timonensis;D_7__;D_8__;D_9__;D_10__;D_11__;D_12__;D_13__;D_14__	0.60(0.06,6.05)	0.48(0.05,4.87)	
ASV_565	Firmicutes;D_2__Clostridia;D_3__Clostridiales;D_4__Ruminococcaceae;D_5__Oscillibacter;D_6__uncultured bacterium;D_7__;D_8__;D_9__;D_10__;D_11__;D_12__;D_13__;D_14__		2.42(0.39,15.15)	
ASV_566	Firmicutes;D_2__Clostridia;D_3__Clostridiales;D_4__Ruminococcaceae	0.22(0.04,1.27)	0.52(0.10,2.79)	
ASV_568	Firmicutes;D_2__Clostridia;D_3__Clostridiales;D_4__Clostridiales vadinBB60 group;D_5__gut metagenome;D_6__;D_7__;D_8__;D_9__;D_10__;D_11__;D_12__;D_13__;D_14__	0.78(0.10,5.91)	0.85(0.12,6.29)	
ASV_571	Firmicutes;D_2__Clostridia;D_3__Clostridiales;D_4__Lachnospiraceae	1.83(0.16,21.29)		
ASV_572	Firmicutes;D_2__Clostridia;D_3__Clostridiales;D_4__Christensenellaceae;D_5__Christensenellaceae R-7 group;D_6__uncultured marine bacterium;D_7__;D_8__;D_9__;D_10__;D_11__;D_12__;D_13__;D_14__	1.70(0.75,3.85)	1.77(0.81,3.86)	
ASV_573	Firmicutes;D_2__Bacilli;D_3__Lactobacillales;D_4__Streptococcaceae;D_5__Streptococcus;D_6__Streptococcus mutans;D_7__;D_8__;D_9__;D_10__;D_11__;D_12__;D_13__;D_14__	4.34(1.60,11.76);p=0.00395	3.66(1.40,9.60);p=0.00837	↑
ASV_576	Actinobacteria;D_2__Actinobacteria;D_3__Actinomycetales;D_4__Actinomycetaceae;D_5__Actinomyces	0.75(0.04,15.35)	0.66(0.03,14.31)	
ASV_577	Firmicutes;D_2__Clostridia;D_3__Clostridiales;D_4__Eubacteriaceae;D_5__Eubacterium;D_6__Eubacterium limosum;D_7__;D_8__;D_9__;D_10__;D_11__;D_12__;D_13__;D_14__	2.13(0.42,10.71)	1.68(0.34,8.36)	
ASV_580	Firmicutes;D_2__Clostridia;D_3__Clostridiales;D_4__Ruminococcaceae;D_5__Anaerotruncus;D_6__uncultured bacterium;D_7__;D_8__;D_9__;D_10__;D_11__;D_12__;D_13__;D_14__	0.21(0.01,4.19)		
ASV_581	Firmicutes;D_2__Erysipelotrichia;D_3__Erysipelotrichales;D_4__Erysipelotrichaceae;D_5__Solobacterium	0.08(0.01,0.46);p=0.00457	0.37(0.08,1.66)	
ASV_582	Firmicutes;D_2__Clostridia;D_3__Clostridiales;D_4__Family XIII;D_5__Family XIII AD3011 group;D_6__gut metagenome;D_7__;D_8__;D_9__;D_10__;D_11__;D_12__;D_13__;D_14__	0.63(0.04,10.37)	0.79(0.05,11.62)	
ASV_583	Firmicutes;D_2__Bacilli;D_3__Lactobacillales;D_4__Lactobacillaceae;D_5__Lactobacillus;D_6__Lactobacillus helveticus;D_7__;D_8__;D_9__;D_10__;D_11__;D_12__;D_13__;D_14__		0.00(0.00,Inf)	
ASV_585	Firmicutes;D_2__Clostridia;D_3__Clostridiales;D_4__Ruminococcaceae;D_5__Ruminiclostridium 5;D_6__uncultured bacterium;D_7__;D_8__;D_9__;D_10__;D_11__;D_12__;D_13__;D_14__	0.85(0.21,3.45)		
ASV_586	Firmicutes;D_2__Clostridia;D_3__Clostridiales;D_4__Ruminococcaceae;D_5__Anaerotruncus;D_6__uncultured organism;D_7__;D_8__;D_9__;D_10__;D_11__;D_12__;D_13__;D_14__	0.53(0.22,1.31)	0.56(0.23,1.39)	
ASV_588	Firmicutes;D_2__Erysipelotrichia;D_3__Erysipelotrichales;D_4__Erysipelotrichaceae;D_5__uncultured;D_6__Clostridiales bacterium 60-7e;D_7__;D_8__;D_9__;D_10__;D_11__;D_12__;D_13__;D_14__	3.20(0.45,22.93)	3.54(0.51,24.46)	
ASV_589	Proteobacteria;D_2__Gammaproteobacteria;D_3__Betaproteobacteriales;D_4__Burkholderiaceae;D_5__Variovorax	0.90(0.28,2.91)	0.90(0.28,2.89)	
ASV_590	Firmicutes;D_2__Clostridia;D_3__Clostridiales;D_4__Lachnospiraceae	0.43(0.09,2.08)	0.35(0.07,1.68)	
ASV_591	Firmicutes;D_2__Clostridia;D_3__Clostridiales;D_4__Clostridiales vadinBB60 group;D_5__gut metagenome;D_6__;D_7__;D_8__;D_9__;D_10__;D_11__;D_12__;D_13__;D_14__	0.50(0.09,2.81)		
ASV_594	Firmicutes;D_2__Clostridia;D_3__Clostridiales;D_4__Ruminococcaceae;D_5__Candidatus Soleaferrea;D_6__Bittarella massiliensis;D_7__;D_8__;D_9__;D_10__;D_11__;D_12__;D_13__;D_14__	0.87(0.45,1.66)	0.84(0.44,1.62)	
ASV_595	Firmicutes;D_2__Clostridia;D_3__Clostridiales;D_4__Lachnospiraceae	4.70(0.66,33.78)	6.53(0.91,46.62)	
ASV_596	Firmicutes;D_2__Clostridia;D_3__Clostridiales;D_4__Lachnospiraceae;D_5__[Eubacterium] fissicatena group	0.65(0.28,1.50)	0.67(0.29,1.55)	
ASV_597	Firmicutes;D_2__Clostridia;D_3__Clostridiales;D_4__Lachnospiraceae;D_5__Lachnoclostridium;D_6__[Clostridium] scindens;D_7__;D_8__;D_9__;D_10__;D_11__;D_12__;D_13__;D_14__	1.34(0.37,4.94)	1.26(0.35,4.60)	
ASV_598	Firmicutes;D_2__Clostridia;D_3__Clostridiales;D_4__Lachnospiraceae;D_5__[Ruminococcus] torques group;D_6__uncultured Clostridiales bacterium;D_7__;D_8__;D_9__;D_10__;D_11__;D_12__;D_13__;D_14__	1.69(0.44,6.51)	1.37(0.36,5.25)	
ASV_599	Firmicutes;D_2__Clostridia;D_3__Clostridiales;D_4__Ruminococcaceae;D_5__Ruminiclostridium 9	1.17(0.51,2.72)	1.16(0.50,2.70)	

ASV_600	Firmicutes;D_2__Clostridia;D_3__Clostridiales;D_4__Lachnospiraceae;D_5__UC5-1-2E3;D_6__uncultured bacterium;D_7__;D_8__;D_9__;D_10__;D_11__;D_12__;D_13__;D_14__		1.19(0.17,8.16)	
ASV_601	Firmicutes;D_2__Clostridia;D_3__Clostridiales;D_4__Lachnospiraceae		1.15(0.36,3.64)	
ASV_604	Proteobacteria;D_2__Gammaproteobacteria;D_3__Betaproteobacteriales;D_4__Burkholderiaceae;D_5__Sutterella;D_6__gut metagenome;D_7__;D_8__;D_9__;D_10__;D_11__;D_12__;D_13__;D_14__	28.50(1.40,578.69);p=0.02922	27.66(1.35,566.11);p=0.03114	↑
ASV_611	Firmicutes;D_2__Clostridia;D_3__Clostridiales;D_4__Lachnospiraceae;D_5__uncultured	0.93(0.17,4.97)	0.84(0.16,4.50)	
ASV_613	Firmicutes;D_2__Erysipelotrichia;D_3__Erysipelotrichales;D_4__Erysipelotrichaceae;D_5__Turicibacter	1.45(0.33,6.46)	1.94(0.48,7.94)	
ASV_614	Firmicutes;D_2__Clostridia;D_3__Clostridiales;D_4__Ruminococcaceae;D_5__Ruminococcaceae UCG-005;D_6__metagenome;D_7__;D_8__;D_9__;D_10__;D_11__;D_12__;D_13__;D_14__	36.17(1.34,975.69);p=0.03281		
ASV_620	Firmicutes;D_2__Clostridia;D_3__Clostridiales;D_4__Lachnospiraceae;D_5__uncultured;D_6__uncultured organism;D_7__;D_8__;D_9__;D_10__;D_11__;D_12__;D_13__;D_14__	0.23(0.03,1.48)	0.04(0.01,0.24);p=0.00046	↓
ASV_621	Firmicutes;D_2__Clostridia;D_3__Clostridiales;D_4__Ruminococcaceae;D_5__Intestinimonas;D_6__Intestinimonas butyriciproducens;D_7__;D_8__;D_9__;D_10__;D_11__;D_12__;D_13__;D_14__	1.09(0.32,3.77)		
ASV_622	Firmicutes;D_2__Clostridia;D_3__Clostridiales;D_4__Lachnospiraceae;D_5__Blautia	9.81(0.78,122.68)	11.27(0.90,141.89)	
ASV_623	Firmicutes;D_2__Clostridia;D_3__Clostridiales;D_4__Lachnospiraceae;D_5__[Ruminococcus] torques group;D_6__uncultured bacterium;D_7__;D_8__;D_9__;D_10__;D_11__;D_12__;D_13__;D_14__	1.25(0.33,4.73)	1.34(0.35,5.10)	
ASV_628	Firmicutes;D_2__Clostridia;D_3__Clostridiales;D_4__Ruminococcaceae;D_5__Butyricicoccus	2.16(0.93,5.04)	2.29(0.99,5.29)	
ASV_630	Firmicutes;D_2__Clostridia;D_3__Clostridiales;D_4__Clostridiaceae 1;D_5__Clostridium sensu stricto 1	7.06(1.08,45.95);p=0.04097	12.68(2.00,80.19);p=0.00695	↑
ASV_631	Firmicutes;D_2__Clostridia;D_3__Clostridiales;D_4__Lachnospiraceae	2.35(0.16,35.30)	8.09(0.47,139.99)	
ASV_632	Firmicutes;D_2__Clostridia;D_3__Clostridiales;D_4__Lachnospiraceae;D_5__Roseburia		0.18(0.01,2.57)	
ASV_634	Firmicutes;D_2__Clostridia;D_3__Clostridiales;D_4__Ruminococcaceae;D_5__Ruminiclostridium 5	0.14(0.02,0.94);p=0.04272	0.11(0.02,0.64);p=0.01457	↓
ASV_636	Firmicutes;D_2__Clostridia;D_3__Clostridiales;D_4__Lachnospiraceae;D_5__Blautia;D_6__Ruminococcus sp. Marseille-P328;D_7__;D_8__;D_9__;D_10__;D_11__;D_12__;D_13__;D_14__	4.76(0.30,75.73)	3.65(0.25,54.11)	
ASV_637	Firmicutes;D_2__Clostridia;D_3__Clostridiales;D_4__Ruminococcaceae;D_5__Ruminococcaceae UCG-005	3.73(0.90,15.55)	5.35(1.27,22.56);p=0.02231	↑
ASV_638	Bacteroidetes;D_2__Bacteroidia;D_3__Bacteroidales;D_4__Prevotellaceae;D_5__Prevotella 9	6.55(0.25,170.22)		
ASV_640	Firmicutes;D_2__Clostridia;D_3__Clostridiales;D_4__Ruminococcaceae;D_5__[Eubacterium] coprostanoligenes group	1.75(0.09,32.26)	2.51(0.12,51.42)	
ASV_641	Actinobacteria;D_2__Coriobacteriia;D_3__Coriobacteriales;D_4__Coriobacteriaceae;D_5__Collinsella;D_6__uncultured bacterium;D_7__;D_8__;D_9__;D_10__;D_11__;D_12__;D_13__;D_14__	1.92(0.17,21.30)	3.71(0.34,40.38)	
ASV_642	Firmicutes;D_2__Clostridia;D_3__Clostridiales;D_4__Lachnospiraceae	0.99(0.21,4.54)	1.00(0.22,4.63)	
ASV_643	Firmicutes;D_2__Clostridia;D_3__Clostridiales;D_4__Ruminococcaceae;D_5__Candidatus Soleaferrea;D_6__uncultured Anaerotruncus sp.;D_7__;D_8__;D_9__;D_10__;D_11__;D_12__;D_13__;D_14__	1.25(0.39,4.02)	1.27(0.40,4.04)	
ASV_644	Firmicutes;D_2__Clostridia;D_3__Clostridiales;D_4__Lachnospiraceae;D_5__Lachnospiraceae UCG-008;D_6__uncultured organism;D_7__;D_8__;D_9__;D_10__;D_11__;D_12__;D_13__;D_14__	3.19(0.68,15.00)	2.40(0.56,10.35)	
ASV_646	Firmicutes;D_2__Clostridia;D_3__Clostridiales;D_4__Ruminococcaceae	0.75(0.16,3.62)		
ASV_647	Firmicutes;D_2__Clostridia;D_3__Clostridiales;D_4__Ruminococcaceae;D_5__Acetanaerobacterium;D_6__uncultured rumen bacterium;D_7__;D_8__;D_9__;D_10__;D_11__;D_12__;D_13__;D_14__	0.61(0.23,1.65)	0.60(0.22,1.62)	
ASV_648	Firmicutes;D_2__Clostridia;D_3__Clostridiales;D_4__Lachnospiraceae;D_5__Lachnospiraceae UCG-004;D_6__uncultured organism;D_7__;D_8__;D_9__;D_10__;D_11__;D_12__;D_13__;D_14__	1.92(0.12,30.04)	1.41(0.09,21.62)	
ASV_649	Firmicutes;D_2__Clostridia;D_3__Clostridiales;D_4__Clostridiaceae 1;D_5__Clostridium sensu stricto 1	34.79(0.94,1285.63)		
ASV_650	Firmicutes;D_2__Clostridia;D_3__Clostridiales;D_4__Christensenellaceae;D_5__Catabacter	0.95(0.21,4.42)		
ASV_651	Actinobacteria;D_2__Actinobacteria;D_3__Micrococcales;D_4__Micrococcaceae;D_5__Rothia;D_6__uncultured organism;D_7__;D_8__;D_9__;D_10__;D_11__;D_12__;D_13__;D_14__	0.34(0.03,4.19)		
ASV_652	Proteobacteria;D_2__Gammaproteobacteria;D_3__Betaproteobacteriales;D_4__Burkholderiaceae;D_5__Noviherbaspirillum	1.96(0.36,10.64)	1.24(0.25,6.28)	
ASV_653	Firmicutes;D_2__Clostridia;D_3__Clostridiales;D_4__Ruminococcaceae;D_5__GCA-900066225;D_6__Massilimaliae massiliensis;D_7__;D_8__;D_9__;D_10__;D_11__;D_12__;D_13__;D_14__	0.87(0.02,41.09)		

ASV_654	Proteobacteria;D_2__Gammaproteobacteria;D_3__Betaproteobacteriales;D_4__Burkholderiaceae;D_5__Acidovorax	2.67(0.36,20.00)	2.77(0.37,20.95)	
ASV_656	Firmicutes;D_2__Clostridia;D_3__Clostridiales;D_4__Family XIII;D_5__Family XIII AD3011 group;D_6__uncultured bacterium;D_7__;D_8__;D_9__;D_10__;D_11__;D_12__;D_13__;D_14__	2.59(0.45,14.84)	5.01(0.94,26.64)	
ASV_658	Firmicutes;D_2__Clostridia;D_3__Clostridiales;D_4__Ruminococcaceae;D_5__Flavonifractor;D_6__uncultured bacterium;D_7__;D_8__;D_9__;D_10__;D_11__;D_12__;D_13__;D_14__	0.75(0.27,2.06)	0.72(0.27,1.97)	
ASV_659	Bacteroidetes;D_2__Bacteroidia;D_3__Bacteroidales;D_4__Tannerellaceae;D_5__Parabacteroides	7607540261.62(0.00,Inf)		
ASV_668	Firmicutes;D_2__Bacilli;D_3__Lactobacillales;D_4__Lactobacillaceae;D_5__Lactobacillus	18.15(1.88,175.32);p=0.01223		
ASV_672	Firmicutes;D_2__Bacilli;D_3__Lactobacillales;D_4__Lactobacillaceae;D_5__Lactobacillus	180.88(23.19,1410.89);p=0	254.94(27.17,2392.18);p=0;q=0.00061	↑*
ASV_676	Firmicutes;D_2__Bacilli;D_3__Lactobacillales;D_4__Lactobacillaceae;D_5__Lactobacillus	1.26(0.20,7.90)	1.14(0.19,6.85)	
ASV_678	Firmicutes;D_2__Erysipelotrichia;D_3__Erysipelotrichales;D_4__Erysipelotrichaceae;D_5__Erysipelatoclostridium	1.15(0.43,3.07)	1.10(0.41,2.94)	
ASV_679	Firmicutes;D_2__Clostridia;D_3__Clostridiales;D_4__Peptostreptococcaceae;D_5__Paeniclostridium;D_6__uncultured bacterium;D_7__;D_8__;D_9__;D_10__;D_11__;D_12__;D_13__;D_14__	2.14(0.14,33.64)		
ASV_683	Firmicutes;D_2__Clostridia;D_3__Clostridiales;D_4__Ruminococcaceae;D_5__Ruminiclostridium 5;D_6__uncultured organism;D_7__;D_8__;D_9__;D_10__;D_11__;D_12__;D_13__;D_14__	1.16(0.44,3.04)	1.27(0.49,3.32)	
ASV_685	Firmicutes;D_2__Clostridia;D_3__Clostridiales;D_4__Lachnospiraceae;D_5__[Ruminococcus] torques group	0.35(0.06,2.04)	0.35(0.06,1.92)	
ASV_686	Firmicutes;D_2__Bacilli;D_3__Lactobacillales;D_4__Enterococcaceae;D_5__Enterococcus	3.78(0.52,27.49)	2.60(0.42,16.20)	
ASV_690	Firmicutes;D_2__Clostridia;D_3__Clostridiales;D_4__Lachnospiraceae;D_5__[Ruminococcus] torques group;D_6__uncultured Clostridiales bacterium;D_7__;D_8__;D_9__;D_10__;D_11__;D_12__;D_13__;D_14__	0.57(0.03,11.65)	0.86(0.05,15.54)	
ASV_692	Actinobacteria;D_2__Actinobacteria;D_3__Bifidobacteriales;D_4__Bifidobacteriaceae;D_5__Alloscardovia;D_6__Bifidobacterium longum subsp. longum;D_7__;D_8__;D_9__;D_10__;D_11__;D_12__;D_13__;D_14__	4.44(0.29,69.08)	17.22(0.82,363.12)	
ASV_695	Actinobacteria;D_2__Actinobacteria;D_3__Micrococcales;D_4__Micrococcaceae;D_5__Rothia	3.74(0.46,30.23)		
ASV_696	Actinobacteria;D_2__Actinobacteria;D_3__Actinomycetales;D_4__Actinomycetaceae;D_5__Actinomyces	4.74(1.01,22.25);p=0.04879	10.71(1.88,60.84);p=0.00747	↑
ASV_697	Firmicutes;D_2__Erysipelotrichia;D_3__Erysipelotrichales;D_4__Erysipelotrichaceae;D_5__Faecalitalea	0.51(0.10,2.61)	0.55(0.11,2.72)	
ASV_698	Firmicutes;D_2__Clostridia;D_3__Clostridiales;D_4__Lachnospiraceae;D_5__Sellimonas;D_6__Lachnoclostridium phocaeense;D_7__;D_8__;D_9__;D_10__;D_11__;D_12__;D_13__;D_14__	0.77(0.12,5.10)	0.76(0.12,4.83)	
ASV_699	Bacteroidetes;D_2__Bacteroidia;D_3__Bacteroidales;D_4__Prevotellaceae;D_5__Prevotella 6;D_6__uncultured bacterium;D_7__;D_8__;D_9__;D_10__;D_11__;D_12__;D_13__;D_14__	40.70(4.28,386.60);p=0.00125		
ASV_704	Firmicutes;D_2__Clostridia;D_3__Clostridiales;D_4__Ruminococcaceae;D_5__uncultured;D_6__uncultured bacterium;D_7__;D_8__;D_9__;D_10__;D_11__;D_12__;D_13__;D_14__	0.19(0.03,1.14)		
ASV_705	Firmicutes;D_2__Clostridia;D_3__Clostridiales;D_4__Family XI;D_5__Ezakiella;D_6__Fenollaria timonensis;D_7__;D_8__;D_9__;D_10__;D_11__;D_12__;D_13__;D_14__	0.39(0.05,2.75)	0.39(0.07,2.32)	
ASV_708	Firmicutes;D_2__Clostridia;D_3__Clostridiales;D_4__Ruminococcaceae;D_5__Hydrogenoanaerobacterium;D_6__uncultured bacterium;D_7__;D_8__;D_9__;D_10__;D_11__;D_12__;D_13__;D_14__	1.97(0.25,15.49)		
ASV_709	Firmicutes;D_2__Clostridia;D_3__Clostridiales;D_4__Ruminococcaceae;D_5__Oscillibacter;D_6__uncultured bacterium;D_7__;D_8__;D_9__;D_10__;D_11__;D_12__;D_13__;D_14__	1.37(0.08,22.40)		
ASV_711	Firmicutes;D_2__Clostridia;D_3__Clostridiales;D_4__Lachnospiraceae;D_5__uncultured organism;D_6__;D_7__;D_8__;D_9__;D_10__;D_11__;D_12__;D_13__;D_14__	0.41(0.05,3.48)	0.31(0.04,2.55)	
ASV_712	Bacteroidetes;D_2__Bacteroidia;D_3__Bacteroidales;D_4__Prevotellaceae;D_5__Prevotella;D_6__Prevotella disiens JCM 6334 = ATCC 29426;D_7__;D_8__;D_9__;D_10__;D_11__;D_12__;D_13__;D_14__	0.84(0.08,8.55)	0.49(0.05,4.81)	
ASV_713	Firmicutes;D_2__Clostridia;D_3__Clostridiales;D_4__Lachnospiraceae;D_5__Tyzzerella;D_6__uncultured bacterium;D_7__;D_8__;D_9__;D_10__;D_11__;D_12__;D_13__;D_14__	0.50(0.08,3.33)	0.40(0.06,2.51)	
ASV_714	Firmicutes;D_2__Clostridia;D_3__Clostridiales;D_4__Lachnospiraceae;D_5__Lachnoclostridium	0.71(0.16,3.25)	1.03(0.24,4.43)	
ASV_715	Proteobacteria;D_2__Alphaproteobacteria;D_3__Rhizobiales;D_4__Rhizobiaceae;D_5__Allorhizobium-Neorhizobium-Pararhizobium-Rhizobium	3.00(0.15,60.90)		
ASV_716	Proteobacteria;D_2__Gammaproteobacteria;D_3__Betaproteobacteriales;D_4__Burkholderiaceae	1.28(0.10,16.37)	1.69(0.12,24.25)	
ASV_718	Actinobacteria;D_2__Actinobacteria;D_3__Corynebacteriales;D_4__Corynebacteriaceae;D_5__Corynebacterium 1	0.80(0.02,35.33)		
ASV_719	Proteobacteria;D_2__Gammaproteobacteria;D_3__Betaproteobacteriales;D_4__Burkholderiaceae	1.73(0.39,7.69)	1.97(0.43,8.95)	

ASV_721	Actinobacteria;D_2__Actinobacteria;D_3__Actinomycetales;D_4__Actinomycetaceae;D_5__Actinomyces	1.53(0.51,4.57)	1.59(0.54,4.68)	
ASV_722	Firmicutes;D_2__Erysipelotrichia;D_3__Erysipelotrichales;D_4__Erysipelotrichaceae;D_5__Erysipelatoclostridium;D_6__Massiliomicrobiota timonensis;D_7__;D_8__;D_9__;D_10__;D_11__;D_12__;D_13__;D_14__	4.99(0.45,55.33)		
ASV_723	Firmicutes;D_2__Clostridia;D_3__Clostridiales;D_4__Ruminococcaceae;D_5__Ruminiclostridium;D_6__Massilioclostridium coli;D_7__;D_8__;D_9__;D_10__;D_11__;D_12__;D_13__;D_14__	1.86(0.30,11.39)	1.92(0.31,11.74)	
ASV_726	Firmicutes;D_2__Negativicutes;D_3__Selenomonadales;D_4__Veillonellaceae;D_5__Veillonella	0.22(0.01,3.94)	0.21(0.01,3.74)	
ASV_729	Firmicutes;D_2__Clostridia;D_3__Clostridiales;D_4__Lachnospiraceae;D_5__[Ruminococcus] gnavus group	0.14(0.01,2.43)		
ASV_733	Firmicutes;D_2__Clostridia;D_3__Clostridiales;D_4__Clostridiaceae 1;D_5__Clostridium sensu stricto 1	5.34(0.52,54.65)	3.79(0.44,32.37)	
ASV_734	Firmicutes;D_2__Clostridia;D_3__Clostridiales;D_4__Lachnospiraceae;D_5__Eisenbergiella;D_6__uncultured organism;D_7__;D_8__;D_9__;D_10__;D_11__;D_12__;D_13__;D_14__	0.48(0.15,1.61)		
ASV_738	Firmicutes;D_2__Clostridia;D_3__Clostridiales;D_4__Ruminococcaceae;D_5__Ruminiclostridium 9;D_6__uncultured bacterium;D_7__;D_8__;D_9__;D_10__;D_11__;D_12__;D_13__;D_14__	1.17(0.14,9.80)	1.34(0.19,9.59)	
ASV_740	Firmicutes;D_2__Clostridia;D_3__Clostridiales;D_4__Lachnospiraceae;D_5__Lachnospiraceae UCG-004;D_6__uncultured organism;D_7__;D_8__;D_9__;D_10__;D_11__;D_12__;D_13__;D_14__	1.63(0.29,9.32)	1.40(0.25,7.80)	
ASV_741	Firmicutes;D_2__Clostridia;D_3__Clostridiales;D_4__Lachnospiraceae;D_5__Lachnoclostridium;D_6__uncultured organism;D_7__;D_8__;D_9__;D_10__;D_11__;D_12__;D_13__;D_14__	0.68(0.04,10.39)		
ASV_744	Firmicutes;D_2__Clostridia;D_3__Clostridiales;D_4__Lachnospiraceae;D_5__Lachnoclostridium	4.74(0.34,66.48)	12.04(0.85,171.26)	
ASV_746	Firmicutes;D_2__Clostridia;D_3__Clostridiales;D_4__Ruminococcaceae;D_5__Oscillibacter;D_6__uncultured organism;D_7__;D_8__;D_9__;D_10__;D_11__;D_12__;D_13__;D_14__	0.16(0.02,1.48)	0.25(0.03,2.24)	
ASV_747	Firmicutes;D_2__Clostridia;D_3__Clostridiales;D_4__Lachnospiraceae;D_5__Sellimonas;D_6__uncultured bacterium;D_7__;D_8__;D_9__;D_10__;D_11__;D_12__;D_13__;D_14__	1.78(0.44,7.24)	1.31(0.33,5.14)	
ASV_749	Firmicutes;D_2__Erysipelotrichia;D_3__Erysipelotrichales;D_4__Erysipelotrichaceae;D_5__Candidatus Stoquefichus	8.84(0.94,82.94)	11.28(1.20,106.16);p=0.03409	↑
ASV_751	Firmicutes;D_2__Clostridia;D_3__Clostridiales;D_4__Lachnospiraceae	8.26(0.30,230.00)	5.92(0.22,162.45)	
ASV_754	Firmicutes;D_2__Clostridia;D_3__Clostridiales;D_4__Lachnospiraceae;D_5__Lachnospiraceae UCG-004	0.06(0.00,1.42)	0.07(0.00,1.78)	
ASV_755	Firmicutes;D_2__Clostridia;D_3__Clostridiales;D_4__Ruminococcaceae;D_5__Ruminococcaceae UCG-008;D_6__uncultured bacterium;D_7__;D_8__;D_9__;D_10__;D_11__;D_12__;D_13__;D_14__	0.27(0.01,7.30)		
ASV_757	Firmicutes;D_2__Erysipelotrichia;D_3__Erysipelotrichales;D_4__Erysipelotrichaceae;D_5__Faecalitalea;D_6__[Eubacterium] dolichum;D_7__;D_8__;D_9__;D_10__;D_11__;D_12__;D_13__;D_14__	7.18(0.52,99.71)	9.13(0.62,134.00)	
ASV_759	Actinobacteria;D_2__Actinobacteria;D_3__Actinomycetales;D_4__Actinomycetaceae;D_5__Actinomyces;D_6__unidentified;D_7__;D_8__;D_9__;D_10__;D_11__;D_12__;D_13__;D_14__	0.43(0.01,24.25)		
ASV_760	Firmicutes;D_2__Clostridia;D_3__Clostridiales;D_4__Family XIII;D_5__[Eubacterium] nodatum group	5.69(0.52,62.71)	5.70(0.50,65.07)	
ASV_761	Firmicutes;D_2__Erysipelotrichia;D_3__Erysipelotrichales;D_4__Erysipelotrichaceae;D_5__Merdibacter;D_6__uncultured bacterium;D_7__;D_8__;D_9__;D_10__;D_11__;D_12__;D_13__;D_14__	1.31(0.05,31.28)	1.57(0.06,39.51)	
ASV_765	Firmicutes;D_2__Clostridia;D_3__Clostridiales;D_4__Lachnospiraceae;D_5__[Ruminococcus] torques group;D_6__uncultured bacterium;D_7__;D_8__;D_9__;D_10__;D_11__;D_12__;D_13__;D_14__	0.33(0.03,3.65)	0.52(0.05,6.06)	
ASV_767	Firmicutes;D_2__Erysipelotrichia;D_3__Erysipelotrichales;D_4__Erysipelotrichaceae;D_5__[Clostridium] innocuum group	0.24(0.04,1.47)		
ASV_770	Firmicutes;D_2__Clostridia;D_3__Clostridiales;D_4__Lachnospiraceae;D_5__Lachnospiraceae UCG-004	1.51(0.03,83.43)		
ASV_773	Firmicutes;D_2__Clostridia;D_3__Clostridiales;D_4__Ruminococcaceae;D_5__Ruminiclostridium	1.41(0.12,16.22)	0.56(0.05,6.53)	
ASV_774	Firmicutes;D_2__Clostridia;D_3__Clostridiales;D_4__Ruminococcaceae;D_5__Faecalibacterium	0.42(0.02,7.29)	0.34(0.02,6.21)	
ASV_775	Actinobacteria;D_2__Actinobacteria;D_3__Propionibacteriales;D_4__Propionibacteriaceae;D_5__Cutibacterium		0.00(0.00,Inf)	
ASV_776	Firmicutes;D_2__Clostridia;D_3__Clostridiales;D_4__Lachnospiraceae;D_5__Eisenbergiella	1.72(0.09,33.36)		
ASV_777	Firmicutes;D_2__Clostridia;D_3__Clostridiales;D_4__Lachnospiraceae;D_5__Lachnoclostridium	1.53(0.10,23.70)		
ASV_779	Firmicutes;D_2__Clostridia;D_3__Clostridiales;D_4__Lachnospiraceae;D_5__Lachnoclostridium		4.95(0.50,48.95)	
ASV_780	Actinobacteria;D_2__Coriobacteriia;D_3__Coriobacteriales;D_4__Eggerthellaceae;D_5__Gordonibacter;D_6__unidentified;D_7__;D_8__;D_9__;D_10__;D_11__;D_12__;D_13__;D_14__	0.72(0.08,6.62)	1.20(0.14,10.00)	

ASV_781	Firmicutes;D_2__Clostridia;D_3__Clostridiales;D_4__Ruminococcaceae;D_5__Ruminococcaceae UCG-008;D_6__uncultured bacterium;D_7__;D_8__;D_9__;D_10__;D_11__;D_12__;D_13__;D_14__	1.22(0.10,14.98)	0.52(0.04,6.67)	
ASV_790	Firmicutes;D_2__Clostridia;D_3__Clostridiales;D_4__Lachnospiraceae;D_5__Lachnospira	5.78(0.07,504.67)		
ASV_792	Firmicutes;D_2__Clostridia;D_3__Clostridiales;D_4__Lachnospiraceae;D_5__Lachnospiraceae UCG-001;D_6__Lachnospiraceae bacterium TF01-11;D_7__;D_8__;D_9__;D_10__;D_11__;D_12__;D_13__;D_14__	0.30(0.03,3.11)		
ASV_793	Firmicutes;D_2__Clostridia;D_3__Clostridiales;D_4__Lachnospiraceae;D_5__Lachnospiraceae UCG-009	0.20(0.02,2.27)		
ASV_794	Firmicutes;D_2__Clostridia;D_3__Clostridiales;D_4__Lachnospiraceae;D_5__[Ruminococcus] torques group	1.65(0.25,11.07)	2.56(0.35,18.57)	
ASV_795	Firmicutes;D_2__Clostridia;D_3__Clostridiales;D_4__Lachnospiraceae;D_5__[Ruminococcus] gauvreauii group;D_6__uncultured bacterium;D_7__;D_8__;D_9__;D_10__;D_11__;D_12__;D_13__;D_14__	1.28(0.18,8.89)	1.37(0.20,9.46)	
ASV_798	Actinobacteria;D_2__Coriobacteriia;D_3__Coriobacteriales;D_4__Eggerthellaceae;D_5__Eggerthella;D_6__uncultured bacterium;D_7__;D_8__;D_9__;D_10__;D_11__;D_12__;D_13__;D_14__	0.13(0.01,1.88)		
ASV_805	Firmicutes;D_2__Clostridia;D_3__Clostridiales;D_4__Lachnospiraceae;D_5__Lachnospiraceae NK4A136 group	10.72(0.42,276.33)		
ASV_810	Firmicutes;D_2__Clostridia;D_3__Clostridiales;D_4__Lachnospiraceae;D_5__Marvinbryantia	0.85(0.14,5.18)	0.46(0.08,2.69)	
ASV_811	Firmicutes;D_2__Clostridia;D_3__Clostridiales;D_4__Ruminococcaceae;D_5__Ruminococcaceae UCG-010;D_6__uncultured bacterium;D_7__;D_8__;D_9__;D_10__;D_11__;D_12__;D_13__;D_14__	0.53(0.04,6.81)		
ASV_812	Firmicutes;D_2__Erysipelotrichia;D_3__Erysipelotrichales;D_4__Erysipelotrichaceae;D_5__[Clostridium] innocuum group	5.36(1.03,27.78);p=0.04564	7.11(1.38,36.47);p=0.01877	↑
ASV_813	Firmicutes;D_2__Clostridia;D_3__Clostridiales;D_4__Ruminococcaceae;D_5__uncultured	1.93(0.19,19.77)	4.40(0.39,49.21)	
ASV_814	Firmicutes;D_2__Clostridia;D_3__Clostridiales;D_4__Peptococcaceae;D_5__uncultured	0.78(0.15,4.22)	0.59(0.11,3.00)	
ASV_817	Firmicutes;D_2__Clostridia;D_3__Clostridiales;D_4__Ruminococcaceae	0.84(0.19,3.78)	0.84(0.19,3.78)	
ASV_819	Firmicutes;D_2__Clostridia;D_3__Clostridiales;D_4__Ruminococcaceae	0.30(0.05,1.96)	0.33(0.05,2.11)	
ASV_821	Firmicutes;D_2__Clostridia;D_3__Clostridiales;D_4__Lachnospiraceae;D_5__Shuttleworthia;D_6__uncultured bacterium;D_7__;D_8__;D_9__;D_10__;D_11__;D_12__;D_13__;D_14__	2.63(0.50,13.77)	3.10(0.63,15.20)	
ASV_822	Firmicutes;D_2__Clostridia;D_3__Clostridiales;D_4__Family XIII;D_5__Family XIII AD3011 group	4.57(0.28,74.84)		
ASV_825	Firmicutes;D_2__Clostridia;D_3__Clostridiales;D_4__Ruminococcaceae;D_5__Candidatus Soleaferrea;D_6__uncultured bacterium;D_7__;D_8__;D_9__;D_10__;D_11__;D_12__;D_13__;D_14__		1.23(0.17,9.08)	
ASV_826	Firmicutes;D_2__Clostridia;D_3__Clostridiales;D_4__Christensenellaceae;D_5__Christensenellaceae R-7 group		0.70(0.19,2.55)	
ASV_827	Firmicutes;D_2__Clostridia;D_3__Clostridiales;D_4__Ruminococcaceae;D_5__Caproiciproducens;D_6__uncultured bacterium;D_7__;D_8__;D_9__;D_10__;D_11__;D_12__;D_13__;D_14__	0.17(0.02,1.30)	0.19(0.03,1.36)	
ASV_828	Firmicutes;D_2__Clostridia;D_3__Clostridiales;D_4__Family XIII;D_5__Family XIII AD3011 group	0.56(0.05,6.52)	0.32(0.03,3.48)	
ASV_829	Firmicutes;D_2__Clostridia;D_3__Clostridiales;D_4__Christensenellaceae;D_5__Christensenellaceae R-7 group;D_6__uncultured Clostridium sp.;D_7__;D_8__;D_9__;D_10__;D_11__;D_12__;D_13__;D_14__	2.14(0.41,11.22)		
ASV_830	Firmicutes;D_2__Clostridia;D_3__Clostridiales;D_4__Eubacteriaceae;D_5__Anaerofustis;D_6__unidentified;D_7__;D_8__;D_9__;D_10__;D_11__;D_12__;D_13__;D_14__		1.71(0.15,19.30)	
ASV_831	Firmicutes;D_2__Clostridia;D_3__Clostridiales;D_4__Lachnospiraceae;D_5__GCA-900066755;D_6__uncultured bacterium;D_7__;D_8__;D_9__;D_10__;D_11__;D_12__;D_13__;D_14__	0.74(0.16,3.46)		
ASV_832	Firmicutes;D_2__Clostridia;D_3__Clostridiales	0.35(0.03,3.92)		
ASV_834	Firmicutes;D_2__Clostridia;D_3__Clostridiales;D_4__Lachnospiraceae;D_5__[Eubacterium] fissicatena group		0.65(0.03,13.21)	
ASV_835	Firmicutes;D_2__Clostridia;D_3__Clostridiales;D_4__Ruminococcaceae	3.99(0.16,99.98)		
ASV_836	Firmicutes;D_2__Clostridia;D_3__Clostridiales;D_4__Peptococcaceae;D_5__uncultured		0.95(0.07,12.40)	
ASV_837	Firmicutes;D_2__Clostridia;D_3__Clostridiales;D_4__Christensenellaceae;D_5__Christensenellaceae R-7 group;D_6__uncultured bacterium;D_7__;D_8__;D_9__;D_10__;D_11__;D_12__;D_13__;D_14__		10.46(0.58,187.56)	
ASV_838	Firmicutes;D_2__Clostridia;D_3__Clostridiales;D_4__Christensenellaceae;D_5__uncultured;D_6__uncultured bacterium;D_7__;D_8__;D_9__;D_10__;D_11__;D_12__;D_13__;D_14__	2.10(0.11,41.64)	1.91(0.11,32.23)	
ASV_839	Firmicutes;D_2__Clostridia;D_3__Clostridiales;D_4__Ruminococcaceae;D_5__Ruminococcaceae UCG-005	29.36(2.14,403.67);p=0.0115	23.38(1.71,319.88);p=0.0182	↑

ASV_840	Firmicutes;D_2__Clostridia;D_3__Clostridiales;D_4__Lachnospiraceae;D_5__Lachnospiraceae UCG-010;D_6__uncultured organism;D_7__;D_8__;D_9__;D_10__;D_11__;D_12__;D_13__;D_14__	0.15(0.01,2.80)		
ASV_842	Verrucomicrobia;D_2__Verrucomicrobiae;D_3__Verrucomicrobiales;D_4__Akkermansiaceae;D_5__Akkermansia	0.69(0.02,31.00)		
ASV_845	Firmicutes;D_2__Clostridia;D_3__Clostridiales;D_4__Ruminococcaceae;D_5__Harryflintia	1.15(0.18,7.44)	0.74(0.11,4.84)	
ASV_846	Firmicutes;D_2__Clostridia;D_3__Clostridiales;D_4__Ruminococcaceae;D_5__Candidatus Soleaferrea;D_6__uncultured bacterium;D_7__;D_8__;D_9__;D_10__;D_11__;D_12__;D_13__;D_14__	0.93(0.05,18.14)		
ASV_849	Firmicutes;D_2__Clostridia;D_3__Clostridiales;D_4__Clostridiales vadinBB60 group;D_5__uncultured organism;D_6__;D_7__;D_8__;D_9__;D_10__;D_11__;D_12__;D_13__;D_14__	0.27(0.08,0.97);p=0.04426		
ASV_850	Firmicutes;D_2__Clostridia;D_3__Clostridiales;D_4__Christensenellaceae;D_5__Christensenella;D_6__Christensenella minuta;D_7__;D_8__;D_9__;D_10__;D_11__;D_12__;D_13__;D_14__	0.30(0.06,1.57)	0.34(0.07,1.77)	
ASV_852	Firmicutes;D_2__Clostridia;D_3__Clostridiales;D_4__Lachnospiraceae;D_5__Blautia	0.74(0.02,32.20)	3.00(0.06,158.94)	
ASV_856	Firmicutes;D_2__Clostridia;D_3__Clostridiales;D_4__Ruminococcaceae;D_5__Oscillibacter;D_6__uncultured organism;D_7__;D_8__;D_9__;D_10__;D_11__;D_12__;D_13__;D_14__	0.20(0.01,3.99)	0.20(0.01,3.88)	
ASV_869	Firmicutes;D_2__Clostridia;D_3__Clostridiales;D_4__Lachnospiraceae;D_5__Coprococcus 2	0.24(0.01,5.04)		
ASV_887	Firmicutes;D_2__Clostridia;D_3__Clostridiales;D_4__Clostridiales vadinBB60 group;D_5__uncultured bacterium;D_6__;D_7__;D_8__;D_9__;D_10__;D_11__;D_12__;D_13__;D_14__	3.06(0.26,36.55)		
ASV_912	Firmicutes;D_2__Clostridia;D_3__Clostridiales;D_4__Ruminococcaceae;D_5__Fournierella;D_6__uncultured bacterium;D_7__;D_8__;D_9__;D_10__;D_11__;D_12__;D_13__;D_14__	3.57(0.23,55.23)		
ASV_921	Firmicutes;D_2__Clostridia;D_3__Clostridiales;D_4__Lachnospiraceae;D_5__Dorea	1.94(0.34,11.09)	2.50(0.44,14.23)	
ASV_922	Firmicutes;D_2__Clostridia;D_3__Clostridiales;D_4__Ruminococcaceae;D_5__Angelakisella;D_6__uncultured bacterium;D_7__;D_8__;D_9__;D_10__;D_11__;D_12__;D_13__;D_14__		0.22(0.04,1.18)	
ASV_923	Bacteroidetes;D_2__Bacteroidia;D_3__Bacteroidales;D_4__Bacteroidaceae;D_5__Bacteroides;D_6__Bacteroides stercoris ATCC 43183;D_7__;D_8__;D_9__;D_10__;D_11__;D_12__;D_13__;D_14__	0.04(0.00,0.89);p=0.04207		
ASV_925	Firmicutes;D_2__Clostridia;D_3__Clostridiales;D_4__Ruminococcaceae;D_5__Ruminococcaceae UCG-009	2.25(0.40,12.72)	1.16(0.21,6.38)	
ASV_932	Actinobacteria;D_2__Actinobacteria;D_3__Actinomycetales;D_4__Actinomycetaceae;D_5__Actinomyces	0.16(0.01,3.55)		
ASV_937	Firmicutes;D_2__Clostridia;D_3__Clostridiales;D_4__Family XI;D_5__Parvimonas	1.45(0.29,7.21)	0.74(0.15,3.56)	
ASV_943	Firmicutes;D_2__Clostridia;D_3__Clostridiales;D_4__Ruminococcaceae	2.44(0.08,78.08)	1.58(0.05,49.57)	
ASV_949	Firmicutes;D_2__Clostridia;D_3__Clostridiales;D_4__Family XI;D_5__Peptoniphilus;D_6__uncultured organism;D_7__;D_8__;D_9__;D_10__;D_11__;D_12__;D_13__;D_14__	0.35(0.01,8.49)	1.42(0.07,30.40)	
ASV_953	Firmicutes;D_2__Clostridia;D_3__Clostridiales;D_4__Ruminococcaceae;D_5__Subdoligranulum	0.11(0.02,0.55);p=0.007	0.09(0.02,0.45);p=0.00311	↓
ASV_954	Firmicutes;D_2__Clostridia;D_3__Clostridiales;D_4__Family XI;D_5__Peptoniphilus;D_6__Peptoniphilus lacrimalis;D_7__;D_8__;D_9__;D_10__;D_11__;D_12__;D_13__;D_14__	0.74(0.07,8.36)		
ASV_955	Bacteroidetes;D_2__Bacteroidia;D_3__Bacteroidales;D_4__Porphyromonadaceae;D_5__Porphyromonas	0.49(0.09,2.69)		
ASV_956	Firmicutes;D_2__Clostridia;D_3__Clostridiales;D_4__Family XI;D_5__Finegoldia;D_6__uncultured bacterium;D_7__;D_8__;D_9__;D_10__;D_11__;D_12__;D_13__;D_14__	6.93(0.64,75.03)	9.10(0.84,98.65)	
ASV_959	Firmicutes;D_2__Clostridia;D_3__Clostridiales;D_4__Eubacteriaceae;D_5__Anaerofustis;D_6__Anaerofustis sp. Marseille-P2441;D_7__;D_8__;D_9__;D_10__;D_11__;D_12__;D_13__;D_14__	14.31(1.06,194.09);p=0.04543		
ASV_964	Firmicutes;D_2__Clostridia;D_3__Clostridiales;D_4__Clostridiales vadinBB60 group;D_5__gut metagenome;D_6__;D_7__;D_8__;D_9__;D_10__;D_11__;D_12__;D_13__;D_14__	0.68(0.12,3.77)	1.31(0.24,7.11)	
ASV_965	Firmicutes;D_2__Negativicutes;D_3__Selenomonadales;D_4__Veillonellaceae;D_5__Dialister;D_6__uncultured bacterium;D_7__;D_8__;D_9__;D_10__;D_11__;D_12__;D_13__;D_14__	1.45(0.11,18.34)	1.15(0.09,14.54)	
ASV_966	Firmicutes;D_2__Clostridia;D_3__Clostridiales;D_4__Ruminococcaceae;D_5__Ruminococcaceae UCG-005;D_6__uncultured rumen bacterium;D_7__;D_8__;D_9__;D_10__;D_11__;D_12__;D_13__;D_14__	0.69(0.11,4.31)	0.76(0.12,4.78)	
ASV_968	Firmicutes;D_2__Clostridia;D_3__Clostridiales;D_4__Lachnospiraceae;D_5__Lachnospiraceae UCG-010;D_6__uncultured bacterium;D_7__;D_8__;D_9__;D_10__;D_11__;D_12__;D_13__;D_14__		0.25(0.02,2.96)	
ASV_969	Firmicutes;D_2__Clostridia;D_3__Clostridiales;D_4__Ruminococcaceae;D_5__Ruminococcaceae UCG-010	0.06(0.00,0.79);p=0.03204	0.07(0.01,0.92);p=0.04306	↓
ASV_973	Bacteroidetes;D_2__Bacteroidia;D_3__Bacteroidales;D_4__Porphyromonadaceae;D_5__Porphyromonas;D_6__unidentified;D_7__;D_8__;D_9__;D_10__;D_11__;D_12__;D_13__;D_14__		0.66(0.12,3.69)	
ASV_974	Firmicutes;D_2__Clostridia;D_3__Clostridiales;D_4__Ruminococcaceae;D_5__Ruminiclostridium 5	3.71(0.56,24.65)	6.00(0.82,43.90)	

ASV_976	Firmicutes;D_2__Clostridia;D_3__Clostridiales;D_4__Christensenellaceae;D_5__Christensenellaceae R-7 group;D_6__uncultured Christensenellaceae bacterium;D_7__;D_8__;D_9__;D_10__;D_11__;D_12__;D_13__;D_14__	0.39(0.08,1.75)	0.41(0.09,1.85)	
ASV_978	Bacteroidetes;D_2__Bacteroidia;D_3__Bacteroidales;D_4__Prevotellaceae;D_5__Prevotella;D_6__Prevotella bivia;D_7__;D_8__;D_9__;D_10__;D_11__;D_12__;D_13__;D_14__	7.52(0.31,185.31)	1.78(0.07,43.11)	
ASV_980	Bacteroidetes;D_2__Bacteroidia;D_3__Bacteroidales;D_4__Porphyromonadaceae;D_5__Porphyromonas	1.39(0.20,9.58)	1.94(0.28,13.32)	
ASV_981	Firmicutes;D_2__Clostridia;D_3__Clostridiales;D_4__Family XI;D_5__Anaerococcus	3.45(0.32,37.29)	5.00(0.46,54.47)	
ASV_984	Firmicutes;D_2__Clostridia;D_3__Clostridiales;D_4__Lachnospiraceae;D_5__Marvinbryantia;D_6__uncultured bacterium;D_7__;D_8__;D_9__;D_10__;D_11__;D_12__;D_13__;D_14__	0.12(0.02,0.68);p=0.01666	0.07(0.01,0.37);p=0.00211	↓
ASV_985	Firmicutes;D_2__Clostridia;D_3__Clostridiales;D_4__Lachnospiraceae;D_5__Lachnoclostridium	18.95(1.24,290.15);p=0.03458		
ASV_986	Firmicutes;D_2__Clostridia;D_3__Clostridiales;D_4__Ruminococcaceae;D_5__Ruminiclostridium 5;D_6__uncultured bacterium;D_7__;D_8__;D_9__;D_10__;D_11__;D_12__;D_13__;D_14__	1.22(0.31,4.79)	0.88(0.23,3.29)	
ASV_1003	Firmicutes;D_2__Clostridia;D_3__Clostridiales;D_4__Ruminococcaceae;D_5__Fournierella;D_6__uncultured organism;D_7__;D_8__;D_9__;D_10__;D_11__;D_12__;D_13__;D_14__	0.21(0.02,2.27)	0.62(0.07,5.89)	
ASV_1004	Firmicutes;D_2__Clostridia;D_3__Clostridiales;D_4__Ruminococcaceae	7.42(0.64,86.44)	2.66(0.26,26.91)	
ASV_1005	Firmicutes;D_2__Clostridia;D_3__Clostridiales;D_4__Ruminococcaceae	0.46(0.08,2.52)		
ASV_1009	Firmicutes;D_2__Clostridia;D_3__Clostridiales;D_4__Ruminococcaceae;D_5__GCA-900066225;D_6__uncultured bacterium;D_7__;D_8__;D_9__;D_10__;D_11__;D_12__;D_13__;D_14__	1.72(0.14,20.44)		
ASV_1010	Firmicutes;D_2__Clostridia;D_3__Clostridiales;D_4__Ruminococcaceae;D_5__Ruminiclostridium 5;D_6__uncultured organism;D_7__;D_8__;D_9__;D_10__;D_11__;D_12__;D_13__;D_14__	0.93(0.10,8.48)	0.93(0.10,8.47)	
ASV_1013	Actinobacteria;D_2__Actinobacteria;D_3__Corynebacteriales;D_4__Corynebacteriaceae;D_5__Corynebacterium;D_6__Corynebacterium durum;D_7__;D_8__;D_9__;D_10__;D_11__;D_12__;D_13__;D_14__		1.95(0.54,6.96)	
ASV_1017	Firmicutes;D_2__Clostridia;D_3__Clostridiales;D_4__Ruminococcaceae;D_5__[Eubacterium] coprostanoligenes group;D_6__uncultured organism;D_7__;D_8__;D_9__;D_10__;D_11__;D_12__;D_13__;D_14__	0.55(0.02,14.59)		
ASV_1018	Firmicutes;D_2__Clostridia;D_3__Clostridiales;D_4__Christensenellaceae;D_5__Christensenellaceae R-7 group;D_6__uncultured bacterium;D_7__;D_8__;D_9__;D_10__;D_11__;D_12__;D_13__;D_14__	2.80(0.44,17.69)		
ASV_1019	Firmicutes;D_2__Clostridia;D_3__Clostridiales;D_4__Lachnospiraceae;D_5__Sellimonas;D_6__uncultured Firmicutes bacterium;D_7__;D_8__;D_9__;D_10__;D_11__;D_12__;D_13__;D_14__	4.18(0.25,69.63)	6.03(0.37,97.14)	
ASV_1020	Firmicutes;D_2__Erysipelotrichia;D_3__Erysipelotrichales;D_4__Erysipelotrichaceae;D_5__Erysipelatoclostridium;D_6__Massiliomicrobiota timonensis;D_7__;D_8__;D_9__;D_10__;D_11__;D_12__;D_13__;D_14__	2.21(0.38,12.91)	2.41(0.41,14.06)	
ASV_1021	Actinobacteria;D_2__Actinobacteria;D_3__Actinomycetales;D_4__Actinomycetaceae;D_5__Actinomyces	0.06(0.00,1.54)	0.01(0.00,0.58);p=0.02461	↓
ASV_1022	Firmicutes;D_2__Clostridia;D_3__Clostridiales;D_4__Ruminococcaceae	3.38(0.24,47.80)		
ASV_1024	Firmicutes;D_2__Clostridia;D_3__Clostridiales;D_4__Lachnospiraceae		6.71(0.57,78.58)	
ASV_1026	Firmicutes;D_2__Clostridia;D_3__Clostridiales;D_4__Lachnospiraceae	0.19(0.01,6.03)		
ASV_1029	Firmicutes;D_2__Clostridia;D_3__Clostridiales;D_4__Ruminococcaceae;D_5__uncultured;D_6__uncultured Firmicutes bacterium;D_7__;D_8__;D_9__;D_10__;D_11__;D_12__;D_13__;D_14__	1.50(0.25,9.15)		
ASV_1030	Firmicutes;D_2__Clostridia;D_3__Clostridiales;D_4__Ruminococcaceae	0.25(0.06,0.98);p=0.04669	0.23(0.06,0.90);p=0.03526	↓
ASV_1031	Actinobacteria;D_2__Actinobacteria;D_3__Bifidobacteriales;D_4__Bifidobacteriaceae;D_5__Scardovia;D_6__unidentified;D_7__;D_8__;D_9__;D_10__;D_11__;D_12__;D_13__;D_14__	2.75(0.20,38.70)		
ASV_1040	Firmicutes;D_2__Bacilli;D_3__Lactobacillales;D_4__Lactobacillaceae;D_5__Lactobacillus	17.40(0.82,369.55)	21.36(0.84,539.72)	
ASV_1048	Bacteroidetes;D_2__Bacteroidia;D_3__Bacteroidales;D_4__Prevotellaceae;D_5__Paraprevotella	0.70(0.05,10.79)		
ASV_1049	Firmicutes;D_2__Bacilli;D_3__Lactobacillales;D_4__Lactobacillaceae;D_5__Lactobacillus;D_6__Lactobacillus rhamnosus;D_7__;D_8__;D_9__;D_10__;D_11__;D_12__;D_13__;D_14__	89.74(5.68,1416.83);p=0.0014	156.03(8.21,2964.64);p=0.00078	↑
ASV_1052	Firmicutes;D_2__Clostridia;D_3__Clostridiales;D_4__Ruminococcaceae;D_5__Anaerofilum;D_6__uncultured bacterium;D_7__;D_8__;D_9__;D_10__;D_11__;D_12__;D_13__;D_14__		0.91(0.22,3.85)	
ASV_1055	Firmicutes;D_2__Clostridia;D_3__Clostridiales;D_4__Ruminococcaceae;D_5__GCA-900066225;D_6__uncultured bacterium;D_7__;D_8__;D_9__;D_10__;D_11__;D_12__;D_13__;D_14__	0.85(0.10,7.03)	1.65(0.23,11.76)	
ASV_1057	Actinobacteria;D_2__Coriobacteriia;D_3__Coriobacteriales;D_4__Coriobacteriaceae;D_5__Collinsella;D_6__uncultured bacterium;D_7__;D_8__;D_9__;D_10__;D_11__;D_12__;D_13__;D_14__	0.54(0.13,2.26)	0.76(0.18,3.09)	
ASV_1059	Firmicutes;D_2__Clostridia;D_3__Clostridiales;D_4__Ruminococcaceae;D_5__uncultured	24.00(1.12,514.98);p=0.0422		

ASV_1062	Firmicutes;D_2__Clostridia;D_3__Clostridiales;D_4__Ruminococcaceae;D_5__GCA-900066225;D_6__uncultured Ruminococcus sp.;D_7__;D_8__;D_9__;D_10__;D_11__;D_12__;D_13__;D_14__	0.34(0.05,2.34)	0.45(0.07,2.93)	
ASV_1064	Firmicutes;D_2__Clostridia;D_3__Clostridiales;D_4__Ruminococcaceae;D_5__Ruminococcaceae UCG-010;D_6__gut metagenome;D_7__;D_8__;D_9__;D_10__;D_11__;D_12__;D_13__;D_14__		0.19(0.01,2.61)	
ASV_1066	Actinobacteria;D_2__Actinobacteria;D_3__Corynebacteriales;D_4__Corynebacteriaceae;D_5__Corynebacterium 1;D_6__Corynebacterium pseudodiphtheriticum;D_7__;D_8__;D_9__;D_10__;D_11__;D_12__;D_13__;D_14__	0.98(0.03,37.30)		
ASV_1067	Firmicutes;D_2__Clostridia;D_3__Clostridiales;D_4__Ruminococcaceae;D_5__Ruminococcaceae UCG-005;D_6__uncultured Clostridiales bacterium;D_7__;D_8__;D_9__;D_10__;D_11__;D_12__;D_13__;D_14__		3321179236534424.00(0.00,Inf)	
ASV_1068	Bacteroidetes;D_2__Bacteroidia;D_3__Bacteroidales;D_4__Tannerellaceae;D_5__Parabacteroides	1.55(0.17,13.82)	1.79(0.19,17.15)	
ASV_1070	Firmicutes;D_2__Clostridia;D_3__Clostridiales;D_4__Lachnospiraceae;D_5__Lactonifactor	0.53(0.08,3.57)	0.52(0.08,3.43)	
ASV_1072	Firmicutes;D_2__Clostridia;D_3__Clostridiales;D_4__Lachnospiraceae;D_5__Lachnoclostridium	2.28(0.25,20.92)		
ASV_1104	Firmicutes;D_2__Clostridia;D_3__Clostridiales;D_4__Lachnospiraceae;D_5__Lachnoclostridium	2.70(0.08,86.66)		
ASV_1107	Firmicutes;D_2__Clostridia;D_3__Clostridiales;D_4__Ruminococcaceae;D_5__UBA1819	0.33(0.02,6.51)	0.13(0.01,2.32)	
ASV_1108	Firmicutes;D_2__Clostridia;D_3__Clostridiales;D_4__Lachnospiraceae;D_5__Lachnospiraceae UCG-004	0.96(0.01,74.55)		
ASV_1114	Firmicutes;D_2__Clostridia;D_3__Clostridiales;D_4__Ruminococcaceae;D_5__Butyricicoccus;D_6__uncultured bacterium;D_7__;D_8__;D_9__;D_10__;D_11__;D_12__;D_13__;D_14__	1.87(0.03,106.90)		
ASV_1129	Bacteroidetes;D_2__Bacteroidia;D_3__Bacteroidales;D_4__Prevotellaceae;D_5__Prevotella;D_6__Prevotella buccalis;D_7__;D_8__;D_9__;D_10__;D_11__;D_12__;D_13__;D_14__	0.64(0.11,3.89)		
ASV_1131	Firmicutes;D_2__Clostridia;D_3__Clostridiales;D_4__Peptostreptococcaceae	0.04(0.00,0.79);p=0.03379	0.05(0.00,0.98);p=0.04837	↓
ASV_1132	Firmicutes;D_2__Bacilli;D_3__Lactobacillales;D_4__Streptococcaceae;D_5__Streptococcus	0.15(0.02,1.19)	0.24(0.03,1.64)	
ASV_1133	Firmicutes;D_2__Clostridia;D_3__Clostridiales;D_4__Family XI;D_5__Parvimonas	0.12(0.00,4.81)		
ASV_1142	Firmicutes;D_2__Clostridia;D_3__Clostridiales;D_4__Christensenellaceae;D_5__Christensenellaceae R-7 group;D_6__uncultured bacterium;D_7__;D_8__;D_9__;D_10__;D_11__;D_12__;D_13__;D_14__		0.29(0.02,5.60)	
ASV_1143	Firmicutes;D_2__Erysipelotrichia;D_3__Erysipelotrichales;D_4__Erysipelotrichaceae;D_5__Coprobacillus;D_6__uncultured bacterium;D_7__;D_8__;D_9__;D_10__;D_11__;D_12__;D_13__;D_14__	0.09(0.01,1.25)		
ASV_1145	Bacteroidetes;D_2__Bacteroidia;D_3__Bacteroidales;D_4__Porphyromonadaceae;D_5__Porphyromonas;D_6__unidentified;D_7__;D_8__;D_9__;D_10__;D_11__;D_12__;D_13__;D_14__		1.79(0.10,31.93)	
ASV_1147	Firmicutes;D_2__Bacilli;D_3__Lactobacillales;D_4__Streptococcaceae;D_5__Streptococcus	0.67(0.13,3.42)	0.58(0.11,2.95)	
ASV_1148	Firmicutes;D_2__Clostridia;D_3__Clostridiales;D_4__Family XIII;D_5__S5-A14a		2.44(0.17,35.70)	
ASV_1149	Firmicutes;D_2__Clostridia;D_3__Clostridiales;D_4__Ruminococcaceae	2.40(0.13,43.14)	2.99(0.17,52.60)	
ASV_1150	Firmicutes;D_2__Bacilli;D_3__Lactobacillales;D_4__Streptococcaceae;D_5__Streptococcus	1.45(0.09,24.23)	0.37(0.04,3.74)	
ASV_1151	Firmicutes;D_2__Negativicutes;D_3__Selenomonadales;D_4__Veillonellaceae;D_5__Dialister;D_6__uncultured bacterium;D_7__;D_8__;D_9__;D_10__;D_11__;D_12__;D_13__;D_14__	0.22(0.02,2.48)	0.33(0.03,3.26)	
ASV_1154	Actinobacteria;D_2__Actinobacteria;D_3__Actinomycetales;D_4__Actinomycetaceae;D_5__Mobiluncus	0.38(0.02,6.87)		
ASV_1160	Firmicutes;D_2__Clostridia;D_3__Clostridiales;D_4__Ruminococcaceae;D_5__Candidatus Soleaferrea;D_6__uncultured bacterium;D_7__;D_8__;D_9__;D_10__;D_11__;D_12__;D_13__;D_14__	0.51(0.10,2.61)		
ASV_1162	Firmicutes;D_2__Clostridia;D_3__Clostridiales;D_4__Ruminococcaceae;D_5__[Eubacterium] coprostanoligenes group;D_6__uncultured organism;D_7__;D_8__;D_9__;D_10__;D_11__;D_12__;D_13__;D_14__	0.43(0.04,4.76)	0.52(0.05,5.46)	
ASV_1164	Actinobacteria;D_2__Actinobacteria;D_3__Actinomycetales;D_4__Actinomycetaceae;D_5__Actinomyces	3.08(0.50,18.92)	3.54(0.58,21.65)	
ASV_1195	Firmicutes;D_2__Clostridia;D_3__Clostridiales;D_4__Ruminococcaceae;D_5__Intestinimonas;D_6__uncultured bacterium;D_7__;D_8__;D_9__;D_10__;D_11__;D_12__;D_13__;D_14__	1.87(0.09,38.85)		
ASV_1196	Firmicutes;D_2__Clostridia;D_3__Clostridiales;D_4__Ruminococcaceae;D_5__uncultured	0.59(0.04,8.09)		
ASV_1198	Firmicutes;D_2__Clostridia;D_3__Clostridiales;D_4__Family XIII;D_5__[Eubacterium] brachy group	0.49(0.01,17.71)		
ASV_1199	Firmicutes;D_2__Clostridia;D_3__Clostridiales;D_4__Ruminococcaceae;D_5__Ruminiclostridium 1		1.02(0.05,19.63)	

ASV_1201	Cyanobacteria;D_2__Oxyphotobacteria;D_3__Chloroplast	9.33(0.32,273.46)		
ASV_1202	Firmicutes;D_2__Clostridia;D_3__Clostridiales;D_4__Peptococcaceae;D_5__uncultured;D_6__uncultured bacterium;D_7__;D_8__;D_9__;D_10__;D_11__;D_12__;D_13__;D_14__	0.26(0.01,6.96)		
ASV_1208	Firmicutes;D_2__Erysipelotrichia;D_3__Erysipelotrichales;D_4__Erysipelotrichaceae;D_5__[Clostridium] innocuum group	0.24(0.02,3.95)	0.78(0.05,11.72)	
ASV_1209	Firmicutes;D_2__Clostridia;D_3__Clostridiales;D_4__Ruminococcaceae;D_5__Papillibacter;D_6__uncultured bacterium;D_7__;D_8__;D_9__;D_10__;D_11__;D_12__;D_13__;D_14__		2.20(0.42,11.62)	
ASV_1210	Firmicutes;D_2__Clostridia;D_3__Clostridiales;D_4__Family XIII;D_5__[Eubacterium] nodatum group;D_6__[Eubacterium] sulci;D_7__;D_8__;D_9__;D_10__;D_11__;D_12__;D_13__;D_14__	0.65(0.08,5.29)		
ASV_1216	Firmicutes;D_2__Clostridia;D_3__Clostridiales;D_4__Ruminococcaceae	0.31(0.02,3.90)		
ASV_1217	Firmicutes;D_2__Clostridia;D_3__Clostridiales;D_4__Christensenellaceae;D_5__Christensenellaceae R-7 group	0.18(0.02,1.49)	0.21(0.03,1.82)	
ASV_1220	Bacteroidetes;D_2__Bacteroidia;D_3__Bacteroidales;D_4__Prevotellaceae;D_5__Prevotella;D_6__Chlamydia trachomatis;D_7__;D_8__;D_9__;D_10__;D_11__;D_12__;D_13__;D_14__	3.28(0.50,21.38)	2.35(0.36,15.22)	
ASV_1221	Firmicutes;D_2__Clostridia;D_3__Clostridiales;D_4__Ruminococcaceae;D_5__Faecalibacterium	0.18(0.01,3.70)	0.17(0.01,3.50)	
ASV_1222	Firmicutes;D_2__Bacilli;D_3__Lactobacillales;D_4__Lactobacillaceae;D_5__Lactobacillus;D_6__Lactobacillus rhamnosus;D_7__;D_8__;D_9__;D_10__;D_11__;D_12__;D_13__;D_14__		0.00(0.00,Inf)	
ASV_1223	Firmicutes;D_2__Clostridia;D_3__Clostridiales;D_4__Ruminococcaceae	1.17(0.08,16.50)	0.53(0.04,6.75)	
ASV_1227	Firmicutes;D_2__Clostridia;D_3__Clostridiales;D_4__Ruminococcaceae;D_5__Ruminiclostridium 5;D_6__uncultured bacterium;D_7__;D_8__;D_9__;D_10__;D_11__;D_12__;D_13__;D_14__	1.47(0.16,13.60)	1.05(0.11,10.02)	
ASV_1228	Firmicutes;D_2__Clostridia;D_3__Clostridiales;D_4__Christensenellaceae;D_5__uncultured;D_6__uncultured bacterium;D_7__;D_8__;D_9__;D_10__;D_11__;D_12__;D_13__;D_14__	0.89(0.08,9.51)	0.80(0.07,8.64)	
ASV_1229	Firmicutes;D_2__Clostridia;D_3__Clostridiales;D_4__Ruminococcaceae;D_5__uncultured bacterium;D_6__;D_7__;D_8__;D_9__;D_10__;D_11__;D_12__;D_13__;D_14__		5.09(0.24,107.80)	
ASV_1230	Firmicutes;D_2__Clostridia;D_3__Clostridiales;D_4__Ruminococcaceae;D_5__Acetanaerobacterium	2.04(0.20,21.10)		
ASV_1231	Firmicutes;D_2__Clostridia;D_3__Clostridiales;D_4__Lachnospiraceae	0.28(0.03,2.98)	0.47(0.05,4.71)	
ASV_1233	Firmicutes;D_2__Bacilli;D_3__Lactobacillales;D_4__Streptococcaceae;D_5__Streptococcus	3.36(0.21,54.63)		
ASV_1248	Firmicutes;D_2__Clostridia;D_3__Clostridiales;D_4__Ruminococcaceae;D_5__Ruminococcaceae UCG-002	9.74(0.43,220.01)		
ASV_1256	Firmicutes;D_2__Clostridia;D_3__Clostridiales;D_4__Ruminococcaceae;D_5__uncultured	0.19(0.01,4.79)		
ASV_1257	Firmicutes;D_2__Clostridia;D_3__Clostridiales;D_4__Lachnospiraceae;D_5__[Ruminococcus] torques group	0.31(0.01,7.82)		
ASV_1260	Firmicutes;D_2__Bacilli;D_3__Lactobacillales;D_4__Carnobacteriaceae;D_5__Granulicatella	2.34(0.17,31.42)		
ASV_1262	Firmicutes;D_2__Clostridia;D_3__Clostridiales;D_4__Lachnospiraceae	8.58(0.22,328.28)		
ASV_1264	Firmicutes;D_2__Clostridia;D_3__Clostridiales;D_4__Family XIII;D_5__Family XIII AD3011 group;D_6__uncultured organism;D_7__;D_8__;D_9__;D_10__;D_11__;D_12__;D_13__;D_14__	2.00(0.11,36.89)	3.93(0.17,90.00)	
ASV_1276	Firmicutes;D_2__Clostridia;D_3__Clostridiales;D_4__Lachnospiraceae;D_5__Coprococcus 3;D_6__uncultured bacterium;D_7__;D_8__;D_9__;D_10__;D_11__;D_12__;D_13__;D_14__	30.66(0.42,2231.08)		
ASV_1284	Firmicutes;D_2__Clostridia;D_3__Clostridiales;D_4__Family XIII;D_5__[Eubacterium] brachy group;D_6__Eubacterium brachy ATCC 33089;D_7__;D_8__;D_9__;D_10__;D_11__;D_12__;D_13__;D_14__	0.69(0.07,6.88)		
ASV_1286	Deinococcus-Thermus;D_2__Deinococci;D_3__Thermales;D_4__Thermaceae;D_5__Meiothermus	0.93(0.04,19.40)		
ASV_1287	Firmicutes;D_2__Clostridia;D_3__Clostridiales;D_4__Lachnospiraceae;D_5__[Ruminococcus] gnavus group	1.29(0.08,20.60)	1.33(0.08,21.49)	
ASV_1289	Firmicutes;D_2__Erysipelotrichia;D_3__Erysipelotrichales;D_4__Erysipelotrichaceae;D_5__Holdemania;D_6__uncultured bacterium;D_7__;D_8__;D_9__;D_10__;D_11__;D_12__;D_13__;D_14__	5.15(0.23,112.89)		
ASV_1294	Proteobacteria;D_2__Gammaproteobacteria;D_3__Betaproteobacteriales;D_4__Burkholderiaceae;D_5__Sutterella;D_6__uncultured organism;D_7__;D_8__;D_9__;D_10__;D_11__;D_12__;D_13__;D_14__	1.74(0.09,34.21)		
ASV_1301	Firmicutes;D_2__Clostridia;D_3__Clostridiales;D_4__Ruminococcaceae;D_5__Candidatus Soleaferrea	1.34(0.04,42.52)		
ASV_1302	Firmicutes;D_2__Clostridia;D_3__Clostridiales;D_4__Ruminococcaceae;D_5__Ruminiclostridium 9	0.57(0.03,12.11)		

ASV_1303	Firmicutes;D_2__Clostridia;D_3__Clostridiales;D_4__Ruminococcaceae;D_5__uncultured	0.28(0.04,2.21)	0.26(0.04,1.90)	
ASV_1305	Firmicutes;D_2__Clostridia;D_3__Clostridiales;D_4__Ruminococcaceae;D_5__Papillibacter;D_6__uncultured bacterium;D_7__;D_8__;D_9__;D_10__;D_11__;D_12__;D_13__;D_14__	0.70(0.04,12.26)		
ASV_1306	Actinobacteria;D_2__Actinobacteria;D_3__Actinomycetales;D_4__Actinomycetaceae;D_5__F0332;D_6__uncultured bacterium;D_7__;D_8__;D_9__;D_10__;D_11__;D_12__;D_13__;D_14__	3.13(0.42,23.04)		
ASV_1320	Fusobacteria;D_2__Fusobacteriia;D_3__Fusobacteriales;D_4__Fusobacteriaceae;D_5__Fusobacterium	0.03(0.00,2.06)		
ASV_1339	Patescibacteria;D_2__Saccharimonadia;D_3__Saccharimonadales;D_4__Candidatus Saccharibacteria bacterium UB2523;D_5__;D_6__;D_7__;D_8__;D_9__;D_10__;D_11__;D_12__;D_13__;D_14__	0.60(0.01,35.71)		
ASV_1343	Firmicutes;D_2__Clostridia;D_3__Clostridiales;D_4__Lachnospiraceae;D_5__Dorea	0.71(0.03,15.99)		
ASV_1344	Firmicutes;D_2__Clostridia;D_3__Clostridiales;D_4__Family XIII;D_5__Family XIII AD3011 group	0.83(0.05,12.53)	0.84(0.06,12.27)	
ASV_1345	Firmicutes;D_2__Clostridia;D_3__Clostridiales;D_4__Christensenellaceae;D_5__uncultured;D_6__uncultured bacterium;D_7__;D_8__;D_9__;D_10__;D_11__;D_12__;D_13__;D_14__	2.59(0.12,57.61)		
ASV_1349	Firmicutes;D_2__Clostridia;D_3__Clostridiales;D_4__Lachnospiraceae;D_5__Lachnospiraceae NK4A136 group	3.38(0.06,189.38)		
ASV_1357	Firmicutes;D_2__Clostridia;D_3__Clostridiales;D_4__Ruminococcaceae;D_5__Ruminiclostridium 1;D_6__uncultured bacterium;D_7__;D_8__;D_9__;D_10__;D_11__;D_12__;D_13__;D_14__	0.66(0.04,10.19)	0.89(0.06,12.90)	
ASV_1361	Firmicutes;D_2__Clostridia;D_3__Clostridiales;D_4__Lachnospiraceae;D_5__Lachnoclostridium;D_6__Eubacterium sp. Marseille-P3202;D_7__;D_8__;D_9__;D_10__;D_11__;D_12__;D_13__;D_14__	1.07(0.07,15.94)		
ASV_1362	Actinobacteria;D_2__Actinobacteria;D_3__Micrococcales;D_4__Micrococcaceae;D_5__Rothia	2.02(0.36,11.30)	2.78(0.54,14.27)	
ASV_1364	Firmicutes;D_2__Clostridia;D_3__Clostridiales;D_4__Lachnospiraceae;D_5__GCA-900066575;D_6__uncultured bacterium;D_7__;D_8__;D_9__;D_10__;D_11__;D_12__;D_13__;D_14__	0.40(0.02,7.37)		
ASV_1369	Firmicutes;D_2__Clostridia;D_3__Clostridiales;D_4__Clostridiales vadinBB60 group;D_5__uncultured bacterium;D_6__;D_7__;D_8__;D_9__;D_10__;D_11__;D_12__;D_13__;D_14__	2.05(0.07,56.96)		
ASV_1371	Firmicutes;D_2__Clostridia;D_3__Clostridiales;D_4__Ruminococcaceae;D_5__Ruminococcaceae UCG-010;D_6__uncultured organism;D_7__;D_8__;D_9__;D_10__;D_11__;D_12__;D_13__;D_14__	0.41(0.02,8.77)		
ASV_1374	Firmicutes;D_2__Clostridia;D_3__Clostridiales;D_4__Ruminococcaceae;D_5__Ruminococcaceae NK4A214 group	2762848831044.24(0.00,Inf)		
ASV_1375	Bacteroidetes;D_2__Bacteroidia;D_3__Bacteroidales;D_4__Rikenellaceae;D_5__Alistipes;D_6__uncultured bacterium;D_7__;D_8__;D_9__;D_10__;D_11__;D_12__;D_13__;D_14__	10.82(0.89,131.01)	13.79(0.97,196.73)	
ASV_1376	Proteobacteria;D_2__Gammaproteobacteria;D_3__Betaproteobacteriales;D_4__Burkholderiaceae;D_5__Aquabacterium;D_6__Aquabacterium citratiphilum;D_7__;D_8__;D_9__;D_10__;D_11__;D_12__;D_13__;D_14__	2.55(0.25,25.54)	4.07(0.31,53.12)	
ASV_1378	Firmicutes;D_2__Erysipelotrichia;D_3__Erysipelotrichales;D_4__Erysipelotrichaceae;D_5__Merdibacter;D_6__uncultured bacterium;D_7__;D_8__;D_9__;D_10__;D_11__;D_12__;D_13__;D_14__	1.08(0.20,5.76)	0.73(0.14,3.67)	
ASV_1393	Firmicutes;D_2__Clostridia;D_3__Clostridiales;D_4__Ruminococcaceae;D_5__Intestinimonas;D_6__uncultured Clostridiales bacterium;D_7__;D_8__;D_9__;D_10__;D_11__;D_12__;D_13__;D_14__		1.96(0.21,17.90)	
ASV_1405	Actinobacteria;D_2__Actinobacteria;D_3__Bifidobacteriales;D_4__Bifidobacteriaceae;D_5__Bifidobacterium		0.04(0.00,1.47)	
ASV_1419	Firmicutes;D_2__Bacilli;D_3__Bacillales;D_4__Bacillaceae;D_5__Bacillus;D_6__Bacillus vietnamensis;D_7__;D_8__;D_9__;D_10__;D_11__;D_12__;D_13__;D_14__		1113720065499111.00(0.00,Inf)	
ASV_1422	Firmicutes;D_2__Clostridia;D_3__Clostridiales;D_4__Lachnospiraceae;D_5__Lachnoclostridium	1.62(0.12,22.44)	9.10(0.42,196.05)	
ASV_1424	Bacteroidetes;D_2__Bacteroidia;D_3__Bacteroidales;D_4__Marinifilaceae;D_5__Butyricimonas;D_6__uncultured organism;D_7__;D_8__;D_9__;D_10__;D_11__;D_12__;D_13__;D_14__	7.19(0.51,101.67)		
ASV_1459	Firmicutes;D_2__Clostridia;D_3__Clostridiales;D_4__Ruminococcaceae;D_5__Fournierella;D_6__uncultured bacterium;D_7__;D_8__;D_9__;D_10__;D_11__;D_12__;D_13__;D_14__	0.24(0.00,12.48)		
ASV_1464	Firmicutes;D_2__Clostridia;D_3__Clostridiales;D_4__Ruminococcaceae;D_5__Subdoligranulum;D_6__uncultured bacterium;D_7__;D_8__;D_9__;D_10__;D_11__;D_12__;D_13__;D_14__	4.12(0.24,71.97)	3.71(0.22,62.70)	
ASV_1465	Bacteroidetes;D_2__Bacteroidia;D_3__Bacteroidales;D_4__Prevotellaceae;D_5__Prevotella 7;D_6__uncultured bacterium;D_7__;D_8__;D_9__;D_10__;D_11__;D_12__;D_13__;D_14__	0.49(0.02,12.83)	0.67(0.03,15.64)	
ASV_1512	Firmicutes;D_2__Clostridia;D_3__Clostridiales;D_4__Christensenellaceae;D_5__Christensenellaceae R-7 group;D_6__Blattella germanica (German cockroach);D_7__;D_8__;D_9__;D_10__;D_11__;D_12__;D_13__;D_14__		1.13(0.05,23.48)	
ASV_1517	Firmicutes;D_2__Bacilli;D_3__Lactobacillales;D_4__Lactobacillaceae;D_5__Lactobacillus	0.33(0.02,5.36)	0.41(0.03,6.39)	
ASV_1520	Actinobacteria;D_2__Actinobacteria;D_3__Bifidobacteriales;D_4__Bifidobacteriaceae;D_5__Bifidobacterium	1.17(0.13,10.24)	0.87(0.10,7.54)	
ASV_1522	Firmicutes;D_2__Clostridia;D_3__Clostridiales;D_4__Peptococcaceae;D_5__uncultured;D_6__uncultured rumen bacterium;D_7__;D_8__;D_9__;D_10__;D_11__;D_12__;D_13__;D_14__	0.23(0.01,6.54)	0.32(0.01,8.46)	

ASV_1524	Bacteroidetes;D_2__Bacteroidia;D_3__Bacteroidales;D_4__Porphyromonadaceae;D_5__Porphyromonas;D_6__Porphyromonas asaccharolytica;D_7__;D_8__;D_9__;D_10__;D_11__;D_12__;D_13__;D_14__	75.81(2.24,2563.50);p=0.01598	86.49(2.43,3079.79);p=0.01441	↑
ASV_1526	Firmicutes;D_2__Clostridia;D_3__Clostridiales;D_4__Lachnospiraceae;D_5__uncultured;D_6__intestinal bacterium CG19-1;D_7__;D_8__;D_9__;D_10__;D_11__;D_12__;D_13__;D_14__	16.58(1.42,193.21);p=0.025		
ASV_1527	Firmicutes;D_2__Clostridia;D_3__Clostridiales;D_4__Ruminococcaceae;D_5__uncultured;D_6__uncultured bacterium;D_7__;D_8__;D_9__;D_10__;D_11__;D_12__;D_13__;D_14__	4.18(0.20,88.77)		
ASV_1530	Proteobacteria;D_2__Gammaproteobacteria;D_3__Betaproteobacteriales;D_4__Burkholderiaceae;D_5__Sutterella	0.20(0.01,3.78)		
ASV_1531	Firmicutes;D_2__Clostridia;D_3__Clostridiales;D_4__Ruminococcaceae;D_5__uncultured;D_6__Ruminococcaceae bacterium Marseille-P3738;D_7__;D_8__;D_9__;D_10__;D_11__;D_12__;D_13__;D_14__		26140412214293.30(0.00,Inf)	
ASV_1548	Proteobacteria;D_2__Alphaproteobacteria;D_3__Rhizobiales;D_4__Rhizobiaceae;D_5__Allorhizobium-Neorhizobium-Pararhizobium-Rhizobium	0.18(0.01,3.29)		
ASV_1552	Firmicutes;D_2__Clostridia;D_3__Clostridiales;D_4__Lachnospiraceae;D_5__Blautia	5.40(0.67,43.76)	1.96(0.27,14.46)	
ASV_1554	Bacteroidetes;D_2__Bacteroidia;D_3__Bacteroidales;D_4__Prevotellaceae;D_5__Prevotella 6;D_6__uncultured bacterium;D_7__;D_8__;D_9__;D_10__;D_11__;D_12__;D_13__;D_14__	39.03(1.35,1132.17);p=0.03295		
ASV_1556	Firmicutes;D_2__Clostridia;D_3__Clostridiales;D_4__Lachnospiraceae;D_5__Lachnoclostridium	23.58(0.74,747.20)	33.58(0.85,1319.92)	
ASV_1569	Firmicutes;D_2__Bacilli;D_3__Lactobacillales;D_4__Leuconostocaceae;D_5__Leuconostoc;D_6__uncultured bacterium;D_7__;D_8__;D_9__;D_10__;D_11__;D_12__;D_13__;D_14__	2.01(0.18,23.04)	6.37(0.54,75.19)	
ASV_1571	Firmicutes;D_2__Clostridia;D_3__Clostridiales;D_4__Lachnospiraceae;D_5__Sellimonas;D_6__uncultured bacterium;D_7__;D_8__;D_9__;D_10__;D_11__;D_12__;D_13__;D_14__	0.52(0.04,7.16)	0.52(0.04,6.58)	
ASV_1572	Firmicutes;D_2__Clostridia;D_3__Clostridiales;D_4__Lachnospiraceae;D_5__Lachnospira	1.42(0.04,55.82)		
ASV_1574	Bacteroidetes;D_2__Bacteroidia;D_3__Bacteroidales;D_4__Tannerellaceae;D_5__Parabacteroides	0.09(0.00,2.80)		
ASV_1586	Firmicutes;D_2__Clostridia;D_3__Clostridiales;D_4__Lachnospiraceae	0.06(0.00,3.39)		
ASV_1589	Firmicutes;D_2__Clostridia;D_3__Clostridiales;D_4__Ruminococcaceae;D_5__Flavonifractor	3.70(0.20,67.08)		
ASV_1639	Bacteroidetes;D_2__Bacteroidia;D_3__Bacteroidales;D_4__Muribaculaceae;D_5__Porphyromonadaceae bacterium C941;D_6__;D_7__;D_8__;D_9__;D_10__;D_11__;D_12__;D_13__;D_14__	0.65(0.02,28.46)		
ASV_1653	Bacteroidetes;D_2__Bacteroidia;D_3__Bacteroidales;D_4__Prevotellaceae;D_5__Prevotella;D_6__Chlamydia trachomatis;D_7__;D_8__;D_9__;D_10__;D_11__;D_12__;D_13__;D_14__	3.36(0.23,48.43)	1.98(0.15,25.66)	
ASV_1654	Actinobacteria;D_2__Actinobacteria;D_3__Bifidobacteriales;D_4__Bifidobacteriaceae;D_5__Gardnerella;D_6__uncultured bacterium;D_7__;D_8__;D_9__;D_10__;D_11__;D_12__;D_13__;D_14__	0.84(0.04,16.64)		
ASV_1659	Firmicutes;D_2__Clostridia;D_3__Clostridiales;D_4__Family XIII;D_5__Family XIII AD3011 group;D_6__metagenome;D_7__;D_8__;D_9__;D_10__;D_11__;D_12__;D_13__;D_14__	0.67(0.05,8.51)	0.26(0.02,3.25)	
ASV_1660	Firmicutes;D_2__Negativicutes;D_3__Selenomonadales;D_4__Veillonellaceae;D_5__Negativicoccus;D_6__uncultured bacterium;D_7__;D_8__;D_9__;D_10__;D_11__;D_12__;D_13__;D_14__	1.28(0.02,73.75)		
ASV_1661	Proteobacteria;D_2__Gammaproteobacteria;D_3__Betaproteobacteriales;D_4__Burkholderiaceae;D_5__Tepidimonas	0.45(0.02,9.83)		
ASV_1663	Bacteroidetes;D_2__Bacteroidia;D_3__Bacteroidales;D_4__Porphyromonadaceae;D_5__Porphyromonas	0.88(0.06,12.58)		
ASV_1714	Firmicutes;D_2__Clostridia;D_3__Clostridiales;D_4__Ruminococcaceae	0.93(0.08,11.08)		
ASV_1719	Actinobacteria;D_2__Actinobacteria;D_3__Micrococcales;D_4__Micrococcaceae;D_5__Rothia;D_6__uncultured bacterium;D_7__;D_8__;D_9__;D_10__;D_11__;D_12__;D_13__;D_14__	1.45(0.21,10.12)	1.29(0.19,8.80)	
ASV_1721	Actinobacteria;D_2__Coriobacteriia;D_3__Coriobacteriales;D_4__Atopobiaceae;D_5__Atopobium;D_6__uncultured bacterium;D_7__;D_8__;D_9__;D_10__;D_11__;D_12__;D_13__;D_14__	2.64(0.31,22.34)	3.00(0.36,25.35)	
ASV_1732	Firmicutes;D_2__Clostridia;D_3__Clostridiales;D_4__Lachnospiraceae	0.47(0.03,7.50)		
ASV_1736	Firmicutes;D_2__Clostridia;D_3__Clostridiales;D_4__Lachnospiraceae;D_5__Sellimonas;D_6__uncultured organism;D_7__;D_8__;D_9__;D_10__;D_11__;D_12__;D_13__;D_14__	6.17(0.38,100.42)		
ASV_1738	Firmicutes;D_2__Clostridia;D_3__Clostridiales;D_4__Lachnospiraceae;D_5__[Ruminococcus] gauvreauii group;D_6__uncultured bacterium;D_7__;D_8__;D_9__;D_10__;D_11__;D_12__;D_13__;D_14__	0.06(0.00,2.32)		
ASV_1741	Firmicutes;D_2__Clostridia;D_3__Clostridiales;D_4__Lachnospiraceae;D_5__Lachnoclostridium	0.51(0.01,20.10)	0.50(0.01,20.05)	
ASV_1748	Firmicutes;D_2__Clostridia;D_3__Clostridiales;D_4__Lachnospiraceae;D_5__Sellimonas	10.75(0.37,310.63)		
ASV_1751	Firmicutes;D_2__Clostridia;D_3__Clostridiales;D_4__Lachnospiraceae;D_5__[Eubacterium] hallii group;D_6__uncultured bacterium;D_7__;D_8__;D_9__;D_10__;D_11__;D_12__;D_13__;D_14__	7.65(0.25,230.05)	9.42(0.28,313.59)	

ASV_1756	Firmicutes;D_2__Clostridia;D_3__Clostridiales;D_4__Clostridiaceae 1;D_5__Clostridium sensu stricto 1	0.28(0.01,5.96)		
ASV_1761	Proteobacteria;D_2__Alphaproteobacteria;D_3__Caulobacterales;D_4__Caulobacteraceae;D_5__Phenylobacterium	1.43(0.09,22.80)	1.57(0.10,25.77)	
ASV_1777	Firmicutes;D_2__Clostridia;D_3__Clostridiales;D_4__Lachnospiraceae	1.93(0.08,48.61)		
ASV_1781	Firmicutes;D_2__Clostridia;D_3__Clostridiales;D_4__Lachnospiraceae;D_5__[Ruminococcus] torques group;D_6__uncultured organism;D_7__;D_8__;D_9__;D_10__;D_11__;D_12__;D_13__;D_14__	0.54(0.01,38.72)		
ASV_1782	Firmicutes;D_2__Clostridia;D_3__Clostridiales;D_4__Lachnospiraceae;D_5__Lachnoclostridium	0.42(0.01,15.47)		
ASV_1795	Firmicutes;D_2__Clostridia;D_3__Clostridiales;D_4__Christensenellaceae;D_5__Christensenellaceae R-7 group	0.17(0.00,6.23)	0.22(0.01,8.14)	
ASV_1801	Firmicutes;D_2__Clostridia;D_3__Clostridiales;D_4__Lachnospiraceae;D_5__Lachnospiraceae NC2004 group;D_6__uncultured bacterium;D_7__;D_8__;D_9__;D_10__;D_11__;D_12__;D_13__;D_14__	0.61(0.01,26.08)		
ASV_1818	Bacteroidetes;D_2__Bacteroidia;D_3__Bacteroidales;D_4__Bacteroidaceae;D_5__Bacteroides	0.23(0.01,4.12)	0.24(0.02,3.82)	
ASV_1820	Bacteroidetes;D_2__Bacteroidia;D_3__Bacteroidales;D_4__Bacteroidaceae;D_5__Bacteroides	1.19(0.04,38.29)		
ASV_1863	Firmicutes;D_2__Clostridia;D_3__Clostridiales;D_4__Ruminococcaceae;D_5__Ruminiclostridium 5;D_6__uncultured bacterium;D_7__;D_8__;D_9__;D_10__;D_11__;D_12__;D_13__;D_14__	0.88(0.10,7.44)	0.80(0.10,6.67)	
ASV_1869	Firmicutes;D_2__Clostridia;D_3__Clostridiales;D_4__Ruminococcaceae;D_5__Butyricicoccus;D_6__uncultured bacterium;D_7__;D_8__;D_9__;D_10__;D_11__;D_12__;D_13__;D_14__	2.78(0.15,51.73)		
ASV_1872	Firmicutes;D_2__Clostridia;D_3__Clostridiales;D_4__Peptococcaceae;D_5__uncultured;D_6__uncultured organism;D_7__;D_8__;D_9__;D_10__;D_11__;D_12__;D_13__;D_14__	1.28(0.03,57.50)		
ASV_1874	Firmicutes;D_2__Clostridia;D_3__Clostridiales;D_4__Lachnospiraceae;D_5__Lachnospiraceae UCG-010;D_6__uncultured bacterium;D_7__;D_8__;D_9__;D_10__;D_11__;D_12__;D_13__;D_14__	2.41(0.07,89.01)		
ASV_1876	Firmicutes;D_2__Clostridia;D_3__Clostridiales;D_4__Peptococcaceae;D_5__uncultured	0.88(0.02,38.62)		
ASV_1878	Firmicutes;D_2__Clostridia;D_3__Clostridiales;D_4__Ruminococcaceae;D_5__Subdoligranulum	1.61(0.09,28.32)		
ASV_1880	Actinobacteria;D_2__Coriobacteriia;D_3__Coriobacteriales;D_4__Coriobacteriaceae;D_5__Collinsella	0.69(0.04,11.32)	0.38(0.02,7.53)	
ASV_1895	Firmicutes;D_2__Clostridia;D_3__Clostridiales;D_4__Peptostreptococcaceae;D_5__Intestinibacter;D_6__uncultured bacterium;D_7__;D_8__;D_9__;D_10__;D_11__;D_12__;D_13__;D_14__	1.61(0.07,35.34)		
ASV_1896	Actinobacteria;D_2__Coriobacteriia;D_3__Coriobacteriales;D_4__Coriobacteriaceae;D_5__Collinsella	1.84(0.15,22.14)	2.07(0.17,25.31)	
ASV_1899	Firmicutes;D_2__Clostridia;D_3__Clostridiales;D_4__Lachnospiraceae;D_5__Tyzzerella;D_6__uncultured Firmicutes bacterium;D_7__;D_8__;D_9__;D_10__;D_11__;D_12__;D_13__;D_14__	0.16(0.01,3.68)		
ASV_1900	Bacteroidetes;D_2__Bacteroidia;D_3__Bacteroidales;D_4__Tannerellaceae;D_5__Parabacteroides	0.14(0.01,3.49)		
ASV_1989	Firmicutes;D_2__Clostridia;D_3__Clostridiales;D_4__Ruminococcaceae;D_5__Faecalibacterium	10.11(0.48,214.66)		
ASV_2054	Firmicutes;D_2__Clostridia;D_3__Clostridiales;D_4__Lachnospiraceae;D_5__Coprococcus 3		11633716720354.00(0.00,Inf)	
ASV_2058	Firmicutes;D_2__Clostridia;D_3__Clostridiales;D_4__Ruminococcaceae;D_5__Faecalibacterium;D_6__uncultured bacterium;D_7__;D_8__;D_9__;D_10__;D_11__;D_12__;D_13__;D_14__	0.85(0.02,30.56)		
ASV_2059	Firmicutes;D_2__Clostridia;D_3__Clostridiales;D_4__Lachnospiraceae	1.57(0.05,45.38)		
ASV_2067	Bacteroidetes;D_2__Bacteroidia;D_3__Bacteroidales;D_4__Bacteroidaceae;D_5__Bacteroides;D_6__uncultured bacterium;D_7__;D_8__;D_9__;D_10__;D_11__;D_12__;D_13__;D_14__	0.68(0.02,19.46)		
ASV_2071	Firmicutes;D_2__Clostridia;D_3__Clostridiales;D_4__Lachnospiraceae	0.49(0.01,16.97)		
ASV_2072	Firmicutes;D_2__Clostridia;D_3__Clostridiales;D_4__Clostridiaceae 1;D_5__Clostridium sensu stricto 1;D_6__uncultured bacterium;D_7__;D_8__;D_9__;D_10__;D_11__;D_12__;D_13__;D_14__	1.59(0.07,33.92)	0.91(0.05,17.92)	
ASV_2081	Firmicutes;D_2__Clostridia;D_3__Clostridiales;D_4__Lachnospiraceae;D_5__Agathobacter;D_6__uncultured bacterium;D_7__;D_8__;D_9__;D_10__;D_11__;D_12__;D_13__;D_14__	0.12(0.01,1.93)	0.08(0.00,1.48)	
ASV_2104	Firmicutes;D_2__Clostridia;D_3__Clostridiales;D_4__Lachnospiraceae;D_5__Lachnospiraceae UCG-004	4.26(0.21,85.77)	2.18(0.14,34.47)	
ASV_2109	Firmicutes;D_2__Clostridia;D_3__Clostridiales;D_4__Ruminococcaceae;D_5__Butyricicoccus;D_6__uncultured bacterium;D_7__;D_8__;D_9__;D_10__;D_11__;D_12__;D_13__;D_14__	0.79(0.04,13.90)		
ASV_2115	Firmicutes;D_2__Clostridia;D_3__Clostridiales;D_4__Lachnospiraceae;D_5__Blautia	0.13(0.00,3.81)	0.12(0.00,3.82)	

ASV_2116	Firmicutes;D_2__Clostridia;D_3__Clostridiales;D_4__Lachnospiraceae;D_5__Lactonifactor	0.53(0.03,8.82)		
ASV_2134	Firmicutes;D_2__Clostridia;D_3__Clostridiales;D_4__Lachnospiraceae;D_5__Oribacterium;D_6__Oribacterium sinus;D_7__;D_8__;D_9__;D_10__;D_11__;D_12__;D_13__;D_14__	1.30(0.13,12.54)		
ASV_2142	Actinobacteria;D_2__Actinobacteria;D_3__Corynebacteriales;D_4__Nocardiaceae;D_5__Rhodococcus		0.00(0.00,Inf)	
ASV_2149	Epsilonbacteraeota;D_2__Campylobacteria;D_3__Campylobacterales;D_4__Campylobacteraceae;D_5__Campylobacter	0.58(0.04,8.03)	0.38(0.03,5.59)	
ASV_2150	Firmicutes;D_2__Clostridia;D_3__Clostridiales;D_4__Lachnospiraceae;D_5__Blautia	0.78(0.02,29.08)	0.58(0.01,22.92)	
ASV_2162	Firmicutes;D_2__Clostridia;D_3__Clostridiales;D_4__Lachnospiraceae;D_5__Cuneatibacter;D_6__Cuneatibacter caecimuris;D_7__;D_8__;D_9__;D_10__;D_11__;D_12__;D_13__;D_14__	2.51(0.11,58.09)	5.19(0.17,155.85)	
ASV_2166	Firmicutes;D_2__Clostridia;D_3__Clostridiales;D_4__Lachnospiraceae		1030567417621.56(0.00,Inf)	
ASV_2167	Firmicutes;D_2__Clostridia;D_3__Clostridiales;D_4__Ruminococcaceae;D_5__Negativibacillus;D_6__uncultured bacterium;D_7__;D_8__;D_9__;D_10__;D_11__;D_12__;D_13__;D_14__	1.48(0.05,45.08)		
ASV_2185	Firmicutes;D_2__Clostridia;D_3__Clostridiales;D_4__Lachnospiraceae	0.41(0.02,9.82)		
ASV_2190	Bacteroidetes;D_2__Bacteroidia;D_3__Bacteroidales;D_4__Bacteroidaceae;D_5__Bacteroides	0.39(0.01,20.20)		
ASV_2195	Firmicutes;D_2__Clostridia;D_3__Clostridiales;D_4__Ruminococcaceae;D_5__Subdoligranulum	0.12(0.00,3.37)		
ASV_2219	Firmicutes;D_2__Bacilli;D_3__Bacillales;D_4__Alicyclobacillaceae;D_5__Effusibacillus;D_6__uncultured organism;D_7__;D_8__;D_9__;D_10__;D_11__;D_12__;D_13__;D_14__	0.20(0.01,8.18)		
ASV_2236	Firmicutes;D_2__Clostridia;D_3__Clostridiales;D_4__Lachnospiraceae;D_5__Lachnospiraceae NC2004 group;D_6__uncultured bacterium;D_7__;D_8__;D_9__;D_10__;D_11__;D_12__;D_13__;D_14__	0.36(0.02,5.85)		
ASV_2239	Firmicutes;D_2__Clostridia;D_3__Clostridiales;D_4__Ruminococcaceae	0.70(0.01,36.28)		
ASV_2240	Firmicutes;D_2__Clostridia;D_3__Clostridiales;D_4__Ruminococcaceae;D_5__Ruminiclostridium;D_6__metagenome;D_7__;D_8__;D_9__;D_10__;D_11__;D_12__;D_13__;D_14__	0.74(0.05,11.41)	0.25(0.01,5.24)	
ASV_2252	Firmicutes;D_2__Clostridia;D_3__Clostridiales;D_4__Lachnospiraceae;D_5__Agathobacter;D_6__uncultured bacterium;D_7__;D_8__;D_9__;D_10__;D_11__;D_12__;D_13__;D_14__		36938116459108.90(0.00,Inf)	
ASV_2261	Firmicutes;D_2__Clostridia;D_3__Clostridiales;D_4__Christensenellaceae;D_5__Christensenellaceae R-7 group		1030567417621.56(0.00,Inf)	
ASV_2263	Firmicutes;D_2__Clostridia;D_3__Clostridiales;D_4__Peptococcaceae;D_5__uncultured;D_6__uncultured organism;D_7__;D_8__;D_9__;D_10__;D_11__;D_12__;D_13__;D_14__		1030567417621.56(0.00,Inf)	
ASV_2264	Firmicutes;D_2__Clostridia;D_3__Clostridiales;D_4__Ruminococcaceae;D_5__Fastidiosipila;D_6__Clostridiales bacterium oral clone MCE3_9;D_7__;D_8__;D_9__;D_10__;D_11__;D_12__;D_13__;D_14__		1030567417621.56(0.00,Inf)	
ASV_2265	Firmicutes;D_2__Clostridia;D_3__Clostridiales;D_4__Lachnospiraceae;D_5__[Ruminococcus] torques group;D_6__Ruminococcus sp. AT10;D_7__;D_8__;D_9__;D_10__;D_11__;D_12__;D_13__;D_14__	1.89(0.18,20.35)		
ASV_2273	Firmicutes;D_2__Clostridia;D_3__Clostridiales;D_4__Ruminococcaceae;D_5__uncultured;D_6__uncultured bacterium;D_7__;D_8__;D_9__;D_10__;D_11__;D_12__;D_13__;D_14__		1.62(0.09,28.71)	
ASV_2282	Firmicutes;D_2__Clostridia;D_3__Clostridiales;D_4__Ruminococcaceae;D_5__Ruminococcaceae UCG-005;D_6__uncultured organism;D_7__;D_8__;D_9__;D_10__;D_11__;D_12__;D_13__;D_14__	0.01(0.00,0.87);p=0.04288		
ASV_2300	Firmicutes;D_2__Clostridia;D_3__Clostridiales;D_4__Clostridiales vadinBB60 group;D_5__uncultured bacterium;D_6__;D_7__;D_8__;D_9__;D_10__;D_11__;D_12__;D_13__;D_14__	0.12(0.01,2.44)		
ASV_2307	Proteobacteria;D_2__Gammaproteobacteria;D_3__Enterobacteriales;D_4__Enterobacteriaceae	8.28(0.26,268.38)		
ASV_2343	Bacteroidetes;D_2__Bacteroidia;D_3__Bacteroidales;D_4__Bacteroidaceae;D_5__Bacteroides	1.06(0.03,40.50)		
ASV_2418	Firmicutes;D_2__Clostridia;D_3__Clostridiales;D_4__Lachnospiraceae;D_5__Lachnospiraceae UCG-001	0.08(0.00,4.19)		
ASV_2460	Firmicutes;D_2__Clostridia;D_3__Clostridiales;D_4__Ruminococcaceae;D_5__Ruminiclostridium 5;D_6__gut metagenome;D_7__;D_8__;D_9__;D_10__;D_11__;D_12__;D_13__;D_14__	0.09(0.00,4.64)		
ASV_2480	Firmicutes;D_2__Clostridia;D_3__Clostridiales;D_4__Ruminococcaceae;D_5__Flavonifractor;D_6__uncultured bacterium;D_7__;D_8__;D_9__;D_10__;D_11__;D_12__;D_13__;D_14__	8.07(0.26,250.31)		
ASV_2488	Firmicutes;D_2__Clostridia;D_3__Clostridiales;D_4__Ruminococcaceae;D_5__Flavonifractor;D_6__uncultured bacterium;D_7__;D_8__;D_9__;D_10__;D_11__;D_12__;D_13__;D_14__	0.98(0.06,16.44)		
ASV_2501	Firmicutes;D_2__Clostridia;D_3__Clostridiales	1.32(0.04,40.43)		
ASV_2557	Firmicutes;D_2__Clostridia;D_3__Clostridiales;D_4__Ruminococcaceae;D_5__Ruminococcaceae UCG-013	2.71(0.12,62.71)	4.15(0.16,107.11)	

ASV_2609	Firmicutes;D_2__Clostridia;D_3__Clostridiales;D_4__Family XI;D_5__Peptoniphilus;D_6__Peptoniphilus coxii;D_7__;D_8__;D_9__;D_10__;D_11__;D_12__;D_13__;D_14__	1.36(0.03,69.79)		
ASV_2611	Firmicutes;D_2__Clostridia;D_3__Clostridiales;D_4__Lachnospiraceae;D_5__[Ruminococcus] gauvreauii group;D_6__Ruminococcus gauvreauii;D_7__;D_8__;D_9__;D_10__;D_11__;D_12__;D_13__;D_14__	1.06(0.03,38.84)		
ASV_2665	Patescibacteria;D_2__Saccharimonadia;D_3__Saccharimonadales;D_4__TM7 phylum sp. oral clone FR058;D_5__;D_6__;D_7__;D_8__;D_9__;D_10__;D_11__;D_12__;D_13__;D_14__	0.60(0.03,13.64)	1.01(0.04,26.92)	
ASV_2687	Actinobacteria;D_2__Actinobacteria;D_3__Actinomycetales;D_4__Actinomycetaceae;D_5__Actinomyces	0.68(0.03,13.28)	0.54(0.02,12.39)	
ASV_2933	Firmicutes;D_2__Clostridia;D_3__Clostridiales;D_4__Lachnospiraceae;D_5__Fusicatenibacter;D_6__uncultured bacterium;D_7__;D_8__;D_9__;D_10__;D_11__;D_12__;D_13__;D_14__	0.20(0.00,13.84)		
ASV_3357	Firmicutes;D_2__Clostridia;D_3__Clostridiales;D_4__Ruminococcaceae;D_5__uncultured	0.00(0.00,Inf)	0.00(0.00,Inf)	

Table 7.2. Species-level differences in the gut microbial communities by individual amplicon sequence variants (ASVs) for USA-only cohort participants with: pediatric-onset multiple sclerosis (MS) [disease-modifying drug (DMD) exposed and naïve], and unaffected controls, expressed as rate ratios

		Rate ratios (95%CIs) unadjusted			Rate ratios (95%CIs) age and sex adjusted					
		Ref.: MS cases DMD-	Ref.: Controls		Ref.: MS cases DMD-	Ref.: Controls				
Taxa Identifier (for internal use)	Species-level findings (shown as: Phylum;Class;Order;Family;Genus;Species) i.e., D_6= species	MS cases DMD+	MS cases DMD-	MS cases DMD+	MS cases DMD+	MS cases DMD-	MS cases DMD+	MS DMD+ (vs MS DMD-)	*MS DMD- (vs control)*	*MS DMD+ (vs control)*
ASV_3	Firmicutes;D_2__Bacilli;D_3__Lactobacillales;D_4__Streptococcaceae;D_5__Streptococcus;D_6__Streptococcus salivarius subsp. thermophilus;D_7__;D_8__;D_9__;D_10__;D_11__;D_12__;D_13__;D_14__	1.53(0.70,3.31)	0.59(0.28,1.24)	0.90(0.48,1.66)	1.79(0.85,3.79)	0.71(0.35,1.44)	1.26(0.70,2.29)			
ASV_4	Firmicutes;D_2__Clostridia;D_3__Clostridiales;D_4__Lachnospiraceae;D_5__Blautia	1.52(0.74,3.15)	1.37(0.68,2.74)	2.08(1.17,3.70);p=0.01252	1.55(0.74,3.22)	1.37(0.68,2.75)	2.12(1.19,3.78);p=0.01126			↑
ASV_5	Firmicutes;D_2__Clostridia;D_3__Clostridiales;D_4__Lachnospiraceae;D_5__Blautia	1.12(0.60,2.10)	1.40(0.77,2.56)	1.57(0.96,2.59)	1.08(0.58,2.02)	1.51(0.83,2.74)	1.62(0.99,2.67)			
ASV_7	Firmicutes;D_2__Clostridia;D_3__Clostridiales;D_4__Lachnospiraceae;D_5__Blautia	1.66(0.91,3.00)	0.72(0.40,1.27)	1.19(0.74,1.90)	1.64(0.90,2.99)	0.73(0.41,1.29)	1.19(0.74,1.91)			
ASV_8	Firmicutes;D_2__Clostridia;D_3__Clostridiales;D_4__Lachnospiraceae;D_5__Coprococcus 3	1.45(0.57,3.66)	0.60(0.25,1.47)	0.87(0.42,1.82)	1.36(0.53,3.45)	0.63(0.26,1.53)	0.85(0.41,1.79)			
ASV_9	Firmicutes;D_2__Clostridia;D_3__Clostridiales;D_4__Lachnospiraceae;D_5__Anaerostipes	1.40(0.69,2.83)	0.89(0.45,1.76)	1.24(0.71,2.18)	1.35(0.66,2.74)	1.00(0.51,1.97)	1.35(0.77,2.36)			
ASV_11	Firmicutes;D_2__Clostridia;D_3__Clostridiales;D_4__Lachnospiraceae;D_5__Dorea	1.16(0.55,2.45)	0.87(0.42,1.79)	1.01(0.56,1.82)	1.17(0.55,2.49)	0.90(0.44,1.84)	1.05(0.58,1.91)			
ASV_13	Firmicutes;D_2__Bacilli;D_3__Lactobacillales;D_4__Streptococcaceae;D_5__Streptococcus	1.87(0.82,4.25)	1.41(0.64,3.11)	2.63(1.37,5.05);p=0.00378	2.15(0.94,4.92)	1.21(0.55,2.68)	2.61(1.35,5.02);p=0.00418			↑
ASV_14	Firmicutes;D_2__Clostridia;D_3__Clostridiales;D_4__Lachnospiraceae;D_5__Blautia	0.85(0.41,1.76)	1.20(0.59,2.42)	1.02(0.57,1.82)	0.82(0.40,1.66)	1.12(0.57,2.19)	0.91(0.52,1.60)			
ASV_15	Firmicutes;D_2__Clostridia;D_3__Clostridiales;D_4__Lachnospiraceae;D_5__[Eubacterium] hallii group	2.61(1.02,6.70);p=0.0461	0.64(0.26,1.58)	1.67(0.79,3.53)	2.55(0.99,6.61)	0.63(0.26,1.57)	1.61(0.76,3.43)			
ASV_16	Firmicutes;D_2__Clostridia;D_3__Clostridiales;D_4__Lachnospiraceae;D_5__Coprococcus 1	0.94(0.34,2.59)	0.67(0.25,1.76)	0.63(0.28,1.40)	0.90(0.33,2.49)	0.75(0.28,1.96)	0.67(0.30,1.51)			
ASV_17	Firmicutes;D_2__Clostridia;D_3__Clostridiales;D_4__Ruminococcaceae;D_5__Ruminococcaceae UCG-004;D_6__uncultured bacterium;D_7__;D_8__;D_9__;D_10__;D_11__;D_12__;D_13__;D_14__	1.77(0.51,6.15)	0.52(0.16,1.73)	0.92(0.34,2.48)	1.70(0.48,5.94)	0.52(0.16,1.73)	0.89(0.33,2.40)			

ASV_20	Actinobacteria;D_2__Actinobacteria;D_3__Bifidobacteriales;D_4__Bifidobacteriaceae;D_5__Bifidobacterium	7.81(1.18,51.86);p=0.03341	0.27(0.04,1.67)	2.11(0.47,9.50)						
ASV_24	Bacteroidetes;D_2__Bacteroidia;D_3__Bacteroidales;D_4__Bacteroidaceae;D_5__Bacteroides	3.66(0.74,18.25)	0.36(0.08,1.68)	1.31(0.37,4.70)	3.38(0.69,16.56)	0.52(0.11,2.36)	1.75(0.50,6.17)			
ASV_25	Firmicutes;D_2__Clostridia;D_3__Clostridiales;D_4__Lachnospiraceae;D_5__Blautia	1.07(0.26,4.35)	1.80(0.47,6.97)	1.93(0.63,5.88)	1.37(0.34,5.57)	1.52(0.40,5.79)	2.08(0.68,6.32)			
ASV_26	Firmicutes;D_2__Clostridia;D_3__Clostridiales;D_4__Ruminococcaceae;D_5__Ruminiclostridium 5	4.55(1.32,15.70);p=0.0166	0.44(0.13,1.45)	2.00(0.75,5.34)	4.70(1.35,16.44);p=0.01531	0.45(0.14,1.47)	2.10(0.78,5.66)	↑		
ASV_27	Actinobacteria;D_2__Coriobacteriia;D_3__Coriobacteriales;D_4__Coriobacteriaceae;D_5__Collinsella	1.82(0.63,5.27)	0.78(0.28,2.18)	1.42(0.61,3.31)	1.83(0.63,5.35)	0.74(0.26,2.05)	1.35(0.58,3.15)			
ASV_28	Firmicutes;D_2__Clostridia;D_3__Clostridiales;D_4__Lachnospiraceae;D_5__uncultured;D_6__uncultured Eubacterium sp.;D_7__;D_8__;D_9__;D_10__;D_11__;D_12__;D_13__;D_14__	1.58(0.48,5.15)	0.85(0.27,2.65)	1.34(0.52,3.42)	1.33(0.41,4.35)	0.96(0.31,2.96)	1.28(0.50,3.25)			
ASV_29	Firmicutes;D_2__Clostridia;D_3__Clostridiales;D_4__Ruminococcaceae;D_5__Ruminococcaceae NK4A214 group;D_6__gut metagenome;D_7__;D_8__;D_9__;D_10__;D_11__;D_12__;D_13__;D_14__	2.02(0.42,9.70)	0.23(0.05,1.03)	0.46(0.13,1.59)	3.37(0.71,16.04)	0.18(0.04,0.78);p=0.0223	0.60(0.17,2.05)		↓	
ASV_31	Firmicutes;D_2__Clostridia;D_3__Clostridiales;D_4__Ruminococcaceae;D_5__[Eubacterium] coprostanoligenes group;D_6__uncultured organism;D_7__;D_8__;D_9__;D_10__;D_11__;D_12__;D_13__;D_14__				1.11(0.08,15.92)	0.55(0.04,6.97)	0.61(0.07,5.02)			
ASV_32	Firmicutes;D_2__Clostridia;D_3__Clostridiales;D_4__Christensenellaceae;D_5__Christensenellaceae R-7 group	7.39(0.43,128.47)	0.80(0.05,12.45)	5.90(0.61,56.70)	5.60(0.33,96.27)	1.06(0.07,15.99)	5.95(0.63,56.42)			
ASV_33	Bacteroidetes;D_2__Bacteroidia;D_3__Bacteroidales;D_4__Rikenellaceae;D_5__Alistipes;D_6__Alistipes indistinctus YIT 12060;D_7__;D_8__;D_9__;D_10__;D_11__;D_12__;D_13__;D_14__	0.49(0.07,3.35)	3.46(0.54,22.08)	1.69(0.37,7.81)	0.56(0.08,3.87)	3.23(0.51,20.43)	1.81(0.39,8.37)			
ASV_34	Firmicutes;D_2__Negativicutes;D_3__Selenomonadales;D_4__Veillonellaceae;D_5__Dialister	0.14(0.02,0.84);p=0.03134	5.87(1.03,33.38);p=0.0459	0.81(0.19,3.39)	0.14(0.02,0.85);p=0.03233	6.17(1.09,34.87);p=0.03943	0.85(0.20,3.58)	↓	↑	
ASV_36	Bacteroidetes;D_2__Bacteroidia;D_3__Bacteroidales;D_4__Bacteroidaceae;D_5__Bacteroides;D_6__Bacteroides thetaiotaomicron;D_7__;D_8__;D_9__;D_10__;D_11__;D_12__;D_13__;D_14__	1.18(0.37,3.69)	0.89(0.30,2.66)	1.04(0.42,2.58)	1.19(0.38,3.77)	0.83(0.28,2.49)	0.98(0.39,2.46)			
ASV_37	Firmicutes;D_2__Clostridia;D_3__Clostridiales;D_4__Lachnospiraceae;D_5__[Ruminococcus] torques group;D_6__uncultured organism;D_7__;D_8__;D_9__;D_10__;D_11__;D_12__;D_13__;D_14__	3.07(0.96,9.77)	0.28(0.09,0.84);p=0.02331	0.84(0.34,2.12)	1.77(0.56,5.56)	0.39(0.13,1.17)	0.69(0.28,1.72)			
ASV_38	Firmicutes;D_2__Clostridia;D_3__Clostridiales;D_4__Ruminococcaceae;D_5__Ruminococcus 1;D_6__uncultured organism;D_7__;D_8__;D_9__;D_10__;D_11__;D_12__;D_13__;D_14__	1.83(0.42,8.04)	0.32(0.08,1.32)	0.58(0.18,1.88)	1.75(0.40,7.76)	0.33(0.08,1.34)	0.57(0.18,1.86)			
ASV_39	Bacteroidetes;D_2__Bacteroidia;D_3__Bacteroidales;D_4__Rikenellaceae;D_5__Alistipes;D_6__uncultured organism;D_7__;D_8__;D_9__;D_10__;D_11__;D_12__;D_13__;D_14__	1.08(0.23,5.08)	0.75(0.17,3.32)	0.81(0.24,2.77)	1.34(0.29,6.33)	0.80(0.18,3.51)	1.08(0.32,3.68)			
ASV_40	Firmicutes;D_2__Clostridia;D_3__Clostridiales;D_4__Lachnospiraceae;D_5__[Eubacterium] hallii group	2.13(0.21,21.23)	0.57(0.06,5.19)	1.21(0.20,7.51)	3.69(0.39,34.69)	0.55(0.07,4.70)	2.05(0.35,12.06)			
ASV_41	Firmicutes;D_2__Clostridia;D_3__Clostridiales;D_4__Lachnospiraceae;D_5__Marvinbryantia	4.90(0.59,40.62)	0.17(0.02,1.27)	0.82(0.15,4.32)	5.35(0.66,43.72)	0.09(0.01,0.67);p=0.01843	0.48(0.09,2.45)		↓	
ASV_43	Firmicutes;D_2__Clostridia;D_3__Clostridiales;D_4__Ruminococcaceae;D_5__Ruminococcaceae UCG-005;D_6__uncultured organism;D_7__;D_8__;D_9__;D_10__;D_11__;D_12__;D_13__;D_14__	0.85(0.16,4.41)	1.39(0.29,6.78)	1.18(0.32,4.37)	0.96(0.19,4.88)	1.29(0.27,6.11)	1.24(0.34,4.49)			
ASV_46	Firmicutes;D_2__Clostridia;D_3__Clostridiales;D_4__Lachnospiraceae;D_5__Agathobacter	1.35(0.59,3.08)	1.21(0.55,2.67)	1.63(0.85,3.14)	1.34(0.58,3.06)	1.23(0.56,2.70)	1.64(0.85,3.16)			
ASV_47	Firmicutes;D_2__Clostridia;D_3__Clostridiales;D_4__Ruminococcaceae;D_5__Faecalibacterium	0.68(0.36,1.31)	1.53(0.82,2.86)	1.05(0.62,1.76)	0.66(0.34,1.28)	1.55(0.83,2.91)	1.03(0.61,1.74)			
ASV_48	Firmicutes;D_2__Clostridia;D_3__Clostridiales;D_4__Ruminococcaceae;D_5__Intestinimonas;D_6__uncultured bacterium;D_7__;D_8__;D_9__;D_10__;D_11__;D_12__;D_13__;D_14__	3.20(0.32,32.53)	0.26(0.03,2.42)	0.83(0.13,5.22)						
ASV_50	Firmicutes;D_2__Clostridia;D_3__Clostridiales;D_4__Lachnospiraceae;D_5__[Ruminococcus] gauvreauii group	0.93(0.31,2.82)	0.61(0.21,1.78)	0.57(0.24,1.38)	1.01(0.33,3.07)	0.62(0.21,1.79)	0.62(0.26,1.50)			
ASV_51	Firmicutes;D_2__Clostridia;D_3__Clostridiales;D_4__Ruminococcaceae;D_5__Subdoligranulum	1.08(0.47,2.45)	0.71(0.32,1.56)	0.76(0.40,1.46)	1.08(0.47,2.47)	0.70(0.32,1.55)	0.76(0.39,1.47)			
ASV_52	Firmicutes;D_2__Clostridia;D_3__Clostridiales;D_4__Ruminococcaceae;D_5__Ruminiclostridium 5	36.52(3.02,440.94);p=0.00464	0.05(0.00,0.59);p=0.01675	1.96(0.27,14.09)	35.89(3.28,393.15);p=0.00337	0.04(0.00,0.35);p=0.00423	1.27(0.21,7.78)	↑	↓	
ASV_53	Firmicutes;D_2__Clostridia;D_3__Clostridiales;D_4__Lachnospiraceae;D_5__[Ruminococcus] torques group	1.02(0.35,2.99)	1.27(0.45,3.59)	1.30(0.55,3.05)	0.98(0.33,2.91)	1.30(0.46,3.67)	1.28(0.54,3.02)			

ASV_56	Firmicutes;D_2__Bacilli;D_3__Lactobacillales;D_4__Leuconostocaceae;D_5__Weissella	1.20(0.05,26.08)	4.15(0.21,80.75)	4.97(0.43,57.68)						
ASV_58	Bacteroidetes;D_2__Bacteroidia;D_3__Bacteroidales;D_4__Rikenellaceae;D_5__Alistipes	1.20(0.37,3.89)	1.30(0.42,4.01)	1.56(0.61,3.96)						
ASV_60	Firmicutes;D_2__Clostridia;D_3__Clostridiales;D_4__Lachnospiraceae;D_5__Blautia; D_6__uncultured Blautia sp.;D_7__;D_8__;D_9__;D_10__;D_11__;D_12__;D_13__;D_14__	1.33(0.40,4.38)	0.32(0.10,1.00)	0.42(0.16,1.09)	1.55(0.47,5.07)	0.45(0.14,1.39)	0.69(0.27,1.77)			
ASV_62	Firmicutes;D_2__Clostridia;D_3__Clostridiales;D_4__Lachnospiraceae;D_5__Agathobacter;D_6__Eubacterium ramulus;D_7__;D_8__;D_9__;D_10__;D_11__;D_12__;D_13__;D_14__	0.63(0.21,1.85)	0.73(0.26,2.04)	0.46(0.19,1.08)	0.59(0.20,1.75)	0.76(0.27,2.13)	0.45(0.19,1.06)			
ASV_65	Firmicutes;D_2__Clostridia;D_3__Clostridiales;D_4__Ruminococcaceae;D_5__uncultured;D_6__uncultured Clostridium sp.;D_7__;D_8__;D_9__;D_10__;D_11__;D_12__;D_13__;D_14__	4.73(0.77,28.92)	0.15(0.03,0.87);p=0.03398	0.72(0.18,2.95)	4.25(0.69,26.31)	0.12(0.02,0.70);p=0.01789	0.52(0.13,2.13)		↓	
ASV_66	Firmicutes;D_2__Clostridia;D_3__Clostridiales;D_4__Ruminococcaceae;D_5__Subdoligranulum	0.79(0.29,2.16)	0.78(0.30,2.05)	0.62(0.28,1.37)	0.86(0.31,2.38)	0.76(0.29,2.01)	0.66(0.30,1.47)			
ASV_68	Firmicutes;D_2__Clostridia;D_3__Clostridiales;D_4__Family XIII;D_5__Family XIII AD3011 group;D_6__uncultured Eubacterium sp.;D_7__;D_8__;D_9__;D_10__;D_11__;D_12__;D_13__;D_14__	6.15(1.16,32.56);p=0.03253	0.07(0.01,0.35);p=0.00126	0.44(0.13,1.51)	5.57(1.05,29.59);p=0.04384	0.08(0.02,0.41);p=0.00219	0.46(0.13,1.60)	↑	↓	
ASV_69	Bacteroidetes;D_2__Bacteroidia;D_3__Bacteroidales;D_4__Rikenellaceae;D_5__Alistipes;D_6__Alistipes sp. N15.MGS-157;D_7__;D_8__;D_9__;D_10__;D_11__;D_12__;D_13__;D_14__	94.20(10.32,859.64);p=0.00006	0.01(0.00,0.09);p=0.00003	0.99(0.19,5.09)	111.82(11.97,1044.37);p=0.00004;q=0.03345	0.01(0.00,0.10);p=0.00005;q=0.03345	1.35(0.26,6.93)	↑*	↓*	
ASV_70	Firmicutes;D_2__Bacilli;D_3__Lactobacillales;D_4__Streptococcaceae;D_5__Streptococcus;D_6__Streptococcus anginosus subsp. anginosus;D_7__;D_8__;D_9__;D_10__;D_11__;D_12__;D_13__;D_14__	5.44(0.64,46.18)	0.10(0.01,0.81);p=0.03111	0.56(0.11,2.87)	4.18(0.49,35.57)	0.15(0.02,1.20)	0.64(0.13,3.25)			
ASV_72	Firmicutes;D_2__Clostridia;D_3__Clostridiales;D_4__Lachnospiraceae;D_5__Eisenbergiella;D_6__uncultured organism;D_7__;D_8__;D_9__;D_10__;D_11__;D_12__;D_13__;D_14__	0.70(0.20,2.52)	1.29(0.38,4.39)	0.91(0.33,2.50)						
ASV_73	Firmicutes;D_2__Bacilli;D_3__Lactobacillales;D_4__Streptococcaceae;D_5__Streptococcus	1.19(0.45,3.11)	1.96(0.77,4.98)	2.33(1.08,5.02);p=0.03106	1.28(0.48,3.37)	1.73(0.68,4.37)	2.21(1.02,4.76);p=0.04308			↑
ASV_74	Firmicutes;D_2__Bacilli;D_3__Bacillales;D_4__Family XI;D_5__Gemella	1.69(0.51,5.62)	0.85(0.27,2.72)	1.44(0.56,3.71)	1.70(0.51,5.68)	0.80(0.25,2.54)	1.36(0.53,3.50)			
ASV_75	Firmicutes;D_2__Clostridia;D_3__Clostridiales;D_4__Lachnospiraceae;D_5__Fusicatenibacter	1.80(0.91,3.57)	0.63(0.33,1.22)	1.13(0.66,1.95)	1.75(0.88,3.48)	0.65(0.34,1.25)	1.14(0.66,1.96)			
ASV_80	Firmicutes;D_2__Clostridia;D_3__Clostridiales;D_4__Peptostreptococcaceae;D_5__Romboutsia	0.98(0.42,2.28)	1.52(0.67,3.44)	1.49(0.76,2.91)	1.05(0.45,2.47)	1.59(0.70,3.58)	1.67(0.85,3.28)			
ASV_83	Firmicutes;D_2__Clostridia;D_3__Clostridiales;D_4__Lachnospiraceae;D_5__Roseburia;D_6__gut metagenome;D_7__;D_8__;D_9__;D_10__;D_11__;D_12__;D_13__;D_14__	0.99(0.24,4.09)	0.59(0.15,2.30)	0.59(0.19,1.80)	1.14(0.28,4.61)	0.57(0.15,2.15)	0.65(0.22,1.96)			
ASV_85	Firmicutes;D_2__Clostridia;D_3__Clostridiales;D_4__Ruminococcaceae;D_5__Ruminococcaceae UCG-002;D_6__uncultured rumen bacterium;D_7__;D_8__;D_9__;D_10__;D_11__;D_12__;D_13__;D_14__	0.36(0.05,2.54)	0.48(0.07,3.16)	0.17(0.04,0.82);p=0.02701	0.31(0.04,2.22)	0.53(0.08,3.47)	0.17(0.04,0.79);p=0.02401			↓
ASV_86	Firmicutes;D_2__Clostridia;D_3__Clostridiales;D_4__Ruminococcaceae;D_5__Ruminococcaceae UCG-005;D_6__human gut metagenome;D_7__;D_8__;D_9__;D_10__;D_11__;D_12__;D_13__;D_14__	1.40(0.37,5.39)	0.33(0.09,1.21)	0.46(0.16,1.35)	1.18(0.31,4.46)	0.36(0.10,1.30)	0.43(0.15,1.23)			
ASV_87	Firmicutes;D_2__Clostridia;D_3__Clostridiales;D_4__Ruminococcaceae;D_5__UBA1819;D_6__uncultured organism;D_7__;D_8__;D_9__;D_10__;D_11__;D_12__;D_13__;D_14__	2.52(0.99,6.39)	0.34(0.14,0.85);p=0.01994	0.87(0.42,1.82)	2.27(0.89,5.78)	0.37(0.15,0.89);p=0.02741	0.83(0.40,1.74)		↓	
ASV_88	Firmicutes;D_2__Erysipelotrichia;D_3__Erysipelotrichales;D_4__Erysipelotrichaceae; D_5__[Clostridium] innocuum group	1.17(0.36,3.80)	1.90(0.61,5.91)	2.22(0.87,5.66)	1.13(0.35,3.69)	2.05(0.66,6.34)	2.32(0.91,5.93)			
ASV_89	Firmicutes;D_2__Clostridia;D_3__Clostridiales;D_4__Family XIII;D_5__Family XIII AD3011 group;D_6__uncultured bacterium;D_7__;D_8__;D_9__;D_10__;D_11__;D_12__;D_13__;D_14__	1.28(0.45,3.64)	0.51(0.19,1.40)	0.65(0.29,1.50)	1.20(0.43,3.38)	0.74(0.28,1.97)	0.89(0.39,2.00)			
ASV_93	Firmicutes;D_2__Clostridia;D_3__Clostridiales;D_4__Lachnospiraceae;D_5__Roseburia;D_6__metagenome;D_7__;D_8__;D_9__;D_10__;D_11__;D_12__;D_13__;D_14__	0.54(0.05,5.29)	1.65(0.18,14.86)	0.89(0.14,5.45)						
ASV_95	Bacteroidetes;D_2__Bacteroidia;D_3__Bacteroidales;D_4__Rikenellaceae;D_5__Alistipes	2.28(0.54,9.63)	0.49(0.12,1.96)	1.12(0.36,3.51)	2.26(0.53,9.64)	0.50(0.12,1.98)	1.12(0.36,3.54)			
ASV_96	Firmicutes;D_2__Clostridia;D_3__Clostridiales;D_4__Lachnospiraceae;D_5__Hungatella	12.77(1.20,136.34);p=0.03499	0.13(0.01,1.28)	1.67(0.27,10.37)	26.08(2.35,289.11);p=0.00788	0.09(0.01,0.93);p=0.04342	2.40(0.44,12.99)	↑	↓	

ASV_98	Firmicutes;D_2__Clostridia;D_3__Clostridiales;D_4__Lachnospiraceae;D_5__Lachnospiraceae FCS020 group;D_6__uncultured Clostridium sp.;D_7__;D_8__;D_9__;D_10__;D_11__;D_12__;D_13__;D_14__	2.03(0.45,9.16)	0.39(0.09,1.65)	0.79(0.24,2.61)	1.82(0.41,8.02)	0.31(0.07,1.26)	0.56(0.17,1.80)			
ASV_99	Firmicutes;D_2__Clostridia;D_3__Clostridiales;D_4__Lachnospiraceae;D_5__Hungatella;D_6__uncultured bacterium;D_7__;D_8__;D_9__;D_10__;D_11__;D_12__;D_13__;D_14__	1.60(0.16,16.54)	0.09(0.01,0.84);p=0.03435	0.14(0.02,0.90);p=0.03868						
ASV_100	Firmicutes;D_2__Clostridia;D_3__Clostridiales;D_4__Family XIII;D_5__Family XIII AD3011 group	4.66(0.34,64.43)	0.79(0.06,9.96)	3.70(0.46,29.60)	7.56(0.59,96.25)	0.60(0.05,6.87)	4.54(0.63,32.86)			
ASV_101	Firmicutes;D_2__Clostridia;D_3__Clostridiales;D_4__Lachnospiraceae;D_5__Dorea	1.45(0.73,2.87)	0.73(0.38,1.42)	1.06(0.62,1.83)	1.35(0.68,2.69)	0.75(0.39,1.45)	1.02(0.59,1.75)			
ASV_102	Firmicutes;D_2__Negativicutes;D_3__Selenomonadales;D_4__Veillonellaceae;D_5__Veillonella	0.24(0.07,0.83);p=0.02412	1.98(0.61,6.43)	0.48(0.18,1.28)	0.68(0.20,2.26)	0.77(0.25,2.43)	0.52(0.20,1.36)			
ASV_104	Bacteroidetes;D_2__Bacteroidia;D_3__Bacteroidales;D_4__Marinifilaceae;D_5__Odoribacter	1.73(0.48,6.24)	0.72(0.21,2.48)	1.26(0.45,3.47)						
ASV_105	Firmicutes;D_2__Bacilli;D_3__Lactobacillales;D_4__Streptococcaceae;D_5__Streptococcus	42.18(1.80,987.03);p=0.02	0.10(0.00,2.25)	4.38(0.51,37.45)	58.39(2.23,1529.74);p=0.01465	0.12(0.00,2.95)	7.01(0.88,55.59)	↑		
ASV_106	Firmicutes;D_2__Clostridia;D_3__Clostridiales;D_4__Ruminococcaceae;D_5__Ruminiclostridium 6;D_6__uncultured bacterium;D_7__;D_8__;D_9__;D_10__;D_11__;D_12__;D_13__;D_14__	0.62(0.11,3.40)	3.27(0.63,16.95)	2.01(0.52,7.84)	0.83(0.16,4.44)	2.40(0.49,11.86)	1.99(0.53,7.52)			
ASV_107	Firmicutes;D_2__Clostridia;D_3__Clostridiales;D_4__Lachnospiraceae;D_5__CAG-56;D_6__uncultured bacterium;D_7__;D_8__;D_9__;D_10__;D_11__;D_12__;D_13__;D_14__	1.47(0.35,6.14)	0.59(0.15,2.33)	0.86(0.28,2.69)	1.42(0.33,6.02)	0.62(0.16,2.48)	0.89(0.28,2.78)			
ASV_110	Firmicutes;D_2__Clostridia;D_3__Clostridiales;D_4__Ruminococcaceae;D_5__Negativibacillus;D_6__uncultured bacterium;D_7__;D_8__;D_9__;D_10__;D_11__;D_12__;D_13__;D_14__	1.34(0.17,10.82)	1.77(0.24,13.20)	2.36(0.45,12.42)	0.61(0.08,4.79)	4.37(0.62,31.06)	2.69(0.53,13.71)			
ASV_113	Firmicutes;D_2__Clostridia;D_3__Clostridiales;D_4__Family XIII;D_5__Family XIII UCG-001;D_6__uncultured bacterium;D_7__;D_8__;D_9__;D_10__;D_11__;D_12__;D_13__;D_14__	1.46(0.05,45.46)	22.09(0.72,674.97)	32.22(1.84,563.14);p=0.01735						
ASV_117	Firmicutes;D_2__Clostridia;D_3__Clostridiales;D_4__Lachnospiraceae				0.42(0.04,4.71)	0.77(0.08,7.59)	0.32(0.05,2.18)			
ASV_119	Bacteroidetes;D_2__Bacteroidia;D_3__Bacteroidales;D_4__Bacteroidaceae;D_5__Bacteroides	3.01(0.65,14.05)	1.01(0.23,4.46)	3.05(0.90,10.37)	2.59(0.56,11.99)	0.82(0.19,3.53)	2.12(0.63,7.14)			
ASV_122	Firmicutes;D_2__Clostridia;D_3__Clostridiales;D_4__Lachnospiraceae;D_5__Eisenbergiella	1.39(0.04,45.59)	6.70(0.20,220.65)	9.31(0.50,172.18)	1.40(0.04,45.97)	25.10(0.54,1157.11)	35.24(1.39,890.53);p=0.03064			↑
ASV_123	Firmicutes;D_2__Clostridia;D_3__Clostridiales;D_4__Ruminococcaceae;D_5__uncultured	2.23(0.10,49.88)	0.45(0.02,8.96)	1.00(0.09,11.27)						
ASV_124	Proteobacteria;D_2__Deltaproteobacteria;D_3__Desulfovibrionales;D_4__Desulfovibrionaceae;D_5__Bilophila;D_6__uncultured bacterium;D_7__;D_8__;D_9__;D_10__;D_11__;D_12__;D_13__;D_14__	1.33(0.39,4.60)	1.01(0.31,3.31)	1.34(0.50,3.59)	1.31(0.37,4.55)	1.06(0.32,3.48)	1.38(0.51,3.72)			
ASV_127	Actinobacteria;D_2__Actinobacteria;D_3__Actinomycetales;D_4__Actinomycetaceae;D_5__Actinomyces	2.11(0.90,4.92)	0.79(0.35,1.80)	1.67(0.86,3.24)	2.41(1.03,5.68);p=0.0435	0.71(0.31,1.61)	1.72(0.88,3.33)	↑		
ASV_128	Firmicutes;D_2__Clostridia;D_3__Clostridiales;D_4__Family XIII;D_5__[Eubacterium] brachy group;D_6__uncultured bacterium;D_7__;D_8__;D_9__;D_10__;D_11__;D_12__;D_13__;D_14__	0.67(0.18,2.54)	2.12(0.59,7.65)	1.42(0.49,4.13)	0.70(0.18,2.66)	1.97(0.55,7.01)	1.38(0.48,3.99)			
ASV_129	Bacteroidetes;D_2__Bacteroidia;D_3__Bacteroidales;D_4__Tannerellaceae;D_5__Parabacteroides;D_6__Parabacteroides distasonis;D_7__;D_8__;D_9__;D_10__;D_11__;D_12__;D_13__;D_14__	0.27(0.01,9.74)	10.63(0.34,330.78)	2.90(0.17,49.59)	0.61(0.02,16.71)	21.74(0.93,509.30)	13.35(0.97,184.18)			
ASV_130	Firmicutes;D_2__Clostridia;D_3__Clostridiales;D_4__Ruminococcaceae;D_5__uncultured	2.57(0.44,15.12)	0.37(0.07,2.05)	0.96(0.24,3.84)						
ASV_131	Firmicutes;D_2__Clostridia;D_3__Clostridiales;D_4__Lachnospiraceae;D_5__Lachnoclostridium	1.74(0.61,4.96)	0.65(0.24,1.77)	1.13(0.49,2.58)	1.60(0.57,4.51)	0.77(0.29,2.07)	1.23(0.54,2.79)			
ASV_132	Firmicutes;D_2__Clostridia;D_3__Clostridiales;D_4__Lachnospiraceae;D_5__[Eubacterium] eligens group	3.05(0.96,9.66)	0.47(0.16,1.43)	1.44(0.58,3.59)	2.70(0.85,8.58)	0.46(0.15,1.39)	1.25(0.50,3.11)			
ASV_134	Firmicutes;D_2__Clostridia;D_3__Clostridiales;D_4__Ruminococcaceae;D_5__Ruminococcaceae UCG-013;D_6__uncultured organism;D_7__;D_8__;D_9__;D_10__;D_11__;D_12__;D_13__;D_14__	0.86(0.39,1.92)	1.21(0.56,2.63)	1.05(0.55,1.98)	0.86(0.39,1.94)	1.23(0.57,2.64)	1.06(0.56,2.00)			
ASV_135	Firmicutes;D_2__Clostridia;D_3__Clostridiales;D_4__Lachnospiraceae;D_5__Roseburia	0.48(0.17,1.36)	0.82(0.30,2.24)	0.39(0.17,0.89);p=0.02606	0.57(0.20,1.62)	0.76(0.28,2.07)	0.43(0.19,0.99);p=0.04785			↓
ASV_136	Firmicutes;D_2__Clostridia;D_3__Clostridiales;D_4__Ruminococcaceae;D_5__Ruminiclostridium 5;D_6__uncultured organism;D_7__;D_8__;D_9__;D_10__;D_11__;D_12__;D_13__;D_14__	1.29(0.60,2.77)	0.61(0.29,1.28)	0.79(0.43,1.45)	1.29(0.60,2.80)	0.60(0.29,1.25)	0.78(0.42,1.43)			

ASV_137	Firmicutes;D_2__Negativicutes;D_3__Selenomonadales;D_4__Veillonellaceae;D_5__Veillonella	2.02(0.53,7.61)	0.98(0.27,3.53)	1.98(0.69,5.68)	2.51(0.66,9.59)	0.75(0.21,2.69)	1.88(0.65,5.41)			
ASV_138	Firmicutes;D_2__Clostridia;D_3__Clostridiales;D_4__Christensenellaceae;D_5__Christensenellaceae R-7 group;D_6__metagenome;D_7__;D_8__;D_9__;D_10__;D_11__;D_12__;D_13__;D_14__	12.71(1.00,161.61)	0.08(0.01,0.88);p=0.03933	0.97(0.13,7.09)	41.71(3.01,578.37);p=0.00542	0.03(0.00,0.42);p=0.00859	1.42(0.21,9.77)	↑	↓	
ASV_140	Firmicutes;D_2__Clostridia;D_3__Clostridiales;D_4__Family XIII;D_5__Family XIII AD3011 group;D_6__uncultured bacterium;D_7__;D_8__;D_9__;D_10__;D_11__;D_12__;D_13__;D_14__				8451594559140486.00(0.00,Inf)	0.00(0.00,Inf)	1.73(0.08,35.54)			
ASV_141	Firmicutes;D_2__Clostridia;D_3__Clostridiales;D_4__Ruminococcaceae;D_5__Ruminiclostridium 5;D_6__gut metagenome;D_7__;D_8__;D_9__;D_10__;D_11__;D_12__;D_13__;D_14__	1.78(0.68,4.64)	1.22(0.49,3.07)	2.18(1.02,4.66);p=0.04531	1.76(0.68,4.54)	1.32(0.54,3.28)	2.33(1.10,4.94);p=0.02771			↑
ASV_144	Firmicutes;D_2__Bacilli;D_3__Lactobacillales;D_4__Carnobacteriaceae;D_5__Granulicatella	1.68(0.55,5.11)	0.93(0.32,2.73)	1.57(0.65,3.75)	2.02(0.66,6.17)	0.76(0.26,2.21)	1.53(0.64,3.65)			
ASV_145	Bacteroidetes;D_2__Bacteroidia;D_3__Bacteroidales;D_4__Rikenellaceae;D_5__Alistipes;D_6__Faecalibacterium prausnitzii;D_7__;D_8__;D_9__;D_10__;D_11__;D_12__;D_13__;D_14__	0.49(0.01,17.14)	0.51(0.02,15.80)	0.25(0.01,4.22)						
ASV_146	Firmicutes;D_2__Negativicutes;D_3__Selenomonadales;D_4__Veillonellaceae;D_5__Veillonella	0.55(0.10,3.00)	2.26(0.44,11.57)	1.24(0.32,4.78)						
ASV_147	Firmicutes;D_2__Clostridia;D_3__Clostridiales;D_4__Ruminococcaceae;D_5__Ruminococcaceae UCG-010;D_6__gut metagenome;D_7__;D_8__;D_9__;D_10__;D_11__;D_12__;D_13__;D_14__	1.87(0.03,138.02)	0.20(0.00,12.46)	0.37(0.01,11.15)						
ASV_148	Firmicutes;D_2__Clostridia;D_3__Clostridiales;D_4__Ruminococcaceae	0.42(0.09,1.95)	2.49(0.57,10.78)	1.05(0.31,3.59)	0.41(0.09,1.91)	2.87(0.66,12.41)	1.18(0.34,4.06)			
ASV_150	Firmicutes;D_2__Bacilli;D_3__Lactobacillales;D_4__Streptococcaceae;D_5__Lactococcus	1.27(0.44,3.64)	0.69(0.25,1.92)	0.88(0.38,2.03)	1.56(0.55,4.41)	0.62(0.23,1.68)	0.97(0.43,2.21)			
ASV_153	Firmicutes;D_2__Clostridia;D_3__Clostridiales;D_4__Lachnospiraceae;D_5__Lachnospiraceae NK4A136 group;D_6__uncultured organism;D_7__;D_8__;D_9__;D_10__;D_11__;D_12__;D_13__;D_14__	3.04(0.99,9.32)	0.29(0.10,0.86);p=0.02579	0.89(0.37,2.17)	4.23(1.39,12.87);p=0.01112	0.25(0.09,0.72);p=0.01002	1.05(0.44,2.53)	↑	↓	
ASV_154	Bacteroidetes;D_2__Bacteroidia;D_3__Bacteroidales;D_4__Bacteroidaceae;D_5__Bacteroides	0.87(0.17,4.46)	1.68(0.35,8.12)	1.46(0.40,5.36)	0.54(0.11,2.67)	1.67(0.36,7.74)	0.89(0.25,3.20)			
ASV_155	Firmicutes;D_2__Erysipelotrichia;D_3__Erysipelotrichales;D_4__Erysipelotrichaceae;D_5__Erysipelotrichaceae UCG-003	0.98(0.37,2.59)	0.78(0.31,1.98)	0.77(0.36,1.66)	0.97(0.37,2.59)	0.78(0.31,1.99)	0.76(0.35,1.66)			
ASV_157	Firmicutes;D_2__Clostridia;D_3__Clostridiales;D_4__Peptostreptococcaceae;D_5__Peptostreptococcus;D_6__uncultured organism;D_7__;D_8__;D_9__;D_10__;D_11__;D_12__;D_13__;D_14__	0.60(0.02,17.58)	0.41(0.02,10.14)	0.24(0.02,3.52)						
ASV_158	Firmicutes;D_2__Clostridia;D_3__Clostridiales;D_4__Lachnospiraceae;D_5__Lachnoclostridium;D_6__human gut metagenome;D_7__;D_8__;D_9__;D_10__;D_11__;D_12__;D_13__;D_14__	0.76(0.28,2.06)	1.93(0.74,5.03)	1.46(0.66,3.23)	0.85(0.31,2.33)	1.58(0.61,4.10)	1.35(0.61,2.99)			
ASV_159	Firmicutes;D_2__Clostridia;D_3__Clostridiales;D_4__Christensenellaceae;D_5__Christensenellaceae R-7 group;D_6__Clostridiales bacterium Marseille-P2846;D_7__;D_8__;D_9__;D_10__;D_11__;D_12__;D_13__;D_14__	5.55(0.05,578.47)	0.30(0.00,27.81)	1.67(0.06,45.32)						
ASV_160	Firmicutes;D_2__Clostridia;D_3__Clostridiales;D_4__Ruminococcaceae	1.38(0.09,22.34)	0.30(0.02,4.25)	0.42(0.05,3.42)	1.26(0.08,19.65)	0.43(0.03,5.94)	0.54(0.07,4.06)			
ASV_161	Firmicutes;D_2__Clostridia;D_3__Clostridiales;D_4__Defluviitaleaceae;D_5__Defluviitaleaceae UCG-011;D_6__uncultured bacterium;D_7__;D_8__;D_9__;D_10__;D_11__;D_12__;D_13__;D_14__	2.01(0.19,21.01)	1.20(0.13,11.56)	2.41(0.38,15.51)						
ASV_163	Firmicutes;D_2__Clostridia;D_3__Clostridiales;D_4__Ruminococcaceae	1.55(0.15,15.71)	0.75(0.08,6.96)	1.17(0.19,7.16)						
ASV_166	Firmicutes;D_2__Clostridia;D_3__Clostridiales;D_4__Lachnospiraceae;D_5__Lachnoclostridium;D_6__uncultured Clostridium sp.;D_7__;D_8__;D_9__;D_10__;D_11__;D_12__;D_13__;D_14__	3.10(0.08,128.38)	1.88(0.05,72.18)	5.83(0.30,113.07)						
ASV_167	Firmicutes;D_2__Clostridia;D_3__Clostridiales;D_4__Clostridiales vadinBB60 group;D_5__uncultured Thermoanaerobacterales bacterium;D_6__;D_7__;D_8__;D_9__;D_10__;D_11__;D_12__;D_13__;D_14__	0.65(0.02,20.33)	10.90(0.39,302.43)	7.06(0.45,110.16)						
ASV_168	Firmicutes;D_2__Clostridia;D_3__Clostridiales;D_4__Lachnospiraceae	2.55(0.58,11.11)	0.29(0.07,1.20)	0.74(0.24,2.34)	2.68(0.61,11.78)	0.31(0.07,1.25)	0.82(0.26,2.58)			
ASV_169	Firmicutes;D_2__Clostridia;D_3__Clostridiales;D_4__Lachnospiraceae;D_5__[Eubacterium] eligens group	0.82(0.16,4.27)	0.77(0.16,3.75)	0.63(0.17,2.32)						
ASV_170	Firmicutes;D_2__Clostridia;D_3__Clostridiales;D_4__Lachnospiraceae;D_5__Lachnospiraceae NK4A136 group	1.66(0.44,6.30)	0.65(0.18,2.34)	1.07(0.37,3.10)	1.45(0.38,5.46)	0.58(0.16,2.05)	0.84(0.29,2.39)			

ASV_171	Firmicutes;D_2__Clostridia;D_3__Clostridiales;D_4__Ruminococcaceae;D_5__Ruminococcaceae NK4A214 group;D_6__uncultured organism;D_7__;D_8__;D_9__;D_10__;D_11__;D_12__;D_13__;D_14__	1.09(0.09,12.73)	1.88(0.18,19.98)	2.04(0.29,14.41)						
ASV_172	Firmicutes;D_2__Erysipelotrichia;D_3__Erysipelotrichales;D_4__Erysipelotrichaceae;D_5__Holdemania	0.44(0.14,1.35)	1.57(0.54,4.60)	0.69(0.28,1.69)	0.44(0.14,1.37)	1.56(0.53,4.57)	0.69(0.28,1.70)			
ASV_173	Firmicutes;D_2__Clostridia;D_3__Clostridiales;D_4__Lachnospiraceae;D_5__Sellimonas;D_6__uncultured bacterium;D_7__;D_8__;D_9__;D_10__;D_11__;D_12__;D_13__;D_14__	11.17(0.47,267.29)	0.21(0.01,4.92)	2.40(0.32,17.74)	21.58(1.18,394.15);p=0.03824	1.28(0.06,26.74)	27.57(2.82,269.74);p=0.00437	↑		↑
ASV_174	Firmicutes;D_2__Clostridia;D_3__Clostridiales;D_4__Lachnospiraceae;D_5__uncultured;D_6__uncultured organism;D_7__;D_8__;D_9__;D_10__;D_11__;D_12__;D_13__;D_14__	0.71(0.15,3.30)	3.57(0.81,15.74)	2.52(0.74,8.58)	0.57(0.12,2.70)	4.13(0.94,18.09)	2.37(0.69,8.11)			
ASV_175	Firmicutes;D_2__Clostridia;D_3__Clostridiales;D_4__Christensenellaceae;D_5__uncultured;D_6__uncultured bacterium;D_7__;D_8__;D_9__;D_10__;D_11__;D_12__;D_13__;D_14__	1.77(0.34,9.37)	0.31(0.06,1.53)	0.55(0.15,2.01)	1.74(0.32,9.33)	0.32(0.06,1.56)	0.55(0.15,2.03)			
ASV_177	Firmicutes;D_2__Clostridia;D_3__Clostridiales;D_4__Christensenellaceae;D_5__Christensenellaceae R-7 group	2044367874.72(0.00,Inf)	0.00(0.00,Inf)	0.09(0.01,1.29)	2733595161221388.00(0.00,Inf)	0.00(0.00,Inf)	0.18(0.01,2.50)			
ASV_179	Firmicutes;D_2__Clostridia;D_3__Clostridiales;D_4__Ruminococcaceae;D_5__DTU089	1.85(0.63,5.42)	0.77(0.27,2.18)	1.43(0.61,3.34)	1.95(0.66,5.75)	0.75(0.27,2.11)	1.47(0.63,3.45)			
ASV_180	Actinobacteria;D_2__Coriobacteriia;D_3__Coriobacteriales;D_4__Coriobacteriales Incertae Sedis;D_5__uncultured;D_6__gut metagenome;D_7__;D_8__;D_9__;D_10__;D_11__;D_12__;D_13__;D_14__	3.00(0.55,16.40)	0.55(0.11,2.80)	1.63(0.43,6.25)	2.21(0.40,12.23)	0.58(0.11,2.97)	1.29(0.33,4.95)			
ASV_181	Firmicutes;D_2__Clostridia;D_3__Clostridiales;D_4__Ruminococcaceae;D_5__Anaerotruncus;D_6__Anaerotruncus sp. AT3;D_7__;D_8__;D_9__;D_10__;D_11__;D_12__;D_13__;D_14__	1.39(0.04,44.58)	0.39(0.01,10.95)	0.54(0.04,8.38)						
ASV_182	Firmicutes;D_2__Clostridia;D_3__Clostridiales;D_4__Lachnospiraceae	0.96(0.36,2.56)	0.78(0.30,1.99)	0.75(0.35,1.62)	0.86(0.32,2.28)	0.89(0.35,2.23)	0.76(0.35,1.64)			
ASV_183	Actinobacteria;D_2__Coriobacteriia;D_3__Coriobacteriales;D_4__Eggerthellaceae	1.65(0.40,6.85)	0.21(0.05,0.81);p=0.024	0.34(0.12,1.03)	1.93(0.48,7.74)	0.28(0.07,1.04)	0.54(0.19,1.56)			
ASV_184	Firmicutes;D_2__Clostridia;D_3__Clostridiales;D_4__Ruminococcaceae;D_5__uncultured	2.57(0.26,25.74)	0.13(0.01,1.22)	0.34(0.06,2.12)	1.72(0.17,16.93)	0.22(0.02,1.94)	0.38(0.06,2.29)			
ASV_185	Firmicutes;D_2__Clostridia;D_3__Clostridiales;D_4__Ruminococcaceae;D_5__Anaerotruncus;D_6__Anaerotruncus rubiinfantis;D_7__;D_8__;D_9__;D_10__;D_11__;D_12__;D_13__;D_14__	3.06(0.40,23.58)	0.67(0.09,4.84)	2.04(0.42,9.81)	1.87(0.25,14.05)	0.69(0.10,4.76)	1.28(0.27,6.03)			
ASV_189	Firmicutes;D_2__Clostridia;D_3__Clostridiales;D_4__Lachnospiraceae;D_5__[Eubacterium] ventriosum group	0.39(0.13,1.18)	1.67(0.58,4.80)	0.66(0.28,1.57)	0.32(0.11,0.97);p=0.04362	1.74(0.61,4.93)	0.56(0.24,1.34)	↓		
ASV_191	Proteobacteria;D_2__Gammaproteobacteria;D_3__Pseudomonadales;D_4__Pseudomonadaceae;D_5__Pseudomonas	2.09(0.21,20.50)	0.63(0.07,5.67)	1.31(0.22,7.88)	2.98(0.30,29.19)	0.61(0.07,5.41)	1.82(0.31,10.80)			
ASV_192	Firmicutes;D_2__Clostridia;D_3__Clostridiales;D_4__Lachnospiraceae;D_5__GCA-900066575				1.96(0.36,10.57)	1.36(0.27,6.90)	2.66(0.70,10.06)			
ASV_194	Firmicutes;D_2__Clostridia;D_3__Clostridiales;D_4__Ruminococcaceae;D_5__Fournierella;D_6__uncultured bacterium;D_7__;D_8__;D_9__;D_10__;D_11__;D_12__;D_13__;D_14__	1.96(0.28,13.55)	1.02(0.16,6.55)	2.00(0.43,9.24)	1.76(0.26,12.02)	0.82(0.13,5.13)	1.44(0.31,6.55)			
ASV_196	Firmicutes;D_2__Clostridia;D_3__Clostridiales;D_4__Ruminococcaceae;D_5__Angelakisella;D_6__uncultured bacterium;D_7__;D_8__;D_9__;D_10__;D_11__;D_12__;D_13__;D_14__	1.21(0.13,11.18)	2.58(0.30,22.14)	3.11(0.53,18.38)						
ASV_198	Actinobacteria;D_2__Coriobacteriia;D_3__Coriobacteriales;D_4__Eggerthellaceae;D_5__Gordonibacter;D_6__unidentified;D_7__;D_8__;D_9__;D_10__;D_11__;D_12__;D_13__;D_14__	0.79(0.23,2.69)	0.56(0.17,1.80)	0.44(0.17,1.16)	0.80(0.24,2.63)	0.76(0.25,2.38)	0.61(0.24,1.56)			
ASV_199	Firmicutes;D_2__Clostridia;D_3__Clostridiales;D_4__Lachnospiraceae;D_5__Lachnospiraceae UCG-010;D_6__uncultured organism;D_7__;D_8__;D_9__;D_10__;D_11__;D_12__;D_13__;D_14__	0.81(0.17,4.00)	0.82(0.18,3.80)	0.67(0.19,2.37)	0.71(0.15,3.49)	1.06(0.23,4.80)	0.75(0.21,2.64)			
ASV_202	Firmicutes;D_2__Clostridia;D_3__Clostridiales;D_4__Lachnospiraceae;D_5__GCA-900066755;D_6__uncultured bacterium;D_7__;D_8__;D_9__;D_10__;D_11__;D_12__;D_13__;D_14__	4.02(0.37,44.14)	0.61(0.06,6.31)	2.47(0.39,15.47)	3.51(0.31,39.20)	0.58(0.06,5.96)	2.04(0.32,12.85)			
ASV_205	Firmicutes;D_2__Clostridia;D_3__Clostridiales;D_4__Christensenellaceae;D_5__Christensenellaceae R-7 group	1.63(0.13,20.42)	1.58(0.13,18.85)	2.59(0.35,19.30)						
ASV_206	Actinobacteria;D_2__Coriobacteriia;D_3__Coriobacteriales;D_4__Eggerthellaceae;D_5__Gordonibacter;D_6__uncultured bacterium;D_7__;D_8__;D_9__;D_10__;D_11__;D_12__;D_13__;D_14__				6.14(0.92,40.85)	0.40(0.06,2.47)	2.43(0.60,9.81)			
ASV_207	Firmicutes;D_2__Clostridia;D_3__Clostridiales;D_4__Lachnospiraceae;D_5__Lachnoclostridium	2.39(0.14,39.71)	1.91(0.10,35.33)	4.58(0.47,44.80)						

ASV_209	Firmicutes;D_2__Clostridia;D_3__Clostridiales;D_4__Lachnospiraceae;D_5__GCA-900066575;D_6__uncultured organism;D_7__;D_8__;D_9__;D_10__;D_11__;D_12__;D_13__;D_14__	6.58(0.52,83.62)	0.28(0.02,3.22)	1.81(0.26,12.76)	5.93(0.46,77.03)	0.29(0.02,3.35)	1.70(0.24,12.13)			
ASV_211	Firmicutes;D_2__Clostridia;D_3__Clostridiales;D_4__Christensenellaceae;D_5__uncultured;D_6__uncultured bacterium;D_7__;D_8__;D_9__;D_10__;D_11__;D_12__;D_13__;D_14__	1.16(0.04,30.38)	2.88(0.12,67.33)	3.35(0.25,45.25)	1.68(0.07,40.80)	1.45(0.07,30.52)	2.43(0.19,30.52)			
ASV_212	Actinobacteria;D_2__Coriobacteriia;D_3__Coriobacteriales;D_4__Eggerthellaceae;D_5__Adlercreutzia;D_6__uncultured bacterium;D_7__;D_8__;D_9__;D_10__;D_11__;D_12__;D_13__;D_14__	2.14(0.72,6.39)	0.58(0.20,1.66)	1.24(0.52,2.96)	1.83(0.61,5.49)	0.56(0.20,1.61)	1.03(0.43,2.46)			
ASV_213	Firmicutes;D_2__Clostridia;D_3__DTU014;D_4__uncultured bacterium;D_5__;D_6__;D_7__;D_8__;D_9__;D_10__;D_11__;D_12__;D_13__;D_14__	3.95(0.42,36.85)	0.69(0.08,5.97)	2.73(0.47,15.92)	2.64(0.30,23.31)	0.65(0.08,5.24)	1.71(0.31,9.44)			
ASV_214	Actinobacteria;D_2__Actinobacteria;D_3__Actinomycetales;D_4__Actinomycetaceae;D_5__Actinomyces	5.77(0.16,202.95)	0.26(0.01,8.18)	1.48(0.11,19.22)	4.88(0.14,172.71)	0.34(0.01,11.25)	1.66(0.14,19.02)			
ASV_215	Firmicutes;D_2__Clostridia;D_3__Clostridiales;D_4__Lachnospiraceae;D_5__Lachnoclostridium	1.72(0.39,7.59)	0.88(0.21,3.67)	1.50(0.46,4.87)	1.72(0.38,7.70)	0.88(0.21,3.70)	1.51(0.46,4.95)			
ASV_216	Firmicutes;D_2__Clostridia;D_3__Clostridiales;D_4__Lachnospiraceae;D_5__Lachnospiraceae NK4A136 group;D_6__uncultured organism;D_7__;D_8__;D_9__;D_10__;D_11__;D_12__;D_13__;D_14__	0.38(0.12,1.26)	0.65(0.21,2.04)	0.25(0.10,0.64);p=0.00395	0.42(0.13,1.36)	0.94(0.31,2.89)	0.39(0.15,1.00)			
ASV_218	Firmicutes;D_2__Clostridia;D_3__Clostridiales;D_4__Ruminococcaceae;D_5__Ruminococcaceae UCG-003;D_6__uncultured organism;D_7__;D_8__;D_9__;D_10__;D_11__;D_12__;D_13__;D_14__	1.79(0.57,5.65)	0.32(0.11,0.98);p=0.04511	0.58(0.23,1.44)	1.78(0.56,5.70)	0.32(0.11,0.98);p=0.04603	0.58(0.23,1.45)		↓	
ASV_219	Firmicutes;D_2__Clostridia;D_3__Clostridiales;D_4__Christensenellaceae;D_5__Christensenellaceae R-7 group	8.98(1.25,64.62);p=0.02927	0.28(0.04,1.87)	2.48(0.56,11.06)						
ASV_220	Firmicutes;D_2__Clostridia;D_3__Clostridiales;D_4__Ruminococcaceae;D_5__Hydrogenoanaerobacterium	0.23(0.01,7.92)	2.58(0.09,75.40)	0.60(0.04,10.05)						
ASV_222	Firmicutes;D_2__Clostridia;D_3__Clostridiales;D_4__Lachnospiraceae;D_5__uncultured;D_6__intestinal bacterium CG19-1;D_7__;D_8__;D_9__;D_10__;D_11__;D_12__;D_13__;D_14__	0.41(0.05,3.26)	1.42(0.20,10.13)	0.59(0.11,3.07)	0.45(0.06,3.52)	1.74(0.25,12.12)	0.79(0.15,4.10)			
ASV_223	Firmicutes;D_2__Clostridia;D_3__Clostridiales;D_4__Christensenellaceae;D_5__Catabacter;D_6__Christensenella massiliensis;D_7__;D_8__;D_9__;D_10__;D_11__;D_12__;D_13__;D_14__	1.06(0.03,35.11)	0.19(0.01,5.48)	0.20(0.01,3.19)	0.81(0.04,18.17)	5.07(0.25,104.52)	4.09(0.32,53.12)			
ASV_224	Firmicutes;D_2__Clostridia;D_3__Clostridiales;D_4__Ruminococcaceae;D_5__Ruminococcaceae UCG-007	2.69(0.15,49.03)	0.98(0.06,16.64)	2.64(0.28,25.33)	3.36(0.17,65.60)	1.19(0.06,22.99)	4.01(0.40,39.68)			
ASV_225	Firmicutes;D_2__Clostridia;D_3__Clostridiales;D_4__Lachnospiraceae;D_5__uncultured	0.62(0.03,14.42)	0.42(0.02,8.47)	0.26(0.02,3.13)						
ASV_227	Firmicutes;D_2__Clostridia;D_3__Clostridiales;D_4__Lachnospiraceae;D_5__Lachnospiraceae NK4A136 group;D_6__uncultured organism;D_7__;D_8__;D_9__;D_10__;D_11__;D_12__;D_13__;D_14__	3.31(1.02,10.76);p=0.04649	0.20(0.06,0.61);p=0.00478	0.65(0.25,1.65)	3.79(1.16,12.35);p=0.02702	0.19(0.06,0.60);p=0.0042	0.73(0.29,1.86)	↑	↓	
ASV_229	Firmicutes;D_2__Clostridia;D_3__Clostridiales;D_4__Ruminococcaceae;D_5__[Eubacterium] coprostanoligenes group;D_6__human gut metagenome;D_7__;D_8__;D_9__;D_10__;D_11__;D_12__;D_13__;D_14__	0.88(0.04,21.41)	0.99(0.05,21.23)	0.87(0.07,10.92)	1.93(0.10,36.09)	8.50(0.52,139.15)	16.37(1.60,167.45);p=0.01844			↑
ASV_233	Proteobacteria;D_2__Deltaproteobacteria;D_3__Desulfovibrionales;D_4__Desulfovibrionaceae;D_5__Desulfovibrio;D_6__Desulfovibrio desulfuricans;D_7__;D_8__;D_9__;D_10__;D_11__;D_12__;D_13__;D_14__	0.01(0.00,0.35);p=0.01352	8.64(0.21,350.07)	0.05(0.00,1.45)						
ASV_234	Firmicutes;D_2__Clostridia;D_3__Clostridiales;D_4__Ruminococcaceae;D_5__Ruminiclostridium 5;D_6__uncultured bacterium;D_7__;D_8__;D_9__;D_10__;D_11__;D_12__;D_13__;D_14__				1725307888166370.00(0.00,Inf)	0.00(0.00,Inf)	0.77(0.11,5.45)			
ASV_236	Firmicutes;D_2__Erysipelotrichia;D_3__Erysipelotrichales;D_4__Erysipelotrichaceae;D_5__Dielma;D_6__uncultured bacterium;D_7__;D_8__;D_9__;D_10__;D_11__;D_12__;D_13__;D_14__	0.75(0.02,22.62)	0.58(0.03,13.45)	0.44(0.03,5.94)	0.79(0.03,22.56)	0.58(0.03,12.98)	0.46(0.04,5.89)			
ASV_237	Firmicutes;D_2__Clostridia;D_3__Clostridiales;D_4__Lachnospiraceae;D_5__Lachnospira	3.63(0.61,21.77)	0.22(0.04,1.23)	0.79(0.19,3.29)	2.38(0.41,13.81)	0.28(0.05,1.52)	0.68(0.17,2.72)			
ASV_238	Firmicutes;D_2__Clostridia;D_3__Clostridiales;D_4__Ruminococcaceae	0.32(0.03,3.99)	1.26(0.12,13.51)	0.41(0.05,3.08)	0.41(0.04,4.08)	3.08(0.35,27.04)	1.28(0.19,8.48)			
ASV_239	Firmicutes;D_2__Bacilli;D_3__Lactobacillales;D_4__Leuconostocaceae;D_5__Leuconostoc	2466246965.88(0.00,Inf)	0.00(0.00,Inf)	0.67(0.13,3.50)						
ASV_240	Firmicutes;D_2__Clostridia;D_3__Clostridiales;D_4__Christensenellaceae;D_5__Christensenellaceae R-7 group;D_6__uncultured bacterium;D_7__;D_8__;D_9__;D_10__;D_11__;D_12__;D_13__;D_14__	24.43(2.02,295.75);p=0.01202	0.91(0.08,10.50)	22.19(3.22,152.71);p=0.00163						

ASV_241	Firmicutes;D_2__Clostridia;D_3__Clostridiales;D_4__Ruminococcaceae;D_5__uncultured	0.14(0.01,3.28)	51175190315.32(0.00,Inf)	7224506364.62(0.00,Inf)	0.10(0.00,3.39)	7331486318609254.00(0.00,Inf)	699093926968992.00(0.00,Inf)			
ASV_242	Firmicutes;D_2__Bacilli;D_3__Bacillales;D_4__Staphylococcaceae;D_5__Staphylococcus	27.50(2.16,350.27);p=0.01069	0.20(0.02,2.38)	5.52(0.85,35.74)	55.08(3.93,772.65);p=0.00293	0.18(0.01,2.28)	9.70(1.66,56.65);p=0.01165	↑		↑
ASV_243	Firmicutes;D_2__Clostridia;D_3__Clostridiales;D_4__Ruminococcaceae;D_5__uncultured	13398045521.52(0.00,Inf)	0.00(0.00,Inf)	21.44(0.80,578.41)						
ASV_246	Firmicutes;D_2__Clostridia;D_3__Clostridiales;D_4__Lachnospiraceae	1638095069.03(0.00,Inf)	0.00(0.00,Inf)	0.42(0.08,2.26)						
ASV_249	Firmicutes;D_2__Clostridia;D_3__Clostridiales;D_4__Lachnospiraceae;D_5__Lachnoclostridium	2.32(0.53,10.04)	0.92(0.22,3.78)	2.13(0.67,6.77)	2.89(0.67,12.47)	0.87(0.22,3.54)	2.52(0.80,7.97)			
ASV_250	Firmicutes;D_2__Clostridia;D_3__Clostridiales;D_4__Christensenellaceae;D_5__Christensenellaceae R-7 group	3.60(0.30,42.60)	4.49(0.35,57.75)	16.17(1.98,132.04);p=0.0094						
ASV_252	Firmicutes;D_2__Erysipelotrichia;D_3__Erysipelotrichales;D_4__Erysipelotrichaceae;D_5__Erysipelatoclostridium	0.31(0.03,2.85)	1.04(0.12,8.84)	0.32(0.05,1.87)						
ASV_253	Firmicutes;D_2__Clostridia;D_3__Clostridiales;D_4__Family XIII;D_5__Mogibacterium	1.77(0.38,8.22)	0.46(0.11,2.00)	0.81(0.26,2.50)	2.06(0.42,10.02)	0.37(0.08,1.70)	0.77(0.25,2.35)			
ASV_255	Actinobacteria;D_2__Actinobacteria;D_3__Actinomycetales;D_4__Actinomycetaceae;D_5__Actinomyces	2.91(0.86,9.79)	0.55(0.17,1.78)	1.60(0.64,4.01)	2.79(0.82,9.47)	0.56(0.17,1.81)	1.56(0.62,3.93)			
ASV_256	Firmicutes;D_2__Clostridia;D_3__Clostridiales;D_4__Family XIII;D_5__Family XIII AD3011 group;D_6__uncultured bacterium;D_7__;D_8__;D_9__;D_10__;D_11__;D_12__;D_13__;D_14__	1.11(0.11,11.50)	1.59(0.17,15.20)	1.75(0.27,11.30)	0.94(0.09,9.79)	1.65(0.18,15.44)	1.55(0.24,9.94)			
ASV_258	Firmicutes;D_2__Clostridia;D_3__Clostridiales;D_4__Defluviitaleaceae;D_5__Defluviitaleaceae UCG-011;D_6__uncultured bacterium;D_7__;D_8__;D_9__;D_10__;D_11__;D_12__;D_13__;D_14__	2.07(0.31,13.73)	0.33(0.05,2.04)	0.69(0.16,2.91)	2.57(0.41,16.14)	0.35(0.06,1.99)	0.89(0.23,3.46)			
ASV_259	Patescibacteria;D_2__Saccharimonadia;D_3__Saccharimonadales;D_4__Saccharimonadaceae	2.70(0.69,10.62)	1.28(0.33,4.92)	3.47(1.18,10.16);p=0.02349	2.51(0.64,9.88)	1.36(0.36,5.16)	3.41(1.17,9.97);p=0.02491			↑
ASV_260	Actinobacteria;D_2__Coriobacteriia;D_3__Coriobacteriales;D_4__Eggerthellaceae;D_5__Enterorhabdus;D_6__uncultured bacterium;D_7__;D_8__;D_9__;D_10__;D_11__;D_12__;D_13__;D_14__	2.81(0.22,36.46)	0.25(0.02,2.91)	0.70(0.10,4.71)	2.48(0.19,32.01)	0.27(0.02,3.11)	0.68(0.10,4.61)			
ASV_262	Firmicutes;D_2__Clostridia;D_3__Clostridiales;D_4__Defluviitaleaceae;D_5__Defluviitaleaceae UCG-011;D_6__uncultured bacterium;D_7__;D_8__;D_9__;D_10__;D_11__;D_12__;D_13__;D_14__	21078703731.27(0.00,Inf)	0.00(0.00,Inf)	0.11(0.00,2.58)						
ASV_264	Firmicutes;D_2__Clostridia;D_3__Clostridiales;D_4__Ruminococcaceae;D_5__Ruminiclostridium 5;D_6__uncultured bacterium;D_7__;D_8__;D_9__;D_10__;D_11__;D_12__;D_13__;D_14__	4.46(0.57,34.71)	0.21(0.03,1.55)	0.94(0.24,3.73)	3.15(0.46,21.50)	0.32(0.05,2.02)	1.01(0.27,3.78)			
ASV_265	Firmicutes;D_2__Clostridia;D_3__Clostridiales;D_4__Lachnospiraceae;D_5__[Ruminococcus] torques group	4.74(0.44,51.53)	1.34(0.13,14.18)	6.34(0.98,41.20)	2.91(0.28,29.99)	1.38(0.14,13.39)	4.01(0.64,25.22)			
ASV_268	Proteobacteria;D_2__Gammaproteobacteria;D_3__Betaproteobacteriales;D_4__Burkholderiaceae;D_5__Parasutterella	0.86(0.20,3.60)	0.69(0.17,2.74)	0.59(0.19,1.85)	0.91(0.21,3.84)	0.74(0.19,2.92)	0.67(0.21,2.10)			
ASV_270	Bacteroidetes;D_2__Bacteroidia;D_3__Bacteroidales;D_4__Bacteroidaceae;D_5__Bacteroides;D_6__Bacteroides uniformis;D_7__;D_8__;D_9__;D_10__;D_11__;D_12__;D_13__;D_14__	4.16(0.75,23.18)	0.41(0.08,2.15)	1.71(0.44,6.70)	5.26(0.94,29.29)	0.32(0.06,1.65)	1.69(0.43,6.57)			
ASV_271	Firmicutes;D_2__Clostridia;D_3__Clostridiales;D_4__Ruminococcaceae;D_5__Ruminococcaceae UCG-002				2.04(0.13,33.15)	0.30(0.02,4.28)	0.61(0.07,5.57)			
ASV_274	Firmicutes;D_2__Clostridia;D_3__Clostridiales;D_4__Christensenellaceae;D_5__Christensenellaceae R-7 group	0.29(0.03,3.01)	0.39(0.04,3.72)	0.11(0.02,0.73);p=0.02197	0.45(0.04,4.71)	0.35(0.04,3.29)	0.16(0.03,1.02)			
ASV_275	Firmicutes;D_2__Clostridia;D_3__Clostridiales;D_4__Ruminococcaceae;D_5__Ruminococcaceae UCG-002;D_6__uncultured organism;D_7__;D_8__;D_9__;D_10__;D_11__;D_12__;D_13__;D_14__	0.55(0.14,2.13)	1.26(0.34,4.67)	0.69(0.23,2.03)	0.55(0.14,2.15)	1.23(0.33,4.56)	0.67(0.23,2.00)			
ASV_277	Firmicutes;D_2__Clostridia;D_3__Clostridiales;D_4__Ruminococcaceae;D_5__Faecalibacterium;D_6__metagenome;D_7__;D_8__;D_9__;D_10__;D_11__;D_12__;D_13__;D_14__	0.79(0.15,4.13)	1.08(0.22,5.27)	0.86(0.23,3.18)	0.55(0.10,2.91)	1.34(0.28,6.55)	0.74(0.20,2.77)			
ASV_278	Bacteroidetes;D_2__Bacteroidia;D_3__Bacteroidales;D_4__Bacteroidaceae;D_5__Bacteroides;D_6__Bacteroides stercoris ATCC 43183;D_7__;D_8__;D_9__;D_10__;D_11__;D_12__;D_13__;D_14__	3.65(0.57,23.32)	0.56(0.09,3.30)	2.03(0.47,8.83)						
ASV_282	Firmicutes;D_2__Clostridia;D_3__Clostridiales;D_4__Ruminococcaceae;D_5__Faecalibacterium	0.92(0.21,3.95)	0.44(0.11,1.80)	0.40(0.13,1.29)	1.06(0.25,4.62)	0.43(0.11,1.75)	0.46(0.14,1.47)			
ASV_283	Firmicutes;D_2__Clostridia;D_3__Clostridiales;D_4__Ruminococcaceae;D_5__Ruminococcaceae UCG-002	1.32(0.24,7.35)	0.61(0.12,3.17)	0.80(0.21,3.14)						

ASV_288	Firmicutes;D_2__Clostridia;D_3__Clostridiales;D_4__Lachnospiraceae;D_5__[Eubacterium] hallii group	2.42(1.12,5.25);p=0.02477	0.65(0.31,1.37)	1.58(0.86,2.92)	2.49(1.15,5.43);p=0.02122	0.66(0.31,1.38)	1.64(0.88,3.03)	↑		
ASV_289	Bacteroidetes;D_2__Bacteroidia;D_3__Bacteroidales;D_4__Bacteroidaceae;D_5__Bacteroides	0.92(0.10,8.30)	1.26(0.15,10.42)	1.16(0.20,6.64)						
ASV_291	Firmicutes;D_2__Clostridia;D_3__Clostridiales;D_4__Lachnospiraceae;D_5__Lachnospira	0.42(0.14,1.24)	1.30(0.46,3.70)	0.55(0.23,1.29)	0.41(0.14,1.22)	1.28(0.45,3.64)	0.52(0.22,1.24)			
ASV_292	Firmicutes;D_2__Clostridia;D_3__Clostridiales;D_4__Ruminococcaceae;D_5__Butyricicoccus	0.66(0.24,1.76)	1.25(0.48,3.22)	0.82(0.37,1.79)	0.66(0.25,1.78)	1.37(0.54,3.50)	0.91(0.42,1.98)			
ASV_296	Firmicutes;D_2__Clostridia;D_3__Clostridiales;D_4__Lachnospiraceae	1.09(0.42,2.82)	0.97(0.39,2.42)	1.06(0.50,2.25)	1.08(0.41,2.83)	0.96(0.38,2.40)	1.04(0.49,2.23)			
ASV_298	Firmicutes;D_2__Clostridia;D_3__Clostridiales;D_4__Ruminococcaceae;D_5__Oscillibacter;D_6__uncultured organism;D_7__;D_8__;D_9__;D_10__;D_11__;D_12__;D_13__;D_14__	0.98(0.44,2.16)	1.24(0.58,2.67)	1.22(0.65,2.28)	0.99(0.45,2.19)	1.20(0.56,2.55)	1.19(0.63,2.22)			
ASV_301	Firmicutes;D_2__Clostridia;D_3__Clostridiales;D_4__Lachnospiraceae;D_5__Lachnospiraceae UCG-008;D_6__uncultured organism;D_7__;D_8__;D_9__;D_10__;D_11__;D_12__;D_13__;D_14__	0.63(0.21,1.93)	1.39(0.47,4.08)	0.87(0.36,2.13)	0.65(0.21,2.02)	1.35(0.46,3.96)	0.88(0.36,2.15)			
ASV_302	Firmicutes;D_2__Clostridia;D_3__Clostridiales;D_4__Lachnospiraceae;D_5__uncultured	1.11(0.43,2.84)	1.17(0.47,2.89)	1.29(0.61,2.73)	1.17(0.45,3.02)	1.14(0.46,2.81)	1.33(0.62,2.81)			
ASV_303	Bacteroidetes;D_2__Bacteroidia;D_3__Bacteroidales;D_4__Marinifilaceae;D_5__Butyricimonas;D_6__uncultured organism;D_7__;D_8__;D_9__;D_10__;D_11__;D_12__;D_13__;D_14__	1.17(0.05,25.67)	0.14(0.01,2.65)	0.16(0.01,1.84)	0.18(0.01,4.20)	0.15(0.01,2.75)	0.03(0.00,0.34);p=0.00488			↓
ASV_304	Firmicutes;D_2__Clostridia;D_3__Clostridiales;D_4__Lachnospiraceae;D_5__Lachnospiraceae UCG-010;D_6__uncultured organism;D_7__;D_8__;D_9__;D_10__;D_11__;D_12__;D_13__;D_14__	0.09(0.01,1.17)	6.36(0.52,77.76)	0.55(0.07,4.38)	0.08(0.01,1.07)	4.34(0.38,49.11)	0.36(0.05,2.75)			
ASV_306	Firmicutes;D_2__Clostridia;D_3__Clostridiales;D_4__Lachnospiraceae;D_5__Lachnospiraceae UCG-001	14.57(2.14,99.17);p=0.00619	0.12(0.02,0.78);p=0.02657	1.80(0.40,8.06)	13.09(1.91,89.73);p=0.00882	0.12(0.02,0.76);p=0.02457	1.59(0.36,7.10)	↑	↓	
ASV_309	Firmicutes;D_2__Clostridia;D_3__Clostridiales;D_4__Lachnospiraceae;D_5__Lachnoclostridium;D_6__uncultured Firmicutes bacterium;D_7__;D_8__;D_9__;D_10__;D_11__;D_12__;D_13__;D_14__	0.86(0.25,2.90)	1.14(0.35,3.70)	0.98(0.37,2.58)	0.78(0.23,2.60)	1.32(0.42,4.19)	1.03(0.39,2.67)			
ASV_310	Bacteroidetes;D_2__Bacteroidia;D_3__Bacteroidales;D_4__Marinifilaceae;D_5__Butyricimonas;D_6__uncultured organism;D_7__;D_8__;D_9__;D_10__;D_11__;D_12__;D_13__;D_14__	2.16(0.15,31.59)	0.19(0.01,2.51)	0.41(0.05,3.44)	3.40(0.25,46.71)	0.15(0.01,1.83)	0.51(0.07,4.03)			
ASV_313	Firmicutes;D_2__Clostridia;D_3__Clostridiales;D_4__Ruminococcaceae;D_5__Ruminiclostridium 9	0.28(0.07,1.08)	1.81(0.50,6.53)	0.52(0.18,1.49)	0.22(0.06,0.83);p=0.02557	2.12(0.60,7.54)	0.47(0.16,1.34)	↓		
ASV_314	Firmicutes;D_2__Clostridia;D_3__Clostridiales;D_4__Lachnospiraceae;D_5__Lachnospiraceae UCG-004	1.36(0.30,6.25)	1.38(0.32,5.96)	1.88(0.56,6.30)	1.51(0.32,7.00)	1.23(0.28,5.31)	1.85(0.55,6.24)			
ASV_315	Firmicutes;D_2__Clostridia;D_3__Clostridiales;D_4__Lachnospiraceae;D_5__Lachnospiraceae UCG-004	0.64(0.23,1.78)	1.63(0.61,4.36)	1.04(0.46,2.35)	0.67(0.24,1.85)	1.34(0.51,3.54)	0.90(0.40,2.01)			
ASV_317	Firmicutes;D_2__Clostridia;D_3__Clostridiales;D_4__Ruminococcaceae;D_5__[Eubacterium] coprostanoligenes group				0.77(0.04,13.52)	0.26(0.02,4.01)	0.20(0.02,1.96)			
ASV_318	Firmicutes;D_2__Clostridia;D_3__Clostridiales;D_4__Lachnospiraceae	1.46(0.56,3.81)	0.83(0.33,2.10)	1.21(0.57,2.60)	1.37(0.52,3.61)	0.87(0.35,2.19)	1.19(0.56,2.57)			
ASV_322	Firmicutes;D_2__Clostridia;D_3__Clostridiales;D_4__Ruminococcaceae;D_5__Ruminococcaceae UCG-005;D_6__uncultured organism;D_7__;D_8__;D_9__;D_10__;D_11__;D_12__;D_13__;D_14__	1.06(0.17,6.44)	0.36(0.06,2.02)	0.38(0.09,1.58)	1.19(0.20,7.15)	0.36(0.07,2.00)	0.43(0.10,1.78)			
ASV_324	Firmicutes;D_2__Clostridia;D_3__Clostridiales;D_4__Ruminococcaceae;D_5__Angelakisella;D_6__uncultured bacterium;D_7__;D_8__;D_9__;D_10__;D_11__;D_12__;D_13__;D_14__	2.00(0.14,28.48)	0.52(0.04,6.67)	1.03(0.13,8.35)						
ASV_325	Firmicutes;D_2__Clostridia;D_3__Clostridiales;D_4__Clostridiales vadinBB60 group;D_5__gut metagenome;D_6__;D_7__;D_8__;D_9__;D_10__;D_11__;D_12__;D_13__;D_14__	18.42(0.42,810.23)	0.13(0.00,4.92)	2.37(0.12,44.80)						
ASV_327	Firmicutes;D_2__Clostridia;D_3__Clostridiales;D_4__Lachnospiraceae;D_5__GCA-900066575	0.58(0.19,1.82)	1.14(0.38,3.40)	0.66(0.27,1.64)	0.56(0.18,1.77)	1.19(0.40,3.57)	0.67(0.27,1.67)			
ASV_334	Firmicutes;D_2__Clostridia;D_3__Clostridiales;D_4__Lachnospiraceae;D_5__uncultured;D_6__uncultured bacterium adhufec382;D_7__;D_8__;D_9__;D_10__;D_11__;D_12__;D_13__;D_14__	1.92(0.58,6.33)	0.74(0.24,2.34)	1.43(0.56,3.67)	1.60(0.49,5.22)	0.73(0.24,2.26)	1.17(0.46,2.98)			
ASV_340	Firmicutes;D_2__Clostridia;D_3__Clostridiales;D_4__Ruminococcaceae;D_5__Flavonifractor;D_6__uncultured bacterium;D_7__;D_8__;D_9__;D_10__;D_11__;D_12__;D_13__;D_14__	6.32(0.97,41.42)	0.20(0.03,1.20)	1.24(0.29,5.43)	7.78(1.20,50.46);p=0.03143	0.23(0.04,1.38)	1.80(0.42,7.75)	↑		

ASV_345	Firmicutes;D_2__Clostridia;D_3__Clostridiales;D_4__Lachnospiraceae;D_5__Lachnospira				1.83(0.17,19.42)	0.51(0.05,4.81)	0.93(0.14,6.02)			
ASV_348	Firmicutes;D_2__Clostridia;D_3__Clostridiales;D_4__Lachnospiraceae;D_5__Moryella;D_6__human gut metagenome;D_7__;D_8__;D_9__;D_10__;D_11__;D_12__;D_13__;D_14__	0.48(0.15,1.56)	1.33(0.43,4.11)	0.64(0.25,1.63)	0.44(0.14,1.42)	1.20(0.39,3.66)	0.53(0.21,1.34)			
ASV_349	Firmicutes;D_2__Clostridia;D_3__Clostridiales;D_4__Ruminococcaceae;D_5__Flavonifractor	1.08(0.47,2.48)	1.12(0.50,2.49)	1.20(0.62,2.33)	1.11(0.48,2.56)	1.06(0.48,2.36)	1.17(0.60,2.28)			
ASV_350	Bacteroidetes;D_2__Bacteroidia;D_3__Bacteroidales;D_4__Marinifilaceae;D_5__Butyricimonas;D_6__uncultured organism;D_7__;D_8__;D_9__;D_10__;D_11__;D_12__;D_13__;D_14__	3.40(0.06,209.39)	0.13(0.00,6.70)	0.43(0.02,10.91)	3.09(0.07,132.72)	0.21(0.01,7.50)	0.65(0.04,12.02)			
ASV_354	Firmicutes;D_2__Clostridia;D_3__Clostridiales;D_4__Lachnospiraceae;D_5__Lachnospiraceae FCS020 group	0.71(0.15,3.37)	0.41(0.10,1.77)	0.29(0.09,0.98);p=0.04667	0.65(0.14,3.04)	0.39(0.09,1.60)	0.25(0.07,0.83);p=0.02319			↓
ASV_355	Firmicutes;D_2__Clostridia;D_3__Clostridiales;D_4__Ruminococcaceae;D_5__Ruminococcaceae UCG-009;D_6__uncultured bacterium;D_7__;D_8__;D_9__;D_10__;D_11__;D_12__;D_13__;D_14__	0.05(0.00,0.78);p=0.032	3.18(0.27,37.65)	0.17(0.02,1.52)						
ASV_357	Firmicutes;D_2__Clostridia;D_3__Clostridiales;D_4__Ruminococcaceae;D_5__Oscillibacter;D_6__human gut metagenome;D_7__;D_8__;D_9__;D_10__;D_11__;D_12__;D_13__;D_14__	0.69(0.29,1.63)	0.98(0.43,2.22)	0.68(0.34,1.33)	0.72(0.31,1.69)	0.85(0.38,1.91)	0.61(0.31,1.20)			
ASV_360	Firmicutes;D_2__Clostridia;D_3__Clostridiales;D_4__Ruminococcaceae;D_5__Ruminiclostridium 9;D_6__uncultured organism;D_7__;D_8__;D_9__;D_10__;D_11__;D_12__;D_13__;D_14__	0.62(0.21,1.81)	1.41(0.51,3.93)	0.88(0.37,2.05)	0.63(0.22,1.78)	1.13(0.42,3.01)	0.71(0.31,1.61)			
ASV_365	Firmicutes;D_2__Clostridia;D_3__Clostridiales;D_4__Ruminococcaceae;D_5__Intestinimonas	2.23(0.27,18.48)	0.89(0.12,6.84)	1.99(0.37,10.56)	1.99(0.25,15.89)	0.83(0.11,6.06)	1.65(0.32,8.44)			
ASV_366	Proteobacteria;D_2__Gammaproteobacteria;D_3__Pasteurellales;D_4__Pasteurellaceae;D_5__Haemophilus	1.10(0.29,4.12)	0.81(0.23,2.88)	0.89(0.31,2.53)	1.01(0.26,3.83)	0.83(0.23,2.96)	0.83(0.29,2.40)			
ASV_369	Firmicutes;D_2__Clostridia;D_3__Clostridiales;D_4__Ruminococcaceae;D_5__Ruminococcaceae UCG-003;D_6__uncultured bacterium;D_7__;D_8__;D_9__;D_10__;D_11__;D_12__;D_13__;D_14__	1.14(0.20,6.61)	0.66(0.12,3.59)	0.75(0.19,3.03)						
ASV_370	Firmicutes;D_2__Bacilli;D_3__Lactobacillales;D_4__Lactobacillaceae;D_5__Lactobacillus;D_6__Lactobacillus fermentum;D_7__;D_8__;D_9__;D_10__;D_11__;D_12__;D_13__;D_14__	0.13(0.01,2.26)	106.69(6.19,1838.99);p=0.0013	13.36(1.23,145.23);p=0.03317	0.08(0.00,1.29)	406.35(19.68,8389.74);p=0.0001;q=0.04098	31.99(2.59,394.52);p=0.00686		↑*	↑
ASV_371	Firmicutes;D_2__Clostridia;D_3__Clostridiales;D_4__Ruminococcaceae;D_5__uncultured;D_6__Clostridium phoceensis;D_7__;D_8__;D_9__;D_10__;D_11__;D_12__;D_13__;D_14__	2.39(1.05,5.47);p=0.03835	1.12(0.50,2.48)	2.67(1.39,5.13);p=0.00321	2.22(0.97,5.07)	1.01(0.46,2.23)	2.25(1.17,4.31);p=0.01465			↑
ASV_373	Firmicutes;D_2__Clostridia;D_3__Clostridiales;D_4__Ruminococcaceae;D_5__Oscillospira;D_6__uncultured bacterium;D_7__;D_8__;D_9__;D_10__;D_11__;D_12__;D_13__;D_14__	0.29(0.01,6.14)	0.43(0.03,7.02)	0.13(0.01,1.42)	0.66(0.02,25.24)	0.06(0.00,1.53)	0.04(0.00,0.53);p=0.01462			↓
ASV_374	Firmicutes;D_2__Clostridia;D_3__Clostridiales;D_4__Clostridiaceae 1;D_5__Clostridium sensu stricto 1	2.43(0.50,11.89)	0.68(0.15,3.12)	1.65(0.47,5.79)	3.05(0.63,14.71)	0.99(0.22,4.42)	3.01(0.87,10.43)			
ASV_376	Firmicutes;D_2__Clostridia;D_3__Clostridiales;D_4__Clostridiales vadinBB60 group;D_5__gut metagenome;D_6__;D_7__;D_8__;D_9__;D_10__;D_11__;D_12__;D_13__;D_14__	1.24(0.02,74.08)	1.82(0.04,94.13)	2.25(0.09,58.36)						
ASV_377	Firmicutes;D_2__Clostridia;D_3__Clostridiales;D_4__Ruminococcaceae;D_5__Phocea;D_6__uncultured bacterium;D_7__;D_8__;D_9__;D_10__;D_11__;D_12__;D_13__;D_14__	0.96(0.24,3.81)	1.54(0.41,5.81)	1.47(0.49,4.41)	0.94(0.23,3.76)	1.36(0.36,5.13)	1.28(0.42,3.83)			
ASV_378	Proteobacteria;D_2__Gammaproteobacteria;D_3__Enterobacteriales;D_4__Enterobacteriaceae;D_5__Escherichia-Shigella	1.65(0.40,6.84)	0.85(0.22,3.35)	1.41(0.46,4.36)						
ASV_380	Firmicutes;D_2__Clostridia;D_3__Clostridiales;D_4__Ruminococcaceae;D_5__Oscillibacter;D_6__Oscillibacter sp. PC13;D_7__;D_8__;D_9__;D_10__;D_11__;D_12__;D_13__;D_14__	0.10(0.00,2.12)	1.95(0.12,31.86)	0.19(0.02,2.40)	0.10(0.00,2.14)	2.00(0.13,30.62)	0.20(0.02,2.58)			
ASV_383	Firmicutes;D_2__Clostridia;D_3__Clostridiales;D_4__Clostridiales vadinBB60 group;D_5__uncultured bacterium;D_6__;D_7__;D_8__;D_9__;D_10__;D_11__;D_12__;D_13__;D_14__	1.70(0.22,13.05)	1.04(0.15,7.42)	1.76(0.35,8.87)	1.50(0.20,11.29)	1.18(0.17,8.10)	1.77(0.36,8.73)			
ASV_384	Firmicutes;D_2__Clostridia;D_3__Clostridiales;D_4__Ruminococcaceae;D_5__Ruminococcaceae UCG-005				0.92(0.11,7.90)	0.71(0.09,5.48)	0.65(0.12,3.52)			
ASV_387	Firmicutes;D_2__Clostridia;D_3__Clostridiales;D_4__Ruminococcaceae;D_5__Oscillospira;D_6__uncultured organism;D_7__;D_8__;D_9__;D_10__;D_11__;D_12__;D_13__;D_14__	3.04(0.05,199.42)	0.19(0.00,10.60)	0.58(0.03,11.43)						
ASV_388	Firmicutes;D_2__Clostridia;D_3__Clostridiales;D_4__Ruminococcaceae;D_5__Oscillospira	1.64(0.42,6.47)	0.51(0.14,1.91)	0.84(0.28,2.47)	2.10(0.53,8.29)	0.34(0.09,1.24)	0.71(0.24,2.06)			

ASV_389	Firmicutes;D_2__Clostridia;D_3__Clostridiales				0.00(0.00,Inf)	7.09(0.64,78.02)	0.00(0.00,Inf)			
ASV_392	Actinobacteria;D_2__Coriobacteriia;D_3__Coriobacteriales;D_4__Eggerthellaceae;D_5__Eggerthella	1.82(0.65,5.07)	0.97(0.36,2.61)	1.76(0.78,3.97)	1.59(0.57,4.45)	1.12(0.42,2.99)	1.79(0.79,4.03)			
ASV_393	Firmicutes;D_2__Clostridia;D_3__Clostridiales;D_4__Ruminococcaceae;D_5__Ruminococcaceae UCG-005;D_6__uncultured organism;D_7__;D_8__;D_9__;D_10__;D_11__;D_12__;D_13__;D_14__	1.92(0.27,13.62)	0.50(0.08,3.28)	0.96(0.20,4.50)	1.74(0.25,12.28)	0.80(0.13,5.18)	1.40(0.30,6.55)			
ASV_394	Firmicutes;D_2__Clostridia;D_3__Clostridiales;D_4__Family XIII;D_5__Family XIII AD3011 group;D_6__uncultured bacterium;D_7__;D_8__;D_9__;D_10__;D_11__;D_12__;D_13__;D_14__	0.21(0.02,2.00)	2.08(0.25,17.55)	0.43(0.07,2.71)	0.20(0.02,1.98)	2.22(0.26,18.76)	0.44(0.07,2.84)			
ASV_396	Firmicutes;D_2__Clostridia;D_3__Clostridiales;D_4__Lachnospiraceae;D_5__Blautia	31477719004.79(0.00,Inf)	0.00(0.00,Inf)	1.80(0.12,26.42)	688509934243357.00(0.00,Inf)	0.00(0.00,Inf)	0.20(0.02,2.34)			
ASV_397	Firmicutes;D_2__Clostridia;D_3__Clostridiales;D_4__Family XIII;D_5__Family XIII AD3011 group;D_6__uncultured organism;D_7__;D_8__;D_9__;D_10__;D_11__;D_12__;D_13__;D_14__	0.17(0.03,0.96);p=0.04471	1.52(0.29,8.06)	0.25(0.06,1.03)	0.14(0.02,0.83);p=0.03029	1.19(0.23,6.19)	0.17(0.04,0.69);p=0.01356	↓		↓
ASV_399	Firmicutes;D_2__Clostridia;D_3__Clostridiales;D_4__Ruminococcaceae;D_5__Ruminiclostridium;D_6__uncultured bacterium;D_7__;D_8__;D_9__;D_10__;D_11__;D_12__;D_13__;D_14__				465740268329141.00(0.00,Inf)	0.00(0.00,Inf)	0.52(0.04,7.62)			
ASV_401	Firmicutes;D_2__Clostridia;D_3__Clostridiales;D_4__Ruminococcaceae;D_5__uncultured;D_6__uncultured Ruminococcus sp.;D_7__;D_8__;D_9__;D_10__;D_11__;D_12__;D_13__;D_14__	1.24(0.34,4.57)	0.61(0.17,2.11)	0.75(0.27,2.10)	1.32(0.36,4.86)	0.68(0.20,2.35)	0.90(0.32,2.51)			
ASV_405	Actinobacteria;D_2__Actinobacteria;D_3__Actinomycetales;D_4__Actinomycetaceae;D_5__Actinomyces	0.39(0.09,1.65)	4.04(1.00,16.34);p=0.04998	1.59(0.47,5.38)	0.45(0.11,1.88)	3.95(0.97,16.12)	1.78(0.54,5.92)			
ASV_407	Firmicutes;D_2__Clostridia;D_3__Clostridiales;D_4__Peptococcaceae;D_5__Peptococcus;D_6__uncultured organism;D_7__;D_8__;D_9__;D_10__;D_11__;D_12__;D_13__;D_14__	6025599856.50(0.00,Inf)	0.00(0.00,Inf)	0.96(0.08,11.85)						
ASV_410	Firmicutes;D_2__Clostridia;D_3__Clostridiales;D_4__Lachnospiraceae;D_5__Anaerosporobacter;D_6__uncultured organism;D_7__;D_8__;D_9__;D_10__;D_11__;D_12__;D_13__;D_14__	11.90(1.41,100.59);p=0.02295	0.14(0.02,1.11)	1.69(0.32,8.87)	13.21(1.53,114.39);p=0.01911	0.09(0.01,0.75);p=0.02515	1.25(0.24,6.54)	↑	↓	
ASV_411	Firmicutes;D_2__Clostridia;D_3__Clostridiales;D_4__Ruminococcaceae;D_5__Ruminiclostridium 9;D_6__uncultured bacterium;D_7__;D_8__;D_9__;D_10__;D_11__;D_12__;D_13__;D_14__	1.96(0.06,68.21)	0.31(0.01,9.35)	0.61(0.04,9.52)						
ASV_413	Firmicutes;D_2__Clostridia;D_3__Clostridiales;D_4__Lachnospiraceae;D_5__Lachnospiraceae UCG-010;D_6__uncultured bacterium;D_7__;D_8__;D_9__;D_10__;D_11__;D_12__;D_13__;D_14__	0.48(0.10,2.26)	1.83(0.42,8.06)	0.88(0.25,3.05)						
ASV_414	Firmicutes;D_2__Clostridia;D_3__Clostridiales;D_4__Ruminococcaceae;D_5__GCA-900066225;D_6__uncultured bacterium;D_7__;D_8__;D_9__;D_10__;D_11__;D_12__;D_13__;D_14__	0.68(0.02,18.56)	0.35(0.02,7.95)	0.24(0.02,3.17)						
ASV_415	Firmicutes;D_2__Clostridia;D_3__Clostridiales;D_4__Ruminococcaceae;D_5__Pseudoflavonifractor;D_6__uncultured bacterium;D_7__;D_8__;D_9__;D_10__;D_11__;D_12__;D_13__;D_14__	0.33(0.03,3.50)	1.24(0.13,11.72)	0.40(0.06,2.69)	0.96(0.10,9.74)	0.38(0.04,3.35)	0.37(0.06,2.17)			
ASV_417	Firmicutes;D_2__Clostridia;D_3__Clostridiales;D_4__Christensenellaceae;D_5__Christensenellaceae R-7 group	39328732456.55(0.00,Inf)	0.00(0.00,Inf)	58.85(1.50,2310.66);p=0.02955						
ASV_418	Firmicutes;D_2__Clostridia;D_3__Clostridiales;D_4__Christensenellaceae;D_5__uncultured;D_6__uncultured bacterium;D_7__;D_8__;D_9__;D_10__;D_11__;D_12__;D_13__;D_14__	0.36(0.01,10.78)	1.31(0.06,30.14)	0.47(0.03,7.51)						
ASV_420	Firmicutes;D_2__Clostridia;D_3__Clostridiales;D_4__Ruminococcaceae;D_5__Fournierella;D_6__uncultured organism;D_7__;D_8__;D_9__;D_10__;D_11__;D_12__;D_13__;D_14__	0.51(0.07,3.60)	0.35(0.06,2.15)	0.18(0.04,0.82);p=0.02732	0.33(0.05,2.21)	0.61(0.11,3.45)	0.20(0.04,0.91);p=0.03754			↓
ASV_423	Firmicutes;D_2__Clostridia;D_3__Clostridiales;D_4__Lachnospiraceae;D_5__Lachnoclostridium	0.78(0.19,3.28)	2.08(0.52,8.33)	1.63(0.51,5.15)	0.81(0.20,3.35)	2.72(0.70,10.63)	2.21(0.70,6.97)			
ASV_424	Firmicutes;D_2__Clostridia;D_3__Clostridiales;D_4__Lachnospiraceae	0.52(0.04,5.94)	1.01(0.10,10.60)	0.52(0.08,3.64)						
ASV_426	Firmicutes;D_2__Clostridia;D_3__Clostridiales;D_4__Lachnospiraceae;D_5__[Ruminococcus] torques group	1.08(0.20,5.79)	0.43(0.09,2.14)	0.46(0.13,1.71)	0.99(0.19,5.20)	0.60(0.13,2.89)	0.59(0.16,2.17)			
ASV_428	Firmicutes;D_2__Clostridia;D_3__Clostridiales;D_4__Lachnospiraceae;D_5__Lachnoclostridium	6.11(0.28,131.33)	0.15(0.01,2.97)	0.94(0.09,10.18)						
ASV_429	Firmicutes;D_2__Clostridia;D_3__Clostridiales;D_4__Ruminococcaceae;D_5__uncultured;D_6__uncultured Clostridium sp.;D_7__;D_8__;D_9__;D_10__;D_11__;D_12__;D_13__;D_14__	2.64(0.30,23.64)	0.68(0.08,5.63)	1.78(0.33,9.77)	1.04(0.13,8.62)	0.82(0.11,6.15)	0.86(0.17,4.43)			

ASV_430	Firmicutes;D_2__Clostridia;D_3__Clostridiales;D_4__Ruminococcaceae;D_5__Ruminococcaceae UCG-013;D_6__uncultured Clostridiaceae bacterium;D_7__;D_8__;D_9__;D_10__;D_11__;D_12__;D_13__;D_14__				0.86(0.05,14.12)	1.60(0.11,23.55)	1.37(0.15,12.63)			
ASV_431	Firmicutes;D_2__Clostridia;D_3__Clostridiales;D_4__Ruminococcaceae	0.71(0.17,2.92)	0.84(0.22,3.24)	0.60(0.19,1.83)	0.66(0.16,2.68)	0.97(0.26,3.67)	0.64(0.21,1.94)			
ASV_432	Firmicutes;D_2__Erysipelotrichia;D_3__Erysipelotrichales;D_4__Erysipelotrichaceae;D_5__Holdemania	0.16(0.03,0.90);p=0.03689	1.38(0.28,6.74)	0.23(0.06,0.89);p=0.03322	0.17(0.03,0.93);p=0.04079	1.32(0.27,6.37)	0.22(0.06,0.88);p=0.03243	↓		↓
ASV_434	Firmicutes;D_2__Clostridia;D_3__Clostridiales;D_4__Ruminococcaceae	0.58(0.02,19.40)	0.62(0.02,15.41)	0.36(0.02,5.59)	0.18(0.01,5.71)	1.85(0.09,38.63)	0.33(0.02,5.87)			
ASV_437	Firmicutes;D_2__Clostridia;D_3__Clostridiales;D_4__Ruminococcaceae;D_5__Candidatus Soleaferrea;D_6__uncultured bacterium;D_7__;D_8__;D_9__;D_10__;D_11__;D_12__;D_13__;D_14__	6.26(0.64,60.98)	0.10(0.01,0.87);p=0.03747	0.60(0.13,2.91)	5.81(0.58,58.43)	0.09(0.01,0.85);p=0.03516	0.54(0.11,2.62)		↓	
ASV_438	Proteobacteria;D_2__Gammaproteobacteria;D_3__Betaproteobacteriales;D_4__Burkholderiaceae;D_5__Noviherbaspirillum	1152761066727.39(0.00,Inf)	0.00(0.00,Inf)	0.94(0.14,6.25)	2514116052066608.00(0.00,Inf)	0.00(0.00,Inf)	0.95(0.14,6.38)			
ASV_440	Firmicutes;D_2__Clostridia;D_3__Clostridiales;D_4__Ruminococcaceae;D_5__Ruminococcaceae UCG-010;D_6__uncultured bacterium;D_7__;D_8__;D_9__;D_10__;D_11__;D_12__;D_13__;D_14__	0.72(0.02,23.48)	0.48(0.02,12.69)	0.35(0.02,5.22)						
ASV_441	Firmicutes;D_2__Clostridia;D_3__Clostridiales;D_4__Lachnospiraceae;D_5__Blautia	0.00(0.00,Inf)	6.25(0.28,137.32)	0.00(0.00,Inf)						
ASV_442	Firmicutes;D_2__Clostridia;D_3__Clostridiales;D_4__Ruminococcaceae;D_5__uncultured	1396577614.65(0.00,Inf)	0.00(0.00,Inf)	0.43(0.06,3.17)	507446747475005.00(0.00,Inf)	0.00(0.00,Inf)	0.27(0.03,2.12)			
ASV_448	Actinobacteria;D_2__Actinobacteria;D_3__Bifidobacteriales;D_4__Bifidobacteriaceae;D_5__Bifidobacterium	1.52(0.28,8.22)	0.47(0.09,2.39)	0.72(0.19,2.74)	1.61(0.29,8.81)	0.50(0.10,2.53)	0.81(0.21,3.10)			
ASV_453	Firmicutes;D_2__Clostridia;D_3__Clostridiales;D_4__Lachnospiraceae;D_5__Anaerostipes	0.83(0.15,4.47)	0.67(0.13,3.37)	0.56(0.15,2.11)	0.53(0.10,2.81)	0.71(0.14,3.44)	0.38(0.10,1.40)			
ASV_456	Firmicutes;D_2__Clostridia;D_3__Clostridiales;D_4__Peptostreptococcaceae;D_5__Intestinibacter	1.12(0.47,2.65)	0.55(0.24,1.25)	0.61(0.31,1.21)	1.34(0.60,3.04)	0.64(0.30,1.40)	0.86(0.45,1.64)			
ASV_457	Firmicutes;D_2__Clostridia;D_3__Clostridiales;D_4__Lachnospiraceae;D_5__uncultured	2.81(0.21,37.24)	2.01(0.16,24.59)	5.65(0.72,44.18)	1.34(0.11,17.01)	3.61(0.31,41.71)	4.82(0.63,36.86)			
ASV_460	Firmicutes;D_2__Clostridia;D_3__Clostridiales;D_4__Lachnospiraceae	0.53(0.11,2.55)	2.56(0.57,11.61)	1.36(0.39,4.76)	0.90(0.19,4.37)	1.94(0.43,8.74)	1.76(0.50,6.14)			
ASV_462	Firmicutes;D_2__Clostridia;D_3__Clostridiales;D_4__Lachnospiraceae;D_5__uncultured;D_6__uncultured bacterium adhufec382;D_7__;D_8__;D_9__;D_10__;D_11__;D_12__;D_13__;D_14__	1.71(0.24,12.19)	0.97(0.14,6.45)	1.65(0.35,7.75)	1.97(0.27,14.16)	0.74(0.11,4.90)	1.46(0.32,6.76)			
ASV_465	Firmicutes;D_2__Bacilli;D_3__Lactobacillales;D_4__Lactobacillaceae;D_5__Lactobacillus	23707091480.34(0.00,Inf)	0.00(0.00,Inf)	3.68(0.49,27.42)	22255804037493144.00(0.00,Inf)	0.00(0.00,Inf)	5.63(0.77,41.24)			
ASV_466	Firmicutes;D_2__Clostridia;D_3__Clostridiales;D_4__Lachnospiraceae;D_5__uncultured	0.99(0.04,24.04)	14.93(0.52,431.99)	14.71(0.83,262.05)						
ASV_467	Firmicutes;D_2__Bacilli;D_3__Lactobacillales;D_4__Lactobacillaceae;D_5__Lactobacillus	2.19(0.23,20.65)	0.15(0.02,1.27)	0.32(0.06,1.79)	1.88(0.20,17.89)	0.15(0.02,1.27)	0.28(0.05,1.55)			
ASV_468	Firmicutes;D_2__Clostridia;D_3__Clostridiales;D_4__Lachnospiraceae;D_5__uncultured;D_6__metagenome;D_7__;D_8__;D_9__;D_10__;D_11__;D_12__;D_13__;D_14__	4.30(0.21,86.93)	0.77(0.04,14.86)	3.31(0.34,32.02)	11.87(0.33,428.60)	0.29(0.01,10.17)	3.41(0.35,33.21)			
ASV_471	Firmicutes;D_2__Bacilli;D_3__Lactobacillales;D_4__Streptococcaceae;D_5__Streptococcus	2.23(0.50,9.93)	1.05(0.25,4.46)	2.34(0.72,7.58)	2.46(0.55,10.98)	1.08(0.25,4.57)	2.66(0.82,8.57)			
ASV_472	Firmicutes;D_2__Erysipelotrichia;D_3__Erysipelotrichales;D_4__Erysipelotrichaceae;D_5__Dielma	1.03(0.24,4.47)	2.17(0.52,8.98)	2.23(0.69,7.21)	1.03(0.24,4.41)	2.10(0.52,8.51)	2.15(0.67,6.88)			
ASV_474	Firmicutes;D_2__Bacilli;D_3__Lactobacillales;D_4__Leuconostocaceae;D_5__Leuconostoc	0.10(0.01,1.23)	8.42(0.77,92.71)	0.85(0.11,6.46)						
ASV_475	Firmicutes;D_2__Negativicutes;D_3__Selenomonadales;D_4__Veillonellaceae;D_5__Veillonella	4.37(0.36,53.40)	0.37(0.03,4.12)	1.61(0.23,11.43)	3.55(0.29,43.75)	0.42(0.04,4.58)	1.48(0.21,10.54)			
ASV_476	Cyanobacteria;D_2__Oxyphotobacteria;D_3__Chloroplast	6254265621.12(0.00,Inf)	0.00(0.00,Inf)	12.62(0.99,160.41)						
ASV_477	Firmicutes;D_2__Clostridia;D_3__Clostridiales;D_4__Peptostreptococcaceae;D_5__Terrisporobacter;D_6__uncultured bacterium;D_7__;D_8__;D_9__;D_10__;D_11__;D_12__;D_13__;D_14__	3.22(0.84,12.36)	0.26(0.07,0.96);p=0.04243	0.84(0.29,2.43)	3.28(0.89,12.13)	0.47(0.14,1.64)	1.54(0.55,4.33)			
ASV_481	Proteobacteria;D_2__Alphaproteobacteria;D_3__Caulobacterales;D_4__Caulobacteraceae;D_5__Brevundimonas	1.80(0.11,29.80)	0.29(0.02,4.13)	0.52(0.07,3.89)	1.92(0.12,31.27)	0.33(0.02,4.57)	0.63(0.08,4.71)			

ASV_483	Firmicutes;D_2__Bacilli;D_3__Lactobacillales;D_4__Streptococcaceae;D_5__Lactococcus	16.04(0.00,Inf)	0.15(0.00,Inf)	2.37(0.00,Inf)						
ASV_485	Actinobacteria;D_2__Actinobacteria;D_3__Micrococcales;D_4__Micrococcaceae;D_5__Rothia;D_6__uncultured organism;D_7__;D_8__;D_9__;D_10__;D_11__;D_12__;D_13__;D_14__	0.35(0.05,2.65)	12.59(1.33,119.26);p=0.02725	4.38(0.55,34.85)	0.35(0.04,2.80)	13.78(1.39,136.78); p=0.02512	4.82(0.58,39.83)		↑	
ASV_488	Proteobacteria;D_2__Gammaproteobacteria;D_3__Pasteurellales;D_4__Pasteurellaceae	0.55(0.05,6.55)	0.53(0.05,5.60)	0.29(0.04,2.07)	1.42(0.13,15.47)	0.45(0.05,4.32)	0.64(0.10,4.12)			
ASV_489	Actinobacteria;D_2__Actinobacteria;D_3__Actinomycetales;D_4__Actinomycetaceae;D_5__Actinomyces;D_6__Actinomyces graevenitzii F0530;D_7__;D_8__;D_9__;D_10__;D_11__;D_12__;D_13__;D_14__	1.27(0.33,4.94)	0.91(0.25,3.37)	1.16(0.41,3.34)	1.27(0.32,4.98)	0.93(0.25,3.44)	1.18(0.41,3.40)			
ASV_490	Firmicutes;D_2__Clostridia;D_3__Clostridiales;D_4__Lachnospiraceae;D_5__[Ruminococcus] gnavus group	1.00(0.25,3.97)	1.49(0.40,5.61)	1.49(0.50,4.46)	0.69(0.17,2.77)	2.11(0.57,7.87)	1.47(0.49,4.38)			
ASV_491	Proteobacteria;D_2__Alphaproteobacteria;D_3__Sphingomonadales;D_4__Sphingomonadaceae;D_5__Sphingomonas	563408360778.49(0.00,Inf)	0.00(0.00,Inf)	0.53(0.03,9.61)	19779986507764.20(0.00,Inf)	0.00(0.00,Inf)	0.61(0.04,10.01)			
ASV_493	Actinobacteria;D_2__Actinobacteria;D_3__Micrococcales;D_4__Micrococcaceae;D_5__Rothia;D_6__uncultured bacterium;D_7__;D_8__;D_9__;D_10__;D_11__;D_12__;D_13__;D_14__	0.39(0.01,11.40)	1.48(0.07,31.52)	0.58(0.03,9.63)	0.70(0.02,24.81)	1.10(0.04,32.00)	0.77(0.05,11.77)			
ASV_502	Bacteroidetes;D_2__Bacteroidia;D_3__Bacteroidales;D_4__Bacteroidaceae;D_5__Bacteroides;D_6__Bacteroides fragilis;D_7__;D_8__;D_9__;D_10__;D_11__;D_12__;D_13__;D_14__	0.55(0.05,6.11)	0.44(0.04,4.47)	0.24(0.04,1.65)	0.42(0.04,4.76)	0.65(0.06,6.54)	0.28(0.04,1.87)			
ASV_503	Proteobacteria;D_2__Gammaproteobacteria;D_3__Enterobacteriales;D_4__Enterobacteriaceae	0.24(0.02,3.07)	1.75(0.15,20.46)	0.42(0.05,3.18)	0.29(0.02,3.74)	1.67(0.15,19.25)	0.48(0.06,3.67)			
ASV_504	Firmicutes;D_2__Erysipelotrichia;D_3__Erysipelotrichales;D_4__Erysipelotrichaceae;D_5__Turicibacter	2.88(1.00,8.27);p=0.04994	0.59(0.21,1.62)	1.68(0.73,3.89)	3.14(1.09,9.06);p=0.03439	0.60(0.22,1.66)	1.90(0.82,4.39)	↑		
ASV_506	Firmicutes;D_2__Clostridia;D_3__Clostridiales;D_4__Lachnospiraceae	5765413750.33(0.00,Inf)	0.00(0.00,Inf)	0.53(0.09,3.08)	24996370088630264.00(0.00,Inf)	0.00(0.00,Inf)	0.43(0.07,2.51)			
ASV_508	Firmicutes;D_2__Clostridia;D_3__Clostridiales;D_4__Ruminococcaceae;D_5__Ruminococcaceae UCG-005	0.76(0.13,4.28)	0.51(0.10,2.68)	0.39(0.10,1.52)	0.69(0.13,3.81)	0.56(0.11,2.86)	0.39(0.10,1.50)			
ASV_510	Firmicutes;D_2__Clostridia;D_3__Clostridiales;D_4__Lachnospiraceae;D_5__Blautia	53.09(1.20,2355.79);p=0.04012	0.00(0.00,0.11);p=0.0018	0.15(0.01,1.83)						
ASV_512	Firmicutes;D_2__Clostridia;D_3__Clostridiales;D_4__Lachnospiraceae;D_5__Lachnoclostridium	0.23(0.02,2.71)	1.99(0.18,21.49)	0.45(0.06,3.24)	0.13(0.01,1.47)	4.06(0.41,39.82)	0.53(0.08,3.65)			
ASV_513	Bacteroidetes;D_2__Bacteroidia;D_3__Bacteroidales;D_4__Marinifilaceae;D_5__Butyricimonas;D_6__uncultured bacterium;D_7__;D_8__;D_9__;D_10__;D_11__;D_12__;D_13__;D_14__	0.03(0.00,2.09)	0.72(0.02,25.36)	0.02(0.00,0.78);p=0.03633						
ASV_515	Firmicutes;D_2__Clostridia;D_3__Clostridiales;D_4__Ruminococcaceae;D_5__Ruminiclostridium 9	42.25(1.14,1569.07);p=0.04236	0.05(0.00,1.52)	1.98(0.12,33.60)						
ASV_516	Firmicutes;D_2__Clostridia;D_3__Clostridiales;D_4__Ruminococcaceae;D_5__Negativibacillus;D_6__uncultured bacterium;D_7__;D_8__;D_9__;D_10__;D_11__;D_12__;D_13__;D_14__	10.89(0.62,191.73)	0.22(0.01,3.48)	2.39(0.26,22.14)	1.16(0.07,19.91)	0.30(0.02,4.55)	0.35(0.04,3.23)			
ASV_518	Firmicutes;D_2__Clostridia;D_3__Clostridiales;D_4__Peptostreptococcaceae;D_5__Romboutsia	64.28(2.88,1432.74);p=0.00857	0.09(0.00,1.72)	5.49(0.55,54.96)	54.71(2.45,1223.27); p=0.01159	0.09(0.00,1.85)	5.14(0.51,52.16)	↑		
ASV_522	Firmicutes;D_2__Clostridia;D_3__Clostridiales;D_4__Family XIII;D_5__Family XIII UCG-001;D_6__uncultured bacterium;D_7__;D_8__;D_9__;D_10__;D_11__;D_12__;D_13__;D_14__	0.55(0.14,2.26)	0.87(0.22,3.34)	0.48(0.16,1.47)						
ASV_524	Firmicutes;D_2__Clostridia;D_3__Clostridiales;D_4__Ruminococcaceae;D_5__Butyricicoccus	1.12(0.21,6.08)	1.28(0.25,6.51)	1.43(0.37,5.49)						
ASV_526	Firmicutes;D_2__Clostridia;D_3__Clostridiales;D_4__Lachnospiraceae;D_5__uncultured;D_6__metagenome;D_7__;D_8__;D_9__;D_10__;D_11__;D_12__;D_13__;D_14__	0.84(0.18,3.86)	2.91(0.66,12.75)	2.44(0.72,8.31)	0.35(0.08,1.58)	3.90(0.92,16.50)	1.37(0.41,4.60)			
ASV_529	Firmicutes;D_2__Clostridia;D_3__Clostridiales;D_4__Ruminococcaceae;D_5__Ruminiclostridium 9	0.74(0.10,5.47)	2.78(0.41,18.94)	2.06(0.42,10.09)	0.73(0.10,5.49)	2.78(0.41,18.99)	2.03(0.41,10.04)			
ASV_531	Firmicutes;D_2__Clostridia;D_3__Clostridiales;D_4__Lachnospiraceae;D_5__Lachnoclostridium	1.95(0.06,63.00)	0.70(0.02,19.86)	1.36(0.09,20.78)	2.05(0.06,67.75)	0.84(0.03,23.93)	1.72(0.11,26.67)			
ASV_533	Firmicutes;D_2__Clostridia;D_3__Clostridiales;D_4__Lachnospiraceae;D_5__Marvinbryantia	2.35(0.50,11.01)	0.38(0.09,1.69)	0.90(0.26,3.05)	2.24(0.47,10.64)	0.35(0.08,1.53)	0.78(0.23,2.66)			
ASV_536	Firmicutes;D_2__Clostridia;D_3__Clostridiales;D_4__Clostridiaceae 1;D_5__Clostridium sensu stricto 1	2.50(0.80,7.81)	0.79(0.26,2.35)	1.97(0.80,4.85)	2.77(0.88,8.70)	0.75(0.25,2.25)	2.09(0.84,5.16)			

ASV_538	Firmicutes;D_2__Clostridia;D_3__Clostridiales;D_4__Ruminococcaceae;D_5__Ruminiclostridium 5;D_6__uncultured organism;D_7__;D_8__;D_9__;D_10__;D_11__;D_12__;D_13__;D_14__	12.58(0.72,219.18)	2.38(0.15,37.20)	29.90(3.09,289.26);p=0.00334						
ASV_539	Firmicutes;D_2__Clostridia;D_3__Clostridiales;D_4__Lachnospiraceae;D_5__[Eubacterium] ventriosum group	0.93(0.08,10.42)	1.36(0.13,13.89)	1.27(0.19,8.62)	0.99(0.09,11.18)	1.39(0.14,14.04)	1.37(0.20,9.38)			
ASV_545	Firmicutes;D_2__Clostridia;D_3__Clostridiales;D_4__Lachnospiraceae;D_5__[Eubacterium] ventriosum group;D_6__uncultured bacterium;D_7__;D_8__;D_9__;D_10__;D_11__;D_12__;D_13__;D_14__	1.28(0.05,34.84)	0.05(0.00,1.20)	0.07(0.01,0.85);p=0.03722						
ASV_547	Firmicutes;D_2__Clostridia;D_3__Clostridiales;D_4__Ruminococcaceae;D_5__Oscillibacter;D_6__Oscillibacter sp. Marseille-P3260;D_7__;D_8__;D_9__;D_10__;D_11__;D_12__;D_13__;D_14__	0.53(0.05,5.88)	0.58(0.06,5.46)	0.31(0.05,2.07)	0.53(0.05,5.94)	0.59(0.06,5.51)	0.31(0.05,2.08)			
ASV_549	Firmicutes;D_2__Clostridia;D_3__Clostridiales;D_4__Lachnospiraceae	1.67(0.44,6.29)	0.77(0.22,2.76)	1.29(0.45,3.67)	1.49(0.40,5.59)	1.02(0.29,3.62)	1.53(0.54,4.33)			
ASV_551	Firmicutes;D_2__Clostridia;D_3__Clostridiales;D_4__Lachnospiraceae;D_5__[Eubacterium] xylanophilum group	0.94(0.19,4.69)	1.16(0.25,5.44)	1.10(0.31,3.92)	0.94(0.19,4.74)	1.25(0.27,5.86)	1.17(0.32,4.23)			
ASV_556	Firmicutes;D_2__Bacilli;D_3__Lactobacillales;D_4__Leuconostocaceae;D_5__Weissella;D_6__Weissella paramesenteroides;D_7__;D_8__;D_9__;D_10__;D_11__;D_12__;D_13__;D_14__	0.00(0.00,Inf)	4.76(0.11,212.52)	0.00(0.00,Inf)						
ASV_557	Firmicutes;D_2__Clostridia;D_3__Clostridiales;D_4__Christensenellaceae;D_5__uncultured;D_6__uncultured bacterium;D_7__;D_8__;D_9__;D_10__;D_11__;D_12__;D_13__;D_14__	1.10(0.28,4.33)	1.61(0.43,6.04)	1.76(0.59,5.26)	0.91(0.23,3.54)	1.27(0.35,4.63)	1.16(0.40,3.38)			
ASV_559	Firmicutes;D_2__Clostridia;D_3__Clostridiales;D_4__Ruminococcaceae;D_5__Anaerofilum;D_6__uncultured bacterium;D_7__;D_8__;D_9__;D_10__;D_11__;D_12__;D_13__;D_14__	15535837132.39(0.00,Inf)	0.00(0.00,Inf)	0.42(0.01,12.54)						
ASV_560	Firmicutes;D_2__Clostridia;D_3__Clostridiales;D_4__Lachnospiraceae;D_5__Anaerostipes;D_6__unidentified;D_7__;D_8__;D_9__;D_10__;D_11__;D_12__;D_13__;D_14__	1.78(0.18,17.46)	0.18(0.02,1.60)	0.32(0.05,1.85)	1.63(0.17,15.96)	0.21(0.02,1.81)	0.34(0.06,1.96)			
ASV_561	Actinobacteria;D_2__Coriobacteriia;D_3__Coriobacteriales;D_4__Coriobacteriales Incertae Sedis;D_5__Raoultibacter;D_6__Raoultibacter timonensis;D_7__;D_8__;D_9__;D_10__;D_11__;D_12__;D_13__;D_14__	0.28(0.01,7.25)	1.15(0.06,21.99)	0.32(0.02,4.56)	0.11(0.00,4.08)	1.17(0.05,26.35)	0.13(0.01,2.19)			
ASV_565	Firmicutes;D_2__Clostridia;D_3__Clostridiales;D_4__Ruminococcaceae;D_5__Oscillibacter;D_6__uncultured bacterium;D_7__;D_8__;D_9__;D_10__;D_11__;D_12__;D_13__;D_14__				25149396734439980.00(0.00,Inf)	0.00(0.00,Inf)	4.36(0.75,25.44)			
ASV_566	Firmicutes;D_2__Clostridia;D_3__Clostridiales;D_4__Ruminococcaceae	1.46(0.13,16.87)	0.17(0.02,1.80)	0.25(0.04,1.74)	1.49(0.14,16.30)	0.39(0.04,3.83)	0.59(0.09,3.87)			
ASV_571	Firmicutes;D_2__Clostridia;D_3__Clostridiales;D_4__Lachnospiraceae	0.41(0.01,12.48)	2.97(0.11,79.50)	1.21(0.08,18.41)						
ASV_572	Firmicutes;D_2__Clostridia;D_3__Clostridiales;D_4__Christensenellaceae;D_5__Christensenellaceae R-7 group;D_6__uncultured marine bacterium;D_7__;D_8__;D_9__;D_10__;D_11__;D_12__;D_13__;D_14__	1.11(0.35,3.50)	1.58(0.52,4.80)	1.76(0.71,4.38)	0.99(0.33,2.99)	1.78(0.62,5.10)	1.76(0.73,4.24)			
ASV_573	Firmicutes;D_2__Bacilli;D_3__Lactobacillales;D_4__Streptococcaceae;D_5__Streptococcus;D_6__Streptococcus mutans;D_7__;D_8__;D_9__;D_10__;D_11__;D_12__;D_13__;D_14__	2.48(0.64,9.71)	2.21(0.58,8.35)	5.49(1.85,16.31);p=0.00219	1.85(0.49,7.00)	2.38(0.66,8.67)	4.41(1.52,12.76);p=0.00618			↑
ASV_577	Firmicutes;D_2__Clostridia;D_3__Clostridiales;D_4__Eubacteriaceae;D_5__Eubacterium;D_6__Eubacterium limosum;D_7__;D_8__;D_9__;D_10__;D_11__;D_12__;D_13__;D_14__	1.21(0.13,11.54)	1.88(0.21,16.63)	2.27(0.38,13.69)	1.11(0.12,10.74)	1.58(0.18,13.74)	1.76(0.29,10.62)			
ASV_581	Firmicutes;D_2__Erysipelotrichia;D_3__Erysipelotrichales;D_4__Erysipelotrichaceae;D_5__Solobacterium	3.11(0.24,40.24)	0.03(0.00,0.40);p=0.00701	0.11(0.02,0.72);p=0.02122						
ASV_582	Firmicutes;D_2__Clostridia;D_3__Clostridiales;D_4__Family XIII;D_5__Family XIII AD3011 group;D_6__gut metagenome;D_7__;D_8__;D_9__;D_10__;D_11__;D_12__;D_13__;D_14__				1530100808289539.00(0.00,Inf)	0.00(0.00,Inf)	1.32(0.09,20.08)			
ASV_583	Firmicutes;D_2__Bacilli;D_3__Lactobacillales;D_4__Lactobacillaceae;D_5__Lactobacillus;D_6__Lactobacillus helveticus;D_7__;D_8__;D_9__;D_10__;D_11__;D_12__;D_13__;D_14__				0.69(0.00,Inf)	0.00(0.00,Inf)	0.00(0.00,Inf)			
ASV_586	Firmicutes;D_2__Clostridia;D_3__Clostridiales;D_4__Ruminococcaceae;D_5__Anaerotruncus;D_6__uncultured organism;D_7__;D_8__;D_9__;D_10__;D_11__;D_12__;D_13__;D_14__	2.22(0.60,8.18)	0.30(0.08,1.04)	0.66(0.24,1.78)	2.57(0.69,9.55)	0.30(0.09,1.06)	0.78(0.29,2.10)			
ASV_588	Firmicutes;D_2__Erysipelotrichia;D_3__Erysipelotrichales;D_4__Erysipelotrichaceae;D_5__uncultured;D_6__Clostridiales bacterium 60-7e;D_7__;D_8__;D_9__;D_10__;D_11__;D_12__;D_13__;D_14__	1.64(0.10,25.96)	2.26(0.16,32.18)	3.71(0.41,33.25)	1.43(0.09,21.84)	3.10(0.23,41.87)	4.43(0.51,38.70)			

ASV_589	Proteobacteria;D_2__Gammaproteobacteria;D_3__Betaproteobacteriales;D_4__Burkholderiaceae;D_5__Variovorax	5.45(0.94,31.62)	0.23(0.04,1.28)	1.27(0.37,4.37)	5.16(0.88,30.11)	0.25(0.05,1.37)	1.28(0.37,4.43)			
ASV_590	Firmicutes;D_2__Clostridia;D_3__Clostridiales;D_4__Lachnospiraceae	0.14(0.01,1.45)	0.94(0.14,6.34)	0.13(0.02,0.95);p=0.04417	0.10(0.01,1.24)	0.85(0.13,5.61)	0.08(0.01,0.78);p=0.02922			↓
ASV_591	Firmicutes;D_2__Clostridia;D_3__Clostridiales;D_4__Clostridiales vadinBB60 group;D_5__gut metagenome;D_6__;D_7__;D_8__;D_9__;D_10__;D_11__;D_12__;D_13__;D_14__	3.08(0.27,35.40)	0.21(0.02,2.22)	0.66(0.10,4.40)						
ASV_594	Firmicutes;D_2__Clostridia;D_3__Clostridiales;D_4__Ruminococcaceae;D_5__Candidatus Soleaferrea;D_6__Bittarella massiliensis;D_7__;D_8__;D_9__;D_10__;D_11__;D_12__;D_13__;D_14__	0.64(0.26,1.59)	1.13(0.47,2.70)	0.72(0.35,1.50)	0.64(0.25,1.61)	1.11(0.46,2.65)	0.71(0.34,1.47)			
ASV_595	Firmicutes;D_2__Clostridia;D_3__Clostridiales;D_4__Lachnospiraceae	0.81(0.06,11.78)	5.37(0.39,73.96)	4.34(0.49,38.62)	0.53(0.04,7.05)	9.62(0.73,126.10)	5.14(0.59,45.11)			
ASV_596	Firmicutes;D_2__Clostridia;D_3__Clostridiales;D_4__Lachnospiraceae;D_5__[Eubacterium] fissicatena group	1.06(0.32,3.52)	0.62(0.20,1.96)	0.66(0.26,1.69)	1.03(0.31,3.45)	0.66(0.21,2.06)	0.68(0.26,1.74)			
ASV_597	Firmicutes;D_2__Clostridia;D_3__Clostridiales;D_4__Lachnospiraceae;D_5__Lachnoclostridium;D_6__[Clostridium] scindens;D_7__;D_8__;D_9__;D_10__;D_11__;D_12__;D_13__;D_14__	1.29(0.21,8.05)	1.13(0.19,6.58)	1.46(0.34,6.23)	1.04(0.17,6.50)	1.23(0.21,7.04)	1.28(0.30,5.46)			
ASV_598	Firmicutes;D_2__Clostridia;D_3__Clostridiales;D_4__Lachnospiraceae;D_5__[Ruminococcus] torques group;D_6__uncultured Clostridiales bacterium;D_7__;D_8__;D_9__;D_10__;D_11__;D_12__;D_13__;D_14__	0.88(0.13,5.73)	1.84(0.30,11.27)	1.61(0.36,7.23)	0.83(0.12,5.46)	1.55(0.26,9.42)	1.28(0.29,5.76)			
ASV_599	Firmicutes;D_2__Clostridia;D_3__Clostridiales;D_4__Ruminococcaceae;D_5__Ruminiclostridium 9	2.18(0.67,7.07)	0.67(0.21,2.07)	1.45(0.57,3.67)	2.15(0.65,7.08)	0.67(0.21,2.08)	1.44(0.56,3.66)			
ASV_600	Firmicutes;D_2__Clostridia;D_3__Clostridiales;D_4__Lachnospiraceae;D_5__UC5-1-2E3;D_6__uncultured bacterium;D_7__;D_8__;D_9__;D_10__;D_11__;D_12__;D_13__;D_14__				1.82(0.11,29.29)	0.76(0.05,10.95)	1.39(0.16,11.76)			
ASV_601	Firmicutes;D_2__Clostridia;D_3__Clostridiales;D_4__Lachnospiraceae				1.07(0.21,5.54)	1.10(0.23,5.26)	1.18(0.32,4.30)			
ASV_611	Firmicutes;D_2__Clostridia;D_3__Clostridiales;D_4__Lachnospiraceae;D_5__uncultured	0.89(0.08,9.38)	1.00(0.10,9.63)	0.89(0.14,5.78)	0.86(0.08,9.26)	0.91(0.09,8.80)	0.78(0.12,5.16)			
ASV_613	Firmicutes;D_2__Erysipelotrichia;D_3__Erysipelotrichales;D_4__Erysipelotrichaceae;D_5__Turicibacter	0.44(0.05,3.53)	2.28(0.31,16.87)	1.00(0.19,5.24)	1.09(0.15,7.93)	1.85(0.28,12.29)	2.00(0.41,9.71)			
ASV_614	Firmicutes;D_2__Clostridia;D_3__Clostridiales;D_4__Ruminococcaceae;D_5__Ruminococcaceae UCG-005;D_6__metagenome;D_7__;D_8__;D_9__;D_10__;D_11__;D_12__;D_13__;D_14__	1.91(0.03,124.59)	22.71(0.32,1605.51)	43.33(1.19,1571.44);p=0.03967						
ASV_621	Firmicutes;D_2__Clostridia;D_3__Clostridiales;D_4__Ruminococcaceae;D_5__Intestinimonas;D_6__Intestinimonas butyriciproducens;D_7__;D_8__;D_9__;D_10__;D_11__;D_12__;D_13__;D_14__	2.40(0.42,13.66)	0.57(0.11,3.06)	1.38(0.35,5.41)						
ASV_622	Firmicutes;D_2__Clostridia;D_3__Clostridiales;D_4__Lachnospiraceae;D_5__Blautia	0.14(0.00,4.29)	22.17(0.81,606.79)	3.06(0.20,47.19)						
ASV_623	Firmicutes;D_2__Clostridia;D_3__Clostridiales;D_4__Lachnospiraceae;D_5__[Ruminococcus] torques group;D_6__uncultured bacterium;D_7__;D_8__;D_9__;D_10__;D_11__;D_12__;D_13__;D_14__	0.69(0.11,4.48)	1.56(0.26,9.44)	1.07(0.24,4.76)						
ASV_627	Firmicutes;D_2__Clostridia;D_3__Clostridiales;D_4__Lachnospiraceae	7.09(0.28,181.41)	0.35(0.01,8.11)	2.47(0.22,28.27)						
ASV_628	Firmicutes;D_2__Clostridia;D_3__Clostridiales;D_4__Ruminococcaceae;D_5__Butyricicoccus	0.94(0.29,3.04)	2.25(0.72,7.00)	2.12(0.83,5.42)	0.94(0.29,3.02)	2.38(0.77,7.35)	2.23(0.87,5.70)			
ASV_630	Firmicutes;D_2__Clostridia;D_3__Clostridiales;D_4__Clostridiaceae 1;D_5__Clostridium sensu stricto 1	2.19(0.17,28.80)	3.99(0.33,48.83)	8.73(1.10,69.02);p=0.03999	4.63(0.39,55.36)	4.72(0.41,53.98)	21.86(2.92,163.85);p=0.00269			↑
ASV_631	Firmicutes;D_2__Clostridia;D_3__Clostridiales;D_4__Lachnospiraceae	0.58(0.01,22.69)	3.23(0.09,114.73)	1.86(0.09,37.56)						
ASV_632	Firmicutes;D_2__Clostridia;D_3__Clostridiales;D_4__Lachnospiraceae;D_5__Roseburia				4.62(0.06,358.62)	0.05(0.00,3.22)	0.24(0.01,4.54)			
ASV_634	Firmicutes;D_2__Clostridia;D_3__Clostridiales;D_4__Ruminococcaceae;D_5__Ruminiclostridium 5	0.58(0.04,8.18)	0.20(0.02,2.49)	0.11(0.01,0.93);p=0.04249						
ASV_635	Firmicutes;D_2__Clostridia;D_3__Clostridiales;D_4__Ruminococcaceae;D_5__DTU089;D_6__uncultured organism;D_7__;D_8__;D_9__;D_10__;D_11__;D_12__;D_13__;D_14__	0.14(0.00,12.72)	0.36(0.01,24.15)	0.05(0.00,1.85)						
ASV_636	Firmicutes;D_2__Clostridia;D_3__Clostridiales;D_4__Lachnospiraceae;D_5__Blautia;D_6__Ruminococcus sp. Marseille-P328;D_7__;D_8__;D_9__;D_10__;D_11__;D_12__;D_13__;D_14__	1.14(0.04,34.41)	4.36(0.12,153.58)	4.97(0.25,99.93)	0.57(0.03,12.27)	5.65(0.22,143.01)	3.24(0.15,69.33)			

ASV_637	Firmicutes;D_2__Clostridia;D_3__Clostridiales;D_4__Ruminococcaceae;D_5__Ruminococcaceae UCG-005	1.18(0.17,7.93)	3.35(0.50,22.30)	3.94(0.82,18.99)	1.01(0.16,6.51)	5.33(0.83,34.32)	5.36(1.09,26.40);p=0.03898			↑
ASV_638	Bacteroidetes;D_2__Bacteroidia;D_3__Bacteroidales;D_4__Prevotellaceae;D_5__Prevotella 9	14889453492.73(0.00,Inf)	0.00(0.00,Inf)	9.72(0.44,216.51)						
ASV_640	Firmicutes;D_2__Clostridia;D_3__Clostridiales;D_4__Ruminococcaceae;D_5__[Eubacterium] coprostanoligenes group	1191444902856.36(0.00,Inf)	0.00(0.00,Inf)	2.76(0.16,47.47)	5300612006238418.00(0.00,Inf)	0.00(0.00,Inf)	4.53(0.22,92.02)			
ASV_641	Actinobacteria;D_2__Coriobacteriia;D_3__Coriobacteriales;D_4__Coriobacteriaceae;D_5__Collinsella;D_6__uncultured bacterium;D_7__;D_8__;D_9__;D_10__;D_11__;D_12__;D_13__;D_14__	2051810336129.26(0.00,Inf)	0.00(0.00,Inf)	3.02(0.29,31.46)	18884783106366440.00(0.00,Inf)	0.00(0.00,Inf)	7.31(0.72,74.32)			
ASV_642	Firmicutes;D_2__Clostridia;D_3__Clostridiales;D_4__Lachnospiraceae	0.71(0.08,6.09)	1.21(0.15,9.54)	0.86(0.16,4.74)	0.57(0.07,4.96)	1.41(0.18,11.06)	0.80(0.14,4.45)			
ASV_643	Firmicutes;D_2__Clostridia;D_3__Clostridiales;D_4__Ruminococcaceae;D_5__Candidatus Soleaferrea;D_6__uncultured Anaerotruncus sp.;D_7__;D_8__;D_9__;D_10__;D_11__;D_12__;D_13__;D_14__	1.10(0.21,5.66)	1.18(0.24,5.72)	1.29(0.35,4.73)	1.10(0.21,5.68)	1.19(0.25,5.73)	1.31(0.36,4.78)			
ASV_644	Firmicutes;D_2__Clostridia;D_3__Clostridiales;D_4__Lachnospiraceae;D_5__Lachnospiraceae UCG-008;D_6__uncultured organism;D_7__;D_8__;D_9__;D_10__;D_11__;D_12__;D_13__;D_14__	2.57(0.31,21.44)	1.59(0.20,12.83)	4.08(0.76,21.97)	0.89(0.12,6.58)	2.58(0.37,18.11)	2.28(0.45,11.62)			
ASV_646	Firmicutes;D_2__Clostridia;D_3__Clostridiales;D_4__Ruminococcaceae	0.30(0.03,2.70)	1.37(0.18,10.20)	0.42(0.07,2.49)						
ASV_647	Firmicutes;D_2__Clostridia;D_3__Clostridiales;D_4__Ruminococcaceae;D_5__Acetanaerobacterium;D_6__uncultured rumen bacterium;D_7__;D_8__;D_9__;D_10__;D_11__;D_12__;D_13__;D_14__	0.54(0.13,2.19)	0.88(0.23,3.31)	0.47(0.15,1.45)	0.56(0.14,2.34)	0.85(0.23,3.19)	0.48(0.16,1.47)			
ASV_648	Firmicutes;D_2__Clostridia;D_3__Clostridiales;D_4__Lachnospiraceae;D_5__Lachnospiraceae UCG-004;D_6__uncultured organism;D_7__;D_8__;D_9__;D_10__;D_11__;D_12__;D_13__;D_14__	0.87(0.02,38.65)	2.11(0.05,83.93)	1.83(0.09,38.83)						
ASV_649	Firmicutes;D_2__Clostridia;D_3__Clostridiales;D_4__Clostridiaceae 1;D_5__Clostridium sensu stricto 1	11883930856.82(0.00,Inf)	0.00(0.00,Inf)	52.80(1.80,1547.14);p=0.02135						
ASV_650	Firmicutes;D_2__Clostridia;D_3__Clostridiales;D_4__Christensenellaceae;D_5__Catabacter	0.92(0.10,8.07)	1.01(0.13,8.09)	0.93(0.17,5.14)						
ASV_651	Actinobacteria;D_2__Actinobacteria;D_3__Micrococcales;D_4__Micrococcaceae;D_5__Rothia;D_6__uncultured organism;D_7__;D_8__;D_9__;D_10__;D_11__;D_12__;D_13__;D_14__	118015402553.65(0.00,Inf)	0.00(0.00,Inf)	0.53(0.04,6.99)						
ASV_652	Proteobacteria;D_2__Gammaproteobacteria;D_3__Betaproteobacteriales;D_4__Burkholderiaceae;D_5__Noviherbaspirillum	2.01(0.19,21.41)	1.19(0.11,12.37)	2.39(0.38,15.06)	1.40(0.13,14.73)	0.99(0.10,9.72)	1.39(0.23,8.20)			
ASV_653	Firmicutes;D_2__Clostridia;D_3__Clostridiales;D_4__Ruminococcaceae;D_5__GCA-900066225;D_6__Massilimaliae massiliensis;D_7__;D_8__;D_9__;D_10__;D_11__;D_12__;D_13__;D_14__	16629273736.61(0.00,Inf)	0.00(0.00,Inf)	1.36(0.03,64.84)						
ASV_654	Proteobacteria;D_2__Gammaproteobacteria;D_3__Betaproteobacteriales;D_4__Burkholderiaceae;D_5__Acidovorax	4656892048.79(0.00,Inf)	0.00(0.00,Inf)	4.08(0.60,27.78)	4460630453426185.00(0.00,Inf)	0.00(0.00,Inf)	4.53(0.65,31.70)			
ASV_656	Firmicutes;D_2__Clostridia;D_3__Clostridiales;D_4__Family XIII;D_5__Family XIII AD3011 group;D_6__uncultured bacterium;D_7__;D_8__;D_9__;D_10__;D_11__;D_12__;D_13__;D_14__	1.14(0.10,12.89)	2.37(0.23,24.93)	2.70(0.39,18.86)	2.25(0.24,20.84)	2.83(0.31,25.43)	6.36(1.00,40.48)			
ASV_658	Firmicutes;D_2__Clostridia;D_3__Clostridiales;D_4__Ruminococcaceae;D_5__Flavonifractor;D_6__uncultured bacterium;D_7__;D_8__;D_9__;D_10__;D_11__;D_12__;D_13__;D_14__	0.56(0.13,2.31)	1.05(0.27,4.08)	0.58(0.19,1.81)	0.71(0.17,2.95)	0.88(0.23,3.39)	0.63(0.20,1.93)			
ASV_659	Bacteroidetes;D_2__Bacteroidia;D_3__Bacteroidales;D_4__Tannerellaceae;D_5__Parabacteroides	0.07(0.00,1.48)	51797690149.56(0.00,Inf)	3459442852.27(0.00,Inf)						
ASV_668	Firmicutes;D_2__Bacilli;D_3__Lactobacillales;D_4__Lactobacillaceae;D_5__Lactobacillus	3.41(0.16,72.25)	7.09(0.36,141.18)	24.17(2.03,287.60);p=0.01171						
ASV_672	Firmicutes;D_2__Bacilli;D_3__Lactobacillales;D_4__Lactobacillaceae;D_5__Lactobacillus	0.13(0.01,1.39)	417.05(34.43,5051.51);p=0	52.89(6.25,447.32);p=0.00027						
ASV_676	Firmicutes;D_2__Bacilli;D_3__Lactobacillales;D_4__Lactobacillaceae;D_5__Lactobacillus	2.19(0.17,28.76)	0.71(0.06,8.52)	1.56(0.21,11.91)	0.67(0.05,8.48)	1.44(0.13,16.19)	0.96(0.13,7.19)			
ASV_678	Firmicutes;D_2__Erysipelotrichia;D_3__Erysipelotrichales;D_4__Erysipelotrichaceae;D_5__Erysipelatoclostridium	0.90(0.23,3.59)	1.23(0.33,4.63)	1.11(0.37,3.32)	0.89(0.22,3.58)	1.18(0.31,4.47)	1.05(0.35,3.17)			

ASV_679	Firmicutes;D_2__Clostridia;D_3__Clostridiales;D_4__Peptostreptococcaceae;D_5__Paeniclostridium;D_6__uncultured bacterium;D_7__;D_8__;D_9__;D_10__;D_11__;D_12__;D_13__;D_14__	1.86(0.04,87.41)	1.37(0.03,57.17)	2.56(0.12,53.81)						
ASV_683	Firmicutes;D_2__Clostridia;D_3__Clostridiales;D_4__Ruminococcaceae;D_5__Ruminiclostridium 5;D_6__uncultured organism;D_7__;D_8__;D_9__;D_10__;D_11__;D_12__;D_13__;D_14__	0.62(0.16,2.38)	1.54(0.42,5.61)	0.96(0.33,2.80)	0.44(0.12,1.69)	2.04(0.57,7.30)	0.91(0.31,2.63)			
ASV_684	Firmicutes;D_2__Clostridia;D_3__Clostridiales;D_4__Lachnospiraceae;D_5__[Eubacterium] xylanophilum group;D_6__metagenome;D_7__;D_8__;D_9__;D_10__;D_11__;D_12__;D_13__;D_14__				0.04(0.00,2.05)	48.09(1.06,2183.28);p=0.04665	1.81(0.07,47.17)		↑	
ASV_685	Firmicutes;D_2__Clostridia;D_3__Clostridiales;D_4__Lachnospiraceae;D_5__[Ruminococcus] torques group	1.33(0.11,16.59)	0.29(0.03,3.22)	0.38(0.05,2.72)	1.18(0.10,13.94)	0.31(0.03,3.25)	0.37(0.05,2.47)			
ASV_686	Firmicutes;D_2__Bacilli;D_3__Lactobacillales;D_4__Enterococcaceae;D_5__Enterococcus	0.65(0.04,10.40)	4.90(0.34,71.11)	3.17(0.35,28.91)	0.25(0.02,3.13)	4.69(0.41,53.07)	1.18(0.16,8.90)			
ASV_690	Firmicutes;D_2__Clostridia;D_3__Clostridiales;D_4__Lachnospiraceae;D_5__[Ruminococcus] torques group;D_6__uncultured Clostridiales bacterium;D_7__;D_8__;D_9__;D_10__;D_11__;D_12__;D_13__;D_14__				0.16(0.00,9.95)	3.76(0.08,186.30)	0.60(0.02,17.26)			
ASV_692	Actinobacteria;D_2__Actinobacteria;D_3__Bifidobacteriales;D_4__Bifidobacteriaceae;D_5__Alloscardovia;D_6__Bifidobacterium longum subsp. longum;D_7__;D_8__;D_9__;D_10__;D_11__;D_12__;D_13__;D_14__	17545545288.02(0.00,Inf)	0.00(0.00,Inf)	6.99(0.52,94.74)						
ASV_695	Actinobacteria;D_2__Actinobacteria;D_3__Micrococcales;D_4__Micrococcaceae;D_5__Rothia	3.16(0.18,54.93)	1.55(0.09,25.89)	4.92(0.51,47.10)	2.75(0.15,49.49)	2.64(0.14,49.86)	7.25(0.71,73.93)			
ASV_696	Actinobacteria;D_2__Actinobacteria;D_3__Actinomycetales;D_4__Actinomycetaceae;D_5__Actinomyces	4.07(0.53,31.07)	1.57(0.19,13.05)	6.38(1.29,31.49);p=0.02288	4.83(0.65,36.06)	2.85(0.32,24.97)	13.75(2.31,81.84);p=0.00397			↑
ASV_697	Firmicutes;D_2__Erysipelotrichia;D_3__Erysipelotrichales;D_4__Erysipelotrichaceae;D_5__Faecalitalea	0.20(0.02,1.81)	1.07(0.13,8.87)	0.21(0.04,1.24)	0.20(0.02,1.85)	1.01(0.13,8.15)	0.20(0.03,1.19)			
ASV_698	Firmicutes;D_2__Clostridia;D_3__Clostridiales;D_4__Lachnospiraceae;D_5__Sellimonas;D_6__Lachnoclostridium phocaeense;D_7__;D_8__;D_9__;D_10__;D_11__;D_12__;D_13__;D_14__	3.64(0.24,54.47)	0.28(0.02,3.85)	1.03(0.13,8.14)	3.22(0.22,48.16)	0.32(0.02,4.24)	1.03(0.14,7.79)			
ASV_699	Bacteroidetes;D_2__Bacteroidia;D_3__Bacteroidales;D_4__Prevotellaceae;D_5__Prevotella 6;D_6__uncultured bacterium;D_7__;D_8__;D_9__;D_10__;D_11__;D_12__;D_13__;D_14__	53.67(2.86,1008.18);p=0.00778	1.16(0.07,20.20)	62.40(6.21,627.01);p=0.00045	80.55(3.52,1840.98);p=0.00598	0.37(0.02,8.62)	29.78(3.21,275.86);p=0.00281	↑		↑
ASV_704	Firmicutes;D_2__Clostridia;D_3__Clostridiales;D_4__Ruminococcaceae;D_5__uncultured;D_6__uncultured bacterium;D_7__;D_8__;D_9__;D_10__;D_11__;D_12__;D_13__;D_14__	1.35(0.10,18.74)	0.15(0.01,1.85)	0.20(0.03,1.55)						
ASV_705	Firmicutes;D_2__Clostridia;D_3__Clostridiales;D_4__Family XI;D_5__Ezakiella;D_6__Fenollaria timonensis;D_7__;D_8__;D_9__;D_10__;D_11__;D_12__;D_13__;D_14__	9.94(0.59,168.76)	0.06(0.00,0.88);p=0.03983	0.57(0.07,4.63)	7.35(0.47,115.18)	0.08(0.01,1.17)	0.61(0.09,4.19)			
ASV_708	Firmicutes;D_2__Clostridia;D_3__Clostridiales;D_4__Ruminococcaceae;D_5__Hydrogenoanaerobacterium;D_6__uncultured bacterium;D_7__;D_8__;D_9__;D_10__;D_11__;D_12__;D_13__;D_14__	1.07(0.06,18.39)	1.89(0.12,30.15)	2.02(0.21,19.87)						
ASV_709	Firmicutes;D_2__Clostridia;D_3__Clostridiales;D_4__Ruminococcaceae;D_5__Oscillibacter;D_6__uncultured bacterium;D_7__;D_8__;D_9__;D_10__;D_11__;D_12__;D_13__;D_14__	2843252920.36(0.00,Inf)	0.00(0.00,Inf)	2.09(0.13,32.80)						
ASV_710	Firmicutes;D_2__Clostridia;D_3__Clostridiales;D_4__Lachnospiraceae;D_5__Lachnospiraceae UCG-008;D_6__metagenome;D_7__;D_8__;D_9__;D_10__;D_11__;D_12__;D_13__;D_14__				1.11(0.08,15.37)	0.26(0.02,3.07)	0.28(0.04,2.12)			
ASV_711	Firmicutes;D_2__Clostridia;D_3__Clostridiales;D_4__Lachnospiraceae;D_5__uncultured organism;D_6__;D_7__;D_8__;D_9__;D_10__;D_11__;D_12__;D_13__;D_14__	0.39(0.02,7.70)	0.69(0.04,11.75)	0.27(0.02,2.87)	0.50(0.02,10.26)	0.48(0.03,8.18)	0.24(0.02,2.59)			
ASV_713	Firmicutes;D_2__Clostridia;D_3__Clostridiales;D_4__Lachnospiraceae;D_5__Tyzzerella;D_6__uncultured bacterium;D_7__;D_8__;D_9__;D_10__;D_11__;D_12__;D_13__;D_14__	2752443900.26(0.00,Inf)	0.00(0.00,Inf)	0.77(0.11,5.22)	32191459879085.90(0.00,Inf)	0.00(0.00,Inf)	0.60(0.09,3.82)			
ASV_714	Firmicutes;D_2__Clostridia;D_3__Clostridiales;D_4__Lachnospiraceae;D_5__Lachnoclostridium	12.98(0.85,199.07)	0.08(0.01,1.18)	1.05(0.22,5.05)	10.22(0.85,123.44)	0.14(0.01,1.56)	1.41(0.32,6.28)			
ASV_716	Proteobacteria;D_2__Gammaproteobacteria;D_3__Betaproteobacteriales;D_4__Burkholderiaceae	1659445338.53(0.00,Inf)	0.00(0.00,Inf)	1.93(0.16,23.85)						
ASV_718	Actinobacteria;D_2__Actinobacteria;D_3__Corynebacteriales;D_4__Corynebacteriaceae;D_5__Corynebacterium 1	15056395178.50(0.00,Inf)	0.00(0.00,Inf)	1.23(0.03,55.88)						
ASV_719	Proteobacteria;D_2__Gammaproteobacteria;D_3__Betaproteobacteriales;D_4__Burkholderiaceae	0.56(0.08,3.95)	2.41(0.36,16.02)	1.35(0.26,7.14)	0.29(0.04,2.13)	4.17(0.60,29.14)	1.22(0.22,6.77)			

ASV_721	Actinobacteria;D_2__Actinobacteria;D_3__Actinomycetales;D_4__Actinomycetaceae;D_5__Actinomyces	2.92(0.63,13.63)	0.68(0.15,3.04)	1.99(0.61,6.49)	3.72(0.79,17.52)	0.58(0.13,2.61)	2.16(0.68,6.94)			
ASV_722	Firmicutes;D_2__Erysipelotrichia;D_3__Erysipelotrichales;D_4__Erysipelotrichaceae;D_5__Erysipelatoclostridium;D_6__Massiliomicrobiota timonensis;D_7__;D_8__;D_9__;D_10__;D_11__;D_12__;D_13__;D_14__	0.18(0.01,4.41)	10.67(0.49,234.16)	1.92(0.14,25.65)						
ASV_723	Firmicutes;D_2__Clostridia;D_3__Clostridiales;D_4__Ruminococcaceae;D_5__Ruminiclostridium;D_6__Massilioclostridium coli;D_7__;D_8__;D_9__;D_10__;D_11__;D_12__;D_13__;D_14__	16030112554.41(0.00,Inf)	0.00(0.00,Inf)	2.89(0.50,16.59)	5572803550627860.00(0.00,Inf)	0.00(0.00,Inf)	3.24(0.56,18.67)			
ASV_725	Firmicutes;D_2__Clostridia;D_3__Clostridiales;D_4__Lachnospiraceae;D_5__Fusicatenibacter;D_6__uncultured bacterium;D_7__;D_8__;D_9__;D_10__;D_11__;D_12__;D_13__;D_14__	0.22(0.01,5.73)	895416592747005.00(0.00,Inf)	198310532369821.00(0.00,Inf)	0.43(0.02,10.17)	53359483211339.20(0.00,Inf)	22726948145776.70(0.00,Inf)			
ASV_726	Firmicutes;D_2__Negativicutes;D_3__Selenomonadales;D_4__Veillonellaceae;D_5__Veillonella	0.15(0.00,8.05)	0.49(0.01,20.18)	0.08(0.00,1.80)						
ASV_729	Firmicutes;D_2__Clostridia;D_3__Clostridiales;D_4__Lachnospiraceae;D_5__[Ruminococcus] gnavus group	2.11(0.04,119.71)	0.08(0.00,3.92)	0.17(0.01,4.10)						
ASV_733	Firmicutes;D_2__Clostridia;D_3__Clostridiales;D_4__Clostridiaceae 1;D_5__Clostridium sensu stricto 1	5.03(0.20,125.85)	1.48(0.07,32.86)	7.44(0.58,95.82)	0.92(0.05,18.18)	4.01(0.22,72.25)	3.68(0.34,40.07)			
ASV_734	Firmicutes;D_2__Clostridia;D_3__Clostridiales;D_4__Lachnospiraceae;D_5__Eisenbergiella;D_6__uncultured organism;D_7__;D_8__;D_9__;D_10__;D_11__;D_12__;D_13__;D_14__	9.27(1.76,48.85);p=0.00864	0.08(0.02,0.38);p=0.00162	0.71(0.20,2.56)						
ASV_738	Firmicutes;D_2__Clostridia;D_3__Clostridiales;D_4__Ruminococcaceae;D_5__Ruminiclostridium 9;D_6__uncultured bacterium;D_7__;D_8__;D_9__;D_10__;D_11__;D_12__;D_13__;D_14__	14.74(0.76,286.78)	0.12(0.01,2.07)	1.74(0.18,16.66)						
ASV_740	Firmicutes;D_2__Clostridia;D_3__Clostridiales;D_4__Lachnospiraceae;D_5__Lachnospiraceae UCG-004;D_6__uncultured organism;D_7__;D_8__;D_9__;D_10__;D_11__;D_12__;D_13__;D_14__	0.34(0.03,3.80)	2.86(0.28,29.26)	0.96(0.14,6.61)	0.18(0.02,1.98)	5.16(0.52,51.16)	0.92(0.14,6.23)			
ASV_741	Firmicutes;D_2__Clostridia;D_3__Clostridiales;D_4__Lachnospiraceae;D_5__Lachnoclostridium;D_6__uncultured organism;D_7__;D_8__;D_9__;D_10__;D_11__;D_12__;D_13__;D_14__	0.13(0.00,5.05)	1.55(0.05,49.54)	0.19(0.01,3.81)						
ASV_744	Firmicutes;D_2__Clostridia;D_3__Clostridiales;D_4__Lachnospiraceae;D_5__Lachnoclostridium				0.13(0.00,4.55)	26.70(0.85,839.06)	3.52(0.20,61.92)			
ASV_746	Firmicutes;D_2__Clostridia;D_3__Clostridiales;D_4__Ruminococcaceae;D_5__Oscillibacter;D_6__uncultured organism;D_7__;D_8__;D_9__;D_10__;D_11__;D_12__;D_13__;D_14__	0.09(0.00,2.25)	0.38(0.02,6.26)	0.03(0.00,0.50);p=0.0137	0.02(0.00,0.49);p=0.01765	1.76(0.13,24.31)	0.03(0.00,0.54);p=0.01718	↓		↓
ASV_747	Firmicutes;D_2__Clostridia;D_3__Clostridiales;D_4__Lachnospiraceae;D_5__Sellimonas;D_6__uncultured bacterium;D_7__;D_8__;D_9__;D_10__;D_11__;D_12__;D_13__;D_14__	4.07(0.58,28.48)	0.60(0.09,3.88)	2.43(0.52,11.30)	3.34(0.49,22.80)	0.53(0.08,3.31)	1.77(0.39,8.01)			
ASV_751	Firmicutes;D_2__Clostridia;D_3__Clostridiales;D_4__Lachnospiraceae	0.07(0.00,4.28)	20.87(0.37,1162.49)	1.37(0.04,42.06)						
ASV_757	Firmicutes;D_2__Erysipelotrichia;D_3__Erysipelotrichales;D_4__Erysipelotrichaceae;D_5__Faecalitalea;D_6__[Eubacterium] dolichum;D_7__;D_8__;D_9__;D_10__;D_11__;D_12__;D_13__;D_14__	0.58(0.02,18.30)	9.85(0.32,304.39)	5.73(0.32,103.51)	0.33(0.01,10.81)	20.13(0.58,702.53)	6.58(0.33,130.69)			
ASV_760	Firmicutes;D_2__Clostridia;D_3__Clostridiales;D_4__Family XIII;D_5__[Eubacterium] nodatum group	0.20(0.01,3.51)	11.86(0.68,205.95)	2.38(0.19,30.55)	0.06(0.00,1.28)	12.88(0.81,205.88)	0.84(0.06,11.68)			
ASV_761	Firmicutes;D_2__Erysipelotrichia;D_3__Erysipelotrichales;D_4__Erysipelotrichaceae;D_5__Merdibacter;D_6__uncultured bacterium;D_7__;D_8__;D_9__;D_10__;D_11__;D_12__;D_13__;D_14__	0.00(0.00,Inf)	3.74(0.16,85.40)	0.00(0.00,Inf)	0.00(0.00,Inf)	7.10(0.24,212.63)	0.00(0.00,Inf)			
ASV_765	Firmicutes;D_2__Clostridia;D_3__Clostridiales;D_4__Lachnospiraceae;D_5__[Ruminococcus] torques group;D_6__uncultured bacterium;D_7__;D_8__;D_9__;D_10__;D_11__;D_12__;D_13__;D_14__	0.16(0.00,6.55)	0.78(0.04,15.77)	0.12(0.01,2.84)						
ASV_767	Firmicutes;D_2__Erysipelotrichia;D_3__Erysipelotrichales;D_4__Erysipelotrichaceae;D_5__[Clostridium] innocuum group	2.12(0.15,29.75)	0.14(0.01,1.72)	0.29(0.04,2.20)						
ASV_773	Firmicutes;D_2__Clostridia;D_3__Clostridiales;D_4__Ruminococcaceae;D_5__Ruminiclostridium	1.51(0.05,47.66)	1.07(0.04,31.19)	1.62(0.11,23.75)	0.45(0.01,18.17)	0.92(0.03,29.16)	0.41(0.03,6.84)			
ASV_774	Firmicutes;D_2__Clostridia;D_3__Clostridiales;D_4__Ruminococcaceae;D_5__Faecalibacterium	1654965025.51(0.00,Inf)	0.00(0.00,Inf)	0.62(0.03,11.71)						
ASV_775	Actinobacteria;D_2__Actinobacteria;D_3__Propionibacteriales;D_4__Propionibacteriaceae;D_5__Cutibacterium				0.41(0.00,Inf)	0.00(0.00,Inf)	0.00(0.00,Inf)			
ASV_776	Firmicutes;D_2__Clostridia;D_3__Clostridiales;D_4__Lachnospiraceae;D_5__Eisenbergiella	200804247981.67(0.00,Inf)	0.00(0.00,Inf)	2.62(0.15,47.26)						

ASV_777	Firmicutes;D_2__Clostridia;D_3__Clostridiales;D_4__Lachnospiraceae;D_5__Lachnoclostridium	0.11(0.00,4.12)	3.58(0.16,82.51)	0.39(0.02,9.53)						
ASV_780	Actinobacteria;D_2__Coriobacteriia;D_3__Coriobacteriales;D_4__Eggerthellaceae;D_5__Gordonibacter;D_6__unidentified;D_7__;D_8__;D_9__;D_10__;D_11__;D_12__;D_13__;D_14__	2.18(0.09,50.76)	0.41(0.02,8.44)	0.89(0.08,10.30)	2.98(0.14,65.16)	0.57(0.03,11.39)	1.69(0.16,17.31)			
ASV_781	Firmicutes;D_2__Clostridia;D_3__Clostridiales;D_4__Ruminococcaceae;D_5__Ruminococcaceae UCG-008;D_6__uncultured bacterium;D_7__;D_8__;D_9__;D_10__;D_11__;D_12__;D_13__;D_14__	0.61(0.02,18.94)	1.63(0.06,42.92)	0.99(0.06,16.60)	0.66(0.01,29.45)	0.68(0.02,19.18)	0.44(0.02,9.12)			
ASV_790	Firmicutes;D_2__Clostridia;D_3__Clostridiales;D_4__Lachnospiraceae;D_5__Lachnospira	416087655138.13(0.00,Inf)	0.00(0.00,Inf)	9.13(0.13,651.08)						
ASV_792	Firmicutes;D_2__Clostridia;D_3__Clostridiales;D_4__Lachnospiraceae;D_5__Lachnospiraceae UCG-001;D_6__Lachnospiraceae bacterium TF01-11;D_7__;D_8__;D_9__;D_10__;D_11__;D_12__;D_13__;D_14__	46.06(1.39,1525.70);p=0.03199	0.01(0.00,0.30);p=0.00776	0.46(0.04,5.27)						
ASV_793	Firmicutes;D_2__Clostridia;D_3__Clostridiales;D_4__Lachnospiraceae;D_5__Lachnospiraceae UCG-009	3008919634.20(0.00,Inf)	0.00(0.00,Inf)	0.31(0.03,3.72)						
ASV_794	Firmicutes;D_2__Clostridia;D_3__Clostridiales;D_4__Lachnospiraceae;D_5__[Ruminococcus] torques group	1.20(0.09,16.27)	1.46(0.11,19.56)	1.76(0.22,14.11)	1.70(0.12,23.29)	1.82(0.13,26.61)	3.11(0.36,27.15)			
ASV_795	Firmicutes;D_2__Clostridia;D_3__Clostridiales;D_4__Lachnospiraceae;D_5__[Ruminococcus] gauvreauii group;D_6__uncultured bacterium;D_7__;D_8__;D_9__;D_10__;D_11__;D_12__;D_13__;D_14__	0.51(0.03,7.61)	1.88(0.14,25.06)	0.96(0.11,8.27)	0.21(0.01,3.12)	3.35(0.26,42.70)	0.71(0.08,6.18)			
ASV_798	Actinobacteria;D_2__Coriobacteriia;D_3__Coriobacteriales;D_4__Eggerthellaceae;D_5__Eggerthella;D_6__uncultured bacterium;D_7__;D_8__;D_9__;D_10__;D_11__;D_12__;D_13__;D_14__	1.30(0.03,54.77)	0.11(0.00,4.04)	0.15(0.01,2.78)						
ASV_810	Firmicutes;D_2__Clostridia;D_3__Clostridiales;D_4__Lachnospiraceae;D_5__Marvinbryantia	1.66(0.13,21.21)	0.60(0.05,6.93)	0.99(0.13,7.36)	0.85(0.07,10.67)	0.50(0.05,5.50)	0.42(0.06,3.08)			
ASV_812	Firmicutes;D_2__Erysipelotrichia;D_3__Erysipelotrichales;D_4__Erysipelotrichaceae;D_5__[Clostridium] innocuum group	0.63(0.07,5.97)	7.06(0.79,63.18)	4.44(0.72,27.55)	0.46(0.05,4.19)	11.00(1.29,93.95);p=0.02847	5.04(0.82,31.02)		↑	
ASV_813	Firmicutes;D_2__Clostridia;D_3__Clostridiales;D_4__Ruminococcaceae;D_5__uncultured	0.35(0.02,8.14)	3.30(0.16,67.31)	1.16(0.09,15.12)	0.18(0.01,3.85)	8.93(0.48,166.99)	1.64(0.11,25.19)			
ASV_814	Firmicutes;D_2__Clostridia;D_3__Clostridiales;D_4__Peptococcaceae;D_5__uncultured	2.29(0.21,24.77)	0.43(0.04,4.23)	0.98(0.15,6.29)	1.81(0.17,18.92)	0.42(0.05,3.98)	0.77(0.12,4.73)			
ASV_817	Firmicutes;D_2__Clostridia;D_3__Clostridiales;D_4__Ruminococcaceae	0.49(0.06,4.05)	1.25(0.17,9.18)	0.61(0.11,3.31)	0.43(0.05,3.61)	1.32(0.18,9.55)	0.57(0.10,3.13)			
ASV_819	Firmicutes;D_2__Clostridia;D_3__Clostridiales;D_4__Ruminococcaceae	0.04(0.00,0.81);p=0.03561	0.79(0.09,7.05)	0.03(0.00,0.44);p=0.00973	0.04(0.00,0.73);p=0.03003	0.85(0.10,7.35)	0.03(0.00,0.43);p=0.00974	↓		↓
ASV_821	Firmicutes;D_2__Clostridia;D_3__Clostridiales;D_4__Lachnospiraceae;D_5__Shuttleworthia;D_6__uncultured bacterium;D_7__;D_8__;D_9__;D_10__;D_11__;D_12__;D_13__;D_14__	1.64(0.16,16.44)	1.86(0.20,17.34)	3.05(0.49,19.11)	0.83(0.09,7.39)	3.44(0.41,28.98)	2.86(0.49,16.76)			
ASV_822	Firmicutes;D_2__Clostridia;D_3__Clostridiales;D_4__Family XIII;D_5__Family XIII AD3011 group	0.21(0.01,5.69)	9.37(0.35,247.84)	2.02(0.10,41.19)						
ASV_825	Firmicutes;D_2__Clostridia;D_3__Clostridiales;D_4__Ruminococcaceae;D_5__Candidatus Soleaferrea;D_6__uncultured bacterium;D_7__;D_8__;D_9__;D_10__;D_11__;D_12__;D_13__;D_14__	0.12(0.01,1.71)	2.70(0.25,28.82)	0.32(0.03,3.09)						
ASV_826	Firmicutes;D_2__Clostridia;D_3__Clostridiales;D_4__Christensenellaceae;D_5__Christensenellaceae R-7 group				1.02(0.16,6.60)	0.69(0.12,4.02)	0.70(0.16,3.01)			
ASV_828	Firmicutes;D_2__Clostridia;D_3__Clostridiales;D_4__Family XIII;D_5__Family XIII AD3011 group	2.78(0.08,95.31)	0.26(0.01,7.73)	0.72(0.05,10.79)	2.52(0.07,92.68)	0.16(0.01,4.99)	0.41(0.03,5.74)			
ASV_829	Firmicutes;D_2__Clostridia;D_3__Clostridiales;D_4__Christensenellaceae;D_5__Christensenellaceae R-7 group;D_6__uncultured Clostridium sp.;D_7__;D_8__;D_9__;D_10__;D_11__;D_12__;D_13__;D_14__	14.60(1.30,164.51);p=0.03003	0.22(0.02,2.31)	3.17(0.57,17.58)						
ASV_830	Firmicutes;D_2__Clostridia;D_3__Clostridiales;D_4__Eubacteriaceae;D_5__Anaerofustis;D_6__unidentified;D_7__;D_8__;D_9__;D_10__;D_11__;D_12__;D_13__;D_14__				1.80(0.06,53.89)	1.17(0.04,31.21)	2.11(0.14,31.32)			
ASV_831	Firmicutes;D_2__Clostridia;D_3__Clostridiales;D_4__Lachnospiraceae;D_5__GCA-900066755;D_6__uncultured bacterium;D_7__;D_8__;D_9__;D_10__;D_11__;D_12__;D_13__;D_14__	2.69(0.28,25.55)	0.35(0.04,3.11)	0.95(0.18,5.12)						
ASV_832	Firmicutes;D_2__Clostridia;D_3__Clostridiales	0.65(0.02,22.06)	0.45(0.02,11.92)	0.29(0.02,4.53)	0.68(0.02,22.48)	0.53(0.02,14.23)	0.36(0.03,5.12)			
ASV_834	Firmicutes;D_2__Clostridia;D_3__Clostridiales;D_4__Lachnospiraceae;D_5__[Eubacterium] fissicatena group	0.41(0.01,25.34)	0.94(0.02,43.56)	0.38(0.01,10.45)						

ASV_838	Firmicutes;D_2__Clostridia;D_3__Clostridiales;D_4__Christensenellaceae;D_5__uncultured;D_6__uncultured bacterium;D_7__;D_8__;D_9__;D_10__;D_11__;D_12__;D_13__;D_14__	0.58(0.01,28.36)	2.88(0.06,132.26)	1.66(0.06,45.85)						
ASV_839	Firmicutes;D_2__Clostridia;D_3__Clostridiales;D_4__Ruminococcaceae;D_5__Ruminococcaceae UCG-005	0.34(0.02,6.53)	50.90(2.10,1232.72);p=0.01567	17.51(1.08,284.07);p=0.04404						
ASV_840	Firmicutes;D_2__Clostridia;D_3__Clostridiales;D_4__Lachnospiraceae;D_5__Lachnospiraceae UCG-010;D_6__uncultured organism;D_7__;D_8__;D_9__;D_10__;D_11__;D_12__;D_13__;D_14__	0.92(0.01,70.08)	0.16(0.00,9.08)	0.14(0.01,3.94)						
ASV_845	Firmicutes;D_2__Clostridia;D_3__Clostridiales;D_4__Ruminococcaceae;D_5__Harryflintia	1.39(0.10,19.83)	0.92(0.07,11.98)	1.27(0.16,10.04)	0.48(0.03,7.75)	1.10(0.08,15.45)	0.52(0.06,4.41)			
ASV_846	Firmicutes;D_2__Clostridia;D_3__Clostridiales;D_4__Ruminococcaceae;D_5__Candidatus Soleaferrea;D_6__uncultured bacterium;D_7__;D_8__;D_9__;D_10__;D_11__;D_12__;D_13__;D_14__	1.31(0.02,91.95)	0.77(0.01,46.22)	1.01(0.04,27.52)						
ASV_849	Firmicutes;D_2__Clostridia;D_3__Clostridiales;D_4__Clostridiales vadinBB60 group;D_5__uncultured organism;D_6__;D_7__;D_8__;D_9__;D_10__;D_11__;D_12__;D_13__;D_14__	1.23(0.20,7.63)	0.24(0.04,1.36)	0.29(0.07,1.20)						
ASV_850	Firmicutes;D_2__Clostridia;D_3__Clostridiales;D_4__Christensenellaceae;D_5__Christensenella;D_6__Christensenella minuta;D_7__;D_8__;D_9__;D_10__;D_11__;D_12__;D_13__;D_14__	0.17(0.02,1.81)	0.65(0.08,5.16)	0.11(0.02,0.77);p=0.02567	0.09(0.01,1.02)	1.10(0.15,8.31)	0.10(0.01,0.75);p=0.02507			↓
ASV_852	Firmicutes;D_2__Clostridia;D_3__Clostridiales;D_4__Lachnospiraceae;D_5__Blautia	0.00(0.00,Inf)	2.17(0.04,110.51)	0.00(0.00,Inf)						
ASV_856	Firmicutes;D_2__Clostridia;D_3__Clostridiales;D_4__Ruminococcaceae;D_5__Oscillibacter;D_6__uncultured organism;D_7__;D_8__;D_9__;D_10__;D_11__;D_12__;D_13__;D_14__	0.00(0.00,Inf)	0.59(0.02,17.12)	0.00(0.00,Inf)	0.00(0.00,Inf)	0.65(0.02,21.98)	0.00(0.00,Inf)			
ASV_887	Firmicutes;D_2__Clostridia;D_3__Clostridiales;D_4__Clostridiales vadinBB60 group;D_5__uncultured bacterium;D_6__;D_7__;D_8__;D_9__;D_10__;D_11__;D_12__;D_13__;D_14__	0.75(0.02,24.11)	3.66(0.13,103.86)	2.73(0.17,43.42)						
ASV_921	Firmicutes;D_2__Clostridia;D_3__Clostridiales;D_4__Lachnospiraceae;D_5__Dorea	0.11(0.01,1.11)	4.59(0.50,42.24)	0.50(0.08,3.21)	0.10(0.01,1.01)	5.37(0.59,49.27)	0.53(0.08,3.38)			
ASV_922	Firmicutes;D_2__Clostridia;D_3__Clostridiales;D_4__Ruminococcaceae;D_5__Angelakisella;D_6__uncultured bacterium;D_7__;D_8__;D_9__;D_10__;D_11__;D_12__;D_13__;D_14__				1.69(0.15,18.65)	0.16(0.02,1.60)	0.28(0.04,1.80)			
ASV_925	Firmicutes;D_2__Clostridia;D_3__Clostridiales;D_4__Ruminococcaceae;D_5__Ruminococcaceae UCG-009	0.10(0.01,0.99);p=0.0486	5.39(0.60,48.89)	0.53(0.08,3.35)	0.09(0.01,0.87);p=0.03767	5.05(0.56,45.15)	0.44(0.07,2.75)	↓		
ASV_932	Actinobacteria;D_2__Actinobacteria;D_3__Actinomycetales;D_4__Actinomycetaceae;D_5__Actinomyces	1.54(0.02,126.93)	0.12(0.00,8.25)	0.19(0.01,5.75)						
ASV_937	Firmicutes;D_2__Clostridia;D_3__Clostridiales;D_4__Family XI;D_5__Parvimonas	10709169098.99(0.00,Inf)	0.00(0.00,Inf)	2.22(0.47,10.59)	7146621479752325.00(0.00,Inf)	0.00(0.00,Inf)	1.51(0.32,7.14)			
ASV_943	Firmicutes;D_2__Clostridia;D_3__Clostridiales;D_4__Ruminococcaceae	3000283454496.91(0.00,Inf)	0.00(0.00,Inf)	3.86(0.13,110.47)	2406588950391878.00(0.00,Inf)	0.00(0.00,Inf)	4.13(0.14,119.19)			
ASV_949	Firmicutes;D_2__Clostridia;D_3__Clostridiales;D_4__Family XI;D_5__Peptoniphilus;D_6__uncultured organism;D_7__;D_8__;D_9__;D_10__;D_11__;D_12__;D_13__;D_14__	0.24(0.00,20.95)	0.69(0.01,45.41)	0.17(0.00,5.87)						
ASV_953	Firmicutes;D_2__Clostridia;D_3__Clostridiales;D_4__Ruminococcaceae;D_5__Subdoligranulum	1.04(0.11,9.75)	0.11(0.01,0.94);p=0.04374	0.11(0.02,0.67);p=0.0164						
ASV_954	Firmicutes;D_2__Clostridia;D_3__Clostridiales;D_4__Family XI;D_5__Peptoniphilus;D_6__Peptoniphilus lacrimalis;D_7__;D_8__;D_9__;D_10__;D_11__;D_12__;D_13__;D_14__	3.64(0.10,126.60)	0.27(0.01,8.40)	1.00(0.07,13.79)						
ASV_955	Bacteroidetes;D_2__Bacteroidia;D_3__Bacteroidales;D_4__Porphyromonadaceae;D_5__Porphyromonas	18.93(0.98,364.06)	0.04(0.00,0.70);p=0.02782	0.74(0.13,4.30)						
ASV_956	Firmicutes;D_2__Clostridia;D_3__Clostridiales;D_4__Family XI;D_5__Finegoldia;D_6__uncultured bacterium;D_7__;D_8__;D_9__;D_10__;D_11__;D_12__;D_13__;D_14__	0.61(0.02,14.95)	9.31(0.40,216.49)	5.64(0.41,78.44)	1.01(0.05,22.19)	9.07(0.40,206.47)	9.13(0.67,124.47)			
ASV_959	Firmicutes;D_2__Clostridia;D_3__Clostridiales;D_4__Eubacteriaceae;D_5__Anaerofustis;D_6__Anaerofustis sp. Marseille-P2441;D_7__;D_8__;D_9__;D_10__;D_11__;D_12__;D_13__;D_14__	0.31(0.02,6.03)	26.16(1.11,614.85);p=0.04271	8.15(0.51,131.20)						
ASV_964	Firmicutes;D_2__Clostridia;D_3__Clostridiales;D_4__Clostridiales vadinBB60 group;D_5__gut metagenome;D_6__;D_7__;D_8__;D_9__;D_10__;D_11__;D_12__;D_13__;D_14__	1.03(0.09,11.53)	0.67(0.07,6.82)	0.69(0.10,4.64)	1.85(0.17,20.32)	0.94(0.10,9.32)	1.74(0.26,11.53)			

ASV_965	Firmicutes;D_2__Negativicutes;D_3__Selenomonadales;D_4__Veillonellaceae;D_5__Dialister;D_6__uncultured bacterium;D_7__;D_8__;D_9__;D_10__;D_11__;D_12__;D_13__;D_14__	12503641998.82(0.00,Inf)	0.00(0.00,Inf)	2.20(0.18,26.43)	98137197429800.50(0.00,Inf)	0.00(0.00,Inf)	2.49(0.21,29.55)			
ASV_966	Firmicutes;D_2__Clostridia;D_3__Clostridiales;D_4__Ruminococcaceae;D_5__Ruminococcaceae UCG-005;D_6__uncultured rumen bacterium;D_7__;D_8__;D_9__;D_10__;D_11__;D_12__;D_13__;D_14__	1.43(0.10,19.40)	0.54(0.04,6.62)	0.77(0.10,5.92)						
ASV_968	Firmicutes;D_2__Clostridia;D_3__Clostridiales;D_4__Lachnospiraceae;D_5__Lachnospiraceae UCG-010;D_6__uncultured bacterium;D_7__;D_8__;D_9__;D_10__;D_11__;D_12__;D_13__;D_14__				0.41(0.01,14.99)	0.38(0.01,10.81)	0.16(0.01,2.61)			
ASV_969	Firmicutes;D_2__Clostridia;D_3__Clostridiales;D_4__Ruminococcaceae;D_5__Ruminococcaceae UCG-010	0.24(0.01,8.27)	0.12(0.00,3.54)	0.03(0.00,0.49);p=0.01424	0.24(0.01,8.38)	0.21(0.01,5.93)	0.05(0.00,0.85);p=0.03804			↓
ASV_973	Bacteroidetes;D_2__Bacteroidia;D_3__Bacteroidales;D_4__Porphyromonadaceae;D_5__Porphyromonas;D_6__unidentified;D_7__;D_8__;D_9__;D_10__;D_11__;D_12__;D_13__;D_14__				0.25(0.02,3.18)	1.60(0.16,16.47)	0.40(0.05,3.06)			
ASV_974	Firmicutes;D_2__Clostridia;D_3__Clostridiales;D_4__Ruminococcaceae;D_5__Ruminiclostridium 5	0.92(0.08,10.22)	3.92(0.34,45.66)	3.60(0.45,28.64)	0.63(0.06,6.58)	8.26(0.65,104.55)	5.19(0.61,44.30)			
ASV_976	Firmicutes;D_2__Clostridia;D_3__Clostridiales;D_4__Christensenellaceae;D_5__Christensenellaceae R-7 group;D_6__uncultured Christensenellaceae bacterium;D_7__;D_8__;D_9__;D_10__;D_11__;D_12__;D_13__;D_14__	1.72(0.18,16.72)	0.26(0.03,2.29)	0.45(0.08,2.41)	1.79(0.18,17.40)	0.28(0.03,2.44)	0.51(0.10,2.72)			
ASV_978	Bacteroidetes;D_2__Bacteroidia;D_3__Bacteroidales;D_4__Prevotellaceae;D_5__Prevotella;D_6__Prevotella bivia;D_7__;D_8__;D_9__;D_10__;D_11__;D_12__;D_13__;D_14__	7.22(0.10,543.23)	1.49(0.02,103.21)	10.75(0.35,325.61)						
ASV_980	Bacteroidetes;D_2__Bacteroidia;D_3__Bacteroidales;D_4__Porphyromonadaceae;D_5__Porphyromonas	12799350670.25(0.00,Inf)	0.00(0.00,Inf)	2.14(0.32,14.03)	4247259934398016.00(0.00,Inf)	0.00(0.00,Inf)	3.03(0.46,19.88)			
ASV_981	Firmicutes;D_2__Clostridia;D_3__Clostridiales;D_4__Family XI;D_5__Anaerococcus	4.23(0.16,112.17)	1.12(0.04,28.28)	4.72(0.37,60.22)	5.13(0.19,138.16)	1.36(0.05,37.40)	7.00(0.56,87.69)			
ASV_984	Firmicutes;D_2__Clostridia;D_3__Clostridiales;D_4__Lachnospiraceae;D_5__Marvinbryantia;D_6__uncultured bacterium;D_7__;D_8__;D_9__;D_10__;D_11__;D_12__;D_13__;D_14__	4.12(0.31,54.26)	0.04(0.00,0.48);p=0.01082	0.16(0.02,1.10)						
ASV_985	Firmicutes;D_2__Clostridia;D_3__Clostridiales;D_4__Lachnospiraceae;D_5__Lachnoclostridium	4.92(0.19,125.71)	5.27(0.17,166.01)	25.95(1.51,445.67);p=0.0248						
ASV_986	Firmicutes;D_2__Clostridia;D_3__Clostridiales;D_4__Ruminococcaceae;D_5__Ruminiclostridium 5;D_6__uncultured bacterium;D_7__;D_8__;D_9__;D_10__;D_11__;D_12__;D_13__;D_14__	1.31(0.19,9.10)	1.02(0.16,6.65)	1.33(0.29,6.02)	1.99(0.26,15.12)	0.55(0.08,3.86)	1.08(0.25,4.65)			
ASV_1003	Firmicutes;D_2__Clostridia;D_3__Clostridiales;D_4__Ruminococcaceae;D_5__Fournierella;D_6__uncultured organism;D_7__;D_8__;D_9__;D_10__;D_11__;D_12__;D_13__;D_14__	2.21(0.07,67.66)	0.12(0.00,3.11)	0.26(0.02,3.65)						
ASV_1004	Firmicutes;D_2__Clostridia;D_3__Clostridiales;D_4__Ruminococcaceae	2.15(0.08,60.11)	4.25(0.16,111.72)	9.14(0.62,135.86)	0.12(0.00,3.12)	5.49(0.24,125.86)	0.65(0.05,9.13)			
ASV_1005	Firmicutes;D_2__Clostridia;D_3__Clostridiales;D_4__Ruminococcaceae	0.47(0.04,5.14)	0.70(0.07,6.96)	0.33(0.05,2.20)						
ASV_1009	Firmicutes;D_2__Clostridia;D_3__Clostridiales;D_4__Ruminococcaceae;D_5__GCA-900066225;D_6__uncultured bacterium;D_7__;D_8__;D_9__;D_10__;D_11__;D_12__;D_13__;D_14__	1.88(0.06,60.63)	1.09(0.04,31.04)	2.06(0.13,32.12)						
ASV_1010	Firmicutes;D_2__Clostridia;D_3__Clostridiales;D_4__Ruminococcaceae;D_5__Ruminiclostridium 5;D_6__uncultured organism;D_7__;D_8__;D_9__;D_10__;D_11__;D_12__;D_13__;D_14__	0.90(0.04,20.55)	1.00(0.05,19.89)	0.90(0.08,10.58)	0.55(0.02,12.58)	1.45(0.08,27.69)	0.80(0.06,9.76)			
ASV_1013	Actinobacteria;D_2__Actinobacteria;D_3__Corynebacteriales;D_4__Corynebacteriaceae;D_5__Corynebacterium;D_6__Corynebacterium durum;D_7__;D_8__;D_9__;D_10__;D_11__;D_12__;D_13__;D_14__				1.47(0.25,8.73)	1.43(0.26,8.02)	2.10(0.51,8.65)			
ASV_1018	Firmicutes;D_2__Clostridia;D_3__Clostridiales;D_4__Christensenellaceae;D_5__Christensenellaceae R-7 group;D_6__uncultured bacterium;D_7__;D_8__;D_9__;D_10__;D_11__;D_12__;D_13__;D_14__	3.27(0.25,42.08)	1.13(0.09,13.60)	3.71(0.50,27.61)						
ASV_1019	Firmicutes;D_2__Clostridia;D_3__Clostridiales;D_4__Lachnospiraceae;D_5__Sellimonas;D_6__uncultured Firmicutes bacterium;D_7__;D_8__;D_9__;D_10__;D_11__;D_12__;D_13__;D_14__	11.24(0.23,538.20)	0.55(0.01,23.54)	6.16(0.31,120.93)	20.04(0.48,834.53)	0.74(0.02,28.51)	14.91(0.85,261.45)			
ASV_1020	Firmicutes;D_2__Erysipelotrichia;D_3__Erysipelotrichales;D_4__Erysipelotrichaceae;D_5__Erysipelatoclostridium;D_6__Massiliomicrobiota timonensis;D_7__;D_8__;D_9__;D_10__;D_11__;D_12__;D_13__;D_14__	0.24(0.02,2.44)	4.39(0.47,41.20)	1.03(0.15,7.08)						

ASV_1022	Firmicutes;D_2__Clostridia;D_3__Clostridiales;D_4__Ruminococcaceae	1.09(0.04,31.60)	3.19(0.10,100.81)	3.48(0.19,62.57)						
ASV_1024	Firmicutes;D_2__Clostridia;D_3__Clostridiales;D_4__Lachnospiraceae				2.77(0.11,67.83)	2.89(0.11,74.52)	8.00(0.60,105.83)			
ASV_1028	Firmicutes;D_2__Clostridia;D_3__Clostridiales;D_4__Ruminococcaceae;D_5__Fournierella				0.00(0.00,Inf)	224366624870891.00(0.00,Inf)	3.02(0.00,Inf)			
ASV_1029	Firmicutes;D_2__Clostridia;D_3__Clostridiales;D_4__Ruminococcaceae;D_5__uncultured;D_6__uncultured Firmicutes bacterium;D_7__;D_8__;D_9__;D_10__;D_11__;D_12__;D_13__;D_14__	1.32(0.10,16.79)	1.24(0.11,14.62)	1.64(0.22,12.19)						
ASV_1030	Firmicutes;D_2__Clostridia;D_3__Clostridiales;D_4__Ruminococcaceae	0.48(0.07,3.51)	0.37(0.06,2.36)	0.18(0.04,0.86);p=0.03124	0.42(0.06,3.08)	0.41(0.07,2.61)	0.17(0.04,0.84);p=0.02907			↓
ASV_1031	Actinobacteria;D_2__Actinobacteria;D_3__Bifidobacteriales;D_4__Bifidobacteriaceae;D_5__Scardovia;D_6__unidentified;D_7__;D_8__;D_9__;D_10__;D_11__;D_12__;D_13__;D_14__	2.69(0.07,103.32)	1.31(0.04,48.15)	3.52(0.20,61.24)						
ASV_1040	Firmicutes;D_2__Bacilli;D_3__Lactobacillales;D_4__Lactobacillaceae;D_5__Lactobacillus	0.00(0.00,0.18);p=0.0039	47.51(2.45,922.55);p=0.01073	0.24(0.01,6.00)	0.01(0.00,0.22);p=0.00493	54.25(2.81,1046.18);p=0.00817	0.35(0.02,8.33)	↓	↑	
ASV_1048	Bacteroidetes;D_2__Bacteroidia;D_3__Bacteroidales;D_4__Prevotellaceae;D_5__Paraprevotella	2.69(0.06,124.46)	0.33(0.01,13.31)	0.90(0.04,18.77)						
ASV_1049	Firmicutes;D_2__Bacilli;D_3__Lactobacillales;D_4__Lactobacillaceae;D_5__Lactobacillus;D_6__Lactobacillus rhamnosus;D_7__;D_8__;D_9__;D_10__;D_11__;D_12__;D_13__;D_14__	7.10(0.19,262.49)	18.07(0.51,639.17)	128.34(6.59,2499.63);p=0.00135	9.08(0.26,320.50)	26.52(0.63,1115.20)	240.84(11.04,5255.21);p=0.00049			↑
ASV_1052	Firmicutes;D_2__Clostridia;D_3__Clostridiales;D_4__Ruminococcaceae;D_5__Anaerofilum;D_6__uncultured bacterium;D_7__;D_8__;D_9__;D_10__;D_11__;D_12__;D_13__;D_14__	0.53(0.07,4.07)	1.07(0.15,7.42)	0.57(0.11,2.88)	0.52(0.07,3.99)	1.30(0.19,8.87)	0.68(0.14,3.41)			
ASV_1055	Firmicutes;D_2__Clostridia;D_3__Clostridiales;D_4__Ruminococcaceae;D_5__GCA-900066225;D_6__uncultured bacterium;D_7__;D_8__;D_9__;D_10__;D_11__;D_12__;D_13__;D_14__	3.29(0.17,62.96)	0.34(0.02,5.87)	1.13(0.11,11.61)	0.97(0.06,15.69)	1.68(0.12,23.88)	1.63(0.18,14.81)			
ASV_1057	Actinobacteria;D_2__Coriobacteriia;D_3__Coriobacteriales;D_4__Coriobacteriaceae;D_5__Collinsella;D_6__uncultured bacterium;D_7__;D_8__;D_9__;D_10__;D_11__;D_12__;D_13__;D_14__	0.48(0.06,3.64)	0.81(0.12,5.48)	0.39(0.08,1.95)	0.52(0.07,3.79)	1.05(0.16,6.83)	0.54(0.11,2.66)			
ASV_1059	Firmicutes;D_2__Clostridia;D_3__Clostridiales;D_4__Ruminococcaceae;D_5__uncultured	23.27(0.41,1327.32)	1.56(0.03,80.66)	36.34(1.48,891.62);p=0.02778						
ASV_1062	Firmicutes;D_2__Clostridia;D_3__Clostridiales;D_4__Ruminococcaceae;D_5__GCA-900066225;D_6__uncultured Ruminococcus sp.;D_7__;D_8__;D_9__;D_10__;D_11__;D_12__;D_13__;D_14__	0.41(0.03,6.50)	0.55(0.04,7.21)	0.22(0.02,2.01)	0.71(0.05,10.69)	0.54(0.04,6.69)	0.38(0.04,3.21)			
ASV_1066	Actinobacteria;D_2__Actinobacteria;D_3__Corynebacteriales;D_4__Corynebacteriaceae;D_5__Corynebacterium 1;D_6__Corynebacterium pseudodiphtheriticum;D_7__;D_8__;D_9__;D_10__;D_11__;D_12__;D_13__;D_14__	0.00(0.00,Inf)	2.80(0.07,112.54)	0.00(0.00,Inf)						
ASV_1068	Bacteroidetes;D_2__Bacteroidia;D_3__Bacteroidales;D_4__Tannerellaceae;D_5__Parabacteroides	0.00(0.00,Inf)	4.56(0.57,36.56)	0.00(0.00,Inf)	0.00(0.00,Inf)	4.76(0.58,39.24)	0.00(0.00,Inf)			
ASV_1070	Firmicutes;D_2__Clostridia;D_3__Clostridiales;D_4__Lachnospiraceae;D_5__Lactonifactor	0.93(0.06,14.72)	0.56(0.04,7.66)	0.52(0.06,4.39)	1.08(0.07,17.32)	0.50(0.04,6.75)	0.54(0.06,4.47)			
ASV_1072	Firmicutes;D_2__Clostridia;D_3__Clostridiales;D_4__Lachnospiraceae;D_5__Lachnoclostridium	17.64(0.74,418.03)	0.19(0.01,4.21)	3.42(0.34,34.62)	31.44(1.24,796.23);p=0.03653	0.23(0.01,5.20)	7.18(0.71,72.68)	↑		
ASV_1104	Firmicutes;D_2__Clostridia;D_3__Clostridiales;D_4__Lachnospiraceae;D_5__Lachnoclostridium	3103159948322.27(0.00,Inf)	0.00(0.00,Inf)	4.14(0.14,121.55)						
ASV_1107	Firmicutes;D_2__Clostridia;D_3__Clostridiales;D_4__Ruminococcaceae;D_5__UBA1819	2.09(0.03,168.64)	0.19(0.00,12.96)	0.41(0.01,11.18)						

ASV_1114	Firmicutes;D_2__Clostridia;D_3__Clostridiales;D_4__Ruminococcaceae;D_5__Butyricicoccus;D_6__uncultured bacterium;D_7__;D_8__;D_9__;D_10__;D_11__;D_12__;D_13__;D_14__	11043253361147.00(0.00,Inf)	0.00(0.00,Inf)	2.92(0.05,157.06)						
ASV_1117	Firmicutes;D_2__Clostridia;D_3__Clostridiales;D_4__Lachnospiraceae;D_5__Lachnospiraceae NK4A136 group;D_6__uncultured organism;D_7__;D_8__;D_9__;D_10__;D_11__;D_12__;D_13__;D_14__	0.53(0.01,55.71)	0.84(0.01,64.11)	0.44(0.01,17.91)						
ASV_1129	Bacteroidetes;D_2__Bacteroidia;D_3__Bacteroidales;D_4__Prevotellaceae;D_5__Prevotella;D_6__Prevotella buccalis;D_7__;D_8__;D_9__;D_10__;D_11__;D_12__;D_13__;D_14__	4.56(0.34,61.91)	0.19(0.02,2.39)	0.88(0.13,6.23)	4.81(0.34,68.57)	0.14(0.01,1.81)	0.68(0.10,4.45)			
ASV_1131	Firmicutes;D_2__Clostridia;D_3__Clostridiales;D_4__Peptostreptococcaceae	235916001.92(0.00,Inf)	0.00(0.00,Inf)	0.07(0.00,1.42)						
ASV_1132	Firmicutes;D_2__Bacilli;D_3__Lactobacillales;D_4__Streptococcaceae;D_5__Streptococcus	0.96(0.05,19.18)	0.16(0.01,2.67)	0.15(0.02,1.51)	0.53(0.03,9.10)	0.38(0.03,5.15)	0.20(0.02,1.92)			
ASV_1143	Firmicutes;D_2__Erysipelotrichia;D_3__Erysipelotrichales;D_4__Erysipelotrichaceae;D_5__Coprobacillus;D_6__uncultured bacterium;D_7__;D_8__;D_9__;D_10__;D_11__;D_12__;D_13__;D_14__	7.07(0.17,290.32)	0.02(0.00,0.63);p=0.02674	0.12(0.01,2.31)						
ASV_1147	Firmicutes;D_2__Bacilli;D_3__Lactobacillales;D_4__Streptococcaceae;D_5__Streptococcus	0.41(0.04,4.02)	1.10(0.13,9.53)	0.45(0.07,2.81)	0.39(0.04,4.03)	0.91(0.10,7.93)	0.36(0.06,2.27)			
ASV_1150	Firmicutes;D_2__Bacilli;D_3__Lactobacillales;D_4__Streptococcaceae;D_5__Streptococcus	5.47(0.11,275.48)	0.37(0.01,16.28)	2.03(0.09,43.68)						
ASV_1151	Firmicutes;D_2__Negativicutes;D_3__Selenomonadales;D_4__Veillonellaceae;D_5__Dialister;D_6__uncultured bacterium;D_7__;D_8__;D_9__;D_10__;D_11__;D_12__;D_13__;D_14__	1825595835.36(0.00,Inf)	0.00(0.00,Inf)	0.33(0.03,4.11)						
ASV_1156	Bacteroidetes;D_2__Bacteroidia;D_3__Bacteroidales;D_4__Prevotellaceae;D_5__Prevotella 7;D_6__uncultured Bacteroidetes bacterium;D_7__;D_8__;D_9__;D_10__;D_11__;D_12__;D_13__;D_14__	23716017299.58(0.00,Inf)	0.00(0.00,Inf)	29.27(1.86,460.18);p=0.0163						
ASV_1162	Firmicutes;D_2__Clostridia;D_3__Clostridiales;D_4__Ruminococcaceae;D_5__[Eubacterium] coprostanoligenes group;D_6__uncultured organism;D_7__;D_8__;D_9__;D_10__;D_11__;D_12__;D_13__;D_14__				1777501641641746.00(0.00,Inf)	0.00(0.00,Inf)	0.88(0.08,9.55)			
ASV_1164	Actinobacteria;D_2__Actinobacteria;D_3__Actinomycetales;D_4__Actinomycetaceae;D_5__Actinomyces	11.25(0.85,149.72)	0.40(0.03,5.06)	4.49(0.68,29.47)	17.09(1.16,251.00);p=0.03844	0.30(0.02,4.17)	5.17(0.79,33.71)	↑		
ASV_1196	Firmicutes;D_2__Clostridia;D_3__Clostridiales;D_4__Ruminococcaceae;D_5__uncultured	1.51(0.03,70.15)	0.45(0.01,17.52)	0.67(0.04,12.27)						
ASV_1208	Firmicutes;D_2__Erysipelotrichia;D_3__Erysipelotrichales;D_4__Erysipelotrichaceae;D_5__[Clostridium] innocuum group	918445610.50(0.00,Inf)	0.00(0.00,Inf)	0.37(0.02,6.61)	1282719999787251.00(0.00,Inf)	0.00(0.00,Inf)	1.68(0.10,28.15)			
ASV_1209	Firmicutes;D_2__Clostridia;D_3__Clostridiales;D_4__Ruminococcaceae;D_5__Papillibacter;D_6__uncultured bacterium;D_7__;D_8__;D_9__;D_10__;D_11__;D_12__;D_13__;D_14__				0.60(0.06,5.60)	2.80(0.31,25.25)	1.69(0.28,10.30)			
ASV_1216	Firmicutes;D_2__Clostridia;D_3__Clostridiales;D_4__Ruminococcaceae	15265054968.59(0.00,Inf)	0.00(0.00,Inf)	0.47(0.03,6.50)						
ASV_1217	Firmicutes;D_2__Clostridia;D_3__Clostridiales;D_4__Christensenellaceae;D_5__Christensenellaceae R-7 group	2911913569.44(0.00,Inf)	0.00(0.00,Inf)	0.27(0.03,2.45)						
ASV_1218	Firmicutes;D_2__Clostridia;D_3__Clostridiales;D_4__Lachnospiraceae;D_5__[Eubacterium] hallii group;D_6__uncultured bacterium;D_7__;D_8__;D_9__;D_10__;D_11__;D_12__;D_13__;D_14__	0.09(0.00,3.90)	7.04(0.21,236.87)	0.60(0.02,16.24)						
ASV_1220	Bacteroidetes;D_2__Bacteroidia;D_3__Bacteroidales;D_4__Prevotellaceae;D_5__Prevotella;D_6__Chlamydia trachomatis;D_7__;D_8__;D_9__;D_10__;D_11__;D_12__;D_13__;D_14__	0.78(0.06,10.45)	3.84(0.31,47.57)	2.98(0.37,23.99)	0.78(0.06,10.79)	2.76(0.22,34.00)	2.16(0.27,17.50)			
ASV_1221	Firmicutes;D_2__Clostridia;D_3__Clostridiales;D_4__Ruminococcaceae;D_5__Faecalibacterium	942473898.80(0.00,Inf)	0.00(0.00,Inf)	0.27(0.01,6.35)	7554365579731.21(0.00,Inf)	0.00(0.00,Inf)	0.27(0.01,6.04)			

ASV_1222	Firmicutes;D_2__Bacilli;D_3__Lactobacillales;D_4__Lactobacillaceae;D_5__Lactobacillus;D_6__Lactobacillus rhamnosus;D_7__;D_8__;D_9__;D_10__;D_11__;D_12__;D_13__;D_14__				0.38(0.00,Inf)	0.00(0.00,Inf)	0.00(0.00,Inf)			
ASV_1223	Firmicutes;D_2__Clostridia;D_3__Clostridiales;D_4__Ruminococcaceae				1996263307817491.00(0.00,Inf)	0.00(0.00,Inf)	0.89(0.07,11.28)			
ASV_1227	Firmicutes;D_2__Clostridia;D_3__Clostridiales;D_4__Ruminococcaceae;D_5__Ruminiclostridium 5;D_6__uncultured bacterium;D_7__;D_8__;D_9__;D_10__;D_11__;D_12__;D_13__;D_14__	2.96(0.12,72.59)	0.65(0.03,14.67)	1.91(0.17,21.31)						
ASV_1228	Firmicutes;D_2__Clostridia;D_3__Clostridiales;D_4__Christensenellaceae;D_5__uncultured;D_6__uncultured bacterium;D_7__;D_8__;D_9__;D_10__;D_11__;D_12__;D_13__;D_14__	15270607690.67(0.00,Inf)	0.00(0.00,Inf)	1.36(0.13,14.58)	2020626159117119.00(0.00,Inf)	0.00(0.00,Inf)	1.42(0.13,15.44)			
ASV_1231	Firmicutes;D_2__Clostridia;D_3__Clostridiales;D_4__Lachnospiraceae	0.70(0.02,23.52)	0.34(0.01,8.96)	0.24(0.02,3.60)						
ASV_1233	Firmicutes;D_2__Bacilli;D_3__Lactobacillales;D_4__Streptococcaceae;D_5__Streptococcus	441558271790.76(0.00,Inf)	0.00(0.00,Inf)	5.00(0.33,75.20)						
ASV_1248	Firmicutes;D_2__Clostridia;D_3__Clostridiales;D_4__Ruminococcaceae;D_5__Ruminococcaceae UCG-002	0.00(0.00,Inf)	27.71(1.38,556.53);p=0.03	0.00(0.00,Inf)						
ASV_1253	Firmicutes;D_2__Clostridia;D_3__Clostridiales;D_4__Lachnospiraceae;D_5__Fusicatenibacter	0.14(0.00,20.09)	1.97(0.02,214.38)	0.28(0.01,14.96)						
ASV_1257	Firmicutes;D_2__Clostridia;D_3__Clostridiales;D_4__Lachnospiraceae;D_5__[Ruminococcus] torques group	1.49(0.01,153.26)	0.23(0.00,19.80)	0.35(0.01,12.82)						
ASV_1260	Firmicutes;D_2__Bacilli;D_3__Lactobacillales;D_4__Carnobacteriaceae;D_5__Granulicatella	1.21(0.04,38.71)	2.06(0.06,66.27)	2.48(0.14,42.79)						
ASV_1262	Firmicutes;D_2__Clostridia;D_3__Clostridiales;D_4__Lachnospiraceae	0.00(0.00,Inf)	28.16(1.15,690.34);p=0.04088	0.00(0.00,Inf)						
ASV_1286	Deinococcus-Thermus;D_2__Deinococci;D_3__Thermales;D_4__Thermaceae;D_5__Meiothermus	8620158393.42(0.00,Inf)	0.00(0.00,Inf)	1.41(0.07,29.45)						
ASV_1287	Firmicutes;D_2__Clostridia;D_3__Clostridiales;D_4__Lachnospiraceae;D_5__[Ruminococcus] gnavus group	1.13(0.02,56.18)	1.18(0.03,51.18)	1.34(0.06,29.38)						
ASV_1289	Firmicutes;D_2__Erysipelotrichia;D_3__Erysipelotrichales;D_4__Erysipelotrichaceae;D_5__Holdemania;D_6__uncultured bacterium;D_7__;D_8__;D_9__;D_10__;D_11__;D_12__;D_13__;D_14__	284671877516.18(0.00,Inf)	0.00(0.00,Inf)	8.18(0.44,153.97)						
ASV_1294	Proteobacteria;D_2__Gammaproteobacteria;D_3__Betaproteobacteriales;D_4__Burkholderiaceae;D_5__Sutterella;D_6__uncultured organism;D_7__;D_8__;D_9__;D_10__;D_11__;D_12__;D_13__;D_14__	9.39(0.15,572.89)	0.27(0.01,14.22)	2.54(0.10,63.38)						
ASV_1301	Firmicutes;D_2__Clostridia;D_3__Clostridiales;D_4__Ruminococcaceae;D_5__Candidatus Soleaferrea	322459132919.95(0.00,Inf)	0.00(0.00,Inf)	2.09(0.07,64.49)						
ASV_1302	Firmicutes;D_2__Clostridia;D_3__Clostridiales;D_4__Ruminococcaceae;D_5__Ruminiclostridium 9	6896184578.13(0.00,Inf)	0.00(0.00,Inf)	0.89(0.04,19.06)						
ASV_1303	Firmicutes;D_2__Clostridia;D_3__Clostridiales;D_4__Ruminococcaceae;D_5__uncultured	7.48(0.39,144.28)	0.05(0.00,0.94);p=0.04529	0.41(0.04,3.75)	9.75(0.46,205.85)	0.05(0.00,0.87);p=0.0398	0.45(0.05,3.81)		↓	
ASV_1305	Firmicutes;D_2__Clostridia;D_3__Clostridiales;D_4__Ruminococcaceae;D_5__Papillibacter;D_6__uncultured bacterium;D_7__;D_8__;D_9__;D_10__;D_11__;D_12__;D_13__;D_14__	0.66(0.01,39.13)	0.89(0.02,41.93)	0.59(0.02,14.77)						
ASV_1306	Actinobacteria;D_2__Actinobacteria;D_3__Actinomycetales;D_4__Actinomycetaceae;D_5__F0332;D_6__uncultured bacterium;D_7__;D_8__;D_9__;D_10__;D_11__;D_12__;D_13__;D_14__	7048730620.77(0.00,Inf)	0.00(0.00,Inf)	4.74(0.71,31.71)						

ASV_1344	Firmicutes;D_2__Clostridia;D_3__Clostridiales;D_4__Family XIII;D_5__Family XIII AD3011 group	0.56(0.01,25.73)	1.18(0.03,43.30)	0.66(0.03,14.20)						
ASV_1349	Firmicutes;D_2__Clostridia;D_3__Clostridiales;D_4__Lachnospiraceae;D_5__Lachnospiraceae NK4A136 group	0.00(0.00,Inf)	10.29(0.27,395.13)	0.00(0.00,Inf)						
ASV_1357	Firmicutes;D_2__Clostridia;D_3__Clostridiales;D_4__Ruminococcaceae;D_5__Ruminiclostridium 1;D_6__uncultured bacterium;D_7__;D_8__;D_9__;D_10__;D_11__;D_12__;D_13__;D_14__	0.23(0.01,10.08)	1.31(0.03,50.22)	0.30(0.01,6.06)	0.55(0.01,24.19)	1.18(0.03,43.20)	0.65(0.03,12.96)			
ASV_1362	Actinobacteria;D_2__Actinobacteria;D_3__Micrococcales;D_4__Micrococcaceae;D_5__Rothia	0.56(0.05,5.89)	2.82(0.29,27.43)	1.58(0.23,10.68)	1.44(0.15,13.85)	2.18(0.22,21.84)	3.15(0.54,18.54)			
ASV_1364	Firmicutes;D_2__Clostridia;D_3__Clostridiales;D_4__Lachnospiraceae;D_5__GCA-900066575;D_6__uncultured bacterium;D_7__;D_8__;D_9__;D_10__;D_11__;D_12__;D_13__;D_14__	84504320718.23(0.00,Inf)	0.00(0.00,Inf)	0.63(0.03,12.47)						
ASV_1369	Firmicutes;D_2__Clostridia;D_3__Clostridiales;D_4__Clostridiales vadinBB60 group;D_5__uncultured bacterium;D_6__;D_7__;D_8__;D_9__;D_10__;D_11__;D_12__;D_13__;D_14__	5961181581.49(0.00,Inf)	0.00(0.00,Inf)	3.14(0.12,80.45)						
ASV_1371	Firmicutes;D_2__Clostridia;D_3__Clostridiales;D_4__Ruminococcaceae;D_5__Ruminococcaceae UCG-010;D_6__uncultured organism;D_7__;D_8__;D_9__;D_10__;D_11__;D_12__;D_13__;D_14__	0.66(0.01,55.12)	0.52(0.01,33.14)	0.34(0.01,10.94)						
ASV_1374	Firmicutes;D_2__Clostridia;D_3__Clostridiales;D_4__Ruminococcaceae;D_5__Ruminococcaceae NK4A214 group	1.97(0.04,86.26)	552734380929935.00(0.00,Inf)	1086759081326153.00(0.00,Inf)						
ASV_1375	Bacteroidetes;D_2__Bacteroidia;D_3__Bacteroidales;D_4__Rikenellaceae;D_5__Alistipes;D_6__uncultured bacterium;D_7__;D_8__;D_9__;D_10__;D_11__;D_12__;D_13__;D_14__	3.99(0.30,53.29)	3.59(0.15,85.53)	14.31(1.17,175.71);p=0.03756	3.31(0.26,41.46)	4.98(0.20,122.15)	16.48(1.18,230.54);p=0.03735			↑
ASV_1376	Proteobacteria;D_2__Gammaproteobacteria;D_3__Betaproteobacteriales;D_4__Burkholderiaceae;D_5__Aquabacterium;D_6__Aquabacterium citratiphilum;D_7__;D_8__;D_9__;D_10__;D_11__;D_12__;D_13__;D_14__	2.09(0.09,48.28)	1.50(0.06,36.24)	3.14(0.26,37.52)	1.00(0.04,26.73)	4.07(0.11,148.90)	4.08(0.28,59.32)			
ASV_1378	Firmicutes;D_2__Erysipelotrichia;D_3__Erysipelotrichales;D_4__Erysipelotrichaceae;D_5__Merdibacter;D_6__uncultured bacterium;D_7__;D_8__;D_9__;D_10__;D_11__;D_12__;D_13__;D_14__	8.05(0.60,107.20)	0.19(0.02,2.42)	1.56(0.27,9.05)	7.37(0.44,124.79)	0.15(0.01,2.36)	1.08(0.20,5.95)			
ASV_1393	Firmicutes;D_2__Clostridia;D_3__Clostridiales;D_4__Ruminococcaceae;D_5__Intestinimonas;D_6__uncultured Clostridiales bacterium;D_7__;D_8__;D_9__;D_10__;D_11__;D_12__;D_13__;D_14__				8.96(0.34,236.37)	0.30(0.01,7.38)	2.71(0.26,28.08)			
ASV_1422	Firmicutes;D_2__Clostridia;D_3__Clostridiales;D_4__Lachnospiraceae;D_5__Lachnoclostridium	39345201314.46(0.00,Inf)	0.00(0.00,Inf)	2.48(0.19,32.34)						
ASV_1424	Bacteroidetes;D_2__Bacteroidia;D_3__Bacteroidales;D_4__Marinifilaceae;D_5__Butyricimonas;D_6__uncultured organism;D_7__;D_8__;D_9__;D_10__;D_11__;D_12__;D_13__;D_14__	0.02(0.00,0.40);p=0.01204	20.00(1.51,265.70);p=0.02319	0.31(0.01,6.46)	0.02(0.00,0.45);p=0.01435	19.67(1.43,270.23);p=0.02582	0.36(0.02,6.88)	↓	↑	
ASV_1443	Firmicutes;D_2__Clostridia;D_3__Clostridiales;D_4__Peptococcaceae;D_5__uncultured	0.11(0.00,3.99)	21.44(0.63,735.67)	2.35(0.11,51.50)						
ASV_1453	Bacteroidetes;D_2__Bacteroidia;D_3__Bacteroidales;D_4__Bacteroidaceae;D_5__Bacteroides;D_6__uncultured bacterium;D_7__;D_8__;D_9__;D_10__;D_11__;D_12__;D_13__;D_14__	0.05(0.00,2.70)	1.85(0.05,74.96)	0.09(0.00,2.38)						
ASV_1464	Firmicutes;D_2__Clostridia;D_3__Clostridiales;D_4__Ruminococcaceae;D_5__Subdoligranulum;D_6__uncultured bacterium;D_7__;D_8__;D_9__;D_10__;D_11__;D_12__;D_13__;D_14__	0.00(0.00,Inf)	10.85(0.85,138.60)	0.00(0.00,Inf)	0.00(0.00,Inf)	12.60(0.83,191.78)	0.00(0.00,Inf)			
ASV_1465	Bacteroidetes;D_2__Bacteroidia;D_3__Bacteroidales;D_4__Prevotellaceae;D_5__Prevotella 7;D_6__uncultured bacterium;D_7__;D_8__;D_9__;D_10__;D_11__;D_12__;D_13__;D_14__	0.00(0.00,Inf)	1.43(0.04,45.58)	0.00(0.00,Inf)						
ASV_1515	Firmicutes;D_2__Clostridia;D_3__Clostridiales;D_4__Christensenellaceae;D_5__Christensenellaceae R-7 group;D_6__gut metagenome;D_7__;D_8__;D_9__;D_10__;D_11__;D_12__;D_13__;D_14__	0.04(0.00,6.04)	58.87(0.45,7640.56)	2.53(0.04,175.70)						
ASV_1517	Firmicutes;D_2__Bacilli;D_3__Lactobacillales;D_4__Lactobacillaceae;D_5__Lactobacillus	3158912025.73(0.00,Inf)	0.00(0.00,Inf)	0.50(0.03,8.99)						

ASV_1520	Actinobacteria;D_2__Actinobacteria;D_3__Bifidobacteriales;D_4__Bifidobacteriaceae;D_5__Bifidobacterium	1.77(0.08,41.33)	0.78(0.04,16.84)	1.39(0.13,15.06)						
ASV_1524	Bacteroidetes;D_2__Bacteroidia;D_3__Bacteroidales;D_4__Porphyromonadaceae;D_5__Porphyromonas;D_6__Porphyromonas asaccharolytica;D_7__;D_8__;D_9__;D_10__;D_11__;D_12__;D_13__;D_14__	19601058410.84(0.00,Inf)	0.00(0.00,Inf)	115.74(4.44,3013.70);p=0.00428						
ASV_1526	Firmicutes;D_2__Clostridia;D_3__Clostridiales;D_4__Lachnospiraceae;D_5__uncultured;D_6__intestinal bacterium CG19-1;D_7__;D_8__;D_9__;D_10__;D_11__;D_12__;D_13__;D_14__	1.12(0.10,11.98)	15.41(0.84,281.90)	17.23(1.30,229.06);p=0.03108						
ASV_1527	Firmicutes;D_2__Clostridia;D_3__Clostridiales;D_4__Ruminococcaceae;D_5__uncultured;D_6__uncultured bacterium;D_7__;D_8__;D_9__;D_10__;D_11__;D_12__;D_13__;D_14__	4842433556.85(0.00,Inf)	0.00(0.00,Inf)	6.42(0.35,118.80)						
ASV_1530	Proteobacteria;D_2__Gammaproteobacteria;D_3__Betaproteobacteriales;D_4__Burkholderiaceae;D_5__Sutterella	0.33(0.00,27.57)	0.33(0.01,16.13)	0.11(0.00,3.71)						
ASV_1531	Firmicutes;D_2__Clostridia;D_3__Clostridiales;D_4__Ruminococcaceae;D_5__uncultured;D_6__Ruminococcaceae bacterium Marseille-P3738;D_7__;D_8__;D_9__;D_10__;D_11__;D_12__;D_13__;D_14__	0.15(0.00,5.42)	22342794841.98(0.00,Inf)	3415144688.60(0.00,Inf)						
ASV_1554	Bacteroidetes;D_2__Bacteroidia;D_3__Bacteroidales;D_4__Prevotellaceae;D_5__Prevotella 6;D_6__uncultured bacterium;D_7__;D_8__;D_9__;D_10__;D_11__;D_12__;D_13__;D_14__	12642434606.80(0.00,Inf)	0.00(0.00,Inf)	61.08(2.66,1400.89);p=0.01009						
ASV_1556	Firmicutes;D_2__Clostridia;D_3__Clostridiales;D_4__Lachnospiraceae;D_5__Lachnoclostridium	0.18(0.00,8.58)	50.63(0.84,3052.87)	9.19(0.25,339.22)						
ASV_1569	Firmicutes;D_2__Bacilli;D_3__Lactobacillales;D_4__Leuconostocaceae;D_5__Leuconostoc;D_6__uncultured bacterium;D_7__;D_8__;D_9__;D_10__;D_11__;D_12__;D_13__;D_14__	1.64(0.05,49.56)	1.42(0.05,38.48)	2.33(0.16,34.81)	1.27(0.04,37.24)	5.05(0.19,135.14)	6.40(0.42,98.53)			
ASV_1571	Firmicutes;D_2__Clostridia;D_3__Clostridiales;D_4__Lachnospiraceae;D_5__Sellimonas;D_6__uncultured bacterium;D_7__;D_8__;D_9__;D_10__;D_11__;D_12__;D_13__;D_14__	3176664047.97(0.00,Inf)	0.00(0.00,Inf)	0.80(0.06,11.60)	680950525937373.00(0.00,Inf)	0.00(0.00,Inf)	0.82(0.06,10.86)			
ASV_1572	Firmicutes;D_2__Clostridia;D_3__Clostridiales;D_4__Lachnospiraceae;D_5__Lachnospira	6467223637.87(0.00,Inf)	0.00(0.00,Inf)	2.15(0.06,82.09)						
ASV_1574	Bacteroidetes;D_2__Bacteroidia;D_3__Bacteroidales;D_4__Tannerellaceae;D_5__Parabacteroides	62583313325.71(0.00,Inf)	0.00(0.00,Inf)	0.13(0.00,5.34)						
ASV_1589	Firmicutes;D_2__Clostridia;D_3__Clostridiales;D_4__Ruminococcaceae;D_5__Flavonifractor	26076580584.46(0.00,Inf)	0.00(0.00,Inf)	5.84(0.36,93.64)						
ASV_1653	Bacteroidetes;D_2__Bacteroidia;D_3__Bacteroidales;D_4__Prevotellaceae;D_5__Prevotella;D_6__Chlamydia trachomatis;D_7__;D_8__;D_9__;D_10__;D_11__;D_12__;D_13__;D_14__				0.68(0.02,25.14)	2.26(0.07,72.66)	1.54(0.09,27.23)			
ASV_1654	Actinobacteria;D_2__Actinobacteria;D_3__Bifidobacteriales;D_4__Bifidobacteriaceae;D_5__Gardnerella;D_6__uncultured bacterium;D_7__;D_8__;D_9__;D_10__;D_11__;D_12__;D_13__;D_14__	0.18(0.00,10.56)	1.83(0.04,75.69)	0.32(0.01,9.47)						
ASV_1661	Proteobacteria;D_2__Gammaproteobacteria;D_3__Betaproteobacteriales;D_4__Burkholderiaceae;D_5__Tepidimonas	6663752171989.03(0.00,Inf)	0.00(0.00,Inf)	0.67(0.03,15.61)						
ASV_1663	Bacteroidetes;D_2__Bacteroidia;D_3__Bacteroidales;D_4__Porphyromonadaceae;D_5__Porphyromonas	0.98(0.02,43.14)	0.89(0.02,33.46)	0.87(0.04,17.04)						
ASV_1714	Firmicutes;D_2__Clostridia;D_3__Clostridiales;D_4__Ruminococcaceae	6750713090.93(0.00,Inf)	0.00(0.00,Inf)	1.43(0.12,16.89)	2824198440065964.00(0.00,Inf)	0.00(0.00,Inf)	0.36(0.04,3.73)			
ASV_1719	Actinobacteria;D_2__Actinobacteria;D_3__Micrococcales;D_4__Micrococcaceae;D_5__Rothia;D_6__uncultured bacterium;D_7__;D_8__;D_9__;D_10__;D_11__;D_12__;D_13__;D_14__	0.74(0.05,10.92)	1.74(0.13,23.24)	1.29(0.15,11.33)	0.26(0.02,3.61)	3.77(0.30,47.00)	0.96(0.11,8.73)			
ASV_1721	Actinobacteria;D_2__Coriobacteriia;D_3__Coriobacteriales;D_4__Atopobiaceae;D_5__Atopobium;D_6__uncultured bacterium;D_7__;D_8__;D_9__;D_10__;D_11__;D_12__;D_13__;D_14__	1.96(0.10,37.17)	1.63(0.09,29.34)	3.19(0.31,33.15)	2.97(0.15,57.88)	1.47(0.08,27.10)	4.36(0.42,44.87)			

ASV_1732	Firmicutes;D_2__Clostridia;D_3__Clostridiales;D_4__Lachnospiraceae	0.01(0.00,0.34);p=0.01047	1.31(0.05,32.83)	0.01(0.00,0.23);p=0.0033	0.01(0.00,0.38);p=0.01319	1.02(0.04,25.08)	0.01(0.00,0.21);p=0.00306	↓		↓
ASV_1736	Firmicutes;D_2__Clostridia;D_3__Clostridiales;D_4__Lachnospiraceae;D_5__Sellimonas;D_6__uncultured organism;D_7__;D_8__;D_9__;D_10__;D_11__;D_12__;D_13__;D_14__	0.10(0.00,4.15)	14.77(0.41,526.61)	1.49(0.08,29.09)	0.05(0.00,1.91)	20.82(0.61,713.32)	0.97(0.05,18.85)			
ASV_1741	Firmicutes;D_2__Clostridia;D_3__Clostridiales;D_4__Lachnospiraceae;D_5__Lachnoclostridium	0.00(0.00,Inf)	1.72(0.03,91.46)	0.00(0.00,Inf)						
ASV_1748	Firmicutes;D_2__Clostridia;D_3__Clostridiales;D_4__Lachnospiraceae;D_5__Sellimonas	27148061578.54(0.00,Inf)	0.00(0.00,Inf)	16.89(0.71,399.28)	474411596197123.00(0.00,Inf)	0.00(0.00,Inf)	15.63(0.68,358.54)			
ASV_1751	Firmicutes;D_2__Clostridia;D_3__Clostridiales;D_4__Lachnospiraceae;D_5__[Eubacterium] hallii group;D_6__uncultured bacterium;D_7__;D_8__;D_9__;D_10__;D_11__;D_12__;D_13__;D_14__	4.41(0.07,299.03)	2.38(0.03,221.57)	10.49(0.31,353.04)	4.53(0.06,320.36)	2.91(0.03,290.39)	13.18(0.35,498.91)			
ASV_1756	Firmicutes;D_2__Clostridia;D_3__Clostridiales;D_4__Clostridiaceae 1;D_5__Clostridium sensu stricto 1	3382440930.41(0.00,Inf)	0.00(0.00,Inf)	0.43(0.02,9.98)						
ASV_1761	Proteobacteria;D_2__Alphaproteobacteria;D_3__Caulobacterales;D_4__Caulobacteraceae;D_5__Phenylobacterium	1155445972891.61(0.00,Inf)	0.00(0.00,Inf)	2.16(0.14,32.35)	1017000808677164.00(0.00,Inf)	0.00(0.00,Inf)	2.37(0.15,36.70)			
ASV_1777	Firmicutes;D_2__Clostridia;D_3__Clostridiales;D_4__Lachnospiraceae	1.90(0.02,173.39)	1.22(0.01,104.54)	2.32(0.07,78.79)						
ASV_1782	Firmicutes;D_2__Clostridia;D_3__Clostridiales;D_4__Lachnospiraceae;D_5__Lachnoclostridium	180030511997.96(0.00,Inf)	0.00(0.00,Inf)	0.66(0.02,27.26)						
ASV_1801	Firmicutes;D_2__Clostridia;D_3__Clostridiales;D_4__Lachnospiraceae;D_5__Lachnospiraceae NC2004 group;D_6__uncultured bacterium;D_7__;D_8__;D_9__;D_10__;D_11__;D_12__;D_13__;D_14__	0.00(0.00,Inf)	1.72(0.03,88.65)	0.00(0.00,Inf)						
ASV_1818	Bacteroidetes;D_2__Bacteroidia;D_3__Bacteroidales;D_4__Bacteroidaceae;D_5__Bacteroides	965332964.06(0.00,Inf)	0.00(0.00,Inf)	0.35(0.02,6.91)	267806494831 83.30(0.00,Inf)	0.00(0.00,Inf)	0.41(0.03,6.71)			
ASV_1820	Bacteroidetes;D_2__Bacteroidia;D_3__Bacteroidales;D_4__Bacteroidaceae;D_5__Bacteroides	101895226143.82(0.00,Inf)	0.00(0.00,Inf)	1.77(0.06,56.00)						
ASV_1863	Firmicutes;D_2__Clostridia;D_3__Clostridiales;D_4__Ruminococcaceae;D_5__Ruminiclostridium 5;D_6__uncultured bacterium;D_7__;D_8__;D_9__;D_10__;D_11__;D_12__;D_13__;D_14__	1.65(0.06,43.42)	0.62(0.03,14.46)	1.02(0.10,10.58)	3.85(0.09,161.94)	0.30(0.01,11.32)	1.17(0.12,11.85)			
ASV_1873	Firmicutes;D_2__Clostridia;D_3__Clostridiales;D_4__Ruminococcaceae;D_5__Ruminococcaceae UCG-003	0.22(0.00,10.84)	0.26(0.01,10.31)	0.06(0.00,1.28)						
ASV_1878	Firmicutes;D_2__Clostridia;D_3__Clostridiales;D_4__Ruminococcaceae;D_5__Subdoligranulum	0.96(0.02,47.93)	1.65(0.04,76.38)	1.58(0.07,37.83)						
ASV_1880	Actinobacteria;D_2__Coriobacteriia;D_3__Coriobacteriales;D_4__Coriobacteriaceae;D_5__Collinsella	2117108757.05(0.00,Inf)	0.00(0.00,Inf)	1.06(0.06,18.03)						
ASV_1881	Firmicutes;D_2__Clostridia;D_3__Clostridiales;D_4__Lachnospiraceae;D_5__[Eubacterium] hallii group	0.12(0.00,4.88)	4348006756090.08(0.00,Inf)	518368458889.97(0.00,Inf)						
ASV_1895	Firmicutes;D_2__Clostridia;D_3__Clostridiales;D_4__Peptostreptococcaceae;D_5__Intestinibacter;D_6__uncultured bacterium;D_7__;D_8__;D_9__;D_10__;D_11__;D_12__;D_13__;D_14__	0.37(0.01,22.86)	2.78(0.06,140.21)	1.04(0.03,33.28)						
ASV_1896	Actinobacteria;D_2__Coriobacteriia;D_3__Coriobacteriales;D_4__Coriobacteriaceae;D_5__Collinsella	0.53(0.02,13.94)	2.69(0.11,64.39)	1.44(0.09,23.31)						
ASV_1899	Firmicutes;D_2__Clostridia;D_3__Clostridiales;D_4__Lachnospiraceae;D_5__Tyzzerella;D_6__uncultured Firmicutes bacterium;D_7__;D_8__;D_9__;D_10__;D_11__;D_12__;D_13__;D_14__				0.00(0.00,Inf)	5.33(0.15,195.70)	0.00(0.00,Inf)			

ASV_1989	Firmicutes;D_2__Clostridia;D_3__Clostridiales;D_4__Ruminococcaceae;D_5__Faecalibacterium	0.31(0.01,8.87)	18.21(0.49,677.80)	5.59(0.22,143.15)	2.38(0.08,72.26)	7.98(0.15,427.46)	18.97(0.50,713.45)			
ASV_1991	Firmicutes;D_2__Clostridia;D_3__Clostridiales;D_4__Ruminococcaceae;D_5__Faecalibacterium;D_6__uncultured bacterium;D_7__;D_8__;D_9__;D_10__;D_11__;D_12__;D_13__;D_14__				0.00(0.00,Inf)	4.77(0.13,177.49)	0.00(0.00,Inf)			
ASV_2054	Firmicutes;D_2__Clostridia;D_3__Clostridiales;D_4__Lachnospiraceae;D_5__Coprococcus 3				0.00(0.00,Inf)	91082681259069.80(0.00,Inf)	1.00(0.00,Inf)			
ASV_2058	Firmicutes;D_2__Clostridia;D_3__Clostridiales;D_4__Ruminococcaceae;D_5__Faecalibacterium;D_6__uncultured bacterium;D_7__;D_8__;D_9__;D_10__;D_11__;D_12__;D_13__;D_14__	0.00(0.00,Inf)	2.33(0.06,88.47)	0.00(0.00,Inf)						
ASV_2059	Firmicutes;D_2__Clostridia;D_3__Clostridiales;D_4__Lachnospiraceae	0.00(0.00,Inf)	4.96(0.22,113.00)	0.00(0.00,Inf)						
ASV_2067	Bacteroidetes;D_2__Bacteroidia;D_3__Bacteroidales;D_4__Bacteroidaceae;D_5__Bacteroides;D_6__uncultured bacterium;D_7__;D_8__;D_9__;D_10__;D_11__;D_12__;D_13__;D_14__	0.00(0.00,Inf)	1.83(0.06,57.76)	0.00(0.00,Inf)						
ASV_2071	Firmicutes;D_2__Clostridia;D_3__Clostridiales;D_4__Lachnospiraceae	0.00(0.00,Inf)	1.35(0.03,58.91)	0.00(0.00,Inf)						
ASV_2072	Firmicutes;D_2__Clostridia;D_3__Clostridiales;D_4__Clostridiaceae 1;D_5__Clostridium sensu stricto 1;D_6__uncultured bacterium;D_7__;D_8__;D_9__;D_10__;D_11__;D_12__;D_13__;D_14__	0.00(0.00,Inf)	4.56(0.26,80.89)	0.00(0.00,Inf)	0.00(0.00,Inf)	2.90(0.17,48.79)	0.00(0.00,Inf)			
ASV_2081	Firmicutes;D_2__Clostridia;D_3__Clostridiales;D_4__Lachnospiraceae;D_5__Agathobacter;D_6__uncultured bacterium;D_7__;D_8__;D_9__;D_10__;D_11__;D_12__;D_13__;D_14__	0.00(0.00,Inf)	0.33(0.01,7.30)	0.00(0.00,Inf)	0.00(0.00,Inf)	0.17(0.01,4.19)	0.00(0.00,Inf)			
ASV_2104	Firmicutes;D_2__Clostridia;D_3__Clostridiales;D_4__Lachnospiraceae;D_5__Lachnospiraceae UCG-004	0.70(0.02,21.78)	5.30(0.13,212.90)	3.72(0.14,96.61)						
ASV_2109	Firmicutes;D_2__Clostridia;D_3__Clostridiales;D_4__Ruminococcaceae;D_5__Butyricicoccus;D_6__uncultured bacterium;D_7__;D_8__;D_9__;D_10__;D_11__;D_12__;D_13__;D_14__	2.32(0.03,167.57)	0.43(0.01,26.43)	0.99(0.04,22.85)						
ASV_2116	Firmicutes;D_2__Clostridia;D_3__Clostridiales;D_4__Lachnospiraceae;D_5__Lactonifactor	3078168944.57(0.00,Inf)	0.00(0.00,Inf)	0.80(0.05,13.97)						
ASV_2134	Firmicutes;D_2__Clostridia;D_3__Clostridiales;D_4__Lachnospiraceae;D_5__Oribacterium;D_6__Oribacterium sinus;D_7__;D_8__;D_9__;D_10__;D_11__;D_12__;D_13__;D_14__	1.06(0.04,25.61)	1.25(0.06,27.36)	1.33(0.11,16.40)	1.27(0.04,37.59)	1.64(0.05,52.57)	2.08(0.17,26.13)			
ASV_2142	Actinobacteria;D_2__Actinobacteria;D_3__Corynebacteriales;D_4__Nocardiaceae;D_5__Rhodococcus				0.46(0.00,Inf)	0.00(0.00,Inf)	0.00(0.00,Inf)			
ASV_2149	Epsilonbacteraeota;D_2__Campylobacteria;D_3__Campylobacterales;D_4__Campylobacteraceae;D_5__Campylobacter	0.21(0.01,8.37)	1.19(0.04,32.88)	0.25(0.01,5.13)	0.08(0.00,4.06)	2.24(0.07,70.00)	0.18(0.01,4.28)			
ASV_2150	Firmicutes;D_2__Clostridia;D_3__Clostridiales;D_4__Lachnospiraceae;D_5__Blautia	1727133506.45(0.00,Inf)	0.00(0.00,Inf)	1.18(0.03,45.13)						
ASV_2195	Firmicutes;D_2__Clostridia;D_3__Clostridiales;D_4__Ruminococcaceae;D_5__Subdoligranulum	180489721935.71(0.00,Inf)	0.00(0.00,Inf)	0.19(0.01,6.38)						
ASV_2219	Firmicutes;D_2__Bacilli;D_3__Bacillales;D_4__Alicyclobacillaceae;D_5__Effusibacillus;D_6__uncultured organism;D_7__;D_8__;D_9__;D_10__;D_11__;D_12__;D_13__;D_14__	36213126583.21(0.00,Inf)	0.00(0.00,Inf)	0.29(0.01,13.91)						
ASV_2236	Firmicutes;D_2__Clostridia;D_3__Clostridiales;D_4__Lachnospiraceae;D_5__Lachnospiraceae NC2004 group;D_6__uncultured bacterium;D_7__;D_8__;D_9__;D_10__;D_11__;D_12__;D_13__;D_14__	24376802887.30(0.00,Inf)	0.00(0.00,Inf)	0.55(0.03,9.77)						
ASV_2240	Firmicutes;D_2__Clostridia;D_3__Clostridiales;D_4__Ruminococcaceae;D_5__Ruminiclostridium;D_6__metagenome;D_7__;D_8__;D_9__;D_10__;D_11__;D_12__;D_13__;D_14__	116755936184.06(0.00,Inf)	0.00(0.00,Inf)	1.11(0.07,17.86)	275872810192885.00(0.00,Inf)	0.00(0.00,Inf)	0.46(0.02,9.13)			

ASV_2241	Proteobacteria;D_2__Gammaproteobacteria;D_3__Betaproteobacteriales;D_4__Burkholderiaceae;D_5__Lautropia;D_6__uncultured bacterium;D_7__;D_8__;D_9__;D_10__;D_11__;D_12__;D_13__;D_14__	0.10(0.00,6.97)	24.07(0.34,1694.96)	2.36(0.05,109.94)						
ASV_2265	Firmicutes;D_2__Clostridia;D_3__Clostridiales;D_4__Lachnospiraceae;D_5__[Ruminococcus] torques group;D_6__Ruminococcus sp. AT10;D_7__;D_8__;D_9__;D_10__;D_11__;D_12__;D_13__;D_14__	0.24(0.01,5.22)	3.70(0.21,65.39)	0.90(0.06,12.92)						
ASV_2273	Firmicutes;D_2__Clostridia;D_3__Clostridiales;D_4__Ruminococcaceae;D_5__uncultured;D_6__uncultured bacterium;D_7__;D_8__;D_9__;D_10__;D_11__;D_12__;D_13__;D_14__				6.51(0.07,596.36)	0.36(0.00,29.23)	2.36(0.10,55.48)			
ASV_2343	Bacteroidetes;D_2__Bacteroidia;D_3__Bacteroidales;D_4__Bacteroidaceae;D_5__Bacteroides	0.29(0.00,42.99)	1.98(0.02,221.63)	0.58(0.01,32.95)						
ASV_2480	Firmicutes;D_2__Clostridia;D_3__Clostridiales;D_4__Ruminococcaceae;D_5__Flavonifractor;D_6__uncultured bacterium;D_7__;D_8__;D_9__;D_10__;D_11__;D_12__;D_13__;D_14__	0.51(0.01,27.18)	11.80(0.17,806.17)	6.03(0.15,245.78)						
ASV_2488	Firmicutes;D_2__Clostridia;D_3__Clostridiales;D_4__Ruminococcaceae;D_5__Flavonifractor;D_6__uncultured bacterium;D_7__;D_8__;D_9__;D_10__;D_11__;D_12__;D_13__;D_14__	1.55(0.03,91.57)	0.73(0.01,37.18)	1.13(0.05,25.49)						
ASV_2501	Firmicutes;D_2__Clostridia;D_3__Clostridiales	2049072338608.41(0.00,Inf)	0.00(0.00,Inf)	1.95(0.07,58.06)						
ASV_2557	Firmicutes;D_2__Clostridia;D_3__Clostridiales;D_4__Ruminococcaceae;D_5__Ruminococcaceae UCG-013	0.45(0.01,25.45)	4.23(0.08,225.66)	1.91(0.06,61.45)						
ASV_2611	Firmicutes;D_2__Clostridia;D_3__Clostridiales;D_4__Lachnospiraceae;D_5__[Ruminococcus] gauvreauii group;D_6__Ruminococcus gauvreauii;D_7__;D_8__;D_9__;D_10__;D_11__;D_12__;D_13__;D_14__	0.00(0.00,Inf)	2.98(0.08,113.28)	0.00(0.00,Inf)						
ASV_2665	Patescibacteria;D_2__Saccharimonadia;D_3__Saccharimonadales;D_4__TM7 phylum sp. oral clone FR058;D_5__;D_6__;D_7__;D_8__;D_9__;D_10__;D_11__;D_12__;D_13__;D_14__	0.00(0.00,Inf)	1.80(0.06,52.37)	0.00(0.00,Inf)						
ASV_2687	Actinobacteria;D_2__Actinobacteria;D_3__Actinomycetales;D_4__Actinomycetaceae;D_5__Actinomyces	0.00(0.00,Inf)	1.90(0.09,41.26)	0.00(0.00,Inf)	0.00(0.00,Inf)	2.35(0.08,72.04)	0.00(0.00,Inf)			
ASV_3357	Firmicutes;D_2__Clostridia;D_3__Clostridiales;D_4__Ruminococcaceae;D_5__uncultured	0.90(0.00,Inf)	0.00(0.00,Inf)	0.00(0.00,Inf)	0.70(0.00,Inf)	0.00(0.00,Inf)	0.00(0.00,Inf)			

Supplementary File: Gut microbiota network analysis

Supplementary Fig. 1: Annotated gut microbiota network analysis plots (genus-level) based on stool samples from paediatric-onset MS cases (DMD naïve and exposed), ADS and unaffected controls (Panels A-E, below)

Panel A. Unaffected controls (n=36)

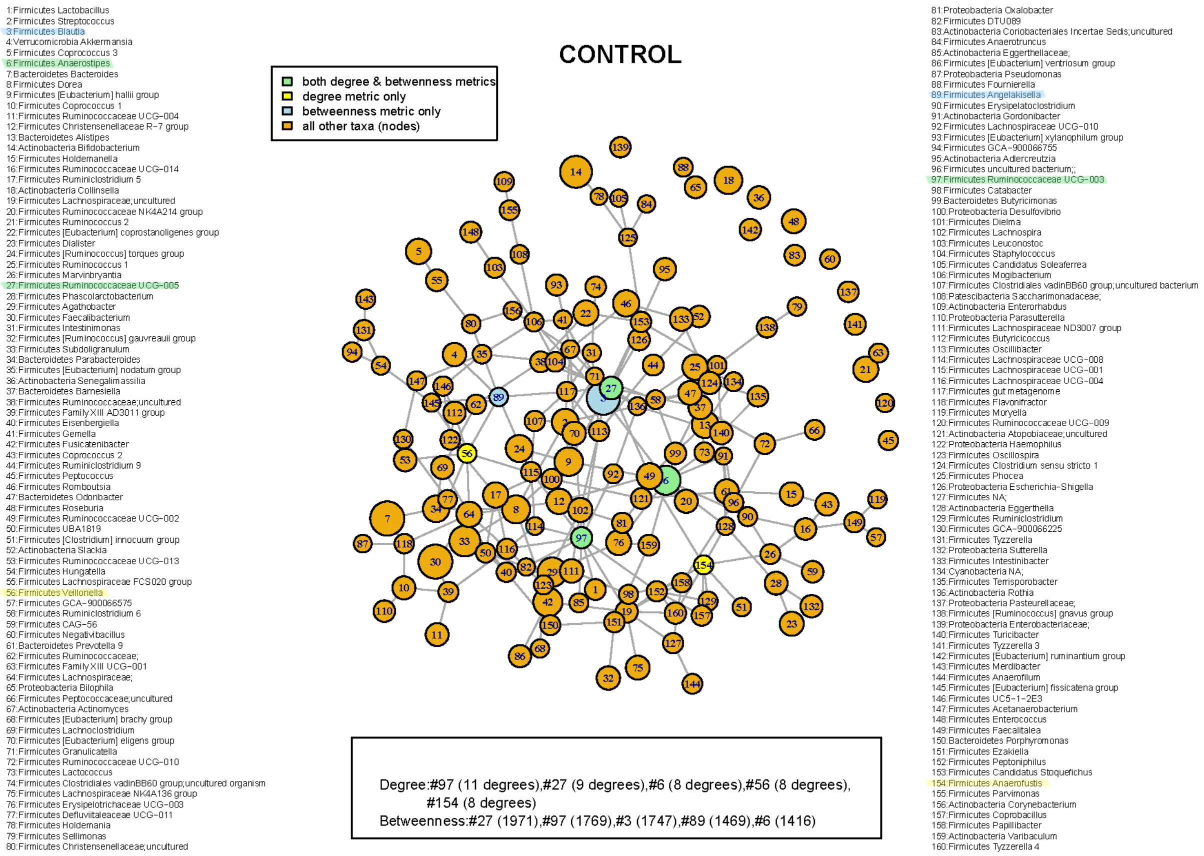

Panel B. ADS participants (n=41)

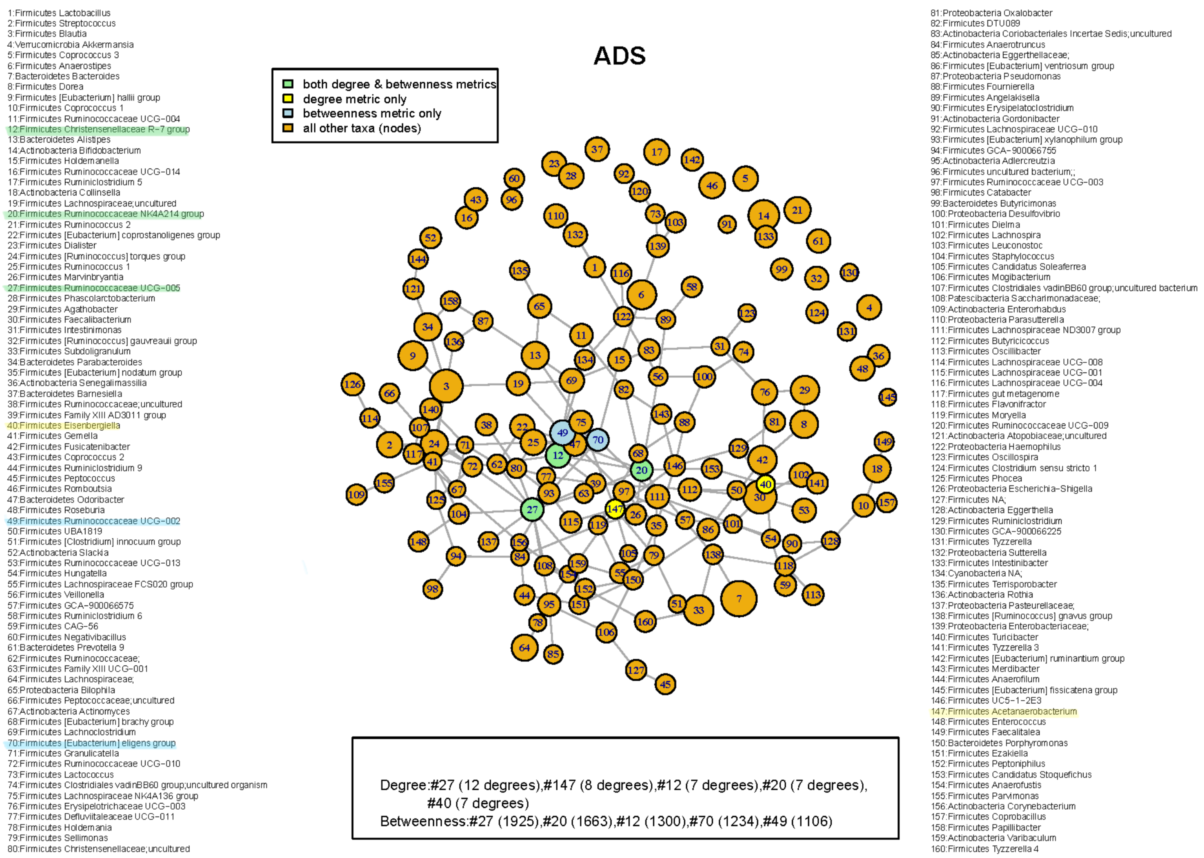

Panel C. All paediatric-onset MS cases (n=32)

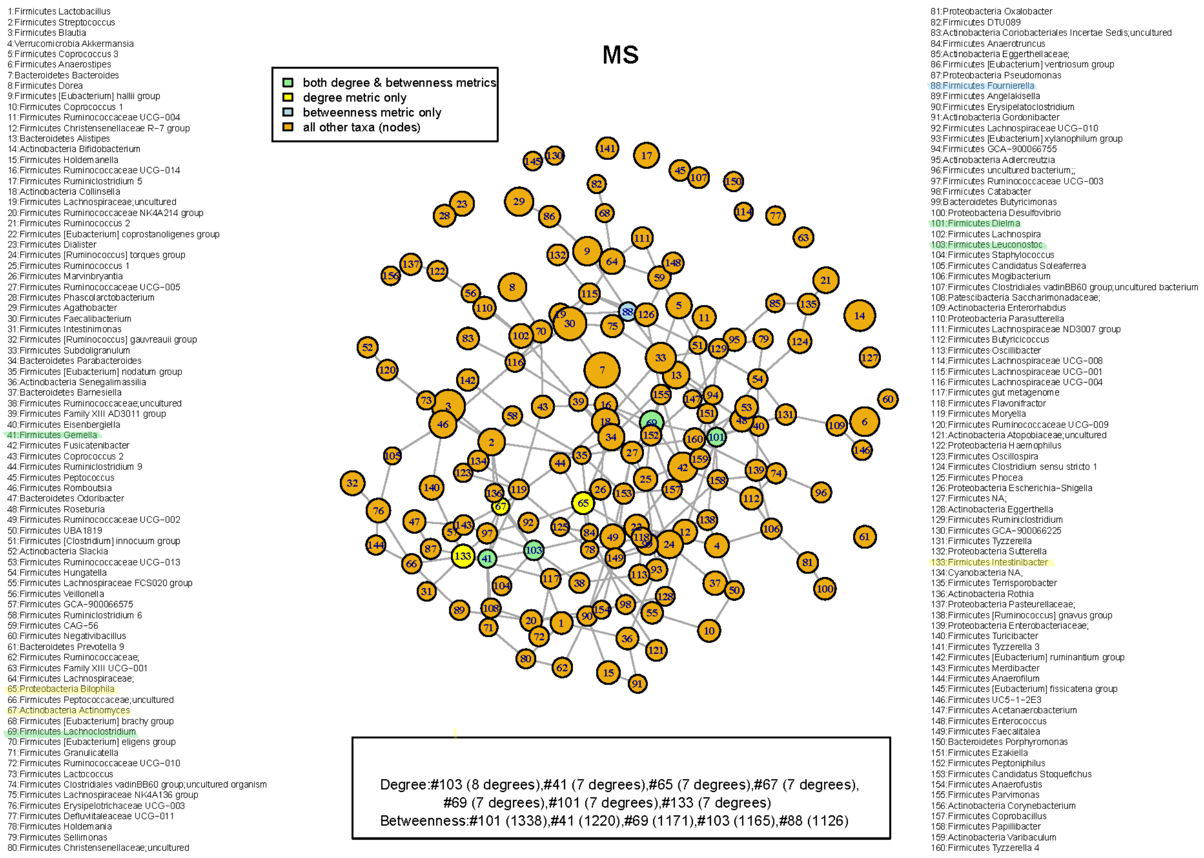

Panel D. DMD exposed MS cases only (n=23)

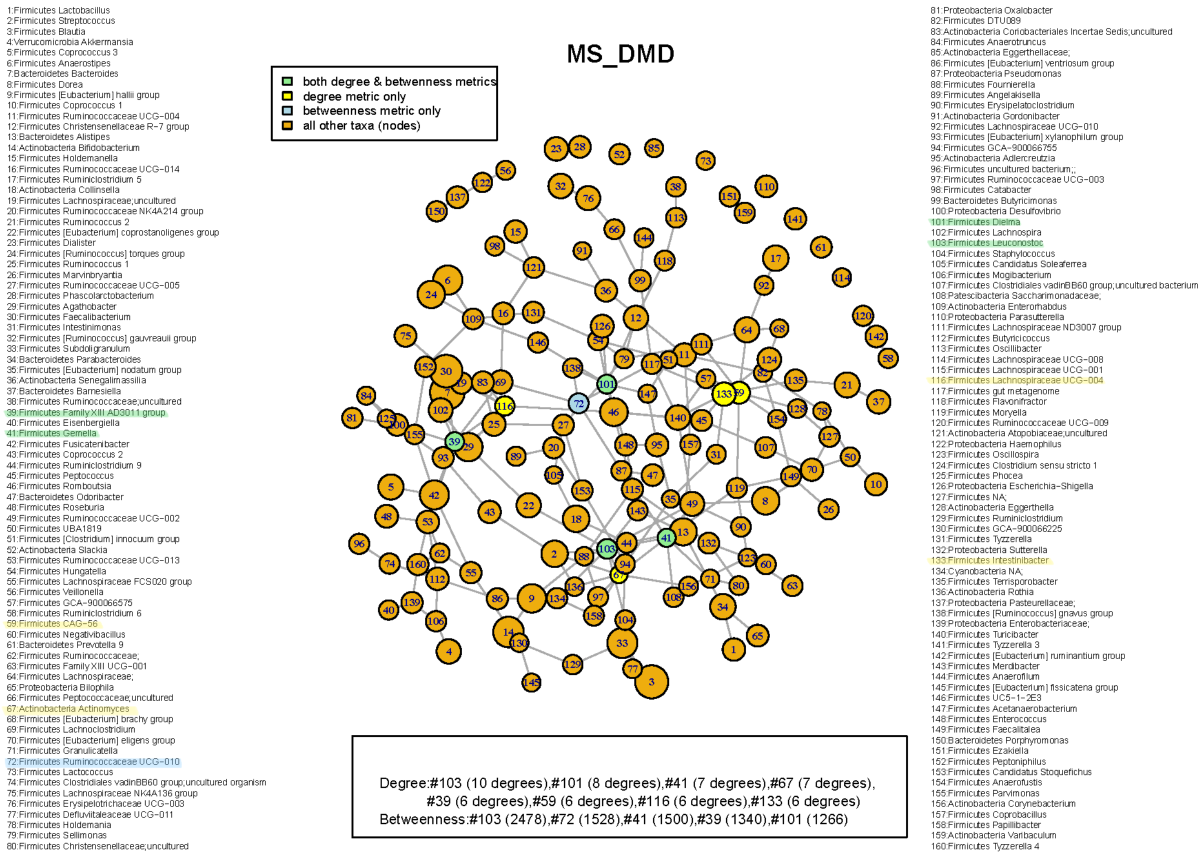

Panel E. DMD naïve MS cases only (n=9)

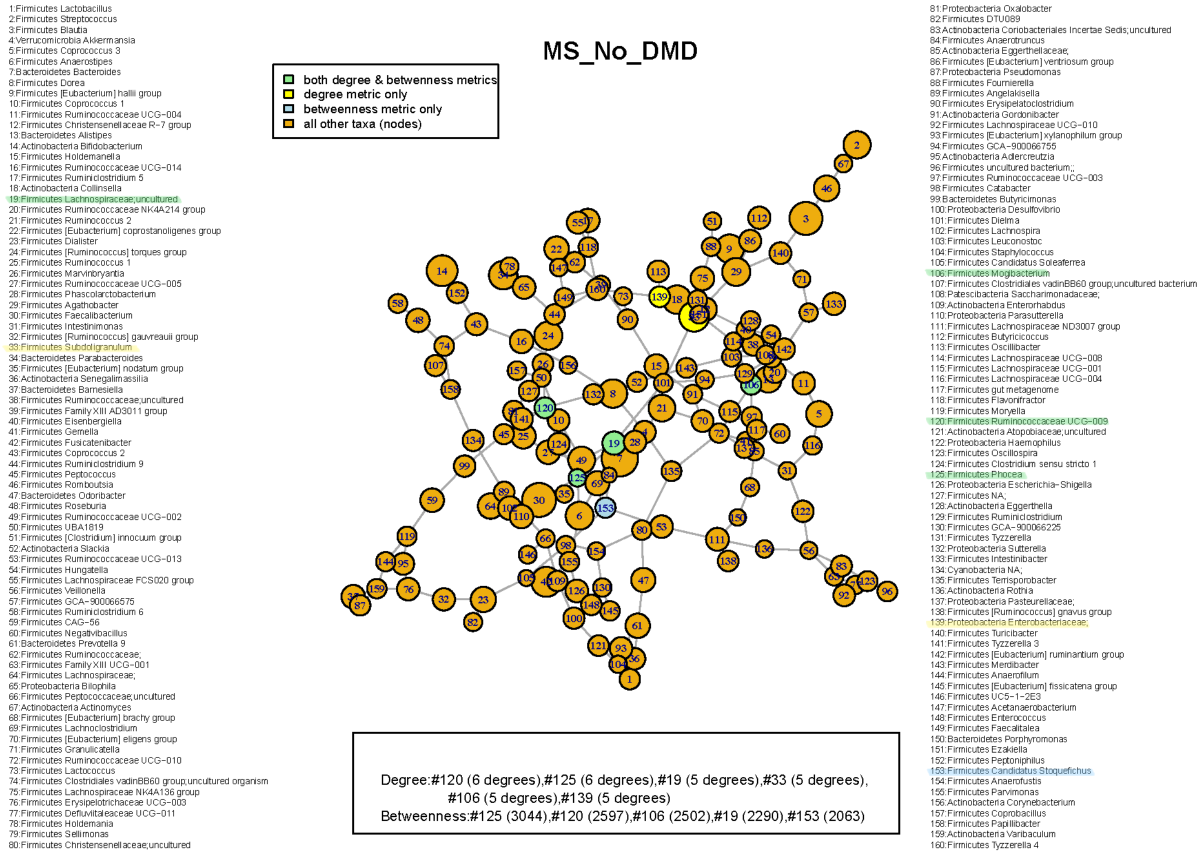

Key: Each sphere represents a node (taxa); sphere size represents the normalized counts (number of taxa). Lines show connectivity; longer lines indicate less connectivity.

The top 5 connected nodes (taxa) are highlighted (figure) and summarized in the Table (below), with more nodes highlighted if ties existed. Green highlight = taxa that were among the top 5 most connected node for both degrees and betweenness; Yellow = top 5 for degrees only; Blue = top 5 for betweenness only; orange = all other taxa

Boxed text at the foot of each Figure shows the top 5 connectivity metrics for degrees and betweenness with the corresponding nominally assigned taxa number (#). For example, in Panel C, for the MS participants, taxa #101 *Firmicutes Dielma* was one of the top five most connected taxa as measured by betweenness and degrees [betweenness metric = 1338 and degrees = 8; the latter indicating that this taxon was linked to 8 other nodes (taxa)].

ADS=acquired demyelinating syndromes, DMD=disease-modifying drug, MS=paediatric-onset multiple sclerosis; MS DMD/ MS no DMD =MS cases exposed/ naïve (ever / never) at the time of the stool sample.

Table Summary for Panels A-E: the top five connected taxa (genus-level) for MS cases (DMD exposed and naïve), ADS and unaffected control participants

Ordered alphabetically by *Phylum* and *Genus*

	**Top 5 connected taxa:** **number** indicates no. of connections **(degrees),** shown as lines in the Figures + indicates a top 5 connected taxa for **betweenness**						
**Nominal node (taxa) number**	***Phylum Genus***	**Controls**	**ADS**	**All MS cases**	***MS DMD exposed***	***MS DMD naïve***	**Remark/role & PubMed reference** (limited literature shown as '-')
67	***Actinobacteria*** *Actinomyces*			7	7		**Opportunistic pathogen**[1]
147	***Firmicutes*** *Acetanaerobacterium*		8				**Energy metabolism**[2]
154	*Firmicutes Anaerofustis*	8					**Little known**[3]
6	*Firmicutes Anaerostipes*	8+					**Short-chain fatty acid** producer (butyrate)
89	*Firmicutes Angelakisella*	+					**Recently identified & named (2017)**[4]
3	*Firmicutes Blautia*	+					**Short-chain fatty acid** producer (butyrate and acetic acid) **Anti-inflammatory;** depletion associated with metabolic syndrome & gut inflammation in children[5]
153	*Firmicutes Candidatus Stoquefichus*					+	**Little known;** provisional name assigned, recognized by the International Code

							of Nomenclature of Prokaryotes as an unculturable taxa[6]
59	*Firmicutes CAG-56*				*6*		**Little known;** uncultured, unnamed
12	*Firmicutes Christensenellaceae R-7 grp*		7+				*Christensenellaceae* considered highly heritable[7]
101	*Firmicutes Dielma*			7+	*8+*		**Link with progression, other diseases** [kidney] (not found in controls) PMID: 30088332
40	*Firmicutes Eisenbergiella*		7				**Considered metabolically important &highly connected gut community member**[8]
70	*Firmicutes [Eubacterium] eligens group*		+				**Little known;** uncultured relative of *Roseburia*, *in silico* study suggested enhanced by pectin[9]
39	*Firmicutes Family XIII AD3011 group*				*6+*		**Little known** in humans; some emerging data in domesticated animals only
88	*Firmicutes Fournierella*			+			**Little known;** member of recently identified & named taxa (2017)[10]
41	*Firmicutes Gemella*			7+	*7+*		**Opportunistic pathogen**[11-13]
133	*Firmicutes Intestinibacter*			7	*6*		**Recently re-named** (from genus Clostridium) **Highly influenced by risk genes (HLA haplotypes)** involved in other immune-mediated diseases.[14]
69	*Firmicutes Lachnoclostridium*			7+			**Little known**; recently identified/named (2013)[15]
116	*Firmicutes Lachnospiraceae UCG-004*				*6*		**Considered 'controversial' in human health – taxa include short-chain fatty acid** producers (butyrate) but also associated with disease, e.g., metabolic disease via incr extraction of energy from diet[16]
19	*Firmicutes Lachnospiraceae; uncult.*					*5+*	**As above** (little known; uncultured/unnamed)[16]
103	*Firmicutes Leuconostoc*			8+	*10+*		**Opportunistic pathogen vancomycin resistance**[17]
106	*Firmicutes Mogibacterium*					*5+*	**Newly identified**[17]
125	*Firmicutes Phocea*					*6+*	**Newly identified**, link with **metabolic syndromes**[18]
20	*Firmicutes Ruminococcaceae NK4A214 group*		7+				**Short-chain fatty acid** producing family (butyrate)

49	*Firmicutes Ruminococcaceae UCG−002*		+				*As above*
97	*Firmicutes Ruminococcaceae UCG-003*	11+					*As above*
27	*Firmicutes Ruminococcaceae UCG-005*	9+	12+				*As above*
120	*Firmicutes Ruminococcaceae UCG-009*					*6+*	*As above*
72	*Firmicutes Ruminococcaceae UCG−010*				+		*As above*
33	*Firmicutes Subdoligranulum*					*5*	**Newly identified**[19]
56	*Firmicutes Veillonella*	8					**Short-chain fatty acid** producer (lactate fermenter, to propionate & acetate)
65	***Proteobacteria*** *Bilophila*			7			**Bile-tolerant; can be modified by diet** ↑ during animal-based diet[20] ↓prebiotic diet (inulin)[21] ↑associated with immune-mediated disease[22]
139	*Proteobacteria Enterobacteriaceae*					*5*	**Opportunistic pathogens** & antibiotic resistant plasmids[23]
Overlap: Top five nodes for both betweenness & degree		3	3	4	*4*	*4*	

References

1. Könönen E, Wade WG. Actinomyces and related organisms in human infections. *Clin Microbiol Rev*. Apr 2015;28(2):419-42. doi:10.1128/cmr.00100-14
2. Painold A, Mörkl S, Kashofer K, et al. A step ahead: Exploring the gut microbiota in inpatients with bipolar disorder during a depressive episode. *Bipolar Disord*. Feb 2019;21(1):40-49. doi:10.1111/bdi.12682
3. Masoodi I, Alshanqeeti AS, Ahmad S, et al. Microbial dysbiosis in inflammatory bowel diseases: results of a metagenomic study in Saudi Arabia. *Minerva Gastroenterol Dietol*. Sep 2019;65(3):177-186. doi:10.23736/s1121-421x.19.02576-5
4. Mailhe M, Ricaboni D, Vitton V, Cadoret F, Fournier PE, Raoult D. 'Angelakisella massiliensis' gen. nov., sp. nov., a new bacterial species isolated from human ileum. *New Microbes New Infect*. Mar 2017;16:51-53. doi:10.1016/j.nmni.2017.01.003
5. Benítez-Páez A, Gómez del Pugar EM, López-Almela I, Moya-Pérez Á, Codoñer-Franch P, Sanz Y. Depletion of Blautia Species in the Microbiota of Obese Children Relates to Intestinal Inflammation and Metabolic Phenotype Worsening. *mSystems*. 2020;5(2):e00857-19. doi:10.1128/mSystems.00857-19

6. Oren A, Garrity GM, Parker CT, Chuvochina M, Trujillo ME. Lists of names of prokaryotic Candidatus taxa. *Int J Syst Evol Microbiol*. Jul 2020;70(7):3956-4042. doi:10.1099/ijsem.0.003789
7. Waters JL, Ley RE. The human gut bacteria Christensenellaceae are widespread, heritable, and associated with health. *BMC Biol*. Oct 28 2019;17(1):83. doi:10.1186/s12915-019-0699-4
8. Li X, Brejnrod AD, Ernst M, et al. Heavy metal exposure causes changes in the metabolic health-associated gut microbiome and metabolites. *Environ Int*. May 2019;126:454-467. doi:10.1016/j.envint.2019.02.048
9. Chung WS, Walker AW, Louis P, et al. Modulation of the human gut microbiota by dietary fibres occurs at the species level. *BMC Biol*. Jan 11 2016;14:3. doi:10.1186/s12915-015-0224-3
10. Togo AH, Durand G, Khelaifia S, et al. Fournierella massiliensis gen. nov., sp. nov., a new human-associated member of the family Ruminococcaceae. *Int J Syst Evol Microbiol*. May 2017;67(5):1393-1399. doi:10.1099/ijsem.0.001826
11. Desmottes MC, Brehier Q, Bertolini E, Monteiro I, Terreaux W. Septic arthritis of the knee due to Gemella morbillorum. *Int J Rheum Dis*. May 2018;21(5):1146-1147. doi:10.1111/1756-185x.13293
12. McQuinn M, Horswell BB. First Case of Cutaneous Orbital Abscess Caused by Gemella: A Case Report and Review of the Literature. *J Oral Maxillofac Surg*. Jul 2019;77(7):1414-1417. doi:10.1016/j.joms.2019.01.049
13. Maraki S, Plevritaki A, Kofteridis D, et al. Bicuspid aortic valve endocarditis caused by Gemella sanguinis: Case report and literature review. *J Infect Public Health*. May-Jun 2019;12(3):304-308. doi:10.1016/j.jiph.2019.01.001
14. Russell JT, Roesch LFW, Ördberg M, et al. Genetic risk for autoimmunity is associated with distinct changes in the human gut microbiome. *Nature communications*. Aug 9 2019;10(1):3621. doi:10.1038/s41467-019-11460-x
15. Yutin N, Galperin MY. A genomic update on clostridial phylogeny: Gram-negative spore formers and other misplaced clostridia. *Environmental microbiology*. Oct 2013;15(10):2631-41. doi:10.1111/1462-2920.12173
16. Vacca M, Celano G, Calabrese FM, Portincasa P, Gobbetti M, De Angelis M. The Controversial Role of Human Gut Lachnospiraceae. *Microorganisms*. 2020;8(4):573. doi:10.3390/microorganisms8040573
17. García-Granja PE, López J, Ladrón R, San Román JA. Infective Endocarditis Due to Leuconostoc Species. *Rev Esp Cardiol (Engl Ed)*. Jul 2018;71(7):592-594. doi:10.1016/j.rec.2017.04.013
18. Nakazawa F, Sato M, Poco SE, et al. Description of Mogibacterium pumilum gen. nov., sp. nov. and Mogibacterium vescum gen. nov., sp. nov., and reclassification of Eubacterium timidum (Holdeman et al. 1980) as Mogibacterium timidum gen. nov., comb. nov. *Int J Syst Evol Microbiol*. Mar 2000;50 Pt 2:679-688. doi:10.1099/00207713-50-2-679
19. Holmstrøm K, Collins MD, Møller T, Falsen E, Lawson PA. Subdoligranulum variabile gen. nov., sp. nov. from human feces. *Anaerobe*. Jun 2004;10(3):197-203. doi:10.1016/j.anaerobe.2004.01.004
20. David LA, Maurice CF, Carmody RN, et al. Diet rapidly and reproducibly alters the human gut microbiome. *Nature*. Jan 23 2014;505(7484):559-63. doi:10.1038/nature12820
21. Vandeputte D, Falony G, Vieira-Silva S, et al. Prebiotic inulin-type fructans induce specific changes in the human gut microbiota. *Gut*. Nov 2017;66(11):1968-1974. doi:10.1136/gutjnl-2016-313271
22. Ye Z, Zhang N, Wu C, et al. A metagenomic study of the gut microbiome in Behcet's disease. *Microbiome*. Aug 4 2018;6(1):135. doi:10.1186/s40168-018-0520-6
23. Rozwandowicz M, Brouwer MSM, Fischer J, et al. Plasmids carrying antimicrobial resistance genes in Enterobacteriaceae. *J Antimicrob Chemother*. May 1 2018;73(5):1121-1137. doi:10.1093/jac/dkx488
